# MALDI Matrix: Origins, Innovations, and Frontiers

**DOI:** 10.1021/acs.chemrev.5c00786

**Published:** 2026-02-03

**Authors:** Ran Wu, Rui Liu, Hao Hu, Liang Qin, Lulu Chen, Zhibin Bao, Jinxiang Fu, Hua Guo, Lei Wang, Anna Wang, Zihan Wang, Chenyu Yang, Xiangrui Cheng, Difan Chen, Haiqiang Liu, Yanping Jing, Shuai Guo, Yujie Fu, Xiaodong Wang

**Affiliations:** † College of Biological Sciences and Biotechnology, 12380 Beijing Forestry University, #35 Qinghua East Road, Haidian District Beijing 100083, China; ‡ College of Life and Environmental Sciences, 12505 Minzu University of China, #27 Zhongguancun South Avenue, Beijing 100081, China; § State Key Laboratory of Natural and Biomimetic Drugs School of Pharmaceutical Sciences, Peking University, Beijing 100191, China; ∥ HIT Research Center, Beijing 100094, China; ⊥ State Key Laboratory of Biotherapy and Cancer Center, West China Hospital, Sichuan University, Chengdu 610064, China; # Xiangtan Environmental Science Research Institute, Xiangtan 411100, China; ∇ School of Basic Medicine and Clinical Pharmacy, China Pharmaceutical University, Nanjing 210009, China; ○ State Key Laboratory of Efficient Production of Forest Resources, Beijing Forestry University, #35 Qinghua East Road, Haidian District, Beijing 100083, China

## Abstract

Matrix-assisted laser desorption/ionization mass spectrometry (MALDI-MS) and its imaging variant (MALDI-MSI) are pivotal analytical tools for biological sample analysis, allowing detection and visualization of diverse biomolecules. At the heart of MALDI-MS/MSI performance lies the crucial yet often empirically determined choice of matrix. A suitable matrix not only boosts analyte ionization efficiency but also enhances sensitivity, salt tolerance, and overall applicability, which are key for specific compound detection. To date, a cumulative total of 467 matrices have been successfully discovered and developed. However, merely a handful of them have achieved widespread utilization, which strongly suggests that there remains a substantial reservoir of untapped potential within this domain. This review offers a systematic and comprehensive overview of MALDI matrices over four decades (1985–present). It starts by outlining MALDI-MS analysis principles and procedures, providing a basis for understanding matrix functions. Then, matrices are systematically classified according to their features. We also spotlight recent matrix applications in MALDI-MS detection and imaging in proteomics, lipidomics, metabolomics, glycomics, nucleic acid analysis, and quantitative analysis. Finally, we chart future research directions, aiming to unlock the full potential of matrices in this dynamic field.

## Graphical abstract

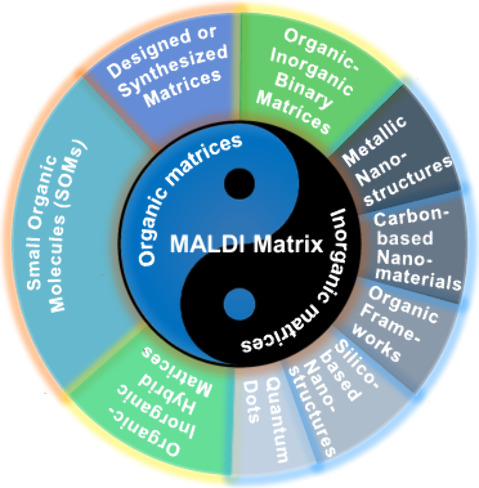

## 1 Introduction

### 1.1 History of MALDI-MS

Mass spectrometry (MS) has emerged as a powerful tool in the field of analytical chemistry, enabling scientists to unravel the complexities of molecular structures, elucidate chemical compositions, and gain profound insights into biological processes.^1^ To date, 13 Nobel laureates have significantly contributed to the birth, progression, and applications of mass spectrometry (Figure 1). The origins of MS trace back to the late 19th century, with simpler forms of the technique.^2^ Pioneering work was laid by Eugen Goldstein’s discovery of canal rays,^3^ Wilhelm Wien’s use of electric and magnetic fields to separate positive rays based on their charge-to-mass ratio (*q/m*),^4^ Joseph John Thomson’s exploration of the composition of positive rays (positive ions),^5^ *etc*. Building on this foundation, Francis William Aston developed the first fully functional mass spectrometer, which led to the discovery of isotopes in a vast array of nonradioactive elements and the elucidation of the whole number rule,^6,7^ for which he was awarded the Nobel Prize in 1922. In 1932, Harold Clayton Urey identified the isotopes of the smallest atom, hydrogen, and discovered heavy hydrogen, later named deuterium.^8,9^ In structural studies of molecules using MS, ionization is a central step: only after ionization can molecules be separated and analyzed in an electric or magnetic field according to their mass to charge ratios (*m*/*z*).^10^ Ion generation takes place in the gas phase following the evaporation/desorption of samples under vacuum conditions. While suitable for volatile organic molecules, this method is not applicable to biomolecules, which are highly polar and thermally labile, and thus undergo pyrolytic degradation upon heating. For decades, MS was therefore restricted to small, thermally stable biocompounds due to the lack of effective techniques for soft ionization and the transfer of ionized molecules from the condensed phase to the gas phase without excessive fragmentation.^11^ Between the 1960s and the 1980s, MS saw a surge in development, introducing alternative vacuum desorption/ionization (D/I) modes suitable for biomolecules. These approaches enabled fragile and low-molecular-weight (LMW) biomolecules to ionize without degradation during their transition to the gas phase, including field desorption (FD),^12,13^ fast atom bombardment (FAB),^14–16^ liquid secondary ion mass spectrometry (LSIMS),^17,18^ atmospheric pressure ionization (API),^19^ or atmospheric pressure chemical ionization (APCI),^20^ and electrohydrodynamic ionization.^21^ While effective for complex biochemical small molecules, these ionization techniques remain unsuitable for biological macromolecule analysis. The development and advancement of two soft ionization techniques between the 1960s and 1990s, *i.e.*, electrospray ionization (ESI)^22^ and matrix-assisted laser desorption/ionization (MALDI),^23,24^ were pivotal in maintaining the integrity of biomolecules, especially biopolymers. This enabled MS analysis of polypeptides (proteins) and even protein complexes, facilitating a historic leap from the study of hydrogen isotopes to biomolecules. In recognition, the 2002 Nobel Prize in Chemistry was awarded to two outstanding scientists, John B. Fenn (for ESI) and Koichi Tanaka (for MALDI) (Figure 1). Since their discovery, ESI and MALDI have become indispensable in proteomic analysis based on MS, with each method offering complementary strengths. The key difference between the MALDI and ESI ionization sources lies in the former producing low-charge ions (mono and doubly-charged),^25^ which simplifies molecular mass determination for most biomolecules and makes data interpretation easier compared to ESI-MS.^26^

**Figure 1:**
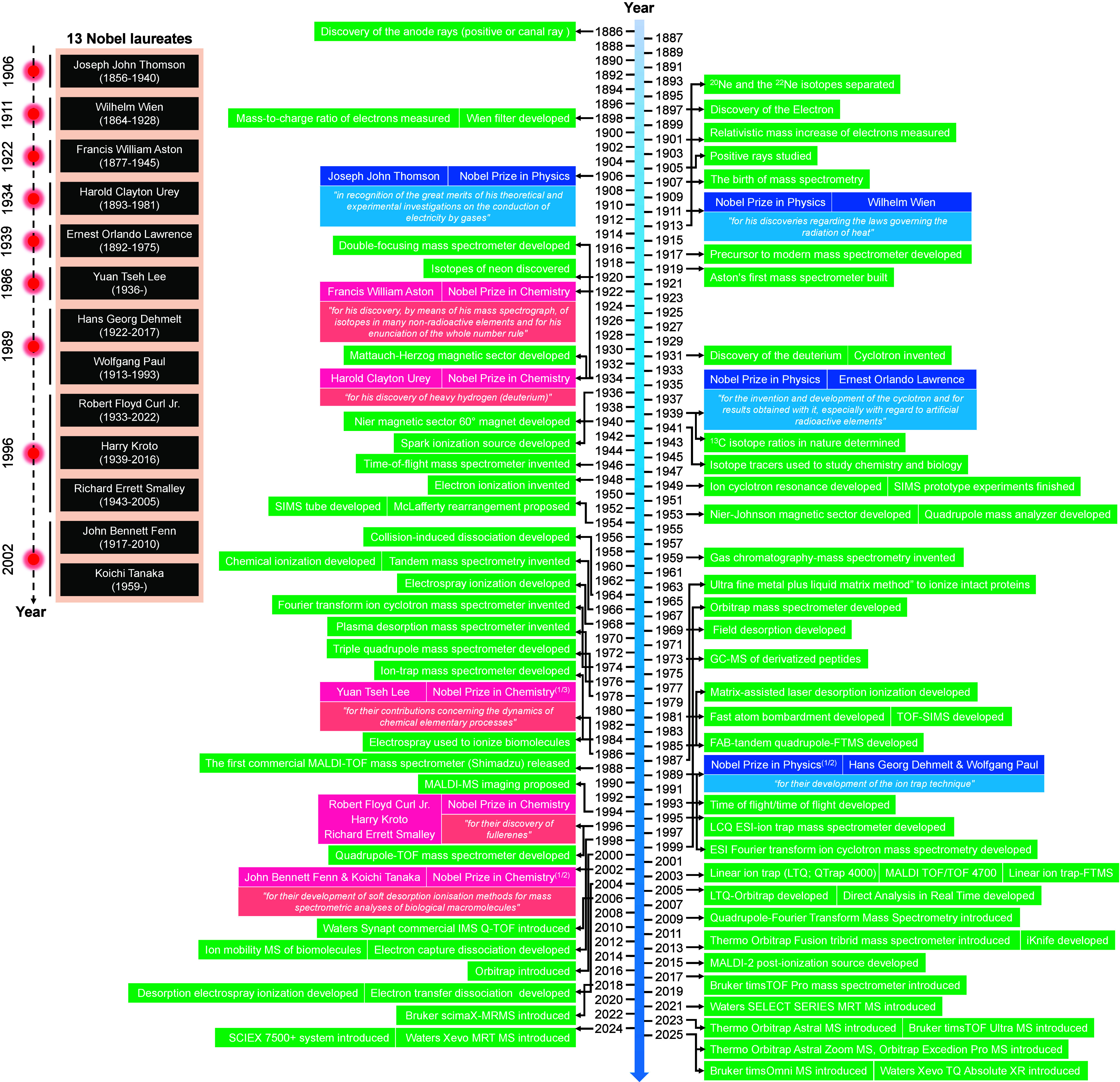
Timeline depicting key advancements in mass spectrometry and featuring 13 Nobel laureates that have significantly contributed to the birth, progression, and applications of mass spectrometry.

#### 1.1.1 MALDI-MS

MALDI-MS is a key technique for analyzing a wide variety of substances, ranging from biomolecules to polymers with molecular weights spanning several hundred to millions of daltons (Da). The terminology and concept of MALDI were introduced by Karas et al. in 1985, who discovered that in the presence of tryptophan, the ionization of alanine was enhanced at a laser wavelength of 266 nm.^27^ Subsequently, a breakthrough in laser D/I (LDI) of large molecules followed in 1987, when Tanaka et al. employed a fine metal powder matrix dispersed in glycerol with a pulsed N_2_ laser at 337 nm to successfully ionize proteins up to 34,000 Da.^24^ For this development of a soft desorption–ionization method enabling MS analysis of biological macromolecules, Koichi Tanaka was awarded the 2002 Nobel Prize in Chemistry. The advent of MALDI, together with advances in MS instrumentation, improving specificity, sensitivity, speed, sampling, and automated computer data acquisition/deconvolution, established MALDI-based MS as an essential tool for label-free detection of intact biomolecules (*e.g.*, proteins, DNA/RNA, carbohydrates, and lipids) in biological samples.^28–31^

#### 1.1.2 MS Imaging

The development of methods for detecting, identifying, and mapping the spatial localization of molecules using MS imaging (MSI, also known as imaging mass spectrometry) has extended the advantages of analytical MS to cellular and subcellular resolution, allowing detailed molecular localization of hundreds of molecules in biological tissues.^32^ MSI integrates molecular information from MS with spatial visualization of thin sample sections, generating images that reveal the distribution of specific molecules such as peptides, proteins, lipids, drugs, and metabolites.^33–35^ The concept of MS-based tissue imaging dates back to the early 1990s, initially using the well-established secondary ion MS (SIMS) method,^36^ followed by laser ablation MS.^37^ However, it was the introduction of MALDI imaging that enabled MSI for widespread biological and clinical applications.^31^ To date, the three main ionization methods for MSI are MALDI, desorption electrospray ionization (DESI), and SIMS (detailed information is provided in Table 1).^38–40^

**Table 1:** Characteristics of MALDI-MSI, DESI-MSI, and SIMS Imaging

	MALDI-MSI	DESI-MSI	SIMS imaging
ionization source	UV/IR laser	solvent spray	ion beam
ionization type	Soft ionization (low or absent in-source fragmentation of analyte ions)	soft ionization (low or absent in-source fragmentation of analyte ions)	hard ionization (formation of analyte ions accompanied by extensive in-source fragmentation)
ion adduct form	almost single charge ions (*e.g.* [M+H]^+^, [M+Na]^+^, [M+K]^+^, [M+NH_4_]^+^, [M–H]^−^, [M+Cl]^−^, [M+Na-2H]^−^, [M+K-2H]^−^, etc.)	single-charge ions (*e.g.* [M+H]^+^, [M+Na]^+^, [M+K]^+^, [M+NH_4_]^+^, [M–H]^−^, [M+Cl]^−^, [M+Br]^−^, etc.) and/or multiple-charged ions (*e.g.* [M+nH]^*n*+^, [M–nH]^*n*−^, etc.)	almost single charge ions (e.g. [M+H]^+^, [M–OH]^+^, [M+Na]^+^, [M+K]^+^, [M+Au]^+^, [M–H]^−^, etc.)
mass range (Da)	0–200,000	0–2,000	0–1,000

limit spatial resolution (μm)	5 (for commonly-used high vacuum MALDI source)	∼25–50	∼0.05–0.1
	1.4 (for atmospheric pressure MALDI source, AP-MALDI)		

mass analyzer	TOF/TOF, Q-TOF, FT (Orbitrap), ion mobility coupled to Q-TOF, QqQ	Q-TOF, FT (Orbitrap)	TOF
detecting limitation (sensitivity)	Fmol–amol range	Fmol range	Pmol–fmol range
sample preparation	dehydrated, homogeneous matrix coating	no preparation, analysis at atmospheric pressure	dehydrated, not fixed, no matrix
accessible compounds	metabolites, drugs, lipids, glycans, amino acid, peptides, proteins	metabolites, lipids, small peptides, proteins (partial)	elements, drugs, metabolites, lipids, small peptides

MALDI-MSI is an innovative, label-free technique that can generate two-dimensional (2D) ion density maps representing the distribution of analytes on the surface of tissue sections or cells, thereby providing a more thorough understanding of complex biological systems. This imaging technique was first described in 1994 at the 42^nd^ American Society for Mass Spectrometry (ASMS) Conference.^41^ In 1995, Gusev et al. demonstrated the approach using MALDI time-of-flight MS (MALDI-TOF-MS) as a thin-layer chromatography (TLC) detection system, producing images of small peptides (400–1,200 Da).^42^ In 1997, Caprioli and his colleagues further introduced the concept of MALDI-based MSI by directly obtaining images of biomolecules (proteins and peptides) from rat pituitary and colon tissues.^43^ Like DESI-MSI, MALDI-MSI is another soft ionization method. However, unlike DESI-MSI, MALDI-MSI typically produces singly-charged ions, significantly simplifying mass spectra and facilitating data analysis. The cluster model developed by Karas and colleagues offers a possible explanation for this phenomenon: during the laser-induced desorption process in MALDI-MS, multiply charged analyte ions undergo secondary neutralization reactions with free electrons, ultimately yielding singly charged analyte ions, often referred to as “lucky survivors”.^44^ Concurrently, neutral analyte molecules evaporate and acquire charge through secondary ionization via proton transfer from matrix ions in the plume. Compared with SIMS imaging, MALDI-MSI is relatively gentler, generating minimal fragmentation, and is therefore suitable for *in situ* detection and imaging of intact proteins. Although originally developed for the spatial analysis of polypeptides and proteins, MALDI-MSI now enables spatial analysis of a wide range of analytes including neurotransmitters (NTs), lipids, *N*-linked glycans, nucleotides, metabolites, and drugs (Table 1).^33–35^ Direct analysis of tissues or cells using MALDI-MSI enables the acquisition of abundance information of endogenous and exogenous compounds in tissues or cells while preserving their spatial distributions.^45,46^ MALDI-MSI provides distinct advantages in terms of high sensitivity, high throughput, and molecular specificity with the ability to detect *in situ* multiple biomolecules within a tissue section or cell.^31,46,47^ Thus far, MALDI-MSI has been applied to diverse fields, such as zoology, medical sciences, pharmaceutical sciences, microbiology, and plant science.

### 1.2 Historical Development of Matrices

In MALDI-MS, the matrix is an organic or inorganic compound that is added in large or excess amounts to the target sample or analytes. Its crucial role lies in absorbing laser energy, thereby facilitating the desorption and ionization of the analyte.^48^ Therefore, matrix selection is of utmost importance for the success of subsequent experiments. Different classes of biomolecules require different optimal matrices during their ionization processes, and matrix selection must be tailored according to the specific objectives of the experiment.^49,50^ Suitable matrices may be organic or inorganic compounds or mixtures of two or more matrices, designed to enhance the ionization efficiency of specific analyte classes. Since the introduction of MALDI, screening and developing new and more efficient matrices have become an essential and very important aspect for advancing the technique.^51–54^ The continuous expansion and innovative development of the MALDI matrix library have greatly broadened the application of MALDI-MS and MALDI-MSI in various analytical fields.^55,56^ To date, a total of 467 MALDI matrices have been successfully screened or developed and clearly reported. Among, 269 are organic matrices, 174 are inorganic matrices, and 24 fall into other categories (Figure 2). Overall, remarkable progress has been achieved in the study of MALDI matrices.

**Figure 2:**
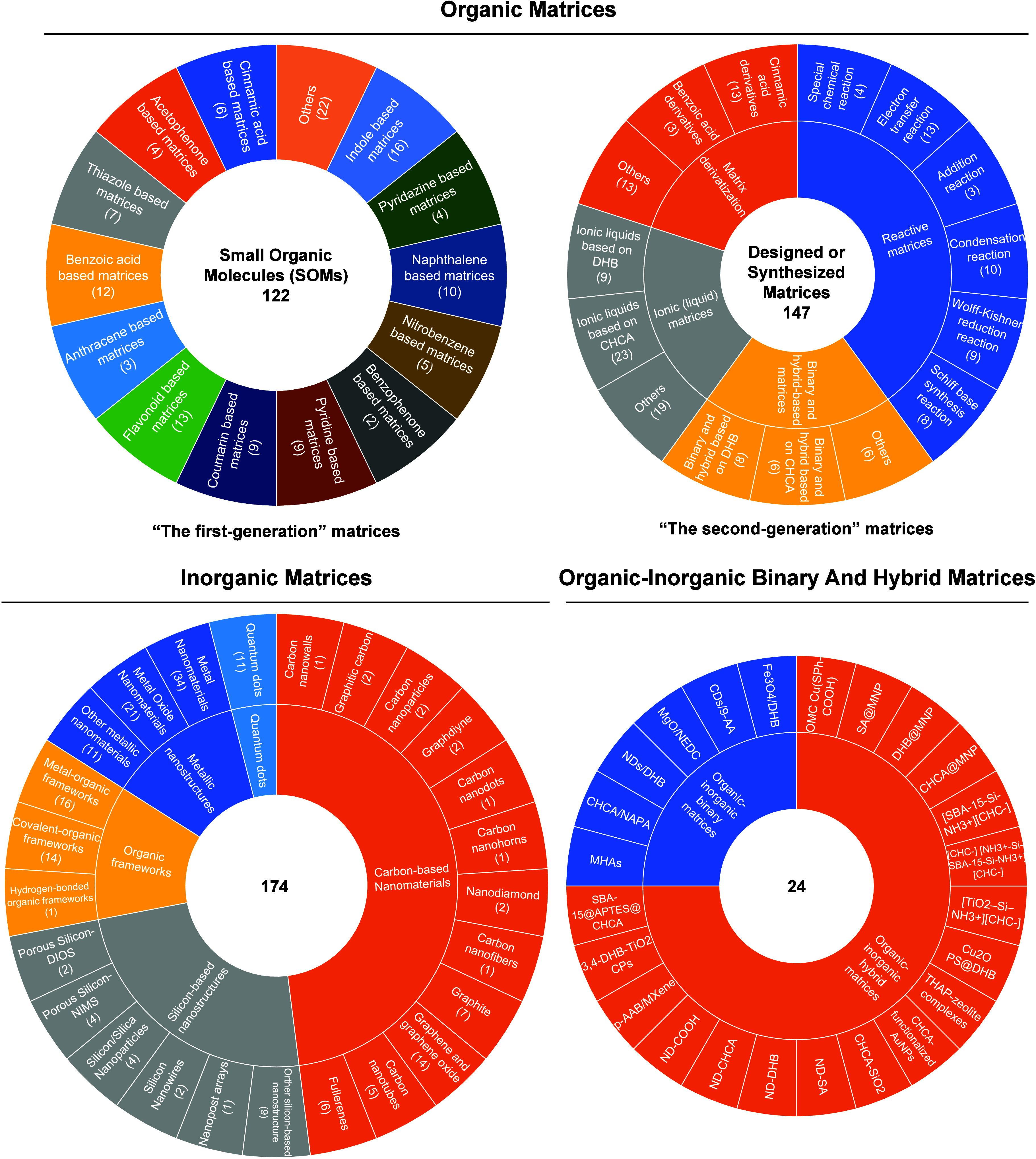
Overview diagram of MALDI matrices used for MS analysis.

#### 1.2.1 Historical Development of Organic Matrices

The development of organic matrices has progressed in parallel with advance in matrix-laser combinations. In 1987, Koichi Tanaka et al. introduced the “ultrafine metal plus liquid matrix method”, combining glycerol containing fine metal powders (30 nm cobalt nanoparticles (Co NPs)) with a 337 nm pulsed N_2_ laser for ionization. Using this matrix-laser combination, they successfully ionized biomolecules as large as the 34,472 Da protein carboxypeptidase-A.^24^ Subsequently, Karas and Hillenkamp used nicotinic acid (NA) as the matrix and a 266 nm Nd-YAG laser to successfully ionize and detect 67 kDa bovine protein albumin.^23^ Further improvements were achieved by coupling a 355 nm pulsed ultraviolet (UV) laser (Nd:YAG UV laser) with cinnamic acid derivatives such as sinapinic acid (SA), ferulic acid (FA), and caffeic acid (CA) as matrices, enabling higher-performance MALDI-MS detection of proteins.^57^ Traditional MALDI matrices are mainly composed of small organic molecules (SOMs). Aromatic compounds derived from benzoic acid^58^ or cinnamic acid^57,59^ have proven particularly effective as matrices for proteins, peptides, and lipids. In contrast, the compounds such as 3-hydroxycholanic acid (3-HPA)^60^ and succinic acid^61^ are more useful for the detection of small polymers and oligonucleotides. To meet different analytical requirements, the emergence of designed and synthesized matrices has effectively addressed some of the limitations and drawbacks of traditional SOMs matrices. For example, ionic liquid matrices (ILMs) improve matrix spot uniformity,^62,63^ reactive matrices enhance the ionization efficiency of target compounds,^64^ and binary matrices reduce the interfering background signals for LMW compounds.^57^ In general, we classify commonly-used SOMs as “first-generation” matrices, because most of them are natural compounds discovered empirically by screening hundreds of small molecules with high absorption coefficients at laser irradiation wavelengths. “Second-generation” matrices are rationally designed by altering the nature, number, and position of functional groups. In many cases, second-generation matrices outperform prototype (substrate) compounds in terms of crystallization uniformity, sensitivity, salt tolerance, high-vacuum stability, and laser energy absorption. It is noteworthy that the majority of matrices were discovered by chance. Table S1 provides an overview of 15 commonly-used organic matrices, detailing their characteristics, such as acid dissociation constant (p*K*_a_ values) and gas-phase basicity (GB, a thermochemical descriptor of proton-transfer affinity in the gas phase), along with their respective applications.

#### 1.2.2 Historical Development of Inorganic Matrices

To date, despite the great success of organic matrices, especially SOMs, their use in MALDI-MS still face several limitations:^65^ (i) interference from matrix-derived ions complicates the detection and identification of LMW compounds (*m*/*z* < 700); (ii) the lack of a universal matrix necessitates prior knowledge of analyte physicochemical properties; (iii) co-crystallization of the matrix and target analytes is often uneven hindering quantitative analysis; (iv) the “sweet-spot” effect is prone to occur, resulting in poor reproducibility of the detection signals. These challenges have prompted scientists to seek new alternative solutions, promoting the development of inorganic matrices. In 1995, Sunner et al. first introduced graphite particles in MALDI-MS, successfully detecting protonated ions and alkali cation adducts of the analytes.^66^ Since then, carbon-based materials developed from graphite have been widely used in the analysis of LMW compounds by MALDI-MS, such as graphite, graphene, carbon nanotubes, activated carbon powder, and fullerenes.^67–70^ In 1999, Wei et al. developed the D/I on silicon (DIOS) technique, employing porous silicon without a liquid matrix to ionize LMW compounds.^71^ This marked the entry of silicon-based materials in MALDI-MS technology. In the subsequent decades, silicon-based materials such as nano-silicon powders, nano-silicon cavities, nano-silicon wires, and porous silicon, were successively developed for MALDI-MS analysis.^72–75^ In addition to carbon- and silicon-based materials, other inorganic matrices have emerged, such as metal-based materials, organic framework nanocomposites, and quantum dots.^76–78^ Moreover, some functionalized inorganic materials can serve dual roles: as ionization matrices and as selective adsorbents to enrich target molecules, which greatly simplifies the analytical process, reduces analysis time, and effectively improves the MALDI-MS efficiency.^79^ Table S2 presents an overview of 10 commonly used inorganic matrices, detailing their size or structure, along with their respective applications.

### 1.3 Content Outline

Advances in MALDI matrices reflect both the rapid development of MALDI-MS technology and the continuous expansion of its application fields. However, it should be noted that only a very small number of matrices are widely used, most of which were established during the early stages of MALDI-MS. In fact, the majority of newly developed MALDI matrices have seen limited adoption, further widening the imbalance between matrix effectiveness and the growing analytical needs of researchers. Over time reliance on empirical matrix selection has become increasingly inefficient, as evidenced by the persistent difficulties practitioners face daily, regarding sensitivity, variable response factors, range of applicability, and reproducibility. Therefore, there is an urgent need for a systematic review and comprehensive summary of MALDI matrices to improve the scientific nature of matrix selection in MALDI-MS analysis, reduce inefficient empiricism, promote the development of new MALDI matrices, and fully enhance the analytical potential of MALDI-MS. To address the above-mentioned issues, we provide a comprehensive review of MALDI matrix development over the past 40 years (from 1985 to the present), offering a critical and authoritative perspective through a systematic summary of their continuous discovery, development, and improvement strategies. In addition, special emphasis is placed on the latest application progress of matrices in MALDI-MSI-based spatial omics including spatial proteomics, spatial metabolomics, spatial lipidomics, and spatial glycomics.

## 2 Matrix Ionization Principle

### 2.1 Fundamentals of MALDI-MS

#### 2.1.1 Composition of MALDI Mass Spectrometer

MS involves converting a sample into moving gaseous ions, separating various charged ions according to their specific *m*/*z*, and then allowing them to form distinct trajectories in a high-vacuum mass spectrometer under an applied electric or magnetic field. Finally, through data recording and conversion, a mass spectrum is generated.^80^ MALDI-MS is a soft ionization technique that directly ionizes molecules via laser energy. It is coupled with a mass analyzer (usually a TOF analyzer) to detect molecules capable of absorbing laser energy. In its simplest configuration, a MALDI-MS mass spectrometer consists of an ionization source, a mass analyzer, and a detector (Figure 3).^81^ The ionization source first transfers sample molecules into the gas phase as charged ions. These ions are then directed into the mass analyzer, where the ions are separated based on their *m*/*z* values. After separation, the ions reach the detector, where the signals are recorded and processed to generate the final mass spectrum. This sequential process of ionization, separation, and detection is the core mechanism of MALDI-MS, enabling precise determination of the mass and relative abundance of different molecules in a sample.

**Figure 3:**
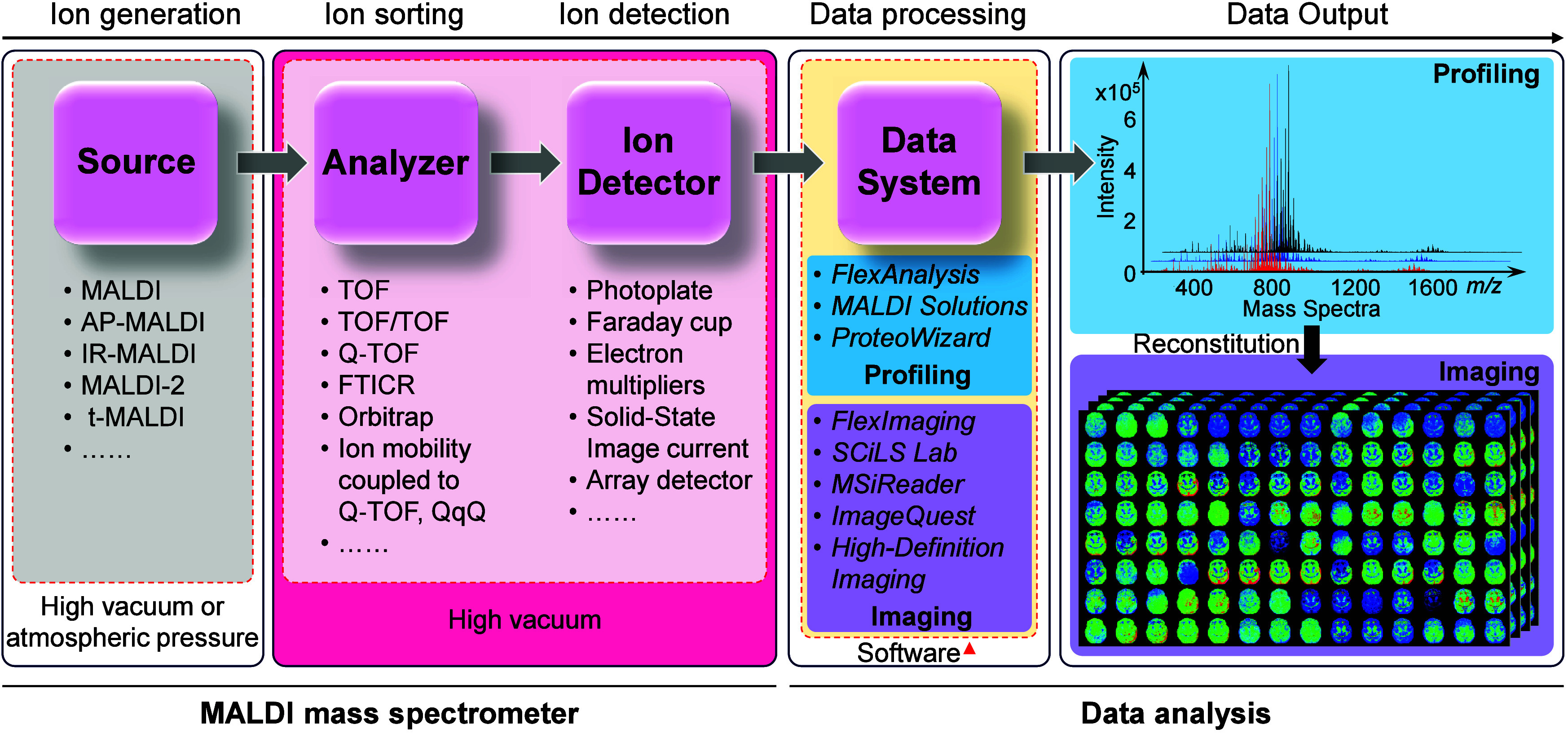
Components of the MALDI source mass spectrometer and data analysis. Software packages commonly employed for MS data processing from various companies are marked with a red triangle. *FlexAnalysis*, *FlexImaging*, and *SCiLS Lab* are products of Bruker Daltonics. *MALDI Solutions* is developed by Shimadzu. *ImageQuest* is provided by Thermo Fisher Scientific. *High-Definition Imaging* is a software offering from Waters. *ProteoWizard* and *MSiReader* are open-source software.

##### 2.1.1.1 Ion Source Laser

The earliest lasers used in MALDI-MS were the pulsed nitrogen (N_2_) laser with a wavelength of 337 nm and the Nd:YAG laser with a wavelength of 266 nm. With the discovery of new matrix compounds, the Nd:YAG laser with a wavelength of 355 nm has been more widely used.^82^ Beyond these UV fixed-wavelength sources, several additional laser sources have been introduced, spanning from the infrared (IR) to UV range, with pulse widths from nanoseconds to femtoseconds. Examples include high-repetition-rate lasers based on neodymium-ion-doped solid-state gain media such as Nd:YVO_4_ (neodymium yttrium vanadate) laser,^83^ Nd:YLF (yttrium lithium fluoride) laser,^84^ excimer lasers,^85^ wavelength-tunable lasers,^86^ and Er:YAG laser.^87^ Among these, UV lasers are particularly advantageous for MALDI-MS because they are strongly absorbed by matrices, efficiently driving the desorption and subsequent ionization of the analytes. Their laser beam diameter typically ranges from 150 μm to 5 μm.^88^ The quality of a mass spectrum generally improves as the excitation wavelength approaches the maximum absorption of the matrix, reaching optimal conditions. For MALDI-MSI, the spatial resolution of MALDI imaging is primarily determined by the diameter of the laser beam and laser step size. Notably, the size of the matrix droplets can affect the displacement of the analytes on the tissue surface, which further impacts resolution. While traditional MALDI-MS uses UV lasers to generate ions, IR-MALDI-MS has also proven capable of generating intact ions of large nucleic acids.^89^ Although IR-MALDI-MS is less frequently used than UV-MALDI-MS and lacks inexpensive laser sources, it possesses certain advantages that make it attractive for specific applications.^90^ Over the past two decades, the growing demand for MALDI imaging has spurred the development of high-repetition-rate solid-state lasers. Solid-state Nd:YAG lasers can operate at the high repetition rates necessary for MALDI-MSI, leading to a shift from low-repetition-rate nitrogen lasers to 355 nm high-repetition-rate Nd:YAG lasers, which are currently the most commonly used lasers in MALDI-MS.^82^ Nevertheless, lasers of different types and configurations continue to be applied in MALDI-MS, and optimal selection depends on two critical factors, the matrix absorption coefficient and the quality of mass spectrum.

##### 2.1.1.2 Ion Source Types of MALDI-MS

MALDI-MS is among the most widely applied MS techniques. Based on the angle (α) of laser irradiation with respect to the sample surface normal and the pressure in the ion source, MALDI-MS systems may be classified into three major categories: reflective MALDI, transmission MALDI, and scanning microprobe MALDI (SMALDI) (Figure 4).^38^ In classical reflective MALDI, the laser beam irradiates the matrix-covered sample surface at an angle between 0° and 90° relative to the analyzer inlet, ejecting ions without perforating the sample.^28^ In transmission MALDI, the laser beam irradiates the sample at 180° relative to the analyzer axis; thus, the laser beam must penetrate the sample, and ions are ejected in the direction of laser propagation. This transmission-geometry configuration has been successfully applied in high-lateral-resolution lipid/metabolite MALDI-MSI, achieving pixel resolutions as low as 5 μm.^28^ SMALDI was developed and first described by Spengler et al. in 2002 and employs perpendicular irradiation of the sample surface.^91^ Since traditional MALDI requires vacuum conditions, it is limited in analyzing volatile molecules and employing matrices that are unstable under vacuum conditions. To overcome this, atmospheric pressure MALDI (AP-MALDI) was proposed by Laiko et al. in 2000.^92^ Unlike high-vacuum MALDI, AP-MALDI operates at atmospheric pressure conditions, which is highly beneficial for liquid matrices and matrices incompatible with vacuum.^93^ An AP-SMALLDI-MS has been reported for MSI of small molecules,^94–96^ while an AP-MALDI ion source has also been developed for MSI,^97^ achieving a lateral resolution of 1.4 μm.^98^ Recently, plasma-ionization-enhanced ion sources and plasma-online-positioning ion sources such as AP transmission-mode MALDI-MSI (AP-TM-MALDI-MSI) have been successfully established, enabling the acquisition of high-quality spectral and imaging data.^99,100^ As mentioned above, MALDI-MSI can simultaneously record the lateral distribution of numerous biomolecules in tissue sections. However, its sensitivity is limited by the restricted degree of ionization limiting ionization efficiency. To enhance sensitivity, Soltwisch et al. introduced MALDI-2, a post-ionization laser strategy, to initiate a secondary MALDI-like ionization process in the gas phase.^101^ By shaping the laser beam and installing a focusing lens inside the MALDI ion source, the effective diameter of the main beam can reach approximately 5 μm. This significantly increases ion yields up to two orders of magnitude for lipids, fat-soluble vitamins, and carbohydrates enabling low-micrometer scale imaging of animal and plant tissues with a laser spot as narrow as 5 μm.^101,102^ Niehaus et al. modified the MALDI-2 ion source by integrating a customized transmission-mode unit into the dual-path backend and further developed a laser-induced post-ionization transmission-mode MALDI ion source, named t-MALDI-2, achieving high pixel sizes as small as 600 nm in brain tissue.^103^ Recently, a novel low-cost post-ionization scheme based on single-photon-induced chemical ionization with a pulsed RF-Kr lamp has been introduced. This scheme can generate a large number of lipid signals from mammalian tissue sections in MALDI-MSI, with an increase in ion yield by three orders of magnitude.^104^

**Figure 4:**
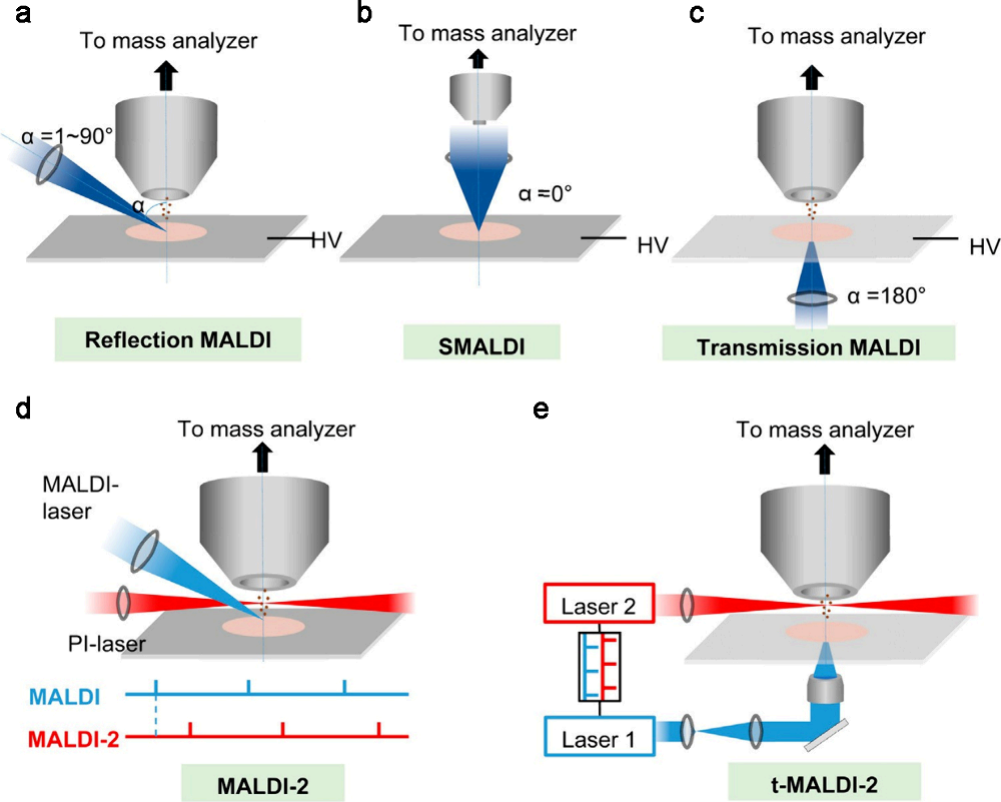
Principles of major ionization methods for MALDI sources. (a–c) Schematic of MALDI at several different laser angles (α). HV, high voltage. (d) Schematic drawing of the modified MALDI ion source. Primary MALDI and post ionization laser beams for more complete ionization and shielding aperture for increasing the cooling gas pressure in the region of ion generation. The lower panel illustrates the laser pulse triggering sequence. (e) Schematics of t-MALDI-2-MS. An actively Q-switched Nd: YLF laser (laser 1, λ = 349 nm, M2–1.05) is focused onto a matrix-coated sample via a UV-transmitting ×50 objective in back-side illumination geometry. The Nd:YAG laser (laser 2 wavelength 2266 nm) intersects the extended analyte matrix plume at a certain distance and a certain delay between the two laser pulses. Adapted and reproduced with permission from ref 28. Copyright 2022 The Authors under the terms of the Creative Commons CC BY license.

##### 2.1.1.3 Mass Analyzer

The commonly-used mass analyzers in MALDI-MS include quadrupole (Q), ion trap (IT), TOF, Q-TOF, Orbitrap, Fourier transform ion cyclotron resonance (FT-ICR), and trapped ion mobility spectrometry (TIMS) (Figure 5).^105–108^ Among them, quadrupole analyzers offer fast-scanning speeds and accurate quantification, being well-suited for rapid quantitative analysis. However, they have relatively low resolution, and MS/MS analysis requires tandem configuration.^109^ The IT offers high sensitivity, which is ideal for qualitative analysis and identification of unknown substances. Nevertheless, quantification is easily hindered by the space-charge effect.^110,111^ MALDI-TOF­(/TOF) remains the most widely used mass spectrometer, owing to its high MS data acquisition speed (with a laser frequency of up to 10 kHz) and wide mass detection range (from 0 to 100 kDa and even above).^28,50^ Q-TOF MS systems perform well in obtaining MS/MS fragment ions but are sensitive to parameter settings, which can affect performance in both the LMW and high-molecular-weight (HMW) ranges.^112^ Orbitrap and FT-ICR have remarkable advantages in acquiring ultra-high-resolution data, which is beneficial for deciphering complex MS signals. Moreover, they exhibit high sensitivity for small molecule analysis. Specifically, FT-ICR MS can provide high mass-resolving power (*R* > 1,000,000 at *m*/*z* 200), thus resolving isotopic fine structures.^28^ Recently, the introduction of ParaCell has further enhanced the FT-ICR mass resolution to over 20,000,000.^113^ ParaCell achieves a harmonized electric field in the ICR cell, overcoming the limitations of traditional ICR traps and providing full-range ion stability. As a result, FT-ICR resolution is orders of magnitude higher than other mass analyzers, allowing precise isotopic structure determination and the analysis of highly complex mixtures.^114^ MALDI Orbitrap FTMS also delivers high mass resolution (*R* > 140,000).^28^ However, both Orbitrap and FT-ICR are limited by high operating costs and long analysis times in the ultra-high-resolution mode. Conversely, some MALDI-Q-TOF instruments with intermediate resolving power have been combined with TIMS, adding an extra separation dimension and showing strong potential for separating isomeric molecules.^115^

**Figure 5:**
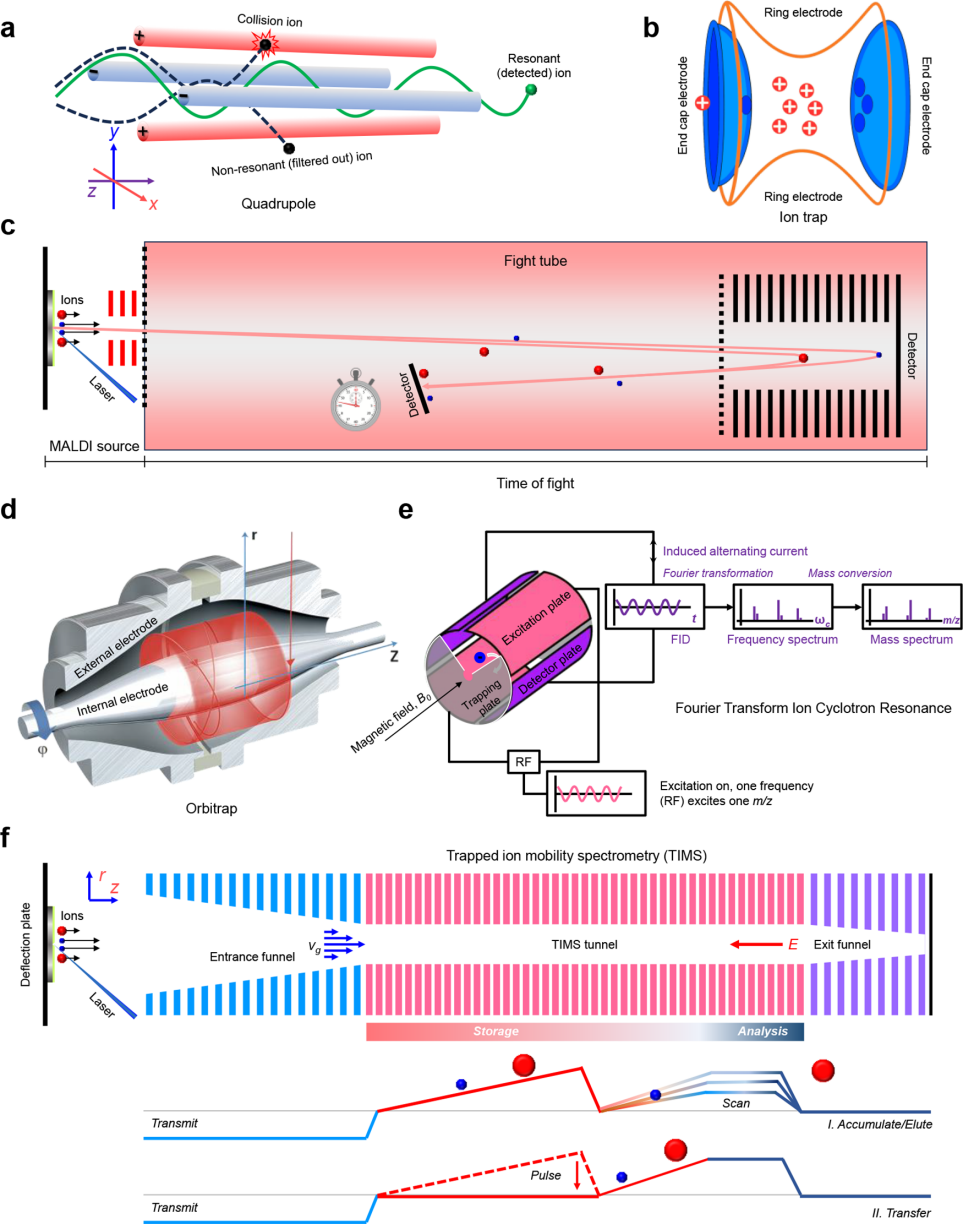
Mass analyzers commonly-used in MALDI source mass spectrometer. (a) Quadrupole (Q). (b) Ion trap (IT). (c) Time of flight (TOF). (d) Orbitrap. Adapted and reproduced with permission from ref 107. Copyright 2011 ASBMB. (e) Fourier transform ion cyclotron resonance (FT-ICR). Adapted and reproduced with permission from ref 108. Copyright 2024 The authors. Licensee MDPI, Basel, Switzerland. (f) trapped ion mobility spectrometry (TIMS).

#### 2.1.2 General Process of MALDI-MS

The fundamental workflow of MALDI-MS broadly comprises two key stages: first, the formation of matrix/analyte co-crystalline spots by either mixing matrix solutions with analyte solutions or spraying matrix solutions onto tissue sections, with the notable exception that inorganic matrices typically do not require such co-crystallization with analytes. Subsequently, when the sample surface is irradiated by a laser, the target molecules are ionized, and the resulting ions are propelled by an electric field into the mass analyzer of the mass spectrometer for detection. The raw mass spectral profiling data collected by the detector are then processed using specialized software or imaging tools to reconstruct the spatial distribution of analytes across the sample surface, ultimately presented in a visualized format.^88^ In essence, the basic workflow of MALDI-MS analysis encompasses sample preparation, matrix application, data acquisition, and analysis (Figure 6).

**Figure 6:**
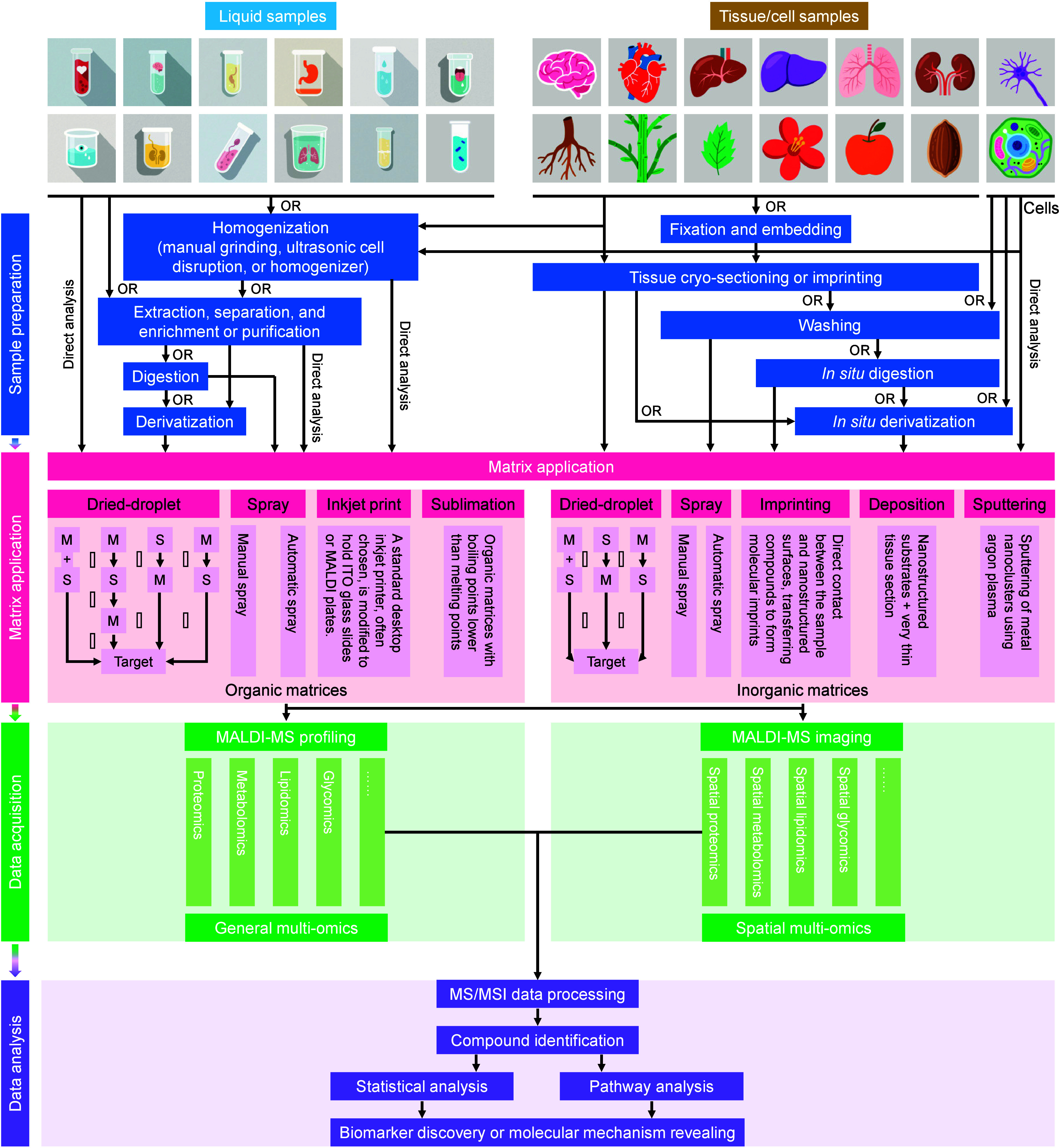
The basic workflow of MALDI-MS analysis. The procedure comprises four main stages: sample preparation (for liquid, tissue, or cell samples), matrix application (via methods such as dried-droplet, spraying, inkjet printing, sublimation, imprinting, deposition, and sputtering), data acquisition, and analysis. Raw spectral data are processed for molecular profiling or spatial imaging, enabling identification, statistical analysis, and pathway investigation of molecules of interest. Labels denote: M, matrix; S, sample; numerals (1, 2, 3) indicate dried-droplet sequence on the target plate.

##### 2.1.2.1 Sample Handling or Tissue Treatment

In MALDI-MS analysis, high-quality sample preparation is a pivotal step for acquiring reliable and reproducible mass spectral data, making the optimization of sample preparation protocols particularly critical. Depending on the nature of the samples, MALDI-MS analysis can be performed in three primary modes: direct sample analysis, *in situ* tissue detection, and tissue imaging. Each mode requires specific sample preparation workflows, as outline below.

###### 2.1.2.1.1 Sample Preparation for Direct Analysis by MALDI-MS

Direct MALDI-MS analysis is primarily applied to liquid samples, tissue or cell extracts, and microbial samples. In clinical settings, biological fluids are frequently analyzed, with plasma and/or serum being the most common specimens, containing a rich array of analytes.^116^ To date, many other biological fluids, such as cerebrospinal fluid,^117^ lymphatic fluid,^118^ gastric juice,^119^ sweat,^120^ saliva,^121^ tears,^122^ urine,^123^ semen,^124^ bronchoalveolar lavage fluid,^125^ and synovial fluid,^126^ have also been investigated by MALDI-MS. Additionally, in environmental assessments, water samples are routinely analyzed to detect contaminants. These liquid samples can be analyzed directly (*i.e.*, without pretreatment) by MALDI-MS for faster turnaround or indirectly via pretreatment steps designed to concentrate analytes and reduce interference from biological matrices. Pretreatment strategies depend on sample characteristics, target analytes, and matrix type. For low abundance compounds, *e.g.*, neuropeptides,^127^ phosphopeptides,^128^ hormones,^129^ and metabolites,^34^ extraction and enrichment are often necessary. Tissue or cell homogenization is another critical step affecting detection sensitivity typically involving manual grinding, sonication, or cell disruption devices, performed in solvents or buffers that dissolve target compounds while minimizing degradation. In clinical microbiology, MALDI-MS sample preparation methods include transferring intact bacterial colonies onto MALDI plates (with or without formic acid solution) and protein extraction.^130,131^ Direct colony processing is the simplest, fastest, and most cost-effective method, where colonies are “picked” from culture plates and deposited onto MALDI target plates. Adding formic acid solution to the MALDI plate can improve mass spectrum quality, particularly for organisms like yeast. Protein extraction, while effective, is less practical for clinical microbiology applications compared to direct colony testing and is generally reserved for hazardous or hard-to-lyse organisms.^131^

###### 2.1.2.1.2 Sample Preparation for MALDI-MS *In Situ* Detection and/or Imaging

High-quality tissue sections are essential for MALDI-MS *in situ* detection and/or imaging. Various solid biological samples, such as plant tissues, animal tissues, and clinical specimens are widely analyzed using MALDI-MS.^31,45,132^ Optimizing sample preparation is critical for accurate detection of endogenous compounds in microdomains and reliable spatial mapping. In general, preparation of solid samples (*e.g.*, biopsies and organs) involves four main steps: sample fixation/embedding, tissue sectioning, washing (optional), and *in situ* tissue derivatization (optional).

Fresh or frozen, chemically unmodified tissues are preferred for MALDI-MS.^133^ Rapid freezing is commonly used to prepare fresh-frozen tissues, employing dry ice powder, liquid nitrogen, or liquid nitrogen-cooled isopentane. However, optimal freezing methods depend on sample type, with priorities including preserving tissue morphology and minimizing analyte degradation.^112^ Tissues can be preserved in embedding agents prior to freezing, where they are immersed in polymers such as optimal cutting temperature (OCT) compound or Neg50.^134^ For example, OCT maintains the integrity and shape of fragile tissues (*e.g.*, breast or cardiovascular tissues) during freezing and sectioning, enabling sectioning at very thin thicknesses (<5 μm),^135^ critical for histochemistry requiring high-quality staining. However, these embedding medium polymers are readily ionized in MS, with OCT often inducing significant ion suppression in high-mass regions and interfering with the detection of small molecules, such as lipids and metabolites.^136,137^ To overcome these issues, alternative embedding materials compatible with MALDI-MS have been developed, such as carboxymethyl cellulose (CMC)^138,139^ and gelatin.^140^ Strohalm et al. introduced poly­(*N*-[2-hydroxypropyl]­methacrylamide) as a novel embedding medium that reduces mass interference and ion suppression,^141^ while Yang et al. demonstrated that polyacrylamide gel (PAAG) enables one-step embedding without heating, preserves section morphology, and enhances metabolite ion signals.^142^

Fresh tissues can also be chemically fixed to prevent degradation and preserve cellular/organelle structure. Formalin-fixed and paraffin-embedded (FFPE) tissues are widely used in clinical and basic medicine.^143^ Formalin crosslinks tissue components, particularly proteins, nucleic acids, and lipids, via methylene bridges, immobilizing soluble proteins to insoluble structural proteins and forming a stable tissue network.^144^ Paraffin embedding enables long-term storage at room temperature (years to decades).^145^ However, formalin-induced protein crosslinking reduces sensitivity in MALDI-MS analysis of FFPE tissues, and molecular diffusion during fixation complicates metabolite analysis.^139^ To address these issues, various FFPE processing protocols have been developed, including tissue proteolytic digestion for protein studies,^146^ and methods for analyzing/imaging *N*-glycans^147^ and metabolites.^148^ Despite chemical denaturation and partial loss of components (*e.g.*, during pre-dehydration), retained analytes in FFPE tissues still provide valuable insights into spatial distribution and underlying pathological mechanisms.

Embedded tissues are typically sectioned using a cryostat or microtome. Care is required to avoid tissue folding, cracking, or displacement, which can hinder subsequent MS analysis. Section thickness (3–20 μm) significantly influences spectral quality.^134^ In typical MALDI-TOF-MS analysis, sections >20 μm may exhibit charge accumulation due to low conductivity, causing charge effects.^149^ For MALDI-MS, sections must be mounted on conductive platforms to facilitate electric field-driven ion extraction, with commonly used conductive platforms including indium tin oxide (ITO)-coated glass slides, stainless steel targets, or nanostructured inorganic substrates. For ITO-coated slides, adhesive films can replace thaw-mounting, particularly for challenging samples difficult to section (*e.g.*, rice grains, bones, or whole-body animals).^150^

Tissue washing is sometimes necessary in MALDI-MS workflows to eliminate interference or enhance sensitivity for specific analytes. Organic solvents such as methanol (MeOH), ethanol (EtOH), acetonitrile (ACN), acetone, hexane, chloroform (CHCl_3_), 2-propanol (iPrOH), acetic acid (AcOH), xylene, toluene, and *tert*-butyl methyl ether can remove small molecules, thereby improving *in situ* protein detection and imaging by reducing ion suppression from salts, lipids, and metabolites.^151–153^ For most small molecules (*e.g.*, lipids), MALDI-MS detection and imaging do not require washing prior to matrix deposition. However, washing may be used to deplete high-abundance species, improving ionization and detection of low-abundance analytes.^154^ Overall, the washing protocols should be optimized for target molecules. Several solvent-based strategies have been reported.^152,155–157^

In MALDI-MS, chemical derivatization reagents react with specific functional groups of analytes on tissue surfaces, altering their molecular structure, polarity, and other physicochemical properties to enable efficient *in situ* analysis of hard-to-ionize analytes.^158^ Ideal derivatization reagents should: (i) react selectively and stably with analytes in complex matrices, (ii) react rapidly under mild conditions without byproducts, (iii) not interfere with detection or be easily removed, and (iiii) be readily accesible.^159^ Common derivatization approaches include esterification, acylation, substitution, addition, and oxidation.^160^ Derivatization serves multiple roles, such as modifying analyte properties to enhance separation/enrichment,^161^ reducing matrix interference to improve detection accuracy and sensitivity,^162^ boosting *in situ* ionization efficiency of hard-to-ionize molecules for enhanced MSI performance,^163^ *etc.*. Derivatization has been applied to catecholamines,^164^ sulfur-containing metabolites and proteins,^165^ NTs with primary/secondary amines or phenolic hydroxyls,^166,167^ cholesterol,^168^ and corticosteroids^169^ to improve ionization and sensitivity. In lipidomics, derivatization has been applied to identify double-bond isomerization in glycolipids and phospholipids (PLs).^170^ To date, many reagents/methods, such as benzaldehyde,^171^ benzophenone,^172^ and ozone-induced dissociation,^173^ have been developed and successfully used to distinguish the position of C=C bonds in lipid isomers. In short, the choice of derivatizing agent depends on analyte characteristics and scientific goals, with comprehensive MALDI-MS derivatization strategies available elsewhere.^163^

##### 2.1.2.2 Matrix Application

Matrix selection is critical for obtaining high-quality data from biological samples and for accurate compound imaging on tissue sections in MALDI-MS experiments. Biomolecules co-crystallize with the matrix, which absorbs laser energy and protects biomolecules from destructive ionization.^133^ For tissue imaging, matrix performance impacts four key aspects of MALDI-MSI: efficient analyte extraction (poor extraction reduces sensitivity), small crystal size (critical for high spatial resolution), high purity (minimizes background ion interference), and uniform crystal layer formation (ensures reproducibility and enables for quantitative analysis).^112^ Thus, choosing appropriate matrices and deposition strategies is essential for the acquisition of high quality MALDI-MS/MSI data.

###### 2.1.2.2.1 Matrix Selection

The choice of MALDI matrix (organic or inorganic) depends on the mass range and chemical properties of analytes. Suitable matrices should be stable, facilitate analyte ionization, and avoid generating interfering ions in the mass range of interest.^174^ Traditional organic matrices are SOMs such as SA, 2,5-dihydroxybenzoic acid (DHB), and α-cyano-4-hydroxycinnamic acid (CHCA), selected empirically for proven performance. To meet diverse analytical needs, numerous novel SOM matrices with distinct ion modes (positive, negative, or dual-polarity) have been rigorously screened, evaluated, and validated for MALDI-MS. Designed and synthesized matrices have also addressed limitations of traditional SOMs,^62,175,176^ offering enhanced ionization efficiency, reduced background interference (via higher molecular weight), lower volatility for small molecules, and improved structural analysis capabilities. Inorganic matrices, including carbon-based, silicon-based, metal-based, organic framework nanocomposites, and quantum dots, provide versatile options for bioanalysis, particularly for small biomolecules.^77,177^ Given the vast and expanding range of available matrices, detailed information is provided in section 3, Matrices for MALDI-MS, and section 4, MALDI Matrices for Multi-Omics Analysis.

###### 2.1.2.2.2 Matrix Deposition Methods

The choice of matrix deposition technique critically influences MALDI-MS data quality and MALDI-MSI reproducibility.^139^ The physicochemical properties of matrix compounds affect coating quality, as different compounds crystallize at varying rates even with identical deposition parameters, resulting in distinct crystal morphologies. Matrices must be applied in a standardized manner to ensure thorough analyte extraction while minimizing delocalization. Common approaches include manual and automated techniques for organic matrices, including dried-droplet, spraying, inkjet printing, and sublimation (Figure 7).^174,178^ Inorganic matrices are deposited as uniform coatings via spraying or sputtering, or as solid nanostructured substrates loaded with samples via imprinting or deposition.^179^ Each technique has unique advantages and limitations in terms of operation, cost, signal intensity, reproducibility, and spatial resolution.

**Figure 7:**
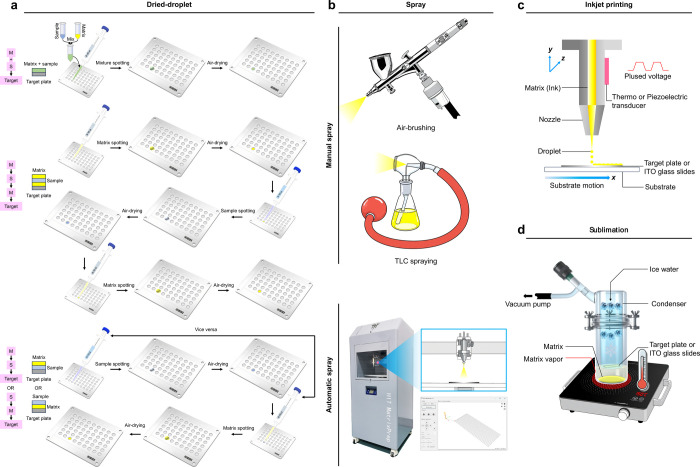
Matrix deposition methods used for organic matrices in MALDI-MS analysis, including (a) dried-droplet, (b) spraying, (c) inkjet printing, and (d) sublimation.

Organic matrices are typically deposited as solutions in water/organic solvent mixtures. In the dried-droplet method, matrix and sample are either premixed or layered them sequentially, followed by co-crystallization during solvent evaporation.^178^ However, this method often forms ring-shaped crystals and exhibits “hotspots” or “sweet spots” with significantly higher signal intensity than surrounding areas. This method is mainly suitable for liquid samples, whereas tissue samples generally require matrix spraying. Matrix spraying methods include manual spraying, automated spraying, and sublimation (Figure 7). Manual spraying employs TLC sprayers or airbrushes.^180^ Due to the simple operation, this method was once widely used but required constant room temperature, low humidity, and significant skill. Automated sprayers, such as the ImagePrep (Bruker Daltonics, Bremen, Germany),^181^ SunCollect (SunChrom, Friedrichsdorf, Germany),^182^ TM Sprayer (HTX Technologies, Carrboro, NC, USA),^183^ and HIT MatrixPrep (HIT Co., Ltd., Beijing, China),^45,184,185^ offer high efficiency, automation, and convenience, reducing operation time and improving deposition uniformity compared to manual methods, thereby enhancing detection efficiency. However, automated sprayers are costly, prone to nozzle clogging, require parameter optimization (*e.g.*, flow rate, spray height, matrix concentration),^50^ and can still cause matrix aggregation. Variants such as applying an electric field during spraying have been also explored.^132,186–188^ Inkjet printing deposits high-density microdroplet arrays on surfaces, creating matrix spots with diameters exceeding 200 μm and accumulating tens of picoliters (∼100 pL) of liquid at each coordinate.^134^ A standard desktop inkjet printer, often chosen, is modified to hold lTO glass slides or MALDl plates, such as CHIP-1000 (Shimadzu Co., Kyoto, Japan)^189^ and Portrait 630 spotter (Labcyte Inc., California, USA).^190^ Despite having lower spatial resolution compared to matrix spray methods, inkjet printing offer significant advantages, including a markedly improved signal-to-noise (S/N) ratio, a greater number of detected peaks, as well as excellent spatial and temporal reproducibility. Sublimation is a solvent-free alternative involving matrix vaporization under reduced pressure and elevated temperature,^191^ producing highly uniform coatings with small co-crystals.^192^ By avoiding solvents, it minimizes analyte dissolution and migration, enabling more accurate spatial mapping.^193^ Limitations include potential loss of non-co-crystallizing analytes, high matrix consumption, and sensitivity to time and temperature.

Compared to organic matrices, inorganic matrix-based MALDI-MS sample preparation is simpler because co-crystallization with analytes is unnecessary.^65^ Inorganic matrices provide higher reproducibility, spatial fidelity, and resolution, exhibiting greater potential for accurate quantitative analysis and imaging.^194,195^ Five primary methods are used for inorganic matrices: dried-droplet, spraying, imprinting, deposition, and sputtering. The dried-droplet method for inorganic matrices resembles that of organic matrices, wherein the inorganic matrix is either pre-mixed with the sample or sequentially layered during spot deposition. Spraying deposits a uniform nanolayer onto samples, either manually or automatically,^179^ though solvents may dissolve small molecules,^196^ and nanoparticle aggregation complicates coating uniformity;^197^ solvent evaporation is often accelerated to form continuous films. Imprinting involves direct contact between the sample and nanostructured surfaces, transferring compounds to form molecular imprints.^198^ However, it may be unsuitable for low-abundance metabolites due to low transfer efficiency^199^ and limited spatial resolution.^200^ Deposition methods immobilize samples on nanostructured substrates during analysis,^179^ requiring very thin sections (often embedded in protective materials); thicker sections reduce ionization efficiency and conductivity.^179^ Sputtering deposits thin, uniform layers of pure metal nanoclusters using argon plasma, avoiding colloid aggregation and enabling conductivity for nonconductive samples,^201^ However, it requires specialized equipment and skills for precise thickness control. Additionally, nanoparticle implantation offers high reproducibility and resolution^202^ but requires dedicated instrumentation. Ultimately, each deposition method involves trade-offs, and protocols must be tailored to analyte type and experimental goals. Comprehensive reviews of matrix application methods are also available in literature.^50,179,203^

##### 2.1.2.3 Data Acquisition and Analysis

###### 2.1.2.3.1 Data Acquisition

To minimize unnecessary degradation of biomolecules, matrix-mixed/coated samples should be analyzed by MS as promptly as possible. For reproducibility with in a single MALDI-MS experiment, instrumental parameters, including voltage settings and laser power, must remain constant. This ensures high reproducibility and accuracy, which are critical for comparing multiple detected spots, specimens, or representative regions within a single tissue section. In profiling mode, statistical robustness requires sufficient sampling of discrete regions (typically 5–20 spots, each 0.2–1 mm in diameter).^204^ For MALDI-MSI, spatial resolution is predefined by the region of interest (ROI) and scan spacing prior to measurement. The total acquisition time depends on the number of data pixels, laser frequency, number of laser shots per pixel, and the time required for stage movement. For example, analysis of a 1 × 1 mm^2^ ROI with 10 μm scan spacing (10,000 data points) using an MS microscope equipped with a 1,000 Hz laser (100 shots per data point) requires approximately 1 h.^49^ Importantly, MALDI-MSI datasets integrate both mass spectral and spatial information, necessitating high-quality data collection across both dimensions. Acquisition procedures for high-quality MALDI-MSI spectra are generally aligned with those of conventional MALDI-MS.

###### 2.1.2.3.2 Data Analysis

Data analysis involves several critical steps to ensure accurate analysis and interpretation of MALDI-MS and MALDI-MSI results. Initially, several preprocessing steps are employed to reduce experimental variance, including normalization, baseline correction, spectral alignment, and smoothing.^205^ Subsequently, compound identification is performed via high-accuracy mass matching against reference databases. This is often followed by tandem MS (MS/MS) analysis to acquire fragment ion spectra, enabling differentiation of isomeric species and validation of structural details. Finally, statistical and pathway analyses are conducted to discern significant abundance changes across samples and to elucidate the biological context of identified molecules. Specialized software facilitates these processes (Figure 3): examples include Bruker’s *FlexAnalysis*, Shimadzu’s *MALDI Solutions*, and the open-source platform *ProteoWizard*, which offer spectral processing, peak matching, data visualization, and compound identification. MALDI-MSI generates particularly large datasets (several gigabytes), driving the development of software packages optimized for efficient spectral analysis and visualization (Figure 3). Commercial and open-source tools for advanced data processing and statistical analysis include *MSiReader* (a free, open-source MSI processing and visualization tool), Bruker’s *FlexImaging*, *SCiLS*, Thermo Fisher Scientific’s *ImageQuest*, and Waters’ *High-Definition Imaging* (HDI). To address challenges in standardization and reproducibility of MSI data, researchers have integrated 18 distinct MSI analysis tools within the open-source Galaxy computational platform, enabling compatibility across key MSI workflows, including visualization, image co-registration, statistical analysis, and data mining.^206^ For more detailed discussions of data processing, visualization, and statistical analysis in MALDI-MS and MALDI-MSI, refer to specialized reviews.^207^

### 2.2 Ionization Mechanism of MALDI-MS

Theories and models of ionization mechanisms of MALDI-MS are instrumental in gaining a better understanding of the factors influencing analyte ionization efficiency. Insight into the ion-formation process can guide rational matrix selection and inspire the design of novel matrices. However, MALDI-MS ionization is a highly complex process that defies simple description. Numerous reviews have delved into the ionization mechanisms of MALDI,^65,208,209^ proposing a multitude of potential mechanisms, which are briefly summarized below.

#### 2.2.1 Organic Matrix Based Ionization Mechanisms

In MALDI-MS using organic matrices, analytes co-crystallize with an excess of energy-absorbing matrix compounds. Upon laser irradiation, the matrix absorbs photon energy, initiating desorption of analytes. During this process, the crystalline sample transitions from the solid to gas phase. Desorption efficiency depends on analyte properties (*e.g.*, MW, polarity, hydrophilicity, and hydrophobicity) as well as excitation conditions (*e.g.*, laser parameters and analyte-matrix co-crystal morphology).^210^ Typically, smaller crystal sizes and elevated system temperatures improve efficiency.^211^ It is essential to recognize that there is no universal mechanism for ion formation that applies across all scenarios. In MALDI-MS utilizing organic matrices, ion formation is often described as a two-step process: primary ionization, followed by secondary reactive ionization in the dense desorption plume.^208^ This initial step occurs when the matrix absorbs the energy from the laser pulse, resulting in the formation of matrix ions. The absorbed energy causes the solid matrix to vaporize, creating a plume of particles, including both neutral matrix molecules and ions. Following primary ionization, a dense plume of desorbed material is produced. In this cloud, secondary ionization can occur through matrix–analyte or analyte–analyte interactions. In this phase, the ionic species formed during primary ionization can interact with the analyte molecules, resulting in the generation of analyte ions, such as protonated species, which are then available for detection by the mass spectrometer. The mechanisms governing primary ionization remain a topic of ongoing investigation, and various models have been proposed to elucidate this phenomenon, such as the photochemical ionization model (photoexcitation/pooling model),^212^ “lucky survivor” model (cluster model),^44^ and coupled photophysical and chemical dynamics (CPCD) model.^213,214^ In contrast, the secondary ionization processes occurring in the MALDI plume are better established and widely accepted,^215^ involving key reactions such as protonation, electron emission, and cation transfer.

##### 2.2.1.1 Primary Ionization

###### 2.2.1.1.1 Photoexcitation/Pooling Model

In 1992, Ehring et al. proposed the photochemical ionization model for the MALDI process.^212^ This model describes a two-step ionization process, which involves the photoionization of matrix molecules and the subsequent gas-phase ionization of analytes through reactions with ionized matrix molecules. The primary mechanism is considered to be matrix photoionization, triggered by a pulsed laser beam, producing matrix ions followed by rapid desorption.^216^ In the photoexcitation/pooling model, photoionization occurs through electron pooling in photo-excited matrix molecules. When matrix molecules absorb photons from the laser beam, electrons are promoted to the first excited state (S_1_) or higher excited states (S_*n*_). The interaction between two excited states (due to wave-function overlap) enables pooling, leading to matrix-derived primary ions generated by the energy pooling/annihilation process.^217^ The proton disproportionation hypothesis further suggests that the absorbed photon energy is converted into thermal energy through ultrafast nonradiative relaxation, thus generating ionized matrix substances.^218^ Energy pooling and multiphoton absorption are therefore the main processes leading to the photoionization of matrix molecules. Analyte ions are subsequently formed through protonation or deprotonation via collisions with matrix ions, generating positive or negative analyte ions.^219^ However, the sum of the energies of two nitrogen laser photons is 7.36 eV, which is lower than the ionization potential of most matrix molecules; therefore, it is considered unlikely for a single matrix molecule to undergo two-photon ionization.^213^ Similarly, while MALDI laser irradiance ranges from 10^6^ to 10^7^ W cm^–2^, the efficiency of three-photon ionization should be very low. Due to the limited number of excited matrix molecules produced, the energy-pooling process is unlikely to generate abundant matrix ions. Moreover, this model fails to explain several characteristic MALDI phenomena, such as the matrix suppression effect,^220^ “sweet spots”,^221^ and the high ratio of polymer ions to monomer ions.^209^

###### 2.2.1.1.2 “Lucky Survivor” or Cluster Model

The “lucky survivor” model or cluster model was developed by Karas et al.^44^ This model postulates that analytes are incorporated into matrix crystals while retaining their charge states in solution, either single- or multicharged. After laser irradiation, analytes exist as preformed ions in the matrix crystals and are released in the form of clusters consisting of analyte ions, matrix molecules, and a corresponding number of counter-ions. In this model, positively charged clusters (analyte ions, matrix molecules, and counterions) undergo dissociation by losing neutral matrix molecules, releasing singly charged analyte ions, which are termed “lucky survivors”.^222^ To confirm the presence of analytes in their charged states in solution, pH indicator probes^223^ and pre-charged analyte adducts of highly acidic ions from the solution can be used for detection.^224^ However, the original “lucky survivor” model^44^ has certain limitations in describing the generation of analyte anions. In negatively charged clusters, excess negative charge can neutralize positively precharged analytes. Nevertheless, subsequent deprotonation of the analyte by counterion neutralization is thermodynamically unfavorable because the acidity of the carboxylic acid groups of peptides and proteins is lower than that of common counterions (such as trifluoroacetic acid (TFA) or chloride). To explain this phenomenon, an improved “lucky survivor” model was proposed.^225^ This model assumes that the excess charge of the matrix [matrix+H]^+^ or [matrix–H]^−^ within the cluster generates protonated analytes or deprotonated analytes through counter-ion neutralization. Matrix anions typically consist of carboxylates or delocalized phenolates, whose acidity is similar to that of the analyte carboxylic acid groups. Therefore, the efficiency of analyte deprotonation is often low, consistent with the typically low-intensity anion analyte signals obtained using acidic matrices.^226^

###### 2.2.1.1.3 Coupled Physical and Chemical Dynamics Model

The CPCD model, also known as the two-step model or two-step energy pooling model, was initially developed solely for the DHB matrix under UV laser irradiation, but was later extended to other analytes and ion polarities. CPCD has been proven to be a quantitative model that is highly compatible with various empirical MALDI rules.^214,227^ Unlike the “lucky survivor” model, the CPCD model assumes that analytes are released in a neutral form, either due to the incorporation of uncharged substances into the matrix crystals or the quantitative charge recombination of pre-charged analytes with corresponding counter-ions. Proposed by Zenobi and Knochenmuss, the CPCD model treats analyte ionization as a secondary process,^228^ including laser excitation, ion formation, ion–molecule and ion–ion reactions, and coupled ablation. Primary ionization involves pooling of matrix electronic excitations, generating matrix ions. These ions undergo secondary charge-transfer reactions with neutral analyte molecules in the expanding ablation plume. Gas-phase collisions between neutral analytes and protonated [matrix+H]^+^ or deprotonated [matrix–H]^−^ ions lead to proton-transfer reactions and protonated or deprotonated analytes respectively, resulting in analyte ionization.^208,229^

###### 2.2.1.1.4 Excited State Proton Transfer Model

Excited state proton transfer (ESPT) is considered an attractive ionization pathway because it can occur in the first electronically excited state, making it a single-photon event under UV excitation. In this ionization mechanism, the primary species is the excited matrix molecule (M*). Proton transfer proceeds from M* to the analyte (A) and then back to the matrix molecule (M) generatinges [analyte+H]^+^ and [matrix+H]^+^ ions, respectively.^27,228^ However, in MALDI, ESPT is highly dependent on environmental conditions that can effectively stabilize charge separation.^208^

###### 2.2.1.1.5 Autoprotolysis, Polar Fluid Model, and Preformed Ion Emission Model

The simplest proposed mechanism for generating native ions is ground-state or thermal autoprotolysis: M + M → [M+H]^+^+[M–H]^−^. This method of simultaneously generating [M+H]^+^ and [M–H]^−^ through autoprotolysis is particularly appealing. However, it requires an exceptionally high enthalpy change, estimated to be approximately 5 eV, which may result in an exceedingly small equilibrium constant, thus hindering the feasibility of the reaction under standard conditions.^208^ The polar fluid model proposed by Beavis et al.^230^ and Chait et al.^231^ is regarded as an improved version of the autoprotolysis model. Here, cations and anions are assumed to be stabilized by the polar properties of the matrix. Due to the presence of certain matrix and analyte molecules as ionic species in solution, it is plausible that some may retain their ionic forms in solid samples prepared via solvent evaporation. Consequently, the preformed ion emission model assumes the emission of [matrix+H]^+^, [analyte+H]^+^, or both under laser irradiation^222^. Because [M+H]^+^ formation in solution may be accompanied by the generation of [M–H]^−^, this model can be considered a special case of autoprotolysis.

###### 2.2.1.1.6 Thermally Induced Proton Transfer Model

In the thermally induced proton transfer model, the total ion generation efficiency changes when the analyte is added to the matrix. This model suggests that analyte ions can be generated either as primary ions during the proton-transfer process between the matrix and the analyte, matrix + analyte → [matrix-H]^−^ + [analyte+H]^+^, or as secondary ions through subsequent ion-molecule reactions.^232^ When the analyte is added to the matrix, the thermally induced proton transfer model predicts that if the proton-transfer efficiency between the matrix and the analyte is greater than that between matrix molecules, the total number of ions can increase significantly.^232,233^

###### 2.2.1.1.7 Summary

Numerous models have been proposed to explain the MALDI primary ionization process. According to the electronic state of M involved in the formation of [M+H]^+^, the models can be classified into two categories: excited-state models (exciton pooling, CPCD, and ESPT models) and ground-state models (polar fluid, thermally induced proton transfer, and cluster models).^225^ However, the primary ionization mechanism in the MALDI process remains unresolved. The interplay of phase transitions, primary ionization, plume expansion, ion–molecule reactions, and thermodynamics is still under active investigation. While current understanding has advanced beyond empirical description and can aid practical applications, MALDI primary ionization is clearly a complex, multifactor-influenced process. Therefore, deeper mechanistic insights are crucial, for the rational design of new matrices to enhance ionization efficiency and sensitivity, ultimately facilitating more accurate analyte detection of various analytes and broadening the potential applications of MALDI across diverse scientific disciplines.

##### 2.2.1.2 Secondary Ionization

Secondary ionization in MALDI-MS plays a pivotal role in determining the final charge state and detectability of analyte ions. Following primary ionization, the dense ablation plume facilitates numerous bimolecular interactions between matrix-derived ions (or clusters) and analytes.^208^ These interactions or collisions lead to proton transfer, electron emission, cation exchange, and other charge-modifying reactions, ultimately shaping the mass spectral output. As the predominant collision partner, the matrix imposes characteristic trends on secondary ionization behavior across different analyte classes.^208^ Herein, we examine six key secondary ionization phenomena in MALDI: the matrix suppression effect (MSE), analyte suppression effect (ASE), proton transfer, cationization, electron transfer, and multicharge ionization, each of which exerting distinct effects on ion yield, charge-state distribution, and spectral interpretability.

###### 2.2.1.2.1 Matrix Suppression Effect

When the matrix-to-analyte concentration ratio is optimized, matrix ions can be almost completely suppressed, yielding mass spectra dominated by analyte ion signals.^234^ This concentration-dependent phenomenon is referred to as the MSE,^208^ being highly significant in MALDI-MS. For MSE to occur, analyte molecules must react with all matrix ions formed during primary ionization. Because both analyte and matrix ion yields depend on laser influence, MSE is also laser-dependent. Novel matrix-precoated MALDI target plates can suppress the matrix signal without diminishing analyte signal intensity, as the analyte is more uniformly dispersed on the precoated plates than in the standard droplet method.^235^ Precoated or non-metallic MALDI plates can influence the kinetic energy of photoelectrons (formed in the primary ionization step), thus enhancing both the MSE and analyte signals.^236^

###### 2.2.1.2.2 Analyte Suppression Effect

Apart from changing the matrix signal in the presence of one analyte, secondary reactions can also strongly affect the relative intensities of multiple analytes. For example, in a mixture of analytes A and B, if B reacts more effectively with the matrix, or if charge transfer occurs from A to B, the signal of analyte A will be weaker relative to its intensity in the absence of B. This phenomenon is called the ASE.^208^ Although ASE represents an extreme case in MALDI, it is a commonly observed phenomenon in MALDI-MS analysis.

###### 2.2.1.2.3 Proton Transfer

Proton transfer is one of the most important secondary reactions in the analysis of polypeptides and proteins. The gas-phase proton affinity of amino acids (AAs) ranges from 885 to 1,025 kJ/mol, and proton transfer from typical matrices is endothermic.^237^ This reflects the frequent observation that peptides containing more basic residues are preferentially observed in MALDI (even though basicity is not the only factor). Additionally, multiple proton coordination further increases the total proton affinity. In contrast, oligonucleotides are weakly acidic and are more readily detected in negative polarity as deprotonated molecules. In this case, deprotonated matrix anions exhibit relatively high proton affinity, which results in a high yield of analyte anions.

###### 2.2.1.2.4 Cationization

Cationization refers to the ionization of analytes through the formation of complexes with cations. Unlike protonation, cationization typically involves alkali metal ions (such as Na^+^ and K^+^) or transition metal ions (such as Cu^+^ and Ag^+^), which can establish stable complexes with the analytes.^208,228^ It is important to note that cationization requires the presence of the corresponding cations in the sample; therefore, in certain cases, the addition of suitable salts (as cation sources) to the MALDI sample can significantly enhance the signals of the resultant analyte–cation complexes.

###### 2.2.1.2.5 Electron Transfer

Electron transfer is used for the analysis of low-polarity molecules, which are often detected as deprotonated species and radical anions.^238^

###### 2.2.1.2.6 Multi-Charge Ionization

Multi-charge ionization occurs when analytes can acquire multiple charges during the ionization process. This phenomenon is analogous to the reduction of polyvalent cations; however, generating higher charge states becomes increasingly difficult due to the associated electrostatic repulsion.^208^ This is reflected in the basicity of biomolecules, which decreases steadily and sharply with increasing preferential protonation. However, when charges can be widely separated, the internal repulsion energy of multiply-charged ions will decrease. Therefore, MALDI typically produces +1 or higher charge states, and the probability increases with increasing molecular size.

###### 2.2.1.2.7 Summary

In summary, secondary ionization plays a crucial role in the MALDI process, significantly influencing the detection and analysis of various analytes through complex interactions between matrix–analyte and analyte–analyte pairs, thereby determining the diversity of the observed ion species. Key phenomena such as MSE and ASE highlight the intricate dynamics of ionization, while processes including proton transfer, cationization, electron transfer, and multicharge ionization showcase the various mechanisms by which analytes are ionized. Overall, a comprehensive understanding of these interactions is essential for optimizing matrix selection, reducing ion suppression, enhancing analytical coverage, and ultimately guiding the rational design of novel matrices to achieve finer control over secondary ionization pathways.

#### 2.2.2 Inorganic Matrix Ionization Mechanisms

The ionization mechanisms of inorganic matrices differ from those of organic matrices. Clarifying the desorption and ionization aspects of inorganic matrices remains challenging due to the various factors affecting their analytical performance. These include the surface morphology (shape, size, and porous nanostructure)^239^ and physicochemical properties (light absorption efficiency, thermal conductivity, and melting point)^240^ of inorganic matrices; the chemical properties of analytes and their interactions with nanosubstrates;^65^ and experimental operational parameters such as laser characteristics (pulse irradiance, wavelength, number of pulses, pulse length, pulse energy, and pulse frequency) and ionization mode (positive or negative)^65^. Despite these complexities, consensus exists that inorganic matrices play a major role in the D/I mechanism by absorbing laser energy, which causes a rapid and sharp increase in the surface temperature. Both thermal and non-thermal processes may be involved in the entire MALDI-MS process.^241,242^ In short, the ionization of inorganic matrices mainly follows a thermally driven mechanism,^71,243^ however, nonthermal mechanisms such as phase transitions have been also proven reasonable explanations.^240,244^

##### 2.2.2.1 Thermal Ionization Mechanisms

The desorption process of inorganic matrices is widely regarded as a laser-induced thermally driven phenomenon. In their initial MALDI-MS study, Tanaka et al. suggested that the main mechanism of ion detection might originate from thermal processes, referring to the thermal properties of ultrafine particles, such as low heat capacity, high light absorption rate, and large surface area.^242^ Wei et al. also emphasized the importance of thermal processes, indicating that the high surface area, strong UV absorption, and pore characteristics of porous silicon underlie its efficiency in macromolecule desorption and ionization.^71^ Chiang et al. further proposed that compared with bulk materials, nanomaterials (NMs) may provide signal enhancement in MALDI-MS, owing to their larger surface area and high molar absorption coefficients.^245^ The thermally driven phenomenon may be based on the rapid heating of inorganic NMs and the thermal confinement effect generated by the interaction between nanosecond pulsed lasers and nanostructures.^65^ Therefore, under laser irradiation, the local temperature around inorganic matrices, especially NMs, can become extremely high, sufficient to desorb most analytes. Inorganic matrices are thus characterized by strong UV–vis absorbance under MALDI laser wavelengths, low heat capacity, and reduced thermal conductivity (related to their size, surface roughness, and electronic thermal conductivity), and may play an active role in this mechanism.^242^ The local thermal density must be high, while the thermal conductivity must be low to prevent rapid dissipation of “thermal spikes”,^246^ allowing the effective energy transfer from the inorganic matrix to the analyte, leading to induce efficient desorption and signal enhancement in MALDI-MS.^242^

##### 2.2.2.2 Nonthermal Ionization Mechanisms

In addition to the thermally driven ionization mechanism, nonthermal processes are also commonly considered as possible mechanisms for the desorption and ionization of inorganic matrices in MALDI-MS. However, the exact mechanism remains largely unclear because various pathways can promote ionization,^247^ such as thermionic emission, charge transfer, phase transition, and nanophotonic ionization.

###### 2.2.2.2.1 Thermionic Emission

Plasmonic NMs (such as gold, silver, and platinum-based NPs) exhibit high photochemical activity under UV–vis laser irradiation.^248^ They convert light energy into chemical energy by generating high-energy electrons (termed hot electrons)^249^ and pairing them with holes.^250^ Under nanosecond pulsed laser excitation, noble metal nanoclusters release a large number of electrons and become positively charged.^251^ When Coulomb repulsion between these charges exceeds the cohesive force within the nanocluster, the NPs carry too much charge and eventually become unstable, ultimately undergoing Coulomb explosion, fragmenting spontaneously,^251^ and ejecting both electrons and nanosubstrate ions.^252^ Recently, Cheng and Ng proposed the simultaneous generation of holes along with hot electrons.^250^ This mechanism involves the formation of a positively charged hole-containing nanosubstrate through the transfer of hot electrons from the substrate to a conductive support (*e.g.*, a MALDI plate adapter). These holes reduce the interaction between the analyte ions and the nanosubstrate surface and enable Coulomb repulsion between the positively charged nanosubstrate and the analyte ions, enabling analyte desorption in the positive-ion mode.

###### 2.2.2.2.2 Charge Transfer

Charge transfer (encompasses proton, cation, or electron transfer) is another fundamental ionization pathway in inorganic matrix MALDI-MS.^225^ Both the composition and surface functionalization of NMs have a crucial impact on charge transfer, thereby influencing ionization.^253^ Shi et al. demonstrated this using HO-GD as the MALDI medium with dipeptide Ala-Gln and glucose molecules as model compounds. They revealed that charged ion transfer reactions occurred between HO-GD and adjacent analytes in both positive- and negative-ion modes.^254^ Under laser irradiation, the carbon-free oxygen-containing groups dissociated from HO-GD as either radicals (OH•) or anions (OH^–^) and participated in complex photochemical reactions. In the negative-ion mode, they facilitated the deprotonation of the terminal carboxyl group of the analyte, generating negatively charged molecular ions. Conversely, in the positive-ion mode, hydroxyl-mediated metal ion transfer promoted strong interactions between metal ions and nearby analytes, forming cationic products.^254^ Electron transfer also plays an important role in MALDI. Using juglone as a model analyte, the photoelectron transfer process on ZnO was studied recently in the negative-ion mode by alternating bias induced fragmentation. As the bias voltage increased, more product ions were observed and assigned, indicating continuous electron transfer into the absorber molecules.^255^ Furthermore, plasmonic hot electron transfer in the negative-ion mode has been proposed as an ionization pathway for isolated AgNIs supported by nanodiamonds.^256^ Comparisons of the ionization effects of different NMs on juglone and indigo molecules demonstrated that, electron transfer plays an important role in the ionization of molecules with high electron affinity.

###### 2.2.2.2.3 Phase Transition

The role of phase transition, also known as laser-induced surface reorganization or destruction, in inorganic matrix MALDI-MS was first demonstrated on carbon NMs in 2009.^257^ Carbon-based materials such as graphite, graphene, fullerenes, carbon nanotubes, carbon dots, and diamonds exhibit different physicochemical properties, such as thermal conductivity, specific heat capacity, and melting point. Tang et al. used phenylpyridine salt as a chemical thermometer to systematically study the influence of different carbon substrates on ion desorption efficiency.^257^ They revealed that the ion desorption efficiency is inversely proportional to the degree of internal energy transfer, a phenomenon inconsistent with traditional thermal desorption mechanisms, indicating the involvement of a nonthermal phase-transition-related desorption mechanism. Metal NMs, another major class of inorganic matrices, have medium melting points and may be melted under laser ablation. Using phenylpyridine salt as a model compound, it was confirmed that ion desorption in the positive-ion mode is related to two internal energy transfer pathways: thermally driven desorption and phase-transition-driven processes. Different metal NPs significantly impact internal energy transfer, which strongly depends on their physicochemical properties, including light absorption efficiency, melting point, and binding affinity of the metal NPs.^244^ Using AuNPs as model NPs, in the positive-ion mode, thermally driven melting, vaporization, and phase explosion processes were further studied, revealing that phase explosion plays the most important role in ion desorption.^240^ Song and Cheng made an analogy between the ionization mechanism of inorganic matrix MALDI-MS and laser ablation, arguing that when sufficient laser energy is absorbed, the surface melting/destruction that occurs resembles laser ablation processes.^242^ Laser ablation is a nonthermal process for generating plasma, triggered by laser irradiation of the sample surface. It involves surface melting, dissociation, vaporization, ionization, and shock-wave removal.^242^ The resulting laser-induced plasma contains various substances, such as electrons, neutrals, excited neutrals, and ions. Within this dense plasma, gas-phase collisions may occur between the contained high-energy substances, possibly inducing further ionization, thus providing an additional pathway for analyte ion formation in MALDI-MS.

###### 2.2.2.2.4 Nanophotonic Ionization

With the development of nanophotonics, the role of photons in the interaction between lasers and nanostructures has attracted increasing attention. A key observation is that nanopost arrays (NAPAs), with subwavelength diameters and specific aspect ratios, can induce quasi-resonant effects under laser irradiation.^258^ This resonance effect enables nanostructures to efficiently capture and amplify laser energy, which is then concentrated on the adsorbed analyte molecules, significantly enhancing the efficiency of laser desorption ionization. Vertes et al. fabricated uniform NAPAs via ultrafast laser processing, facilitating rapid quantitative analysis of complex mixtures, such as resveratrol in red wine, and enabling in situ detection of metabolites within individual yeast cells.^258^ These results highlight the unique advantages and vast potential of nanostructure engineering in enhancing the performance of MALDI-MS.

###### 2.2.2.2.5 Summary

This section summarized the main hypotheses explaining the fundamental mechanisms of inorganic matrices in MALDI. Collectively, these models offer valuable but partial insights into the ionization mechanism of inorganic matrix based MALDI-MS, and no unified consensus has yet been reached. The evidence suggests that inorganic matrix MALDI-MS possibly involves an intricate interplay between thermal and non-thermal energy transfer processes. To fully elucidate these mechanisms, in-depth and systematic studies are essential to disentangle these convoluted processes and clarify the underlying physical principles. Such investigations are essential for the rational design and development of optimized inorganic matrices with improved analyte ionization efficiency and specificity, expected to significantly enhance the analytical performance of MALDI-MS. These advancements will broaden the applicability of MALDI-MS across diverse scientific disciplines and research fields.

## 3 Matrices for MALDI-MS

### 3.1 Organic Matrices

MALDI-MS has emerged as a transformative tool in analytical science, with matrix selection being a critical determinant of its effectiveness.^11^ The matrix is essential in MALDI-MS, as it absorbs laser energy and facilitates the transfer of that energy to analytes, enabling their ionization.^259^ Among all matrix types, organic matrices stand out because of their exceptional UV absorption properties, diverse structural functionalities, and broad applicability, making them a focal point of study and practical application. This section is dedicated to the exploration of organic matrices in MALDI-MS, encompassing a diverse array of categories from SOM matrices to engineered and synthesized matrices, including matrix derivatization, reactive matrices, ionic liquid matrices, and binary and hybrid organic matrices (Tables S1 and S2). It presents a systematic overview of organic matrices classification and performance under different structures, laying a robust foundation for matrix selection and development.

#### 3.1.1 Small Organic Molecule Matrices

SOMs matrices play an integral and important role in the field of MALDI-MS.^260^ Structurally, the molecular weight of SOMs is typically below 1,000 Da, offering superior solubility and stability. However, there are exceptions, such as lignin with a molecular weight of several thousand, which also exhibits unique advantages in MALDI-MS. Functionally, they absorb laser energy and convert it to ionization energy, facilitating the ionization of sample molecules during MALDI-MS. In addition, SOMs are capable of acting as ion sources (*e.g.*, protons, and sodium ions) to increase the intensity of the MALDI-MS detectable ion signal and improve the quality of the mass spectra. So in short, SOMs are crucial in sample preparation and MS analysis, enabling accurate and sensitive MALDI-MS and MALDI-MSI of biomolecules.

MALDI-MS enables the analysis of compounds in both positive and negative ion modes, depending on the experimental requirements.^261^ In positive-ion mode, the molecules to be tested are usually detected as protonated forms ([M+H]^+^) or cations ([M+Na]^+^, [M+K]^+^), whereas in negative-ion mode, the molecules to be tested are usually detected in deprotonated forms ([M–H]^−^). Despite differences in detection sensitivity between positive and negative ion modes, these are primarily attributable to the structural variations in the analyte, but not to ionization mode itself. Therefore, the choice of positive and negative ion modes depends on the structural characteristics of the molecules to be measured, with some molecules being more susceptible to ionization as positive ions and others being more susceptible to ionization as negative ions.^262^ Generally, the sensitivity of negative ion detection is lower than that of positive ion detection. Depending on the actual needs and sample characteristics, the correct choice of ion mode can lead to more accurate and detailed analysis results, helping to fully understand the chemical structure and properties of the sample. SOMs vary in ionization tendencies, as their different structures and functional groups make them suitable for specific ionization modes and target molecules. The SOMs used for analyzing compounds were classified into fourteen major groups according to their structures: cinnamic acid based matrices, acetophenone based matrices, thiazole based matrices, benzoic acid based matrices, anthracene based matrices, flavonoid based matrices, coumarin based matrices, pyridine based matrices, benzophenone based matrices, nitrobenzene based matrices, naphthalene based matrices, pyridazine based matrices, indole based matrices, and other SOM matrices (Figure 2 and Table S3). We describe these types in detail in this section, which will assist readers in better understanding the role of SOMs in MALDI-MS analysis and making suitable selections. In this section, we present a comprehensive and in-depth account of these types. This detailed exposition is crucial as it enables us to gain a more profound understanding of the role that SOMs play in MALDI-MS analysis. Moreover, it empowers us to make judicious matrix selections by taking into full consideration the differences among research target molecules and experimental objectives.

##### 3.1.1.1 Cinnamic Acid Based Matrices

Cinnamic acid, which is found in cinnamon, is a natural plant metabolite. As a monocarboxylic acid, cinnamic acid is composed of acrylic acid with phenyl substituents.^263^ The structure of cinnamic acid is unique in that its stereochemistry can be changed by rotating the acrylic group. Specifically, when the acrylic group is attached to the benzene ring in the cis conformation, it is called *Z*-type (*cis*) cinnamic acid; when the acrylic group is attached to the benzene ring in the trans conformation, it is called *E*-type (*trans*) cinnamic acid. The different locations of the acrylic group in *Z*-type cinnamic acid and *E*-type cinnamic acid affect the stereochemical structure and properties of the molecule. This difference in stereochemistry leads to cinnamic acid compounds showing different properties when used as MALDI substrates, even between isomeric substrates of the same cinnamic acid. Cinnamic acid analogs can act as MALDI matrices, and the number and position of substituents in the aromatic part are extremely crucial as they govern the properties of the MALDI matrix itself. For instance, structural changes in the CHCA matrix molecule have a notable influence on the degree of ionization and the detection range of the analytes in MALDI.^57,264^ As shown in Figure 8, the main cinnamic acid compounds used as MALDI matrices are SA, FA, CA, CHCA, 3,4-dimethoxycinnamic acid (DMCA), and curcumin. The first five of these are phenylpropanoids. Phenylalanine analogs are a variety of organic compounds synthesized from phenylalanine and are widely used as matrices for the detection of proteins and small-molecule compounds.

**Figure 8:**
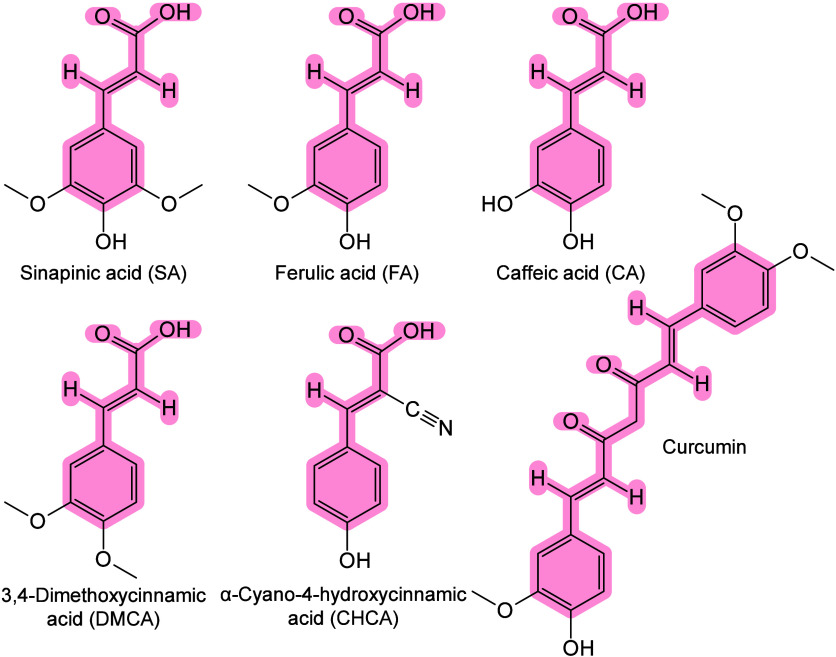
Chemical structures of cinnamic acid and analogue based matrices. The similar structural feature shown in these matrices is highlighted in red color.

SA’s chemical structure contains one carboxyl group (−COOH), one hydroxyl group (−OH), two methoxy groups (−OCH_3_), and an allyl side chain. SA is found mainly in plants, especially in crops like mustard seeds and sesame seeds.^265^ Since its introduction as a MALDI matrix, SA has been widely used, especially in the MALDI-MS analysis of proteins and peptides. For instance, Nelson et al. used SA as a matrix in MALDI-MS and successfully analyzed the average molecular weights of two monoclonal human immunoglobulins (IgM), which were 939,000 ± 2,000 and 982,000 ± 2,000 Da.^266^ In addition, Schlosser et al. utilized SA to detect immune complexes formed by antibodies to β-lactoglobulin and polyclonal-β-lactoglobulin in the gas phase by MALDI-MS and confirmed the specific formation of antibody–antigen complexes.^267^ Although SA is widely used in MALDI-MS for protein/peptide analyses, the presence of more numerous matrix clusters restricts the detection of short peptides. By exploring the SA isomer *Z*-SA, however, Salum et al. successfully detected short peptides by MALDI-MS and observed a small number of clusters in the low *m*/*z* region, providing an efficient tool for the direct, rapid, and sensitive detection of LMW peptides.^268^ Additionally, compared with the commonly used *E*-SA, *Z*-SA showed superior performance in the analysis of neutral/sulfated carbohydrates, extending the applicability of SA for carbohydrate MALDI-MS analysis.^269^

FA, which is chemically known as 3-methoxy-4-hydroxycinnamic acid, belongs to the same category of hydroxycinnamic acid derivatives as SA. FA has two isomers, cis and trans, and both are light yellow solids. It is a common phenolic acid in the plant kingdom and an important component of Chinese herbal medicines such as *Angelica sinensis* and *Rhizoma Ligusticum chuanxiong*.^270^ FA has cinnamic acid as the basic skeleton, with methoxy and hydroxy substituents at the 3rd and 4th positions on the benzene ring, respectively. FA is a commonly used MALDI matrix, particularly in the detection of nucleotides and proteins. FA as a matrix is an effective choice for MALDI-MS analysis of fluorophore-labeled oligonucleotides for the rapid screening of analytes.^271^ Stverakova et al. successfully identified intact staphylococcal phages by MALDI-MS using FA as a matrix and developed a rapid and reliable method to identify staphylococcal phages for the detection of their specific proteins through MALDI-MS analysis.^272^ FA halides showed superior sensitivity and selectivity for peptides. Kato et al. found that the 6-position of FA is optimal for the introduction of bromine, and 6-bromo-ferulic acid (6-BFA) can be used as a novel matrix to efficiently detect cyanocobalamin and a variety of peptides with good sensitivity and selectivity, especially large peptides and peptides containing acidic AAs or proline.^273^

CA is a hydroxycinnamic acid derivative chemically known as 3,4-dihydroxycinnamic acid. Its benzene ring is substituted with hydroxyl groups at positions 3 and 4 and exists in both cis and trans forms, with the latter being more common.^274^ CA is a polyphenol produced through the secondary metabolism of fruits and vegetables, such as olives, coffee beans, fruits, potatoes, carrots, and propolis, and it is the major hydroxycinnamic acid in the human diet.^275^ CA was first reported as a MALDI matrix in 1989, and it has demonstrated good vacuum stability. Ronald et al found that the use of CA as a MALDI matrix in protein qualitative assays resulted in lower intensities of photochemical adduct peaks in protein quasimolecular ion signals, and these peaks were more easily distinguishable than those obtained in other matrices.^57^ In addition, CA has been used for the quantitative determination of proteins. For example, Bucknall et al. successfully determined growth hormone levels in rat pituitary tissue and insulin levels in human pancreatic tissue using CA as a matrix. This method has practical applications in quantitative biomedicine by MALDI-MS.^276^ However, prior to that, studies using CA as a matrix for MALDI-MSI *in situ* detection and imaging of biological tissue proteins had not been reported. In 2021, Liu et al. discovered that CA matrices enhanced the *in situ* detection and imaging of high molecular weight (HMW) proteins with molecular weights close to 20,000 Da in tissues by MALDI-MS. This was the first time that CA as a matrix had been successfully enhanced for the *in situ* detection and imaging of endogenous proteins in three biological tissue sections (*i.e.*, rat brain, *Capparis masaikai* seeds, and germinated soybean seeds) evaluated using MALDI-MSI.^35^ The results indicated that CA, with its strong UV absorption, ultra-wide mass detection range (close to 200,000 Da), μm-scale size matrix crystals, uniform matrix deposition, and high ionization efficiency, has the potential to become a standard organic acid matrix for enhanced tissue imaging of HMW proteins in animal and plant tissues by MALDI-MSI.

CHCA is a monohydroxy-cinnamic acid derivative. It belongs to the class of 4-hydroxycinnamic acid derivatives, in which the α-hydrogen on the carboxyl group is substituted by a cyano-group, conferring specific chemical properties and pharmacological activities. CHCA is typically employed in biomedical studies and other related applications. As a classic MALDI matrix, CHCA has been widely used for the analysis of proteins,^277,278^ oligonucleotides,^219,279^ glycopeptides,^280^ carbohydrates,^281,282^ and phosphopeptides.^283,284^ In their search for suitable MALDI matrices for peptides and proteins, Beavis et al. discovered that CHCA exhibits certain interesting characteristics as a matrix.^59^ Compared with the use of other cinnamic acid derivatives such as SA and FA, the use of CHCA as a matrix yields higher intensity protonated peptide signals, indicating that CHCA enables greater peptide ionization efficiency. Therefore, CHCA is an efficient matrix for MALDI-MS analysis of peptides and glycopeptides with masses ranging from 500 to 5,000.^59^ Moreover, Urso et al. successfully employed CHCA as a matrix for the quantitative detection of thymosin β4 in human cerebrospinal fluid via MALDI-MS.^285^

DMCA is a natural product found in asparagus, black poplar, and other organisms. DMCA is a derivative of *trans*-cinnamic acid, in which the 3′ and 4′ positions are substituted by methoxy groups. The methoxy functional group (−OCH_3_) refers to a methyl group (−CH_3_) covalently bonded to an oxygen atom (O).^286^ Studies have revealed that DMCA is a novel and effective matrix for MALDI-MSI-based detection of LMW compounds. He et al. discovered that DMCA, as a novel matrix, can enhance the *in situ* detection and imaging of LMW compounds in biological tissues using MALDI-MS.^33^ They successfully applied DMCA to improve the *in situ* detection of LMW metabolites and lipids in tissue sections of rat liver, rat brain, and taxus seeds. As a MALDI matrix, DMCA generates strong signals for LMW compounds in positive ion mode with minimal interference from matrix-related ion signals. This study provides a new and powerful matrix for enhancing MALDI-MSI analysis in both animal and plant tissues.

In addition to cinnamic acid derivatives, curcumin contains a structural unit similar to cinnamic acid, known as the cinnamoyl group. Curcumin is a diketone compound consisting of two polymerized aromatic acrylates, formally named 1,7-bis­(4-hydroxy-3-methoxyphenyl)-1,6-heptadiene-3,5-dione.^287^ At room temperature, curcumin appears as an orange-yellow crystalline powder that is insoluble in water but soluble in EtOH and propylene glycol, and readily soluble in glacial acetic acid, MeOH, EtOH, ethyl acetate, and alkaline solutions. Curcumin is commonly used as a colorant and food additive^288^ and has been extensively applied in various medical settings.^289–291^ Francese et al. demonstrated that curcumin is a versatile matrix suitable for MALDI-MSI applications, with its extended conjugated double-bond structure, making it a promising candidate for MALDI-MS matrices. They successfully employed curcumin for drug analysis, lipid imaging in skin and lung tissues, and the analysis of specific compound classes in fingerprints.^292^

Collectively, cinnamic acid derivatives exhibit excellent photophysical properties in MALDI-MS, efficiently absorbing laser energy, promoting the coupled desorption and ionization of analyte molecules within the co-crystal. The use of these matrices expands MALDI-MS’ scope (detectable mass range up to ∼200 kDa), improves ionization/imaging resolution, and enables diverse biomedical analyses. Future directions will likely focus on structure-activity optimization (*e.g.*, tailored substituents/isomers) to reduce background ion interference, enhance LMW/HMW analyte multiplexing, and standardize cinnamic acid based matrices for high-throughput MALDI-MS analysis in biological science.

##### 3.1.1.2 Acetophenone Based Matrices

Acetophenone occurs naturally in camellias, flowering almonds (*Prunus glandulosa*), and other organisms.^293^ Unlike common carboxylic acid-based matrices, acetophenone-based matrices are derived from the acetophenone scaffold, with multiple hydroxyl substitutions on the phenyl ring. The presence of multiple hydroxyl groups, in conjunction with their specific structure and position, facilitates the occurrence of intramolecular proton transfer in acetophenone-based matrices under UV irradiation, thereby providing protons for the ionization of analytes. Compared with ortho-isomers, para- and meta-substituted hydroxyl compounds exhibit significantly reduced or even no matrix activity. Acetophenone based matrices can be classified into four types: 2,4-dihydroxyacetophenone (2,4-DHAP), 2,5-dihydroxyacetophenone (2,5-DHAP), 2,6-dihydroxyacetophenone (2,6-DHAP or DHAP), and 2,4,6-trihydroxyacetophenone (2,4,6-THAP or THAP) (Figure 9). Among these compounds, the first three are structural isomers of dihydroxyacetophenone.

**Figure 9:**
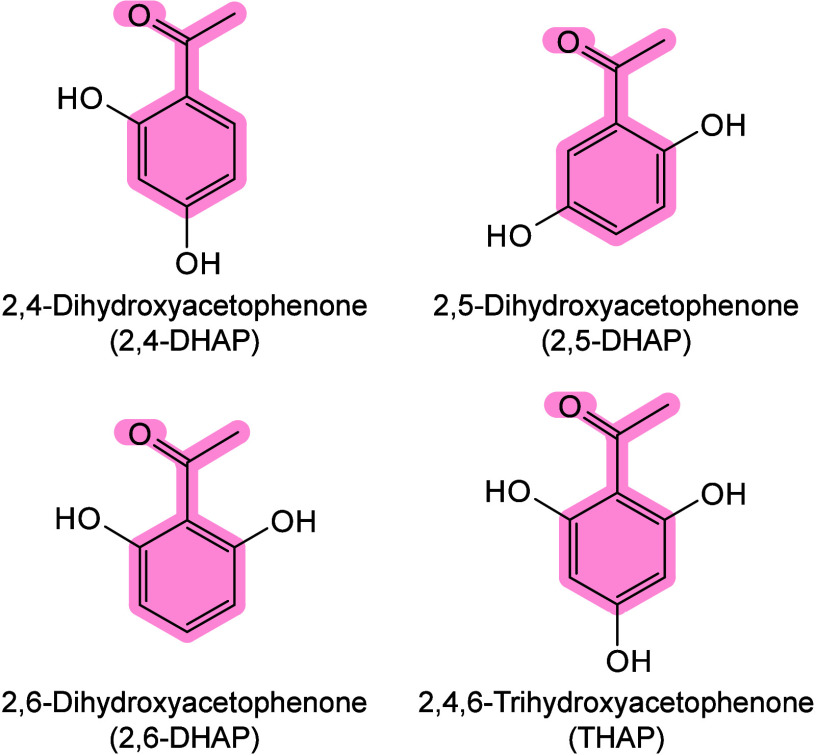
Chemical structures of acetophenone based matrices. The common structural feature shown in these matrices is highlighted in red color.

2,4-DHAP is a dihydroxyacetophenone, featuring hydroxyl substituents at the 2′ and 4′ positions on the acetophenone structure. As a MALDI matrix, 2,4-DHAP is typically used for protein analysis.^294^ For instance, Garozzo et al. reported that when 2,4-DHAP was dissolved in H_2_O/CAN, it yielded spectra with higher peak intensities and exhibited better performance in terms of resolution and reproducibility. They further employed 2,4-DHAP as a matrix for the MALDI-MS analysis of HMW glutenin subunits from three wheat varieties.^294^ Analytes undergo fragmentation during the MALDI ionization process resulting in the so-called in-source decay (ISD), which often complicates the resulting mass spectra. However, this fragmentation can be controlled and effectively applied to the analysis of nucleic acids. Specifically, when used as a matrix for ISD analysis, 2,4-DHAP enables the sequencing of ribonucleic acid (RNA) metabolites, yielding characteristic fragment ions that facilitate detailed structural elucidation. For example, Shimizu et al. investigated the application of high-resolution ESI and MALDI MS in the analysis of small interfering RNA (siRNA) duplex metabolites. Their results indicated that the combination of 2,4-DHAP with high-resolution ESI-Orbitrap and MALDI-MS facilitated the analysis of siRNA compound metabolites.^295^

2,5-DHAP is an acetophenone with hydroxyl substituents at the 2′ and 5′ positions that occurs naturally in plants such as *Cynanchum wilfordii* and *Ganoderma applanatum*. Krause et al. reported that 2,5-DHAP performs well as a matrix in UV-MALDI-MS experiments, achieving a resolution greater than 1,000, with particularly remarkable results in the MALDI-MS analysis of bovine insulin.^296^ Consequently, the use of 2,5-DHAP as a matrix provides excellent resolution and sensitivity in negative ion mode, making it an effective matrix for the analysis of peptides, proteins,^297,298^ and oligosaccharides.^299^ Additionally, Wenzel et al. demonstrated that 2,5-DHAP is also a suitable matrix for high-sensitivity MALDI-TOF-MS protein analysis, enabling the high-sensitivity detection of peptides, proteins, and glycoproteins on Anchor-Chip targets.^300^

2,6-DHAP is an acetophenone with hydroxyl substituents at the 2′ and 6′ positions. This natural compound is found in *Daldinia eschscholtzii*, *Euphorbia lunulate* Bunge, and other organisms. 2,6-DHAP is suitable for investigating the quaternary structure of proteins and noncovalent protein complexes.^301,302^ Moreover, 2,6-DHAP has been introduced into MALDI-MSI for lipid analysis^303^ and has been used as a matrix for the automated MALDI-MS analysis of LMW proteins and peptides.^298^ Jacksen and Emmer evaluated the effectiveness of 2,6-DHAP in the MALDI-MS analysis of hydrophobic proteins and peptides. They reported that 2,6-DHAP performed well in analyzing intact bacteriorhodopsin and chemically digested bacteriorhodopsin.^304^ Additionally, Gorman et al. discovered that combining 2,6-DHAP with diammonium hydrogen citrate (DAHC) can prevent the fragmentation of fragile peptides, disulfide bonds, and small proteins during MALDI-MS analysis.^305^ They further demonstrated that 2,6-DHAP/DAHC exhibits improved tolerance to buffer salts and other chemicals, particularly in experimental settings involving disulfide bond reduction. Furthermore, 2,6-DHAP/DAHC has potential applications in collision-induced dissociation analysis, enabling biomolecule ion acquisition without the need for ISD or post-source decay (PSD).^305^

THAP, a trihydroxyacetophenone, is characterized by the substitution of hydrogen atoms at the 2′, 4′, and 6′ positions of the phenyl group are replaced by hydroxyl groups. THAP is widely used as a matrix in MALDI-MS, particularly for the analysis of nucleic acids and carbohydrates.^306^ Its applications have also expanded to other unstable substances, such as noncovalent complexes, phosphorpeptides, sulfated peptides, and synthetic polymers. For example, Thiede et al. employed THAP as a matrix for the MALDI-MS detection of noncovalent RNA-peptide complexes, achieving comparable peak intensities for both peptides and RNA in negative ion mode, and successfully detecting RNA-peptide complexes.^307^ Using MALDI-MS, Pieles et al. found that THAP was highly effective for the mass and sequence analysis of both natural and modified oligonucleotides. They used THAP alongside diammonium and triammonium salts of organic or inorganic acids to suppress peak broadening caused by multiple ion adducts, enabling precise analysis and characterization of oligonucleotides.^308^ Currently, there are few reports on the use of matrices for the MALDI-MS analysis of polysaccharides whose molecular weights exceed 3,000 Da. Hsu et al. employed THAP as a matrix for the MALDI-MS analysis of polysaccharides, and demonstrated that this approach offers excellent performance for both derivatized and underivatized macromolecular polysaccharides and glycoproteins.^309^ Additionally, Stubiger and Belgacem utilized THAP as a matrix for lipid MALDI-MS analysis, and evaluated its potential as a novel matrix for analyzing various classes of lipids, including neutral storage lipids, polar membrane lipids, and sphingolipids (SLs).^310^

In conclusion, acetophenone-based matrices have become essential for MALDI-MS. Their structure, a phenyl-substituted acetone scaffold with multiple hydroxyls on the phenyl ring, allows intramolecular proton transfer under UV irradiation. Ortho-substituted derivatives show higher matrix activity. Key members such as 2,4-DHAP, 2,5-DHAP, 2,6-DHAP, and 2,4,6-THAP address specific analytical challenges. These matrices have significantly expanded MALDI-MS capabilities. They enhance the detection of diverse analytes, from HMW protein subunits to metabolites, peptides, oligosaccharides, and lipids. Looking ahead, three main development trends are expected, First, structure-activity optimization using computational modeling to design derivatives that boost proton transfer efficiency and reduce matrix ion interference, especially for LMW analytes. Second, enabling multiplexed and spatially resolved analysis through engineered matrices for high-throughput MALDI-MS imaging. Third, integrating with advanced MS platforms like TIMS-MALDI-MS and high-field FT-ICRMS to resolve isomers and achieve ultra-high-resolution analysis. These trends will make acetophenone-based matrices vital for future MALDI-MS in biomedical and biotechnological studies.

##### 3.1.1.3 Thiazole Based Matrices

Thiazole is a five-membered heterocyclic compound composed of three carbon atoms, one nitrogen atom, and one sulfur atom.^311^ As an important nitrogen-containing aromatic heterocycle, thiazole exhibits significant biological and pharmacological activities, making it a focal point in pharmaceutical studies.^312^ Thiazole-based compounds are typically used as matrices in MALDI-MS analysis. Owing to their thiazole ring structure, they efficiently produce MALDI-MS signals and ionize analytes by cocrystallizing with them and transitioning into gas-phase ions under laser irradiation, facilitating MALDI-MS analysis. Given that most conventional matrices are acidic, incorporating neutral materials such as thiazole-based matrices could expand the options for analyzing acid-sensitive biomolecules. Thiazole-based matrices can be categorized into four types: 2-mercaptobenzothiazole (2-MBT), 5-chloro-2-mercaptobenzothiazole (CMBT), 5-ethyl-2-mercaptothiazole (EMT), and 5-amino-2-mercapto-1,3,4-thiadiazole (AMT) (Figure 10). These compounds feature a thiazole ring, which is a crucial structural element for their function as MALDI matrices.

**Figure 10:**
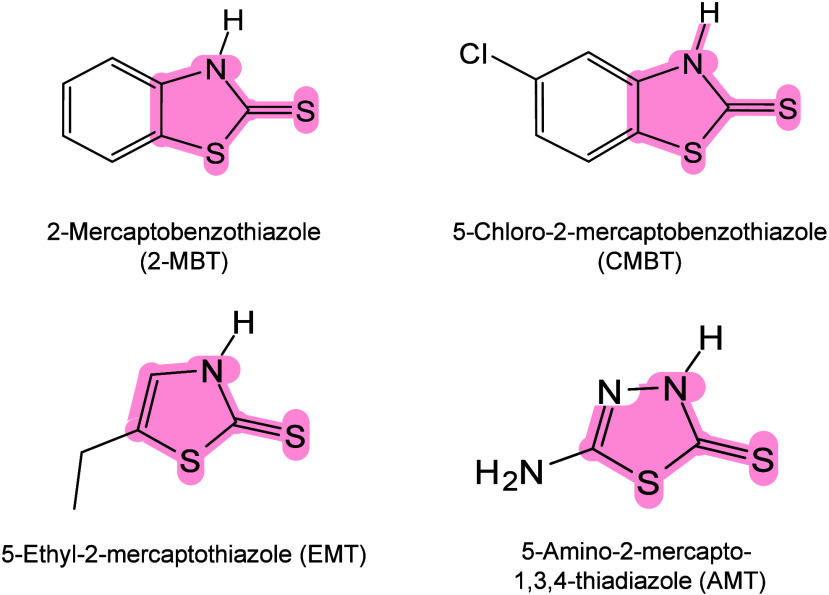
Chemical structures of thiazole based matrices. The similar structural feature shown in these matrices is highlighted in red color.

2-MBT is an organic compound featuring both a benzene ring and a thiazole ring, with a mercapto (-SH) group substituted at the 2′ position. 2-MBT appears as a pale yellow to brownish crystalline powder with a distinctive odor.^313^ As a matrix, 2-MBT has been successfully applied in the detection of macromolecular proteins and lipids, demonstrating its utility in MALDI-MS analysis. For example, Xu et al. reported that 2-MBT provided similar sensitivity and resolution to those of SA and CHCA but showed greater tolerance to sample contaminants such as ionic detergents.^314^ Astigarraga et al. demonstrated that 2-MBT is an excellent matrix with favorable detection sensitivity and stability, making it suitable for MALDI-MS of lipid extracts and tissues. MALDI-MS with 2-MBT, successfully analyzed and imaged lipids from brain and liver tissues, identifying numerous lipid species with high reproducibility in both positive and negative ion modes.^315^

CMBT typically appears as a yellow crystalline or powdery substance. As a derivative of 2-MBT, CMBT has a unique chemical structure that may enhance ionization efficiency and selectivity when it is used as a MALDI matrix.^316^ The presence of both mercapto and chloro substituents may facilitate the ionization process of sample molecules, potentially improving sensitivity and resolution. CMBT is suitable for analyzing a wide range of analytes, including peptides, low-mass proteins, glycolipids, oligosaccharides, and synthetic polymers. For example, Xu et al. reported that MALDI using CMBT as a matrix not only effectively analyzed peptides, low-mass proteins, and glycolipids but also outperformed MALDI using traditional matrices in the analysis of certain peptides and oligosaccharides.^314^ CMBT also enabled the analysis of complex carbohydrates, such as bacterial cell wall components derived from peptidoglycans.^317^ Additionally, the ability of CMBT to allow structural characterization and PSD analysis enables the detection and accurate mass determination of micropeptides at the sub-picomole to femtomole scale, providing structural information through PSD analysis.^317^ PSD, a metastable fragmentation technique in reflector TOF MS, generates sequence-informative fragment ions (*e.g.*, *b*- and *y*-type ions), thereby providing detailed structural insights.^318^ CMBT has been successfully applied in the sublimation process for matrix spraying techniques and used for biological tissue sample analysis. Yousefi-Taemeh et al. evaluated the stability of sublimated CMBT in a vacuum environment and utilized it for MALDI-MSI analysis of specific PLs in kidney tissue samples. The results demonstrated the potential of sublimated CMBT as a stable matrix for high-resolution imaging and analysis of biomolecules in complex tissue environments.^319^

EMT also features a thiazole ring structure, with an ethyl group substituted at the 2′ position and a mercapto group linked at the 5′ position.^320^ The thiazole ring structure may facilitate efficient ultraviolet laser absorption, contributing to an enhanced MALDI-MS signal. The metal-coordinating ability of the mercapto group may enhance the selectivity for specific sample types. EMT has been demonstrated to be an effective matrix for the MALDI-MS analysis of compounds within the molecular weight range of 500-6,000 Da. For instance, Raju et al. employed EMT as a matrix to perform MALDI-MS analysis in both positive and negative ion modes on various analytes such as Substance P, insulin, cyclodextrin, coconut oil TAGs, and polypropylene glycol 2000 (PPG2000).^321^ Additionally, this matrix has shown good performance in the MALDI-MS analysis of analytes with relatively HMW and hydrophobicity.^321^

AMT contains a thiadiazole ring structure with an amino functional group, as well as a thiol group attached at the 2-position. This structure may confer favorable ionization properties (due to the thiadiazole ring and amino group) and selectivity (through the thiol group), potentially resulting in enhanced stability and sensitivity in MALDI-MS analysis.^322^ Compared with alkali metal cations, AMT exhibits a higher proton affinity as a cationization agent. Owing to its noncarboxylic acid, mildly acidic (−SH), and basic (−NH_2_) functional groups, AMT can serve as a cationization matrix for the MALDI-MS analysis of carbohydrates. Mirza et al. demonstrated the high efficiency of AMT as a matrix for neutral carbohydrates in MALDI-MS, particularly for compounds with molecular weights of approximately 5,000 Da.^323^

Thiophene and thiazole are both heterocyclic sulfur compounds, specifically five-membered heterocycles containing sulfur atoms. They share similar structures and chemical properties and are applied in comparable fields of research within organic chemistry and materials science. The cyclic structure of thiophene compounds aids in absorbing laser energy, effectively facilitating the desorption/ionization (D/I) of analytes while also providing excellent optical and chemical stability in MALDI analysis, making these compounds ideal matrix candidates. Three existing thiophene-based matrices have been identified: 3-[5′-(methylthio)-2,2′-bithiophen-5-ylthio]­propanenitrile (MT3P), 2,3,4,5-tetrakis­(3′,4′-dihydroxyphenyl)­thiophene (DHPT), and 2-[5-(2,4-dichlorobenzoyl)-2-thienyl]­acetic acid (DCBTA) (Figure 11). Schinkovitz et al. demonstrated that MT3P, as a novel matrix for MALDI-MS, enables the highly selective ionization detection of alkaloids. Compared with commercially available matrices such as CHCA and DHB, MT3P showed superior ionization properties for 23 out of 25 alkaloids tested but exhibited minimal interactions with other compounds from various chemical origins.^324^ DHPT was proposed by Chen et al. as a novel matrix for the selective analysis of LMW amines and the direct determination of creatinine in urine. Their results indicated that DHPT has excellent selectivity for amine compounds and shows a low detection limit in positive ion mode.^325^ Yasuda et al. reported that a thiophene-containing compound, when used as a MALDI matrix, exhibited conductivity of the matrix crystal as a potential factor in efficient MALDI performance.^316^ DCBTA has been used as a matrix for detecting peptides, peptide mixtures, α-cyclodextrin, testosterone, and caffeine. However, the performance of DCBTA containing a thiophene group does not surpass that of the “gold standard” matrix (CHCA). Notably, the DCBTA molecule contains a thiophene group, similar to the structure of the dyes used in organic solar cells. Based on these observations, the conductivity of the matrix crystals for MALDI-MS was measured. Matrix crystals with electrical conductivity, such as DCBTA, CHCA, and SA, exhibit higher signal intensity and resolution in MALDI-MS. In contrast, nonconductive matrices like 2,5-DHB show lower signal intensity. The addition of conductive enhancers, such as MSA, to non-conductive matrices can significantly improve their conductivity, thereby enhancing the MALDI signal. This indicates that the conductivity of the matrix crystal is closely related to its performance in MALDI. Therefore, the conductivity of matrix compounds may be an important factor, and further investigation of this property could contribute to a better understanding of the MALDI process and the optimization of related methods.^132^

**Figure 11:**
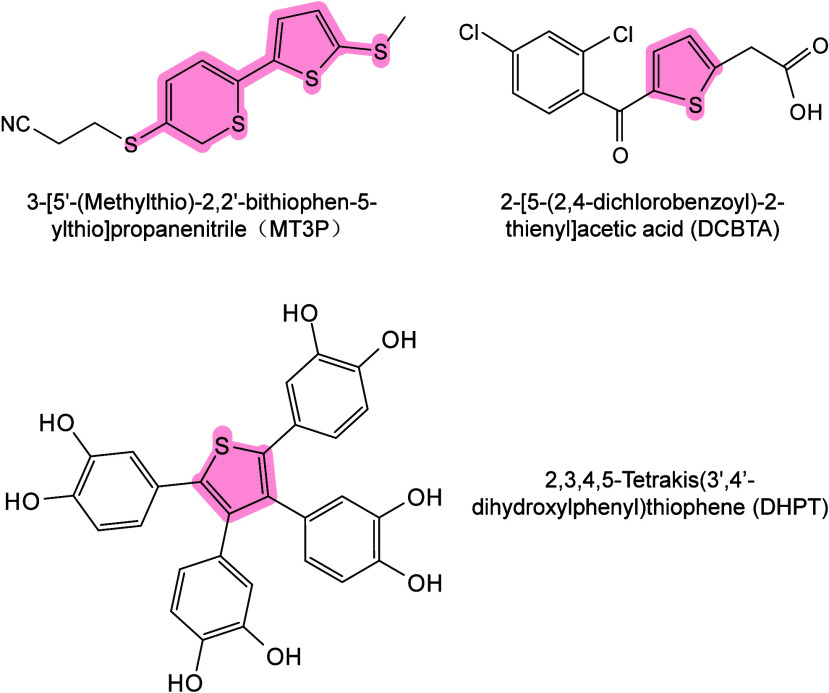
Chemical structures of thiophene based matrices. The similar structural feature shown in these matrices is highlighted in red color.

In summary, thiazole-based compounds, including 2-MBT, CMBT, EMT, and AMT, are widely used as matrices in MALDI-MS analysis. Thiazole-based matrices exhibit excellent ionization efficiency and stability and are therefore widely adopted for the analysis of various samples such as biomacromolecules, pharmaceuticals, and organic compounds.

##### 3.1.1.4 Benzoic Acid Based Matrices

Benzoic acid, which is an aromatic organic acid consisting of a benzene ring substituted with a carboxyl group, is the simplest aromatic acid. Its derivatives are widely employed as matrices in MALDI-MS owing to their strong UV absorption, which promotes efficient sample ionization upon laser irradiation.^326^ These matrices include DHB, nitrobenzoic acids, mefenamic acid (MA), 4-mercaptobenzoic acid (MBA), mono-hydroxybenzoic acids, 2-(4-hydroxyphenylazo)­benzoic acid (HABA), and 2,5-dihydroxyterephthalic acid (DHT) (Figure 12). Because of their exceptional stability and performance, benzoic acid-based matrices are particularly suitable for analyzing biomolecules such as proteins, peptides, and other organic compounds.

**Figure 12:**
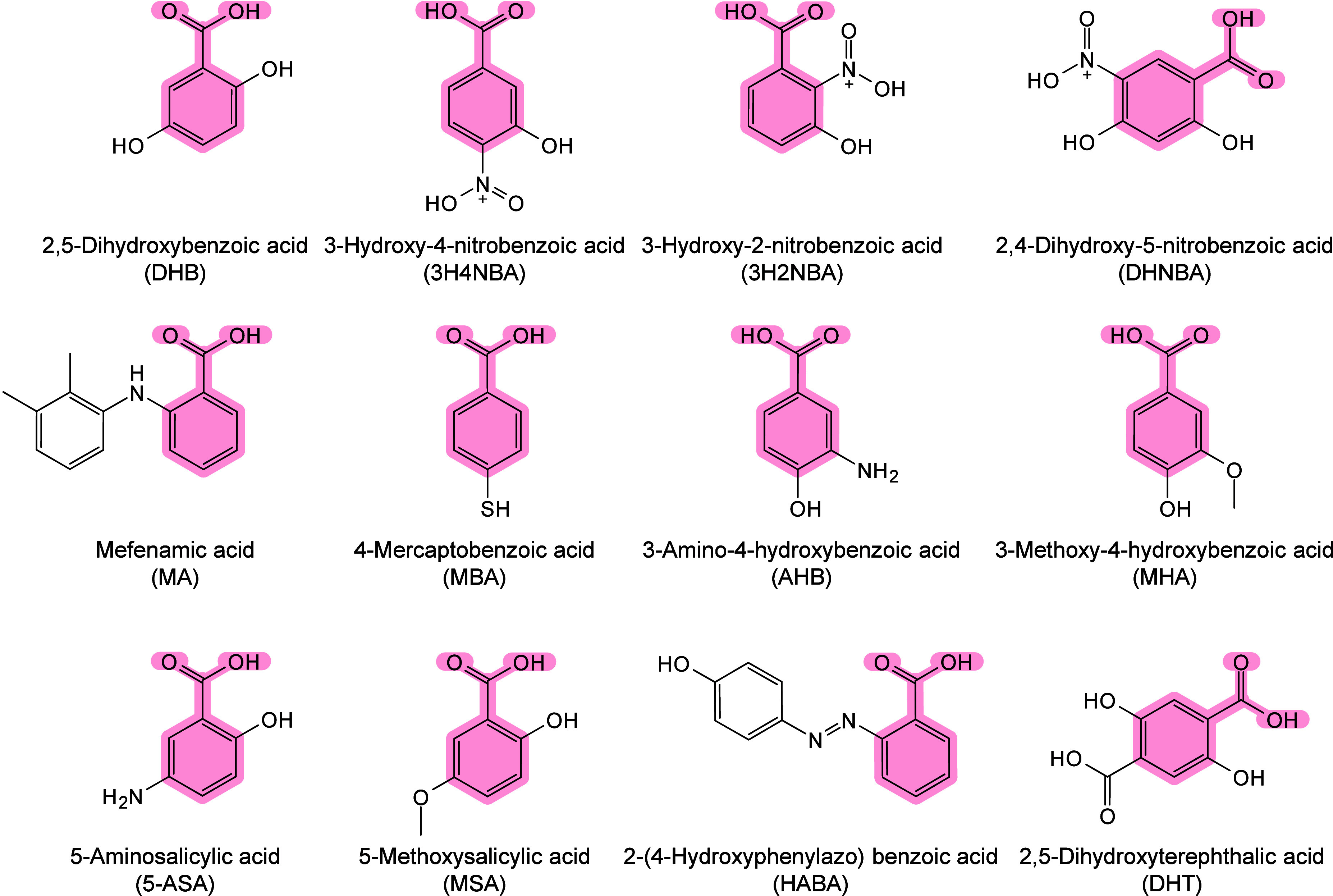
Chemical structures of benzoic acid based matrices. The common structural feature shown in these matrices is highlighted in red color.

DHB, a dihydroxybenzoic acid, was first introduced by Strupat et al. in the early 1990s as a novel matrix for MALDI-MS. The study provided a detailed account of its performance in protein analysis applications.^327^ Since its discovery, DHB has become a focal point of extensive study and is likely one of the most studied MALDI matrices to date. Over the past three decades, the application scope of DHB has expanded significantly, now covering nearly all fields of MALDI-MS study.^327–330^ For instance, Bourcier et al. conducted both experimental and theoretical studies on DHB in the context of MALDI, revealing that variations in matrix efficiency can be attributed to differences in chemical structure. The hydroxyl substituent groups in DHB play a crucial role in its unique efficacy as a MALDI matrix.^331^ Furthermore, Wei et al. were the first to construct highly uniform DHB layers as a matrix for MALDI-FTICR-MS-based lipidomic analysis, facilitating rapid studies on lipid variations within biological systems.^332^

3-Hydroxy-4-nitrobenzoic acid (3H4NBA) and 3-hydroxy-2-nitrobenzoic acid (3H2NBA) are two positional isomers. Both 3H4NBA and 3H2NBA are benzoic acid derivatives substituted with hydroxyl and nitro groups, distinguished by the positions of these substituents. In 3H4NBA, the nitro group is located at the 4-position of the benzene ring, whereas the hydroxyl group is at the 3-position. In 3H2NBA, the nitro group occupies the 2-position, and the hydroxyl group remains at the 3-position. These substituents confer distinct chemical properties and reactivities to the compounds. The presence of the nitro group imparts a strong electron-withdrawing effect, making the compounds highly electrophilic in certain chemical reactions.^333,334^ Moreover, the hydroxyl group contributes to the acidic nature and hydrophilicity of these compounds, further influencing their chemical behavior.^335,336^ Matsuo et al. discovered that 3H4NBA, as a novel matrix, enables the selective detection of 2-nitrobenzenesulfenyl (NBS)-labeled peptides through MALDI-TOF-MS. Their findings revealed that in mass spectrometric analysis involving NBS methods and compounds with nitrobenzene rings, the 3H4NBA matrix exhibits clear advantages in simplicity, sensitivity, and availability, making it more suitable than DHB for MALDI-TOF-MS detection of NBS-labeled peptides.^337^ Fukuyama et al. evaluated a series of 3H4NBA isomers and found that all these isomers (2H3NBA, 2H4NBA, 2H5NBA, 2H6NBA, 3H2NBA, 3H5NBA, 4H2NBA, 4H3NBA, 5H2NBA, and 3H4NBA) could generate *a*-series ions accompanied by *d*-series ions, in which 3H2NBA, 3H5NBA, 4H2NBA, 4H3NBA, and 5H2NBA were first verified as oxidizing matrices for ISD.^338^ Notably, the intensity of the *a*-series ions produced by 3H2NBA is nearly equivalent to, or even exceeds, that of the *a*-series ions generated using 1,5-diaminonaphthalene (DAN). In some cases, the peak resolution of the *c*-series ions produced by 3H2NBA is significantly higher than DAN. Consequently, 3H2NBA, as one of the most effective oxidizing substrates, holds promise for contributing to ISD analysis in MALDI-MS. Additionally, Chen et al. identified 2,4-dihydroxy-5-nitrobenzoic acid (DHNBA) as an innovative matrix that improves the sensitivity of MALDI-MSI for the *in situ* detection and imaging of hormones within plant tissues.^184^ Compared with the commonly used DHB matrix, DHNBA offers stronger UV absorption, negligible matrix background interference, and high ionization efficiency for plant hormones, positioning it as a powerful MALDI matrix with promising potential.

MA is an aminobenzoic acid derivative in which one of the hydrogen atoms attached to nitrogen is substituted by a 2,3-dimethylphenyl group. The suitability of MA as a matrix arises from its strong UV absorption capacity and crystalline properties. Its molecular structure, which contains a carboxylic acid functional group and an aromatic ring, enables effective laser energy absorption and stable ion production, resulting in clear MALDI-MS signals.^339^ MA provides several advantages in the MALDI-MS analysis of small-molecule compounds (such as adenosine triphosphate (ATP), geldanamycin, glutathione, and sulfamethoxazole), including high ionization efficiency, low cost, ease of uniform crystallization, reduced interference, low fragmentation, and high vacuum stability.^339^ However, despite its potential as a MALDI matrix in specific applications, MA is not the most common or preferred matrix choice in general MALDI-MS applications.^339^

MBA is a probe molecule containing thiol and carboxyl groups capable of forming self-assembled monolayers for the development of surface-enhanced Raman spectroscopy sensors. In MALDI-MS analysis, the thiol (−SH) group in MBA can complex with metal ions, facilitating analyte ionization. This characteristic makes MBA effective at promoting electron transfer and enhancing ionization efficiency in LDI processes involving various metals.^340–342^ Sun et al. first employed MBA as a MALDI matrix for the analysis of metal ions such as mercury (Hg^2+^) and cadmium (Cd^2+^), observing its benefits of low matrix background interference, enhanced signal intensity, and high reproducibility, making it suitable for highly sensitive metal detection.^343^ Additionally, Shao et al. utilized MBA-assisted MALDI-MS for the sensitive quantification of cesium (Cs^+^) and strontium (Sr^2+^) in drinking water, achieving rapid detection, low background interference, and high reproducibility. This approach demonstrated significant advantages over the use of traditional organic matrices, highlighting the promising application prospects of MBA.^344^

Single-hydroxybenzoic acid matrices include 3-amino-4-hydroxybenzoic acid (AHB), 3-methoxy-4-hydroxybenzoic acid (3M4HBA) (also known as vanillic acid), and salicylic acid derivatives. AHB is a mono-hydroxybenzoic acid classified as a 4-hydroxybenzoic acid, with an additional amino substituent at the 3-position. AHB was first identified by Mock et al. as the earliest matrix enabling glycosaminoglycan analysis via MALDI-MS,^345^ specifically for detecting free glycans released from glycoproteins. However, AHB was quickly supplanted after the discovery of DHB in 1991, which offered higher ionization efficiency and broader applicability^327,346^ and is now the gold standard matrix for glycan analysis. Few studies utilize AHC for this purpose.^347,348^ To enhance the performance of AHB as a MALDI matrix, Urakami and Hinou explored Na-doped AHB for the direct MALDI-MS analysis of O-linked glycopeptides and intact mucins.^349^ They focused on AHB’s selective ionization capability for glycoproteins and its low noise characteristics in the low-mass range, which were further strengthened by the addition of Na to AHB. 3M4HBA is a monohydroxybenzoic acid, where the 4-hydroxybenzoic acid structure is substituted by a methoxy group at the 3-position. The hydroxyl group of 3M4HBA hydroxyl group provides a site for proton transfer, while the methoxy group increases the polarity of the matrix molecule, facilitating interactions with analytes.^350^ Beavis et al. reported that 3M4HBA is an efficient matrix for protein analysis by MALDI-MS, with excellent performance in dual ionization mode and strong performance with LMW proteins.^350^ Finally, 5-aminosalicylic acid (5-ASA), a salicylic acid derivative, is a monohydroxybenzoic acid with amino (−NH_2_) and hydroxyl (−OH) functional groups. These groups enable interactions with cations or molecules in the sample, enhancing the generation and stability of mass spectrometric signals. With a moderate molecular weight, 5-ASA produces minimal background interference signals and exhibits favorable light absorption properties, effectively absorbing laser energy and facilitating analyte ionization.^335,351,352^ Sakakura and Takayama utilized 5-ASA as a matrix in MALDI-MS to analyze the ISD and fragmentation characteristics of peptides and reported that the protonated molecules and fragment ion peaks generated by 5-ASA displayed sharper resolution. Additionally, no interfering peaks appeared in the ISD mass spectra of the peptides analyzed.^351^ Another salicylic acid derivative, 5-methoxysalicylic acid (MSA) is a salicylic acid substituted with a methoxy group at the 5-position. Distler and Allison identified MSA as a novel matrix for the MALDI-MS analysis of oligonucleotides. When combined with spermine (SPM), it reduces the need for desalting and, compared with other matrix and dopant combinations, the MSA/SPM matrix provides mass spectra with higher resolution, fewer fragments, and a reduced intensity of alkali ion adduct peaks.^353^

HABA is an azo compound featuring a benzoic acid group and a phenyl ring containing an azo bond, classified as a phenol, monocarboxylic acid, and monoazo compound. Juhasz et al. identified HABA as a novel matrix suitable for MALDI-MS and effectively applied to the desorption ionization of peptides, proteins, and glycoproteins, with a mass range extending up to approximately 250 kDa.^354^ Further studies have revealed that the HABA matrix offers high sensitivity and mass resolution in the analysis of permethylated glycolipid and synthetic polymers.^354–357^ Moreover, HABA extends the duration and reproducibility of ion production, resulting in minimal spatial variation in ion signals, allowing for the accumulation of hundreds of individual mass spectra from the same spot, which is particularly beneficial for larger protein samples.

DHT is a dihydroxylated derivative of terephthalic acid. It contains two hydroxyl (−OH) groups positioned at the 2nd and 5th positions on the benzene ring. DHT is an important intermediate in organic synthesis and is widely used in the production of colorants, fluorescent substances, and organic light-emitting polymers with high solubility. Owing to the presence of phenolic hydroxyl groups, DHT has some reducing effects on chemical reactions, and can form complexes with metal ions, making it important for catalysis and materials science.^358^ Fu et al. reported that DHT, as a matrix, effectively improves the detection and imaging performance of AAs.^359^ DHT has strong UV–vis absorption capability, a uniform matrix coating, and high vacuum stability. Using DHT as a matrix in MALDI-MS technology, all 20 protein AAs in human serum were detected, whereas other matrices such as DHB and CHCA enabled the detection of only 7 and 10 AAs, respectively. Moreover, the DHT matrix was the first to reveal the *in situ* spatial distribution of all 20 protein AAs and taurine in oyster tissues. Overall, the use of DHT as a matrix provides new possibilities for the application of MALDI-MSI technology in metabolomics.

In summary, benzoic acid derivatives play a pivotal role as MALDI matrices in MALDI-MS analysis. Their excellent UV laser absorption properties, high stability, broad applicability, and good solubility make them an ideal choice for mass spectrometric analysis of various biomolecules. Benzoic acid compounds effectively facilitate the ionization of sample molecules, generating stable ion signals and providing reliable support for MALDI-MS research. Consequently, benzoic acid derivatives provide essential functions in the field of biological MALDI-MS, where they are widely used.

##### 3.1.1.5 Anthracene Based Matrices

Anthracene derivatives, which are typically composed of multiple anthracene rings, possess a unique structure that provides excellent UV absorption properties during the MALDI process.^360^ Notably, the multiple hydroxyl functional groups of these compounds serve as effective proton donors, facilitating protonation during the ionization process. This characteristic enhances ionization efficiency and plays a significant role in generating a greater number of ion signals.^361^ Anthracene derivatives were first developed as MALDI matrices in 1993 by Juhasz et al.,^361^ who used various anthracene derivatives (dithranol (also known as 1,8-dihydroxy-9-anthrone or DT), 9-nitroanthracene (9-NA), and quinizarin) as matrices to study nonpolar polymers with ferrocene, ferrocenyl naphthalene, and ruthenocenyl naphthalene units via MALDI-MS (Figure 13). Subsequently, anthracene-based matrices were developed for the detection of other compounds, especially lipid molecules and fullerenes. Among them, DT is an anthracene compound derived by substituting hydrogen atoms at C-1 and C-8 with −OH groups and adding an oxygen substituent at C-9. DT appears as lemon-yellow plates or an orange powder. Le et al. utilized DT as a matrix for MALDI-FTICR-MS tissue lipid imaging and reported that DT facilitated the detection of a wider range of lipids, including lipid species that were undetectable using other matrices such as CHCA and DHB.^362^ 1,8,9-Trihydroxyanthracene (DIT) appears as yellow, odorless flakes or powder. DIT is a trihydroxyanthracene substituted with hydroxyl groups at positions 1, 8, and 9, making it a homolog of DT. Thomas et al. identified DIT as a promising matrix for the high-spatial-resolution MALDI-MSI of lipids. Their findings revealed that DIT efficiently detected a wide range of lipids in both positive and negative ion modes, offering advantages such as low background interference, high resolution, and strong vacuum stability.^363^ Additionally, 9-NA has also shown significant utility in MALDI-MS applications. Streletskiy et al. reported that 9-NA plays a crucial role as a matrix in the MALDI-MS analysis of fluorinated fullerenes, facilitating the qualitative analysis of complex fluorofullerene mixtures.^364^ Furthermore, Kotsiris et al. validated the effectiveness of 9-NA as a matrix for the MALDI-MS analysis of derivatized fullerenes, where it minimized analyte decomposition and enhanced analysis efficiency.^365^

**Figure 13:**
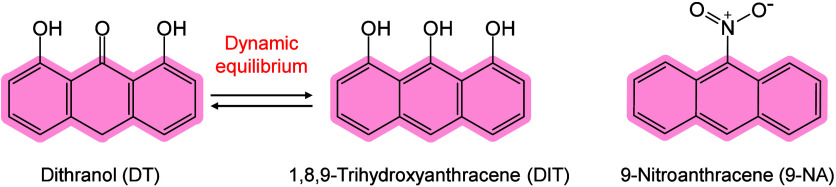
Chemical structures of anthracene based matrices. The common structural feature shown in these matrices is highlighted in red color.

In summary, anthracene derivatives offer several advantages as MALDI matrices. First, anthracene compounds exhibit strong light absorption properties, effectively capturing laser energy and converting it into thermal energy, thereby facilitating the ionization and dissociation of sample molecules. Additionally, anthracene-based matrices have low volatility, allowing them to remain stable under high vacuum conditions. This attribute enables it to function effectively within the high vacuum environment of conventional MALDI ion sources while maintaining constant physical and chemical properties. This consistency contributes to stable signal intensity and reproducible analytical results. Moreover, anthracene matrices demonstrate high molecular ion yield and minimal background noise, increasing the sensitivity and accuracy of mass spectrometric signals. Anthracene derivatives are typically used as matrices for the analysis of polymers and lipid molecules, thereby expanding the application scope of MALDI-MS.

##### 3.1.1.6 Flavonoid Based Matrices

Flavonoids are a diverse class of secondary metabolites widely found in plants, and exist in forms such as sugar-bound glycosides, C-glycosides, or free compounds.^366^ More than 10,000 flavonoid structures have been characterized to date, with a basic C6–C3–C6 skeleton comprising two phenolic rings (A and B rings) connected by a three-carbon bridge. The C3 component can be either an aliphatic chain or part of a five- or six-membered oxygen-containing ring with C6 segments.^367^ On the basis of variations in the ring structure, oxidation level, and substitution patterns in the C3 unit, flavonoids can be further classified into several subgroups, including chalcones (1), flavones (2), anthocyanidins (3), flavanones (4), flavonols (5), and isoflavones (6).^368^ Most flavonoids are phenolic compounds that are typically present in plants as glycosides. The aglycones of many flavonoids exhibit two strong UV absorption bands, commonly referred to as band I (300–380 nm) and band II (240–280 nm), and are generally weakly acidic compounds because of their chemical structure.^369,370^ Given that the UV laser wavelengths used in most MALDI instruments fall within the range of band I or II, these physicochemical properties suggest that flavonoid aglycones could serve as effective potential matrices for MALDI-MS analysis. Currently, the flavonoids confirmed to function as MALDI matrices fall into two main categories: chalcones and hydroxyflavonoids (including mono- and polyhydroxy compounds) (Figure 14).

**Figure 14:**
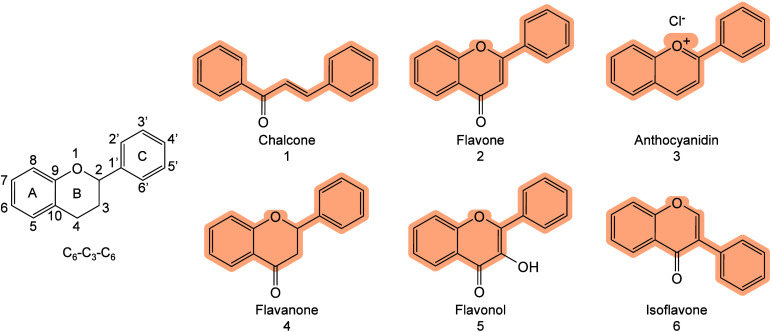
Chemical structures of the main flavonoid subclasses. The similar structural feature shown in these matrices is highlighted in orange color.

Chalcone, which is an aromatic ketone, serves as a biosynthetic precursor to flavonoids and isoflavonoids and is abundant in plants.^371^ Isoliquiritigenin (ISL), also known as 4,2′,4′-trihydroxychalcone, is a *trans*-chalcone hydroxylated at positions C-2′, -4, and -4′. ISL acts as a precursor to various flavonoid compounds in plants.^372^ Yang et al. identified ISL as a high-performance novel matrix suitable for the MALDI-MS analysis of neutral oligosaccharides.^373^ Compared with commonly used matrices, ISL provides superior analyte signals at lower concentrations and laser intensities and demonstrates tolerance to high concentrations of sodium chloride or urea, indicating potential applications in analyzing oligosaccharides within complex samples without the need for additional dopants or co-matrices.^373^

Flavone is the simplest member of the flavonoid compound class and consists of a 4*H*-chromen-4-one structure with a phenyl substituent at the 2-position. Flavones are benzo-γ-pyrone compounds lacking any hydroxyl groups or active hydrogen atoms.^374^ As a MALDI matrix, flavone itself does not generate ion signals because the transfer of laser energy to flavone molecules, or between flavone and any analyte, requires active hydrogen atoms.^51^ Although flavone itself cannot serve as a MALDI matrix, hydroxyflavones have emerged as excellent novel matrices for the MALDI-MS analysis of various compounds.^261^ Hydroxyflavone matrices are categorized into mono-hydroxy and poly-hydroxy flavone compounds.^375^ Mono-hydroxy flavones such as 3-hydroxyflavone (3-HF) and 5-hydroxyflavone (5-HF) have been used for MALDI-MS lipid analysis and tissue imaging.^51^ Poly-hydroxy flavones, which contain multiple hydroxyl groups, can form hydrogen bonds or other noncovalent interactions with target molecules, enhancing analyte stability and ionization efficiency in the matrix.^376^ The poly-hydroxy flavones that are used as MALDI matrices include two dihydroxyflavones (3,7-dihydroxyflavone (3,7-DHF) and chrysin), two trihydroxyflavones (7,3′,4′-trihydroxyflavone (7,3′,4′-THF) and apigenin), three tetrahydroxyflavones (fisetin, luteolin, and kaempferol), two pentahydroxyflavones (quercetin and morin), and rutin. The hydroxyl groups at the C3 and C5 positions in the flavone structure are key factors determining the effectiveness of these compounds as MALDI matrices.

Hydroxyflavonoid compounds were first applied as MALDI matrices by Petkovic et al. for the MALDI-MS analysis of platinum­(II) and palladium­(II) complexes.^377^ The spectra of the Pt­(II) and Pd­(II) complexes recorded in the presence of quercetin and rutin were notably simple, with Pt­(II) complexes forming [M+Na]^+^ or [M+K]^+^ ions, whereas the Pd­(II) complexes in the study generated ions via the loss of a Cl^–^ or HCl ion. Compared with traditional MALDI matrices, quercetin and rutin can be used at lower concentrations and require a reduced laser intensity.^377^ Petkovic’s team further expanded the use of flavonoid compounds as matrices for the MALDI-MS analysis of transition metal complexes. They reported that flavonoids such as apigenin, kaempferol, and luteolin were effective for analyzing Pt­(II), Pd­(II), Pt­(IV), and Ru­(III) complexes, yielding spectra with high S/N and easily interpretable peaks.^378^ In addition to metal complexes, flavonoid compounds serve as efficient matrices for analyzing LMW compounds. Wang et al. proposed hydroxyflavonoids as a new series of MALDI tissue imaging matrices.^51^ Through screening, they identified 10 natural hydroxyflavonoid compounds, including flavone and nine of its mono- or polyhydroxy derivatives (3-HF, 5-HF, 3,7-DHF, chrysin, 7,3′,4′-THF, fisetin, luteolin, quercetin, and morin), as suitable for the MALDI-FTICR-MS analysis and imaging of endogenous lipids in the mouse liver in positive ion mode. Among these compounds, the two penta-hydroxyflavonoids (quercetin and morin), showed the best performance, suggesting that the presence of hydroxyl groups at these specific positions are key factors significantly increasing the efficacy of hydroxyflavonoids as MALDI matrices (Figure 15).^51^ Wang et al. utilized MALDI-FTMS with quercetin matrix for comprehensive imaging of porcine adrenal lipids.^262^ Quercetin was used as the MALDI matrix for detection and imaging of endogenous compounds in the porcine adrenal glands by FTICR MS, with both the positive and negative ion detection.

**Figure 15:**
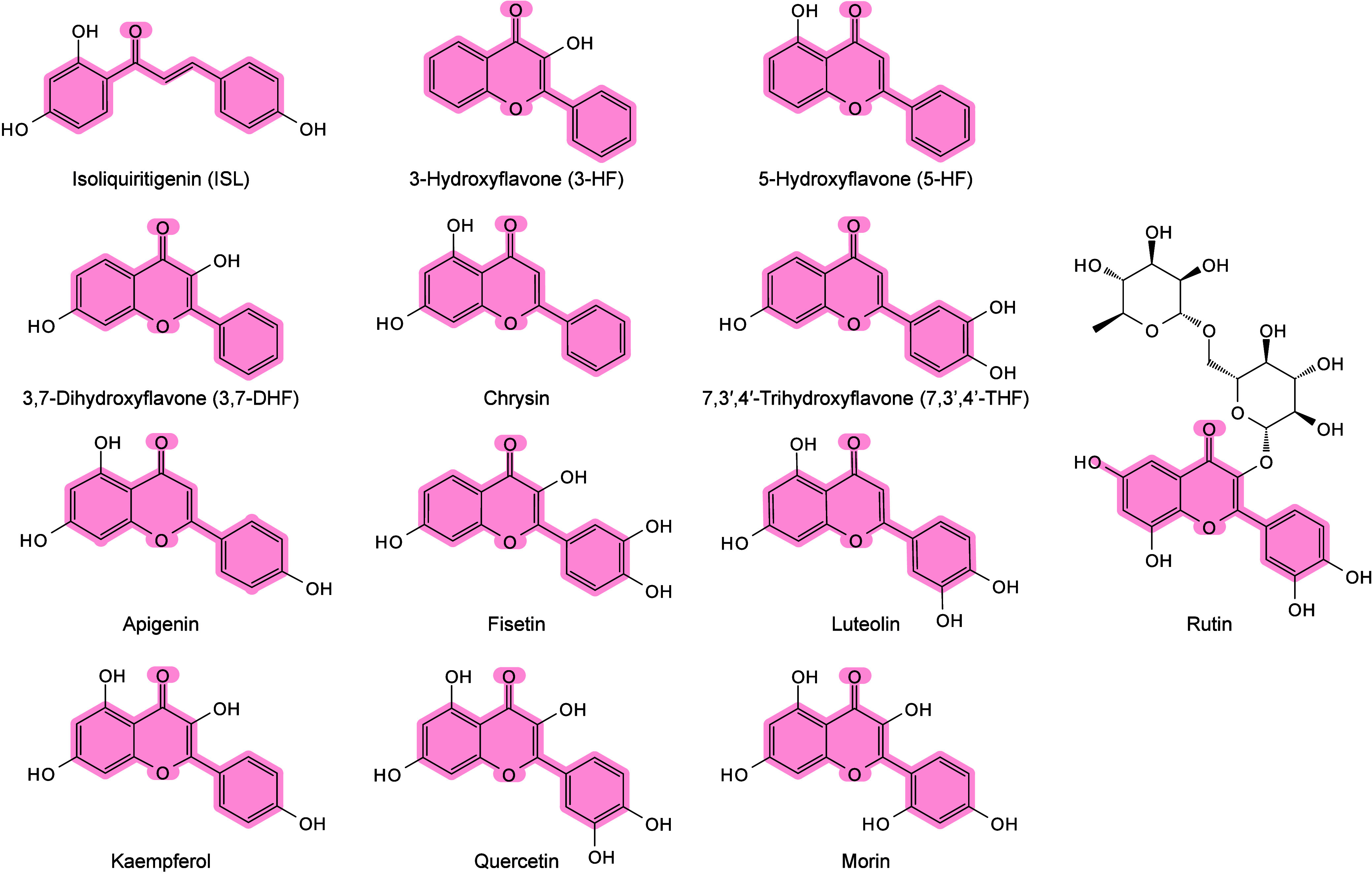
Chemical structures of flavonoid based matrices. The similar structural feature shown in these matrices is highlighted in red color.

In summary, flavonoid compounds are a novel family of MALDI matrices with the notable advantage of their nontoxic or low toxic nature. These flavonoids are derived from plants and are abundant in citrus fruits that are commonly consumed in our daily diet.^379^ Considering the safety concerns associated with selecting suitable matrices for MALDI tissue imaging, the nontoxic or low-toxicity nature of these compounds makes them particularly attractive compared with certain toxic matrices.

##### 3.1.1.7 Coumarin Based Matrices

Coumarin is a natural compound widely found in plants. It appears as a colorless crystal or powder with a pleasant vanilla-like aroma. Diverse coumarin derivatives can be synthesized by introducing various heterocyclic compounds to the benzo-α-pyrone structure, resulting in compounds such as furanocoumarins and pyranocoumarins.^380^ As a MALDI matrix, coumarin exhibits excellent absorption properties and chemical stability, effectively aiding in the ion generation of analytes and playing a crucial role in MALDI-MS analysis.^381^ Nine coumarin derivatives and analogues have been screened as widely applicable MALDI matrices for the analysis of various compounds, including coumarin, 3-hydroxycoumarin (3-HC), 7-hydroxycoumarin (7-HC), esculetin, 7-hydroxycoumarin-3-carboxylic acid (HCA), 6,7-dihydroxycoumarin-3-carboxylic acid (DCA), 7-mercapto-4-methylcoumarin (MMA), aquatic fulvic acid (AFA), and usnic acid (UA) (Figure 16).

**Figure 16:**
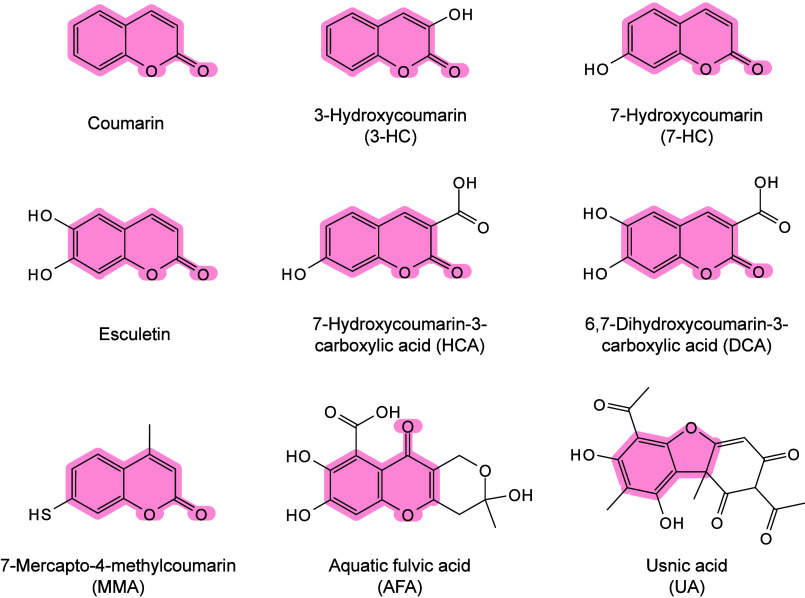
Chemical structures of coumarin and analogue based matrices. The similar structural feature shown in these matrices is highlighted in red color.

First, hydroxycoumarin compounds, which feature a coumarin ring with differing numbers of hydroxyl functional groups, serve as novel MALDI matrices for the detection of various compounds. Several hydroxycoumarins, including 3-HC, 7-HC, esculetin, HCA, and DCA, have been utilized as MALDI matrices. Specifically, 3-HC has demonstrated excellent performance in the MALDI-MS analysis of deoxyribonucleic acid (DNA) fragments. Zhang et al. reported that, compared with conventional matrices, 3-HC significantly improved the resolution, S/N, spot-to-spot, and sample-to-sample reproducibility in the analysis of synthetic oligonucleotides ranging from 3 to 70 bases.^382^ Esculetin (6,7-dihydroxycoumarin), a hydroxycoumarin with an umbelliferone structure, has a hydroxyl group substituting the hydrogen at the 6-position. Wang et al. used esculetin as a matrix for analyzing olanzapine in single hair strands via MALDI-MSI, improving the affinity, extraction efficiency, and ionization efficiency of olanzapine in hair samples.^383^ Hydrophobic compounds with hydroxyl, aldehyde, or ketone groups are often challenging to detect by MALDI-MS because of their low proton affinity and poor ionization.^384^ To address this, Wang et al. proposed coumarin as a new matrix for MALDI-FTICR-MS analysis of hydrophobic compounds. They compared the performance of five coumarin derivatives (coumarin, 7-HC, esculetin, HCA, and DCA) with that of DHB and CHCA matrices.^385^ The results indicated that DCA outperformed the other matrices in terms of sensitivity, stability, reproducibility, and accuracy and that it enabled the analysis of hydrophobic compounds in complex samples without the need for chemical derivatization.

Secondly, Feng et al. screened out MMA from the coumarin derivatives and used it as a highly efficient MALDI matrix for detecting carcinogenic arecoline alkaloids.^386^ They reported that MMA demonstrated low background interference, making it an ideal matrix for small molecule analysis. Additionally, AFA as a coumarin-similar compound is a highly functionalized, water-soluble heterogeneous mixture that requires no complex functional modification, making it a straightforward and convenient material for MALDI sample preparation. The supramolecular structure of AFA provides a large surface area, which aids in dispersing analyte molecules and prevents sample aggregation.^387^ As a primary component of humic substances, AFA contains keto acids, aromatic and aliphatic carboxyl groups, and polycarboxylated ethers and esters, endowing it with strong acidic properties and an abundance of labile protons.^388^ These characteristics make AFA an ideal candidate matrix for MALDI because it facilitates analyte protonation.^389–391^ Mugo and Bottaro first studied and reported AFA as a MALDI matrix in 2007 and demonstrated that AFA is efficient and suitable for analyzing various compounds, such as carbohydrates, cyclodextrins, and peptides,^392^ offering an attractive green alternative for MALDI applications. Finally, UA is a lichen secondary metabolite characterized by a unique dibenzofuran scaffold. Schinkovitz and Richomme reported that UA functions as a versatile MALDI matrix capable of ionizing molecules with varying structures within the range of 200 to 2,000 Da. It provides low background noise during analysis, enabling the detection of very small molecules such as yohimbine and Val-Tyr.^393^

In conclusion, coumarin derivatives are commonly used as MALDI matrices for the mass spectrometric analysis of small molecules, including DNA, hydrophobic compounds, and pharmaceuticals. These compounds possess excellent light absorption properties and high molecular ionization efficiency, contributing to the generation of clear mass spectra. Selecting an appropriate coumarin-based matrix depends on the properties of the sample and the desired analytical outcomes. Commonly used coumarin matrices include MMA, FA, usnic acid, and hydroxycoumarins. Their applications in MALDI-MS have been extensively validated, demonstrating high sensitivity and good resolution.

##### 3.1.1.8 Pyridine Based Matrices

Pyridine-based compounds typically feature an aromatic benzene ring structure, which facilitates the absorption of laser energy and stabilizes the ions generated, promoting the ionization process.^394^ Pyridine compounds often contain amino groups, nitro groups, or other groups that increase electron acceptance from the analyte, aiding in analyte ionization. Additionally, the nitrogen atom in pyridine compounds provides extra hydrogen-bonding capacity, supporting interactions with analytes and facilitating the ionization reaction.^395^ Owing to their structural characteristics, pyridine-based compounds effectively serve as MALDI matrices, and play a significant role in advancing MALDI-MS analysis.^396^ As shown in Figure 17, pyridine-based matrices can be further categorized into three main types: picolinic acid-based matrices, nicotinic acid-based matrices, and nitropyridine-based matrices.

**Figure 17:**
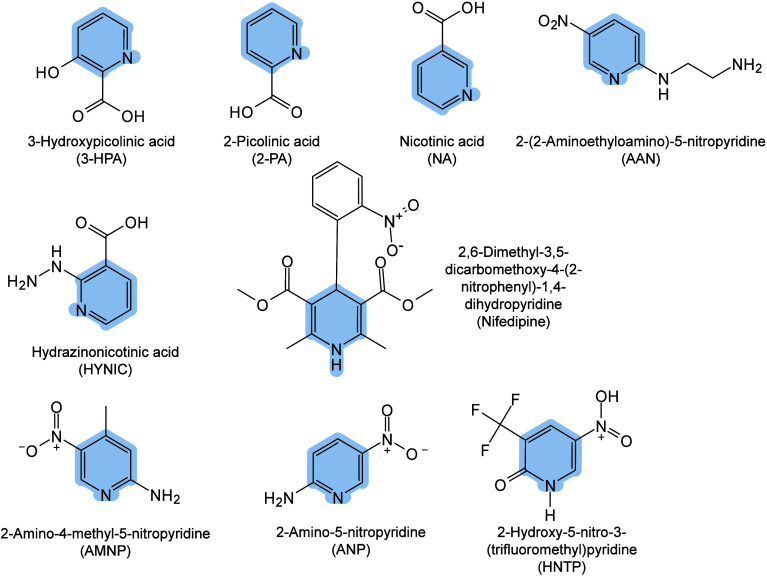
Chemical structures of pyridine based matrices. The common structural feature shown in these matrices is highlighted in blue color.

3-HPA and 2-pyridinecarboxylic acid (2-PA) are two commonly used pyridinecarboxylic acid MALDI matrices. 3-HPA is a monohydroxy pyridine in which the hydrogen at the 3-position is replaced by a hydroxyl group. As a UV-sensitive matrix, 3-HPA has improved the use of MALDI-MS for the study of oligonucleotides. Wu et al. conducted MALDI-MS analysis of underivatized single-stranded DNA oligomers and reported that 3-HPA, as an organic acid matrix, yielded stronger negative ion oligonucleotide signals than positive ion signals.^60^ Fragmentation (base loss) and adduct formation are the primary factors that lower the mass resolution of larger oligonucleotides. Wu et al. used 3-HPA as a UV-sensitive matrix for MALDI-MS analysis of mixed-base single-stranded DNA oligomers that produced quasimolecular ion signals. Their results indicated significant improvements in the mass range, S/N, and ability to analyze mixed-base oligomers compared with previously reported matrices.^397^ Additionally, Tang et al. successfully detected oligonucleotides up to 150 bases in length using 3-HPA as a matrix, showing that it is superior to DHB or FA for analyzing poly-A, poly-G, poly-C, and mixed-type oligonucleotides.^398^ Another pyridinecarboxylic acid matrix is 2-PA, which is the conjugate acid of pyridinecarboxylic acid with a carboxyl group at the 2-position. Tang et al. reported that the 2-PA matrix could be used for laser MALDI-MS analysis of nucleic acids and proteins, effectively detecting homooligonucleotides d­(G)_40_ and d­(C)_60_, as well as mixed-base oligonucleotides up to 190 bases. This marked a significant advancement in rapid DNA sequencing using laser MALDI-MS.^399^

NA is a pyridine monocarboxylic acid with a carboxyl group at the 3-position.^400^ In the 1980s, scientists such as Karas and Hillenkamp began investigating ways to enable MALDI–MS analysis of biomacromolecules.^401^ In 1988, Karas and Hillenkamp first reported the application of NA as a MALDI matrix and reported that it could convert laser energy into thermal energy, thus promoting the ionization of biomacromolecules on the sample surface and enhancing the sensitivity and feasibility of MALDI-MS analysis of biomacromolecules.^23^ The results indicated that using NA as a matrix in MALDI-MS produced abundant intact molecular ions, even for molecules with masses exceeding 10,000 Da, including protein dimers and doubly charged ions, with stability for at least approximately 1 ms. Additionally, it enabled the detection of molecular ion and doubly charged ion oligomers, thus improving molecular ions detection and molecular weight accuracy.^23^ Another nicotinic acid-based matrix is hydrazinonicotinic acid (HYNIC). Both HYNIC and NA are members of the vitamin B family, with slight structural differences but similar bioactivities.^402^ Jiao et al. reported that HYNIC serves as a novel matrix for high-sensitivity and selective MALDI-MS analysis of oligosaccharides.^403^ HYNIC exhibits significant selectivity for oligosaccharide ionization, making glycan detection in glycoproteins easier without pretreatment. Compared with the traditional DHB matrix, HYNIC offers superior crystal homogeneity and salt tolerance, while providing rich fragmentation information that aids in oligosaccharide structural analysis.

There are five types of nitropyridine used as MALDI matrices: 2-(2-aminoethyloamino)-5-nitropyridine (AAN), nifedipine, 2-amino-4-methyl-5-nitropyridine (AMNP), 2-amino-5-nitropyridine (ANP), and 2‑hydroxy-5-nitro-3-(trifluoromethyl)­pyridine (HNTP). First, AAN was identified by Lorkiewicz et al. as a matrix for negative ion mode MALDI-MS analysis of PLs. Compared with DHB, AAN demonstrates higher sensitivity for detecting PL classes in negative ion mode by generating more negative ions, which enhances sensitivity and significantly improves reproducibility while reducing acquisition time.^404^ Nifedipine, a commonly used photobase generator widely applied in polymerization, was introduced by Nguyen et al. as a novel matrix for polyphenol detection in negative ion mode MALDI-MS. Although previous studies have demonstrated that polyphenols can be directly analyzed using LDI technology to a certain extent without matrix assistance, the use of a matrix may offer some additional benefits. For instance, they reported that nifedipine was the only dihydropyridine compound capable of enhancing the detection of tea polyphenols, highlighting its importance for polyphenol analysis in negative ion mode.^405^ Another nitropyridine matrix is AMNP. In a study by Fitzgerald et al., 37 highly substituted pyrimidine, pyridine, and benzene derivatives containing basic amino groups were screened as potential matrices, and AMNP was identified as suitable for analyzing small proteins (<12,000 Da), whereas ANP proved effective for analyzing mixed-base single-stranded oligonucleotides (<20 bases) and thymine homopolymers.^406^ Although there are certain limitations for smaller protein and oligonucleotide samples, these matrices have significantly extended the applicability of MALDI to acid-sensitive species. Another nitropyridine matrix is ANP. Cheng and Chan reported that the use of ANP combined with ammonium halides (NH_4_F, NH_4_Cl, NH_4_Br, and NH_4_I) as comatrices substantially enhances the performance of MALDI-MS for oligonucleotide analysis, particularly for small mixed oligonucleotides (<20 bases) and thymine homopolymers.^407^ During oligonucleotide analysis, the ANP matrix strongly suppresses the formation of alkali metal adducts; adding ammonium halides as co-matrices significantly improves ANP matrix performance in the analysis of various oligonucleotide homopolymers, with NH_4_F offering the greatest increase among the halides.^407^ The last nitropyridine-based matrix is HNTP. Bao et al. identified HNTP as a novel matrix for enhancing the MALDI imaging of tissue metabolites.^394^ The HNTP matrix exhibits excellent properties, including strong ultraviolet absorption, micrometer-sized matrix crystals, high chemical stability, low matrix ion interference, and high ionization efficiency for metabolites. Furthermore, the use of the HNTP matrix enabled the effective detection and imaging of 152 metabolites in rat brain tissue, with their spatial distribution clearly revealing the heterogeneity of the rat brain. These results suggest that HNTP is a new and powerful MALDI matrix, that can enhance the analysis of metabolites in biological tissues using MALDI-MSI.

In summary, pyridine-based matrices exhibit unique advantages in MALDI-MS analysis, primarily because of their pyridine ring structure and the functional properties of their constituent functional groups. These matrices effectively absorb laser energy, converting it into thermal energy, which facilitates the ionization process. Additionally, their electron-accepting capacity and hydrogen-bonding interactions increase their affinity for analytes, thus improving ionization efficiency and analytical sensitivity. Various pyridine-based matrices, depending on their functional groups, are suitable for different types of analytes. For example, 3-HPA and 2-PA matrices are particularly effective for analyzing single-stranded DNA and mixed-base oligonucleotides, especially in negative ion mode. In contrast, NA and its derivatives (such as HYNIC) are more suitable for protein and oligosaccharide analysis, particularly for the ionization of larger molecules. Moreover, pyridine-based matrices are particularly efficient in negative ion mode, with nitro-substituted pyridine derivatives (such as AAN and ANP) promoting the generation of negative ions, thus increasing signal intensity. These matrices are especially effective for analyzing PLs and glycans. They also exhibit good crystal homogeneity and strong salt tolerance, which further enhance their performance in complex biological samples. Overall, pyridine-based matrices, through their ability to promote ionization, their adaptability to different functional groups, and their superiority in negative ion mode, have become indispensable tools in MALDI-MS, and can enable the analysis of a wide range of biomolecules, including nucleic acids, proteins, and carbohydrates.

##### 3.1.1.9 Benzophenone Based Matrices

Benzophenone (BPh) is the simplest benzophenone compound. It is a formaldehyde molecule in which both hydrogen atoms are replaced by phenyl groups, and it functions as a photosensitizer and plant metabolite.^408^ Benzophenone compounds contain an aromatic benzene ring structure that absorbs laser energy and transfers it to sample molecules, promoting ionization. Additionally, benzophenone compounds often contain, such as carbonyl groups, that can form hydrogen bonds or other interactions with sample molecules, thereby promoting efficient co-crystallization and the subsequent desorption and ionization of the analytes. A significant practical advantage of benzophenone compounds is their ability to yield sufficient ion intensity at comparatively low laser power, which minimizes analyte fragmentation and reduces background interference arising from excessive matrix decomposition at higher energy levels.^409–411^ These characteristics make benzophenone compounds ideal MALDI matrices, contributing significantly to enhanced sensitivity and accuracy in MALDI-MS analysis. Benzophenone matrices are classified into two main types: 3,4-diaminobenzophenone (DABP) and Michler’s ethylketone (MEK) (Figure 18).

**Figure 18:**
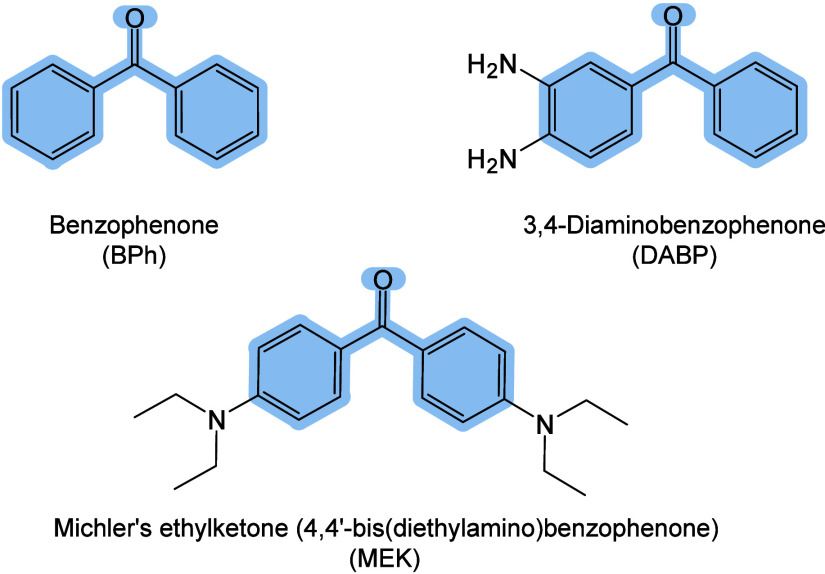
Chemical structures of benzophenone based matrices. The common structural feature shown in these matrices is highlighted in blue color.

DABP was identified by Xu et al. as a highly salt-tolerant MALDI-MS matrix. It enables the detection of insulin ion signals in the presence of 2 M guanidine hydrochloride and 1.5 M urea, effectively suppressing peptide cation adduction in solutions with high concentrations of metal ions.^412^ Additionally, DABP has proven to be an excellent matrix for detecting intact oligonucleotides without the need for additional dopants or co-matrices, offering significantly improved salt tolerance, reduced fragmentation, fewer alkali metal adducts, and notably enhanced detection sensitivity and mass resolution.^410^ Another benzophenone derivative used as a matrix is MEK. MEK is a commonly used intermediate in dye and pigment synthesis.^413^ Shi et al. proposed MEK as a novel negative ion matrix for enhancing lipid MALDI tissue imaging.^414^ In MALDI-MS, MEK is characterized by strong UV absorption, micrometer-scale crystal size, uniform coating, ultra-high vacuum chemical stability, low matrix-related ion interference, and high ionization efficiency. It outperforms traditional matrices such as 2-MBT and DHA in lipid detection and imaging in rat brain and yew tissue sections, expanding the MALDI matrix library and improving lipid detection and imaging coverage in plant and animal tissues.

In summary, benzophenone compounds stand out as superior MALDI matrices with exceptional absorption characteristics, high molecular ionization efficiency, and remarkable molecular recognition capabilities. As exemplary matrix materials, they significantly contribute to enhancing the sensitivity and precision of MALDI-MS analysis.

##### 3.1.1.10 Nitrobenzene Based Matrices

The main types of nitrobenzene compounds used as MALDI matrices are 4-hydroxy-3-nitrobenzonitrile (HNBN), *p*-nitroaniline (PNA), 2-amino-3-nitrophenol (ANP), 4-nitrocatechol (4-NC), and 2‑nitrophloroglucinol (2-NPG) (Figure 19).

**Figure 19:**
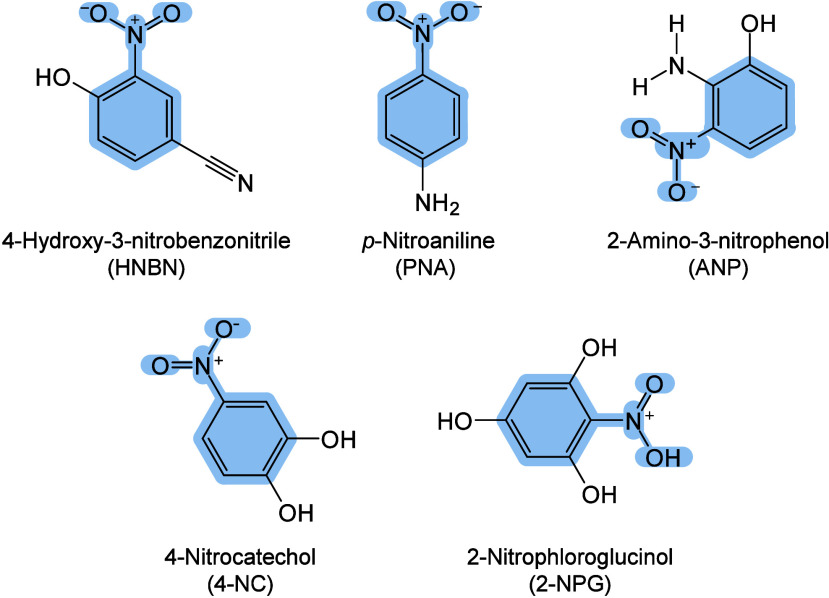
Chemical structures of nitrobenzene based matrices. The common structural feature shown in these matrices is highlighted in blue color.

HNBN was identified by Gu et al. as a versatile MALDI matrix suitable for analyzing small organic molecules, peptides, and proteins. It exhibits strong UV absorption properties, providing a clean background in the low mass range.^56^ The second nitrobenzene compound matrix, PNA also known as 4-nitroaniline, contains a nitro group at the 4-position on the aniline (ANI) ring. Its benzene ring structure and nitro group enable effective UV absorption, facilitating photodesorption, and it demonstrates excellent vapor phase absorption characteristics. This promotes effective co-desorption of the matrix and analyte, followed by gas-phase interactions (*e.g.*, proton transfer) in the plume, which enhances the reproducibility and precision of the analysis.^415,416^ Gimon et al. used PNA as a matrix in MALDI-MS and observed superior ion abundance and reproducibility, making it suitable for detecting paclitaxel and related steroids.^417^ Additionally, Zhang et al., in their screening of novel small-molecule matrices, reported that PNA exhibited excellent performance for the simultaneous MALDI-MS imaging of various lipids and plant hormones. Particularly in positive ion mode, it showed high sensitivity for detecting lysophosphatidylcholines (LPCs), phosphatidylcholines (PCs), and TAGs, with strong UV absorption and uniform crystallization. Compared with traditional matrices, PNA provides higher ionization efficiency sensitivity, and clearer imaging in dual-polarity mode.^418^ The third nitrobenzene compound matrix, ANP, was explored by Chen et al. as a matrix for visible laser (VIS)-MALDI-MS.^419^ These authors reported that the mass spectra generated under visible and UV light shared similar characteristics and sensitivities for peptides, polymers, and small proteins. With a lower optical absorption coefficient at 532 nm, it allows greater optical penetration depth, leading to higher sample consumption per laser shot than with UV-MALDI. However, in applications requiring deeper laser penetration, VIS-MALDI using ANP as a matrix could serve as a complementary technique to traditional UV-MALDI.^419^ The fourth nitrobenzene-based compound matrix is 4-NC. 4-NC is a type of catechol in which a nitro group is substituted at the 4-position on the benzene-1,2-diol ring. Xu et al. utilized 4-NC as a novel matrix for the *in situ* detection and imaging of LMW compounds in biological tissues using MALDI-MSI.^420^ The results indicated that 4-NC exhibits strong ultraviolet absorption, uniform crystal formation, excellent chemical stability, and minimal matrix-related background peaks. Additionally, the use of 4-NC allowed clear visualization of the spatial distribution of LMW compounds in rat brain and germinating *Taxus* seed tissue sections. In conclusion, using 4-NC in MALDI-MSI enhances the imaging of LMW compounds in tissue sections, showing great potential as a matrix for the MALDI tissue imaging of LMW compounds. The last nitrobenzene-type compound matrix is 2-NPG. McDonald et al. conducted a quantitative MALDI-MS and imaging study of the fungicide pyrimethanil (PYM) in strawberries using 2-NPG as an effective matrix.^421^ In this study, MALDI-MS and imaging techniques were used to explore the quantitative analysis of the fungicide PYM in strawberries using 2-NPG as the matrix. Compared with conventional matrices such as CHCA and DHB, 2-NPG exhibited superior sensitivity and accuracy in analyzing PYM. The novel use of 2-NPG as a matrix in MALDI-MS quantification and imaging can be applied to other analytes.

Nitrobenzene compounds function as matrix molecules in MALDI-MS, playing a pivotal role in absorbing laser energy and mediating the co-desorption and ionization of analytes. Their selection is guided by criteria such as absorption efficiency, compatibility, and ion signal intensity. Owing to their exceptional performance in MALDI-MS, nitrobenzene compounds are extensively employed in the analysis of biomolecules such as polymers, proteins, peptides, lipids, and phytohormones. Overall, nitrobenzene compounds constitute an indispensable component of MALDI matrices in MS analysis.

##### 3.1.1.11 Naphthalene Based Matrices

Naphthalene, the simplest fused aromatic hydrocarbon, consists of two benzene rings sharing two adjacent carbon atoms. It is widely used as a raw material for the production of dyes, resins, and solvents, as well as a moth repellent.^422^ Naphthalene compounds can serve as MALDI matrices because of their structural characteristics. First, naphthalene molecules have a planar polycyclic aromatic hydrocarbon structure, which endows them with high UV absorption capability. Second, their structure is relatively stable, allowing energy to be transferred efficiently to sample molecules after absorption.^415^ Additionally, interactions such as hydrogen bonding may occur between naphthalene compounds and sample molecules, promoting ionization.^423^ Naphthalene-based matrices are classified into two main types: proton sponges and naphthyl salts.

A classic MALDI-MS matrix for small molecule analysis is 1,8-bis­(dimethylamino)­naphthalene (DMAN), which belongs to a class of compounds known as proton sponges, named for their ability to absorb any available protons.^52^ The defining characteristics of proton sponges include the destabilizing effect between two neutrally substituted diamine lone electron pairs and the hydrophobic shielding of these basic centers. This hydrophobic shielding and the N–H···N hydrogen bonding at basic centers results in very low rates of protonation and deprotonation, although this does not affect their high thermodynamic basicity.^423,424^ Examples of proton sponges used as MALDI matrices include DMAN, 1,8-bis­(tetramethylguanidino)-naphthalene (TMGN), *N*-phenyl-2-naphthylamine (P2NA), 4-maleic anhydride proton sponge (MAPS), 1,8-bis­(tris-pyrrolidinyl-phosphazenyl)­naphthalene (TPPN), and 1,14-diaza[5]­helicene (Figure 20).

**Figure 20:**
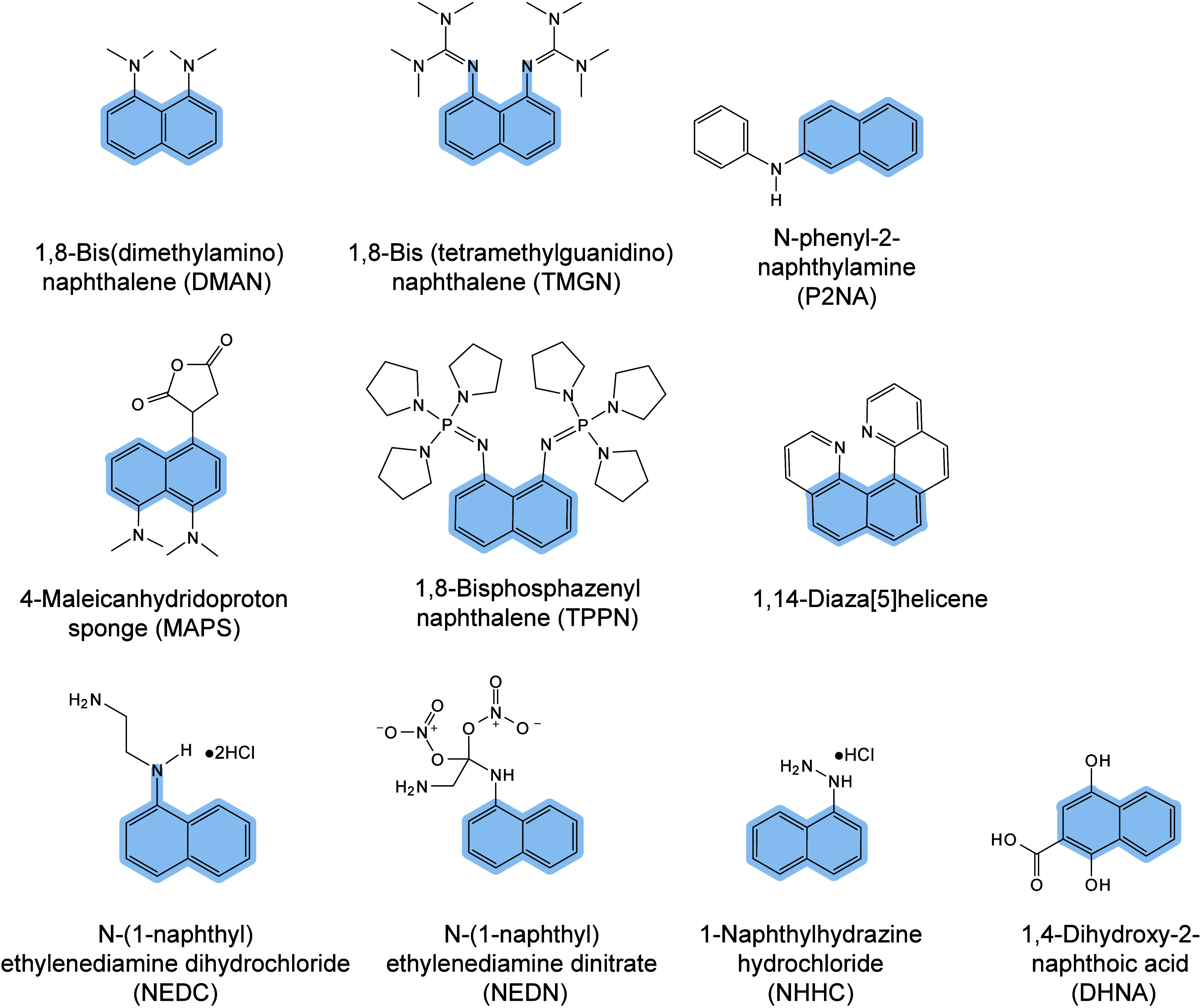
Chemical structures of naphthalene based matrices. The similar structural feature shown in these matrices is highlighted in blue color.

DMAN is a highly basic organic compound. Shroff and Svatos identified DMAN as a novel superbasic matrix for MALDI-MS analysis of fatty acids (FAs). Owing to its high basicity, DMAN can absorb any available proton, including the weakly acidic protons in FAs, to form deprotonated anions. This property enables DMAN to completely suppress matrix-related ions across the low mass range (<1,000 Da), making it an excellent matrix.^425^ Unfortunately, DMAN is highly unstable under vacuum, rendering it unsuitable for MALDI-MS experiments.^363,426^ To address this limitation, researchers have explored alternative matrices to replace DMAN, including structural modifications of DMAN or investigations of other types of organic superbases. MALDI-MSI requires long-term detection under high-vacuum conditions, which limits the application of many matrices that lack vacuum stability. Yu et al. proposed a novel approach that utilizes conjugated polymer anchors to increase the vacuum stability of matrices through ionic bonds, thereby improving MALDI-MSI performance.^427^ Unlike strong covalent bonds, weaker ionic bonds facilitate bond cleavage under laser irradiation while effectively preventing matrix volatilization in a vacuum environment. The results demonstrate that DMAN, which is unstable in the MALDI source, can be effectively used for MSI when protected by polyacrylic acid. This study revealed that conjugated polymer anchors neither introduce additional ion peaks nor affect signal intensity and they maintain good quantitative characteristics. This design significantly enhances the vacuum stability of volatile matrices and improves *in situ* MALDI-MS imaging capabilities for mouse brain and liver cancers.

TMGN is a novel super-basic organic base first synthesized by Raab et al. in 2002.^428^ TMGN has a high basicity constant and exhibits strong UV absorption at 330–350 nm, which is essential for the matrix-assisted desorption and deprotonation of acidic analytes. TMGN can serve as a matrix for the MALDI-MS analysis of acidic perfluorinated compounds (PFCs) or other acidic small molecules. Using TMGN as a matrix, Cao et al. were the first to employ MALDI-MS for the quantitative detection of trace PFCs in environmental water samples.^429^ The results showed that TMGN efficiently deprotonated these PFC ions without interference from other matrix ions.

P2NA was identified by Liu et al. as a novel matrix suitable for MALDI-MS small molecule analysis and *in situ* imaging.^415^ P2NA exhibits strong UV absorption, low background interference, and high salt tolerance, demonstrating excellent performance in the analysis of various small-molecule metabolites, including free FAs, AAs, peptides, antioxidants, and PLs. Imaging experiments further revealed minimal metabolic changes over 48 hours, indicating that P2NA has low volatility and high chemical stability.

Modifying DMAN by adding a maleic anhydride molecule to its para position yielded 3-(4,5-bis­(dimethylamino)­naphthalen-1-yl)­furan-2,5-dione, known as a 4-maleic anhydride proton sponge or “MAPS”.^430^ Giampà et al. proposed MAPS as a novel MALDI matrix suitable for observing small molecules in brain tumors.^431^ By chemically modifying the DMAN matrix with active maleic anhydride, MAPS enables MALDI-MSI analysis of small molecules in biological tissue samples, such as tumors, without the need for further derivatization steps. Additionally, MAPS enhances the proton sponge’s vacuum stability, allowing for small molecule localization using conventional MALDI-MS. The electrophilic maleic anhydride molecule also provides new synthetic pathways for MALDI-MSI of small molecules and inorganic anions, leading to a series of novel vacuum-stable proton sponge-based molecules.^431^

1,8-Bisphosphazenylnaphthalene (PN) was recently successfully synthesized and characterized. This super-basic proton sponge matrix, with a PN core and various P-amino and -alkyl substituents, exhibits remarkable basicity, making it a promising candidate as a negative ion MALDI matrix for deprotonating other difficult-to-ionize small compounds. Calvano et al. reported that these super-basic alkyl-substituted bisphosphazenylnaphthalene proton sponges represent a novel class of deprotonating matrices. Among them, TPPN demonstrated optimal performance in negative ion MALDI-MS analysis for LMW, hard-to-ionize analytes, enabling the direct detection of intact cholesterol without derivatization steps.^432^ This study is the first to demonstrate the practicality of novel super-basic proton sponges in MALDI-MS for detecting hard-to-ionize compounds such as sterols, steroids, fatty alcohols, and saccharides.

As in DMAN, the conformational strain in 1,14-diaza[5]­helicene brings the lone pairs on the nitrogen atoms into close proximity. However, unlike DMAN, where protonation (and deprotonation of DMANH^+^) is slow because of hydrophobic shielding, 1,14-diaza[5]­helicene is a kinetically active proton sponge because of the rapid proton exchange between the donor and the diaza[5]­helicene base.^432^ Napagoda et al. identified 1,14-diaza[5]­helicene, a kinetically active superbase azapentalene superbase, as a suitable MALDI matrix for acidic analytes.^433^ The results indicate that 1,14-diaza[5]­helicene has potential for use as a MALDI matrix for analyzing FAs and organic acids across a wide range of samples, making it well-suited for high-throughput metabolomics analysis.

*N*-(1-Naphthyl)­ethylenediamine dihydrochloride (NEDC) and its derivatives are efficient matrices for small molecule analysis.^434,435^ Both NEDC and *N*-(1-naphthyl) ethylenediamine dinitrate (NEDN) contain a 1-naphthyl group, although they differ slightly in how the 1-naphthyl group is combined with the ethylenediamine molecule and corresponding salts. NEDC is formed by connecting the 1-naphthyl group to an ethylenediamine molecule, which is then combined with hydrochloric acid to form a salt, whereas NEDN connects the 1-naphthyl group to ethylenediamine and forms a salt with two nitrate ions.^436^ Naphthyl-based salt matrices can be categorized into NEDC, NEDN, and 1-naphthylhydrazine hydrochloride (NHHC) (Figure 20).

NEDC consists of a 1-naphthyl residue, one molecule of ethylenediamine, and two molecules of hydrochloric acid. NEDC is an effective MALDI-MS matrix for analyzing glucose levels in rat brain microdialysate^435^ and has also been used for successful identification of metal ions in real samples.^437^ This suggests its potential for broad applications in the biological research of metals and organic compounds. NEDN, a derivative of NEDC. It contains a naphthalene core (a strong UV-absorbing chromophore) and displays a broad absorption band centered at 324 nm. Chen et al. identified NEDN as a novel matrix for negative ion MALDI-MS analysis of small molecules (*m*/*z* < 1,000), with a well-designed UV-absorbing chromophore, hydrogen bonding, and nitrate donor characteristics, making it suitable for the analysis of oligosaccharides, peptides, and metabolites.^434^ Compared with traditional matrices, NEDN increases detection sensitivity, reduces matrix-related fragmentation and cluster ions, and produces spectra in the low-mass range (*m*/*z* < 200) composed solely of nitrate and nitrate cluster ions signals, making it particularly useful for the structural identification of oligosaccharides via PSD MALDI-MS. Another naphthyl-based salt matrix is NHHC. Its structure includes a naphthyl group and hydrazine, and it forms a hydrochloride salt. He et al. found NHHC to be a promising new MALDI-MS matrix for quantifying glucose and homogentisic acid in real samples. NHHC is characterized by salt tolerance, low cost, adequate UV absorption, and minimal interference in the low-mass region, providing significant advantages for enhanced MALDI performance and offering a promising method for rapid, high-throughput analysis of small molecules in complex samples.^438^

In addition, 1,4-dihydroxy-2-naphthoic acid (DHNA) is also a naphthalene-based matrix. DHNA is a naphthoic acid that is 2-naphthoic acid substituted by hydroxy groups at positions 1 and 4. It is functionally related to a 2-naphthoic acid. A previous study has demonstrated that Tang et al. employed DHNA matrix for polymer analysis by MALDI-MS.^439^ However, its application in *in situ* detection and imaging of lipids remains unexplored. For this purpose, Liu et al. successfully screened and optimized DHNA as a MALDI matrix for *in situ* detection and imaging of lipids in rat brain tissue and *Lindera aggregate* (Sims) Kosterm. tuber-root tissues by MALDI MS in the positive-ion mode.^440^ In this study, they reported DHNA can improve the detection and/or imaging performance of lipids in biological samples by MALDI-TOF MS. DHNA has several advantages, including strong ultraviolet-visible absorption, fewer matrix-related ion signals, uniform matrix deposition, high vacuum stability, and superior salt tolerance. Furthermore, DHNA was used for *in situ* detection and imaging of lipids in rat brain and *Lindera aggregate* (Sims) Kosterm. tuber root tissue sections, where 174 and 102 lipid ion signals were successfully detected and imaged using MALDI-MSI, respectively. Overall, their study demonstrates that DHNA is a potent MALDI matrix, exhibiting excellent performance in detecting and imaging lipids in biological tissues.

In summary, naphthalene-based compounds serve essential functions as MALDI matrices in MALDI-MS analysis, with distinctive features. Naphthalene-based matrices can be broadly categorized into two types: super-basic proton sponge matrices and naphthyl salt matrices. The super-basic proton sponge matrices in turn comprise several main types. Classic organic proton sponges, such as DMAN, are characterized by strong electrostatic repulsion between the lone pairs of electrons at their basic centers, which also feature hydrophobic shielding and N–H^+^–N hydrogen bonding upon single protonation. Structurally, naphthyl salt matrices are characterized by the inclusion of a 1-naphthyl group, which enhances the aromatic nature of the molecule. However, it should be noted that these "proton sponge matrices" may exhibit poor stability under high vacuum conditions (*i.e.*, high sublimation rates), leading to changes in the matrix-to-analyte ratio and contamination of the mass spectrometer’s ion optics components.^441^ Therefore, it is necessary to carefully select proton sponge as the MALDI matrix. This is particularly beneficial for the analysis of organic compounds, as it provides stable protonated or deprotonated ions, facilitating the formation of molecular and fragment ions. The ethylenediamine molecule contains two amino groups that can form hydrogen bonds or salt bridges with protons or other ions in the sample, increasing the efficiency of protonation or deprotonation. Selecting different types of naphthalene-based compounds enables the effective analysis and detection of various sample properties, offering crucial analytical tools and technical support for fields such as biomedicine and drug development.

##### 3.1.1.12 Pyridazine Based Matrices

Pyridazine compounds exhibit broad-spectrum antibacterial, insecticidal, herbicidal, and antiviral activity. By introducing various active groups (such as amides, heterocycles, or methoxyacrylate), pyridine-based compounds will have the potential to become candidates for MALDI matrices. Pyridazine-based matrix compounds are categorized into four main types: 3-aminophthalhydrazide (3-APH), hydralazine (HZN), 4-aminoisoquinoline-3-carboxamide (4-AC), and 4-hydrazinoquinoline (4-HQ) (Figure 21).

**Figure 21:**
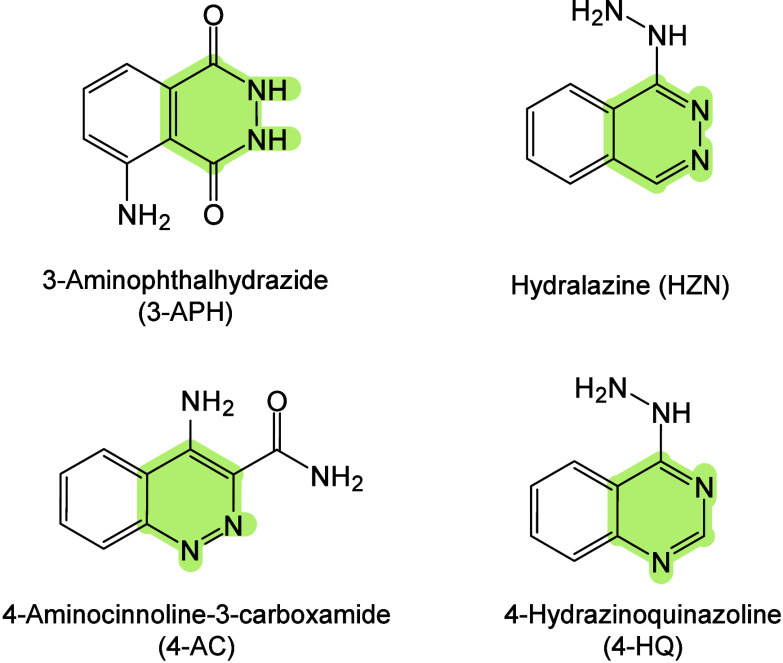
Chemical structures of pyridazine based matrices. The similar structural feature shown in these matrices is highlighted in green color.

Li et al. demonstrated that 3-APH (also known as luminol) serves as an excellent matrix for dual-polarity MALDI-MSI, enabling the detection of metabolites in mouse brain tissue.^442^ They evaluated five structurally related compounds, including phthalhydrazide, 3-APH and its sodium salt, 4-aminophthalhydrazide (4-APH), and 3-nitrophthalhydrazide (3-NPH), and found that these compounds have potential for use as matrices to detect metabolites in mouse brain tissue via MALDI-MSI. Compared with other similar compounds, 3-APH exhibited superior performance under dual-polarity conditions, with higher sensitivity, broader molecular coverage, and lower background noise, making it particularly effective for detecting endogenous metabolites.

HZN has been identified as a versatile, universal matrix for high molecular coverage and dual-polarity MALDI-MSI. Tang et al. prepared HZN by alkalizing HZN-HCl with ammonia and incorporated NH_4_OH or TFA to enhance its imaging performance for biologically relevant compounds in both negative and positive ion modes.^443^ Compared with traditional matrices (DHB, CHCA, and 9-AA), the HZN matrix demonstrates higher sensitivity, broader molecular coverage, and improved signal intensity. It has been successfully applied to observe tissue-specific distributions and changes in small molecules, lipids, and proteins in kidney and liver sections of diabetic mice.

4-AC is an organic compound containing a cinnoline ring. Its structural features include amino and carboxamide groups attached to the cinnoline ring at positions 4 and 3, respectively. Chen et al. used 4-AC as a matrix in MALDI-MS imaging to reveal metabolic alterations in a transgenic mouse model of Alzheimer’s disease (AD).^444^ The results demonstrated that 4-AC exhibits excellent performance in terms of UV absorption, ion yield, background interference, and vacuum stability. Additionally, this matrix can detect a wide range of molecules in both positive and negative ion modes, including AAs, FAs, nucleotides, and various lipids, making it an ideal choice for assessing metabolic changes in the brain and serum of AD transgenic mouse models.

4-HQ has emerged as a promising reactive matrix for glycan analysis. The inherently low ionization efficiency of glycans limits the sensitivity of direct MALDI-MS analysis. To address this challenge, Ling et al. employed 4-HQ as a reactive matrix and successfully achieved the rapid and sensitive analysis of both neutral and glycosylated glycans.^445^ Compared with traditional matrices, 4-HQ reduced the detection limits of maltoheptaose and A3 glycans by 100-fold and 20-fold, respectively. Additionally, 4-HQ formed uniform crystals, resulting in excellent experimental reproducibility. Notably, 4-HQ reacts with the reducing ends of glycans through its hydrazine group, significantly increasing the S/N and signal intensity during glycan analysis. This dual functionality enables 4-HQ to act not only as an efficient matrix for neutral and glycosylated glycans but also as a reactive agent to further improve analytical sensitivity. Its ease of use, combined with its ability to detect both neutral and glycosylated glycans simultaneously, underscores the substantial potential of 4-HQ in the analysis of glycans from biological samples. In particular, 4-HQ has demonstrated remarkable applicability in the analysis of glycans released from glycoproteins and human serum, highlighting its broad utility in glycan study.

In summary, owing to their distinctive properties, pyridazine compounds are important matrices in MALDI. Owing to the unique structural features of these compounds, they are suitable as dual-polarity MALDI matrices. Pyridazine compounds typically bear functional groups such as hydroxyl, carboxyl, and amino moieties, enabling efficient protonation or deprotonation reactions and thus facilitating adaptation to dual-polarity mode detection. These compounds effectively absorb laser energy, donate or accept protons, and minimize background noise, thereby increasing mass spectrometric signal intensity and resolution. In the biological, medical, and materials science fields, pyridazine compounds are extensively utilized as MALDI matrices.

##### 3.1.1.13 Indole Based Matrices

Indole is an electron-rich compound and is widely distributed in biological systems such as proteins (tryptophan side chains) and alkaloids. Structurally, an indole consists of a pyrrole fused with a benzene ring, also known as benzopyrrole. Owing to its two different fusion modes, it exists in two forms, indole and isoindole. Indole and its homologs and derivatives are abundant in nature and are predominantly found in natural floral oils from jasmine, bitter orange, narcissus, and basil. As a MALDI matrix, indole compounds exhibit strong UV laser absorption properties that facilitate the absorption and ionization of target molecules, thereby increasing the intensity and quality of mass spectrometric signals and improving analytical sensitivity and accuracy.^446^ Indole matrices are chemically stable, easy to prepare, and cost-effective, making them widely used in the mass spectrometric analysis of biomacromolecules such as proteins and peptides.^447–449^ Indole-based MALDI matrices can be broadly classified into three types: indole acids, pyridine indoles, and nitro indoles, which are extensively applied in the analysis and detection of biomolecules such as proteins, lipids, and nucleotides (Figure 22).

**Figure 22:**
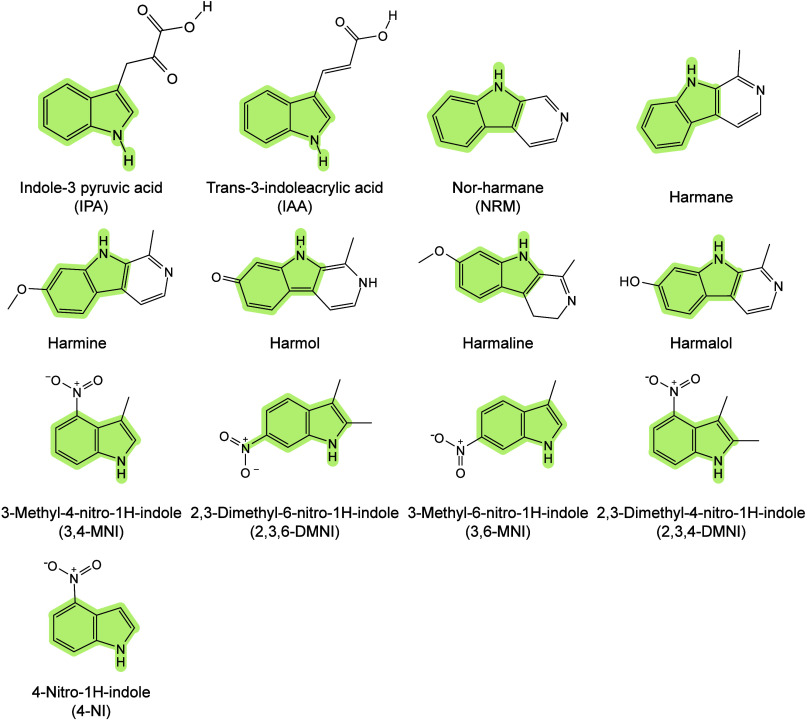
Chemical structures of indole based matrices. The common structural feature shown in these matrices is highlighted in green color.

Indole acids are a notable category of matrix compounds. Indole-3-pyruvic acid (IPA) enables high-sensitivity detection of peptides, cytochrome c, and lysozyme. IPA has been identified as an effective MALDI matrix, particularly suitable for the analysis of LMW proteins and peptides. However, compared to conventional matrices such as DHB, IPA exhibits lower signal stability and reproducibility when analyzing samples containing a higher level of impurities. This may stem from its crystal structure or chemical properties, which make it susceptible to impurities, leading to a deterioration in signal quality.^450^ *trans*-3-Indoleacrylic acid (IAA) is another indole acid used as a matrix for the rapid screening of anabolic steroids in MALDI-MS. In studies by Galesio et al., nine organic matrices were evaluated, and IAA was found to be the most suitable for steroid identification, providing good signal and stability. Using this matrix, MALDI-MS offers a robust method for the rapid screening of steroids with a sensitivity comparable to that of traditional gas chromatography (GC)-MS techniques commonly employed for this purpose.^357^

β-Carboline alkaloids were first proposed as UV-MALDI matrices for carbohydrate analysis in 1997. Nonami et al. revealed that β-carboline alkaloids serve as effective MALDI-MS matrices for the detection of proteins and sulfated oligosaccharides. They successfully employed six types of β-carboline alkaloids, *nor*-harmane (9*H*-pyrido­[3,4-*b*]­indole, NRM), harmane (1-methyl-9*H*-pyrido­[3,4-*b*]­indole), harmine (7-methoxy-1-methyl-9*H*-pyrido­[3,4-*b*]­indole), harmol (1-methyl-9*H*-pyrido­[3,4-*b*]­indol-7-ol), harmaline (3,4-dihydro-7-methoxy-1-methyl-9*H*-pyrido­[3,4-*b*]­indole), and harmalol (3,4-dihydro-1-methyl-9*H*-pyrido­[3,4-*b*]­indol-7-ol), for MALDI-MS analysis.^451^ The results demonstrated that β-carboline alkaloids are highly effective matrices for protein analysis, showing performance comparable or superior to that of traditional matrices. Among the β-carboline alkaloid family, harmine and NRM are the most frequently utilized, enabling structural and functional analyses of biomacromolecules such as proteins, peptides, and nucleic acids. Harmine is a plant alkaloid with a polycyclic aromatic structure that contains both pyrrole and indole rings. Yang et al. employed MALDI-MS to detect anionic adducts (Cl^–^, HSO_4_^–^ and Br^–^) of oligosaccharides and reported that harmine, as a β-carboline alkaloid, performs effectively as a MALDI matrix for this purpose.^452^ Additionally, NRM is an alkaloid with a structure similar to that of harmine, featuring both pyrrole and aromatic rings. Monge et al. used nuclear magnetic resonance spectroscopy and UV-MALDI-MS to analyze the structural characteristics of naturally high-methoxylated pectin and compared DHB and NRM as UV-MALDI matrices.^453^ While NRM has not been previously used as a UV-MALDI matrix for pectin analysis, its use can eliminate the need for pretreatment steps such as enzymatic digestion or acid hydrolysis and avoid salt addition, simplifying and expediting the analysis. Fukuyama et al. applied NRM as a matrix for MALDI-MS in the measurement of xylan, extending its utility to oligosaccharide analysis.^454^ Moreover, Alison et al. reported that the NRM matrix enhanced the ability to detect endotoxins via MALDI-MS, enabling the simultaneous analysis of pathogens, hosts, and vector systems. Their findings indicate that NRM is a powerful and versatile matrix that is widely applicable in bacterial, vector, and host lipid analyses, achieving picogram-level detection of monophosphoryl lipid A (MPLA) and enabling MPLA analysis in primary extracts from both *in vitro* and *in vivo* infection model systems.^455^

Pyridoindole compounds have also been applied as MALDI matrices. Nonami et al. evaluated pyridoindoles, pyridylindoles, and pyridylpyridoindoles as MALDI-MS matrices, selecting compounds containing both indole N-H and pyridine nitrogen to study their combined effects on the MALDI D/I process.^396^ Their research revealed that only aromatic polycyclic structures possessing both an acidic indole N-H group and a basic pyridine group function effectively as protein matrices. These compounds not only serve as matrices for analyzing specific carbohydrates and synthetic polymers but also are suitable for conducting PSD analysis of cyclodextrins in both positive and negative ion modes. Furthermore, the simultaneous presence of proton-donating (indole N-H) and proton-accepting (pyridine ring) functional groups plays a crucial role, with the position and relative orientation of these groups being essential to the performance of the MALDI matrix.

Nitroindole (NI) compounds are widely used as matrices in MALDI-MS detection and imaging. Liang et al. synthesized five NI derivatives, 3-methyl-4-nitro-1*H*-indole (3,4-MNI), 3-methyl-6-nitro-1*H*-indole (3,6-MNI), 2,3-dimethyl-4-nitro-1*H*-indole (2,3,4-DMNI), 2,3-dimethyl-6-nitro-1*H*-indole (2,3,6-DMNI), and 4-nitro-1*H*-indole (4-NI), and demonstrated their utility as MALDI matrices for a variety of analytes in both positive and negative ion modes. These matrices have potential applications in the detection of lipids, peptides, proteins, glycans, and environmental pollutants such as perfluorooctane sulfonate (PFOS).^447^ Among the NI matrices, 3,4-MNI exhibited the best performance, reducing ion suppression and enhancing detection sensitivity for complex mixtures, whereas 2,3,6-DMNI was identified as the optimal matrix for imaging blueberry tissue. NI matrices are a unique family of MALDI matrices with broad applications in both qualitative and quantitative analyses. They effectively detect metabolites, lipids, peptides, proteins, glycans, oligonucleotides, polymers, and environmental pollutants such as PFOS in both positive and negative ion modes.^447^

By meticulously selecting indole compounds as matrices, the experimental conditions for MALDI-MS analysis can be finely optimized, thereby significantly increasing both the success rate and the reliability of the analytical outcomes. In summary, when employed as MALDI matrices, indole compounds demonstrate indispensable characteristics and functions, such as efficient energy absorption, effective ion generation, and robust analyte stabilization. These attributes collectively contribute to the substantial advancement of MALDI-MS analysis. Looking ahead, continued exploration and refinement of indole-based matrices are poised to unlock new frontiers in MALDI-MS, potentially leading to breakthroughs in sample compatibility, sensitivity, and resolution across a wide range of applications.

##### 3.1.1.14 Other Matrices in Small Organic Molecules Matrices

###### 3.1.1.14.1 Other SOM Matrices Used for Positive Ion Mode Detection

The relentless development and continuous optimization of SOM matrices customized for positive ion mode have brought about a paradigm shift in MALDI-MS. This has empowered the technique to conduct both robust qualitative and quantitative analyses across a wide array of biomolecules. Beyond the aforementioned and well-defined SOM matrices, there exists many other SOM matrices whose classification remains ambiguous due to their distinctive structures. Through systematic screening, these matrices have proven to be of great significance in MALDI-MS analysis. Prominent members of this category encompass 4-hydroxy-3-methoxyphenylpyruvic acid (HMPPA), osazones, 2-amino-4,5-diphenylfuran-3-carboxylic acid (ADFA), 2,3-dicyanohydroquinone (DCH), 5-amino-1-naphthol (5,1-ANL), lumazine, and olanzapine (OLZ) (Figure 23). Each of these matrices, characterized by their unique structural motifs, including phenolic hydroxyls, naphthalene rings, and conjugated systems, is capable of surmounting specific analytical challenges. Consequently, they have also broadened the scope of MALDI-MS applications in proteomics, glycomics, lipidomics, and pharmaceutical analysis, heralding new frontiers in the field.

**Figure 23:**
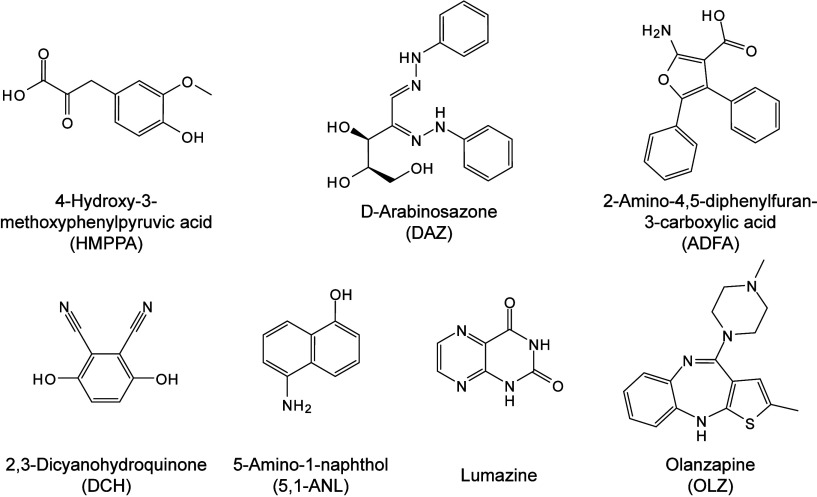
Chemical structures of other SOM matrices used for MALDI-MS positive ion mode detection.

HMPPA (also known as vanilpyruvic acid) is a 2-oxo monocarboxylic acid that is classified as a 3,4-dihydroxyphenylpyruvic acid, with the hydroxyl group at the 3-position replaced by a methoxy group. Bai et al. identified HMPPA as an effective matrix for the MALDI-MS analysis of proteins and peptides, demonstrating both impurity sensitivity and robustness, with proven efficacy in the analysis of proteins in milk.^450^

Osazones are the products of reactions between carbohydrates and aromatic hydrazines; they were first synthesized by Fischer in 1884, and are among the best examples of crystallinity in organic chemistry. The most effective osazone used as a MALDI matrix is d-arabinosazone. d-Arabinosazone is a carbohydrate compound with strong UV absorption properties that efficiently absorbs laser energy and converts it into ionizing capacity, enhancing the stability and sensitivity of MS signals. Its structure contains hydroxyl groups that can donate protons, thereby improving analyte sensitivity and selectivity.^456^ Chen et al. utilized osazones as matrices for the MALDI-MS analysis of carbohydrates and reported that osazones prepared from d- or l-arabinose and phenylhydrazine exhibited optimal overall performance for oligosaccharide analysis. The microcrystalline nature of osazones facilitates the formation of highly uniform layers, and the addition of small amounts of sodium ions to the osazone matrix increases detection limits, while higher sodium concentrations facilitate specific carbohydrate fragmentation.^457^

ADFA was first identified as a high-performance organic matrix for MALDI-MS by Abdelhamid and colleagues.^339^ Structurely, ADFA features a furan core functionalized with a carboxylic acid group (−COOH), an amino group (−NH_2_), and two phenyl substituents at the 4,5-positions. This structure confers key physicochemical properties: the conjugated furan-phenyl system enables efficient UV absorption (matching standard MALDI laser wavelengths), while the −COOH/-NH_2_ moieties facilitate proton transfer, critical for analyte ionization. Its crystalline nature yields uniform, reproducible matrix-analyte deposits. In MALDI-MS analysis, ADFA excels in analyzing peptides, proteins, and pharmaceuticals. Key merits include low cost (vs specialty matrices), minimal background interference (especially in the low *m*/*z* range, <1,000), low analyte fragmentation (preserving intact molecular ions), and high vacuum stability (supporting long-duration analyses). These attributes have made it a reliable choice for both qualitative profiling (*e.g.*, peptide sequencing) and quantitative assays (*e.g.*, pharmaceutical formulations), addressing unmet needs in MALDI-MS for cost-effective, high-fidelity biomolecule detection.

DCH is a fluorescent dye containing phenolic hydroxyl groups derived from hydroquinone, which provide ideal sites for proton transfer, facilitating the generation of high-abundance ion signals in MALDI-MS.^458^ Liu et al. reported that DCH serves as a novel matrix for positive-ion MALDI-MS imaging, exhibiting strong UV absorption, low volatility under high vacuum (∼10^–7^ mbar), robust chemical stability, uniform matrix deposition, and high ionization efficiency. These properties enhance the *in situ* detection and imaging of lipids in biological tissues.^459^

5,1-ANL contains two key functional groups in its molecular structure: a naphthalene ring and an amino group. The naphthalene ring has strong UV absorption properties, effectively promoting analyte D/I, while the amino group has high proton affinity, facilitating the formation of stable ion-matrix complexes during the MALDI process, which enhances ion generation and detection.^460,461^ Osaka et al. identified 5,1-ANL as a novel matrix suitable for the MALDI ISD analysis of phosphorylated peptides.^462^ Their study evaluated the ISD fragment ion yields of phosphorylated peptides using different 1,5-naphthalene derivatives (5,1-ANL, 1,5-dihydroxynaphthalene) as matrices. The results showed that 5,1-ANL provided the highest ISD ion yield for mono-, di-, and tetraphosphorylated peptides, whereas DAN produced lower ISD ion yields for phosphorylated peptides.

Lumazine (1*H*-pteridine-2,4-dione) was identified by Calvano et al. as a novel MALDI matrix for analyzing complex PL mixtures. Lumazine exhibits photochemical stability under UV laser irradiation with minimal matrix-related ions, improving the detection of low-ionization lipids such as phosphatidylethanolamines (PEs), phosphatidylglycerols (PGs), phosphatidylserines (PSs), and phosphatidic acids (PAs). Its S/N for samples such as phosphatidylglycerol and phosphatidylethanolamine is an order of magnitude greater than that of the traditional matrix DHB. Lumazine has been successfully applied to MALDI analyses of crude lipid extracts from milk, soy milk, and eggs, to detect components such as phosphatidylethanolamine, phosphatidylserine, and phosphatidylinositol.^463^

OLZ is a benzodiazepine that is 10*H*-thieno­[2,3-*b*]­[1,5]­benzodiazepine substituted by a methyl group at position 2 and a 4-methylpiperazin-1-yl group at position 4. Musharraf et al. have explored the potential of the atypical antipsychotic drug OLZ as a matrix for MALDI-MS analysis of peptides aided with the theoretical studies.^464^ Seven small peptides were employed as target analytes to check the performance of OLZ and compared with conventional MALDI matrix CHCA. All peptides were successfully detected when OLZ was used as a matrix. Moreover, peptides angiotensin Ι and angiotensin ΙΙ were detected with better S/N ratio and resolution with this method as compared to their analysis by CHCA. Computational studies were performed to determine the thermochemical properties of OLZ in order to further evaluate its similarity to MALDI matrices, which were found in good agreement with the data of existing MALDI matrices.

Taken together, these mentioned above other SOM matrices for positive ion mode MALDI-MS have achieved remarkable advances, delivering targeted solutions to critical analytical needs. Briefly, HMPPA enables sensitive, robust protein/peptide analysis with impurity tolerance; osazones enhance oligosaccharide detection via strong UV absorption, proton donation, and uniform crystallization; ADFA offers low-cost, low-interference analysis of peptides, proteins, and pharmaceuticals with high vacuum stability; DCH supports high-efficiency lipid imaging in tissues via strong UV absorption and minimal volatility; 5,1-ANL maximizes ISD yields for mono-/di-/tetraphosphorylated peptides; lumazine outperforms traditional DHB in PL detection (*e.g.*, PEs, PGs) with superior S/N; and OLZ surpasses CHCA in peptide analysis (*e.g.*, angiotensins) with better resolution and S/N. Collectively, they extend MALDI-MS’ scope to LMW metabolites, modified peptides, and complex lipids. Future trends will prioritize structure-activity optimization (*e.g.*, tuning functional groups to reduce background), multiplexed analysis of diverse biomolecules, integration with high-resolution MS/imaging platforms for spatial metabolomics, and development of clinically compatible matrices for high-throughput diagnostic screening.

###### 3.1.1.14.2 Other SOM Matrices Used for Negative Ion Mode Detection

The continuous advancement and optimization of matrices for negative ion mode have significantly extended the applications of MALDI-MS, supporting both qualitative and quantitative analysis of biomolecules. A variety of other SOM matrices are now used in MALDI-MS studies under negative ion conditions. These include 1,4-dioxo-1,2,3,4-tetrahydrophthalazine-6-carboxylic acid (DTCA), 9-aminoacridine (9-AA), 1,6-diphenyl-1,3,5-hexatriene (DPH), 6-thioguanine (6-TG), *N*1,*N*4-dibenzylidenebenzene-1,4-diamine (DBDA), and 4-aminoazobenzene (AAB), all of which play important roles as matrices in MALDI-MS detection and analysis (Figure 24). The unique chemical properties of these matrices, including strong UV absorption, minimal matrix interference, and high ionization efficiency, significantly enhance the sensitivity and resolution of MALDI-MS analyses for detecting a diverse range of metabolites and lipids in complex biological matrices.

**Figure 24:**
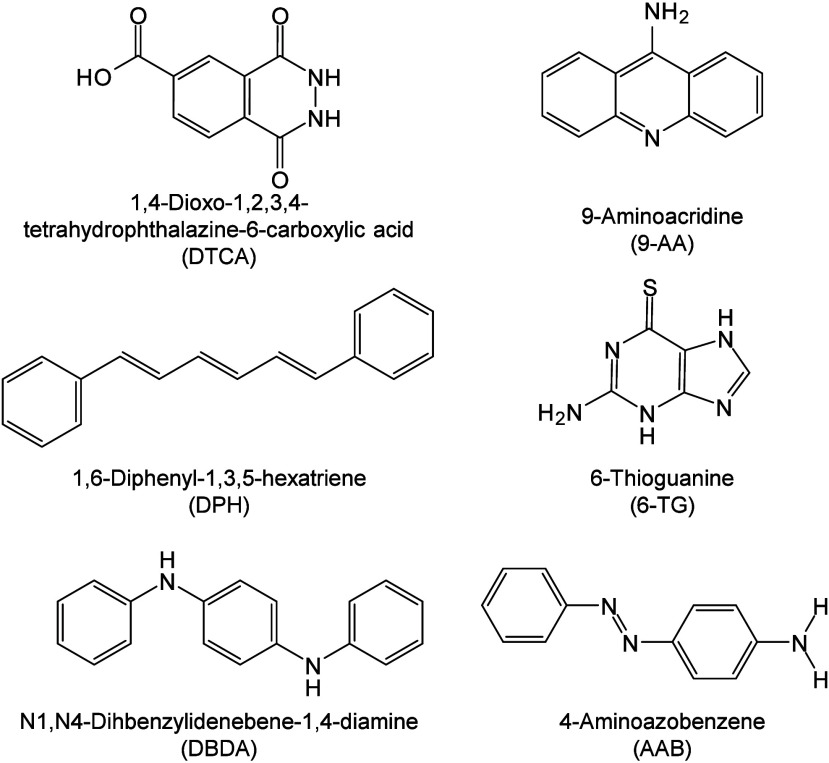
Chemical structures of other SOM matrices used for MALDI-MS negative ion mode detection.

DTCA features a tetrahydrophthalazine ring containing a 1,4-dioxo structure and a carboxyl group. This structure enables strong UV absorption, low background interference, and high ionization efficiency. Yan et al. discovered that DTCA, as a novel MALDI matrix, significantly enhances the analysis of metabolites induced by exposure to imidacloprid.^465^ DTCA has been used for the first time in the detection of LMW compounds, offering advantages such as strong UV absorption, low matrix background interference, high ionization efficiency, and excellent reproducibility. Based on these properties, DTCA was employed to analyze the changes in serum metabolites of mice exposed to imidacloprid and normal control group mice. By integrating machine learning techniques, the study successfully distinguished between imidacloprid-exposed mice and control group mice, identifying 39 potential biomarkers. Additionally, metabolic pathways that may be disrupted were revealed. The results demonstrate that DTCA, as a powerful novel positive-ion mode matrix, holds great potential for applications in small molecule detection.

9-AA is an aminoacridine derivative in which the hydrogen at the 9-position is replaced by an amino group. In 2002, Vermillion-Salsbury and Hercules first introduced 9-AA as a matrix for negative ion mode MALDI-MS.^466^ 9-AA is a moderately strong base, and its ionization mechanism in negative ion mode appears to involve proton abstraction (or deprotonation) of a labile proton in an acid-base reaction. While conventional MALDI matrices easily donate protons, 9-AA readily accepts protons, forming [M–H]^−^ anions.^466^ Since its introduction, 9-AA has been widely used in MALDI-MS as a classic matrix for detecting LMW compounds in negative ion mode and for successfully analyzing phenols, acids, sulfonates, amines, and alcohols.^467–470^

DPH is a polyene compound and is among the most widely used fluorescent probes in studies of biological membranes and PL bilayers because of its affinity for acyl chains in the bilayer. With consecutive pi bonds (C_6_H_5_-CH=CH-CH=CH-CH=CH-CH=CH-C_6_H_5_), DPH exhibits three vibrational peaks, with an absorption range of 330–380 nm, matching the emission wavelength of standard MALDI lasers.^471^ Ibrahim et al. identified DPH as a novel MALDI-MS imaging matrix for FAs, PLs, and sulfatides in brain tissue.^472^ DPH provides minimal matrix background interference in the analysis of small molecules (*m*/*z* < 1,000). In negative ion mode, DPH enables the high-sensitivity detection of small FAs as well as various larger lipids, including lysophospholipids (LPLs), PAs, PEs, PSs, PGs, phosphatidylinositols (PIs), and sulfatides. Its hydrophobic polyene structure and strong interactions with acyl chains contribute to the ideal of DPH characteristics as a matrix.

6-TG is a thiopyrimidine derivative that belongs to the class of pyrimidine compounds. Kimura et al. determined that nucleobase derivatives can serve as novel MALDI matrices for oligonucleotide analysis, providing close contact with analytes and increasing effectiveness compared with that of traditional matrices. They selected 16 nucleobase derivatives as candidate matrices for oligonucleotide analysis and conducted tests, with 6-TG showing outstanding performance. 6-TG induced prominent ISD fragments and successfully characterized 10 single-stranded RNA or DNA sequences, revealing new possibilities for the sequencing of oligonucleotides.^473^

DBDA is a highly sensitive matrix with low ion interference for small molecule analysis, demonstrating strong UV absorption and robust ionization capability at a laser wavelength of 355 nm. In negative ion mode, DBDA enables the successful detection of small molecules, providing reliable mass spectrometric data. Ling et al. reported that DBDA, as a novel matrix in negative ion MALDI-MS, exhibits excellent performance in the analysis of small molecules, including AAs, peptides, and nucleotides, and in quantitative fatty acid analysis.^474^ DBDA allows for the quantification of free FAs in serum on the basis of calibration curves, achieving a correlation coefficient of 0.99.

AAB is an azo compound in which an amino group is substituted at the 4-position of the phenyl ring. AAB functions both as a dye and an allergen, and it shares functional similarities with azobenzene. Wu et al. reported that AAB, as a novel negative ion mode matrix, significantly enhances the MALDI tissue imaging of metabolites.^185^ This study demonstrated that AAB has marked advantages in terms of UV absorption, matrix ion suppression, uniform crystallization, and metabolite ionization efficiency. Using AAB, successful *in situ* detection and imaging of metabolites in rat brain and germinating *Taxus* seed tissue sections were achieved, with 264 and 339 metabolite ion signals detected, respectively. Additionally, high-resolution MALDI-MSI imaging of mouse eye sections was performed using AAB, achieving a spatial resolution as high as 10 μm. These results indicate that AAB is an efficient negative ion MALDI matrix that enables the capture of metabolite images in biological tissue sections, thereby expanding the applicability of MALDI tissue imaging in metabolomics.

In summary, various other SOM matrices demonstrate significant potential in MALDI negative ion mode analysis. Matrices such as DTCA, 9-AA, DPH, 6-TG, DBDA, and AAB have effectively enhanced the sensitivity and spatial resolution of small-molecule metabolite and lipid detection due to their strong UV absorption, low matrix interference, high ionization efficiency, and uniform co-crystallization capabilities. Future research will continue to explore the development of novel matrices with specific functionalities and integrate data analysis methods such as machine learning to further advance the application of MALDI-MS in metabolomics, lipidomics, and multi-omics integration analysis.

###### 3.1.1.14.3 Other SOM Matrices Used for Dual-Polarity Ion Mode Detection

The ongoing development and optimization of dual-polarity matrices have significantly broadened the applications of MALDI-MS, enabling comprehensive qualitative and quantitative analysis of biomolecules. Several MALDI matrices can be utilized in both positive and negative ion modes. These include 6-aza-2-thiothymine (ATT), quinaldic acid (QA), lignin, 1,1′-binaphthyl-2,2′-diamine (BNDM), dansyl compounds, and Basic Blue 7 (BB7), all of which play significant roles as dual-polarity matrices in MS detection and analysis (Figure 25).

**Figure 25:**
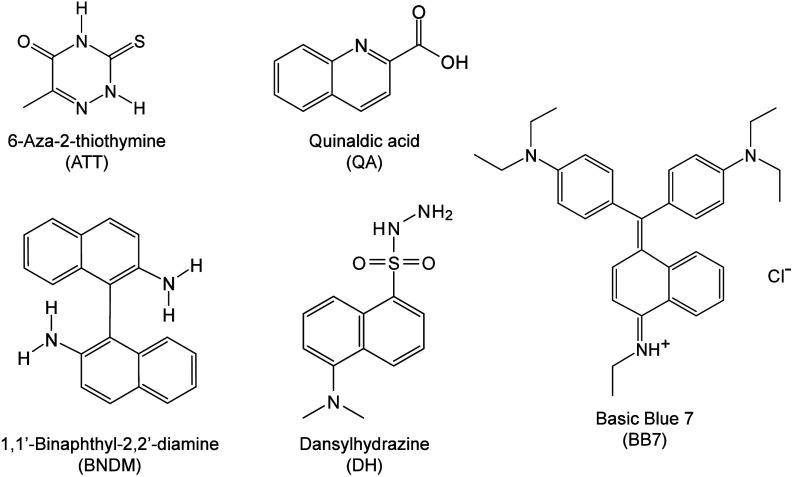
Chemical structures of other SOM matrices used for MALDI-MS dual-polarity ion mode detection (lignin, an extraordinarily intricate polymeric mixture, defies straightforward structural delineation. So, its specific structure is not provided here).

ATT is a pyrimidine derivative containing nitrogen and sulfur. Its structure includes a pyrimidine ring similar to that of thymine, but with an oxygen at the 6-position replaced by nitrogen and an oxygen at the 2-position replaced by sulfur. These substitutions confer favorable light absorption properties and low toxicity, making ATT suitable for use as a MALDI matrix.^475^ Lecchi et al. demonstrated that ATT is an efficient MALDI-MS matrix for the analysis of oligonucleotides. Compared with conventional matrices, ATT provides high resolution, accuracy, and reproducibility, with no fragmentation or depurination of oligonucleotides during analysis, producing high-quality signal peaks and facilitating straightforward sample preparation.^475^ Additionally, Lecchi et al. used ATT in the presence of ammonium citrate (AC) as a matrix for the MALDI-MS detection of intact double-stranded DNA, marking the first demonstration of MALDI spectra for complete double-stranded DNA.^476^ Furthermore, Ohara et al. confirmed that ATT can serve as a matrix for characterizing noncovalent interactions between guanidine probes and single-stranded DNA, offering a new approach to studying interactions between guanidine derivatives and DNA.^477^

QA, a 2-quinoline carboxylic acid with a carboxyl group (−COOH) at the quinoline ring’s 2-position, was first identified as a novel matrix for MALDI-MS for nucleic acid analysis by Song.^478^ QA’s physicochemical properties stem from its unique structure: the fused benzene-pyridine quinoline core enables strong UV absorption (matching 337 nm nitrogen UV laser wavelengths), while the 2-position −COOH facilitates proton transfer, critical for nucleic acid ionization. QA specializes in nucleic acid (DNA/RNA) MALDI-MS analysis. Optimized dissolution and preparation protocols enhance mass resolution, ion yielding consistent, reproducible signals by minimizing adduct formation and matrix background interference, longstanding challenges in nucleic acid MALDI-MS. This performance positions QA as a promising tool for DNA/RNA profiling, with potential in fields like molecular diagnostics and genomics, addressing the need for cost-effective, high-fidelity nucleic acid detection via MALDI-MS.

Lignin is the second most abundant compound in plant biomass after cellulose and consists of an amorphous, three-dimensional (3D) cross-linked polymer of phenylpropane units. Owing to its high hydrophobicity and unique structural characteristics, lignin generates minimal signals under MALDI conditions, particularly in the low mass range, effectively reducing matrix interference in small molecule analysis. Zhao et al. were the first to apply lignin in dual-ion mode MALDI analysis, successfully identifying 30 different small molecules, including the quantitative detection of six representative molecules, and demonstrated a good linear response.^479^ Further characterization through UV and NMR spectroscopy revealed the potential mechanisms of lignin as a matrix, providing additional theoretical support for the rational design of matrices.

BNDM was identified by Sun et al. as a novel MALDI matrix with enhanced *in situ* imaging capabilities for lung cancer metabolic heterogeneity.^480^ BNDM, as a MALDI matrix, offers low background interference, high sensitivity, and suitability for both positive and negative ion modes, making it useful for metabolite detection and imaging. Using BNDM as a matrix, MALDI-MSI was successfully performed for 301 negative metabolite ions and 175 positive metabolite ions, covering a range of LMW molecules and lipid components such as AAs, organic acids, nucleosides, nucleotides, nitrogenous bases, cholesterol, peptides, FAs, choline, carnitine, polyamines, creatine, and PLs.

Dansyl chromophores are often combined with specific chemical functional groups to increase the fluorescence of target biomolecules. These chromophores can also absorb energy from UV laser wavelengths commonly used in MALDI-MS. Godfrey et al. explored dansyl compounds as novel MALDI matrices for proteomics.^481^ Dansyl hydrazine (DH), emits fluorescence upon absorption of UV light at 340 nm and has previously been used to improve the detection and quantification of glycoproteins and hormones.^482^ The other three dansyl compounds (dansylcadaverine, dansyl-dl-α-aminooctanoic acid, and 11-(dansylamino)­undecanoic acid) emit fluorescence upon absorbing UV light at 335 nm and are fatty acid analogs that were previously used to enhance the detection of nonpolar polymers via fluorescence. They studied the fluorescence properties of these new matrices on tissues and compared them with those of the traditional matrices, CHCA, DHB, and SA.^483^ Among the dansylated matrices, DH proved the most effective for protein identification, producing the expected peptide ions and demonstrating competitive performance compared to CHCA and DHB.

BB7 is systematically named *N*-(4-((4-(diethylamino)­phenyl)­(4-(ethylamino)­naphthalen-1-yl)­methylene)­cyclohexa-2,5-dien-1-ylidene)-*N*-ethylethanaminium chloride. It belongs to the class of triarylmethane dyes.^484^ BB7 is a heterocyclic compound containing multiple nitrogen atoms,^485^ typically used for dyeing paper, wool, silk, or nylon, as well as for ink production.^486^ Owing to the sufficient solubility of this dye in water (20 mg·mL^–1^), the sample preparation process is extremely simple, similar to traditional histological staining methods, with no need for the prior chemical fixation of tissues. The tissue-mounted glass slide can simply be immersed in the dye solution. This provides a novel and straightforward approach for MALDI MSI sample preparation. Javorek et al. reported that BB7 serves as a novel dual-polarity matrix for tissue staining in MALDI-MSI.^487^ Using a subatmospheric pressure MALDI source and an orbitrap mass spectrometer, they performed MSI analysis of lipids in mouse brain tissue sections in both positive and negative ion modes. The results were compared with those obtained using traditional matrices such as DHB and DAN. BB7 performed exceptionally well, particularly in negative ion mode, where the signal intensity of many lipid species was notably high. Compared with DAN or DHB, BB7 is a relatively hot matrix, which specifically refers to its ability to promote greater fragmentation of analytes under laser irradiation.^488^ Consequently, using BB7 for lipid analysis induces in-source fragmentation of certain lipids and requires a higher laser pulse energy for optimal results. The use of this hot matrix potentially increases the complexity of the mass spectrum. However, it also provides additional structural information about the analytes. In contrast to DAN, which is a potential carcinogen, BB7 is generally safe. The strong blue staining of tissues makes it easy to inspect, and it can even be used directly for histological labeling, simplifying the comparison of ion images with different tissue regions. BB7 belongs to the diverse class of triarylmethane dyes, and it is expected that many of these dyes will exhibit similar or even superior performance in MALDI-MSI.

Dual-polarity ion mode MALDI matrices represent a recent innovation. The matrix molecules are designed with dual functionality: one to donate protons and the other to accept protons. The dual-polarity matrix enables simultaneous acquisition of positive and negative ion mass spectra, facilitating the mitigation of ion suppression effects. This dual-ion mode design increases the intensity and stability of mass spectrometric signals, thereby improving analytical sensitivity and accuracy. The design and optimization of dual-ion mode MALDI matrices require consideration of various factors, including the chemical properties of the matrix, the rate and stability of ion release, and the interactions with the target analytes. In practical applications, dual-polarity mode MALDI matrices have been widely used in biomedical research, drug development, and clinical diagnostics. With ongoing development and optimization, dual-ion mode MALDI matrices will continue to play a critical role, driving advancements and innovation in the field of MALDI-MS analysis.

##### 3.1.1.15 Summary

SOMs play an integral role in MALDI-MS. These matrices are predominantly LMW aromatic compounds, strategically functionalized to optimize photon absorption, proton transfer, and ionization efficiency. The selection of an appropriate matrix is critical for achieving high ionization efficiency and strong MS signal intensity. In UV-MALDI, matrix molecules must exhibit not only a high molar absorption coefficient but also suitable chemical properties to facilitate efficient ionization. Structurally, SOMs can be categorized into several principal classes, including derivatives of cinnamic acid, acetophenone, thiazole, benzoic acid, anthracene, flavonoid, coumarin, pyridine, benzophenone, nitrobenzene, pyridazine, and indole. A key determinant of their performance lies in the presence of specific functional groups. Proton-donating groups such as hydroxyl or amino moieties promote protonation in positive ion mode, whereas acidic or electron-accepting groups such as carboxyl or nitro functionalities facilitate deprotonation or electron capture in negative ion mode. This structural versatility enables tailored interactions with diverse analytes, significantly enhancing D/I efficiency, signal intensity, and reproducibility across various sample types. Ongoing refinement in matrix design, particularly through functional group engineering and hybridization of structural motifs, holds considerable potential for further improving the sensitivity, specificity, and quantitative capabilities of MALDI-MS. Future advances are expected to open new frontiers in omics research, precision medicine, pharmaceutical development, and environmental monitoring, underscoring the essential role of functionalized SOMs in next-generation mass spectrometry.

#### 3.1.2 Designed and Synthesized Organic Matrices

An attractive strategy for developing novel MALDI matrices involves their rational design, where the core structure of the classic matrix is intentionally modified to achieve specific physical and chemical properties.^175^ This represents a step toward replacing the predominantly empirical approach to matrix selection with a rational approach, which was typical of MALDI in early days. Jaskolla et al were pioneers in rational design of matrices.^54^ These scholars synthesized many CHCA and DHB derivatives with the aim of adjusting their proton affinity and enhancing the ionization efficiency of analytes. The development of novel matrices can be divided into four categories according to the methods used for their rational design: reactive matrices, matrix derivatization, ionic liquid matrices, and binary or mixed matrices (Figure 2 and Table S4).

##### 3.1.2.1 Reactive Matrices

Reactive matrices can function as both MALDI matrices and derivatization reagents, enhancing the ionization yield of analytes and providing structural information. The term “reactive matrix” was coined by Brombacher et al., who described a unique matrix compound that possesses both reagent and matrix characteristics, facilitating the rapid derivatization of small molecules with specialized functions and thereby supporting efficient ionization and detection by MALDI-MS.^489^ In other words, this reagent should exhibit dual functionality as both a derivatization reagent and a MALDI matrix, containing fragments that absorb UV light to promote D/I and active functional groups that react specifically with analytes.

In principle, a true reactive matrix not only chemically modifies analytes but also absorbs UV laser energy, eliminating the need for any standard MALDI matrix and thereby reducing sample preparation time. Notably, some have proposed naming methodologies involving reactive matrices such as “reactive MALDI-MS” (*Re*MALDI-MS)^490^ or “reactive matrix LDI-MS” (RM-LDI-MS).^491^ Reactive matrices serve not only as the matrix in MALDI-MS but also as derivatization reagents that selectively react with specific functional groups in analytes. The resulting derivatives can exhibit more efficient ionization, higher mass resolution, or beneficial molecular mass shifts. The use of chemical derivatization reagents followed by conventional matrices, such as CHCA or DHB, also falls under the category of reactive matrices.^489,492,493^

Recently, an excellent review discussed histochemical derivatization reagents for MALDI-MSI (OTCT reagents).^260^ In this study, we adopt a more specific definition of reactive matrices, focusing on organic compounds that play dual roles as both derivatizing agents and functional matrices. These compounds can detect a variety of functional groups, including carbonyl, amino, and hydroxyl groups, as well as C=C double bonds (DBs), disulfide bonds, and electron-transfer sites. Reactive matrices can be further classified into Schiff base synthesis reactive matrices, Wolff–Kishner reduction reactive matrices, condensation reaction matrices, addition reaction matrices, specialized chemical reaction matrices, and electron transfer reaction matrices.

###### 3.1.2.1.1 Schiff Base Synthesis Reaction

Carbonyl groups, such as aldehydes and ketones, react with amines to form corresponding Schiff bases.^494^ The 10 reactive matrices that enhance MALDI-MS detection efficiency through Schiff base formation include anthranilic acid (AA), aminopyrazine (AP), 3-aminoquinoline (3-AQ), 2-phenyl-3-(*p*-aminophenyl) acrylonitrile (PAPAN), 9-(3,4-diaminophenyl)­acridine (DAA), α-cyano-3-aminocinnamic acid (3-CACA), 2,4-dihydroxybenzaldehyde (2,4-DHBA), and tryptamine. In contrast, nonreductive amination, including the Schiff base synthesis reaction, refers to organic reactions that convert amides into non-reducing amines.^495^ In a basic environment, the carbonyl group (C=O) in amides undergoes nucleophilic addition with ammonia or amine derivatives to yield corresponding amine compounds, with the concurrent elimination of a water molecule.^496^ Common reagents for nonreductive amination include ammonia, various amine compounds (such as ethylenediamine and methylamine), and hydroxylamine, which react with the carbonyl group in amides to produce nonreducing amines.^497^ Nonreductive amination has broad applications in organic synthesis, allowing the production of amine compounds with diverse structures. Because it does not involve reductive reactions, this process typically occurs under mild reaction conditions, making it suitable for substrates or functional groups that are sensitive to reduction.^498^

Zhang and Gross were the first to employ AA as a reactive matrix for analyzing oligodeoxynucleotides.^499^ The amino group on AA reacts with the aldehyde functional group at the basic sites of oligodeoxynucleotides, forming Schiff bases that exhibit intense signals with a mass increase of 119 Da. This derivatization enhances distinct fragmentation, facilitating the identification and localization of basic sites. Notably, Zhang and Gross pioneered the use of matrix-assisted chemical labeling for modified oligodeoxynucleotides, a principle known as *in situ* derivatization, coupled with MALDI-MS. The *in situ* MALDI matrix, a mixture of AA, NA, and AC, reacts with the basic sites of modified oligodeoxynucleotides to form Schiff bases.^499^ AP is another derivatization reagent with an amino-heterocyclic structure that is suitable for increasing the hydrophobicity of oligosaccharides. Cai et al. used AP as both a derivatization reagent and a matrix to improve the MALDI-MS analysis of oligosaccharides.^500^ Following nonreductive amination, AP serves as a MALDI matrix, eliminating the need for purification steps. Under optimal conditions, nearly complete derivatization was achieved, enhancing the S/N of oligosaccharides by a factor of approximately 2-6, with high signal reproducibility (Figure 26). Derivatization with AP is convenient, complete, and free of desalting steps.

**Figure 26:**
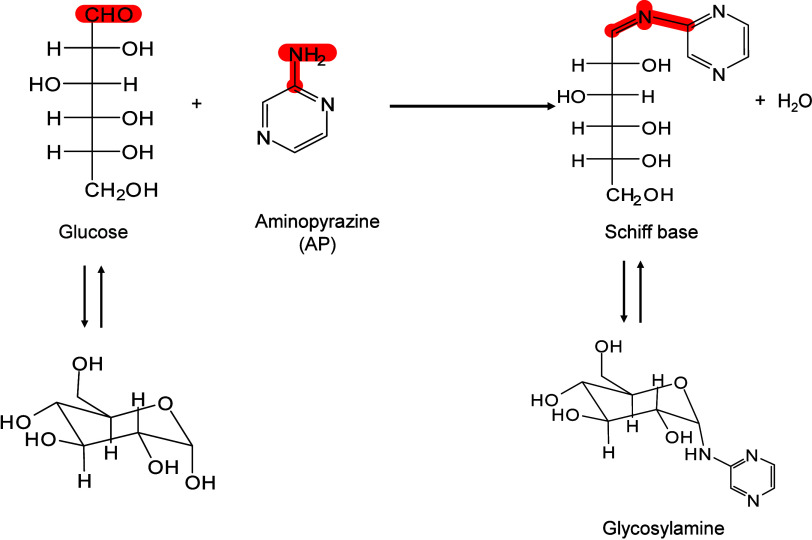
Derivatization of a monosaccharide with AP through a nonreductive amination reaction.

3-AQ is another highly efficient reactive matrix for oligosaccharide analysis. Rohmer et al demonstrated that 3-AQ can serve as both a matrix and derivatizing agent for the MALDI-MS analysis of oligosaccharides. When 3-AQ is used for on-target derivatization, no additional purification steps are needed, and Schiff bases are formed, enabling measurement in both positive and negative ion modes.^501^ By optimizing the reaction conditions, rapid, complete, and reproducible targeted derivatization of all oligosaccharides with 3-AQ was achieved (Figure 27). This approach enhanced PSD fragmentation in both positive and negative ion modes, providing detailed information on oligosaccharide sequences. Ling et al. identified PAPAN as a reactive matrix for the MALDI-MS analysis of glycans. PAPAN, a derivative of CHCA, features a CHCA core unit and exhibits high ionization efficiency under laser irradiation.^502^ PAPAN reacts with terminal aldehydes on glycans, increasing glycan ionization efficiency through nonreductive amination with reducing-end aldehydes, which improves glycan ionization in MALDI-MS analysis. Additionally, PAPAN selectively inhibits peptide ionization, allowing the selective detection of *N*-glycoproteins in deglycosylated trypsin digests without the need for prepurification (Figure 28). A specialized high-ionization reactive matrix, DAA, was initially synthesized by Plater et al. as a fluorescent probe for nitric oxide detection. In MALDI-MS, DAA specifically derivatizes α-dicarbonyl compounds, forming quinoxaline products with high mass and ease of ionization, achieving low detection limits and high sensitivity.^503^ Similarly, Mugo and Bottaro utilized DAA as a reactive matrix for the rapid analysis of α-dicarbonyl compounds via MALDI-MS.^504^ The synthesis of DAA proceeds through two key steps. First, anthracene undergoes electrophilic chlorination with hydrogen chloride gas. While this reaction has the potential to produce isomeric chlorinated by-products due to the multiple reactive sites on the anthracene ring, the authors optimized conditions (HCl amount, temperature, time) and employed TLC and high-performance liquid chromatography (HPLC) monitoring to ensure that 9-chloroacridine was obtained as the dominant product. Subsequently, 9-chloroacridine reacts with *o*-phenylenediamine via nucleophilic substitution to furnish DAA (Figure 29). The reaction of DAA with α-dicarbonyl compounds exclusively yields vacuum-stable dicarbonyl-quinoxaline acridine derivatives, which display excellent ionization efficiency in positive ion mode without the need for additional matrices (Figure 30). Furthermore, Liao et al. developed a novel reactive matrix for MALDI, namely 3-CACA, which cocrystallizes with the conventional matrix CHCA to significantly increase derivatization efficiency. Their study demonstrated that this 3-CACA/CHCA matrix achieved exceptionally low detection limits, reaching femtomole levels in *N*-glycan analysis, while also exhibiting excellent sensitivity, qualitative performance, and robust linear quantification capabilities (*R* ^2^ > 0.998) (Figure 31).^282^

**Figure 27:**
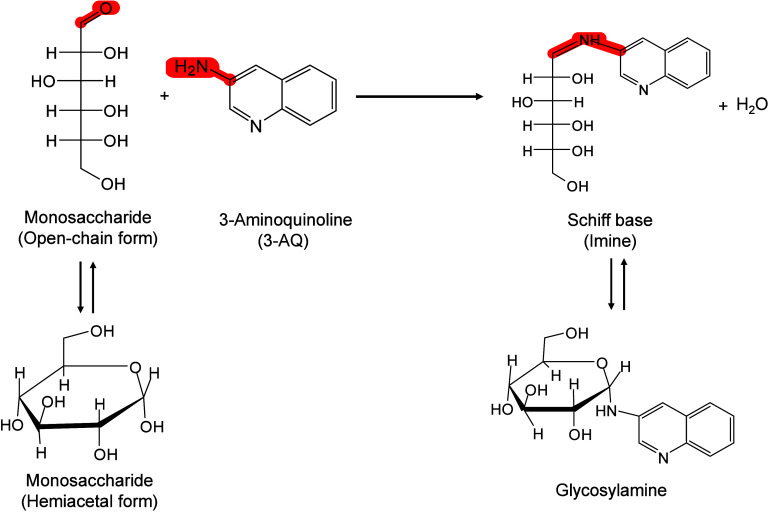
Schiff base formation from 3-AQ and the reducing end of monosaccharide.

**Figure 28:**
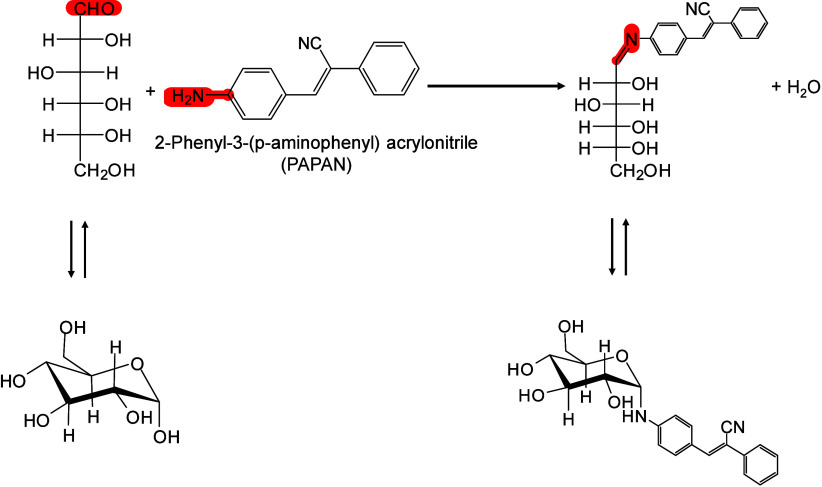
Nonreductive amination reaction of PAPAN with monosaccharide.

**Figure 29:**
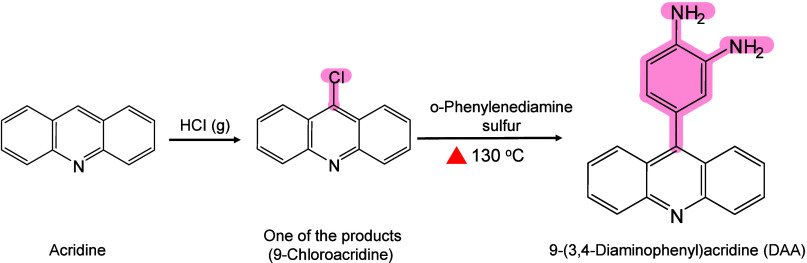
Synthetic scheme for the derivation of DAA.

**Figure 30:**
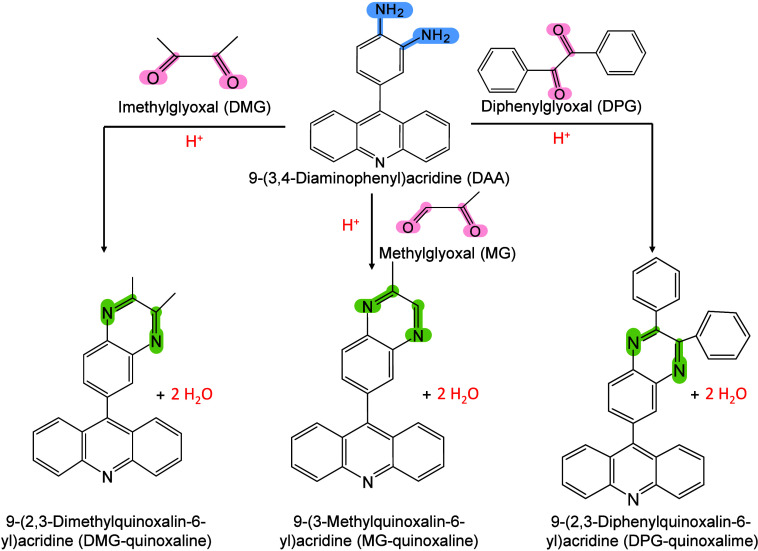
Derivatization reaction: acid-catalyzed condensation reaction of DAA with α-dicarbonyl compounds (methylglyoxal, dimethylglyoxal, and diphenylglyoxal) to form their respective quinoxalines.

**Figure 31:**
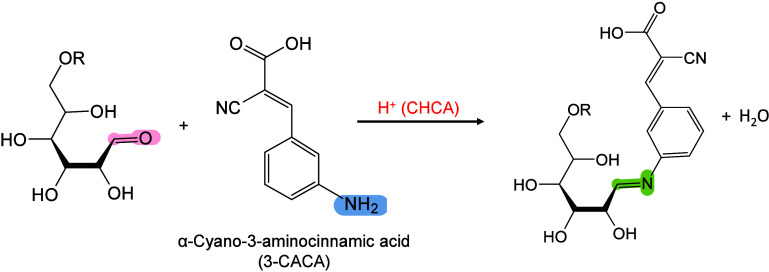
On-target non-reductive amination reaction between 3-CACA and carbohydrate with CHCA as a catalyst.

Phenolic compounds, such as 2,4-DHBA and DHAP, serve as reactive matrices. Zaikin et al. investigated several aromatic carbonyl compounds, including 2,4-DHBA, DHAP, 2,3,5-trihydroxybenzaldehyde, and 2,4-dinitrobenzaldehyde, for use as potential reactive matrices for primary amines and used MALDI-MS to analyze monoamines, diamines, and polyamines.^505^ Although all these compounds can form Schiff bases with primary amines, only 2,4-DHBA and DHAP demonstrate efficient D/I capabilities under MALDI-MS, demonstrating the combined functionality of the derivatization reagent and MALDI-efficient matrix. Slyundina et al. identified tryptamine as an excellent reactive matrix for MALDI-MS analysis.^506^ They used aromatic-substituted amines as derivatizing matrices and assessed their applicability in analyzing aliphatic, alicyclic, and aromatic ketones and aldehydes. The results showed that tryptamine functions effectively as a matrix without the need for additional matrix compounds, generating high S/N spectra of modified ketones and aldehydes while exhibiting high D/I efficiency of the derivatives (Figure 32).

**Figure 32:**
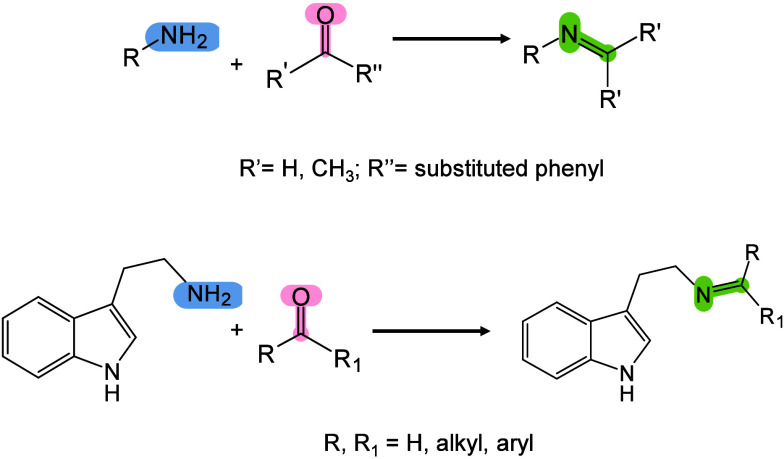
Derivatization reaction of primary amines with nonpolar carbonyl compounds.

###### 3.1.2.1.2 Wolff–Kishner Reduction Reaction

The condensation of hydrazine with carbonyls to form hydrazones has historically been used by Emil Fischer and others for the characterization of carbohydrates.^507^ Numerous MALDI matrices have been developed to improve ionization efficiency in MALDI-MS through this reaction. Notable examples include 2,4-dinitrophenylhydrazine (DNPH), 4-dimethylamino-6-(4-methoxy-1-naphthyl)-1,3,5-triazine-2-hydrazine (DMNTH), 2-nitro-4-carboxyphenylhydrazine (NCPH), 2,4-dicarboxylphenylhydrazine (DCPH), 2-hydroxybenzohydrazide (HBH), 3-hydroxy-2-naphthoic acid hydrazide (3-HNAH), 2-hydrazinoquinoline (2-HQ), 2-hydrazinopyrimidine (2-HPM), and 2-hydrazinoterephthalic acid (2-HTA).

In a pioneering study that introduced the term “reactive matrix”, DNPH was employed as a compound to analyze corticosteroids.^489^ DNPH readily forms hydrazones and exhibits enhanced D/I upon protonation and sodiation, without the need for standard matrix addition. Similar approaches have been applied to analyze natural and oxidized lipids, PLs, and carbonyl-containing peptides.^493,508^ Specifically, Brombacher et al. utilized DNPH as a reactive matrix in an automated coupling of capillary HPLC with MALDI-MS for the derivatization and ionization of small molecules, particularly steroids.^489^ The column eluate mixed with DNPH directly enters the UV detector, enabling efficient analysis. Fenaille et al. employed DNPH as a reactive matrix in MALDI-MS to detect peptides modified by 4-hydroxy-2-nonenal (HNE) in undigested samples, achieving the selective detection of modified peptides through the high D/I yield of hydrazone derivatives (Michael adducts with dinitrophenylhydrazine) (Figure 33).^493^ Schiller’s group introduced DNPH as a reactive matrix to analyze natural and oxidized lipids containing aldehyde groups.^508^ Additionally, DMNTH is a “tailor-made” derivatization reagent that combines selective reactivity, stability, and favorable spectroscopic properties.^509^ Developed by the Karst group at the University of Münster in Germany, DMNTH is synthesized via substitution reactions on melamine, where the melamine base molecule plays a specific role in the detectability, reactivity, and polarity of the reagent. Flinders et al. employed hydrazine-based derivatization agents to detect carbonyl-containing compounds via MALDI-MSI, enhancing sensitivity.^492^ The results indicate that DMNTH outperforms DNPH in terms of detection efficiency. Furthermore, extended reaction times improve the detection limits of protonated hydrazone derivatives, whereas combining the conventional MALDI matrix CHCA with DNPH/DMNTH reactive matrices enhances both the sensitivity and detection limits. Mugo and Bottaro utilized the custom reactive matrix DMNTH in MALDI-TOF-MS to directly derivatize carbonyl compounds, improving detection performance.^509^ Under UV laser irradiation, DMNTH ionizes strongly and selectively reacts rapidly with carbonyls. The resulting hydrazones are readily detectable by MALDI-TOF-MS without additional sample preparation, significantly simplifying the analytical process (Figures 34 and 35).

**Figure 33:**
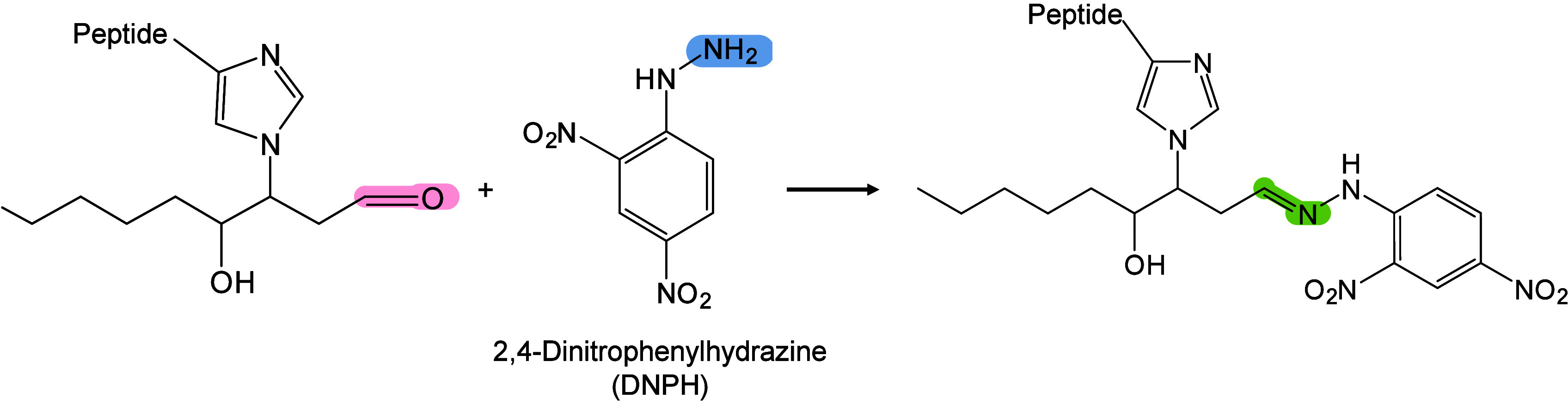
Structures of HNE-modified peptides before and after DNPH derivatization.

**Figure 34:**
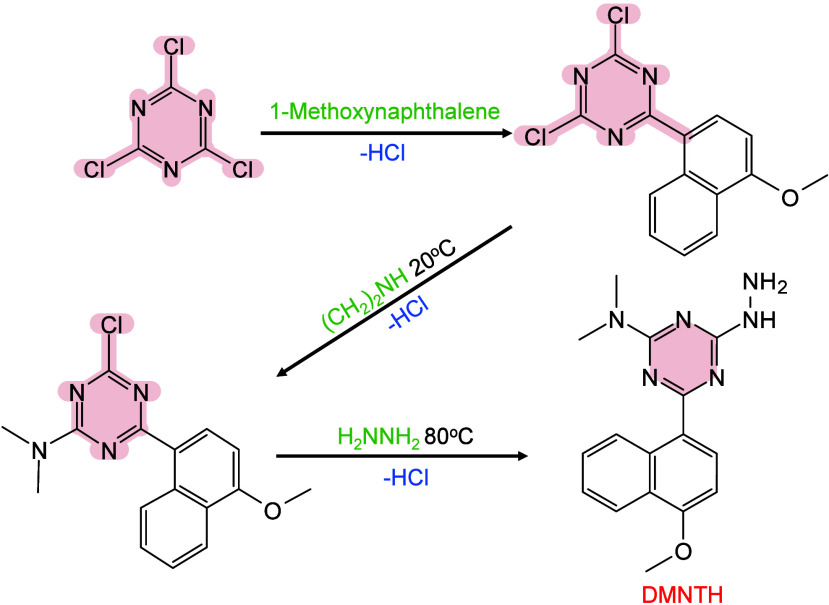
Synthesis of 4-dimethylamino-6-(4-methoxy-1-naphthyl)-1,3,5-triazine-2-hydrazine (DMNTH).

**Figure 35:**
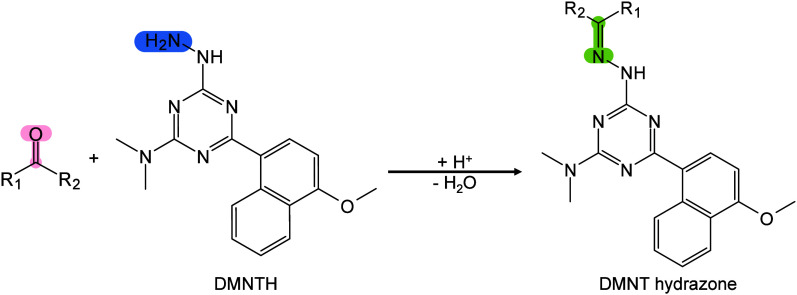
General scheme for reaction of DMNTH with carbonyl compounds to produce DMNT hydrazones.

2-Nitro-4-carboxyphenylhydrazine (NCPH) and 2,4-dicarboxylphenylhydrazine (DCPH) were developed as a novel pair of reactive MALDI matrices for the rapid and accurate analysis of *N*-glycoproteins in dual-ion mode. The introduction of active groups through derivatization reactions enables selective activation of oligosaccharide ionization. Liao et al. rationally designed and synthesized these two reactive matrices, which were separately combined with the conventional acidic matrix DHB to form composite matrices that exhibited targeted derivatization efficiency and significantly enhanced *N*-glycan ionization capability for high-performance MALDI analysis in dual-ion mode.^510^ Using the two composite matrices, they achieved mass calibration in dual-ion mode MALDI-MS and MS^2^ through postderivatization detection and dextran fragmentation, while selectively improving the ionization efficiency of oligosaccharides in complex mixtures. Homogeneous co-crystallization ensures excellent linearity and reproducibility for *N*-glycan quantification. Furthermore, each *N*-glycan labeled with NCPH and DCPH generates a characteristic mass shift in dual-ion mode, facilitating rapid identification and cross-validation to further improve quantitative accuracy. This approach provides an effective strategy for sensitive and reliable *N*-glycoprotein analysis with enhanced specificity and precision (Figure 36).

**Figure 36:**
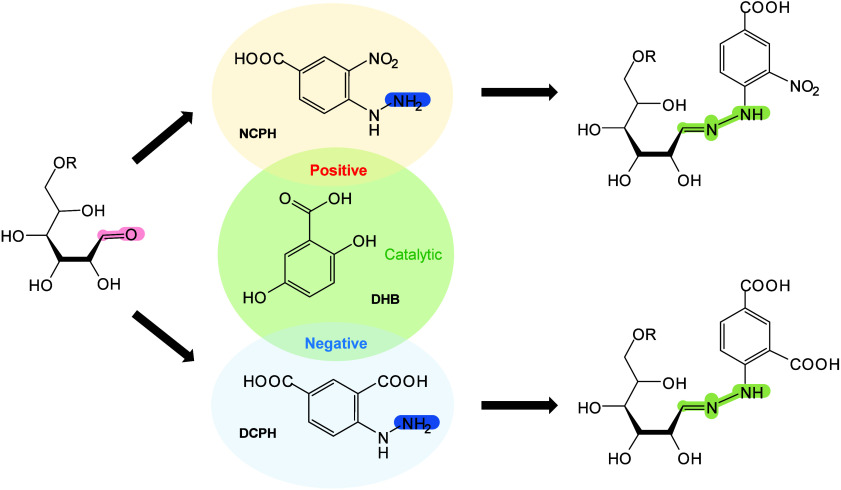
Derivatization reaction of NCPH/DHB and DCPH/DHB composite matrices with *N*-glycans.

Several other hydrazides or hydrazide-containing compounds can also act as reactive matrices for volatile carbonyl-containing aldehydes (such as formaldehyde, acetaldehyde, and propionaldehyde)^511^ and ketones (such as acetone, methyl ethyl ketone, and methyl isobutyl ketone).^512^ It is worth noting that ketones typically react slower than aldehydes in nucleophilic addition reactions with hydrazide groups due to greater steric hindrance and lower electrophilicity of the carbonyl carbon.^513^ Shigeri and colleagues reported that hydrazides and hydrazine-based reagents can serve as novel reactive matrices for the MALDI-MS detection of gaseous aldehydes and investigated the functions of 19 hydrazides and 14 hydrazine reagents.^511^ The results revealed that two hydrazides (HBH and 3-HNAH) and two hydrazine reagents (2-HQ and DNPH) could effectively react with the carbonyl groups of gaseous aldehydes (such as formaldehyde, acetaldehyde, and propionaldehyde). This study was the first to report the use of novel reactive matrices, specifically derivatization agents with enhanced D/I efficiency, for direct one-step derivatization and detection of gaseous molecules via MALDI-MS (Figure 37). Shigeri et al. reported that 2-HQ served as a reactive matrix for the MALDI-MS detection of gaseous carbonyl compounds, yielding spectra with low background and minimal noise, and demonstrated a sensitivity of at least parts per million for gaseous aldehydes and ketones, with acidic conditions being crucial to its functionality (Figure 38).^512^ Furthermore, Shigeri and colleagues explored 51 reagents (37 hydrazides and 14 hydrazines) as reactive matrices in MALDI-MS for detecting carbonyl-containing steroids.^514^ The findings revealed that 3-HNAH exhibited the highest reactivity with carbonyl steroids, detecting steroid levels as low as 1 pmol, and that its use as a derivatization agent significantly improved MALDI-MS D/I efficiency, outperforming DNPH (Figure 39).

**Figure 37:**
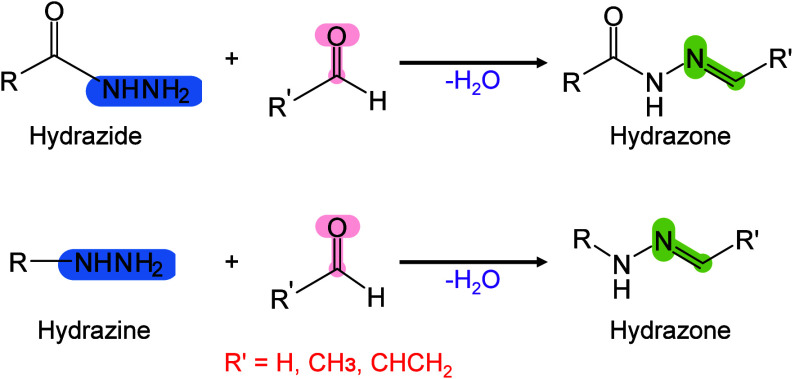
Condensation reactions of hydrazide and hydrazine derivatives with gaseous aldehydes (formaldehyde, acetaldehyde and propionaldehyde).

**Figure 38:**
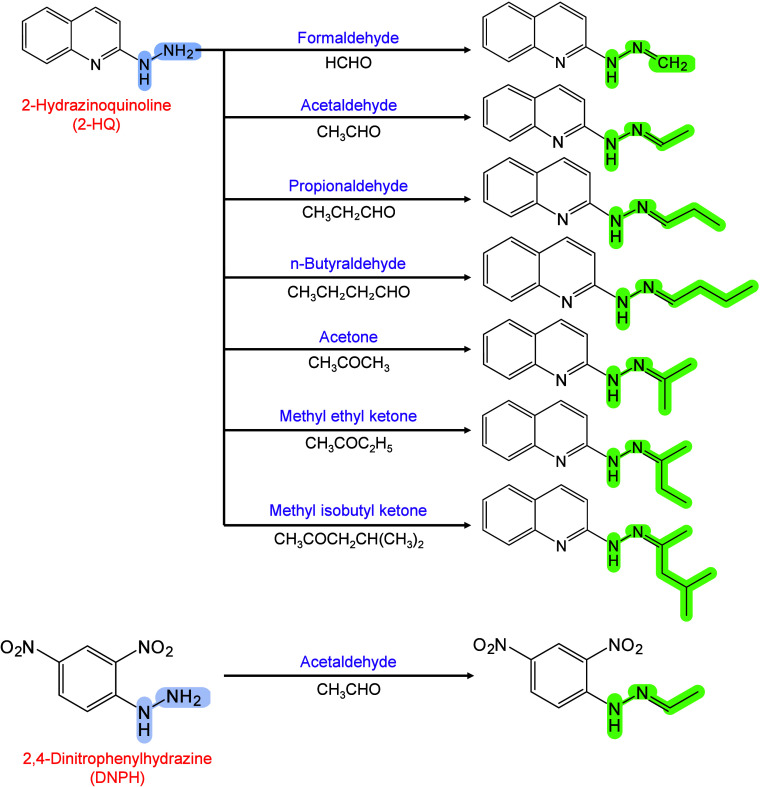
Condensation reactions of 2-HQ and DNPH with gaseous aldehydes (formaldehyde, acetaldehyde, propionaldehyde, and *n*-butyraldehyde) and ketones (acetone, methyl ethyl ketone, and methyl isobutyl ketone).

**Figure 39:**
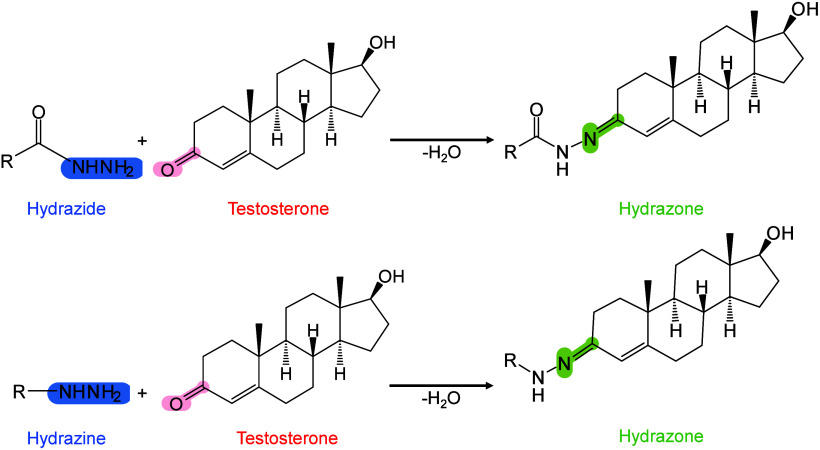
Condensation reactions of hydrazide and hydrazine reagents with steroids (testosterone).

Owing to its two electron-withdrawing imine nitrogen atoms^515^ and pyrimidine ring structure,^516^ 2-HPM can enhance the reactivity of its hydrazino group toward the reducing end of oligosaccharides and amplify the MALDI-MS signal of its targets. It has therefore been proposed as a derivatization reagent for rapid and sensitive oligosaccharide analysis. Jiang and colleagues utilized 2-HPM as both a derivatization reagent and comatrix, enabling the rapid, selective, and quantitative derivatization of oligosaccharides while promoting their ionization and enhancing the MALDI-MS signal. This approach simplifies the enrichment and purification steps prior to analysis (Figure 40).^517^ Additionally, Wang et al. employed the 2-HPM matrix for MALDI-FTMS analysis of cholesterol and fatty alcohols in human hair.^516^ This study combines the direct pyridinium quaternization with MALDI-FTMS, which offers a perspective and an alternative tool for the identification and quantification of substances in a biological matrix by comparing d_0_/d_5_ pairs, especially when isotope-labeled internal standards are unavailable.

**Figure 40:**
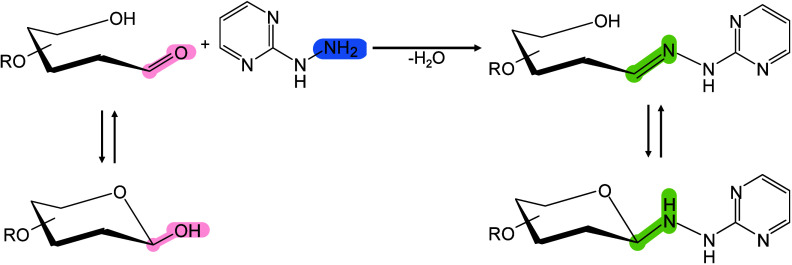
2-HPM derivatization of oligosaccharides.

Wang and colleagues identified 2-HTA as a novel negative ion MALDI matrix suitable for the qualitative and quantitative MALDI-MS analysis of *N*-glycans.^518^ They synthesized a series of hydrazine-benzene dicarboxylic acid-reactive MALDI matrices, among which 2-HTA demonstrated outstanding performance. It can cocrystallize uniformly with glycans and produce high-intensity deprotonated ions in negative-ion mode. They proposed that weak acids could catalyze nonreducing amination reactions and enhance derivatization reactivity and ionization efficiency if traditional organic acid matrices were combined with acid-catalyzed reactive matrices. Accordingly, they combined four commonly used acidic matrices (DHB, CHCA, SA, and CA) with 2-HTA to obtain four combined matrix systems. Among these combinations, SA/2-HTA exhibited superior sensitivity and reproducibility, rendering it particularly effective for the quantitative analysis of *N*-glycans.

###### 3.1.2.1.3 Condensation Reaction

Pyrene cations, which contain a positively charged oxygen atom in a six-membered aromatic heterocycle, can be used for the analysis of analytes with amine functionalities. Pyrene reacts with primary amines to form the corresponding pyridinium cations, a transformation typically carried out under mild conditions at room temperature. This characteristic has been utilized to increase the sensitivity of MALDI-MS for a variety of proteins and small molecules.^519–521^ Many neuroactive substances, including endogenous biomolecules, environmental compounds, and drugs, contain primary amine functional groups. These include catecholamine NTs (*e.g.*, dopamine (DA)), various substituted phenylethylamines (*e.g.*, amphetamine), AAs, and neuropeptides. In most cases, mass spectrometry (ESI and MALDI) analysis of trace amounts of such compounds is challenging because of their poor ionization characteristics.^522^ 2,4-Diphenylpyridinium (DPP) was first tested as a reactive matrix targeting DA, a neuroactive substance with a primary amine functional group, and was used to map the distribution of DA in brain tissue slices.^522^ Shariatgorji et al. discovered that pyridinium salts can serve as MALDI-MS imaging matrices for bioactive primary amines. They evaluated three different pyridinium salts (DPP, 1,4-phenylene-4,4′-bis­(2,6-diphenyl-4-pyrylium) (PBDPP), and 2,4,6-trimethylpyrylium (TMP)) as derivatization agents. The study found that DPP effectively derivatizes primary amines and can be used as a reactive MALDI matrix. Using this approach, the distribution of DA and phenylethylamine in brain tissue slices was mapped, and the distribution of the neurotoxin β-*N*-methylamino-l-alanine was also quantitatively plotted (Figure 41).^522^ Pyridinium derivatives have since been used in many protocols, although additional matrices are sometimes required to achieve sufficiently high ionization efficiency.^523^ Recently, 2-fluoro-1-methyl pyridinium (FMP) has been employed for the detection of NTs containing amine and hydroxyl groups.^165^ Shariatgorji et al. synthesized 10 polyphenylated FMP compounds and evaluated their performance as MALDI-MS reactive matrices for analyzing small molecules with phenolic hydroxyl, primary amine, and secondary amine groups.^165^ The designed matrices consist of two domains: the fluoropyridine molecule reacts with primary amines, secondary amines, and hydroxyl groups through nucleophilic aromatic substitution, while the expanded conjugated system of the anthracene unit provides strong absorption properties. The fluoromethylpyridine-based reactive matrices enable covalent charge labeling of molecules containing phenolic hydroxyl, primary, and secondary amines, including NTs such as DA and serotonin, as well as their metabolites. This approach improves the detection limit of MALDI-MSI for low-abundance NTs and allows the synchronous imaging of NTs in the fine structures of the brain with 10 μm resolution (Figure 42).

**Figure 41:**
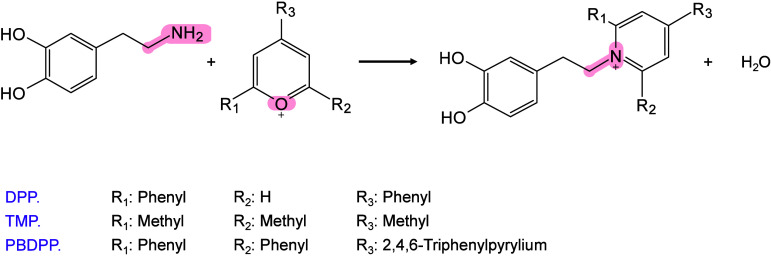
Reaction of dopamine with pyrylium salts.

**Figure 42:**
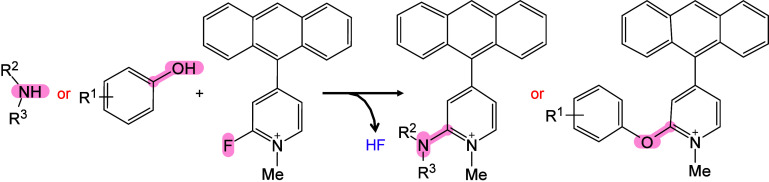
Schematic of the reaction between the FMP reactive matrix and phenolic hydroxyls and amines. R^1^, R^2^, and R^3^ denote variable substituents.

Pyridinium salts readily undergo specific reactions with primary amines to form pyridinium cations. This property has been exploited to increase the sensitivity of MS analyses for various proteins and small molecules.^519–521^ Matveeva et al. reported that 1-pyrenylboronic acid (1-PBA) has the potential to serve as a MALDI-MS reactive matrix, effectively analyzing α-hydroxy acids and various compounds containing 1,2-diols, 1,2-amines, and 1,2-diamines. They successfully detected molecular free-radical cations formed from phenylboronic acid salts of these compounds, even in the absence of conventional matrices (Figures 43 and 44).^524^ Addy et al. reported that label-assisted LDI-MS (LA-LDI-MS) is a rapid technique for detecting *cis*-1,2-diols with 1-PBA as a selective probe. When reacting with boronic acid, both *cis*-1,2-diols and *cis*-1,3-diols form stable cyclic boronate esters (Figure 45).^525^ 4-(*N*-methyl) pyridinium boronic acid (4-(*N*-Me)­Py^+^B­(OH)_2_) is a reactive matrix used for the selective derivatization of catecholamines to enable their sensitive detection. The boronic acid functionality condenses with the diol functional group of catechols, while the permanent charge on the pyridinium ion increases the ionization efficiency of the target analytes (Figure 46).^166^ Monopoli et al. explored the chemistry of boronic acids in MALDI-MS and synthesized a reactive matrix-boronic acid analog of CHCA-with molecular recognition capabilities. This matrix selectively recognizes vic-diols, α-hydroxy acids, and amino alcohols, and its use enabled the first MALDI-MS detection of anions such as fluoride.^490^ To this end, 4-formylphenylboronic acid (FPhBA) was condensed with cyanoacetic acid to yield a novel MALDI matrix ([(*E*)-4-(2-cyano-2-carboxyethenyl)­phenyl]­boronic acid (CCPBA)), which serves as a molecular recognition probe for the selective determination of LMW compounds (Figure 47). Chen et al. developed a reactive matrix for the *in situ* chemical derivatization and specific detection of *cis*-diol compounds by MALDI-MS.^526^ In this method, a 6-borono-1-methylquinoline-1-ium (BMQI) reactive matrix was designed for the *in situ* derivatization of *cis*-diol compounds based on the boronate affinity interaction between boronic acid and *cis*-diol groups. Compared with traditional commercial matrices and other boronic acid reagents, BMQI can significantly accelerate the D/I process, improve reproducibility, eliminate background interference, and increase signal intensity in the analysis of various *cis*-diol compounds, even at concentrations as low as 1 nmol. BMQI-assisted LDI-MS was successfully applied to the rapid screening and identification of sugar alcohols in different sugar-free foods. This work provides an alternative to the MALDI-MS analysis of *cis*-diol-containing molecules, and the method can be extended to other food samples and biofluids. In general, the probes used for primary amine compound detection are all pyrene-based scaffolds, and despite being good laser-active chromophores, they have both reactivity and solubility problems (Figure 48). Cheema et al. used *p*-methoxy cinnamaldehyde (PMC) in LA-LDI-MS for the detection of different primary amines, AAs, and NTs.^527^ Inspired by the chromophoric nature, these scholars performed a systematic study to design and optimize cinnamaldehyde derivatives and found that PMC is simple, cost-effective, and less hydrophobic laser-active label that can detect a series of primary amines, including NTs and AAs. The label PMC can also discriminate between aliphatic and aromatic primary amines.

**Figure 43:**
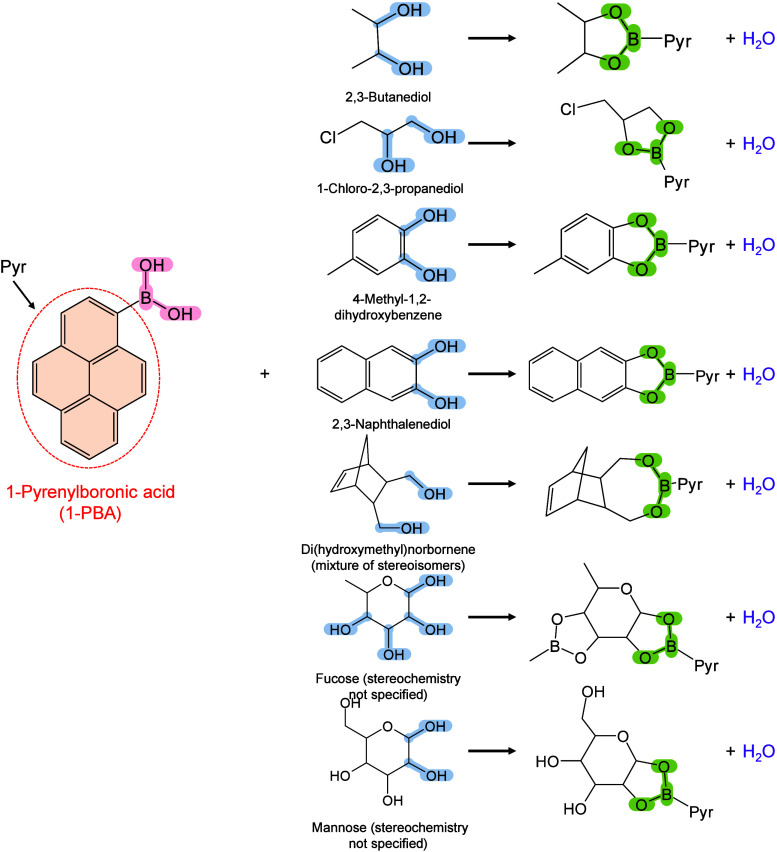
Studied diols and detected products of their interaction with 1-PBA.

**Figure 44:**
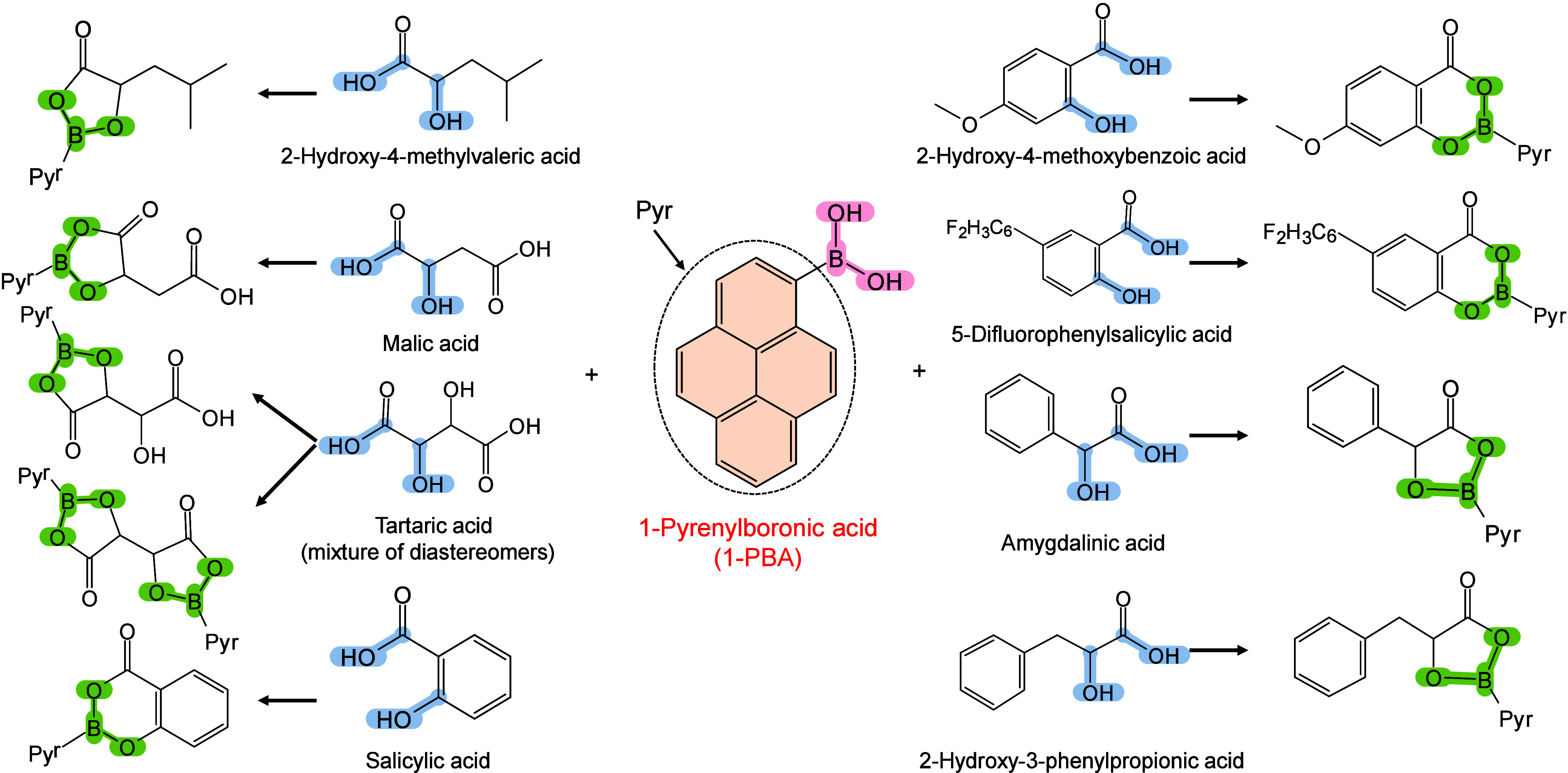
Studied α-hydroxy acids and detected products of their derivatization with 1-PBA.

**Figure 45:**
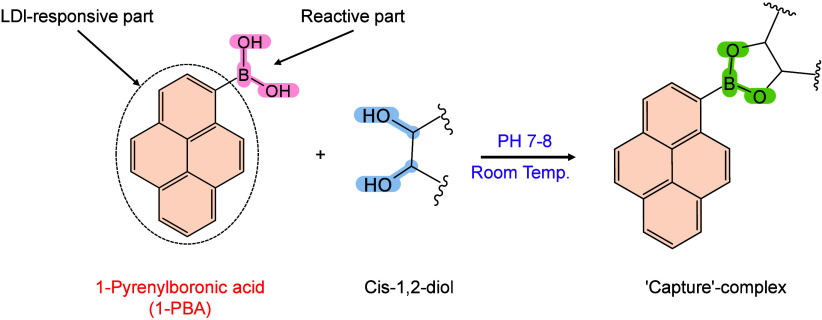
Pyrene boronic acid and expected complexation behavior with *cis*-1,2-diols.

**Figure 46:**
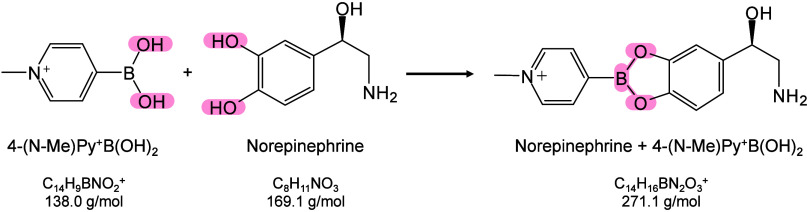
Reaction scheme for the derivatization of norepinephrine with 4-(*N*-methyl)­pyridinium boronic acid (4-(*N*-Me)­Py^+^B­(OH)_2_) which yields the corresponding boronate ester via a boronic acid reaction with the catechol group.

**Figure 47:**
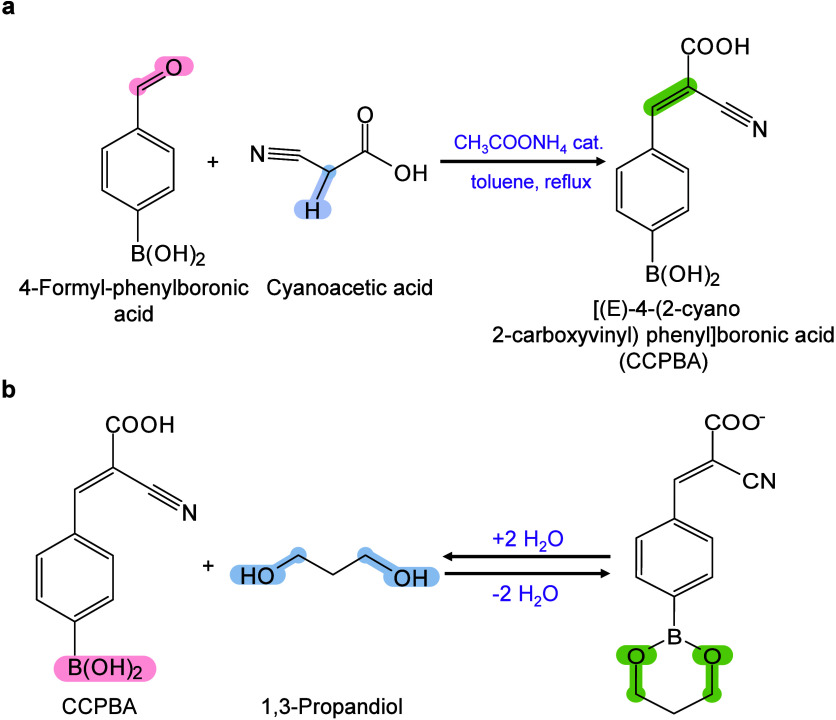
(a) Synthesis of MALDI matrix CCPBA. (b) Scheme of reaction between CCPBA and 1,3-propandiol.

**Figure 48:**
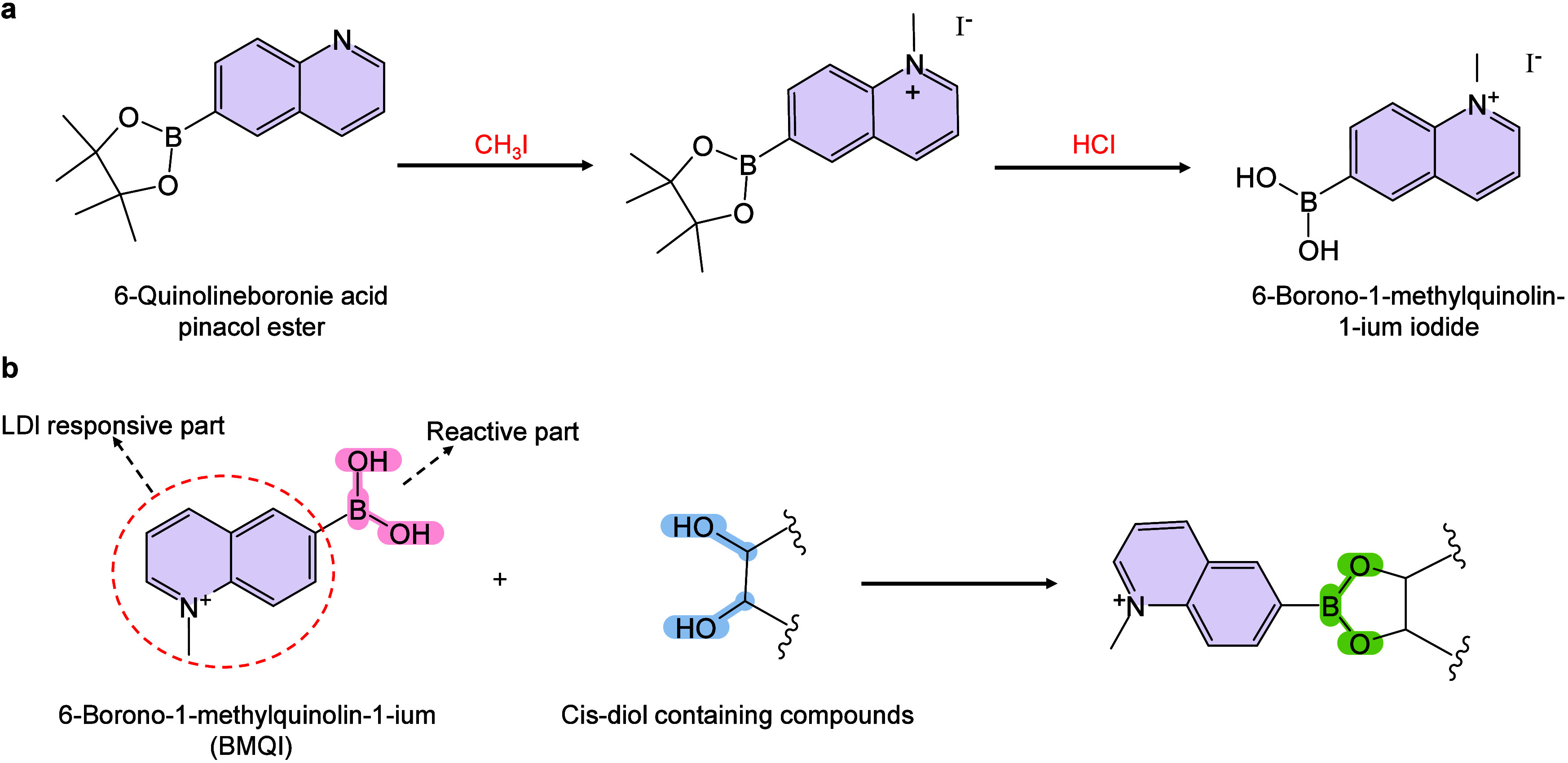
(a) Synthesis scheme of BMQI. (b) Expected complexation behavior of BMQI with *cis*-diol compounds.

The novel reagent 4-trimethylamino-6-(4-methoxy-1-naphthyl)-1,3,5-triazine-2-(3-aminophenylboronic acid (TMNTA) possesses aromatic rings within its molecular structure. Glucose derivatized with TMNTA, in the absence of any additional MALDI matrix, produced distinct mass spectral peaks corresponding to the derivatized products, while the background spectra remained clean and devoid of interference. Qin et al. utilized a boric acid-based reaction matrix for the rapid absolute quantification of glucose and fructose isomers in honey by MALDI-TOF/TOF MS.^528^ This finding suggests a significant enhancement in the ionization efficiency of glucose following derivatization, with TMNTA functioning effectively as a matrix, thereby simplifying the experimental procedure (Figure 49). This study presents the development of a reactive matrix-assisted MALDI-MS technique aimed at the rapid absolute quantification of glucose and fructose isomers in honey. The TMNTA reactive matrix demonstrated exceptional effectiveness, markedly enhancing the ionization efficiency of monosaccharide isomers while simultaneously eliminating matrix interference and simplifying the sample preparation process. Glucose and fructose were identified through the analysis of two diagnostic ions produced following derivatization with TMNTA. Furthermore, the absolute quantification of glucose and fructose in binary mixtures was achieved by integrating the results from MALDI-TOF MS and MALDI-TOF/TOF MS/MS. Finally, the concentrations of glucose and fructose in honey samples were accurately determined following a simple dilution process. Consequently, this study introduces a simple, rapid, accurate, and high-throughput method for the quantification of glucose and fructose in honey, with significant implications for advancing the applications of MALDI-MS in quantitative analysis.

**Figure 49:**
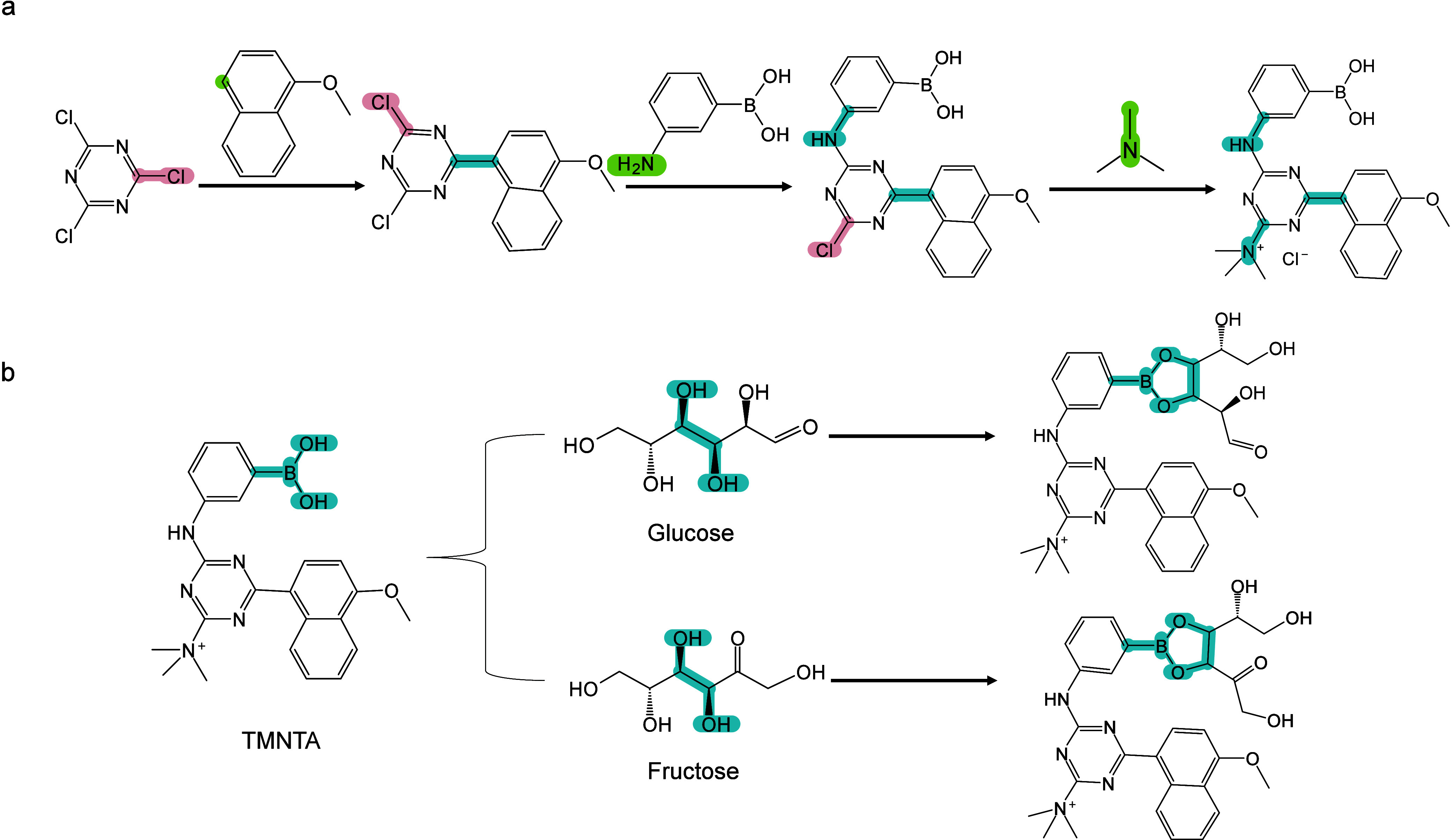
(a) Synthesis of TMNTA and (b) reaction scheme for the derivatization of glucose and fructose with TMNTA.

###### 3.1.2.1.4 Addition Reaction

The C=C DB of alkenes can react with carbonyl groups through the Paternò–Büchi (PB) reaction, a 2+2 photochemical cycloaddition that produces oxetane (four-membered heterocyclic) derivatives. BPh derivatives undergo the PB reaction and exhibit excellent absorption properties, which are required for MALDI-MS. Heiles’ group utilized this method for tissue derivatization and the detection of unsaturated PLs,^529^ and they detected 12 lipid classes in mouse kidney and pancreatic tissues using MALDI-MSI.^530^ Waldchen et al. employed a PB reaction matrix, BPh, which has intrinsic photoactivity in MALDI-MSI to enable the precise localization of DBs in isomeric PLs.^529^ BPh not only facilitated desorption and ionization but also functioned as a derivatizing agent, performing laser-driven PB reactions to functionalize unsaturated PLs without the need for additional equipment and generating PB product ions for various PL classes. The results showed that the use of BPh improved the lateral resolution of DB isomers, potentially revealing its impact on PL metabolism (Figure 50). Waldchen et al. achieved high-resolution dual-polarity MS imaging and lipid C=C position-resolved MS^2^ imaging using multifunctional reactive MALDI matrices.^530^ The analytical capabilities of 2-benzoylpyridine (BzPy) as a multifunctional MALDI-MSI matrix are demonstrated by imaging endogenous and PB-functionalized lipids in mouse kidney sections with 7 μm lateral resolution in both ion modes. Tracking diagnostic lipid DB-position fragment ions in mouse pancreatic tissue with down to 10 μm pixel size allows us to identify the islets of Langerhans associated with lipid isomer upregulation and depletion. Additionally, Asakawa and Osaka utilized thiosalicylic acid (TSA) as a reactive matrix to successfully and achieved the direct MALDI-MS analysis of disulfide bonds in peptides.^491^ Although TSA possesses reducing properties, its interaction with the carboxyl oxygen of peptides is weak, thus avoiding the attenuation caused by reduction of the MALDI source. During the MALDI analysis, TSA partially reacted with peptides containing disulfide bonds, leading to cleavage of the disulfide bonds and the formation of TSA-adducted peptides, allowing the number of disulfide bonds in the peptides to be calculated by comparing mass spectra data from different matrices (Figure 51).

**Figure 50:**
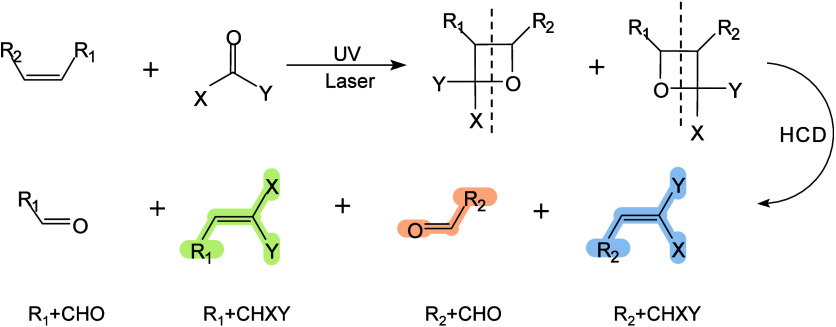
Paternò–Büchi functionalization and higher collisional energy dissociation of functionalized lipids.

**Figure 51:**
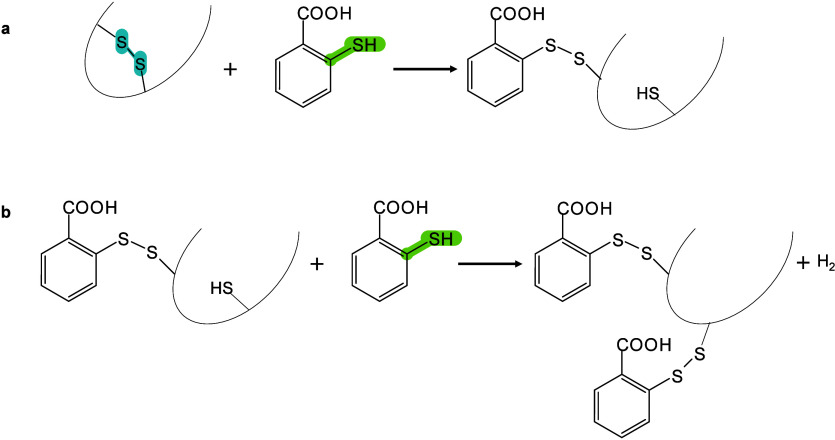
Reactions between peptides containing disulfide bonds and TSA. (a) Disulfide bond cleavage by the with TSA and (b) the production of TSA-modified cysteine residues from free cysteine residues and TSA.

###### 3.1.2.1.5 Electron Transfer Reaction

Protonation, deprotonation, and adduct formation reactions are common in MALDI-MS, but not all organic compounds can acquire a charge through these processes.^64^ Electron transfer is a less commonly used method, but it has the potential to expand the applicability of MALDI-MS, as it can ionize compounds that are crucial in material science, such as polymers, fullerenes, PAHs, organometallics, and coordination complexes.^531,532^ Considering that the proton affinity and ionization energy of the matrix can influence energy transfer during electron transfer processes, a potential strategy to avoid metal loss is to use electron transfer matrices that form radical cations.^533^ While several common matrices are capable of forming radicals, they tend to favor competitive proton transfer rather than electron transfer. The most common secondary ionization mechanism in positive ion mode MALDI involves proton transfer reactions, making peptides and proteins with basic (and acidic) sites prone to proton transfer. However, nonpolar, passivated compounds lacking basic sites are difficult to protonate, making it challenging to generate charged species of these analytes using traditional MALDI matrices. In such cases, electron transfer to form radical molecular ions is a viable alternative, and some matrices may facilitate this process.^534^ Studies on electron transfer matrices have described the use of various organic compounds, including 2-[(2*E*)-3-(4-*tert*-butylphenyl)-2-methylprop-2-enyl]­propanedinitrile (DCTB), 9,10-diphenylanthracene (9,10-DPA), poly­(3-octylthiophene-2,5-diyl) (P3OT), 7,7,8,8-tetracyanoquinodimethane (TCNQ), tetrathiafulvalene (TTF), DAN, phenylenevinylene (PV), and 1-amino-2,4-dichloronaphthalene (ADCN) for MALDI-MS analysis of coordination compounds^535^, conjugated polymers^536^, and polycyclic aromatic hydrocarbons (PAHs), *etc.* (Figure 52).

**Figure 52:**
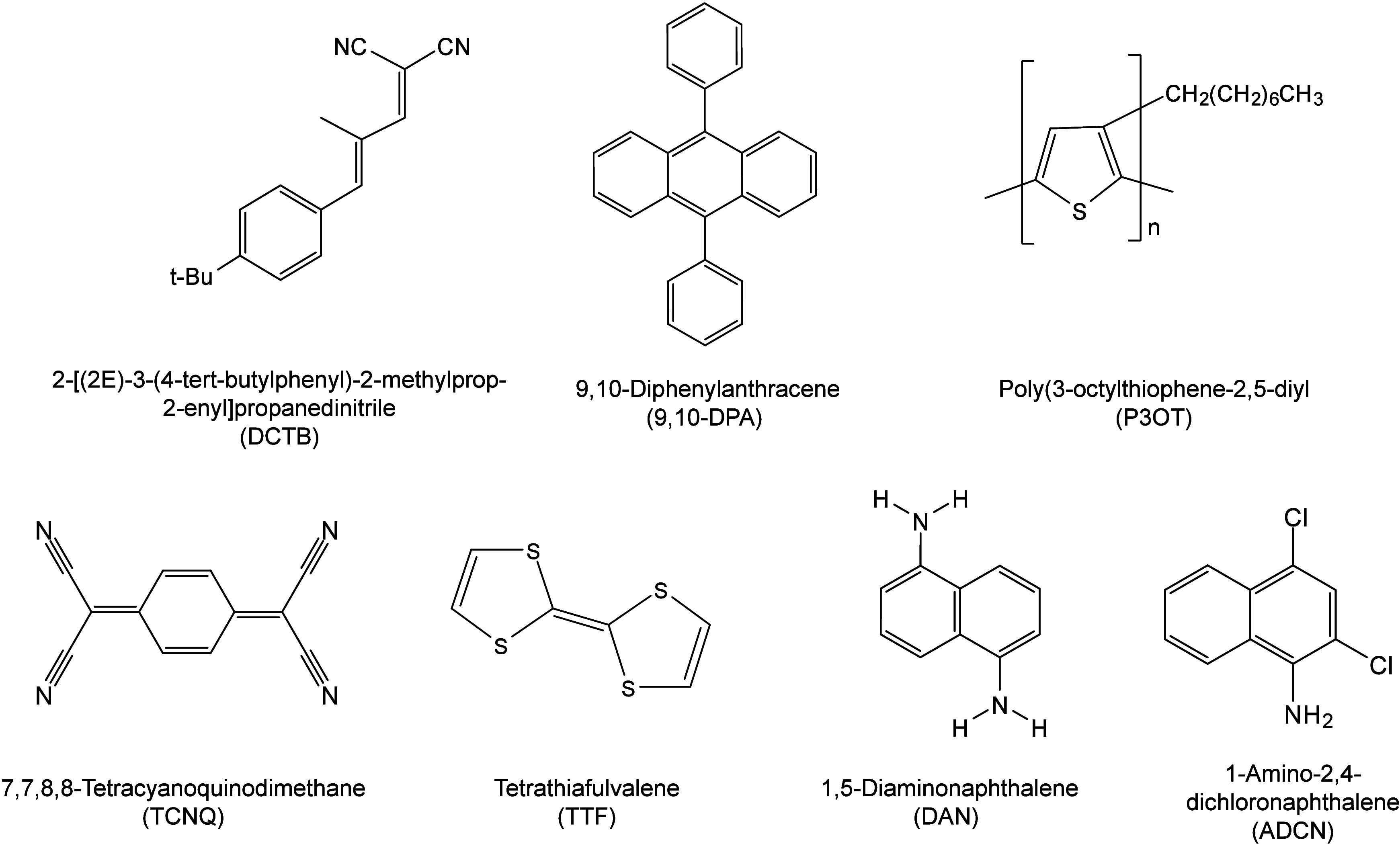
Chemical structures of electron transfer matrices.

Ulmer et al. used DCTB as a matrix for MALDI-MS and found that it exhibited excellent D/I properties for highly unstable compounds.^537^ Their results indicated that DCTB primarily acts as an electron transfer agent, providing a very low ionization threshold, with the generated ions being either free radical anions or cations. Wyatt et al. also employed the DCTB matrix for MALDI-MS characterization of various analytes, and found that DCTB is a nonpolar, nonprotonated matrix, suitable for a wide range of compounds including coordination compounds, organometallics, conjugated organic compounds (such as porphyrins and phthalocyanines), carbohydrates, calixarene derivatives, and macrocycles. DCTB outperformed traditional polar acidic matrices and required significantly lower laser fluence for D/I.^535^

9,10-DPA is a material used in organic light-emitting diodes and can also serve as an electron transfer reagent for electron transfer dissociation reactions.^538^ Boutaghou and Cole reported that 9,10-DPA, as an electron transfer matrix for MALDI-MS, can efficiently promote ionization, and its high photon absorption capacity at low laser intensities allows the detection of intense radical cations with minimal laser impact.^539^ Radical molecular ions of chlorophyll (a), retinol and several other compounds were successfully detected in positive ion MALDI experiments employing this new matrix, whereas buckminsterfullerene gave a high yield of (A^–^) in the negative ion mode.

Ijuin et al. discovered that in negative ion mode MALDI-MS, 1,2-dioxetanes can undergo electron transfer-induced fragmentation, leading to intramolecular charge transfer-induced chemiluminescence (CTICL).^540^ To elucidate the underlying mechanism, they attempted to ionize phenol-containing dioxetanes and found that P3OT is a promising negative ion mode MALDI matrix. By substituting hydroxyl groups with aromatic groups, these groups act as antennas that capture electrons from the matrix, enabling electron transfer ionization in negative ion mode MALDI for the first time.

TCNQ is widely used in the field of organic metals. Known for its strong electron-accepting properties, TCNQ exhibits a pronounced absorption peak at 337 nm, making it a promising candidate as an electron acceptor matrix. Przybilla et al. has demonstrated TCNQ's efficacy as a novel matrix in MALDI-MS analysis, particularly for the detection of insoluble giant PAHs.^541^ TCNQ facilitates the desorption of PAHs and supports their ionization by promoting the formation of radical cations, yielding significant advantages in terms of mass spectral quality, S/N, isotopic resolution, and point-to-point reproducibility.

Asakawa et al. used the electron-donating compound TTF as a matrix for the negative ion mode MALDI-MS analysis of industrial pigments.^542^ Due to the electron-donating nature of TTF, analytes were readily detected as radical anions. Compared with positive ion mode MALDI analysis using electron-accepting matrices such as TCNQ, TTF generated stronger molecular anions and fewer fragment ions, offering a useful complementary approach for MALDI analysis in positive ion mode.

Molin et al. reported that DAN, as a MALDI matrix, exhibits the dual properties of protonation and reduction, enabling effective in-source fragmentation in positive ion mode. This is particularly useful in the analysis of proteins containing disulfide bonds, where it promotes the reduction process and leads to the cleavage of disulfide linkages. Its reducing properties also exhibit radical characteristics.^543^ Calvano et al. utilized DAN as an electron transfer secondary reaction matrix and significantly improved the identification of chlorophyll in food via MALDI-MS. DAN not only prevented the demetallation of chlorophyll but also successfully inhibited the fragmentation of plant ester linkages, allowing the first analysis of intact chlorophyll.^544^ Additionally, they investigated the use of DAN as a matrix for bacterial chlorophyll a and its zinc and copper analogs and reported that its ionization efficiency surpassed that of other common electron transfer matrices, with minimal fragmentation.^545^

The structural flexibility of PV oligomer systems endows them with broad potential in photophysical applications. The photophysical properties and solubility of compounds can be precisely tuned by modifying the length of the electronic conjugation and the chemical nature of the substituents. Castellanos-García et al. discovered that PV derivatives, as UV-MALDI electron transfer matrices, can form radical cations at lower laser powers. Furthermore, the electron transfer process significantly enhances both the matrix and analyte signal intensities, while improving the resolution and sensitivity (Figure 53).^533^

**Figure 53:**
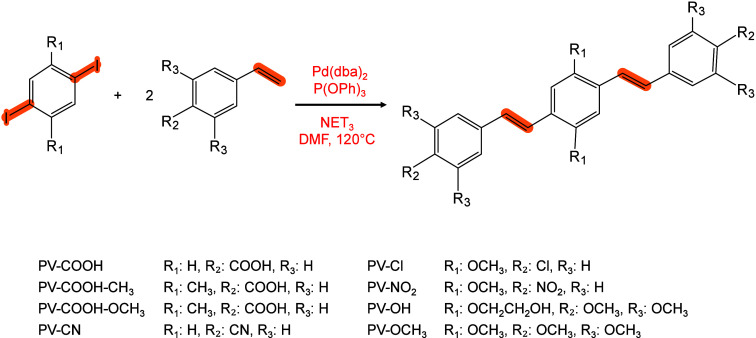
General procedure for PV synthesis using the Mizoroki–Heck cross coupling reaction.

A matrix assisted laser desorption and photoinduced electron transfer ionization mass spectrometric imaging (MALDI^ET^-MSI) technique has been developed as a spatial metabolomic tool to investigate the missing part of the redox network.^546^ It is based on photo active matrix materials such as 1-amino-2,4-dichloronaphthalene (ADCN) that contain structural units for the absorption of laser irradiation and generate electrons for the ionization of redox active species through electron transfer. Although this matrix possesses amino groups, it does not generate protonated cations but instead forms cationic radicals upon electron loss. This indicates that the matrix cannot accept protons and thus cannot deprotonate the sample molecules. In negative ion mode, after capturing electrons, anion radicals undergo selective α-position chemical bond cleavage to produce fragment ions. Using ADCN matrix, mass spectrometry imaging of breast cancer tissue sections via photoelectron transfer can detect negative ions formed when fatty acids lose protons. MALDI^ET^-MSI not only reveals metabolic acids in the tricarboxylic acid (TCA) cycle but also localizes various redox active metabolites that have been underexplored. It provides high spatial resolution that can differentiate the cancer region, adjacent region, and normal tissue region.

Macha et al. applied nonpolar matrices such as anthracene, pyrene, and acenaphthene for the MALDI-MS analysis of LMW nonpolar polymers (*e.g.*, polybutadiene, polyisoprene, and polystyrene). Due to their lower ionization energies and higher ultraviolet molar absorption coefficients, these matrices facilitate the efficient generation of radical molecular cations in MALDI.^547^ McCarley et al. demonstrated that terthiophene and anthracene-*d*_10_ serve as effective matrices for MALDI-MS analysis of three metallocenes (*i.e.*, 1,2-diferrocenylethane, ferrocene, and decamethylferrocene) and one methylene-bridged bisphenol compound of 2,2′-methylenebis-(6-*tert*-butyl-4-methylphenol).^534^ Notably, the mass spectra of these matrix/analyte combinations revealed no formation of protonated molecules; instead, each analyte exclusively formed molecular radical cations (A^+•^) when either matrix was used. This finding indicates that these matrices facilitate electron-transfer ionization, diverging from the conventional proton-transfer mechanism.

###### 3.1.2.1.6 Special Chemical Reactions

In addition to commonly used matrix-assisted derivatization reactions, some specialized reactive matrices have been employed to enhance the detection capabilities of MALDI-MS. Sugar amines exhibit proton affinity and accelerate hydrolysis via an acrylic imine intermediate under mild acidic conditions through a general acid-catalyzed mechanism. Ma et al. utilized MALDI-MSI technology to design and synthesize a novel reactive matrix, glycosyl-3-aminoquinoline (Gly-3AQ), for the acidity imaging of pomelo.^548^ Under acidic catalysis, this matrix undergoes hydrolysis and is sensitive to changes in pH, with the peak intensity of the hydrolysis product, 3-AQ, increasing as the pH decreases. Gly-3AQ demonstrated excellent acid responsiveness and selectivity in real sample analysis, indicating its potential for *in situ* acidity MSI applications in the biomedical field. Slyundina et al. discovered novel reaction matrices for MALDI-MS analysis of alcohols.^549^ A possibility of using a number of aromatic and heteroaromatic carboxylic acids and their halogen anhydrides as reactive matrix compounds for the analysis of alcohols of different structures by MALDI-MS has been studied. It is shown that the acylation of alcohols with nicotinic acid chlorides (NAC) and quinoline-6-carboxylic acid chlorides (quinoline-6CAC) gives derivatives with high D/I efficiency under MALDI conditions, and that the free acids formed as a result of the hydrolysis of anhydrides act as matrix compounds. The proposed approach is tested on a number of aliphatic, alicyclic, and aromatic alcohols. Furthermore, Fulop et al. developed a novel reactive matrix, (*E*)-2-cyano-*N*-(2-(2,5-dioxo-2,5-dihydro-1*H*-pyrrol-1-yl)­ethyl)-3-(4-hydroxyphenyl)­acrylamide (CHC-Mal), for the selective detection of free thiol groups in metabolites and proteins by MALDI-MS.^550^ CHC-Mal effectively derivatized reduced proteins and aided in the detection of thiol-containing metabolites in tissues (Figure 54).

**Figure 54:**
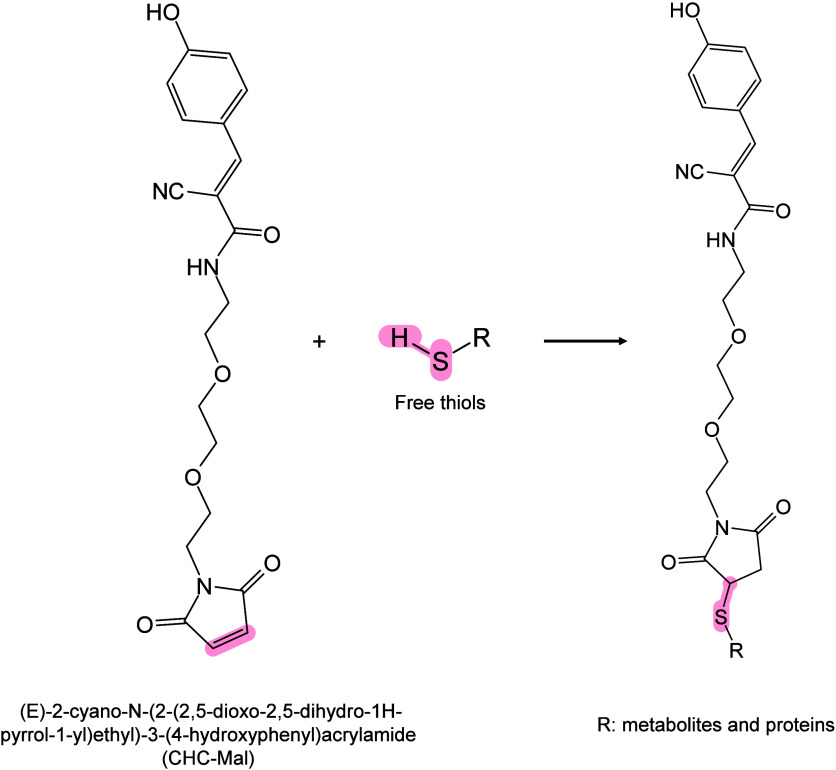
CHC-Mal derivatization of free thiol groups.

###### 3.1.2.1.7 Summary

By introducing reactive matrices that can engage in specific chemical interactions with analytes, this technique integrates traditional MALDI with chemical reactions, significantly enhancing the sensitivity and selectivity of the analysis.^64^ The fundamental principle is that reactive components facilitate ionization of the analytes during sample preparation or laser irradiation, thereby reducing matrix background interference and improving the quality of the MALDI-MS signal.^551^ This technology has demonstrated great potential in the analysis of biomacromolecules, small molecule detection, and disease biomarker research, particularly in the identification and quantification of proteins, peptides, carbohydrates, and nucleic acids, as well as in the analysis of drug metabolites, environmental pollutants, and metabolites.^529,552^ However, reactive MALDI matrices face challenges, such as the complexity of matrix selection and optimization, interference from reaction byproducts, and issues related to widespread adoption and standardization.^175^ With advances in chemical synthesis and materials science, the development of novel reactive matrices is expected to further drive the progress of this technology and expand its applications in clinical diagnostics, food safety, and environmental monitoring. By integration with other MS techniques (*e.g.*, ESI-MS, liquid chromatography (LC)-MS), reactive MALDI matrices can be used to achieve more efficient and precise analyses, providing new solutions for the efficient detection of complex samples.

##### 3.1.2.2 Matrix Derivatization

The rational design of novel MALDI matrices provides a compelling approach to achieving specific physicochemical properties by strategically modifying the structures of classical matrices. This approach signifies a progressive transition toward a rational matrix screening methodology that was previously based on empirical observations. Jaskolla et al. were the first to pioneer the field of rationally designed matrices, synthesizing derivatives of CHCA and DHB, which were used to tune the proton affinity and improve the ionization efficiency of the analytes.^54^ Matrix derivatization can be achieved through a variety of chemical reactions, including esterification, etherification, allylation, and other modifications to enhance signal intensity and reduce background interference.^553^ The objective of derivatization is to enhance interactions between the matrix and the sample, facilitate the desorption and ionization of sample molecules, and augment the MS signal intensity, thereby improving the sensitivity and reliability of the analysis.^162^ To date, cinnamic acid derivatives, benzoic acid derivatives, and other types of MALDI matrices have been rationally designed through the application of derivatization strategies.

###### 3.1.2.2.1 Cinnamic Acid Derivatives

A considerable number of the matrices currently in use, such as CHCA, were discovered empirically by researchers during the early period of MALDI analysis.^350^ In recent years, the development of various cinnamic acid derivatives has facilitated the practical application of MALDI in bioanalysis. These derivatives, when used as MALDI matrices, exhibit favorable ionization characteristics and minimal background noise, and are suitable for the analysis of both micro- and macromolecular biological samples. Consequently, they show considerable promise for use in protein, nucleic acid, and drug analysis. Specific cinnamic acid derivatives include α-cyano-4-hydroxycinnamic methyl ester (CHCE), (*E*)-α-cyano-4-hydroxycinnamic acid propyl ester (CHCA-C3), 4-chloro α-cyanocinnamic acid (ClCCA), (2*E*)-3-(anthracen-9-yl)-2-cyano-propan-2-enoic acid (AnCCA), (*E*)-2-cyano-3-(naphthalen-2-yl)­acrylic acid (CNAA), cyanonitrobenzene diacetic acid (CNDA), α-cyano-2,4-difluorocinnamic acid (Di-FCCA), α-cyano-5-phenyl-2,4-pentadienoic acid (CPPA), D^4^-α-cyano-4-hydroxycinnamic acid (D^4^-CHCA), 4-phenyl-α-cyano-cinnamic acid amide (Ph-CCA-NH_2_), *p*-phenyl-α-cyanocinnamic acid amide (p-Ph-CCAA), 4-aminocinnamic acid (ACA), and 4-(dimethylamino)­cinnamic acid (DMACA) (Figure 55).

**Figure 55:**
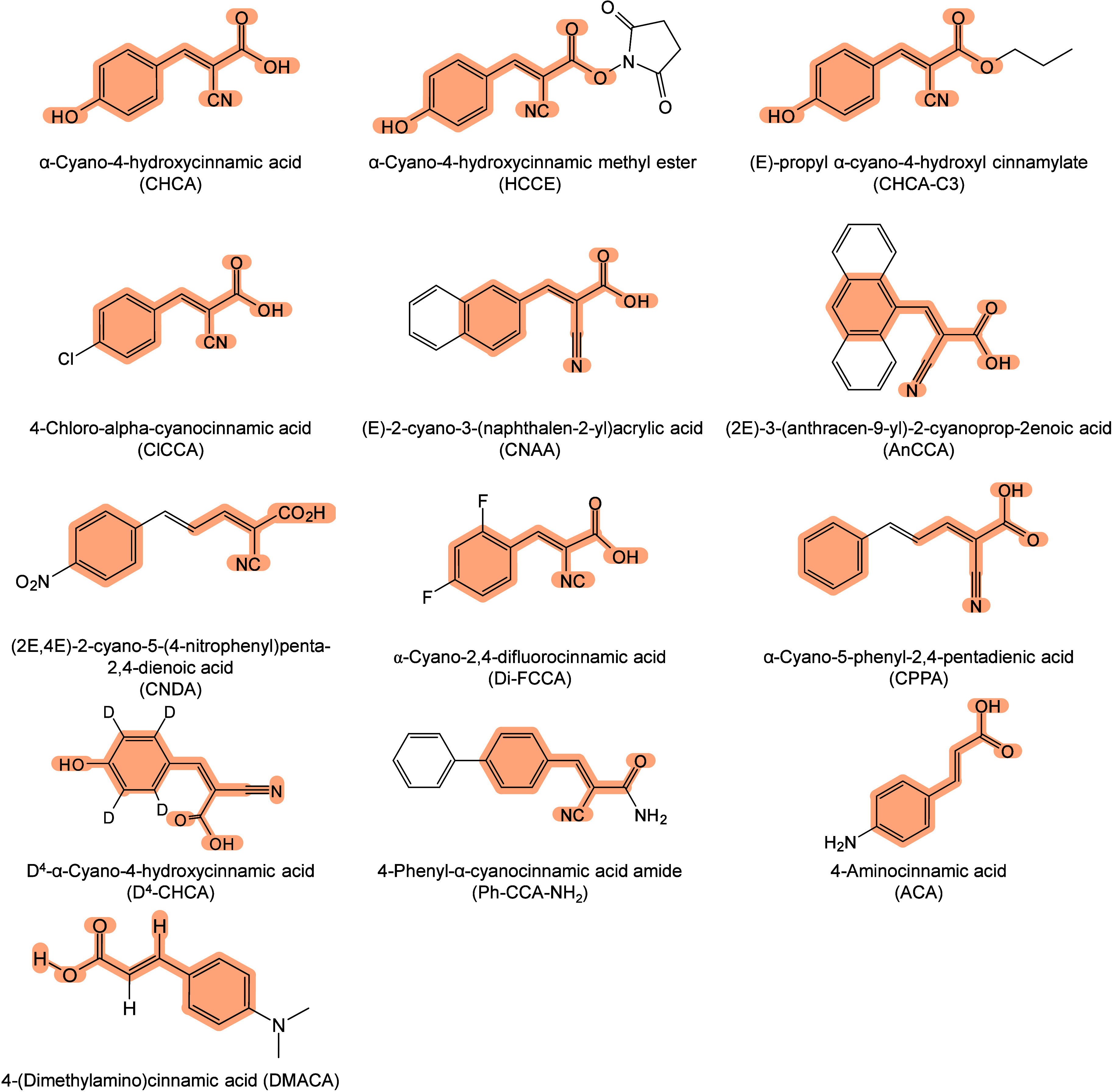
Rational design strategies for cinnamic acid derivatives.

Both CHCE and CHCA-C3 are members of the CHCA family of compounds. Lascoux et al. achieved an enhancement in the signal intensity of proteins detected using MALDI-MS by combining lysine residue labeling with the neutral matrix CHCE.^554^ Peptides labeled with CHCE displayed a greater signal intensity than the unlabeled peptides, despite the former being present in lower abundance. This approach facilitates specific labeling and mitigates interference with the protein structure. Furthermore, the analysis of low-abundance samples and the issue of salt interference have constituted significant challenges in the field of protein analysis by MALDI-MS. Wang et al. demonstrated that CHCA-C3 is a highly sensitive, salt-resistant matrix that is suitable for the analysis of intact proteins by MALDI-MS.^555^ The modification of CHCA with a hydrophobic alkyl group on the carboxyl group resulted in a 10-fold increase in detection sensitivity compared to conventional matrices. In addition, CHCA-C3 showed the ability to effectively detect myoglobin signals in the presence of urea, NH_4_HCO_3_, and KH_2_PO_4_. Meanwhile, CHCA-C3 exhibited strong washability, thereby demonstrating considerable promise for protein analysis.

ClCCA is an advanced MALDI matrix that was selected and synthesized by systematically and purposefully altering the functional groups of the core unit of CHCA. ClCCA exhibited excellent matrix properties and significantly improved the sensitivity and recovery of basic peptides by MALDI-MS.^54^ Furthermore, ClCCA also demonstrated a reduced matrix proton affinity in the detection of phosphatidylethanolamine chloramine, thereby enhancing the protonation efficiency of the analyte and offering enhanced performance compared to existing matrices.^556^ The MALDI-MS analysis of sialylated glycans and glycopeptides using the ClCCA matrix, as described by Selman et al, revealed high sensitivity to intact and soluble glycosylated glycopeptides in negative ion mode.^281^ In comparison to CHCA, ClCCA has been shown to enhance the protonation efficiency of analytes because of its lower proton affinity. In addition, the microcrystalline dots produced by mixing ClCCA with the sample were suitable for automated detection while maintaining superior resolution and mass accuracy. Calvano and coworkers demonstrated that ClCCA represented an efficient soft ionization matrix for detecting yanocobalamin (CNCbl) in foodstuffs in positive ion mode by MALDI-MS.^557^ Moreover, Ventura et al. successfully analyzed cyanocobalamin conjugates of cisplatin and diaminocyclohexane-platinum­(II) using ClCCA, thereby proving the effectiveness of ClCCA in the detection of CNCbl-Pt­(II) conjugates.^558^ In conclusion, their results indicated that ClCCA is an appropriate matrix that allows intact CNCbl to produce the protonated adduct [M+H]^+^.

Two novel α-cyanocinnamic acid derivatives, CNAA and AnCCA, were used as alternative matrices for the analysis of LMW compounds by UV-MALDI-MS/MS, superseding the use of CHCA.^559^ Together with the commonly used CHCA and ClCCA, these two matrices form a series of matrices with similar chemical properties but different spectral interference ranges with tunable background interference signals and enhanced analytical responses. Novel CHCA-derived substrates were synthesized for the MALDI-MS analysis of lipids by Monopoli et al., who reported that the size of the aromatic system and the effect of the substituent group significantly affect the ionization and crystal properties.^264^ In comparison with CHCA and ClCCA, CNDA, (*E*)-2-cyano-3-(6-methoxynaphthalen-2-yl)­acrylic acid, and CNAA exhibited favorable ionization characteristics, interference-free spectra, superior S/N ratios, and reproducibility as MALDI matrices. In particular, CNAA was identified as an optimal matrix in positive ion mode, whereas CNDA demonstrated suitability for the analysis of neutral lipids, including diacylglycerols (DAGs) and TAGs.

Teuber et al. compared a variety of matrices, including DHB, 9-AA, PNA, 2-MBT, and AAN. In addition, they introduced a newly synthesized matrix, Di-FCCA, which significantly enhanced the sensitivity of the MALDI-MS analysis of lipids in egg yolks through matrix optimization. These findings indicated that Di-FCCA is the optimal matrix for the identification of lipids in positive ion mode, with a sensitivity enhancement of more than an order of magnitude.^560^ However, it is not suitable for the detection of PLs in negative ion mode.

Monopoli and colleagues synthesized a CPPA matrix and employed it for MALDI-MS analysis of intact proteins. Their findings indicated that the utilization of CPPA markedly enhances protein signals, reduces spot-to-spot variations, and improves homogeneity. In comparison with conventional matrices (*e.g.*, SA, CHCA), CPPA exhibited a superior S/N ratio and a more homogeneous response in protein detection. Furthermore, high sensitivity and resolution with minimal fragmentation can be attained even at low laser energies.^561^

Shariatgorji et al. demonstrated that the deuterated MALDI matrix D^4^-CHCA was capable of revealing masked MS signals, thereby enhancing the analysis of small-molecule drugs and endogenous compounds.^562^ The use of D^4^-CHCA resulted in shifts of +4, +8, and +12 Da in the cluster and fragment peaks of the conventional matrix CHCA, facilitating the revelation of previously masked low-mass signals. Furthermore, the matrix retained the physicochemical properties (acidic and light absorption properties) of CHCA, providing an effective new method for the analysis of small-molecule compounds.

Fulop et al. successfully revealed the suitability of Ph-CCA-NH_2_ as a matrix for MALDI-MS analysis of a variety of lipids in negative ion mode. The replacement of the carboxyl group with a neutral amide effectively reduced the impact of matrix acid–base chemistry on sensitive compounds, thereby enhancing the analytical reliability.^563^ The introduction of different amide substituents offers the potential for modifying the hydrophobicity of compounds without affecting the conjugated system for UV absorption during the MALDI process. The new matrix not only enhanced the sensitivity and reproducibility of the analysis, but also decreased the background peaks and improved matrix signal suppression, thereby offering a more robust performance than that of traditional lipid matrices.

The objective of the study conducted by Tambe and colleagues was to examine the structure–property relationship between phenyl-α-cinnamic acid derivatives and sulfides. To this end, they constructed a library of 59 structurally analogous compounds and assessed their MALDI matrix potential for sulfides.^564^ In subsequent studies, the team used p-Ph-CCAA as a starting point and designed a related library to establish the structure–property relationship of the MALDI-MS matrix and explore the potential applications of phenyl-α-cinnamic acid derivatives.^563^

Dufresne et al. designed and developed a series of aminocinnamic acid analogs as dual polarity matrices for high spatial resolution MALDI-MSI.^565^ They systematically investigated the vacuum stability, absorption at a wavelength of 355 nm, crystal size, and molecular coverage of these analogs. Among them, ACA and DMACA exhibited lipid IMS performance comparable or even superior to that of traditional MALDI matrices. Owing to its minimal in-source fragmentation, ACA is particularly suitable for imaging heat-sensitive molecules such as gangliosides. In mouse brain ganglioside imaging experiments, a comparison of ACA with 2,5-DHAP revealed that ACA had a greater extinction coefficient at 355 nm, which resulted in higher sensitivity to gangliosides and, consequently, better quality MALDI images than 2,5-DHAP. DMACA, with its high extinction coefficient at 355 nm and excellent vacuum stability, emerged as an ideal candidate for high spatial resolution dual polarity lipid MALDI-MSI. Under the same laser power, DMACA outperforms DAN and 2,5-DHAP in terms of signal intensity and S/N. The results of this study indicated that ACA, as a “soft” matrix, exhibited the lowest in-source fragmentation in the analysis of heat-sensitive molecules like gangliosides. In comparison, DMACA demonstrates the highest sensitivity for PLs and, in contrast to conventional MALDI matrices, achieves efficient desorption and ionization at significantly lower laser power. These matrices, which are based on the ACA, are customized in terms of their optical properties through up to three consecutive methylations. This new matrix series provides a powerful toolkit to advance molecular imaging applications and promote biological discovery research.

In recent years, the development of cinnamic acid derivatives has enabled the implementation of a multitude of practical applications of MALDI. By means of a systematic and targeted modification of the core unit functional group of α-cyanocinnamic acid, it is possible to render it capable of exhibiting excellent matrix properties. Cinnamic acid derivatives have the capacity to enhance the signal intensity and S/N ratio of mass spectra, thereby augmenting the sensitivity and reliability of the analysis. Overall, cinnamic acid derivatives have a promising future as MALDI matrices in MS, offering valuable support for research in the fields of biomedicine, biochemistry, and drug discovery.

###### 3.1.2.2.2 Benzoic Acid Derivatives

Rationally designed benzoic acid derivatives represent a novel class of matrices developed on the basis of the commonly used MALDI matrix and play important roles in biomass spectrometry. These derivatives typically exhibit favorable light absorption characteristics and suitable solubility, enabling the formation of stable co-crystals with the target molecules. This significantly enhances the intensity and stability of MS signals.^566,567^ Furthermore, the specific chemical structures of these derivatives can be optimized for different types of samples, thus enhancing the sensitivity and resolution of MS and providing reliable technical support for the analysis of biological macromolecules.^568^ A total of three types of benzoic acid derivatives are employed as MALDI matrices: lithium 2,5-dihydroxybenzoate (LiDHB), alkylated trihydroxyacetophenone (ATHAP), and COOH-NHMe­(IV) (Figure 56).

**Figure 56:**
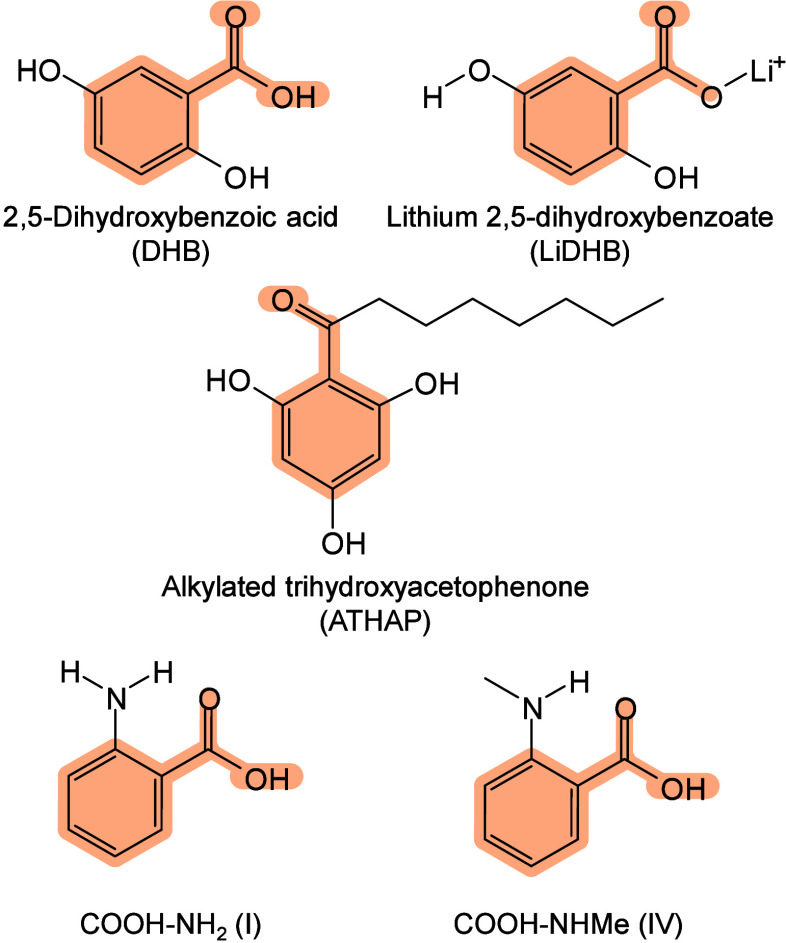
Rational design strategies for benzoic acid derivatives.

The MALDI ionization of saturated hydrocarbons constitutes a significant challenge, given that this class of compounds lacks any polar groups that are readily protonated or susceptible to attachment by anions and cations. Cvacka et al. devised a MALDI-MS analysis of lipids and HMW hydrocarbons using LiDHB as a matrix.^569^ LiDHB was demonstrated to be a straightforward and highly effective matrix for the analysis of nonpolar long-chain lipids, hydrocarbons, and polymers. Furthermore, it can facilitate the lithiation of saturated hydrocarbons and lipids, which is otherwise challenging to achieve using conventional matrices.

The detection of hydrophobic peptides by MALDI-MS is hindered by the hydrophilicity and low affinity for the peptides of conventional matrices. Fukuyama et al. demonstrated that alkylated dihydroxybenzoic acid (ADHB) can be employed as a MALDI dopant for the analysis of hydrophobic peptides.^570^ Soon after, Fukuyama et al. discovered that ATHAP is an efficacious MALDI matrix for the analysis of hydrophobic peptides.^384^ ATHAP is a THAP derivative that contains an acetyl group attached to a hydrophobic alkyl chain and has an affinity for hydrophobic peptides. A comparison of ATHAP structures with different acyl chain lengths (C6, C8, C10, and C12 acyls) revealed that octyl (C8) and decyl (C10) were the most effective at increasing the sensitivity of hydrophobic peptides. In comparison to the dopant ADHB, the sensitivity of ATHAP to hydrophobic peptides was enhanced by a factor of 10, and the peptide signal could be discerned throughout the matrix–analyte dry spot. Furthermore, ATHAP can be employed as a standalone matrix, obviating the need for a dopant such as ADHB.

Huang et al. designed and synthesized a series of new dual-polarity MALDI matrices (I–IV) based on their acid-base bifunctionality, with the objective of improving the detection efficiency of MALDI-MS.^571^ The matrices COOH-NH_2_(I) and COOH-NHMe­(IV) contain carboxyl-anchored simple ANI derivatives, whereas CHO-NH_2_(II) and CHO-NHAc­(III) contain formyl groups. Their findings demonstrated that the amphiphilic character of COOH-NH_2_(I) and COOH-NHMe­(IV) facilitated the concurrent detection of the molecules in both positive and negative ionic modes. Notably, COOH-NHMe­(IV) exhibited remarkable efficacy in ion generation for lipids and proteins under laser excitation, outperforming commercially available matrices, particularly for the analysis of mouse brain samples.

The selection of a MALDI matrix is paramount in the outcome of MS. Benzoic acid derivatives are extensively employed in bioanalysis, pharmaceutical research, and other domains because of their exceptional properties. Taking benzoic acid as the core unit, benzoic acid derivatives obtained by rational design and the addition of functional groups can be employed as efficient MALDI matrices. First, these benzoic acid derivatives exhibit favorable ultraviolet absorption characteristics and enable effective absorption of laser energy, which in turn facilitates photolysis and ionization of the sample during the MALDI process. This results in an enhancement in the intensity and stability of the MS signal. Second, benzoic acid derivatives contain a plethora of functional groups, including carboxyl, amino, alkyl, and others, which can effectively facilitate the ionization of analytes. These derivatives may interact with target molecules through mechanisms such as hydrogen bonding and van der Waals forces, which can enhance the ionization efficiency of samples and the signal intensity. In conclusion, benzoic acid derivatives serve as highly effective MALDI matrices, demonstrating advantageous light absorption characteristics, volatility, and chemical affinity. These attribute enhance the sensitivity, resolution, and reliability of MS analysis. As a result, these derivatives have emerged as a widely favored options in biomedical, biochemical, and pharmaceutical studies.

###### 3.1.2.2.3 Other Matrix Derivatization

To rationally design novel MALDI matrices, it is first necessary to consider the chemical properties and molecular structure of the matrix, as well as its interaction with the target analytes. Matrix derivatization allows the introduction of different functional groups or alteration of the molecular structure, which can enhance the interaction between the matrix and the target analytes and improve the signal intensity and resolution of MS. Furthermore, a comprehensive consideration of the stability of the matrix on the solid phase, light absorption properties, crystal morphology, and other factors is essential for the effective detection and quantitative analysis of different types of analytes. This must be accompanied by experimental verification of the matrix's performance. In light of the aforementioned factors, researchers have devised and synthesized organic aromatic acid lithium salts, 1-aminopyrene (1-AP)-derived group of uniform materials based on organic salts (GUMBOS) matrices, DAN hydrochloride, aggregation-induced luminescence (AIE) compounds, 1,8-di­(piperidinyl)-naphthalene (DPN), 5-(3-trifluoromethylbenzylidene)­thiazolidine-2,4-dione (3-CF_3_-BTD), (*E*)-4-(2,5-dihydroxyphenyl)­but-3-en-2-one (2,5-cDHA), and 4,5-dimethoxy-2-nitrobenzyl-2,5-dihydroxyacetophenone (DMNB-2,5-DHAP) as novel MALDI matrices.

A novel MALDI matrix based on lithium salts was developed by Horka et al. for the analysis of hydrocarbons and wax esters. They first synthesized various lithium salts of organic aromatic acids including lithium benzoate (LiBA), lithium salicylic acid (LiSalA), lithium vanillate (LiVA), lithium 2,5-dimethoxybenzoate (LiDMB), lithium 2,5-dihydroxyterephthalate (Li_2_DHT), lithium CHCA, and lithium sinapate (LiSA). LiVA, LiSA, and LiSalA were demonstrated to be capable of detecting long-chain hydrocarbons in standards and complex mixtures with high sensitivity.^572^ Among these matrices, LiVA exhibited the most effective performance, indicating a strong signal strength and high spectral reproducibility.

Conventional MALDI matrices are amphiphilic but exhibit predominantly hydrophilic behavior, which can limit their affinity for hydrophobic peptides and pose a detection challenge in MALDI-MS.^384^ However, Ghafly et al. demonstrated that hydrophobically tunable GUMBOS matrices can effectively address this issue.^573^ A series of 1-AP-based GUMBOS matrices were synthesized, including [1-AP]­[Cl], [1-AP]­[ascorbate (Asc)], and [1-AP]­[bis­(trifluoromethane)­sulfonimide (NTf_2_)] (Figure 57). The signal intensity of the hydrophobic peptides was significantly correlated with the hydrophobicity of the matrix. A hydrophobicity-tunable GUMBOS matrix can be readily obtained by simply modifying the counterion. The use of hydrophobic matrices proved highly effective for the MALDI determination of hydrophobic peptides. Similarly, more hydrophilic peptides exhibited higher signal intensity in more hydrophilic matrices.

**Figure 57:**
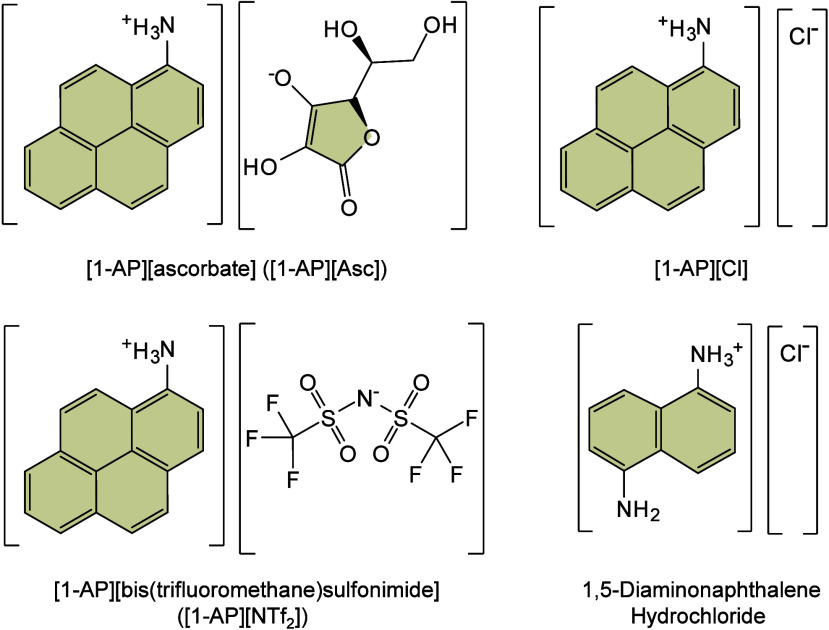
Rational design strategies for 1-aminopyrene salts.

DAN hydrochloride as a MALDI matrix demonstrated a more straightforward mass spectral characterization in comparison to DAN, particularly at *m*/*z* values below 500, accompanied by a notable reduction in the background signal.^574^ This novel matrix is suitable for the analysis of small-molecule compounds in negative ion mode, exhibiting notable characteristics such as low cost, strong UV absorption, high salt tolerance, and a low background signal (especially in the low-mass range). Furthermore, the use of this matrix facilitated the concurrent observation of the spatial distribution of an extensive range of small-molecule metabolites, encompassing metal ions, AAs, carboxylic acids, nucleotide derivatives, peptides, and lipids.

AIE compounds are a class of luminescent materials that were initially described by Luo et al.^575^ These compounds exhibit distinctive aggregation properties, with weak luminescence in solution but strong luminescence in aggregates. Yao et al. innovatively synthesized *N*,*N*′-bis­(4-hydroxysalicylidene)*p*-phenylenediamine (BSPD-OH), *N*,*N*′-bis­(4-methoxysalicylidene)*p*-phenylenediamine (BSPD-OMe), and *N*,*N*′-bis­(salicylidene)*p*-phenylenediamine (BSPD), which belongs to the AIE compounds based on the Schiff base reaction, was employed as a novel matrix for MALDI-MS analysis of small molecules (Figure 58).^576^ These AIE matrices exist in the form of aggregates, which effectively reduce the background interference of the matrix. Furthermore, they display high sensitivity, as the aggregates enhance the absorption of laser emission and modulate the ionization efficiency by altering the aggregation state. The results demonstrated that BSPD-OH exhibited superior ionization efficiency in comparison to the other two AIE matrices, due to its greater number of phenolic hydroxyl groups. This matrix was successfully employed in the analysis of LMW compounds, including AAs, organic amines, isoquinolines, and fluoroquinolones.

**Figure 58:**
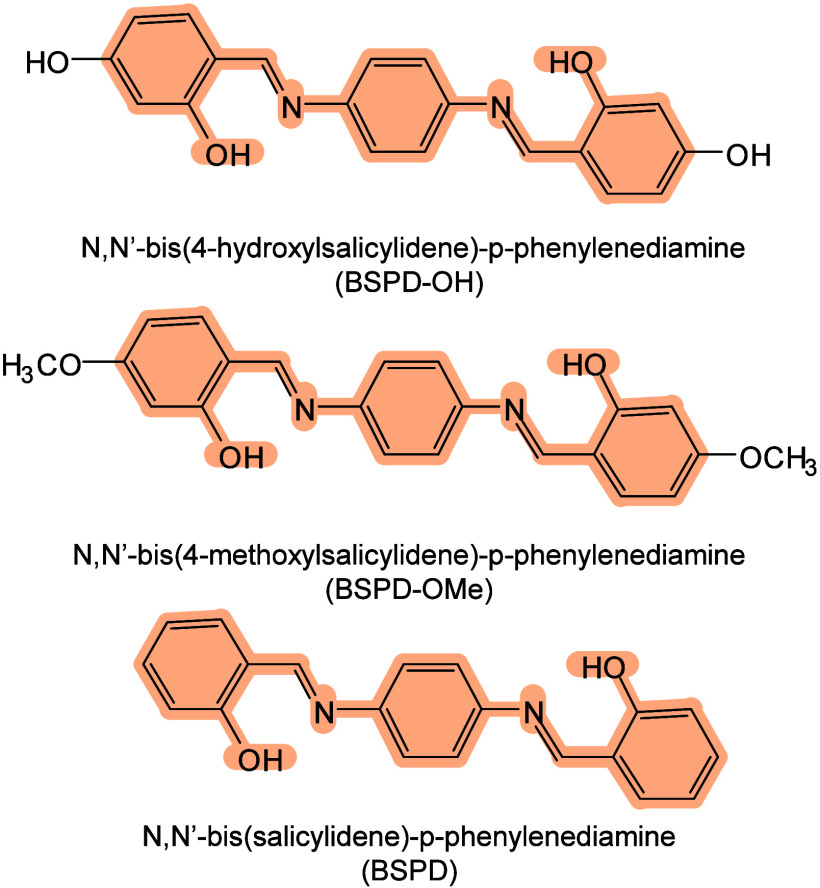
Rational design strategies for aggregation-induced emission (AIE) compounds.

In addition to compounds like AIE mentioned earlier, Figure 59 illustrates rational design strategies for other compounds. Using DMAN as a foundational structure, researchers have devised and synthesized a series of superbasic compounds with the objective of enhancing their sensitivity and detection performance as MALDI matrices. By increasing the distance between the diazonium-centered chelating proton and the deprotonated anion as well as increasing steric repulsion, Weißflog and Svatoš designed DPN and found that it outperforms conventional matrices in metabolomics and MSI in negative ion mode for a wide range of analytes with various acidities.^577^

**Figure 59:**
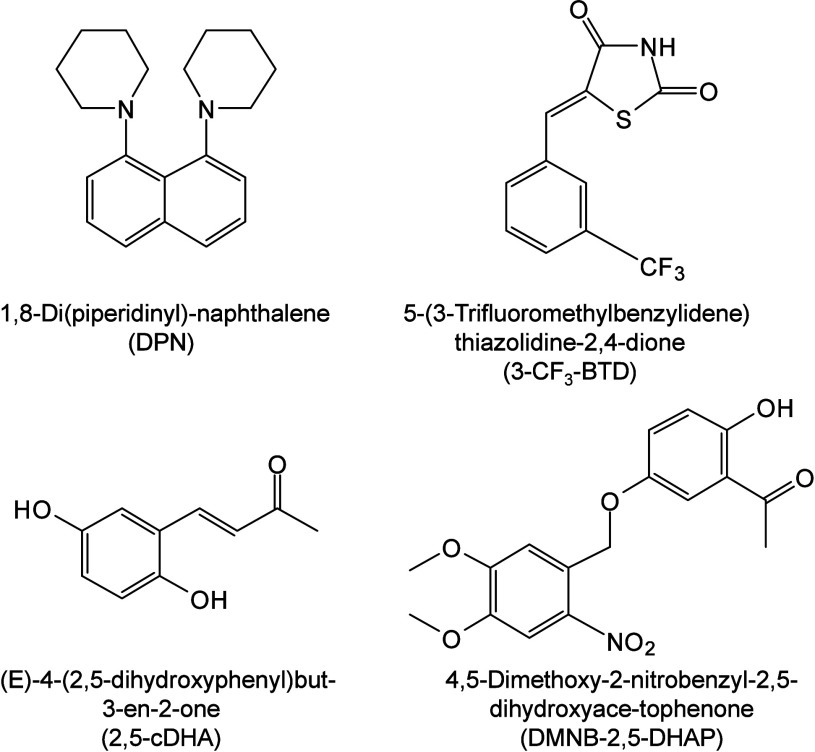
Rational design strategies for other compounds.

The use of 3-CF_3_-BTD as a MALDI matrix enabled the sensitive analysis of biological monoamine transmitters, including DA, serotonin, histamine, and adrenaline, at the picomolar level.^322^ Concurrently, 3-CF_3_-BTD can be effectively employed for the analysis of LMW compounds, and is capable of replacing the conventional matrix CHCA, whose carboxyl and phenolic hydroxyl groups may be incompatible with specific analyte functional groups.

The introduction of 2,5-DHAP as a MALDI matrix has enabled MALDI-MS analysis of peptides, proteins, and glycoproteins, with sensitivity superior to that of SA. However, the volatility of 2,5-DHAP under high vacuum restricts its prolonged use. It is therefore important to investigate matrices with a structure similar to that of 2,5-DHAP that are suitable for the detection of HMW compounds to improve vacuum stability and obtain high-quality images. Yang et al. identified a novel vacuum-stabilized ketone-based matrix suitable for high spatial resolution MALDI imaging.^157^ The compound 2,5-cDHA, based on the structure of 2,5-DHAP, exhibits significantly improved vacuum stability and high sensitivity, and is capable of producing small crystals, which makes it suitable for imaging proteins in biological tissues. Moreover, 2,5-cDHA displays excellent detection capabilities for LMW compounds in both positive and negative ion modes.

Inadequate vacuum stability of the matrix represents a significant limitation to the application of MALDI-MS. Zhou and colleague devised a novel caged intrasource laser-cleavable MALDI matrix with high vacuum stability, which has the potential to extend the application of MALDI imaging.^53^ The proposed cage matrix design employs 4,5-dimethoxy-2-nitrobenzene (DMNB) to transform existing volatile matrices into vacuum-stabilized matrices. Consequently, a photocleavable cage molecule, DMNB-2,5-DHAP, was successfully synthesized and subjected to vacuum stabilization in a high vacuum MALDI ion source for a minimum of 72 hours, showing the capacity to perform lipid MALDI-MSI for mouse brain tissue sections. This study introduces a novel approach to the design of a MALDI matrix, incorporating a caged moiety that can be lifted by laser irradiation during the detection process. This enhances the lipid ion intensity through the use of MALDI-2 laser-induced localization and provides new opportunities for MALDI-MS imaging.

Tetrabromobisphenol A (TBBPA) and tetrabromobisphenol S (TBBPS) have garnered significant attention due to their widespread use as dopants in various products and the consequent health risks associated with their residues entering the human diet. Current detection methods face challenges in achieving direct and sensitive identification of these compounds in food matrices. Recent advancements in MALDI-TOF-MS offer a promising alternative for analyzing low-mass environmental pollutants. Chen et al. used a functional melanin nanoparticles (COOH-MNP-COOH) matrix, which enhances deprotonation efficiency, thereby improving the detection sensitivity of TBBPA and TBBPS in animal-derived food samples when operated in negative-ion mode.^578^ This novel matrix demonstrates superior stability, reproducibility, and tolerance to salts and proteins, alongside enhanced UV-vis absorption and biocompatibility. The integration of an internal standard facilitates detection limits of 300 and 200 pg/mL for TBBPA and TBBPS, respectively. This approach not only enables direct residue identification in complex food matrices like milk and meat but also presents a viable strategy for monitoring environmental pollutants in food products, thereby contributing to enhanced food safety and public health protection.

The design of a new MALDI matrix necessitates the consideration of several factors, including structural design, chemical reaction selection, biocompatibility, selectivity, solubility, thermal stability, sensitivity, and homogeneity.^571^ Matrix derivatization can improve the performance of the matrix, increase the sensitivity and selectivity of the ion signal, facilitate the detection of low concentration analytes, and guarantee the stability and reproducibility of the MS signal.^526^ Furthermore, the matrix design process requires systematic validation and analysis to accurately assess its performance and to continuously optimize and improve it until the most suitable matrix for a particular application is selected.

###### 3.1.2.2.4 Summary

To enhance the sensitivity, resolution, and applicability of the MALDI technique, researchers have focused on the rational design and modification of the matrix via derivatization reactions. This strategy holds promise for improving the matrix’s UV absorption characteristics and ionization efficiency while concurrently mitigating background noise. For example, the matrix’s compatibility with specific samples can be optimized by introducing diverse functional groups onto the matrix molecules and fine-tuning their chemical and physical properties. Furthermore, the derivatization reaction can enhance the stability and reproducibility of the MALDI matrix, thereby optimizing the performance of MALDI in the analysis of complex samples. In conclusion, matrices obtained by a derivatization reaction represent a significant advance in MALDI-MS, both in terms of the application scope and the overall performance of MS. Thus, such matrices provide a highly reliable and efficient tool for research in numerous fields, including biomedicine, environmental monitoring, and material science.

##### 3.1.2.3 Ionic Liquid Matrices

Since their initial discovery in 1914, ionic liquids (ILs) have been employed extensively in a multitude of research domains. Notably, the application of ILs in analytical chemistry has been a significant and rapid contributor to the advancement of the field. ILs are collectively referred to as organic salts and have a melting point that is generally at or below 100°C.^579^ If the melting point is equal to or below room temperature, they are designated as room temperature ILs (RTILs), which exhibit low vapor pressure and the capacity to dissolve a diverse range of analytes. In most cases, ILs comprise an organic cation and an organic or inorganic anion. It has been estimated that there are as many as 10^18^ potential combinations of ILs, given the interchangeability of anions and cations. The advantages of ILs include high thermal stability, negligible vapor pressure, and non-flammability. These properties are attributed to the electrostatic interactions between cation and anion molecules, as well as their unique ability to interact with each other on an intermolecular basis. Furthermore, these properties can be precisely adjusted to align with specific requirements by attaching distinct functional groups or modifying the combination of cations and anions in the ionic liquid. In light of these properties, ILs may serve as a viable alternative to conventional organic solvents in numerous analytical applications and sample preparation techniques. Consequently, they are proving to be a valuable resource for a range of practical applications, including GC and capillary electrophoresis.

The use of RTILs as a matrix (ionic liquid matrix, ILM) for MALDI was initially documented by Armstrong et al. in 2001.^580^ The combination of various amine-based cations with the commonly used matrix anions SA and CHCA yielded ILs with the advantages of high solubility, negligible vapor pressure, and a wide liquid temperature range. The synthesis and testing of several different ILMs were conducted using peptides, proteins, and polyethylene glycol (PEG-2000) as analytes. Their results revealed that these ILs exhibited notable differences from conventional solid matrices in terms of their vacuum stability, ion peak intensities, and ability to generate gas-phase ions of the analytes. Furthermore, some ionic matrices have demonstrated enhanced performance.^580^ The optimal characteristics of liquid and solid matrices can be combined with ionic matrices. The use of ILMs results in the production of homogeneous sample solutions that exhibit excellent vacuum stability. In the context of protein analysis, they have been observed to yield higher spectral peak intensities and lower detection limits than comparable solid matrices in a considerable number of cases. Nevertheless, the quest for efficient ILMs presents significant challenges.^62,581^ It is currently not feasible to accurately predict whether a specific cation or anion will form a salt with a low melting point. Second, even if ILs are successfully obtained, they may not effectively promote the ionization of the target analyte. It is therefore necessary to optimize both the cationic and anionic portions of ILMs.^63,582^ In general, existing ILMs can be specifically divided into three main categories: ILs based on DHB, CHCA, and others.

###### 3.1.2.3.1 Ionic Liquid Matrices Based on DHB

Recently, ILMs, especially those originating from DHB, have attracted considerable interest owing to their distinctive properties and versatile applications across diverse analytical domains. The chemical structure of the DHB-based ILM, illustrated in Figure 60, is pivotal in refining MALDI processes to achieve superior analytical sensitivity and accuracy. One of the primary challenges associated with MALDI-MS for qualitative and quantitative measurements is the heterogeneous distribution of analytes and matrices in solid sample preparation. Zabet-Moghaddam et al. conducted qualitative and quantitative analyses of LMW compounds using UV-MALDI-MS based on ILMs. The matrices comprised classical MALDI matrices (SA, CHCA, and DHB) in equimolar combinations with organic bases (pyridine (Pyr), 1-methylimidazole (IM), and tributylamine (TBA)). ILMs are typically characterized by their ability to form thin layers of liquid with very low vapor pressures.^583^ Compared to conventional solid matrices, ILMs facilitated homogeneous sample preparation and contributed to more precise and accurate qualitative and quantitative measurements of analyt, with DHB/Pyr showing the best performance.

**Figure 60:**
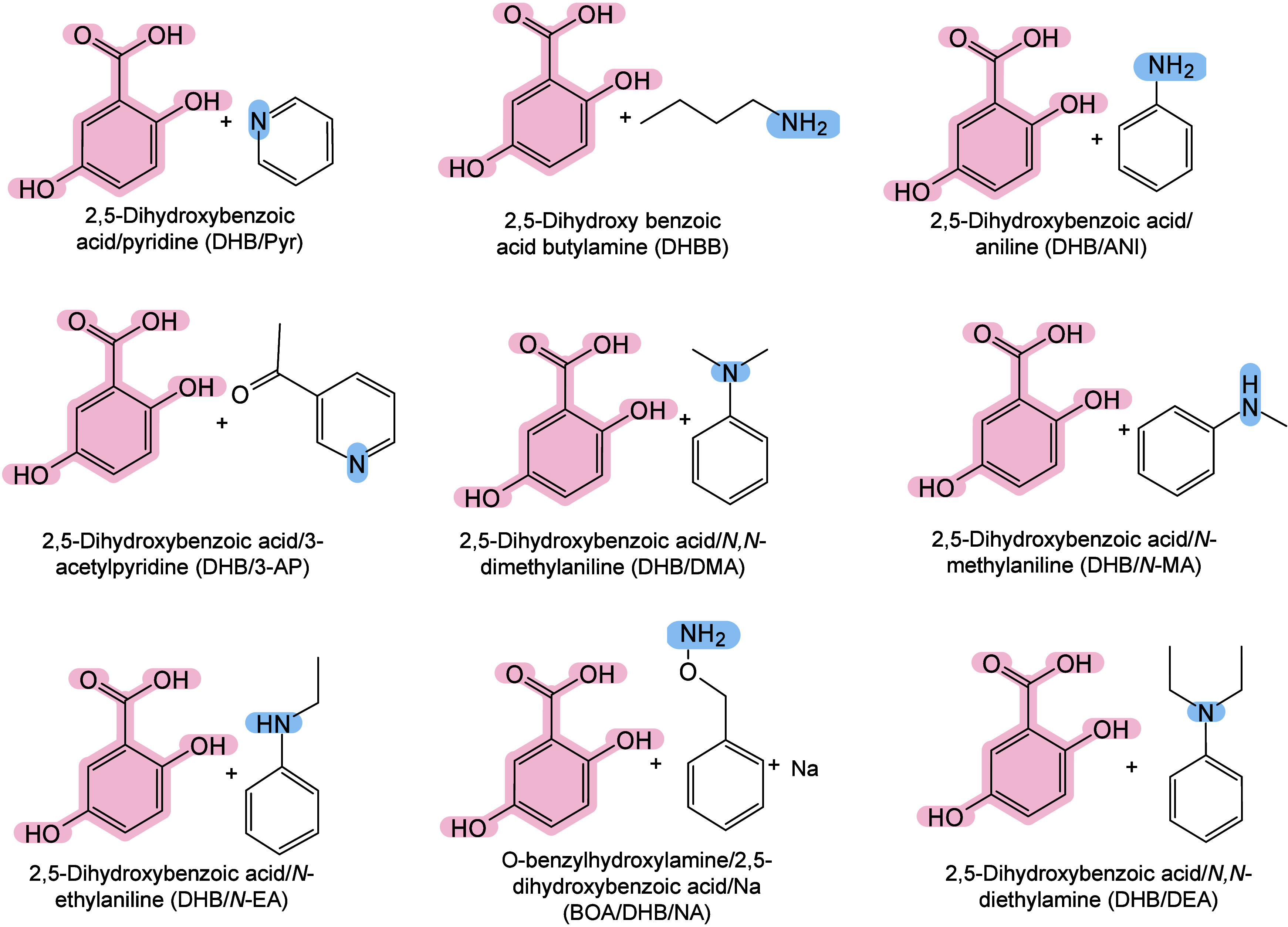
Chemical structures of ionic liquid matrices based on DHB.

Schnoll-Bitai et al. successfully characterized the molecular weight distribution of pullulans using 2,5-dihydroxybenzoate butylamine (DHBB) as a liquid matrix for MALDI-MS.^584^ In comparison with the solid matrices DHB and THAP, the DHBB matrix yielded superior results, exhibiting minimal sample fragmentation and an exceptionally low background noise level, thereby facilitating the analysis of pullulan samples up to 12,000 Da. Mank and coworkers investigated a range of ILMs for enhanced MALDI-MS analysis of biomacromolecules and found that DHBB has broad applicability and is suitable for the analysis of oligosaccharides and polymers.^585^ Meriaux et al. designed three ILMs (*i.e.*, DHB/ANI, DHB/Pyr, and DHB/3-acetylpyridine (3-AP)) based on DHB for the purpose of detecting lipids.^586^ Their results demonstrated that these ILMs were stable during deposition and capable of forming a homogeneous matrix coating. Notably, the DHB/3-AP matrix exhibited efficient lipid detection performance in both positive and negative ion modes. Abdelhamid et al. synthesized and successfully employed novel ILMs for the analysis of pathogenic bacteria in MALDI-MS.^48^ These matrices were based on the coupling of conventional organic matrices (SA and DHB) with a variety of bases (ANI, *N*,*N*-dimethylaniline (DMA)), *N*,*N*-diethylamine (DEA), dicyclohexylamine (DCHA), Pyr, 2-pyridine (2-P), and 3-pyridine (3-P)), which significantly enhanced the protein signals, reduced the spot-to-spot variations, and improved the homogeneity. Among them, DHB/ANI, DHB/DEA, and DHB/Pyr demonstrated particularly impressive performance in intact bacteria studies. Snovida and colleagues demonstrated that the sensitivity of MALDI-MS analysis of oligosaccharides could be enhanced by utilizing the binary matrix DHB/DMA. Compared to the conventional DHB matrix, the morphology of the matrix crystal layer was more homogeneous, which facilitated a high degree of homogenization of the sample distribution and contributed to the acquisition of reproducible and consistent MS signals. This provided a novel method for the rapid, straightforward, and sensitive analysis of neutral carbohydrates.^587^ Zhao et al. evaluated the potential of novel ILMs, specifically DHB/*N*-methylaniline (*N*-MA) and DHB/*N*-ethylaniline (*N*-EA), for the qualitative and quantitative detection of carbohydrates by MALDI-MS. Their findings revealed that compared to conventional DHB matrix, both matrices exhibited superior detection performance, with significantly enhanced ion generation efficiency.^588^ Barada and Hinou developed an efficient UV-solid ionization matrix, *O*-benzylhydroxylamine/DHB/Na (BOA/DHB/Na), for the highly sensitive and automated labeling of glycan chemistry.^589^ The matrix, which combined BOA and a small amount of sodium, exhibited excellent aggregation properties, was capable of forming uniform solid salts on water-resistant MALDI target plates, and it significantly improved the ionization efficiency of the glycan while reducing ISD and PSD.

###### 3.1.2.3.2 Ionic Liquid Matrices Based on CHCA

CHCA-based ILMs facilitate MALDI-MS analysis, presenting multiple advantages over single CHCA matrices, such as enhanced protein analysis capabilities and higher secondary ion yields. The chemical structure of the ILM derived from CHCA is depicted in Figures 61–64. While conventional ILMs consist of an organic cation and an organic or inorganic anion, CHCA-derived ILMs allow the incorporation of amines, pyridines, imidazoles, and aminoquinolines as cation donors.

**Figure 61:**
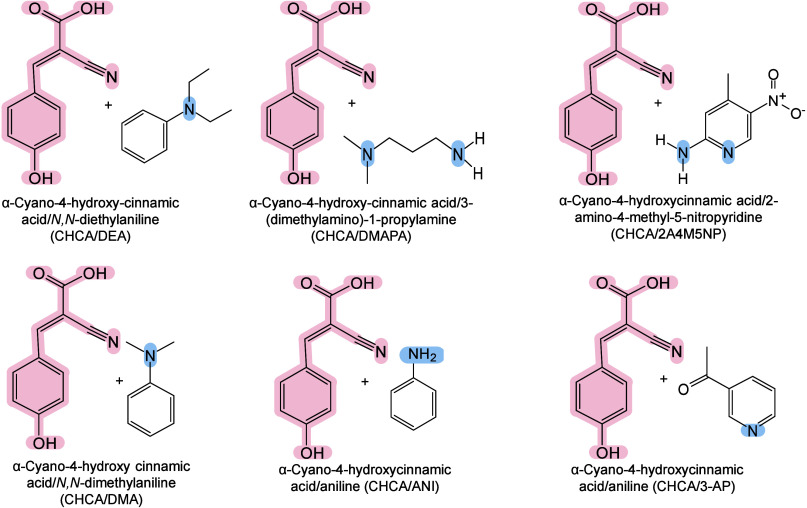
Chemical structures of ionic liquid matrices based on CHCA (I).

**62:**
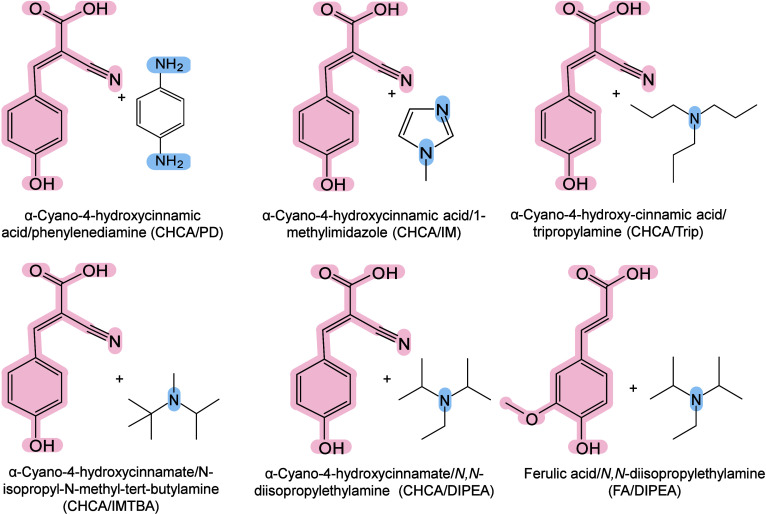
Chemical structures of ionic liquid matrices based on CHCA (II).

**63:**
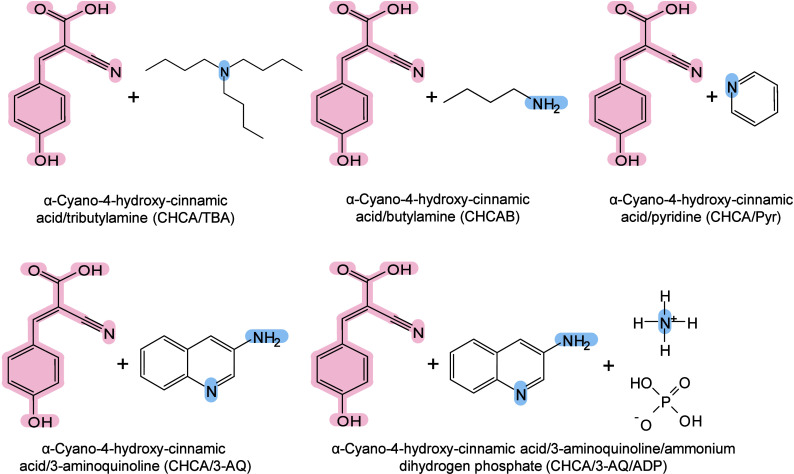
Chemical structures of ionic liquid matrices based on CHCA (III).

**Figure 64:**
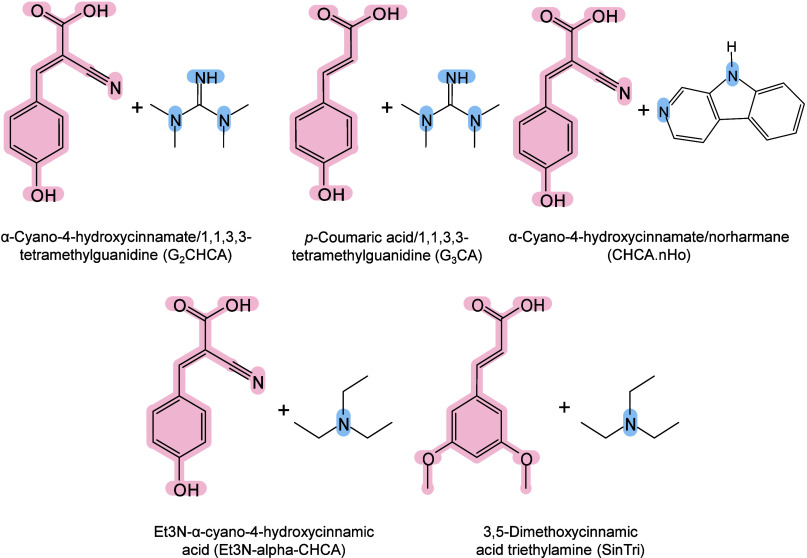
Chemical structures of ionic liquid matrices based on CHCA and analogue (IV).

ILMs present considerable benefits for the MALDI-MS analysis of peptides, oligonucleotides, and PLs.^588,590^ Compared to solid matrices, CHCA-derived ILMs demonstrated superior laser energy transfer efficiency and higher MS signal intensities. Their results indicated that CHCA/DEA enhanced sensitivity to purified compounds and complex biological samples, produced higher signal intensity and better reproducibility, and retained the ability to analyze a diverse range of biological compounds. Tholey et al. employed CHCA/3-(dimethylamino)-1-propylamine (DMAPA) as an ILM for the quantitative monitoring of peptides in protease-catalyzed reactions using MALDI-MS.^591^ They investigated the efficacy of different combinations of acids (CHCA and indoleacrylic acid (InAA)) and bases (*N*,*N*-dimethylethylenediamine (DMED) and DMAPA) for the detection of peptide compounds. As a result, the signal intensity of peptide compounds detected by MALDI-MS was particularly elevated when CHCA/DMAPA was used as the ILM. Lemaire et al. observed a notable increase in signal intensity when CHCA/2-amino-4-methyl-5-nitropyridine (CHCA/2A4M5NP) and CHCA/DMA were used for the analysis of tissue peptides.^592^ Compared with conventional CHCA, CHCA/DMA has been demonstrated to exhibit superior signal intensity, sensitivity, image quality, and reproducibility. Liu et al. identified ionic matrices suitable for matrix-enhanced SALDI-MSI (ME-SALDI-MSI), such as CHCA/ANI, which excelled in eliminating the redistribution of analytes associated with tissue surfaces,^593^ enhanced vacuum stability, and improved ionization efficiency. Calvano et al. also reported CHCA/ANI as a versatile MALDI-MS ILM capable of yielding results identical to those obtained with DHB, but at lower laser energies, resulting in improved mass resolution and reduced analyte fragmentation.^594^ Furthermore, Bonnel et al. devised an ionic matrix prespotted MALDI matrix plate for monitoring patients undergoing treatment and drug titration.^595^ Their study revealed that peptides of up to 5 kDa could be analyzed using CHCA/ANI, peptides of up to 8 or 9 kDa could be analyzed using CHCA/3-AP, and peptides of up to 67 kDa could be analyzed using CHCA/phenylenediamine (CHCA/PD). These combinations demonstrated exceptionally high sensitivity and spectral quality.

The development of ILMs for the MALDI-MS analysis of peptides, proteins, and carbohydrates by Crank and Armstrong showed that CHCA/*N*,*N*-diisopropylethylammonium (CHCA/DIPEA) and CHCA/*N*-isopropyl-*N*-methyl-*t*-butylammonium (CHCA/IMTBA) were the best matrices for analyzing proteins and peptides, whereas CHCA/DIPEA and FA/DIPEA were the best matrices for analyzing carbohydrates.^63^ Furthermore, CHCA/DIPEA was found to be an efficacious matrix for the examination of biodegradable polymers, enabling maximal signal output at minimal laser intensity, reducing polymer degradation, and facilitating analyte ionization in an effective and gentle manner.^596^ Calvano et al. utilized an ILM, CHCA/TBA, for the detection of PLs by MALDI-TOF-MS, which significantly improved the sensitivity and selectivity of the PL fraction of the analyzed samples.^597^ Shrivas et al. identified an ionic matrix, α-cyano-4-hydroxycinnamic acid butylamine (CHCAB), for the enhanced MALDI imaging and identification of PLs in mouse liver and brain tissue sections.^598^ The number of molecular signals observed in the samples increased, as did the signal intensity, when CHCAB was used as a matrix. Furthermore, compared with the DHB matrix, CHCAB demonstrated greater sensitivity for identifying lipid species and was more effective at detecting biomacromolecules in the different layers of the cerebellum. In addition, Mank et al. found CHCAB is the preferred matrix for peptides. Furthermore, they found 3,5-dimethoxycinnamic acid triethylamine (SinTri) exhibits as the best matrix for HMW proteins such as IgG.^585^

Pyr-based ILMs also enhance protein identification in peptide mass fingerprinting by MALDI-MS.^591^ Masoud et al. discovered that when the molar ratio of CHCA/Pyr was 2:1, the optimal outcomes in terms of the S/N ratio, reduction in chemical noise and base adducts, and matrix cluster formation were attained. Consequently, CHCA/Pyr could serve as a prospective alternative approach for protein identification and characterization via peptide mass fingerprinting. Naumann et al. successfully identified thioglycolipids using the ILM by MALDI-MS.^599^ The formation of more precise and homogeneous spots during co-crystallization on the MALDI target allows for higher signal intensities to be obtained compared to conventional matrices. In the experiment, the best mass spectral results were obtained when 1-butylamine-a-cyano-4-hydroxycinnamic acid (1-butylamine-CHCA) was used as the ILM.

ILMs exhibited superior performance in MALDI-TOF MS for PL analysis. These ILMs achieved higher signal intensity, smaller spot size, and more reliable signal reproducibility compared to the solid matrix DHB. In particular, the CHCA/IM combination demonstrated superior performance in terms of sample homogeneity and PL analysis.^58^ Fitzgerald et al. synthesized and tested two CHCA-derived ILs (CHCA/IM and CHCA/tripropylammonium α-hydroxycinnamate (CHCA/Trip)) for the analysis of PLs, cholesterol, and peptides using matrix-enhanced SIMS (ME-SIMS) with room-temperature ILMs. The results showed that the use of ILMs markedly enhanced the signal intensity of molecular ions and facilitated the detection of analytes at a sensitivity that was at least two orders of magnitude greater.^600^ In addition, CHCA/IM has been employed in a multitude of additional studies, including the MALDI-MSI analysis of gangliosides in the mouse brain,^601^ quantitative analysis of polyhexamethylene guanidine oligomers,^602^ and detection of uncomplexed highly sulfated oligosaccharides.^603^

A method has been developed for the deposition of liquid matrices onto conductive hydrophobic surfaces, which is specifically designed to facilitate tuning and quantitative analysis within UV-MALDI mass spectrometers.^604^ The use of ILMs (CHCA/3-AQ) has the potential to enhance the capabilities of mass spectrometers, facilitating a range of studies that are currently not feasible with solid matrices. Future developments will focus on optimizing the composition of liquid matrices for specific analyses or experiments. Furthermore, Yamazaki et al. successfully analysed HMW polyoxymethylenes (PRs) by MALDI-MS utilizing a 3-AQ-based ILM. CHCA/3-AQ was found to be an appropriate combination for CD-PR analysis and was successfully employed to analyze a range of high to ultrahigh HMW PRs with molecular weights of between 90 and 700 kDa.^605^ Towers et al. introduced a ClCCA ILM for high-sensitivity UV-MALDI-MS and demonstrated that ClCCA/3-AQ exhibited a notable enhancement in sensitivity. This ClCCA/3-AQ exhibited the advantageous characteristics of LM and the elevated sensitivity associated with the recently developed ClCCA matrix.^606^ Mukherjee et al. successfully combined 3-AQ, CHCA, and ammonium dihydrogen phosphate (ADP) as a solidified ILM for MALDI-MS analysis of phosphopeptides.^607^ The CHCA/3-AQ/ADP combination offered the benefits of both liquid and solid MALDI matrices to form finely and homogeneously dispersed matrix–analyte surfaces, achieving highly reproducible phosphopeptide analyses in the low femtomolar range.

In addition, the guanidine salt of CHCA (G_2_CHCA) ILM was suitable for the direct MALDI-MS analysis of corticosteroid sulfate and chondroitin sulfate oligosaccharides, which facilitated the detection of uncomplexed, unacidified polysulfated, and polycarboxylated oligosaccharides.^608^ The 1,1,3,3-tetramethylguanidine (TMG) salt of *p*-coumaric acid (G_3_CA) and available G_2_CHCA ILMs allowed the highly sensitive detection of sulfated/amidated/neutral oligosaccharides and glycopeptides.^609^ The ILM CHCA.nHo, comprising NRM and CHCA, exhibited optimal performance in the detection of LMW carbohydrates in both positive and negative ion modes.^610^ Et3N-alpha-CHCA was employed as an ILM for the screening of aflatoxins by MALDI-MS and demonstrated the ability to detect B1, B2, G1, and G2 aflatoxins with high sensitivity and minimal sample preparation, obviating the need for derivatization or chromatographic separation.^611^

###### 3.1.2.3.3 Other Ionic Liquid Matrices

Researchers have synthesized a range of ILMs and successfully used them in the MALDI-MS analysis of a variety of compounds. These matrices enhanced the accuracy and precision of the analytical results, improved the S/N ratio, reduced the fragmentation/desulfurization process, and enhanced point-to-point reproducibility and ionization efficiency. ILMs have demonstrated a diverse range of applications in MALDI-MS analysis across multiple disciplines, including glycopeptides, glycans, lipids, small molecules, polymers, biomarkers, DNA oligomers, and lignin. Apart from the DHB/CHCA-based ILMs, Figures 65 and 66 display the chemical structures of the remaining ILMs. Among the aforementioned applications, Zabet-Moghaddam et al. discovered that MALDI-MS can be employed for the characterization of ILs themselves as well as for the analysis of AAs, peptides, and proteins in ionic liquids. The corresponding ionic signals were observed for different types of ILs.^612^ Przybylski and colleagues synthesized two novel ILMs, HABA/TMG2 and HABA/SPM, based on HABA and successfully applied them for MALDI-MS analysis of heparin and heparin sulfate oligosaccharides.^613^ Their results showed that the novel ILMs not only improved the S/N ratio but also reduced the fragmentation/desulfurization process and cation exchange. In contrast, Ullmer and Rizzi conducted MALDI-MS analysis of glycopeptides and glycans utilizing the innovative ILM 1,1,3,3-tetramethylguanidine-2,4,6-trihydroxyacetophenone (GTHAP), effectively addressing the challenge of the ionization inhibition of carbohydrate structures in the presence of peptides.^614^ Furthermore, Serrano et al. performed MALDI-MS analysis of aliphatic biodegradable photoluminescent polymers using the novel ILMs, 3-HC/DIPEA and THAP/DIPEA. Their findings revealed that the matrices exhibited excellent reproducibility and high ionization efficiency.^615^ By combining the UV absorber PNA (PNA/butyric acid), which enhances the lipid reaction, with the protonator butyric acid, Ham et al. successfully synthesized a novel solid ionic crystal matrix. This matrix functioned as a robust gas-phase proton donor, markedly augmenting the phosphorylated lipid reaction.^616^ In contrast, Gabriel et al. devised three distinct ionic matrices for the MALDI-MS analysis of synthesized polymers, namely DHB/*n*-butylamine, SA/triethylamine, and DCTB/*n*-butylamine.^617^ Abdelhamid et al. designed, characterized, and applied a series of novel ILMs based on mefenamic acid (MA/ANI, MA/DMA, MA/Pyr, MA/2-P). Their findings revealed that this family of matrices offers numerous practical advantages for research in the fields of carbohydrates, biomedicine, pharmaceuticals, and AAs.^618^ Furthermore, other novel ILs and solid ionic crystal matrices are extensively employed for MALDI-MSI analysis. For instance, ATT/Pyr can be employed for the analysis of microRNA biomarkers.^619^ The synthesis of ILs from two acids (3-HPA and DHB) with different bases has been shown to enhance the ionic peak intensities of oligonucleotides.^620^ Wang et al. innovatively employed the ionic liquid 1-butyl-3-methylimidazolium tetrafluoroborate ([BMIM]­BF_4_) as the matrix in MALDI-MS, significantly enhancing the quantitative accuracy and reproducibility of low-polarity compound analysis.^621^ This study cleverly utilized the low volatility and excellent solubility of [BMIM]­BF_4_, effectively mitigating the issue of analyte fluctuation caused by co-evaporation during D/I with traditional volatile matrices. The results demonstrate the successful detection of 17 low-polarity drug molecules and 4 pesticide molecules, showcasing the great potential of ILMs in enhancing MALDI-MS analytical performance. The use of LMs with FA, CHCA, and DHB as anions and DIPEA, IMTBA, 3-AQ, Pyr, and MI as cations has been demonstrated to efficiently D/I lignin, generating singly charged protonated molecules of its oligomers.^622^

**Figure 65:**
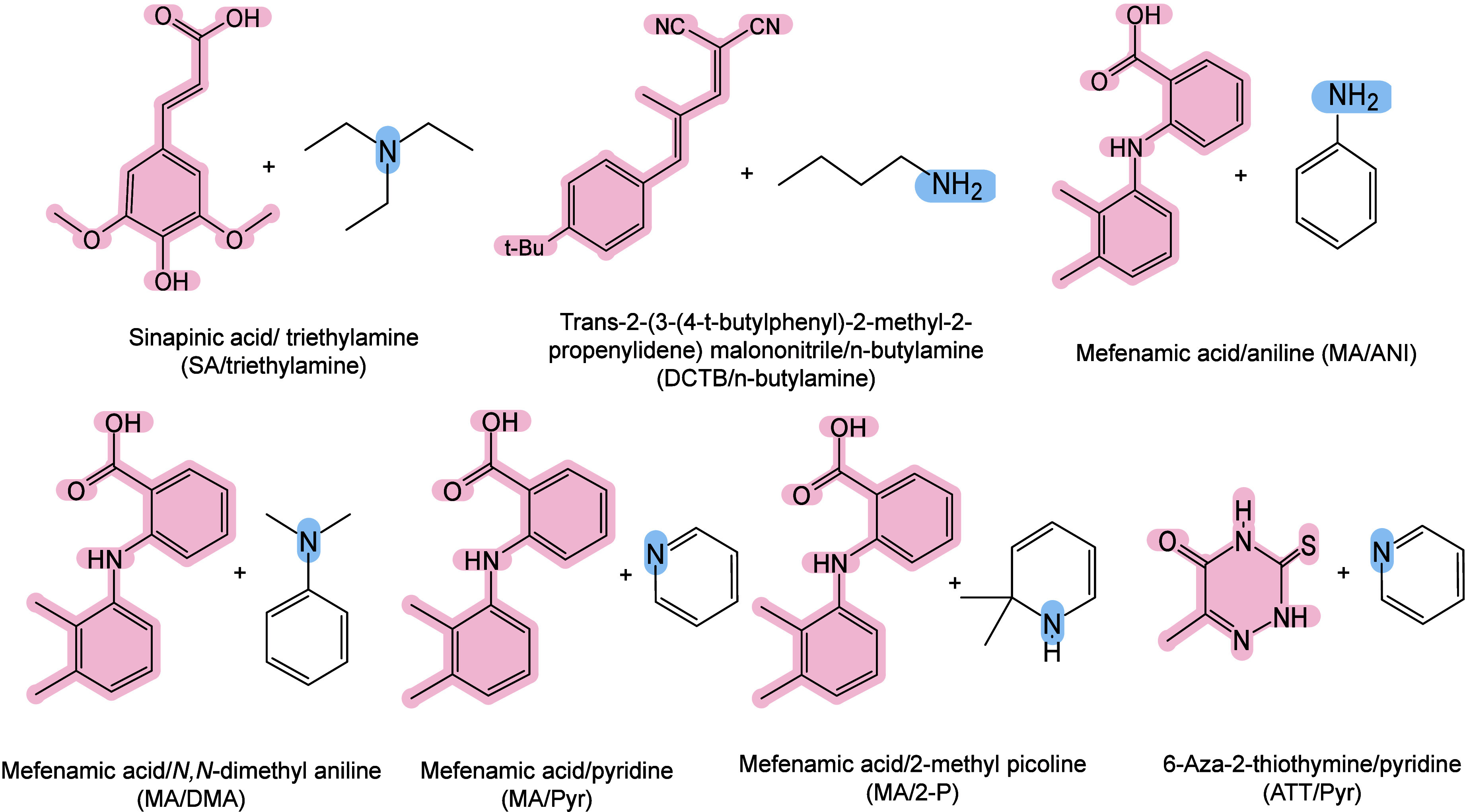
Chemical structures of other ionic liquid matrices (I).

**Figure 66:**
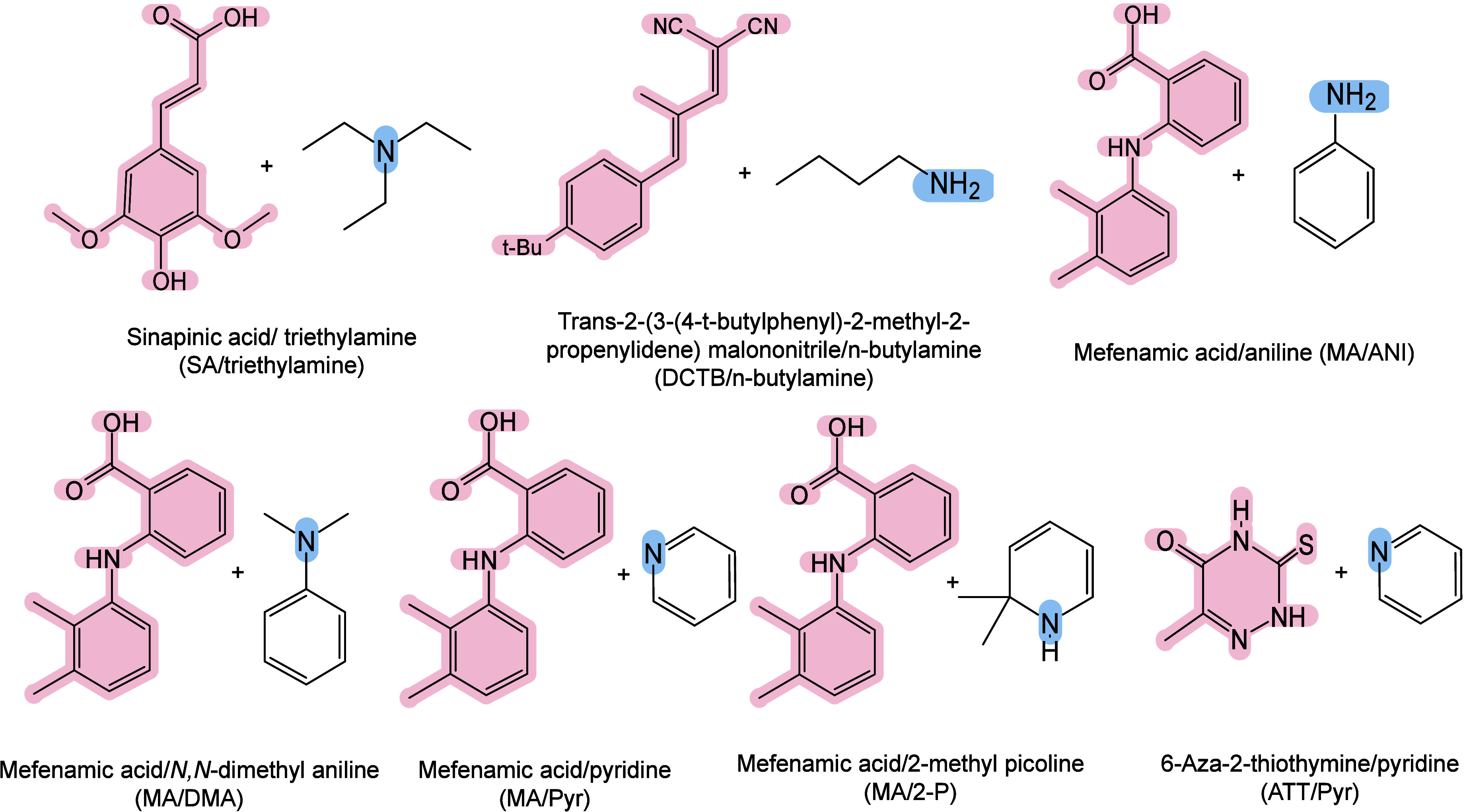
Chemical structures of other ionic liquid matrices (II).

###### 3.1.2.3.4 Summary

In summary, RTILs are an efficacious novel category of matrix for MALDI, and they can address the limitations of traditional matrices. ILMs can be used in MALDI-MS to analyze a diverse array of compounds, including polymers, oligonucleotides, peptides, proteins, lipids, oligosaccharides, and glycoconjugates. Furthermore, the use of ILMs has numerous benefits, including a more uniform mixing of the matrix with the analyte molecules, which results in enhanced sensitivity and reliable reproducibility. In MALDI analysis, ILMs exhibit high performance, with results that are comparable or even superior to those obtained with solid matrices. Due to their low vapor pressure, ILMs can be utilized as an attractive matrix for MALDI-MS under very high vacuum. Second, their liquid nature prevents altering the sample surface chemistry by cocrystallizing with the analyte, thus avoiding the formation of the “sweet spot” effect. Additionally, ILMs are composed of preformed ions, which minimizes the impact of mass disturbances caused by matrix fragmentation, particularly at *m*/*z* < 200 Da. In conclusion, the introduction of ILMs is anticipated to emerge as a novel research focus within the field of MALDI matrix development, offering a potential solution to some of the limitations associated with traditional solid crystalline matrices.

##### 3.1.2.4 Binary and Hybrid Organic Matrices

The concept of binary matrices was first proposed by Solouki et al.^623^ Their objective was to enhance ion yields in MALDI-FTICR MS analysis of biomolecules, such as bovine insulin and lysozyme, by investigating PNA/coumarin and fructose/DHB matrix mixtures. Other strategies have sought to leverage the synergistic effects generated by combining different components into a single matrix system. Such multicomponent systems include binary matrices (consisting of two conventional organic MALDI matrices) and hybrid matrices (consisting of an organic compound and an inorganic compound), the latter of which is typically based on an inorganic matrix.

###### 3.1.2.4.1 Binary and Hybrid Organic Matrices Based on DHB

In MALDI-MS analysis, the use of DHB-based binary and hybrid organic matrices represents a notable breakthrough. The complex chemical architectures of these matrices are shown in Figure 67, which highlight their adaptable design and potential for analytical advancements. Mechref and Novotny employed SPM as an auxiliary matrix with the objective of facilitating MALDI-MS analysis of acidic sugar conjugates.^624^ Their findings indicated that the use of DHB/SPM enhanced matrix crystal formation, increased sample solubility, and decreased the formation of base adducts. This approach enabled the rapid analysis of samples without the need for desalting. Soltwisch et al. reported that the use of DHB/glycerol as a binary matrix allowed the preparation of homogeneous samples for MALDI-MS (337 nm N_2_ laser), which facilitates the analysis of compounds that need to be stabilized in glycerol.^625^ By controlling the irradiation conditions, excellent reproducibility and good quantitative response were achieved for samples prepared using this matrix. Furthermore, compared to standard DHB matrix, the DHB/glycerol showed better peptide, protein, and oligosaccharide mass spectral signals. Kim et al. employed DHB/2,6-DHB as a MALDI matrix for the detection of polysaccharides and found that DHB/2,6-DHB exhibited lower background noise and higher sensitivity compared to the single-component matrix.^626^ Zhao and coworkers devised a novel reaction matrix, 2,5-dihydroxybenzoylhydrazine (DHBH), which was combined with the catalytic matrix, DHB, to create a combined matrix (DHB/DHBH) for the qualitative and quantitative analysis of *N*-glycans by MALDI-MS. The results demonstrated that the acid-base chemistry of DHB/DHBH facilitated uniform crystallization of the analyte–matrix mixture and markedly enhanced the ionization efficiency of reduced carbohydrates.^627^ Schroter et al. investigated the potential of a combination of DHB and 2,5-DHAP matrices (DHB/2,5-DHAP) for the detection of PEs in complex mixtures.^628^ The use of this matrix permitted the unambiguous identification of PEs in complex mixtures and tissue sections, obviating the necessity for prior separation of individual lipids prior to MALDI-MS detection. Lavanant and Loutelier-Bourhis analyzed oligosaccharides utilizing either procaine or procaine amide as a derived co-matrix for DHB.^629^ Their findings revealed that the mixed DHB/procaine matrix was capable of producing exceptionally fine and homogeneous crystallization, obviating the necessity to search for “sweet spot”. Furthermore, the S/N ratio could be enhanced, and the high abundance of cocaine or procaine amide on the target did not impede ionization process. Urakami and Hinou used the DAN/DHB/K^+^ matrix for MALDI O-antigen glycan analysis of *Yersinia pseudotuberculosis*.^630^ By employing the DAN/DHB/K^+^ matrix, the O-antigen repeat units were detected in the form of potassium adducts, which, compared to traditional sodium ion-based matrix systems, facilitates the differentiation of hexoses, deoxyhexoses, and dideoxyhexose. Owing to the selectivity of sugars and the uniformity of adduct ions in the ionization process, the DAN/DHB/K^+^ matrix provides a high S/N ratio O-antigen polysaccharide-derived signal pattern, thereby improving the accuracy of MALDI-based O-antigen glycosylation identification. Yamaguchi et al. were the first to develop alkylated hydroxychalcone/DHB (AHC/DHB) as a novel binary matrix and to employ MALDI-MS to analyze hydrophobic peptides.^631^ The AHC matrix contains alkyl chains analogous to those of ATHAP, and its chalcone structure demonstrates high absorption at a wavelength of 355 nm, rendering it suitable for the analysis of hydrophobic analytes. For traditional hydrophobic analytes, AHC has been demonstrated to demonstrate performance comparable to that of ATHAP. However, AHC has suboptimal crystallinity, which compromises the reproducibility of the samples. When AHC is combined with DHB as a binary matrix, the crystallinity of AHC is significantly improved, thereby enhancing the reproducibility and increasing the sensitivity for hydrophobic peptides. The results of the MALDI-MSI study suggest that these enhancements can be attributed to an augmented number of “sweet spots” within the matrix/analyte cocrystals, where the analytes are efficiently detected as ion peaks.

**Figure 67:**
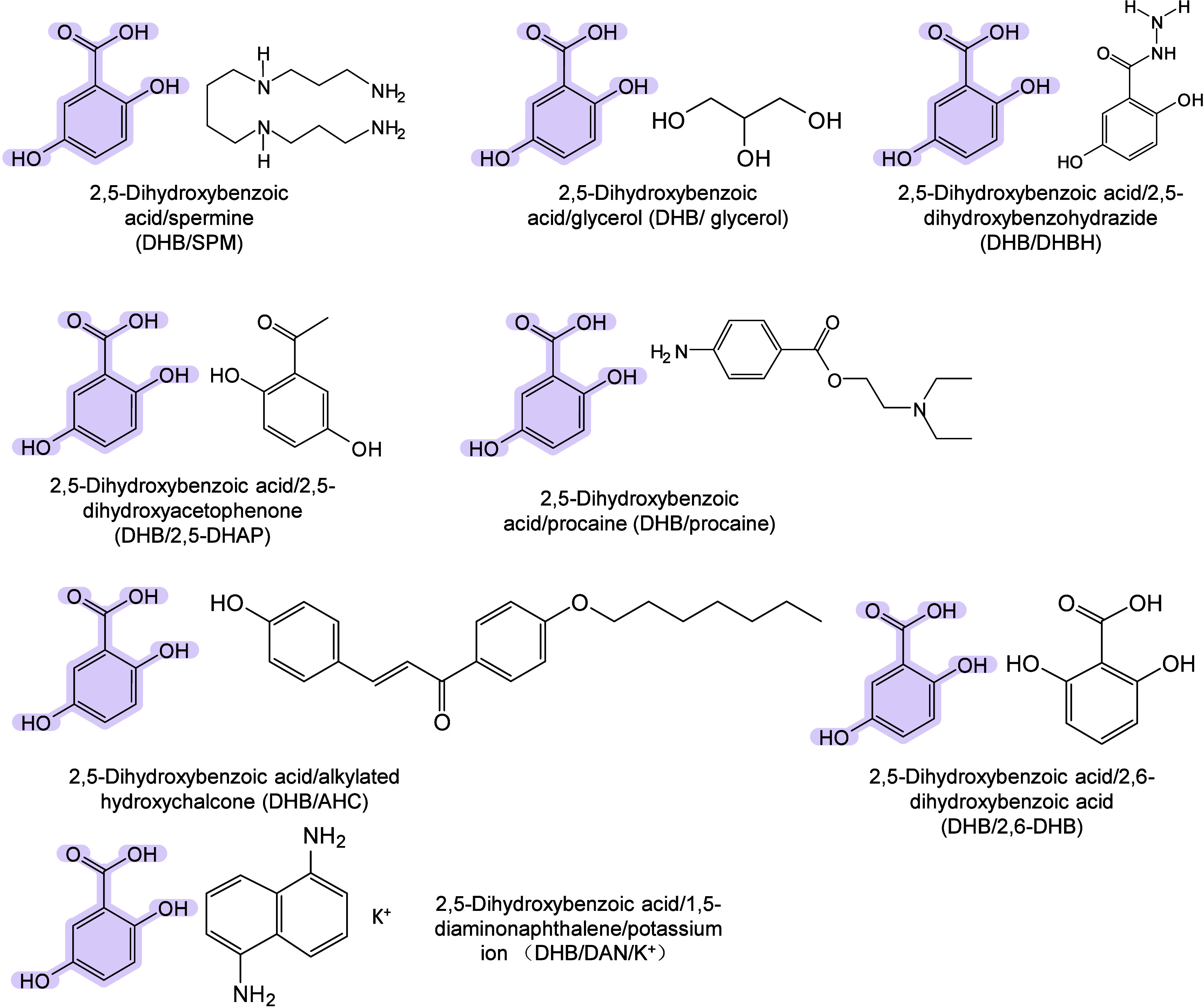
Chemical structures of binary and hybrid matrices based on DHB.

###### 3.1.2.4.2 Binary and Hybrid Organic Matrices Based on CHCA

In the quest for cutting-edge matrix-assisted analytical methods, CHCA-based binary and hybrid organic matrices have become pivotal research focuses. The chemical architectures of these novel matrices are shown in Figure 68, which highlights their structural complexity and potential. Hsieh and Tam employed a combination of CHCA and nitrocellulose (CHCA/nitrocellulose) as a matrix for the detection of dimethylarginine in protein hydrolysates via MALDI-MS.^632^ The results demonstrated that nitrocellulose can attenuate the matrix signal of CHCA, thereby facilitating the observation of an ion peak indicative of dimethylarginine on the mass spectrum. Guo and He reported that the use of acidic CHCA and basic 9-AA (CHCA/9-AA) as binary matrices can effectively suppress the background signal of small molecules in MALDI-MS detection.^633^ The number of matrix background peaks was reduced in both positive and negative ion mode detection of small molecules. Furthermore, the presence of CHCA significantly decreased the laser energy required for analyte desorption and ionization. Zhou et al. demonstrated that the detection of phosphopeptides by MALDI-MS could be enhanced through the use of a binary matrix (CHCA/3-HPA).^634^ In comparison with classical CHCA or DHB, CHCA/3-HPA markedly augmented the ionic signals of phosphopeptides in both positive and negative ion detection modes. Moreover, elevated phosphopeptide signal intensities were acquired at reduced laser powers. Qiu et al. developed a novel binary matrix, CHCA/*p*-phenylenediamine (PPD), for enhanced MALDI-MSI analysis of antibiotics in *Ctenopharyngodon* tissues.^635^ Compared with the conventional matrix, CHCA/PPD significantly increased the detection intensity of antibiotic drugs with better sensitivity and reproducibility. Laugesen and colleagues discovered that blending the two matrices, CHCA and DHB, markedly improved the detection efficacy of peptide mass mapping and protein analysis. It enhanced the coverage and reproducibility of peptide mass mapping, facilitated protein identification and automated data acquisition, and exhibited greater tolerance to salts and impurities.^636^ Shanta and colleagues demonstrated that CHCA/DHB can be employed for the MALDI-MSI analysis of PLs in both ion modes. This matrix composition exhibited vacuum stabilization properties, higher signal intensity, and greater reproducibility than conventional matrices and was capable of identifying a greater number of PLs.^637^ The characterization of phosphorylated amyloid beta (Aβ) peptides is challenging at all levels, from sample preparation to mass spectrometric analysis. In general, the phosphoester bond may be hydrolyzed under the harsh conditions that are required for the solubilization of aggregated Aβ.^638^ To address the technical challenges associated with the analysis of intact phosphorylated Aβ peptides by MALDI-TOF-MS, Liepold et al. chose to enhance the MS response via dopants rather than selective enrichment of phosphopeptides. They developed TOPAC as a customized matrix formulation that allows for the sensitive detection of intact phosphorylated Aβ species.^639^ TOPAC matrix formulation, which includes the nonionic detergent *n*-octyl-β-d-glucopyranoside (OGP), PA, and the commonly used peptide matrix compound CHCA, will facilitate the characterization of phosphorylated Aβ species by MALDI-TOF-MS at the level of both the intact Aβ peptides and their proteolytic cleavage products. They further propose it as a valuable tool for future studies aiming for the mass spectrometric verification of phosphorylated Aβ peptides in brain samples and for revealing their exact molecular identity. The TOPAC matrix may be helpful for addressing this challenge, as it has also shown improved performance for the mass spectrometric detection and sequencing of proteolytic cleavage products of phosphorylated Aβ peptides.

**Figure 68:**
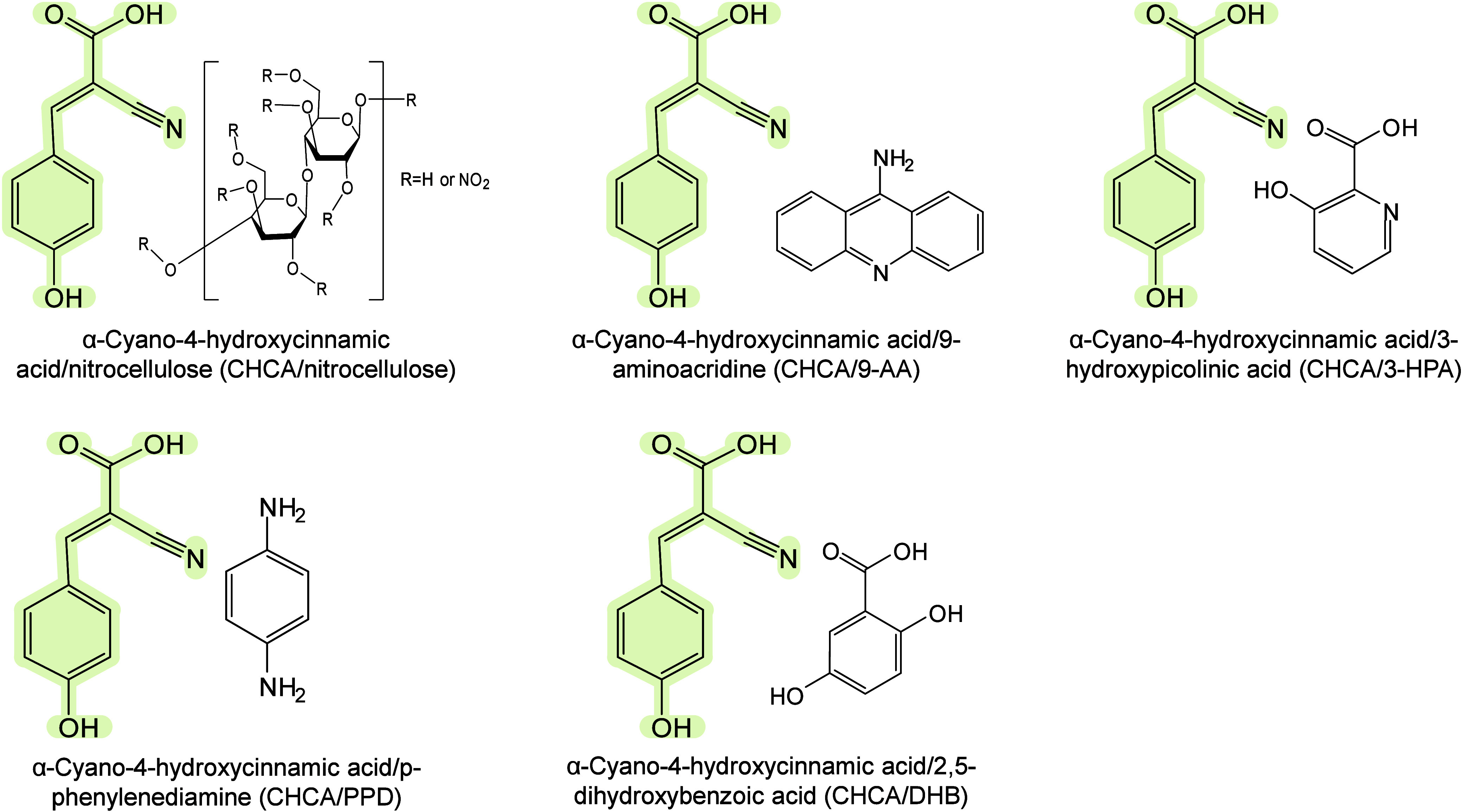
Chemical structures of binary and hybrid matrices based on CHCA.

###### 3.1.2.4.3 Other Binary and Hybrid Organic Matrices

Binary and hybrid organic matrices have attracted significant attention as promising contenders, boasting distinctive chemical attributes and interactions. In this context, Figure 69 offers a thorough depiction of the chemical structures of alternative binary and mixed matrices, illuminating their prospects for advancing the development of analytical methods. Zhu et al. used a matrix comprising a mixture of 2,3,4-THAP and 2,4,6-THAP for the detection of DNA by MALDI-MS. They observed that the matrix with a molar ratio of 2:1:1 of 2,4,6-THAP, 2,3,4-THAP, and AC (2,3,4-THAP/2,4,6-THAP/AC) was particularly effective for the detection of DNA, particularly in instances where the DNA content was low.^306^ The hybrid matrix provided better resolution and reproducibility than the 3-HPA and 2-PA matrices, and had reasonably favorable sensitivity for DNA detection. The hybrid matrix 3-HPA/pyrazinecarboxylic acid (PCA) for oligodeoxynucleotide MALDI-MS analysis was discovered by Zhou et al.^640^ When DNA fragments were analyzed using 3-HPA/PCA, the intersample reproducibility, resolution, S/N, and tolerance to metal salts were superior to those of the 3-HPA matrix alone under the same experimental conditions. In a recent study, Shanta et al. identified a novel MALDI matrix combination, 3-HC/ATT, which they demonstrated to be suitable for MSI analysis of drugs and metabolites, including individual AAs.^641^ Calvano and colleagues developed a novel binary matrix, DMAN/9-AA, for the analysis of intact bacterial lipid fingerprints by MALDI-MS.^642^ The mass spectra obtained with DMAN/9-AA exhibited a higher S/N ratio, lower chemical noise, and an absence of matrix cluster formation compared to those obtained with a single matrix, indicating the potential of this binary matrix to improve sensitivity. It has been demonstrated that the utilization of 9-AA or NEDC as a MALDI matrix results in weak ISD, which is crucial for the analysis of complex biological samples. Wang et al. observed that through the optimization of the matrix mixing strategy, the NEDC/9-AA mixture has the capacity to facilitate the analysis and mapping of all major classes of PLs and sulfolipids and has led to an enhancement in the lipid analytical capability and imaging coverage of MALDI-MS.^643^ Yang et al. introduced a novel binary matrix that is suitable for the MALDI-MS analysis of LMW compounds.^644^ It was discovered that rhodamine (R) 575, a UV-absorbing candidate operating at 532 nm, was capable of binding to proton donors or proton acceptors, thereby enhancing the ionization efficiency of analytes. Their results demonstrated that R 575, in conjunction with an acid (*e.g.*, HCl or CHCA) or a base (*e.g.*, NaOH), such as R 575/CHCA, could form an efficacious binary matrix and be employed for the detection of LMW alkaline and acidic compounds in both positive and negative ion modes.

**Figure 69:**
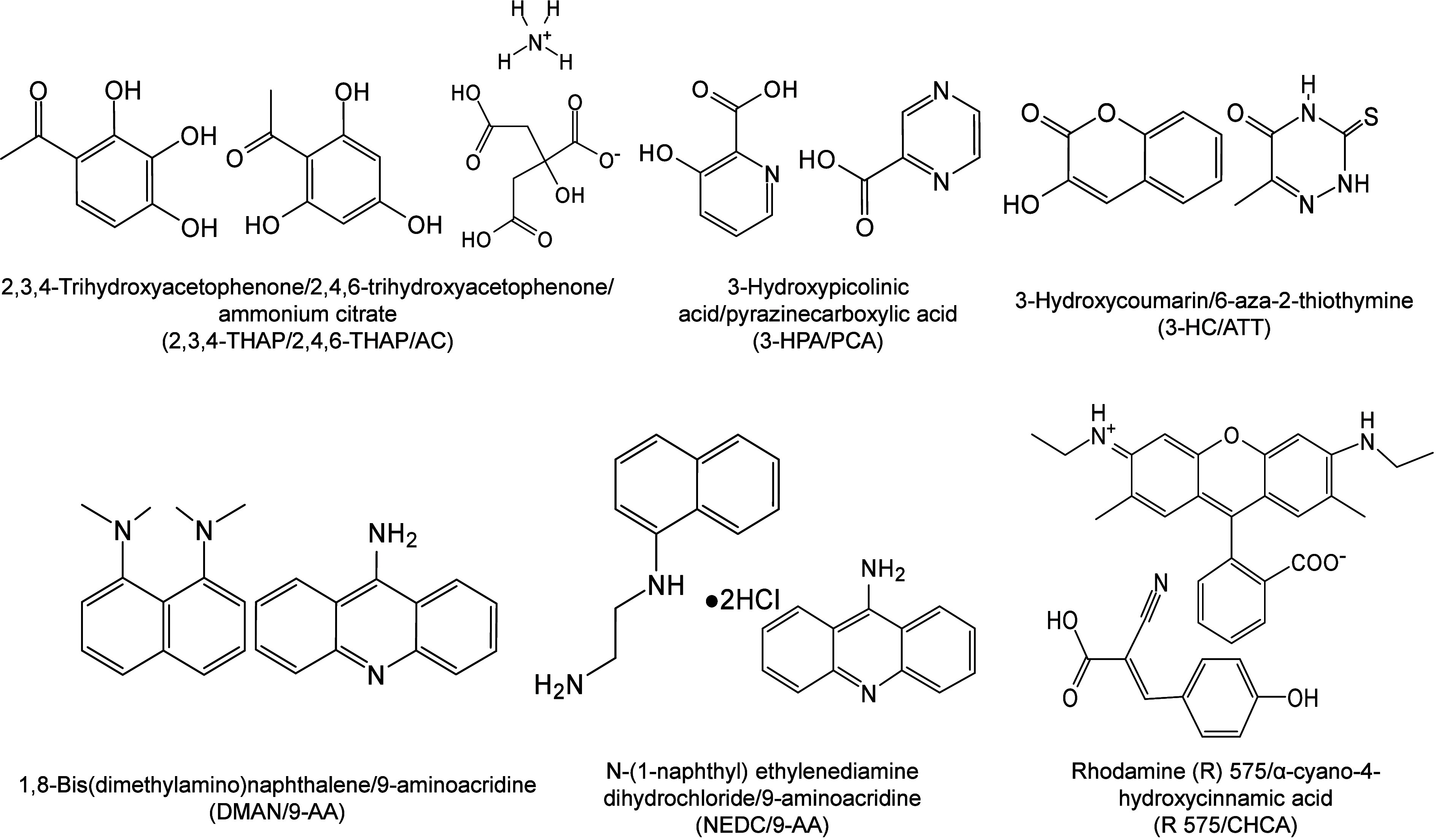
Chemical structures of other binary and hybrid organic matrices.

###### 3.1.2.4.4 Summary

In summary, the use of “binary or multicomponent matrices” enhances the overall detection efficacy of the analyte while concomitantly reducing background noise. By optimizing the physicochemical properties of the matrix, such as crystallinity and light absorption, binary or hybrid matrices can augment the desorption and ionization efficiency of the sample under laser bombardment, thereby improving the detection sensitivity. Binary or hybrid matrices result in a reduction in background interference peaks compared with those present when a single matrix is used, thereby facilitating the detection of the object under measurement. Furthermore, the implementation of binary or hybrid matrices can enhance the detection of specific types of samples, including multicharged and difficult-to-ionize samples. In conclusion, the utilization of MALDI binary matrices optimizes the sensitivity and efficacy of the detection of specific samples by combining the advantages of two or more matrix materials.

#### 3.1.3 Dopants in Organic Matrices

Dopants are important components in matrix solutions and are typically used to improve analytical results including sensitivity, reproducibility, resolution, and signal suppression of desorption. Dopants can improve MALDI-MS analysis results in some cases. The matrix alone sometimes has no signal, and the addition of dopants can yield an ideal map. Currently commonly used dopants include ammonium salts, organic amines, carbohydrates, acidic dopants, and other dopants (Table S5).

##### 3.1.3.1 Ammonium Salts

Ammonium salts are important dopants. Because excessive cations in a sample can strongly affect the experimental results, it is usually necessary to clean the sample using appropriate solvents to improve the experimental results. However, the cleaning steps are complex and can dilute the sample, making it difficult to detect low-concentration macromolecules such as proteins. If the concentration of impurity cations in the sample solution is low, the addition of ammonium compounds can replace the cleaning step and improve the spectral quality. Ammonium-based dopants are mainly used for the determination of DNA and oligonucleotides, and some are also used for the determination of phosphorylated peptides. Common inorganic ammonium compounds include halide ammonium salts, ammonium dihydrogen citrate, and ammonium acetate.

Cheng and Chan used halogenated ammonium salts as co-matrix for MALDI-MS analysis of oligonucleotides. They tested four ammonium salt dopants (NH_4_F, NH_4_Cl, NH_4_Br, and NH_4_I) and used ANP matrix for MALDI analysis of various DNA polymers. All the halogenated ammonium salts significantly enhanced the signal intensity of the complete molecular ion of the DNA polymers to different extents, with NH_4_F showing the greatest enhancement effect. The signal enhancement effects of different halogen ion species on oligonucleotides differed.^407^ Holbrook et al. used ammonium fluoride as a dopant to enhance lipid signals in MALDI-MS. Compared with the NEDC matrix, the addition of NH_4_F significantly increased the sensitivity of lipid detection in both positive and negative ion modes of the MALDI system and could be used for various applications.^645^ Yamagaki et al. found that the interaction between NH_4_Cl and β-carboline biological alkaloid (7-methoxy-1-methyl-9*H*-pyrido­[3,4-*b*]-indole) matrix could promote the generation of chlorinated neutral oligosaccharide molecules in negative ion MALDI-MS.^646^ Kang et al. reported that adding diammonium citrate (DAC) to a CHCA matrix could increase the sensitivity of phosphorylated peptide detection in cell and tissue lysate dephosphorylation reactions, and improve the ability to distinguish background noise.^278^

In summary, ammonium compounds have many advantages as dopants in MALDI-MS. The anions of ammonium compound dopants play important roles in the D/I of oligonucleotides. In addition, ammonium compounds can noncovalently interact with the molecules in the sample to form stable ion-matrix complexes during the MALDI process, which helps to improve the stability and sensitivity of the mass spectrum signal. Ammonium compounds as MALDI dopants can significantly improve the sensitivity, stability, and accuracy of MALDI-MS by absorbing light energy, promoting ionization, forming stable ionic complexes, and improving solubility and volatility.

##### 3.1.3.2 Organic Amines

The high proton affinity of organic amines promotes a proton transfer reaction, which is conducive to the formation of ionized molecular ions. Moreover, organic amines can form hydrogen bonds or ion-molecular complexes between sample molecules and the matrix, promoting ionization reactions and increasing the strength and stability of the mass spectrum signal.^647,648^ Therefore, organic amines can effectively improve the sensitivity and reliability of MALDI-MS and support the structural and compositional analysis of biological macromolecules. Arginine (Arg), Arg hydrochloride, Arg hydrochloride, Arg hydrochloride, and dimethylformamide are commonly used organic amine dopants.

Among them, SPM exists in organisms as a regulator and has regulatory effects on the secondary and quaternary structure of DNA.^649^ Protonated SPM has chemical properties similar to those of ammonium ion. Polyprotonated SPM can better replace basic cations in neutral oligonucleotides.^624^ Compared with other compounds such as indole, piperidine, triethylamine, oxamine, and adenine riboside, Arg and spermidine (SPD) have higher proton affinity. However, when Arg is used as a dopant, an SPD adduct peak is produced, whereas when spermidine is used, less adduct production occurs. In addition, Arg and SPD have a high affinity for the phosphate skeleton, which can stabilize DNA molecules and take away excessive energy during desorption.^650,651^ Vandell et al. reported that the use of polyamine co-matrices with polyamino-functional sites, such as spermine tetrahydrochloride (SPM-4HCl), SPD, spermidine trihydrochloride (SPD-3HCl) can significantly improve the mass spectrum quality of oligonucleotides, including molecular ion resolution and abundance.^652^ MS data revealed that compared with the monofunctional amine comatrix, the polyamine comatrix was more efficient in the MALDI-MS analysis of oligonucleotides. Asara et al. successfully improved the detection of oligonucleotides by ultraviolet MALDI-MS using tetraamine spermine (TETA-SPM) as a dopant.^648^ They repoted that TETA-SPM can effectively remove the adsorbed cations, reducing the detection limit of DNA in the MALDI-MS experiment without the need for a desalination step. Protonated Arg is chemically similar to ammonium ion in that it can bind to the phosphate skeleton and release protons. Furthermore, multi-protonated Arg molecules are superior to ammonium ions in neutralizing oligonucleotides and replacing base cations. In addition, Bashir et al. studied MALDI parameters and explored the effects of solvents and auxiliary dopants on the peak strength of the analytes.^653^ The peak intensities of substance P and β-cyclodextrin were measured by using DHB as matrix. DMF was shown to reduce the number of hydrogen bond interactions between DHB molecules, thus reducing the laser energy requirement and increasing the peak intensity of substance P and β-cyclodextrin.

In summary, amines are commonly used as dopants in MALDI-MS. Amines such as piperidine, imidazole, and triethylamine can improve MS quality by increasing molecular ion resolution and abundance. Their features include the formation of hydrogen bonds or other noncovalent interactions with sample molecules during photodesorption, which helps to desorb and ionize the sample molecules. Therefore, they are very important for helping to obtain more accurate and reliable MS data on complex samples, thereby promoting the development of scientific research and application fields.

##### 3.1.3.3 Carbohydrates

Carbohydrates have been successfully used as dopants for MALDI analysis because they are generally soluble and stable and can form a uniform mixture with the analyte, helping to improve the co-crystallization of the matrix and the analyte. Additionally, the ionization efficiency of carbohydrates under laser irradiation is appropriate to promote analyte ionization, and their relatively low background interference can improve the sensitivity and accuracy of MS analysis. Among them, fucoidan, sorbitol, and cyclodextrin are often used as MALDI dopants.

Distler and Allison used fucose as a dopant to improve the MALDI-MS analysis of oligonucleotides.^654^ Upon binding to the matrix, trehalose can improve the uniformity, signal intensity, and signal duration of the MALDI target, thereby increasing the sensitivity of the experiment and improving the detection of the components of complex oligonucleotide mixtures. Moreover, the addition of trehalose can also reduce the fragmentation of oligonucleotides. Billeci and Stults mapped the trypsin digestion profile of recombinant proteins by MALDI-MS. Compared with the single matrix, the spectral quality of the trehalose/DHB composite was significantly improved, including suppression of the matrix peak, an increased ion signal, and improved resolution.^655^ The tolerance of trehalose/DHB to ion contaminants was also enhanced. Loo and Loo used formic acid (FMA), perfluorooctanoic acid (PFOA), and sorbitol as dopants for the MALDI-MS analysis of hydrophobic proteins in mixtures.^656^ The advantage of the use of sorbitol as a dopant lies in its lower reactivity and lack of formation of Schiff bases with amines, so it may be more suitable as a dopant than trehalose or fructose in certain cases. The results show that adding sorbitol to the SA matrix can promote uniform crystallization and enhance the ion signal of analyte. In addition, Yamaguchi et al. investigated the use of cyclodextrins to inhibit matrix-associated ions by MALDI-MS and reported that cyclodextrin-loaded matrices can effectively inhibit the matrix-associated ions and alkali metal ion adducts.^657^ By using typical organic matrices such as THAP and CHCA in cyclodextrin cavities, they successfully measured the mass peaks of only protonated matrix ions and significantly reduced their intensity and fragmentation, while simultaneously analyzing the mass peaks of analyte molecules such as substance P and adenosine without matrix interference.

In summary, carbohydrates as a highly effective dopant play a significant role in MALDI analysis. Compared with traditional one-component matrices, carbohydrate-containing co-matrices show superior results in many aspects. From the perspective of crystallization characteristics, carbohydrates can improve the crystallization uniformity of the matrix and analyte, and promote the formation of a more favorable structural arrangement for detection and analysis at the microscopic level. Carbohydrates can also significantly increase the ion signal intensity of the analyte, providing a strong guarantee of high detection sensitivity. Moreover, carbohydrates can inhibit matrix-related ions. The shielding and interference effects of background peaks on analyte signals were decreased by reducing background peak interference, thus greatly increasing the accuracy and reliability of analyte signal detection and laying a solid foundation for accurate analysis.

##### 3.1.3.4 Acidic Dopants

The acidic or alkaline properties of a dopant affect the ionization process of the analyte. The alkaline dopant favors deprotonation of the analyte in solution, *i.e.* the Lucky Survivor process whereas the acidic dopant favors gas phase deprotonation, *i.e.* the secondary ionization of the analyte after deprotonation in the matrix.^570,658^ Acid compounds are often used as dopants for MALDI-MS because of their multiple functions in MS. First, acid compounds can promote the ionization of molecules in a sample, making them easier to detect by MS. Second, acid compounds can improve the intensity and stability of the mass spectrum signal and enhance the clarity and resolution of the mass spectrum peak. In addition, they can reduce the background noise of the mass spectrum signal and improve the sensitivity and accuracy of the mass spectrum analysis.^659,660^ Therefore, acidic MALDI dopants play important roles in improving the effectiveness and reliability of MALDI-MS results. Acid MALDI dopants mainly include TFA, phosphoric acid (PA), and nitrilotriacetic acid (NTA).

Zhou et al. reported that the addition of certain chemicals to the matrix can affect the mass spectrum signal of lipid A, especially phosphate, pyrophosphate, sulfate, oxalic acid, citric acid, and ethylenediamine tetraacetic acid (EDTA), which increase the signal of diphosphorylated species.^661^ Jabbar Siddiqui et al. investigated the effects of matrices and dopants on phosphorylation and ketodeoxyoctanoate lipid A by MALDI-MS analysis. They tested the effects of 11 dopants (seven ammonium salts and four acids) on the signal and reported that the best dopants for obtaining a clear signal of both lipids A without immediate fragmentation were 3-HPA and TFA.^662^ Andrade et al. tested atrix/solvent systems with different TFA contents for the analysis of lyophilized microalgae crude preparations by MALDI-MS. The best results in terms of S/N, protein number, and signal strength were obtained when the TFA content was 0.1% in a matrix solvent.^663^ Since there is no need for chemical or enzymatic conversion and no need to choose chromatographic methods, the use of dopants or comatrices to enhance phosphopeptide reactions in MALDI-MS is very attractive. Sven et al. reported that PA can be used as an effective dopant for the MALDI-MS analysis of phosphopeptides and phosphoproteins, especially when DHB is used. PA significantly enhances the phosphopeptide ion signal. Combining PA with DHB not only improves the signaling of phosphopeptides in the crude peptide mixture of phosphorylated proteins (alpha-casein and beta-casein), but also significantly improves the mass resolution of intact proteins. Similarly, through digestive studies of protein standards and plasma samples, Park et al. found that the addition of PA to the DHB matrix significantly increased the peptide ion signaling of phosphopeptides and nonphosphopeptides in MALDI-MS through digestive studies of protein standards and plasma samples.^664^ Kuyama et al. used methanediphosphonic acid (MDPNA) as a dopant and sensitively detected phosphopeptides by the MALDI-MS.^665^ They reported that adding low concentrations of MDPNA to the matrix 2,4-DHBA analytical solution significantly enhanced the signaling of phosphopeptides in both positive and negative ion modes, while eliminating the signaling of alkali metal ion adjuncts such as [M+Na]^+^ and [M+K]^+^. In addition, Kim et al. discovered a non-UV-absorbing chelating agent, NTA, which can improve protein identification by enhancing peptide signaling and the number of peptides observed from several proteins at different concentrations by MALDI-MS.^666^ They reported that adding NTA to the matrix solution significantly reduced the matrix clusters and increased the S/N of the peptide signal by about 5–20 times. In addition, Fukuyama's team reported a novel dopant, ADHB, for improving the sensitivity of MALDI-MS for the detection of hydrophobic peptides.^570^ ADHB is a DHB derivative in which a hydrophobic alkyl chain has been added to the hydroxyl group, thereby increasing its affinity for hydrophobic peptides and improving the detection sensitivity of MALDI-MS. By introducing hydrophobic alkyl chains into the DHB matrix, ADHB enhances the affinity for hydrophobic peptides, allowing for their accumulation at the edge of the matrix/analyte dry spot. Loo and Loo used FMA, perfluorooctanoic acid (PFOA) as dopants for the MALDI-MS analysis of hydrophobic proteins in mixtures.^656^ Formic acid significantly enhances the detection of hydrophobic proteins. Furthermore, the incorporation of perfluorooctanoic acid further improves the solubility and crystallization quality of hydrophobic proteins in the MALDI matrix, thereby increasing the detection efficiency of high-mass-range ions. This provides a powerful new tool for analyzing hydrophobic proteins in complex biological samples.

In summary, acid compounds play important roles in MALDI-MS. These compounds are commonly used as dopants to improve the ionization efficiency and signal intensity of the analyzed sample. They can eliminate the alkali metal adduct signal, increasing the sensitivity and accuracy of sample analysis. Therefore, adding an appropriate amount of acid compounds as an adjunct to the MALDI matrix is a common strategy for improving the performance and reliability of MALDI-MS.

##### 3.1.3.5 Salt Compounds

Salt compounds are commonly used as dopants in MALDI-MS. The main function of salt dopants is to assist in ionization, helping the sample molecules to be more easily struck by the laser and produce ions.^310^ Typically, these salt compounds form non-covalent interactions with molecules in the sample, converting them into ionic forms. This helps to increase the sample’s signal intensity and improve the sensitivity and resolution of MALDI-MS.^667,668^ Common salt compounds used as MALDI dopants include silver salts, copper salts, cesium chloride (CsCl), lithium salts, nitrate salts, and alkyl sulfate salts.

In MALDI-MS technology, the use of silver and copper salts as dopants has significantly enhanced the analytical efficiency of complex organic compounds, leading to new breakthroughs in mass spectrometric analysis of various types of compounds. Apicella et al. investigated the performance of a silver trifluoroacetate (AgTFA) and dithranol composite matrix system in the analysis of silicone oils. They found that silver salts effectively enhanced the desorption/ionization efficiency of silicone oligomers by forming stable metal-organic ion complexes. The silver salt dopant optimized laser energy absorption and ion transport, enabling precise analysis of siloxanes and providing a reliable technical approach for molecular weight distribution and polymerization degree analysis of silicone materials.^669^ Given the outstanding performance of silver salts in specific analyses, Choi et al. explored the effect of different types of silver salts (including silver nitrate (AgNO_3_), silver benzoate (AgBz), AgTFA, and silver p-toluenesulfonate (AgTS)) as cationization reagents on the ionization efficiency of polybutadiene in MALDI-TOF MS analysis. The study found that the type of silver salt and the acidity of its conjugate acid significantly influenced the efficiency of [M+Ag]^+^ ion formation and the generation of silver cluster ions. AgBz, with a conjugate acid less acidic than DHB, reacted more effectively with DHB to form the [Ag­(DHB-H)] intermediate, thereby promoting the formation of [M+Ag]^+^ ions. In contrast, AgNO_3_ and other silver salts, due to their higher conjugate acid acidity or lower reactivity, resulted in weaker [M+Ag]^+^ ion signals or the generation of significant amounts of silver cluster ions.^670^ Silver salts not only demonstrate exceptional performance in polymer analysis but also play a unique role in the analysis of biomolecules. Meier et al. employed silver salts, particularly AgNO_3_, as a dopant to enhance the detection of unsaturated lipids using IR-MALDESI MS. By incorporating trace amounts of AgNO_3_ into the electrospray solvent, they significantly increased the ion abundance of unsaturated lipids, such as calcifediol, and reduced their detection limit by at least one order of magnitude. Silver ions (Ag^+^), due to their high affinity for the π-orbitals in unsaturated compounds, effectively facilitated the ionization of unsaturated lipids. Additionally, by leveraging the unique isotopic distribution pattern of silver (^107^Ag and ^109^Ag), they developed advanced peak filtering algorithms that improved the accuracy and confidence in identifying silver adduct peaks in complex organic compounds.^671^

In the field of polymer analysis, copper salts, in addition to silver salts, have shown promising applications. Deery et al. investigated the use of silver salts and copper salts as dopants in the MALDI and ESI mass spectrometric analysis of polystyrene. Both silver salts (such as AgNO_3_) and copper salts (such as copper chloride (CuCl)) effectively interact with polystyrene molecules to form stable metal-polymer adducts, thereby significantly enhancing the mass spectrometry signals. In MALDI experiments, the use of AgTFA or CuCl as dopant salts resulted in clear and reproducible [polystyrene+Ag]^+^ or [polystyrene+Cu]^+^ ion peaks. Notably, when using silver salts, the MALDI mass spectrum exhibited high-intensity, low-background noise, single-charge adduct peaks, indicating highly effective interactions between the silver ions and the polystyrene chains. Similarly, copper salts also demonstrated good adduct formation. These findings not only provide a new approach for the mass spectrometric analysis of polystyrene but also highlight the importance of metal salt dopants in optimizing the ionization efficiency of synthetic polymers.^672,673^ Different metal salts exhibit varying performance in mass spectrometric analysis. Keki et al. utilized CuCl as a dopant to enhance the cationization of polystyrene in MALDI-MS. CuCl, with good solubility in tetrahydrofuran (THF), effectively interacted with polystyrene molecules to form stable [polystyrene+Cu]^+^ adducts, thereby significantly improving the signal intensity and resolution of the MALDI mass spectrum. Compared to silver salts (such as AgNO_3_), CuCl maintained good signal intensity and resolution even after one month of sample storage, while silver salt samples showed a significant decline in signal intensity and resolution after only a few days. These findings suggest that CuCl, as a simple and cost-effective salt dopant, offers significant advantages in the MALDI-MS analysis of polystyrene.^674^ Patil et al. compared the performance of silver salts and copper salts as dopants in the MALDI mass spectrometric analysis of ultra-high molecular weight (UHMW) polystyrene. While silver salts have commonly been used in previous studies for the MALDI analysis of polystyrene, copper salts demonstrated significant advantages in the analysis of UHMW polystyrene. Copper salts not only effectively ionized UHMW polystyrene, with a detectable molecular weight range extending up to two million Da, but also allowed for flexible control of the ion charge state. This flexibility in charge state control simplified the analysis process and enhanced the method's versatility, making copper salts highly effective for analyzing both individual polystyrene samples and their mixtures. Moreover, copper salts exhibited good compatibility with the DCTB matrix, further enhancing their potential in polymer analysis.^675^

Schiller et al. used CsCl as an auxiliary reagent for MALDI-MS analysis of phosphatidylcholine mixtures and demonstrated that it could effectively overcome peak overlap problems.^676^ Since cesium is a pure element consisting of only one isotope and has a high atomic mass, it causes significant mass transfer of PLs, generating Cs^+^ complexes that are easily identifiable because of mass shift and thereby improving the clarity of the analysis. Lau et al. utilized sodium doping and capture ion mobility spectrometry methods to enhance lipid detection in a novel MALDI-MSI analysis of oats.^677^ During sample preparation, CHCA was selected over DHB and 9-AA as the matrix of choice for the detection of lipids in positive ionization mode. Poor detection of triacylglycerols (TAGs) was resolved by applying sodium chloride during mounting, increasing signal intensity. In combination with TIMS, lipid identification significantly improved, and several isobaric and isomeric lipids were separated with visualization of their “true” spatial distributions. Cerruti et al. performed MALDI-MSI of lipids in a matrix solution by adding five different concentrations of lithium salts (LiAc, LiTFA, LiCitrate, LiCl, and LiI).^668^ They proposed a new sample preparation method involving the use of lithium salts, which only detect lithium cationized molecules. This method simplifies MALDI-MS analysis and combines different lipid adducts into a single lithium cationized species. Among them, lithium iodide showed better results. Additionally, Domann et al. used THAP as a matrix and added ammonium nitrate (AN) as a salt to provide anionic species for the MALDI process to generate and fragment neutral N-linked carbohydrate anions, achieving optimal results.^678^ Griffiths and Bunch introduced nitrates as a useful salt dopant for MALDI-MS lipid analysis,^667^ adding sodium or potassium nitrate to the matrix to increase the sensitivity to sodium and potassium adducts, respectively. They reported that nitrates and potassium nitrate were effective in a certain concentration range, whereas lithium nitrate showed lower concentration dependence, enhancing its potential for this application. Furthermore, they introduced HSO_4_^–^ as an anion dopant, allowing the observation of neutral oligosaccharides in negative ion mode. HSO_4_^–^ has been proven to be a suitable anion dopant, but it has certain limitations, as it binds weakly to small oligosaccharides such as disaccharides. To find a more effective and universal anion dopant, Wong et al. tested alkylsulfonates as an anion dopant for MALDI-MS to quantify desialylation reactions, finding that it had a significant effect on the generation of negative ions for neutral oligosaccharides.^679^ Alkylsulfonates strongly interact with oligosaccharides to form quasi-molecular ions composed of oligosaccharides and desulfonated alkylsulfonates. In negative ion mode, these novel alkylsulfonates have better S/N ratios, sensitivities, and ease of preparation than traditional sulfate.

In summary, salt compounds are crucial as dopants in MALDI-MS that primarily increase the sensitivity and signal intensity of sample analysis while facilitating the ionization of target molecules. These compounds offer superior ionization capabilities, mitigate background noise, and increase the stability of signal peaks, thereby improving the accuracy and reliability of MALDI-MS data. Consequently, the judicious selection and application of salt compounds as MALDI dopants are vital for attaining high-quality MALDI-MS outcomes.

##### 3.1.3.6 Other Dopants

The continuous development and improvement of various MALDI dopants has greatly expanded the research field of MALDI-MS and promoted qualitative and quantitative studies of biomolecules. In addition to the five classes of compounds mentioned above, other compounds can also be used as effective dopants. Transferrin (Tf), cucurbituril (CB[6]), and black phosphorus (BP) all play important roles in MS detection as MALDI dopants.

First, Tf is useful as a MALDI dopant in specific scenarios for MALDI-MS detection of insulin. Kobayashi et al. improved the sensitivity of MALDI-MS for insulin by pre-mixing a CHCA matrix with Tf.^280^ In particular, the signal intensity of large peptides (such as glucagon) was significantly enhanced in the presence of Tf or bovine serum albumin (BSA), while small peptides showed no significant change; compared with large peptides, Tf and BSA had a more pronounced enhancement effect on proteins (such as cytochrome C), effectively increasing the sensitivity of MALDI-MS to large peptides and proteins. Second, carbonyl­[n]­urea (CB­[n]) can effectively enhance the signal of polyamine compounds in MALDI-MS. CB­[n] is generated by the condensation reaction between glycerol urea and formaldehyde, and it can specifically interact with the target molecule, similar to the mechanism of a lock and key, allowing the target molecule in complex organic samples to be precisely bound.^680–683^ Ding et al. used CB[6] to analyze polyamine compounds in plant microstructures and reported that the molecular recognition and mass transfer properties of CB[6] enable related ion signals to be transferred to high-mass regions (*m*/*z* > 1,000), effectively preventing signal interference from conventional organic matrices.^684^ This strategy not only improves the accuracy of complex sample analysis, but also helps to complete the entire analysis process quickly, facilitating high throughput. Finally, BP can be used as a MALDI dopant to enrich organic matrices and improve the performance of traditional matrices. BP is a highly stable allotrope that can form homoatomic supramolecular structures such as cages or layers, and its role in MS is likely related to its nanostructure.^685,686^ By adding BP particles to a standard MALDI matrix, Mandal et al. increased the MS intensity of specific AAs and peptides,^687^ possibly through interactions with aromatic moieties of these molecules. Furthermore, BP promotes the formation of small, well-ordered crystals of the sample on the MALDI plate, reducing inconsistency in matrix and analyte deposition. Compared with the matrix without BP, the MS spectra recorded using a matrix enriched with BP exhibit higher reproducibility and stronger peaks.

##### 3.1.3.7 Summary

In summary, MALDI dopants are compounds that are used in MALDI-MS analytical methodologies and are distinguished by their ability to increase the ionization efficiency and signal intensity of samples by MALDI-MS, diminish sample crystal structure and surface tension, and bolster chemical compatibility between samples and the matrix. The compounds frequently utilized as MALDI dopants can be categorized into five groups: ammonium salts, organic amines, carbohydrates, acids, and salts. Their primary roles include facilitating the absorption and ionization of sample molecules, enhancing the sensitivity and resolution of MALDI-MS and MALDI-MSI, minimizing background noise in MALDI-MS signals, and optimizing the quality and reliability of MALDI-MS data. The various dopants exhibit distinct application ranges. Ammonium salt compounds are employed for the detection of oligonucleotides, neutral oligosaccharides, and lipids. Organic amine compounds are used to detect DNA molecules. Carbohydrates serve in the detection of oligonucleotides and proteins, whereas acid compounds are applied for lipid and phosphopeptide detection. Salt compounds are used for the detection of lipids and central oligosaccharides. The utilization of MALDI dopants in MALDI-MS analysis enables more precise detection and identification of compounds within samples, thereby assuming a pivotal role in domains such as biomedicine, biochemistry, and materials science.

#### 3.1.4 Outlook

Recently, interest in using MALDI for compound analysis has increased, particularly because of the potential of MALDI-MS imaging. While the details of the ionization process remain unclear, the importance of the matrix is widely acknowledged. The development and identification of useful matrices has historically been, and continues to be, an empirical process. For a compound to serve as an effective matrix, it must possess characteristics such as vacuum stability and high absorption at the laser wavelength. This review section summarizes the application of SOM matrices, as well as matrix design and synthesis in MALDI-MS analysis, aiming to identify empirical patterns. SOMs can be classified into 13 categories based on the structure of the compounds, while matrix design and synthesis can be divided into reactive matrices, matrix derivatization, ILMs, and binary or hybrid organic matrices.

Organic matrices play a critical role in MALDI-MS by absorbing laser energy and converting it into ionization energy for analyte molecules, thus enabling MALDI-MS analysis. The design of functional groups is central to their performance, and directly influences the light absorption properties, chemical stability, and ionization efficiency of the matrix. SOMs, with their moderate molecular weight, excellent solubility, and notable volatility, have become essential components of MALDI-MS. Functional substituents such as hydroxyl, carboxyl, and cyano groups can significantly increase the matrix's protonation or deprotonation ability, allowing it to adapt to different ionization modes and sample types. Cinnamic acid derivatives (such as CHCA) and phenylacetone-based matrices (such as 2,4-DHAP) optimize ionization performance through the introduction of specific functional groups, enabling effective analysis of proteins, small molecules, and a wide range of other compounds, including carbohydrates and nucleic acids. The development of organic matrices focuses on the innovative design of functional groups to improve the detection of specific analytes. For example, thiazole-based matrices, with sulfur and nitrogen atoms in their heterocyclic structures, exhibit superior ionization efficiency and selectivity, making them particularly suitable for lipid and peptide analysis. Similarly, benzoic acid-based matrices (such as DHB) increase signal intensity in protein analysis through the synergistic effect of hydroxyl and carboxyl groups and have become essential tools for lipidomics and biological tissue imaging. Furthermore, the sensitivity of functionalized matrices (such as halogenated FA derivatives) can be optimized through chemical modification, extending their application potential for large molecule detection and the selective analysis of specific types of molecules.

Future studies on MALDI-MS matrices will focus on functionality and diversity, aiming to further optimize ionization performance through the introduction of functional groups while enhancing compatibility with complex biological samples. On the one hand, the development of non-acidic or neutral matrices (such as thiazole and thiophene derivatives) will cater to the need to detect acid-sensitive molecules. On the other hand, exploring more environmentally friendly and biocompatible matrix materials will better serve biomedical applications. Additionally, the development of multimodal matrices, combining improvements in crystal deposition uniformity and detection sensitivity, will offer more comprehensive solutions for metabolomics and histological analyses. This matrix design, which is based on innovative functional group structures, will not only drive breakthroughs in basic research using MALDI-MS but also accelerate its adoption in clinical diagnostics and industrial applications.

### 3.2 Inorganic Matrices

With the rapid advances in nanotechnology and nanoscience, inorganic matrices, particularly those based on NMs, have emerged as pivotal components in the field of MALDI-MS, providing a significant complement to traditional organic matrices. The initial use of NPs as a matrix was reported by Tanaka and colleagues in 1988, who employed cobalt NPs suspended in glycerol for the detection of polymers and proteins.^24^ Subsequently, Sunner et al. introduced micrometer-sized graphite powder as a matrix for the detection of peptides, and proposed the novel concept of surface-assisted LDI-MS (SALDI-MS).^66^ Following this, a diverse array of novel NMs with varying compositions, shapes, and sizes has been extensively explored for the development of efficient matrices. Notably, the SALDI-MS technique has also been referred to by other researchers as direct LDI-MS or “matrix-free” LDI-MS.^688^ For the purposes of this review, we define MALDI as a soft ionization method that encompasses the use of both organic and inorganic matrices, while LDI-MS specifically refers to MALDI-MS techniques that utilize inorganic matrices.

Although the mechanisms by which NMs function as MALDI matrices have not yet been fully elucidated, several factors make NMs an attractive matrix for MALDI-MS. (i) The small size of NPs (0.2–100 nm) results in a large surface area per unit mass, which is essential for enhancing reactivity, capacity, and sensitivity in biological analyses. At the nanoscale, their properties change dramatically, making them more effective than bulk materials for sample enrichment.^78^ (ii) NMs possess unique thermal, optical, and electrical properties. NMs themselves are not ionized under laser irradiation, leading to a significant increase in local temperature, which aids in the D/I of analytes and suppresses substrate interference.^689^ (iii) NM-assisted LDI occurs on the surface of NMs, without involving co-crystallization of analytes with the matrices, thereby simplifying the sample preparation process and improving sample homogeneity. (iv) Additionally, NMs are widely used as affinity probes to simultaneously enrich and separate target species, and the detection of coagulated and captured mixtures can be performed using NMs or organic molecules as matrices.^690^ To date, various types of NMs, including metallic NMs (Au, Ag, Co, and Pt), carbon-based NMs (fullerenes, carbon nanotubes, graphene, and graphene oxide), silicon-based NMs (porous silicon, silicon NPs, silicon nanowires, and NAPAs), organic frameworks and quantum dots, have been used as alternative matrices for the determination of analytes ranging from small organic molecules to biopolymers such as proteins and peptides (Figure 2 and Table S6).

#### 3.2.1 Metallic Nanostructures

Among the candidate materials for MALDI matrices, metal NMs have attracted widespread attention in MALDI-MS analysis for two primary reasons:^691^ (i) their excellent optical properties and the generation of hot electrons through surface plasmon resonance effects and (ii) their superior thermal performance. In addition to metal NPs, many metal oxide NPs exhibit stability and strong absorption in the UV region, effectively generating thermal carriers under laser irradiation. This enhances the desorption and ionization capabilities of analytes and makes them excellent MALDI matrices.^692^

##### 3.2.1.1 Metal Nanomaterials

The application of metal NWs in MALDI-MS is primarily focused on gold NPs (AuNPs) and silver NPs (AgNPs), as they exhibit the strongest electromagnetic field enhancement among metals.^691^

###### 3.2.1.1.1 Gold Nanoparticles

AuNPs are among the most significant MALDI matrices. AuNPs have a broad absorption spectrum from visible light to UV with a high absorption coefficient.^245^ Additionally, AuNPs possess unique properties, such as low heat capacity and high thermal conductivity.^693^ Owing to their low heat capacity, the temperature of both the analytes and the matrix can rapidly increase. Moreover, owing to their high thermal conductivity, the accumulated heat on the AuNPs dissipates into the surrounding environment within 10–50 ps, approximately 100 ps to 1 ns.^694^ The rapid heat accumulation and dissipation of AuNPs under laser irradiation have been utilized for the desorption and ionization of small molecules.

In 2005, McLean et al. first used AuNPs with diameters ranging from 2–10 nm as MALDI matrices and successfully detected various peptide compounds (such as substance P and small proteins) by MALDI-MS.^690^ AuNPs exhibited a certain degree of selectivity, with a preference for ionizing peptides containing phosphotyrosine (pTyr), while showing poorer performance for peptides containing phosphoserine (pSer) or phosphothreonine (pThr).^690^ Directly analyzing neutral carbohydrates is challenging because of the lack of acidic or basic functional groups in their structures. Su and Tseng utilized bare AuNPs as an auxiliary matrix to achieve efficient ionization analysis of neutral LMW carbohydrates in MALDI-MS and successfully applied this matrix to the analysis of monosaccharides, disaccharides, and cyclodextrins.^695^ Tang et al. developed a dual-imaging technique for visualizing the chemical distribution in physical model visualization and latent fingerprints (LFPs), as shown in Figure 70a.^696^ This technique integrates two key properties of AuNPs, namely surface plasmon resonance and laser D/I, enabling visualization of LFPs from the macroscopic to molecular scale. Furthermore, because of the strong interaction between gold and sulfur, AuNPs are commonly used for the highly selective recognition and *in situ* detection of sulfur compounds in biological fluids.^697^ Chiang et al. attempted MALDI-MS analysis of amino thiols using AuNPs of different sizes, and the results showed that unmodified 14 nm AuNPs effectively captured amino thiols.^698^ Additionally, Wei et al. proposed a method to increase the detection sensitivity of small analytes in MALDI-MS using plasmonic nanoshells to generate hot carriers.^699^ The results indicated that compared with Au nanorods and Au nanospheres, Au nanoshells within the UV optical range exhibited higher analysis sensitivity and lower detection limits, presenting unique advantages.

**Figure 70:**
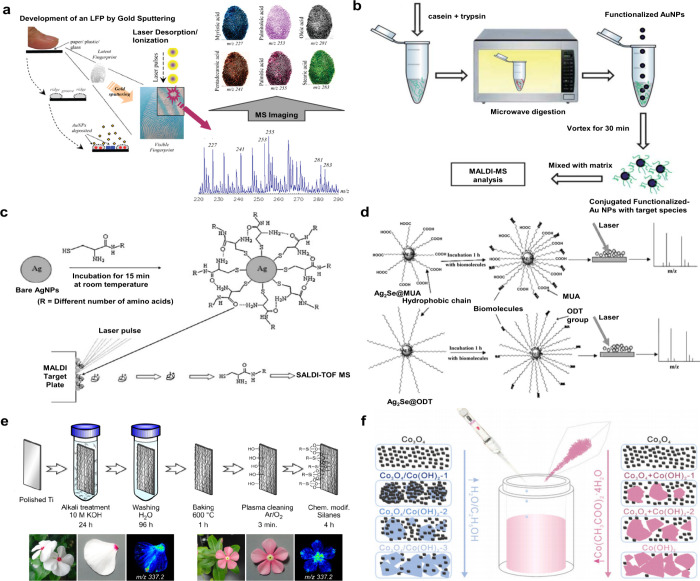
Metal NMs and metal oxide NMs as matrices for MALDI-MS. (a) Molecular imaging of latent fingerprints by MALDI-MSI using gold nanoparticles (AgNPs) as a matrix. Adapted and reproduced with permission from ref 696. Copyright 2010 American Chemical Society. (b) Schematic representation of microwave tryptic digests of casein proteins and their enrichment using dopamine dithiocarbamate functionalized gold nanoparticles (DDTC-AuNPs) as affinity probes. Adapted and reproduced with permission from ref 707. Copyright 2012 Royal Society of Chemistry. (c) Representation of AgNPs both as preconcentrating probes and as the matrix for the MALDI-TOF MS analysis of biothiols. Adapted and reproduced with permission from ref 714. Copyright 2008 John Wiley & Sons. (d) Schematic procedure for NP-LPME for hydrophobic peptide/protein analysis using functionalized silver selenide nanoparticles (Ag_2_Se NPs) as extracting probes followed by MALDI-TOF-MS. Adapted and reproduced with permission from ref 721. Copyright 2010 Elsevier BV. (e) Preparation protocol of a functionalized titanium oxide (TiO_2_) nanowire substrate and its applications for IMS of *C. roseus* petals and intact flowers. Adapted and reproduced with permission from ref 738. Copyright 2020 Society for Experimental Biology and John Wiley & Sons. (f) Synthesis of Co_3_O_4_/Co­(OH)_2_ composites with different proportions via a simple hydrothermal process. Adapted and reproduced with permission from ref 739. Copyright 2025 Elsevier BV.

Bare AuNPs tend to exhibit lower stability, are prone to aggregation, and can generate interfering background ions in the form of metal cluster species.^700^ Moreover, the synthesis of small, monodisperse AuNPs poses a significant challenge, as the driving force for particle aggregation increases. To overcome this, surface engineering techniques can be employed with AuNPs to achieve monodispersity. One approach involves the use of protective coatings or “capping” agents during synthesis to maintain the AuNPs in a finely dispersed state. Citrate has been widely used as a capping agent for AuNPs (citrate-capped AuNPs) in various applications. For instance, citrate-capped AuNPs have been employed as a matrix in MALDI-MS for the detection of progesterone, testosterone, and cortisol in high-salt solutions,^701^ for the detection of lipids in nonbiological or biological samples,^702^ and for the ionization and imaging of individual NT and endogenous NTs in biological samples.^703^ Hsieh et al. employed hexadecyltrimethylammonium bromide (CTAB)-adsorbed AuNPs (CTAB-AuNPs) as a novel matrix for the determination of nucleoside monophosphates by MALDI-MS, which resulted in a threefold increase in the ion signal compared with that of bare AuNPs, with no matrix-related ion peaks detected in the negative ion mode.^704^ Moreover, surface modification was utilized to enhance the affinity of the AuNPs for the improved detection and imaging of specific analytes. Huang and Tang achieved sensitive and reproducible detection of glutathione (GSH), cysteine (Cys), and homocysteine (HCys) in biological samples by using Nile Red-adsorbed AuNPs (NR-AuNPs) as selective probes and matrices in MALDI-MS.^705^ In another study, Huang and Tang used aptamer-modified AuNPs (Apt-AuNPs) as selective probes and MALDI matrices, and successfully analyzed ATP.^706^ Kailasa and Wu introduced a novel approach for the synthesis of DA dithiocarbamate-functionalized AuNPs (DDTC-AuNPs) using a one-pot method (Figure 70b).^707^ Remarkably, DDTC-AuNPs significantly increased the ionization efficiency of small molecular analytes such as AAs (GSH), drugs (desipramine and enrofloxacin), and peptides (valinomycin and gramicidin D), suppressed background noise, and served as affinity probes for the direct identification of phosphopeptides from casein proteins, demonstrating the potential for real-time analysis. Additionally, Goto-Inoue et al. developed and tested alkylamine-modified AuNPs (AuNPs-alkylamine) as a novel matrix, maximizing the detection of various glycosphingolipids (GSLs) such as sulfatides and gangliosides, and successfully applied them to detect GSLs in mouse brain sections.^708^ To mitigate the aggregation of AuNPs, Zhao et al. utilized hexagonal boron nitride nanosheets (h-BNNs) as supports to synthesize Au@BN, achieving high sensitivity and low background noise in MALDI-MS for detecting fipronil and its metabolites.^709^ This method also effectively imaged the spatial distribution of fipronil residues in strawberry and zebrafish slices, highlighting its potential for quantitative analysis of small molecules in complex biological samples. Recently, cellulose nanocrystals (CNCs) have been used as stabilizers for metal NPs, enhancing the dispersion and stability of AuNPs in aqueous media. Additionally, the interaction of CNCs with proteins reduces protein aggregation and decreases the resulting particle size. The interaction of AuNPs/CNC matrices with proteins increases the signal intensity and reproducibility of LDI-TOF MS, which was successfully applied to analyze intact proteins in serum exosomes from patients with non-small cell lung cancer, effectively distinguishing exosomal protein fingerprints between healthy individuals and patients.^710^

In addition to depositing or sputtering a thin and uniform layer of AuNPs on MALDI targets, AuNPs can be directly used as substrates for MALDI-MS analysis. For example, Liu et al. successfully developed Au nanoporous films (NPFs) as a simple and efficient background-free MALDI matrix.^711^ Cysteine-modified Au NPFs exhibited good thermal/electroconductivity and uniformity, allowing for the detection of various analytes such as AAs, drugs, peptides, cyclodextrins, and polyethylene glycols by MALDI-MS with high efficiency and reproducibility.^711^ Recently, Sekuła et al. prepared a durable surface of cationic AuNPs that covered both commercial and custom-made MALDI targets (AuNPET), demonstrating their applications in the detection of LMW organic compounds (nucleosides, saccharides, AAs, glycosides, nucleic bases, *etc.*)^198^ and imaging of renal cell carcinoma.^712^

###### 3.2.1.1.2 Silver Nanoparticles

AgNPs have attracted significant attention as a compelling alternative to AuNPs owing to their physical and chemical properties. Notably, AgNPs exhibit a high molar absorption coefficient (approximately 2×10^8^M^–1^ cm^–1^ at 337 nm) and show excellent MALDI matrix performance under nitrogen laser irradiation.^713^ Additionally, compared with AuNPs, AgNPs plasmon resonance is in the visible region and offers a broader spectrum of applications. The plasmon resonance band of AgNPs is narrower than that of AuNPs and typically occurs at approximately 400 nm.^714^ In the field of biomolecular analysis, AgNPs present additional advantages,^715^ such as: (1) relatively high salt tolerance, (2) the elimination of interference from matrix-related ions, (3) the generation of highly reproducible signals, and (4) antimicrobial and antifungal activity, which aids in the preservation of tissue for analysis. These characteristics position AgNPs as highly promising MALDI matrix candidates for diverse applications in the field of biological analysis.

In MALDI-MS analysis, Ag is a potent cationizing agent^715^ and is typically used to analyze various unsaturated compounds, such as cholesterol and FAs, as well as for moderately and low-polarity polymers. For example, AgNPs have been shown to effectively capture different compounds (*e.g.*, AAs, cholesterol, and FAs) on their surfaces,^714^ facilitating their desorption and gas-phase cationization, as illustrated in Figure 70c. Chiu et al. used AgNPs as a matrix and employed MALDI-MS to directly determine three types of estrogens-estrone (E1), estradiol (E2), and estriol (E3). The tested estrogens weakly adsorbed onto the surface of the AgNPs through van der Waals forces and were concentrated by centrifugation, providing a convenient and effective method for analysis.^713^ Additionally, a uniformly deposited layer of AgNPs has been used as a MALDI matrix for the visualization of lipids in rat heart tissue^716^ and rat kidney tissue,^717^ particularly for the detection and imaging of neutral lipids. Krupa and Nizioł employed single-isotope AgNPs (^109^AgNPs) as a matrix for the LDI-MS analysis of illicit drugs.^718^ Their approach, leveraging a high-resolution reflective TOF MALDI apparatus, achieved exceptional identification and quantification, particularly in the detection of substances such as fentanyl.

AgNPs can be modified with specific functional groups to facilitate the ionization of particular analytes. Hua et al. developed a citrate-capped AgNP method for MALDI-MS, enabling efficient desorption and ionization of specific peptides through strong laser absorption and high thermal conductivity of the nanostructure.^719^ In negative ion mode, Hayasaka et al. utilized alkylcarboxylate- and alkylamine- modified AgNPs as matrices for MALDI-MS analysis, leading to the successful identification of FAs, such as stearic, oleic, linoleic, arachidonic, and eicosapentaenoic acids, as well as palmitic acid in mouse liver tissue sections. They also demonstrated the specific detection and visualization of FAs in mouse retinal samples.^720^ As illustrated in Figure 70d, Kailasa and Wu used functionalized Ag_2_Se NPs as extraction probes for NP-based liquid-phase microextraction and analyzed hydrophobic peptides and proteins in biological samples (urine and plasma) and soybeans using MALDI-MS. Their findings revealed that surface-modified functional groups on Ag_2_Se NPs, including octadecanethiol (ODT) and 11-mercaptoundecanoic acid (MUA), played a crucial role in the efficient extraction of peptides and proteins, providing an effective method for biological sample analysis.^721^ Additionally, Guan et al. used polyvinylpyrrolidone (PVP)-capped AgNPs (PVP-AgNPs) as matrices for MALDI MSI, enabling the simultaneous detection of lipids and other small metabolites from mouse brains, including compounds with low ionization efficiency, such as FAs and sterols.^722^

Similar to AuNPs, AgNPs can also be used as substrates for MALDI-MS analysis. Gamez et al. prepared a sol-gel-derived AgNP-embedded thin film as a MALDI-MS biosensor. This method promoted the desorption and ionization of peptides, TAGs, and PLs, and suggested the possibility of preferential ionization of sterols, providing a simplified method for sterols in complex mixtures.^723^ In 2016, Schnapp et al. used nitric acid etching to produce highly porous silver foils as sample substrates for MALDI-MS and investigated the analysis of complex lipid mixtures in model systems of fruit fly (*Drosophila melanogaster*) and worker bee (*Apis mellifera*) cuticle extracts.^724^ Mass spectra obtained from the extracts spotted on Ag substrates demonstrated sensitive detection of long-chain saturated and unsaturated hydrocarbons, fatty acyl alcohols, wax esters, and TAGs, among other lipids. The Niziol team introduced a novel method for preparing modified MALDI targets by coating them with monoisotopic cationic ^109^AgNPs (^109^AgNPET). The method proved to be highly effective for the analysis of various LMW compounds, including alkaloids, saccharides, AAs, nucleosides, nucleic bases, and other organics.^725^ Moreover, this method has been applied to MALDI MS imaging of human pathological tissues,^726^ plant tissues,^727^ and fingerprints.^727^ Paweł et al. developed uniform AgNPs on a steel substrate using chemical vapor deposition (CVD) and evaluated their potential for analyzing LMW compounds such as lipids, carbohydrates, AAs, and carboxylic acids through MALDI-MS.^728^ Notably, they highlighted the capabilities of these AgNPs in the quantitative analysis of TAGs. Furthermore, the AgNP targets were effectively utilized to distinguish between cefotaxime-resistant and cefotaxime-sensitive strains of *Escherichia coli*, with lipidomic profiling proving successful in classifying these closely related bacterial strains.^729^

###### 3.2.1.1.3 Other Metal Nanoparticles

In addition to AuNPs and AgNPs, other metal NMs such as cobalt (Co), aluminum (Al), manganese (Mn), molybdenum (Mo), platinum (Pt), and palladium (Pd), serve as effective matrices for the detection and imaging of small molecules in MALDI-MS. In 1988, Tanaka et al. first demonstrated the use of Co powders mixed with glycerol as a binary matrix for protein analysis by MALDI-MS.^24^ Subsequently, Kinumi et al. studied the effects of metal NPs, including Al, Mn, Mo, silicon (Si), tin (Sn), tungsten (W), and zinc (Zn), when mixed with glycerol for MALDI-MS.^730^ As alternatives to traditional organic matrices, they mixed analytes with NPs of tens of micrometers in size and a liquid dispersant, and successfully employed them for the MALDI-MS detection of LMW analytes, polyethylene glycol 200 (PEG 200), and methyl stearate. Using a commercial MALDI-TOF MS instrument equipped with an internal 337 nm pulsed nitrogen laser, they observed that when employing inorganic matrices such as Mn, Mo, Si, Sn, W, or Zn, the analytes PEG 200 and methyl stearate were ionized as alkali metal adducts [M+Na]^+^ or [M+K]^+^. In the case of the Al matrix, PEG 200 was ionized as [M+K]^+^, while methyl stearate was ionized as [M+H]^+^ and [M+Al]^+^. This difference in adduct formation arises from the contrasting polarity and metal ion affinity of the two molecules.^730^ These findings highlight the potential of these nanoparticles to serve as effective MALDI matrices.

PtNPs are among the most extensively studied metals, and their potential applications in catalysis and fuel cells being well-established.^731^ Notably, PtNPs are highly stable and do not oxidize under ambient atmospheric conditions. PtNPs can be prepared by the reduction of metal in aqueous solution, forming a pure sol that appears black, allowing them to effectively absorb UV light of any wavelength for desorption and ionization of biomolecules by MALDI-MS.^732^ Shrivas et al. used PtNPs for analyzing AAs, peptides, proteins, and microwave-assisted digested proteins in MALDI-MS.^732^ Furthermore, Nitta et al. prepared fluorocarbon-based hydrophobic perfluorodecyltrichlorosilane (FDTS)-Pt nanoflower (FDTS-PtNf) chips, which exhibited improved sensitivity and reproducibility for analyzing peptides, AAs, FAs, and nucleotides using negative ion MALDI-TOF-MS.^733^ Shen et al. introduced a Pt nanomaterial pre-coated matrix by bulk deposition of PtNPs onto glass slides through ion sputtering, which facilitated MALDI imaging analysis.^734^ This matrix demonstrated a high melting temperature and stability, while simplifying experimental procedures and improving reproducibility, thus allowing effective visualization of oligosaccharides and lipid components in plant tissues. Additionally, Silina et al. investigated a simple method for synthesizing PdNPs with easily adjustable morphology using an electrochemical deposition method and applied them to the MALDI-MS of small biomolecules.^735^ PdNP films exhibited excellent performance in evaluating FAs, TAGs, carbohydrates, and antibiotics.

Ferric NPs (FeNPs) with designed surface roughness (∼5 nm diameter) enable *in situ* enrichment and separation of small molecule metabolites, allowing simple sample preparation, rapid detection, and minimal sample consumption in MALDI-MS analysis. Liu et al. utilized this Fe particle-assisted platform to investigate metabolic fingerprints in patients with type 2 diabetes and employed MALDI-FTICR-MS/MS to identify specific metabolites.^736^ Additionally, nano-zero-valent iron (nZVI), a metal nanomaterial known for its unique physical and chemical properties, has been extensively applied in environmental chemistry, particularly because of its strong reducing and complexing capabilities for positively charged heavy metal ions, as well as its high adsorption capacity for organic compounds.^737^ Leveraging these advantageous characteristics, Wan et al. developed a nZVI-assisted LDI-MS platform that efficiently captures metabolites from complex biological samples, allowing the acquisition of serum metabolic fingerprints in negative ion mode and thereby providing a novel strategy for the molecular diagnosis of pulmonary nodules.^737^

##### 3.2.1.2 Metal Oxide Nanomaterials

Metal-based NMs have the advantages of low detection limits, high D/I efficiency, and relatively simple sample preparation. However, metal NPs and their raw materials are expensive. In contrast, metal oxide NPs are more stable, and can be used as multifunctional probes for the separation, enrichment, and ionization of target analytes. They also offer excellent performance and lower cost, making them a potential alternative matrix for MALDI.^691^ Among metal oxides, transition metal oxide NPs, such as titanium dioxide (TiO_2_), zinc oxide (ZnO), iron­(III) oxide (Fe_2_O_3_), magnetic iron oxide (Fe_3_O_4_), nickel oxide (NiO), and tricobalt tetroxide (Co_3_O_4_), possess unique properties such as large surface area to volume ratios, low porosity, high dispersion rates, high photoabsorption, and low heat capacities,^740^ making them particularly promising for small molecule detection in MALDI-MS.

TiO_2_ nanostructures, which are wide bandgap semiconductor materials, are widely accepted as inorganic MALDI matrices because of their unique physical and electronic properties that improve the ionization efficiency for analyte resolution.^741^ A variety of TiO_2_ nanostructures consisting of NPs, thin films, nanotubes, and nanowires have been reported for MALDI-MS analysis. For example, Kinumi et al. demonstrated that suspending TiO_2_ powder in liquid paraffin resulted in MALDI-TOF MS of PEG 200 and methyl stearate characterized by the low background noise and high intensity.^730^ Watanabe et al. investigated the effects of urea surface modification and photocatalytic cleaning on MALDI-MS with amorphous TiO_2_ NPs, which reduced background noise and improved the sensitivity of the peptides.^742^ Shrivas et al. reported their use of TiO_2_ NPs as a matrix for MALDI-MS analysis of endogenous LMW metabolites in mouse brains without the need for washing and separation steps.^743^ Moreover, Peng et al. employed TiO_2_ NPs as the matrix for MALDI MS to analyze lipids, including small molecular lipids, lipid standards, and complex lipid mixtures.^744^ Notably, TiO_2_ NMs have been extensively modified. For instance, holmium (Ho)-modified TiO_2_ nanocomposites (Ho-TiO_2_) were utilized to detect bisphenol S and indigo pollutants at pg/ml concentrations by MALDI-MS.^745^ DA-functionalized TiO_2_ NPs (TiO_2_-DA) have been successfully employed in MALDI-MS to visualize small metabolites and lipids in mouse brain tissue^746^ and serum lipidomic analysis in breast cancer patients.^747^ Chen and colleague have developed TiO_2_ sol-gel-deposited thin film as MALDI matrices to facilitate D/I of analytes.^748^ This modified glass substrate effectively selects *r*-cyclodextrin (*r*-CD)­from a sample solution containing equivalent amounts of *r*-CD, α-CD, and γ-CD, and allows for direct detection of *r*-CD by MALDI-MS without the need for an additional matrix. Wang et al. harnessed the photocatalytic properties of TiO_2_ to construct an array structure on octadecyltrichlorosilane (OTS)-modified TiO_2_ nanofilms called photocatalytically patterned titanium dioxide array (PPTA).^749^ The PPTA effectively enables selective on-plate enrichment of phosphopeptides, demonstrating significant performance in MALDI-MS applications across complex samples, including phosphorylated protein digests, human serum, and defatted milk. In addition, in MALDI-MS studies of phosphoproteomics, TiO_2_ coated MALDI plate or TiO_2_ films have been used as affinity targets for the selective separation and enrichment of phosphopeptides.^750–752^ Piret et al. successfully used TiO_2_ nanotube layers prepared by electrochemical anodization of titanium foil as a novel inorganic matrix for MALDI-MS analysis of peptides and small organic molecules.^753^ Additionally, Dutkiewicz et al. synthesized functionalized TiO_2_ nanowires and applied them to MALDI-MSI of vinca alkaloids in the petals of *Catharanthus roseus* with spatial resolutions ranging from 250-500 μm.^738^ A schematic diagram of their process is depicted in Figure 70e.

ZnO is among the most widely studied metal oxide NMs, and it is used in applications such as solar cells, sensors, paints, cosmetics, and light-emitting diodes.^754,755^ ZnO is a semiconductor with a wide bandgap (3.37 eV), making it suitable as an inorganic MALDI matrix when used with a N_2_ laser (337 nm).^756^ To date, studies have reported the use of ZnO NPs mixed with glycerol for the MALDI-MS analysis.^730^ Additionally, Watanabe and colleagues utilized ZnO NPs with anisotropic shapes without a liquid matrix for MALDI-MS analysis of LMW compounds such as verapamil hydrochloride, testosterone, and polypropylene glycol.^756^ Gedda and Wu demonstrated the use of a surface-modified ZnO nanorod (NR) array chip for on-plate detection, preconcentration, separation, and subsequent MALDI-MS analysis of bacteria in nanoliter liquid droplets.^757^ A surface-modified ZnO NR array can be easily fabricated on Zn foil using a chemical method. Additionally, the method showed high reproducibility for analyzing bacteria in nanoliter droplets and can be utilized as an effective affinity probe for detecting and separating bacteria from seawater and urine.

With the addition of the recognition unit, magnetic NPs can serve as extraction probes for analytes and as matrices for MALDI-MS, allowing direct detection on the MALDI-MS target plate after rapid separation. Chen and Chen used silane-immobilized Fe_3_O_4_ NPs for MALDI-MS analysis, which enabled the detection of small proteins and peptides with a maximum detection mass range of approximately 16 kDa and a peptide detection limit of approximately 20 fmol.^758^ They also demonstrated the function of silane-immobilized Fe_3_O_4_ magnetic particles as affinity probes, selectively capturing substances with opposite charges. This dual-function particle enabled direct analysis of tryptic digests of cytochrome C at concentrations as low as 10 nM, providing a new approach to affinity MALDI-MS analysis of inorganic MALDI matrices and concentration probes.^758^

In addition to metal oxide NPs, many other metal oxide materials have been developed as inorganic MALDI matrices. Olaitan et al. systematically investigated the application of NiO, Fe_3_O_4_, TiO_2_, and Co_3_O_4_ NPs for the MALDI-MS detection of asphaltenes.^740^ Kuwata and colleagues developed a novel MALDI device based on a porous alumina membrane, called desorption ionization through hole alumina membrane (DIUTHAME) and demonstrated the feasibility of MALDI-MS measurements on liquid samples.^759^ The unique 2D ordered structure of this device, consisting of closely aligned straight-through-holes of submicrometers in diameter, provides rich signal peaks in the PL region without chemical background interference, producing high-quality images that accurately reflect the anatomic structure of brain tissue.^759^ Moreover, Qiu et al. synthesized surface hydroxyl-rich Co_3_O_4_ nanocrystals as a novel matrix for MALDI-TOF MS detection of small molecules. Co_3_O_4_ nanocrystals exhibit excellent characteristics such as low background interference, good reproducibility, and high signal intensity, making them suitable for the analysis of AAs, pesticide residues (*e.g.*, carbofuran and pyridine), and harmful additives (*e.g.*, bisphenol A and melamine).^692^ Qiu et al. subsequently designed and synthesized Co_3_O_4_/cobalt hydroxide (Co­(OH)_2_) heterojunctions by a simple one-pot method (Figure 70f), significantly increasing the target peak intensity and sensitivity for amino acid detection in MALDI-TOF MS.^739^ This composite demonstrates excellent performance for the rapid detection of small molecular environmental pollutants, attributed primarily to the formation of a heterogeneous structure that reduces the surface potential and facilitates the effective separation of photogenerated electron–hole pairs. Sun et al. proposed a simple method for enrichment and identification of phosphopeptides using cerium oxide (CeO_2_) NPs as an inorganic matrix for MALDI-TOF-MS.^760^ After pretreatment of the tryptic digests of phosphoproteins with CeO_2_, phosphopeptides are enriched. By applying the separated CeO_2_ on a target plate and analysis using MALDI-TOF MS, the peaks of phosphopeptides and their corresponding series of dephosphorylated peptides can be observed in the mass spectra. Considering that W has the highest melting point of any element and is therefore resistant to melting, and has a relatively low heat capacity compared with that of pSi, Yang et al. selected W as a potential substrate for MALDI and demonstrated the excellent performance of tungsten trioxide (WO_3_) as an inorganic MALDI-MS substrate, successfully detecting peptides and drugs in more than 20 different compounds.^761^ WO_3_NPs have also been shown to be effective when combined with glycerol for MALDI-MS analysis of PEG 200 and methyl stearate.^730^

##### 3.2.1.3 Other Metallic Nanomaterials

In recent years, researchers have successfully synthesized a variety of multicomponent mesoporous materials with ordered, large nanopores and stable mesoporous structures on the basis of the concept of “acid–base pairs”.^762^ These materials include metal phosphates, metal oxides, and mixed metal oxides, which have high thermal stability, tunable compositions, various morphologies, and abundant useful properties, and exhibit great potential for applications in catalysis, optics, and electronic devices.^763^ Shan and colleagues proposed a novel multicomponent mesoporous material, tungsten-titanium oxide (WTiO), with well-defined 2D nanostructures. Owing to its high UV absorption and large surface area, it has shown strong potential as a new matrix for MALDI-TOF-MS analysis.^763^ Furthermore, Kailasa and Wu reported a novel dual-functional NP system, namely 2-hydroxy octadecanoic acid (HOA)-modified barium titanate NPs (BaTiO_3_ NPs). These NPs not only serve as matrices for PL detection but also act as hydrophobic affinity probes for liquid–liquid microextraction in *Escherichia coli*, which facilitates identification using MALDI-MS.^764^

Hybrid NMs based on metal materials offer several advantages, including high dispersibility, surface roughness, enhanced UV absorption, and thermal conductivity.^691^ The hybrid nanoflowers of AuNPs on MnO (Au@MnO) have been demonstrated to serve as an effective matrix for the MALDI-MS determination of ATP.^765^ Au@MnO composed of two different nanocomponents has significant advantages over individual Au and MnO NPs because of the synergistic ionization effect of the two structural domains within a single nanoflower particle. Lin et al. synthesized a novel mesoporous NiO@ZnO nanofiber membrane using a single-nozzle electrospinning technique.^766^ Compared with individual NiO and ZnO NMs, the NiO@ZnO nanofiber membrane has been proven to be an effective material with numerous NiO@ZnO NPs with mesoporous and hollow structures that enhance the ionization efficiency of analytes. As depicted in the workflow shown in Figure 71a, this nanofiber membrane demonstrated potential applications as a matrix in urine metabolism analysis, revealing differences in smokers’ metabolism. Additionally, Li et al. synthesized a novel nitrogen-enriched Ag@Ti_3_C_2_ (Ag@N-Ti_3_C_2_) using an eco-friendly, UV-assisted strategy, forming a highly uniform film on the target plate (Figure 71b).^767^ Compared with the Ti_3_C_2_ material, the Ag@N-Ti_3_C_2_ composite has enhanced surface area, electrical conductivity, and thermal conductivity. This makes it an ideal candidate for high-throughput pesticide analysis, offering ultrahigh sensitivity, enhanced reproducibility, and good salt tolerance.

**Figure 71:**
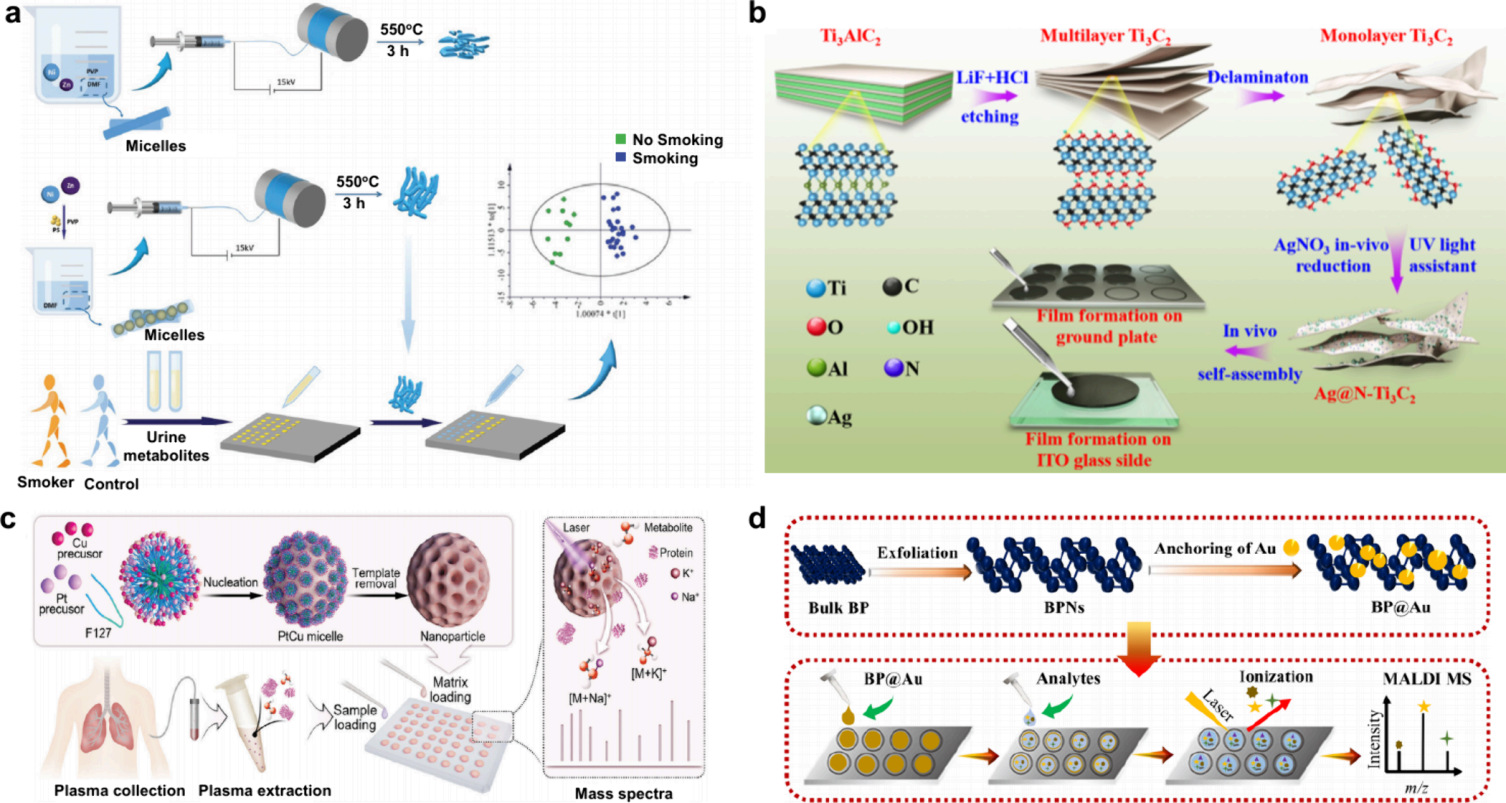
Other metallic nanomaterials as matrices for MALDI-MS. (a) Schematic illustration of the synthesis and application of the mesoporous NiO@ZnO nanofiber membrane. Adapted and reproduced with permission from ref 766. Copyright 2022 Royal Society of Chemistry. (b) Schematic of the synthesis of Ag@N-Ti_3_C_2_ and *in vivo* self-assembly of the Ag@N-Ti_3_C_2_ film. Adapted and reproduced with permission from ref 767. Copyright 2023 American Chemical Society. (c) Scheme of porous PtCu alloys decoding plasma metabolic fingerprints for the recognition of severe community-acquired pneumonia. Adapted and reproduced with permission from ref 771. Copyright 2025 Wiley-VCH. (d) Diagram illustrating the synthesis of plasmonic 2D BP@Au nanocomposites and their subsequent application as a MALDI nanomatrix for the detection of anticancer drugs. Adapted and reproduced with permission from ref 770. Copyright 2025 The Authors under exclusive licence to Springer-Verlag.

Plasmonic materials, particularly noble metals, exhibit unique surface resonances under laser irradiation, making them ideal matrix materials because of their high thermal carrier yield and porous structure, which effectively enhance the desorption and ionization capabilities of analytes.^768^ Zhou et al. synthesized a novel trimetallic alloy (PdPtAu) as a matrix, significantly increasing the sensitivity and selectivity for the detection of biological thiols primarily by enhancing the binding affinity between thiol groups and noble metals.^768^ Its mesoporous structure facilitates direct analysis of complex biological samples without additional processing, and the incorporation of internal standards in quantitative MS enables precise quantification of methionine in serum samples, as well as real-time monitoring of the glutathione oxidation–reduction reaction. Additionally, Qian et al.developed a mesoporous PdPt alloy-assisted LDI-MS technique for plasma metabolite analysis, enabling accurate diagnosis of chronic obstructive pulmonary disease (COPD) and its exacerbations.^769^ The outstanding performance of this alloy is due to its enhanced electric field and photothermal conversion efficiency, which facilitate rapid analysis without complex pretreatment. The area under the curve (AUC) values reached 0.904 and 0.955 in the different cohorts, effectively distinguishing stable COPD from acute exacerbations. Concurrently, they teamy designed a porous PtCu alloy-assisted LDI-MS technique for extracting plasma metabolic fingerprints (Figure 71c), which integrates machine learning to diagnose severe community-acquired pneumonia.^770^ This model achieved an AUC of 0.832, significantly improving diagnostic efficiency. Furthermore, Mandal et al. synthesized a novel plasmonic 2D black phosphorus nanosheet–gold (BP@Au) nanocomposite through a simple one-pot ultrasonic-assisted procedure, as shown in Figure 71d. This plasmonic BP@Au nanocomposite demonstrated low detection limits and high specificity for detecting anticancer drugs such as irinotecan (CPT-11) along with its metabolically active derivative, enabling quantitative analysis in mouse serum.^771^

Recent studies utilized metal-polyphenol networks (MPNs) as coordination and redox agents to synthesize novel noble metal-based nanocomposites. MPN consists of tannic acid (TA) complexed with various transition metal ions, including Fe^3+^, Co^2+^, Ni^2+^, Cu^2+^, and Zn^2+^, which coat the surfaces of AuNPs to form a metal-TA network. Sang et al. synthesized a novel noble metal-based nanocomposite, Au-MPN@Ag, for MALDI-MS detection of small metabolites. Owing to its unique nanostructure and exceptional physicochemical properties, the Au-MPN@Ag nanocomposite demonstrated significantly enhanced ionization efficiency and detection sensitivity. Furthermore, this platform enables the direct detection of metabolites in complex biological fluids, such as urine and cerebrospinal fluid, without the need for any sample pretreatment.^772^ Additionally, Hu et al. functionalized AuNPs with MPN (M-TA@AuNPs) as adsorbents and matrices, and reported varying enrichment effects for different AAs, thus greatly enhancing the ability of MALDI-MS to detect small molecular metabolites. Notably, Cu-TA@AuNPs showed the highest affinity for histidine (His) and were successfully applied for detecting His in human urine.^773^

##### 3.2.1.4 Summary

In summary, metal NMs have been rapidly developed and widely applied in MALDI-MS in recent years because of their high absorption coefficient for UV–visible light, good chemical stability, ease of functionalization, and flexible sample preparation.^774^ In addition, metal NMs exhibit selectivity toward certain analytes, such as the strong interaction between Au/Ag and S, which can be utilized for the detection of sulfur compounds.^706,714^ In recent years, metal NMs have been extensively used as matrices or affinity probes for the analysis of biomolecules in MALDI-MS. However, individual metal NPs have limitations, including aggregation and interference from background ions (*i.e.*, metal cluster species), which lead to reduced sensitivity and selectivity.^691^ Functionalized metal NPs can overcome these issues, as their high surface area-to-volume ratio and excellent chemical and physical properties make them ideal materials for the efficient extraction of biomolecules from low-concentration samples.^775,776^ Additionally, metal oxide NPs have been widely used as cost-effective matrices in MALDI-MS because of their high absorbance, semiconductor properties, and simple synthesis procedures.^777^ Compared with metal-based NMs, metal oxide NPs possess excellent chemical stability and are less reactive in air. Their high melting and boiling points make them difficult to ionize, significantly reducing background noise.^177^ Metal oxide-based MALDI matrices provide high sensitivity with low background signal interference, particularly in the design of composite materials. Metal oxide NPs serve as multifunctional probes and are extensively used in the separation, enrichment, and ionization of target analytes. They offer the advantage of relatively simple sample preparation and are commonly used as inorganic matrices in MALDI-MS.^774^

The core metals and functional groups of metal-based NMs (including metal NPs, metal oxide NPs, and their hybrid materials) can be modified to obtain specific chemical properties to increase analyte ionization. Customized functional materials can be obtained by adjusting the inherent functional groups on the polymer monomer backbone. Factors such as the size selection, surface modification, and matrix application of metal-based NMs significantly impact their performance and the ionization efficiency of analytes. Therefore, the preparation and application methods need to be comprehensively considered when different analytes are being studied. Additionally, the preparation and functionalization of metal-based nanomaterial composite materials is a complex process that requires meticulous preoptimization in matrix design. As a result, the straightforward synthesis of metal oxide-based complexes has become the preferred method for matrix preparation, although further exploration is still needed. In conclusion, metal-based NMs have a wide range of prospects for application in MALDI-MS. In practical applications, the preparation and application methods and the exploration of new synthetic methods and functionalization pathways must be fully considered.

#### 3.2.2 Carbon-Based Nanostructures

Carbon NMs, with properties such as high UV absorption, high theoretical surface area, and high electron conductivity, demonstrate significant capabilities in the MALDI-MS analysis of small molecules.^177^ Carbon NMs are composed of a group of molecules with different forms and physical and chemical properties, or various allotropes, such as fullerenes, carbon nanotubes (CNTs), graphene (G), graphite, carbon nanofibers (CNFs), and nanodiamonds (NDs).^70^ To date, carbon NMs have achieved varying degrees of success in MALDI-MS applications.

##### 3.2.2.1 Fullerenes

Fullerenes are hollow spheres formed by sp^2^-hybridized carbon atoms and possess a highly conjugated system of electron-deficient alkenes.^70^ Owing to their strong light absorption and ability to interact with various chemical groups, fullerenes have a wide range of analytical applications. Among carbon particles, fullerenes are preferred as MALDI matrices because of their well-defined structure, strong UV absorption, and high purity.

The Willett group reported the first use of C60 fullerene as a MALDI matrix in 1994.^778,779^ These studies demonstrated the successful detection of proteins by depositing analytes on a thin film of C60 fullerene (∼10 nm thick) and forming analyte ions through nitrogen laser irradiation, with successful identification of proteins such as cytochrome *c* (12 kDa) and BSA (66 kDa). Furthermore, Montsko and coworkers developed a sensitive method for the analysis of natural steroids using MALDI-TOF-MS. They used C70 fullerene as a matrix for nonderivatized ionization and successfully applied it to the detection of several steroid hormones (estrone, β-estradiol, estriol, progesterone, and testosterone), synthetic steroids, and urine samples from pregnant women.^69^

Owing to the uneven distribution of polar analytes in nonpolar fullerene matrices, fullerene matrices provide lower sensitivity than that obtained with small organic matrices. Therefore, in addition to improving sample preparation schemes to maximize ion yield in MALDI processes, efforts should be made to enhance the surface polarity of fullerenes. The development of fullerene derivatives with multiple functional groups has significantly broadened the possibilities in fullerene science and technology, as evidenced by their successful application as MALDI matrices for protein detection in recent studies. Shiea et al. synthesized a starlike water-soluble fullerene derivative, hexa­(sulfonbutyl)­fullerene (C60­[(CH_2_)_4_SO_3_^–^]_6_; HSBF), which consists of a C60 cage covalently bonded with six negatively charged sulfonate arms, and exhibits amphiphilic properties similar to those of molecular micelles, as shown in Figure 72a.^780^ HSBF has been used to selectively precipitate specific surfactants and biomolecules, including quaternary amines, AAs, peptides, and proteins, because of its net positive charge and interactions with sulfonyl and alkyl groups. It has also been utilized as a MALDI matrix for the direct detection of analytes precipitated by ionic sulfonate arms.^780^ Furthermore, Qin et al. used bromoacetyl functionalized C60 (Br-C60) as a derivatization reagent to label thiols (Figure 72b), thus avoiding the signal interference caused by conventional MALDI matrices and enabling the high-throughput detection of thiols.^781^ They successfully identified four LMW thiols including glutathione, cysteine, homocysteine and cysteinylglycine, in human serum using Br-C60 as a MALDI matrix. Liu et al. used the fullerene alcohol C60­(OH)_24–26_ as a MALDI matrix to prevent heterogeneous crystallization during sample preparation^782^ and successfully detected small molecules (including AAs, peptides, nucleosides, metal ion complexes, and nonpolar compounds) and quantitatively analyzed sodium saccharin in nuts and beverages using MALDI-TOF-MS. In addition, the functionalized fullerenes C60­((CH_2_)_2_COOH)_*n*_ or C_60_(C_11_H_23_)_*n*_ (*n* is nominally 6) are used in MALDI-MS for the detection of peptides and PLs.^783^

**Figure 72:**
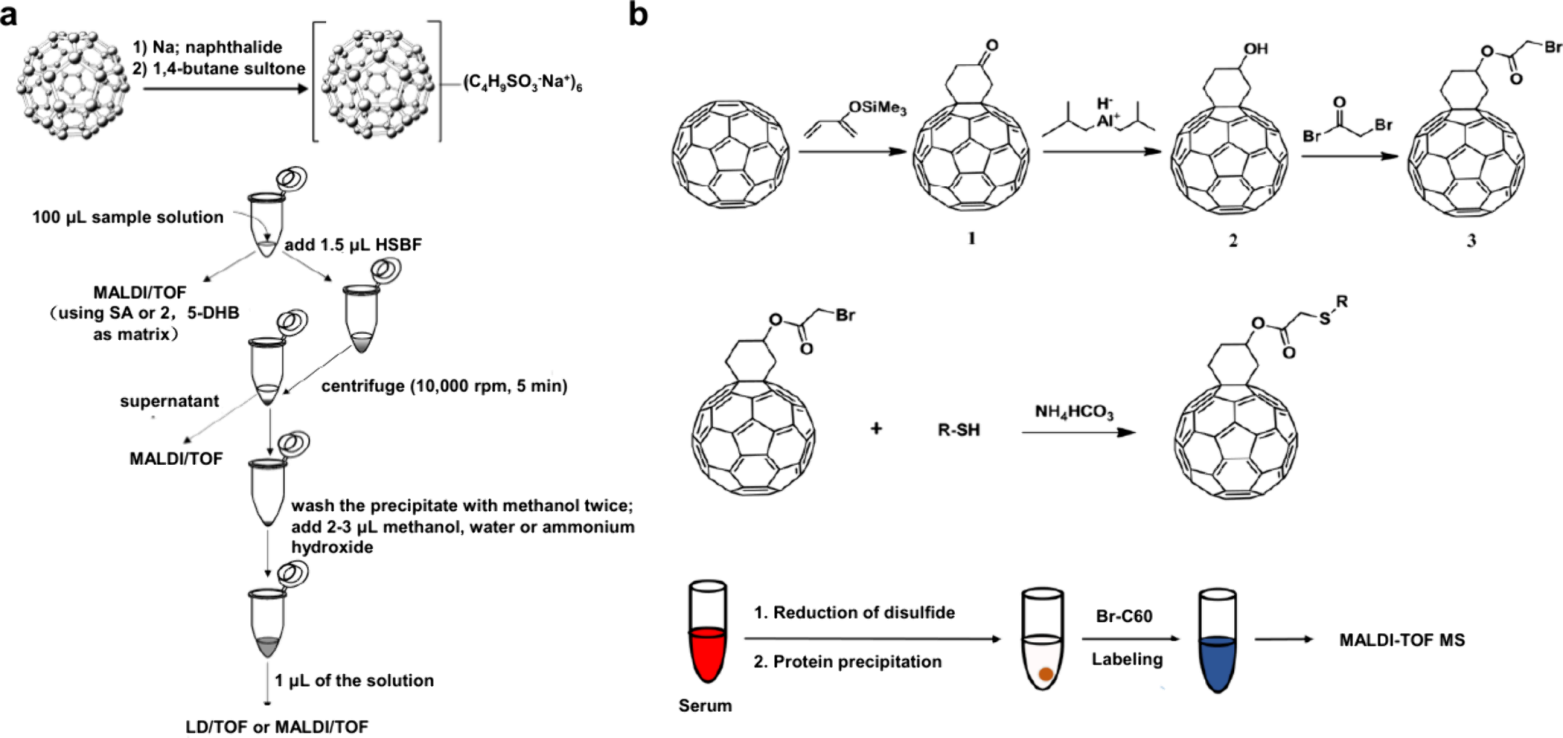
Fullerenes as matrices for MALDI-MS. (a) Synthetic protocol for the sodium salt of the starlike water-soluble fullerene derivative hexa­(sulfonbutyl)­fullerene (C60­[(CH_2_)_4_SO_3_^–^Na^+^]_6_; HSBF) and general analytical procedures for selectively precipitating and detecting charged analytes in aqueous sample solution. Adapted and reproduced with permission from ref 780. Copyright 2003 American Chemical Society. (b) Chemical labeling scheme of bromoacetyl functionalized C60 (Br-C60) and thiols and schematic illustration of the determination of thiols by the Br-C60 labeling-MALDI MS strategy. Adapted and reproduced with permission from ref 781. Copyright 2020 Elsevier BV.

##### 3.2.2.2 Carbon Nanotubes

Since Iijima's discovery in 1991,^784^ single- or multi- walled CNTs have been widely used in various fields because of their unique size, large surface area, hollow and nanoscale layered structure, and excellent physical and chemical properties.^785,786^ CNTs have also been shown to be useful as matrices for MALDI-MS, where they serve as laser receptors and energy transfer agents, enabling the D/I of analytes with minimal or no matrix ion interference.^783^

In 2003, Xu et al. first demonstrated the use of CNTs obtained from coal by arc discharge as MALDI matrices, showing that CNTs can serve as alternative carbon materials for small molecule MALDI analysis.^67^ Although this method has advantages such as low detection limits and low matrix background, the original CNTs have poor solubility, making them difficult to disperse in aqueous systems and deposit onto sample targets to form a uniform layer.^787^ Ren et al. introduced a polyurethane adhesive, NIPPOLAN-DC-205, to fix CNTs onto the target, preventing the CNTs from flying off the target under laser pulse action, which could contaminate the mass spectrometer and cause time-limited signals and time-consuming searches for “hot spots”.^788^ By using immobilized CNT matrices, small neutral carbohydrates that are otherwise difficult to ionize can be cationized efficiently using MALDI-TOF-MS and have been successfully used for the detection of peptides and urine glucose.

Recently, functionalized CNTs (f-CNTs) have attracted increased attention because of their diverse applications in various fields. Oxidized CNTs have been developed as matrices for the analysis of LMW compounds in biological samples by MALDI-TOF MS. After the oxidation process, the CNTs can be transformed into short nanotubes with carboxyl groups (-COOH, -COONa, or -COOK),^789,790^ becoming water-soluble and pure. This method simplifies the sample handling procedure, provides good reproducibility of spots within a sample and between samples, and offers the possibility of quantitative analysis. For example, Hu et al. used oxidized CNTs as matrices in MALDI-MS to detect highly polar LWM compounds in environmental samples, *e.g.*, to identify arsenic speciation in traditional Chinese medicines and to quantify diphenylolpropane in water samples.^791^ Pan et al. also utilized oxidized CNTs as MALDI matrices in MALDI-MS to detect synthetic hydroxypropyl β-cyclodextrin, and simultaneously determine the content of jatrorrhizine and palmatine in *Coptis chinensis Franch* extract.^792^ Additionally, Wang et al. successfully prepared CNTs with short open-ended structures on porous anodic alumina (PAA) templates and found that these oxidized CNTs can bind to alkaline cations and efficiently absorb laser radiation energy, enabling quantitative analysis of neutral carbohydrates and AAs in urine and corn roots.^793^

The surface of CNTs can be covalently or noncovalently modified to prepare dispersible CNTs that can be “dissolved” in the analyte solution and deposited onto sample targets to form a uniform layer.^794^ For instance, Jiang and Gao proposed a method of using cationic polyethyleneamine or anionic citric acid as the dispersant, and the surface properties of CNTs could be changed to yield a basic or acidic surface.^795^ Chen et al. further utilized citric acid-treated CNTs as affinity probes to selectively enrich target substances with specific opposite charges from aqueous solutions (which are suitable for small proteins, peptides, and protein digestion products). This approach simplified the sample preparation process and facilitated direct MALDI-MS detection of the target analytes without the need for additional organic MALDI matrices.^787^ Meng et al. developed a novel method for the hydrothermal synthesis of a multiwalled CNTs (MWCNTs) and polyaniline (PANI) composite (MWCNTs@PANI) as shown in Figure 73a, where the water-soluble polyaniline coating on the surface of the CNTs significantly improved their dispersibility in water.^796^ Shi et al. demonstrated that modifying MWCNTs with polydopamine (PDA) (MWCNTs@PDA), as shown in Figure 73b, made them dispersible and allowed them to bind well to various water-soluble small molecule compounds, thereby enhancing their performance in MALDI-TOF-MS analysis.^794^ Transition metal carbides (MXenes), characterized by their 2D structure and excellent electrical conductivity, can synergistically enhance laser photon absorption,^797^ thereby accelerating the ionization and desorption of analytes and improving the sensitivity of MALDI-MS. Chen et al. prepared a 2D MXene material through sodium hydroxide (NaOH) etching, which was subsequently combined with MWCNTs (MXene/MWCNTs) via hydrothermal synthesis, resulting in a matrix material suitable for metabolomics applications. They used the MXene/MWCNT-assisted LDI-MS method to analyze the metabolomics of psoriasis, identify metabolites associated with the condition and propose potential pathological mechanisms.^798^

**Figure 73:**
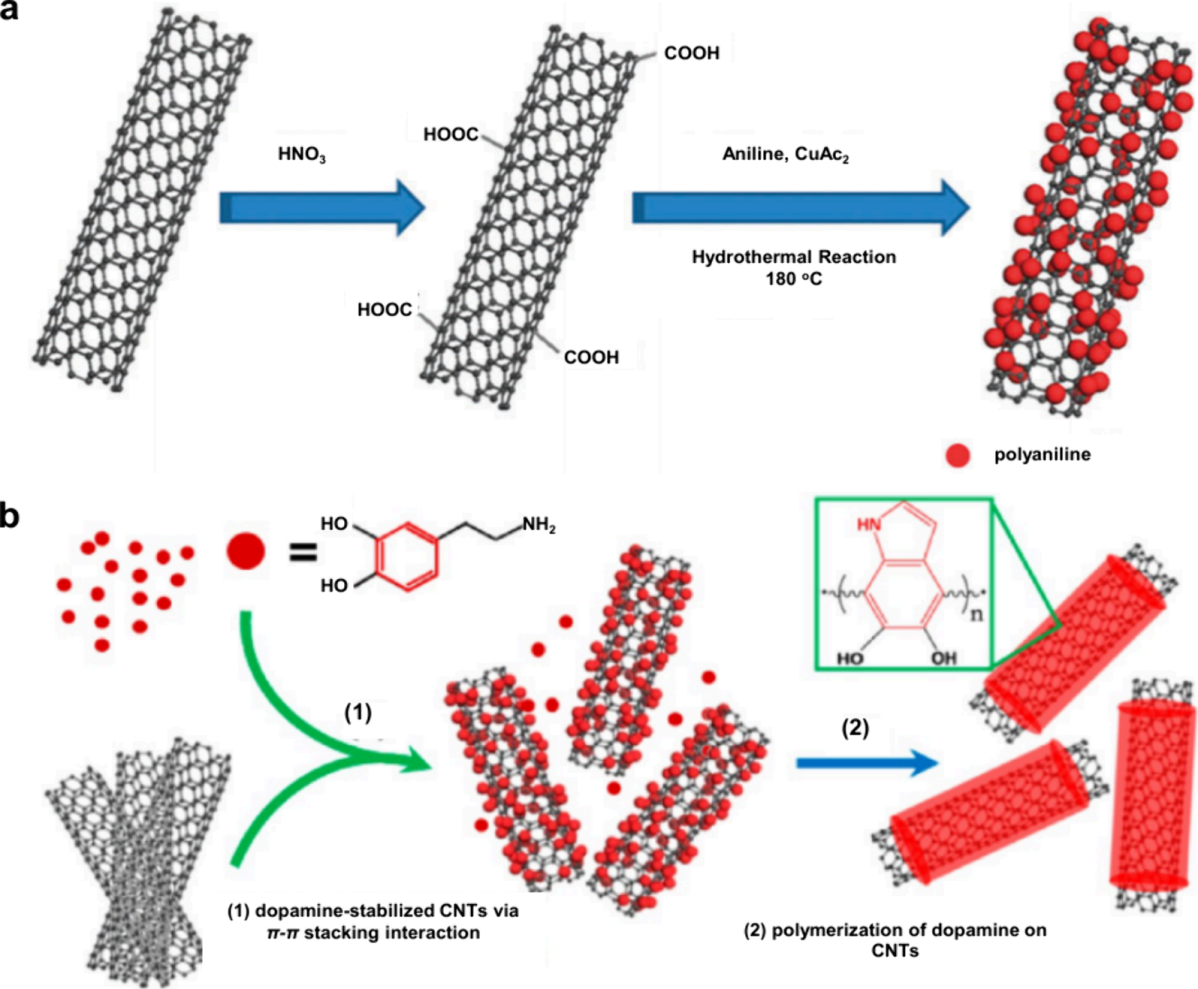
Carbon nanotubes as matrices for MALDI-MS. (a) The synthetic procedure of water-soluble multi-wall carbon nanotubes and polyaniline (MWCNTs@PANI) composites. Adapted and reproduced with permission from ref 796. Copyright 2011 Royal Society of Chemistry. (b) Synthesis procedure for water-dispersible multiwalled carbon nanotubes@polydopamine (MWCNTs@PDA). Adapted and reproduced with permission from ref 794. Copyright 2013 American Chemical Society.

##### 3.2.2.3 Graphene and Graphene Oxide

In recent years, the remarkable physical and chemical properties of G and graphene oxide (GO) have garnered significant attention, leading to extensive research in diverse applications. The large delocalized π-electron system of G and GO has a strong affinity for carbon-based ring structures, making them suitable for use as MALDI matrices,^799^ and they have been effectively employed in this capacity.

Since Novoselov et al.'s pioneering work in 2004, where single-layer G was successfully produced through mechanical exfoliation, G has attracted significant interest because of its remarkable performance.^800^ This 2D carbon material with a single atom thickness and an sp^2^-bonded structure, has exceptional properties, such as a large surface area, high thermal conductivity, excellent mechanical properties, and outstanding electronic transport properties, which make it an ideal choice for energy absorption, storage, and transfer.^800,801^ In 2010, Dong et al. first reported the application of G in the detection of AAs and nucleosides using MALDI-MS,^68^ ushering in a new era of MALDI analysis for small molecules. Lu et al. reported that compared with positive ion mode, the use of G as a matrix for analyzing LMW compounds, including peptides and FAs, resulted in improved sensitivity and reproducibility in negative ion mode.^802^ A study by Zhang et al. revealed that G-based MALDI-MS can effectively detect some environmental pollutants, such as PAHs and estrogen.^803^ Similarly, Chang et al. validated the versatility and effectiveness of G as a MALDI-MS matrix, and simple analyses of more than 40 compounds with different structures confirmed its performance. In addition, the method successfully differentiated three Chinese teas by detecting endogenous caffeine and theanine, demonstrating the potential of this method for the analysis of real samples.^804^ Graphene is considered a surface that can facilitate noncovalent ionization without disrupting weakly bound complexes. Abdelhamid and Wu investigated the noncovalent binding interactions between a steroidal anti-inflammatory drug (*i.e.*, flufenamic acid (FF)) and metals (Fe­(II), Fe­(III), and Cu­(II)) using graphene as a matrix at physiological pH 7.4, confirming the affinity of the drug and its complexes for biological metals.^805^

GO is the most common G derivative, produced by oxidizing G, and it contains hydroxyl and epoxide functional groups on the basal planes, with carbonyl and carboxyl groups located at the edges of the flakes.^806^ All of these properties make GO sheets hydrophilic and thus easy to swell and disperse in water. The water dispersibility and swelling properties of GO simplify sample preparation^807^ and increase reproducibility, making GO a promising alternative matrix for small molecule MALDI-MS analysis. Lee et al. differentiated disaccharide isomers by comparing the dissociation patterns obtained from MALDI-MS using GO as a matrix, while assessing the feasibility of quantitatively analyzing disaccharide isomers using mixtures.^808^ Zhou et al. used GO as a MALDI matrix to image small molecules in tissues in negative ion mode and successfully detected 212 molecules from mouse brain tissue slices without interference from matrix ions/clusters.^809^ To date, G and GO have also been successfully applied as matrices in MALDI-MS analysis of AAs, polyamines, peptides, steroids, FAs, nucleotides, and polysaccharides.^809–811^

The limited applications of G and GO owing to self-aggregation^812^ and the narrow range of target molecular interactions^813^ can be addressed by implementing functionalization strategies.

In addition to oxidation, the fluorination of G provides an opportunity to form hydrogen bonds with target compounds, thereby facilitating the extraction and ionization of the positive and negative modes.^814^ Min et al. successfully synthesized gas-phase nitrogen-doped graphene (gNG) and applied it as a MALDI matrix for small molecule analysis, demonstrating superior performance in obtaining matrix-interference-free mass spectra in negative ion mode.^253^ gNG exhibits good ionization efficiency in negative ion mode because of the π-conjugated system used for laser energy absorption and the nitrogen species doped as deprotonation sites for negative ion mode ionization. Furthermore, Zhao et al. synthesized O-P and N-doped C/G composites (O-P,N-C/G) using pyrolysis of phytic acid, polyaniline, and electrochemically exfoliated graphene.^815^ A G-based matrix with P-O surface functional groups coated on the surface of the G substrate achieved P and N codoping and enabled dual ion mode detection of small molecules by MALDI-MS. Lu et al. prepared 3D mesoporous G (3D-MG) using a microwave-assisted method, which served as an adsorbent and matrix for MALDI-TOF-MS to enable the detection of polyphenols with low background interference and high sensitivity.^816^ The layered mesoporous structure of 3D-MG effectively enriched target molecules, increased detection sensitivity, and eliminated interference from large biomolecules, thus providing high throughput and reliability for the analysis of biological samples.

Functionalized GO materials have also been developed as MALDI matrices. Research by Jiang’s team indicated that nitric acid oxidized G (AOG) is an outstanding MALDI matrix because of its excellent water dispersibility, preventing aggregation in solution or on the MALDI target. Additionally, its oxidation sites are located primarily at the edges of AOG flakes, maintaining the integrity of the AOG π-electron conjugation structure and thereby facilitating high sensitivity in MALDI-MS analysis.^817,818^ Zhang et al. prepared a 4-vinylphenylboronic acid-functionalized GO (GO-VPBA) material (Figure 74a) via atom-transfer radical polymerization (ATRP) and utilized it as a novel matrix for the selective enrichment and analysis of small molecules containing adjacent diols in positive ion mode MALDI-TOF-MS for the first time.^799^ Liang et al. synthesized aggregated GO (AGO) by cross-linking the carboxyl and hydroxyl groups on the surface of GO, then used AGO as a material for the solid-phase extraction of plasma lipids and investigated its application in MALDI-MS and specific neural lipid signal generation.^819^ Because of the cross-linking of carboxyl and hydroxyl groups, AGO possesses potential MALDI-MS matrix properties and interference suppression effects and plays an important role in lipid analysis, especially for TAGs, due to its large, multi-layered structure that preferentially adsorbs hydrophobic molecules.^819^

**Figure 74:**
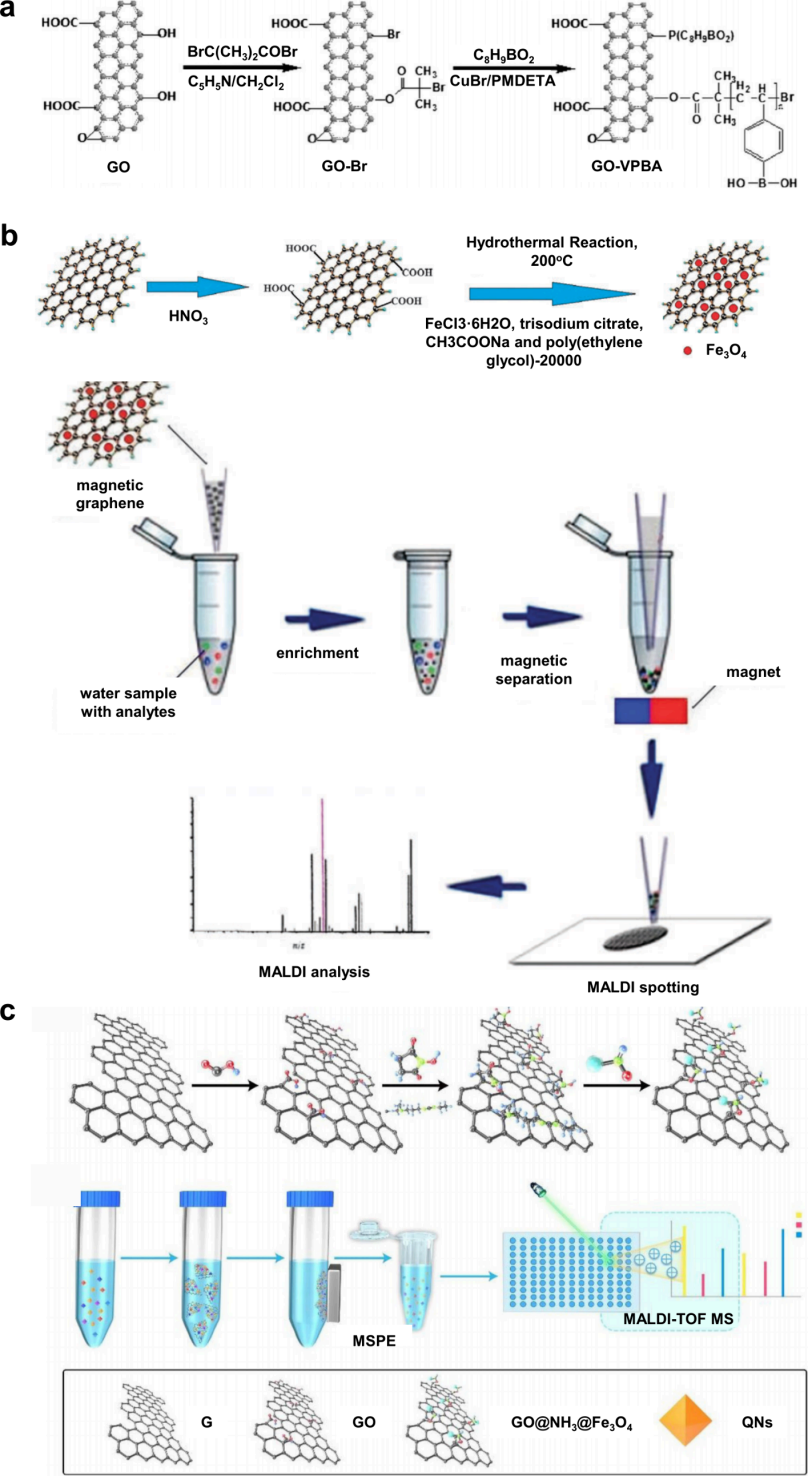
Graphene (G) and graphene oxide (GO) as matrices for MALDI-MS. (a) Flow chart of the synthesis of 4-vinylphenylboronic acid-functionalized GO (GO-VPBA). Adapted and reproduced with permission from ref 799. Copyright 2015 American Society for Mass Spectrometry. (b) Synthetic procedure of magnetic G composites and enrichment procedure using magnetic G as an adsorbent. Adapted and reproduced with permission from ref 820. Copyright 2012 The Royal Society of Chemistry. (c) Schematic illustration of the preparation of GO-functionalized magnetic composites (GO@NH_2_@Fe_3_O_4_) and the operating procedure for magnetic solid phase extraction (MSPE) and MALDI-TOF MS detection of quinolones (QNs). Adapted and reproduced with permission from ref 824. Copyright 2019 Springer-Verlag.

Although G or GO is an excellent adsorbent for sample pretreatment, its hydrophobicity makes it challenging to recover analytes completely.^68^ Introducing magnetic particles to aid in the enrichment process improves separation and recovery efficiency, and the design and synthesis of functional G or GO magnetic composites is a hot research topic.^820^ As shown in Figure 74b, Shi et al. designed and synthesized Fe_3_O_4_/G composite materials for the enrichment and analysis of small molecules, demonstrating their application as novel adsorbents and matrices through MALDI-TOF-MS.^820^ Once prepared, these magnetic G composite materials are stable and can be stored for a long time in suspension or in dried form. Zhang et al. synthesized magnetic G composite material (MAOG) as an adsorbent and matrix for MALDI-TOF-MS analysis, which simplified the enrichment process and eliminated interference, thus establishing a convenient method for detecting nitropolycyclic aromatic hydrocarbons in PM2.5 samples.^821^ In the development of GO magnetic materials, Li et al. used 2-methyl imidazole as an auxiliary method to prepare Fe_3_O_4_/GO nanocomposites, demonstrating their potential as dual-function affinity probes for the selective capture of low-abundance peptides and phosphopeptides.^822^ Yukird et al. utilized Fe_3_O_4_/GO magnetic nanocomposites as selective probes to simultaneously detect and visualize the distribution of pesticides (carbofuran and carbendazim) in pear samples through MALDI-MS.^823^ Additionally, Tang et al. used a GO functionalized magnetic composite material (GO@NH_2_@Fe_3_O_4_) as a magnetic solid-phase extraction adsorbent, combined with MALDI-TOF-MS (using NEDC as the matrix), to analyze 12 quinolone drugs (Figure 74c).^824^ Tang et al. synthesized a novel bismuth oxide-GO (Bi_2_O_3_@GO) hybrid semiconductor matrix, for the analysis of small molecules in negative ion mode MALDI-TOF-MS.^825^ The results demonstrated that Bi_2_O_3_@GO exhibited high signal intensity and reproducibility in small molecule detection, effectively reducing matrix interference, and enabled successful quantitative analysis of glucose in complex samples such as human serum and drinks.

##### 3.2.2.4 Other Carbon-Based Nanomaterials

Many types of graphite have been considered as alternatives to MALDI matrices because they have high UV absorbance and can efficiently disperse energy.^826^ Several forms of graphite with particle or flake sizes ranging from 2 to 150 μm have been used in MALDI-MS, such as graphite in the form of a suspension in glycerol or 2-propanol, graphite powder, graphite target plate, graphite from the “lead” of a pencil, and graphite sheet.

In 1995, Sunner et al. first demonstrated that the use of graphite in glycerol as a matrix facilitated the detection of cytochrome *c* and cytochrome *c* hydrolysis digestion in MALDI-MS.^66^ Dale et al. further developed graphite/liquid matrices and successfully used glycerol/graphite matrices to detect proteins, oligosaccharides, and synthetic polymers of different molecular types by MALDI-MS.^827^ Additionally, Cha and Yeung used an aerosol spray containing 2-propanol-based colloidal graphite as a matrix for the MALDI-MS analysis of standard lipid mixtures containing different components of PCs and GlcCers under high vacuum (HV, ∼1.3 × 10^–4^ Pa) and intermediate-pressure (IP, ∼23 Pa) conditions.^826^ Walton and Mitchell developed a novel rapid detection method using graphite powder as a matrix to analyze radioactive nuclides in environmental samples. The results showed that the induced ionization effect of graphite significantly improved the detection limits and spectral resolution of uranium and lanthanide elements.^828^ Graphite plates have also been widely reported as matrices for the MALDI-MS analysis of various analytes, such as synthetic polymers such as polypropylene glycol and polystyrene,^829^ FAs,^830^ and poly­(methylsilsesquioxane)­s.^831^ Moreover, pencil lead, a mixture of graphite and other components such as clay and wax, has proven to be an effective matrix and calibrator in MALDI-MS. Pencil lead as a matrix enables convenient ionization of a wide range of analyte groups, including peptides, polymers, and actinide metals, and provides a quick method for the quantitative isotope ratio analysis of actinide metals.^832^ Liu et al. developed graphite sheets as a novel matrix that, compared with powdered graphite, reduces the risk of machine malfunction because of the expanded crystal planes, thereby ensuring the reproducibility of MALDI-MS detection. The preparation and operation of the graphite sheets are shown in Figure 75a. Graphite sheets exhibit enhanced UV absorption and ionization efficiency, enabling the detection of a greater number of mass spectral peaks while significantly lowering noise levels, and thus facilitate the effective analysis of small molecules in food samples, such as peptides and AAs.^833^ Additionally, the authors introduced a MALDI-MS method based on graphite black (GCB), which serves as an ion-enhancing nanomaterial that not only significantly decreases noise in the LMW region but also adsorbs volatile organic compounds. This method, involving direct sample droplet application and air drying, effectively analyzes taste and odor-active compounds,^834^ and has been utilized in another study to assess the food quality of different soy sauces.^835^

**Figure 75:**
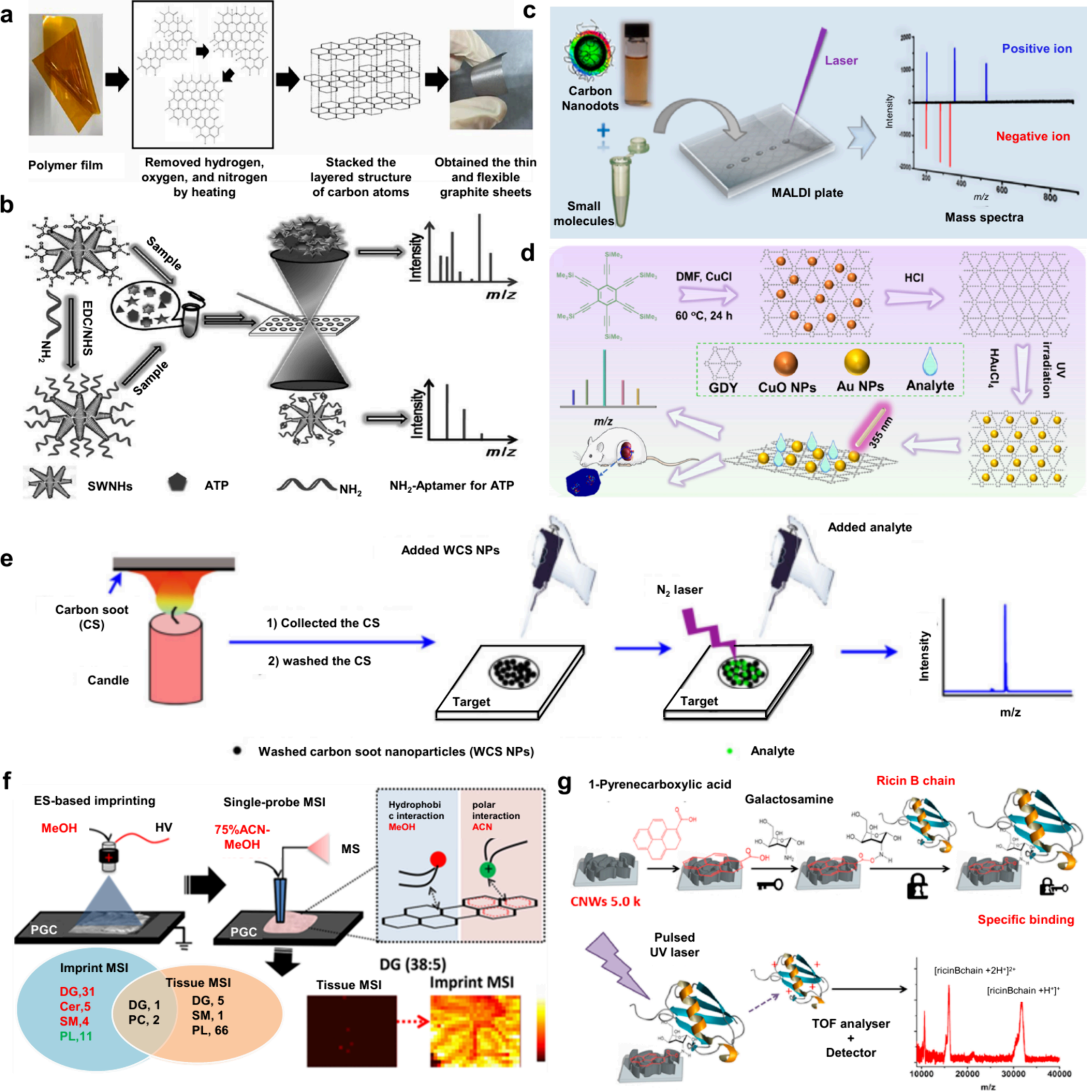
Other carbon-based nanostructures as matrices for MALDI-MS. (a) Schematic diagram of manufacturing processes of graphite sheets. Adapted and reproduced with permission from ref 833. Copyright 2024 The Authors. Published by American Chemical Society. (b) Schematic diagram of the aptamer modification of SWNHs and MALDI-TOF MS analysis using functional SWNHs as the matrix. Adapted and reproduced with permission from ref 840. Copyright 2013 Wiley-VCH. (c) Schematic illustration of the application of carbon nanodots for analyzing low-molecular-weight compounds by MALDI-TOF MS in both positive and negative ion modes. Adapted and reproduced with permission from ref 842. Copyright 2013 American Chemical Society. (d) Scheme of the fabrication and MALDI-MS determination process of the Au/GDY matrix. Adapted and reproduced with permission from ref 846. Copyright 2023 American Chemical Society. (e) Schematic illustration of the application of washed carbon soot nanoparticles (WCS NPs) in MALDI-MS). Adapted and reproduced with permission from ref 847. Copyright 2020, Springer-Verlag. (f) Schematic illustration of the application of porous graphitic carbon (PGC) material to imprint brain tissue sections for the selective enrichment of neutral lipids with polar phospholipids removed. Adapted and reproduced with permission from ref 850. Copyright 2022 American Chemical Society. (g) Chemical modification pathway of boron-doped carbon nanowalls (B-CNWs) by NH_2_-galactosamine and its use for the specific capture of ricin B chain for MALDI-MS detection. Adapted and reproduced with permission from ref 854. Copyright 2022 Elsevier BV.

CNFs are fibers with diameters of 100–200 nm and lengths of 5–20 μm, composed of stacked graphite layers that form a carbonaceous structure. Owing to their large specific surface area, high chemical stability, and unique mechanical properties, CNFs have been widely used as gas adsorbents and catalyst supports.^70^ Recently, Greiderer et al. reported the use of CNF derivatives as carrier materials and matrices for protein detection in MALDI-MS, highlighting their utility for biomaterial enrichment and screening with high protein binding capacity and sensitivity.^836^

NDs (usually 3–10 nm in size), which are members of the carbon family, have many appealing properties such as optical absorption, chemical stability, biocompatibility, and low biotoxicity.^70^ As a carbon NM with great potential, NDs are widely recognized as the hardest substances in nature and have the highest thermal conductivity of all natural materials.^837^ Wei et al. reported the use of NDs as inorganic matrices for MALDI targets and developed an effective and useful method for protein quantification and identification. Owing to the high absorption of laser energy by NDs, the sensitivity of NDs was 3–4 times greater than that of the conventional MALDI sample preparation method.^838^ Furthermore, NDs have proven to be a good platform for protein adsorption and immobilization. Hussain et al. utilized functionalized diamond NPs (diamond-IDA-Fe^3+^/La^3+^) as an immobilized metal ion affinity chromatography (IMAC) material for the reversed-phase enrichment of standard casein and phosphopeptides from real samples such as skimmed milk, egg yolk, and serum, followed by MALDI-TOF-MS analysis.^839^

Carbon nanohornes (CNHs) are end-capped graphite cones with a unique shape that provides abundant internal nanospace and a wide range of chemically functionalized sites.^70^ Among them, single-walled carbon NHs (SWNHs) consist of thousands of closed-end graphite tubes and cone-shaped angles, offering a large surface area, abundant internal nanospace, highly defective angles, and efficient energy transfer.^840^ Ma et al. further explored the application of SWNHs as efficient MALDI matrices. Exploiting the specificity of SWNHs with aptamers and their excellent analytical performance in negative ion mode, they proposed a novel method for the MALDI-TOF-MS determination of biomolecules such as ATP using aptamer-modified single-walled carbon nanocones (Apt-SWNHs) as matrices (Figure 75b).^840^ SWNHs effectively transfer energy under laser irradiation, significantly reducing matrix effects and preventing analyte fragmentation, thereby increasing the signal intensity of biomolecules such as AAs, peptides, and FAs. Notably, this approach capitalizes on both the advantages of SWNHs and the recognition capabilities of aptamers, substantially improving the detection limit of ATP in complex biological samples and providing a novel technique for selective mass spectrometric analysis based on aptamer recognition systems.

Carbon dots (CDs) are nanosized carbon particles with diameters less than 10 nm and are characterized by their quasi-spherical shape, well-defined structure, tunable surface properties, and biocompatibility.^688^ Owing to their strong absorption in the UV range (220–350 nm), ultrasmall size (2–4 nm), and excellent water solubility, CDs are considered ideal matrices for the analysis of small molecules by MALDI-MS.^841^ In 2013, a groundbreaking study by Chen et al. introduced a novel approach utilizing CDs as matrices for MALDI-MS analysis of small molecules, with the straightforward workflow illustrated in Figure 75c.^842^ This work demonstrated the versatile ability of CDs to operate in both positive and negative ion modes in MALDI-TOF-MS, enabling high sensitivity detection of a wide range of LMW compounds, including AAs, peptides, FAs, β-agonists, and neutral oligosaccharides. For instance, the method demonstrated exceptional sensitivity with a detection limit of 0.2 fmol for octadecanoic acid, and excellent linearity in the quantitative determination of glucose and uric acid, with linear ranges of 0.5–9 nm and 0.1–1.8 nm respectively (*R* ^2^ > 0.999).^842^ Gedda et al. reported the use of citric acid derived CDs as a matrix for MALDI-MS detection of the anti-inflammatory drug mefenamic acid.^843^ Compared with DHB, CDs as a matrix successfully eliminated background signals and mefenamic acid signal fragments, and exhibited remarkable sensitivity with low detection limits of 0.51 ng and 0.46 ng for mefenamic acid in serum in positive and negative ion modes, respectively.

Graphdiyne (GD) is a novel type of all-carbon 2D NM with sp and sp^2^ hybridized carbon atoms.^844^ In GD, benzene rings are connected by carbon–carbon triple bonds to form a flat structure, which is considered the most stable structure among various nonnatural diacetylenic carbon allotropes.^845^ Luo et al. utilized GD as a matrix for MALDI-MS and investigated its application in the analysis of small molecules.^845^ A schematic diagram illustrating the use of GD as a matrix for small molecule analysis via MALDI-MS is depicted in Figure 75d. With low background noise and good ion signals in negative ion mode, the GD matrix is suitable for the detection of a wide range of small molecules, such as FAs, AAs, peptides, and drugs. Furthermore, the MALDI-MS analysis of serum samples from liver cancer patients and healthy individuals using a GD matrix revealed that FAs may serve as potential biomarkers for early liver cancer diagnosis.^845^ Recently, Pei et al. developed an Au/GD matrix for MALDI-MS by growing AuNPs on GD through a photoreduction method.^846^ The Au/GD matrix exhibited high D/I efficiency through synergistic enhancements of thermal and electric fields, establishing it as a reliable tool for the determination and imaging of both endogenous compounds and exogenous pesticides.

Wang et al. successfully utilized carbon NPs derived from the incomplete combustion of candles, specifically washed candle soot NPs (WCS NPs), as a matrix for MALDI-MS analysis (Figure 75e).^847^ WCS NPs, comprising diamond, graphite, fullerenes, and amorphous carbon particles,^848^ offer excellent UV absorption, high graphitic quality, efficient D/I, low background signals, reproducibility, sensitivity, and stability, surpassing those of other metal- and carbon-based matrices. They can be widely applied in MALDI-MS for the analysis of carbohydrates, polymers, peptides, drugs, dyes, and FAs, as well as for the quantitative determination of urinary glucose.^847^ A study by Banazadeh et al. investigated the efficiency of the use of graphene nanosheets (GNs) and CNPs as matrices and co-matrices for the MALDI analysis of polysaccharides.^849^ Their results demonstrated that using GNs and CNPs as matrices or co-matrices significantly increased the MALDI analysis signal intensity of various *N*-glycans in biological samples and promoted the formation of in-source fragmentations. Additionally, GNs and CNPs exhibited salt tolerance and, as co-matrices, improved the uniformity of DHB crystals, enhancing the reproducibility and sensitivity of the analysis.^849^

Porous graphitic carbon (PGC) is a type of graphitic carbon material with high surface area, chemical stability, and various surface interactions.^850^ Luo et al. combined an environmental liquid extraction MSI technique with a PGC-based tissue imprinting technique (Figure 75f) to selectively analyze low abundance/low response lipids such as DAGs, ceramides (Cers), and sphingomyelins (SMs) in the rat cerebellum while eliminating interference from high abundance PCs.^850^ Graphitic carbon nitride (g-C_3_N_4_), in its bulk or nanosheet form, is a highly stable isomer of carbon nitride with a graphite-like layered structure and a large surface area, making it a subject of extensive research across diverse fields. Lin et al. utilized g-C_3_N_4_ nanosheets as a matrix for MALDI-MS and successfully detected various LMW compounds, including AAs, nucleobases, and peptides.^851^ They also obtained mass spectra in negative mode without matrix background interference and successfully applied the method to detect 1-nitropyrene in wastewater, with the detection limit reduced to 1 pmol.

Carbon nanowalls (CNWs), often described as vertically oriented multilayer graphene sheets, possess atomically dense graphite edges that serve as potential sites for electron field emission.^852^ Owing to their unique combination of stability, chemical inertness, conductivity, and a high surface-to-mass ratio, CNWs are excellent candidates for biosensing and energy storage applications. Hosu et al. demonstrated that boron-doped CNWs (B-CNWs) are highly effective MALDI-MS matrices for detecting various small compounds relevant to clinical and food industry applications, including carbohydrates, lipids, and peptides.^853^ Notably, they successfully identified glucose in serum and soft drinks; melamine, creatinine, and paracetamol in urine samples, and lecithin in dietary supplements. Additionally, B-CNWs were chemically modified via the amine ethyl galactosamine pathway to enable specific capture of the ricin B chain for laser desorption ionization MALDI-MS analysis, as illustrated in Figure 75g. This approach successfully facilitated the MALDI-MS detection of two proteins: cytochrome C (12 kDa) and the ricin B chain (32 kDa).^854^

##### 3.2.2.5 Summary

In summary, this section highlights the applications of carbon-based NMs in MALDI-MS analysis in recent years. Carbon-based nanostructures are ideal matrices for small molecule analysis in MALDI-MS, and exhibit strong absorption in the 250–350 nm wavelength range, making them compatible with various types of lasers.^688^ Carbon-based NMs offer multiple advantages in MALDI-MS analysis, including: (1) enhancing D/I efficiency and sensitivity, reducing background noise, and expanding applications to small molecules, (2) serving as efficient adsorbents and, when combined with their high performance in MALDI-MS to improve analytical sensitivity, and (3) further improving detection selectivity through the functionalization of carbon-based NMs. However, most methods have been evaluated in the laboratory, and their application potential in complex real samples needs further validation. Overall, carbon-based NMs hold great promise for MALDI-MS analysis, but further research and improvements are necessary to fully exploit their potential.

#### 3.2.3 Silicon-Based Nanostructures

Silicon-based NMs offer effective background suppression for MALDI-MS detection because of their excellent chemical stability, low thermal conductivity, and unique electronic and optical properties^75^ The use of silicon-based MALDI-MS for small molecule analysis first emerged in the 1990s in the form of porous silicon (pSi), which was later developed into other forms such as silica NPs (SiO_2_ NPs), silicon nanowires (SiNWs), NAPAs, and so forth.^71,72,243^

##### 3.2.3.1 Porous Silicon

Porous silicon (pSi) is a semiconductor that absorbs UV light. It is an amorphous porous substrate with a large surface area, pore sizes ranging from tens to hundreds of nanometers and pore depths of less than 500 nm.^855^ The traditional method of preparing silicon-based surfaces involves electrochemical etching, resulting in disordered porous silicon surfaces. Therefore, this method is referred to as desorption/ionization on silicon (DIOS).^71^

DIOS was first used in MALDI matrix development by Wei et al. in 1999, using pSi as a substrate to capture analytes deposited on the surface, as shown in Figure 76a. This inorganic matrix approach allows the MALDI-MS detection of compounds such as peptides and small drug molecules at concentrations as low as femtomole and attomole levels, with little or no mass interference from matrix ions.^71^ In DIOS-MS, analytes from solution or tissue sections are directly deposited onto the surface of nanostructured porous silicon without the need for additional organic matrices.^856^ Porous silicon serves as an efficient medium for desorbing compounds and generating intact ions in the gas phase, effectively absorbing energy from UV laser irradiation and transferring it to the adsorbed analytes while also protecting the analyte molecules from direct laser irradiation-induced fragmentation.^857,858^ Liu et al. used DIOS technology to achieve cellular-level imaging of small molecules in biological tissues on porous silicon substrates.^859^ In this study, PCs and propidium iodide were analyzed by MALDI-MS to serve as membrane and nuclear markers, respectively, in mouse liver tissues, enabling the indirect visualization of mammalian cells, *i.e.*, human embryonic kidney 293 cells. Rudd et al. utilized DIOS to observe changes in the distribution of metabolites in muricid mollusk (*Dicathais orbita)* tissues and investigated the biological roles of two classes of secondary metabolites, brominated indoles and choline esters, in mollusk reproduction.^860^ Kraj et al. applied an improved DIOS technique, D/I on silicon dioxide, to analyze catecholamines in the immune system.^861^ To date, DIOS-based MALDI-MS has been widely used for the analysis of various compounds, such as, small organic molecules, proteins,^862^ hydrophilic synthetic polymers,^857^ and hydrophobic polyester polymers.^863^

**Figure 76:**
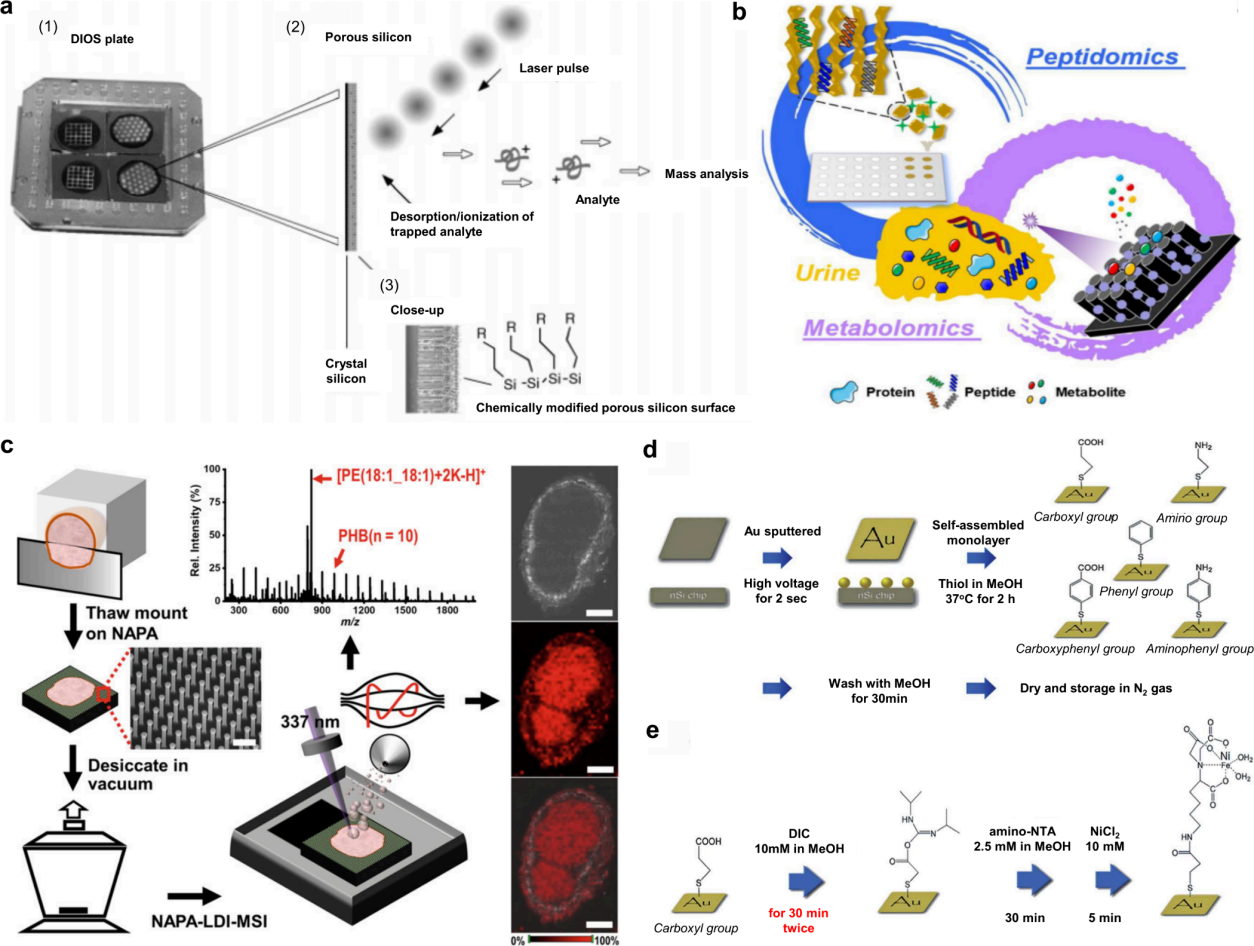
Silicon-based nanostructures as matrices for MALDI-MS. (a) The experimental setup for DIOS-MS includes placing four porous silicon plates on a MALDI plate with photopatterned spots or grids, utilizing a silicon-based laser desorption/ionization process, and demonstrating the cross-section of porous silicon with surface functionalities post-hydrosilylation. Adapted and reproduced with permission from ref 71. Copyright 1999 Macmillan Magazines Ltd. (b) Workflow of urine multi-omics analysis based on porous Si microparticles and fluorinated ethylene propylene coated silicon nanowire (FEP@SiNW) chips. Adapted and reproduced with permission from ref 884. Copyright 2024 Ivyspring International Publisher. (c) Workflow of sample preparation and untargeted silicon nanopost arrays (NAPA)-LDI-MSI of biological tissues. Adapted and reproduced with permission from ref 892. Copyright 2022 The Authors under exclusive license to Springer Science Business Media, LLC part of Springer Nature. (d,e) Schematic illustration of grafting (d) NH_2_, COOH, phenyl, phenyl-NH_2_, and phenyl-COOH and (e) IMA-Ni^2+^ functional groups on the nanostructured silicon (nSi) chip modified by the self-assembled monolayer approach. Adapted and reproduced with permission from ref 900. Copyright 2012 Royal Society of Chemistry.

Based on the original DIOS method, Siuzdak et al. further developed nanostructure-initiator MS (NIMS), which uses liquid initiators to facilitate desorption.^243^ The fundamental difference between NIMS initiators (silane, siloxane, or disiloxane based) and MALDI matrices is that initiators do not absorb UV energy, most do not ionize, and analytes do not co-crystallize with the initiators.^864^ During the NIMS D/I process, pSi absorbs laser energy to rapidly heat the surface, utilizing captured initiators on nanostructured surfaces to release or ionize intact analytes adsorbed on the surface.^865^ Therefore, the NIMS surface is stable in ambient air, exhibits better reproducibility, enables direct analysis of biological fluids and tissue imaging, and allows a significant expansion of the mass range.^866^ Siuzdak’s team utilized NIMS to visualize lipids (*m*/*z* 700–800) in mouse embryo tissue slices^243^ and further extended this method to clinical applications for analyzing exogenous metabolites (including clozapine, *N*-desmethylclozapine, nicotine, ketamine, and their metabolites) in human brain tissues and biofluids.^867^ The surface morphology of the NIMS substrate plays a crucial role in the D/I process of analytical molecules, thereby determining the sensitivity of the substrate to convert intact molecules into gas-phase ions.^864,868^ Gao et al. examined the impact of surface morphology on NIMS sensitivity by creating nanostructured surfaces with 4–12 nm pores using constant current electrochemical etching, and reported that the sensitivity to different analytes was dependent on pore size.^869^

The surface properties of NIMS can be manipulated or modified through the use of different chemical initiators, expanding the detection range of analytes and the ability to extract comprehensive information from complex chemical mixtures.^76^ For instance, Amantonico et al. used 3-aminopropyldimethylethoxysilane as an initiator to detect phosphorylated metabolites in the femtomole range and reported that amino-containing initiators can facilitate ion formation in negative ion mode.^870^ Further modification of the NIMS surface can be achieved by coating it with cationizing agents such as NaCl or AgNO_3_, enabling the analysis of molecules such as carbohydrates and steroids that are difficult to detect with other MS methods.^871^ Patti et al. combined NIMS with NaCl or AgNO_3_ spray deposition, providing a uniform environment with cation sources ([M+Na]^+^ or [M+Ag]^+^) for MALDI-MS imaging of carbohydrates in a *Gerbera jamesonii* flower stem and cholesterol in mouse brain tissue.^871^ More recently, The Northen team utilized low-temperature inductively coupled plasma etching to create black silicon, which serves as a new NIMS surface with enhanced sensitivity because of its fine pillar-like structures.^872^ They successfully assessed its relative NIMS activity using various molecules including agmatine, Arg, adenosine, palmitoylcarnitine, and verapamil. Subsequently, the same group found that morphologically controlled NIMS enables the selective ionization of metabolites, with high porosity enhancing the sensitivity for small molecules and low porosity leading to relatively high sensitivity for large molecules.^869^

Nimzyme is a unique application of NIMS for the analysis of biological fluids, providing a method for measuring enzymatic activity in biological samples. In Nimzyme, single or multiple fluorine-labeled enzyme substrates are noncovalently immobilized on the porous silicon surface, where they favorably interact with the perfluorinated initiator compounds filling the nanopores through fluorous affinity interactions.^76^ After the immobilized substrates are incubated with enzymes or biological fluids and subsequently washed, the fluorine-labeled substrates, which are insoluble in aqueous buffers, remain on the surface.^866^ When Nimzyme was first reported, fluorine-labeled lactose-immobilized substrates (*m*/*z* 1074.30) were incubated with lysates from *E. coli* or microbial community lysates collected from Yellowstone National Park, and the extent of substrate conversion to products was measured on the basis of observed mass changes on the NIMS surface.^873^ Deng et al. combined the Nimzyme method with an accurate mass tagging approach, utilizing unique perfluorinated tails on each reactant to analyze the stereospecific reaction pathways for three stereoisomers (maltose, lactose, and cellobiose) that produce the same product glucose, on a traditional MALDI-TOF instrument. This method is applicable not only to purified enzymes but also to crude cell lysate products.^874^ The sensitivity of the Nimzyme assay is comparable to that of fluorescence-based assays but with much lower noise. Additionally, the Nimzyme assay can operate over a wide range of pH values and temperatures, expanding its detection capabilities beyond those of standard biological conditions, which is particularly useful in industrial applications.^873^

##### 3.2.3.2 Silica Nanoparticles

Since pSi has demonstrated wide value in MALDI-MS applications, silicon and silicon-containing NPs have become reliable alternatives to organic-based matrices for MALDI.^75^ SiO_2_ NPs are easily synthesized through the well-known sol-gel method.^875^ A sol-gel is a polymer structure formed by a siloxane backbone (Si-O), which can be formed as bulk or thin films for the preparation of SiO_2_ NPs, magnetic core-shell NPs (CSNPs), and thin layers of silica, among other materials.^688^ For instance, Dupre et al. used a solution of ammonium hydroxide in MeOH and tetraethoxysilane as precursors for SiO_2_ NPs, enabling cost-effective and easily prepared SiO_2_ NPs that exhibited high sensitivity in the femtomolar range for high-throughput MALDI-MS analysis of peptide mixtures.^875^ Wen et al. utilized silicon nanopowder (5–50 nm) as a matrix and developed optimized procedures for analyzing a wide range of analytes in MALDI-MS, including small molecules such as drugs, peptides, pesticides, acids, and others.^876^ The method demonstrated relative tolerance to salt contamination and was capable of direct analysis of morphine and propaphenone in untreated urine, as well as triazine herbicides in a soil extract. Araujo et al. covered the hand-cut sections of stems of two *Eucalyptus* species with a layer of silica and directly analyzed them using MALDI-MS. They tracked the distribution of soluble lignin subunits and structures in the profiles using information available in the literature and performed relative quantification using software imaging.^877^ Additionally, CSNPs, with a magnetic iron oxide nanoparticle core and a thin layer of silica dioxide shell, were also used as inorganic matrices for MALDI-MS experiments. Briefly, CSNPs are produced through the sol-gel process, where magnetite particles are coated with a thin silica layer using TEOS as the silica source.^75^ Zhu et al. employed different CSNP approaches, replacing the magnetic iron oxide core with AuNPs (ranging from 18–50 nm) and incorporating an ultra-thin silica dioxide shell (≈2–4 nm) on the outer layer (Au@SiO_2_ CSNPs), which proved effective in the analysis of numerous compounds, especially small functional molecules and polymers.^878^ Li et al. employed SiO_2_@Au nanoshells combined with microvolume plasma to obtain metabolic fingerprints for coronary artery disease using MALDI-MS, thereby circumventing the need for complex sample preprocessing. Machine learning analysis of the extracted metabolic information revealed high sensitivity and specificity in distinguishing patients with coronary artery disease from control subjects in the validation cohort.^879^ Additionally, Choi et al. developed a graphene-coated silicon wafer (G/SiO_2_) for sensitive, reproducible, and accurate measurement of proteins in MALDI-TOF MS analyses. Their study demonstrated that the G/SiO_2_ platform exhibited high ionization efficiency and excellent reproducibility in general protein analysis and identification of disease-related target proteins, likely due to because of its ability to facilitate uniform sample crystallization on a flat graphene surface.^880^

##### 3.2.3.3 Silicon Nanowires

In the field of silicon nanostructures, SiNWs prepared through vapor–liquid–solid growth or metal-assisted chemical etching techniques have successfully served as highly sensitive platforms for the D/I MS analysis of small molecules, lipids, peptides, and protein digests.^881^ SiNWs exhibit the basic attributes of porous silicon but with a larger surface area and greater capacity, and they have been widely utilized as enhanced porous silicon surfaces. Their sensitivity is notably impacted by laser energy, surface chemistry, nanowire diameter, length, and growth orientation.^882^ Piret et al. achieved one-step fabrication of SiNWs with controllable diameter and length by chemically etching crystalline silicon in an HF/AgNO_3_ aqueous solution. Through optimization, they successfully prepared SiNWs (average diameter, 20–100 nm; length, 2.5 μm) as MALDI matrices, and achieved remarkable MALDI-MS performance for a wide range of analytes, including small molecules, peptides, and BSA digests, by combining surface morphology and surface chemistry control.^882^ Liu et al. have proposed a membrane-mediated imprinting MSI strategy that employs a isoporous nuclepore track-etched membrane as the mediating imprint layer, selectively transporting metabolites through uniformly vertical pores to the SiNWs array.^883^ This strategy, in contrast to conventional direct imprinting techniques, significantly reduces sample preparation time to just 2 minutes while eliminating the adsorption of large biomolecules and preventing the lateral diffusion of metabolites, resulting in an increase in detected lipid peaks in renal tissue from 46 to 113; moreover, the confinement effect of the pores enhances resolution, allowing for clear delineation of tumor margins in liver cancer and accurate classification of its various subtypes. Moreover, Jiang et al. utilized fluorinated ethylene propylene-coated silicon nanowire chips (FEP@SiNWs) as a MALDI-MS platform to perform nontargeted metabolic fingerprinting of urine samples using a tip-contact extraction method. The workflow for urinary multiomics analysis based on porous silicon microparticles and FEP@SiNWs is shown in Figure 76b. Porous silicon microparticles are employed for urine peptide capture, whereas FEP@SiNWs serve as the matrix for revealing hidden peptide spectra in urine samples using MALDI-TOF-MS.^884^

##### 3.2.3.4 Nanofabricated Silicon Nanopost Array

With the utilization of SiO_2_ NPs and SiNWs, nanofabricated silicon NAPAs have also been tested as inorganic MALDI matrices. NAPAs are highly uniform MALDI platforms with broad molecular coverage, ultra-trace sensitivity, and tissue imaging capabilities in MALDI-MS.^885,886^ Additionally, owing to advancements in photolithography, these NAPAs can be modified and adjusted structurally to provide localized electric field enhancement, resulting in higher ion yields while requiring lower laser fluence.^887^ To date, NAPAs have been employed in MALDI-MS for the analysis of various samples, *e.g.*, peptides, metabolites, drugs, and lipid and metabolite imaging in tissue sections.

The Vertes et al. reported the use of silicon microcolumn arrays (LISMAs)^888^ and NAPAs^889^ as alternative DIOS substrates, demonstrating their ability to induce structure-specific fragmentation. By the laser-induced construction of LISMAs and electron beam lithography introduction of NAPAs as matrix-free MALDI-MS media, significantly improved ion yields were achieved. Recently, they proposed another NAPA platform, namely, an elevated bowtie (EBT) array, which adjusts ionization efficiency by altering the apex angle through the addition of triangular chromium features on top of the silicon pillar pairs.^887^ Fincher and colleagues overcame the limitations in ion suppression of neutral lipids (*e.g.*, TAGs) by PLs (*e.g.*, PCs) in MALDI ionization and imaging using the NAPA platform.^890^ They successfully applied this technique to analyze skin samples from patients with hidradenitis suppurativa, enabling the detection and imaging of different neutral lipids. Furthermore, the NAPA platform enhanced the ionization of certain lipids in mouse brain tissue, such as hexosylceramides (HexCers) and PEs, in mouse brain tissue.^885^ Recently, Samarah et al. team utilized NAPA as a MALDI platform (Figure 76c) with ultra-trace sensitivity and molecular imaging capabilities to analyze the spatial and size distributions of polyhydroxybutyric acid, polyglutamic acid, and polysaccharide oligomers in soybean root nodule sections.^891^ They also achieved imaging of biopolymers, metabolites, lipids, and bio-oligomers in biological tissues through NAPA-based MALDI-MS.^892^

##### 3.2.3.5 Other Silicon-Based Nanostructure

In addition to the aforementioned pSi surfaces, silicon NPs, SiNWs, and NAPAs, other silicon-based nanostructures such as nanoscale calcinated silicate film, mesoporous silica foam (MCF), nanofilament silicon (nSi), and microalgal biomineralized cell walls, have been extensively developed and applied in inorganic MALDI-MS.

Duan et al. developed a method using nanoscale calcinated silicate film on a gold substrate for on-plate desalting and direct MALDI-MS analysis of peptides, effectively removing contaminants and improving sensitivity for low femtomole level peptide detection.^893^ Furthermore, they enhanced the fabrication process of nanofilms by forming a hydrophobic OTS monolayer on a calcined nanofilm deposited on a gold substrate. This modification resulted in rougher surfaces that significantly enhanced peptide recovery and facilitated the selective capture of hydrophobic protein components, thereby providing an innovative strategy for the specific enrichment of peptides. This on-plate desalting technique combined with MALDI-MS was successfully employed for peptide analysis from protein digests.^894^

MCF is a nanomaterial synthesized from a polymer template composed of spherical voids with a diameter of 22–42 nm, interconnected by approximately 10 nm “windows”,^895^ that serves as a catalyst support for separations involving large molecules and for carbon capture^896^ and storage to reduce the emission of carbon dioxide into the atmosphere after combustion.^897^ Barros et al. evaluated the feasibility of using MCF in MALDI-MS analysis of fingerprints by pretreating MCF as an EtOH suspension or a magnetic powder mixture. Fingerprint analysis revealed ions associated with endogenous and exogenous molecular components, including lipids, possibly llipids from human sebum, and quaternary ammonium cations commonly found in cosmetics.^898^

Tsao et al. used electron-beam deposition to sputter 3 nm AuNPs onto the surface of silicon and then etched the surface with HF, H_2_O_2_, and EtOH solutions to form nSi.^899^ By employing the dynamic electrowetting method of nSi and controlling the preparation and etching conditions, they achieved a high surface area and porous morphology of nanowire fields, effectively preventing air entrapment and enhancing the interaction capacity with sample molecules to increase the sensitivity of MALDI-MS.^899^ Additionally, as is shown in Figure 76d, the Tsao team performed surface chemical modification on nSi, chemically modifying the nSi surface to be hydrophilic, hydrophobic cationic, anionic and immobilized metal ion surfaces, thereby improving the absorption specificity and MS performance.^900^ Further studies revealed that the combination of surface modification and nSi-MS technology enables the selective detection of specific compounds in peptide mixtures by MALDI-MS.

Using the ability of biological mineralization in nature to form NMs can simplify the synthesis of silicon-based materials. Diatom frustules are composed mainly of hydrated SiO_2_ tightly bound to peptides and polyamines,^901^ forming a species-specific framework with pore diameters of up to 40 nm.^902^ Jaschinski et al. utilized the biomineralized cell walls of microalgae (*i.e.*, the diatom *Thalassiosira pseudonana* and the dinoflagellate *Prorocentrum minimum*) as an ionization-supporting platform and successfully achieved MALDI-MS detection of molecules, including PEG600, d-sphingosine, and mannose.^903^ They also evaluated the ionization efficiency of commercially available celite, a biogenic nanomaterial primarily composed of mainly amorphous silicon dioxide from fossilized diatoms with remaining patterned particles, and demonstrated its effectiveness in ionization.^903^ Further chemical modification of purified diatom cell walls can generate a material that effectively enhances the sensitivity of biogenic nanomaterial-enhanced MALDI-MS. Jaschinski's team modified bionanostructures from the cell walls of diatoms using 1*H*,1*H*,2*H*,2*H*-perflourooctyldimethylchlorosilane or pentafluorophenylpropyldimethylchlorosilane. Chemically modified diatom cell walls significantly enhance ionization in MALDI-MS, increasing signal intensity by up to 25-fold compared with that of unmodified diatom cell walls.^904^ They allow the direct analysis of small analytes without interference and enable the investigation of crude drug solutions (such as the aspirin complex and IbuHEXAL) without additional sample preparation.

##### 3.2.3.6 Summary

In summary, silicon, which is one of the primary materials for manufacturing inorganic MALDI matrices, can be designed with controllable morphology and properties for MALDI-MS because of its biocompatibility and ease of stability, derivatization, and functionalization characteristics.^75^ These structures include pSi surfaces, silicon NPs, SiNWs, NAPA, MCF, nSi, and others, and they exhibit characteristics such as low limit of detection (LOD), minimal fragmentation, and suitability for both positive and negative ion modes, making them widely applicable in inorganic matrix-based MALDI-MS.^688^ Surface functionalization of silicon nanostructures can enhance specific bonding with analytes, allowing the detection of previously undetectable molecules and improving ionization efficiency.^75^ This approach enables selective detection, enrichment, desalting, and matrix-free analysis in a single run, making it a promising option for developing new MALDI-MS technologies and applications.^688^ Thus, targeted analysis can be designed by selecting appropriate functionalization methods and/or combining various functionalization methods to reduce unwanted signals or to obtain a broader detection range. In addition, all of these surface-modified, matrix-free methods can be used with organic matrices to extend the detection range from small molecules to large molecules. Although current silicon-based nanomaterial manufacturing methods have been continuously improved from wet to dry processes, there are still challenges regarding personal safety risks and surface oxidation, which can affect reproducibility. These limitations may hinder the widespread use of silicon-based NMs in MALDI-MS applications.^76^ To overcome these issues, exploring advanced preparation techniques and alternative materials for silicon could provide potential solutions.

#### 3.2.4 Organic Frameworks

##### 3.2.4.1 Metal-Organic Frameworks

Metal-organic frameworks (MOFs), also known as porous coordination polymers, are a class of porous crystalline materials that assemble metal ions/clusters and organic linkers through strong coordination bonds.^905^ MOFs possess high porosity, large surface areas, tunable morphologies, and diverse functionalities, making them versatile materials for gas separation and storage, catalysis, electrochemical sensing, biomedical, and other applications.^905^ Despite the successful synthesis of thousands of MOFs, their potential as MALDI matrices for the analysis of biomolecules is still in the early stages of investigation.

In 2013, Huang et al. first introduced MOFs as MALDI matrices and systematically studied the effects of several MOFs, including MIL-100­(Fe), MIL-100­(Cr), MIL-100­(Al), MIL-101­(Cr), DUT-4­(Al), DUT-5­(Al), and CYCU-3­(Al), on MALDI-MS performance using five PAHs (including, anthracene (Ant), pyrene (Pyr), benzo­[*a*]­anthracene (BaA), chrysene (Chr), and benzo­[*a*]­pyrene (BaP)) as test analytes.^906^ The researchers reported that MIL-100, with its stable structure and affinity-enhancing metal ions, effectively reduced background noise and improved ionization for analytes. Different MOFs showed no background signals, but the metal type influenced the signal intensity and reproducibility of PAHs, with MIL-100­(Fe), demonstrating excellent reproducibility because of its cage-like 3D structure.^906^ As part of this ongoing research, Huang et al. further explored other types of MOF NPs (MIL-100­(Fe), MIL-100­(Cr), MIL-100­(Al), MIL-101­(Cr), and UiO66­(Zr)) as potential matrices for analyzing highly polar carbohydrates and peptides by MALDI-MS.^907^ They reported that MIL-100­(Fe) showed excellent reproducibility and low background interference, making it a promising matrix for the analysis of monosaccharides/disaccharides, peptides, and complex starch digestion, in MALDI-MS and MS/MS experiments. Han et al. utilized MIL-101­(Cr), an MOF with a high molecular weight, a π-conjugated 3D structure, coordinatively unsaturated chromium sites (CUS), and strong UV absorption, which resulted in a high S/N ratio and significant eliminateion of background peaks during small molecule analysis.^908^ Common flavanols such as quercetin, daidzein, genistein, and naringenin in food and natural products were successfully identified by MALDI-MS using MIL-101­(Cr) as a matrix, which proved to be excellent for the sensitive detection of quercetin, with high sensitivity, good salt tolerance, and reproducibility. Furthermore, various MOFs, including Ti-MOF,^909^ Fe-MOF,^907^ and Zn-MOF,^910^ have been utilized in MALDI-MS for small molecule analysis. Despite their potential, MOF materials face limitations such as poor chemical stability, inadequate processability, and low electronic conductivity, which restrict their practical applications and lead to subpar performance in certain fields.^905^

One approach to overcoming these issues is to introduce additional functional components to construct multicomponent hybrids containing MOFs. By combining MOFs with other functional elements such as carbon materials, metal NPs, and biomolecules, a diverse range of MOF-based hybrids have been developed. The integration of MOFs with other materials can increase their ionization efficiency, thereby providing superior D/I performance.^905^

Lu et al. reported that the use of graphene-based materials and MOFs alone is ineffective for detecting steroids as matrices, but their composite materials exhibit higher sensitivity and lower interference.^911^ They utilized a composite material called MG@UiO-66, which combines 3D-MG and zirconium-based MOF as adsorbents and matrices for detecting steroids in MALDI-TOF MS. This method significantly reduces the LOD of steroids in environmental water samples, providing an effective means of environmental monitoring.^911^ By directly carbonizing MOFs, Shih et al. developed nanoporous carbon materials (cMIL-53 and cCYCU-3) (Figure 77a), which showed promising performance as matrices for MALDI-MS. These carbonized MOFs were successfully used for analyzing a wide range of small molecules, including polar compounds such as carbohydrates, phenolic acids and peptides, as well as nonpolar compounds such as phthalate esters and PAHs.^912^

**Figure 77:**
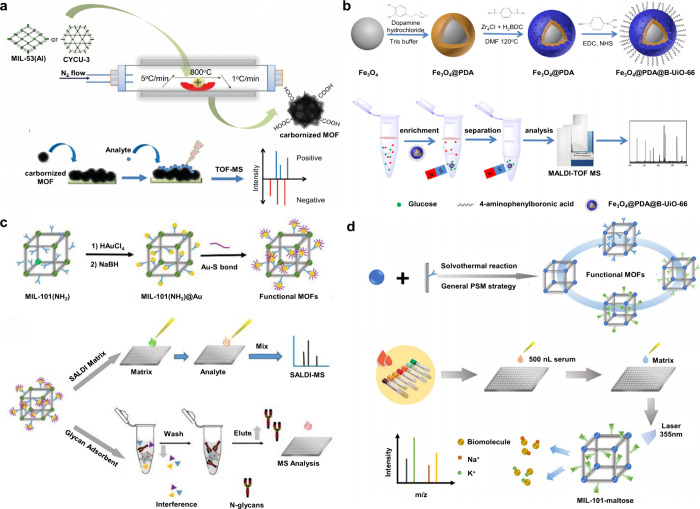
Metal-organic frameworks as matrices for MALDI-MS. (a) Carbonization process of nanoporous carbons derived from metal-organic frameworks (cMIL-53, cCYCU-3) and sample preparation process of MALDI-MS. Adapted and reproduced with permission from ref 912. Copyright 2016 Wiley-VCH. (b) Synthesis route of Fe_3_O_4_@PDA@B-UiO-66 composites and glucose enrichment process. Adapted and reproduced with permission from ref 914. Copyright 2024 Springer Nature. (c) Generic MOF PSM method for functional MOF synthesis and the multifunctional behavior of MIL-101­(NH_2_)@Au-Cys in MALDI-MS analysis with simultaneous enrichment of *N*-glycans. Adapted and reproduced with permission from ref 919. Copyright 2019 Royal Society of Chemistry. (d) Schematic of the synthesis of MOFs, and applications of MIL-101-maltose in small biomolecule analysis. Adapted and reproduced with permission from ref 920. Copyright 2022 The Authors under exclusive license to Springer-Verlag.

Luo et al. developed a composite material, UiO-66-(SH)_2_@Pd NPs, for use as a matrix in MALDI-MS detection. By incorporating Pd NPs into UiO-66-(SH)_2_, they obtained single-dispersed Pd NPs embedded in the MOF structure, which exhibited high UV absorption capacity and stability. This increased the ionization efficiency of oligosaccharides and enabled the identification and relative quantification of two disaccharide isomers.^913^ Li et al. synthesized a boronic acid-modified multifunctional Zr-based MOF (Fe_3_O_4_@PDA@B-UiO-66), as shown in Figure 77b. This MOF is characterized by high ionization efficiency, a large surface area, a low matrix background, and a porous structure ideal for MALDI-TOF MS applications.^914^ They successfully detected glucose with high sensitivity using this MOF as a matrix, with a low LOD of 58.5 nM, providing a simple and effective approach for quantitative analysis in complex samples. In another work, Li et al. used an improved thermal annealing method to synthesize a magnetic Zr-based MOF (Fe_3_O_4_@ PDA@ZrMOF) with low background interference, high D/I efficiency, excellent signal reproducibility, ultrahigh surface area, and magnetic responsiveness. This material was successfully utilized for the analysis and detection of nitrophenol compounds in MALDI-TOF MS negative ion mode.^915^ Lin et al. developed a magnetic nanocomposite material (Fe_3_O_4_@ZIF-8 MNCs) by coating magnetic NPs with ZIF-8 and used it as a matrix in negative ion MALDI-TOF-MS. This material showed improved signal intensity and salt resistance in small molecule analysis and was also explored as an affinity probe for the quantitative analysis of trace His concentrations and direct ionization by MALDI-MS.^916^ Chen et al. developed a core-shell magnetic nanosphere, Fe_3_O_4_@NTU-9, that effectively captures exosomes from human urine, facilitating rapid solid-liquid separation and increasing MALDI-MS ionization efficiency. This innovative approach successfully distinguished healthy controls from clear cell renal cell carcinoma (ccRCC) patients in 176 urine exosome samples, achieving over 94% accuracy and significantly improving diagnostic capabilities for the disease. Additionally, Sun et al. synthesized a metal oxide nanomaterial derived from an MOF, namely, CoFeNMOF-D, which was utilized to assist in extracting serum metabolic fingerprints from COPD patients and healthy controls by MALDI-MS.^917^ Through machine learning algorithms, they successfully differentiated between the COPD group and the healthy control groups, identifying four significantly downregulated potential biomarkers. Moreover, Yin et al. synthesized core-shell structured UiO-66-(OH)_2_@UiO-66-NH_2_ NPs using a simple hydrothermal method, employing them as both an adsorbent and a matrix for MALDI-TOF-MS in the quantitative analysis of rhubarb anthraquinones in plasma.^918^

Ma et al. utilized MIL-101­(NH_2_) as a matrix and employed post-synthetic modification to design multifunctional MOFs.^919^ As shown in Figure 77c, by immobilizing gold NPs on the MOF surface and modifying it with various functional groups, the D/I efficiency of the analytes was enhanced. A thorough exploration of the experimental conditions revealed the optimal performance, and the cysteine-functionalized MOF (MIL-101­(NH_2_)@Au-Cys) was identified as an excellent MALDI-MS matrix with a detection limit in the fmol range, good intersample reproducibility, and high sensitivity and selectivity for *N*-glycan enrichment.^919^ In addition, Ma et al. synthesized various MIL-101-derived NMs including MIL-101, MIL-101-NH_2_, MIL-101-N_3_, and MIL-101-maltose, through a one-pot solvothermal method or the PSM strategy, and investigated their performance as MALDI-MS matrices (Figure 77d).^920^ Among them, MIL-101-maltose showed high ionization efficiency in both positive and negative ion modes, low background interference below 700 Da, good salt resistance, and excellent reproducibility. This method was successfully validated for rapid monitoring of glucose in the serum of diabetic patients and healthy controls, revealing the potential application of MIL-101-maltose in oligosaccharide analysis.^920^ Using MALDI-MS, Zheng et al. developed a method to synthesize functionalized MOFs for the selective enrichment and direct detection of aldehyde compounds.^921^ They introduced -NHNH_2_ groups into NR_3_^+^-MOF through electrostatic adsorption, resulting in NH_2_NH-MOF which selectively enriched aldehydes and formed *p*-hydrazinobenzenesulfonic acid-functionalized aldehydes (NH_2_NH-aldehydes), resulting in a mass increase of 170 Da compared with the initial unfunctionalized aldehydes.

In summary, MOFs have attracted significant attention because of their large surface area, strong UV-visible absorption capability, and ease of preparation of homogeneous samples. However, among the numerous MOFs studied, only a few have been proven to be effective matrix materials for MALDI-MS.^919^ Certain MOFs exhibit significant promise as MALDI-MS matrices, because of their high surface area, large pore structure, accessible coordinatively unsaturated chromium sites, and excellent chemical and thermal stability.^908^ Nonetheless, the inherent lack of sufficient mobile protons in many MOFs restricts their applicability in MALDI-MS.^919^ In recent years, surface functionalization of materials and the incorporation of other functional components such as carbon materials, metal NPs, and biomolecules have been explored to enhance matrix hydrophilicity and proton transfer efficiency, leading to improved ionization efficiency and mass spectral signal intensity. Therefore, there is an urgent need to systematically explore and develop MOFs and the guiding principles for their functionalization strategies to expand the range of analytes and screen for MOFs suitable for MALDI-MS applications.

##### 3.2.4.2 Covalent-Organic Frameworks

Another well-known type of porous crystalline framework is covalent organic frameworks (COFs), which are a new class of network polymers formed by covalent bonds connecting organic building units, creating a structure composed of strong covalent bonds between elements such as B, C, N, O, and Si.^905^ COFs possess unique features such as low density, high surface areas, high charge carrier mobility, tunable pore structures and skeleton functionalities, and significantly enhanced thermal and chemical stability.^922,923^ Since the pioneering work was first reported by Yaghi et al. in 2005,^924^ COFs have shown great potential in various fields and have been widely applied in sensing, catalysis, separation, optoelectrical device fields, and more.^688^ As a promising matrix material, the organic units in COFs contain abundant π–π stacking structures,^925^ which provide efficient UV–visible light absorption and covalent bonds that enhance thermal and chemical stability, thereby reducing interference from background signals.^926^ However, to date, studies on the effective use of COFs as matrix materials in MALDI-MS have been limited.

In 2018, Feng and Xia reported the first example of using COFs as a MALDI-TOF-MS matrix.^927^ Specifically, they utilized spherical COF called TpBD, synthesized from 1,3,5-triformylphloroglucinol (Tp) and benzidine (BD), which demonstrated unique morphology, strong optical absorption properties, and excellent chemical and thermal stability. When various small molecules were tested, the results revealed that COFs had advantages over traditional matrix DHB in terms of higher D/I efficiency, enhanced signal intensity, good salt resistance, and freedom from matrix-background interference.^927^ Wang et al. used COF-LZU1, synthesized from 1,3,5-triformylbenzene and *p*-phenylenediamine, as an adsorbent and matrix for MALDI-TOF MS, achieving rapid analysis of fluorochemicals.^928^ COF-LZU1, with its large surface area, high porosity, and suitable hydrophobicity, enables rapid enrichment of trace amounts of fluorochemicals in aqueous solutions through hydrophobic interactions. Combining solid-phase extraction enrichment with MALDI-TOF-MS allows rapid and highly sensitive quantitative analysis of fluorochemicals in water samples, achieving LODs at parts per trillion (ppt) or sub-ppt levels.^928^ Ouyang et al. recently developed COF-V, spherical continuous organic framework sub-micrometer particles, which show great potential in small molecule analysis by MALDI-MS.^929^ An evaluation of various small molecules such as AAs, nucleobases, peptides, FAs, estrogens, sulfonamide and bisphenols, revealed that COF-V outperforms traditional organic matrices and bulk COF-V in terms of the absence of matrix background interference, high sensitivity, and universal applicability. COF-V also demonstrated high salt resistance and reproducibility, making it suitable for sensitive detection of glucose in diabetic urine.^929^ However, COF submicrometer particles are incompatible with MSI because of their inability to be conveniently sprayed onto the tissue sample, in contrast to traditional organic matrices. To overcome the incompatibility of COF submicrometer particles with MSI, Ouyang et al. developed a MALDI-MS method prepared COF nanomembranes on ITO glass.^930^ This method successfully detected various molecules and exhibited advantages such as an enhanced signal response, minimal background interference, and good reproducibility. Additionally, by utilizing the MALDI-MSI technique based on COF nanomembranes, they visualized the spatial distribution of 5-fluorouracil in mouse liver and evaluated its pharmacokinetics in mouse plasma after different modes of administration. In another study, the COF film was effectively employed for the rapid and accurate quantification of homocysteine in human serum, yielding results that correlated well with clinical enzyme cycling methods.^931^

COFs have diverse structural units and flexible chemical functionalities, providing a wide range of possibilities for their derivatization.^925^ In a study by Hu et al., boronic acid-functionalized COFs (B-COFs) were successfully synthesized using a one-pot strategy, enabling direct detection of *cis*-diol-containing molecules using MALDI-TOF MS (Figure 78a). The introduction of boronic acid groups greatly enhanced the capture selectivity of COFs for *cis*-diol compounds, and the synthesized B-COFs demonstrated excellent enrichment ability and ionization efficiency when tested with model analytes such as quercetin, riboflavin, and catechol.^932^ Recently, Tan et al. synthesized sulfonic acid-functionalized hierarchical porous COFs (H-COF-SO_3_H) as an adsorbent and matrix for the MALDI-TOF-MS detection of paraquat (PQ) and diquat (DQ) in food samples, as shown in Figure 78b.^933^ H-COF-SO_3_H was customized on the basis of the polar cationic nature of quaternary ammonium salt compounds, using a defect structure method and postmodification. Compared with the use of nonfunctionalized microporous COF, the use of H-COF-SO_3_H as an adsorbent and matrix significantly increased the detection sensitivity of PQ and DQ in MALDI-TOF-MS.^933^ Zhang et al. developed a magnetic COFs (Fe_3_O_4_@COFs) as a simultaneous enrichment adsorbent and MALDI matrix to enhance the detection of low-concentration PAHs and their derivatives in PM_2.5_. The Fe_3_O_4_@COFs facilitated strong signals and a clear matrix background in MALDI-TOF-MS analysis for bound PAHs, nitro-PAHs and hydroxy-PAHs in PM_2.5_.^934^

**Figure 78:**
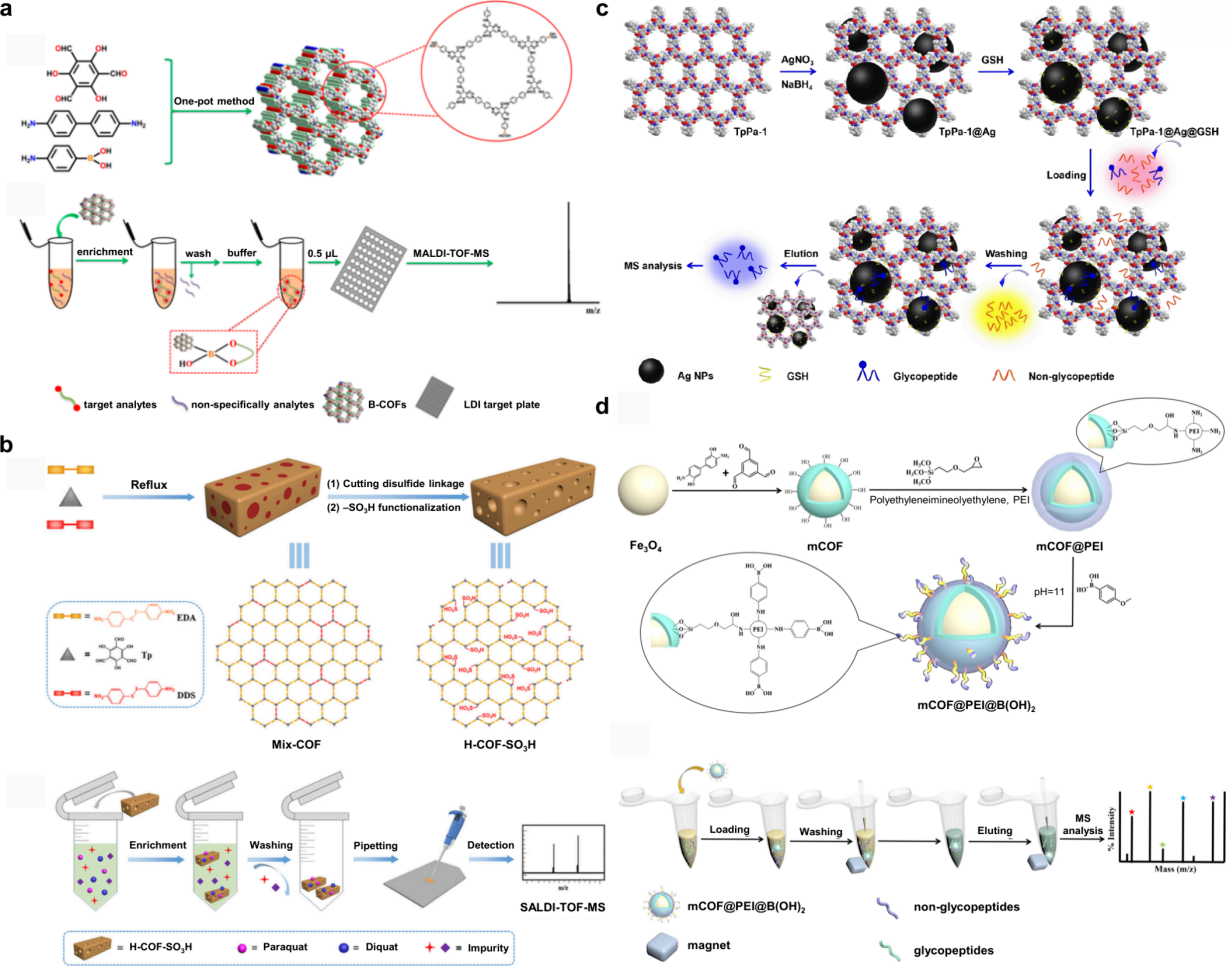
Covalent-organic frameworks as matrices for MALDI-MS. (a) Synthetic route for B-COFs and the selective enrichment of the *cis*-diol-containing compounds using B-COFs. Adapted and reproduced with permission from ref 933. Copyright 2019 American Chemical Society. (b) Flow chart of the preparation of H-COF-SO_3_H and analysis of paraquat (PQ) and diquat (DQ) by MALDI-TOF MS. Adapted and reproduced with permission from ref 937. Copyright 2021 Elsevier Inc. (c) Schematic representation of the preparation process of mCOF@PEI@B­(OH)_2_; enrichment workflow of glycopeptides from digested samples by mCOF@PEI@B­(OH)_2_. Adapted and reproduced with permission from ref 932. Copyright 2021 The Authors under exclusive licence to Springer-Verlag. (d) Procedure for the post-synthetic modification of TpPa-1 and enrichment and detection of the N-linked glycopeptides based on TpPa-1@Ag@GSH. Adapted and reproduced with permission from ref 935. Copyright 2019 Royal Society of Chemistry.

A postsynthetic modification strategy based on preformed frameworks provides a way to enhance the functionality and performance of COFs while minimizing potential damage to their crystallinity. To enhance the interaction between glycopeptides and COFs and improve the selectivity for glycopeptide enrichment, as shown in Figure 78c, Ma et al. developed a postsynthetic modification approach to synthesize a functionalized COF called TpPa-1@Ag@GSH, which was used for efficient enrichment of N-linked glycopeptides.^935^ TpPa-1@Ag@GSH exhibited a fast capture speed, good reusability, and high binding capacity, and demonstrated high sensitivity and selectivity in standard glycoprotein digestion and complex biological samples. Ding et al. prepared a layered imine-based covalent organic polymer with mesopores (p-TpBDH) using a facile solvent-assisted thermal method.^936^ By directly reducing p-TpBDH to p-TpBDH-OH, the hydrophilicity of the polymer was increased, resulting in enhanced glycopeptide capture. p-TpBDH-OH exhibited superior selectivity for glycopeptides, enabling highly selective enrichment of *N*-glycopeptides in biological samples and successful identification of abundant glycopeptides in large-scale *N*-glycoproteomic studies.^936^ Wang et al. used a postsynthetic strategy to synthesize a boric acid-functionalized magnetic COF (mCOF@PEI@B­(OH)_2_) (Figure 78d) with superior properties such as reusability, low detection limits, size exclusion effects, high loading capacity, high recovery rates, and selectivity.^937^ Through MALDI-TOF MS detection, mCOF@PEI@B­(OH)_2_ successfully captured 37 endogenous glycopeptides from human saliva, demonstrating its ability to capture low-abundance glycopeptides.

Furthermore, numerous novel COFs have been developed to enhance the application of MALDI-MS in biological analysis, particularly in terms of offering innovative tools and methodologies for metabolomics and environmental toxicology research. For instance, TPB-BPTP-COF has demonstrated exceptional performance in effectively extracting serum metabolomes and elucidating biomarkers associated with breast cancer.^938^ Additionally, the COF-S@Au nanoparticle composite harnesses the synergistic effects of COFs and AuNPs, resulting in significant signal enhancement and interference resistance for small molecule detection.^939^ Donor–acceptor COFs (D-A COFs) represent a novel class of COFs that integrate donor and acceptor molecules to form ordered heterogeneous structures, which facilitate enhanced charge and energy transfer. Notably, the D-A COF nanofilm has been developed as a matrix for MALDI-MS, exhibiting high sensitivity and low background interference, thereby significantly improving the mass spectral response of small molecules.^940^ This advancement has enabled accurate quantification of creatinine in human serum samples for clinical diagnosis. Additionally, a new type of COF-DhaTab film was successfully synthesized through multiple screening and imprinting processes, whereupon it showed feasibility for small molecule detection and the successful visualization of the spatial distribution of perfluorooctane sulfonate in zebrafish, as well as in rat kidney and liver tissues.^941^

In summary, COFs are highly ordered porous polymers with well-defined structures, offering excellent chemical stability, high surface area, and customizable properties. COFs have attracted attention as matrices for MALDI-MS because of their ability to enhance the detection of small molecules.^841^ Recent research efforts have been dedicated to developing new COFs with enhanced performance for MALDI-MS applications. Emphasis is placed on improving the enrichment efficiency and selectivity of COFs for analytes, achieved through adjustments in synthesis methods, COF structure, and pore size to optimize molecular diffusion. Additionally, the incorporation of specific functional groups into COFs can enable the selective adsorption of target compounds, leading to enhanced performance in MALDI-MS.^933^ Ongoing research and development in COFs and their functionalization strategies are crucial for expanding the range of analyte species and selecting suitable COFs for MALDI-MS applications.

##### 3.2.4.3 Hydrogen-Bonded Organic Frameworks

Another class of porous frameworks, called hydrogen-bonded organic frameworks (HOFs), are ordered 2D or 3D frameworks formed by the self-assembly of discrete organic components through weak hydrogen bonding interactions. They possess unique characteristics such as solution processability, ease of purification, and the ability to heal through recrystallization, distinguishing them from zeolites, MOFs, and COFs.^942^ Initial research on the construction of HOFs started in the early 1990s,^943^ but it was not until 2010 that HOFs with permanent porosity through gas adsorption isotherms were first reported.^944^ The aromatic domains and p-electron systems of HOFs give them a high molar absorption coefficient in the UV–vis region, enabling efficient energy transfer with analytes and making them suitable matrices for MALDI-MS analysis. Yin et al. developed a novel MOF@HOF composite material through a solvent-thermal reaction, which enabled highly sensitive detection and quantitative analysis of flavonoids in kumquats and tangerines using MALDI-MS.^945^ Furthermore, MALDI-MS imaging studies using the MOF@HOF matrix revealed the tissue-specific distribution of flavonoids in the fruit peels. Although research using HOFs for MALDI-MS is limited, these findings demonstrate their potential and call for further development and exploration in this field.

##### 3.2.4.4 Summary

In summary, MOFs and COFs are considered the two most representative types of porous crystalline frameworks, and they exhibit significant differences and complementarity.^905^ MOFs are known for their excellent crystallinity, ultra-high surface area, and stability under aqueous or acidic conditions. In contrast, COFs possess higher chemical and environmental stability but lower crystallinity and surface area.^905^ HOFs, on the other hand, are formed through weaker hydrogen bonding interactions, making them more delicate and less stable. However, HOFs offer advantages such as easier synthesis, characterization, handling, and recyclability, making them particularly advantageous for practical applications.^946^

MOFs used as matrices in MALDI-MS demonstrate high ionization efficiency, salt tolerance, and reproducibility, because of their unique porous structure, which enables the selective enrichment of analytes such as PAHs, carbohydrates, and peptides.^688^ The thousands of reported MOF structures in the Cambridge Structural Database, constitute a rich resource for selecting optimal materials for MALDI-MS applications.^688^ As a result, the use of MOFs as matrices in MALDI-MS is still at an early stage, with vast potential for further exploration and development. Compared with MOFs, COFs exhibit diverse π–π conjugated structures and functional groups, endowing them with stronger enrichment capabilities and higher water stability.^928^ They can interact with contaminants in solution through various modes, such as coordination, electrostatic interactions, hydrophobic interactions, π–π stacking, and hydrogen bonding.^947–949^ Therefore, COFs have potential as efficient solid-phase extraction adsorbents and alternative matrices for the rapid and sensitive detection of organic pollutants using MALDI-TOF-MS analysis. Despite the excellent performance of MOFs, COFs, and HOFs in supporting MALDI small molecule detection, challenges such as poor stability of MOFs and stringent synthesis conditions for COFs still exist.^691^ Further efforts are needed to design and synthesize MOF-, COF-, or HOF-based materials through de novo synthesis and postsynthetic structural processing.

#### 3.2.5 Quantum Dots

Quantum dots (QDs) are nanoscale semiconductor crystals whose physical size is smaller than the exciton Bohr radius (<10 nm).^950^ Owing to quantum confinement effects, QDs exhibit unique and fascinating optical properties, such as sharp and symmetric emission spectra, high quantum yields, and size-dependent tunable emission wavelengths.^951^ In recent years, QDs have demonstrated exceptional capabilities in various fields such as photocatalysis, sensing, immunoassays, DNA hybridization, and cellular imaging, and have garnered widespread attention.^952–954^ Owing to the unique structure and electronic properties of QDs, they are ideal for determining the D/I of analytes by MALDI-MS.^955,956^ Currently, typical QDs used in MALDI-MS include cadmium selinide (CdSe), cadmium telluride (CdTe), cadmium sulfide (CdS), and zinc selenium (ZnSe), among others, which have demonstrated exceptional performance in areas such as peptidomics, proteomics, and glycomics.

Surface modification of QDs enhances their specific adsorption interactions with peptides, carbohydrates, or oligosaccharides, leading to improved D/I efficiency of samples. Shrivas et al. developed a rapid analysis method for compounds such as AAs, peptides, and proteins using water-soluble CdSe QDs functionalized with MUA as matrices and preconcentration probes.^951^ The thiol carboxylic acid groups on CdSe QDs strongly bind to biomolecules, resulting in improved sensitivity with a peptide limit of quantification of 100 pM and a relative standard deviation (RSD) <10%. Moreover, in a microwave enzymatic digestion analysis, CdSe QDs can absorb radiation from microwave and capture lysozyme peptides. In another study by Shrivas et al., functionalized ZnSe QDs capped with cysteine (ZnSe-Cys QDs) were used as affinity probes to enhance protein ion signals and as accelerators for microwave-assisted enzymatic digestion in direct MALDI-TOF MS analysis.^957^ Wu and Chung utilized 3-mercaptopropionic acid (3-MPA) modified ZnSe QDs (ZnSe-3MPA QDs) as matrices to directly analyze peptides and proteins in sodium salt solutions using MALDI-MS.^958^ The method achieved higher sensitivity through electrostatic interactions, with positively charged biomolecules being adsorbed onto the negatively charged ZnSe-3MPA QD surface. Furthermore, the absence of an efficient proton source in these nanomaterials serves to improve the ionization efficiency of sodium adduct ions ([M+Na]^+^).^958^ Furthermore, Bibi and Ju utilized four types of ligand-functionalized quantum dots as matrices for the qualitative and quantitative analysis of small neutral carbohydrates, oligosaccharides, and cyclic oligosaccharides using MALDI-MS.^959^ A schematic representation of quantum dots utilized as a matrix for carbohydrate QDA-LDI-MS analysis is presented in Figure 79a. The QDs included *meso*-2,3-dimercaptosuccinic acid (DMSA) modified CdTe QDs (DMSA-CdTe QDs), 3,3′-dithiodipropionic acid di­(*N*-hydroxysuccinimide ester) (DSP) modified CdTe QDs (DSP-CdTe QDs), mercaptopropionic acid (MPA) modified CdS QDs (MPA-CdS QDs), and thioglycolic acid (TGA) modified CdS QDs (TGA-CdS QDs). The functionalized QDs selectively ionized carbohydrates, achieving higher signal intensity than traditional organic matrices did in MALDI-MS, with DSP-CdTe QDs particularly excelling in both qualitative and quantitative analyses.

**Figure 79:**
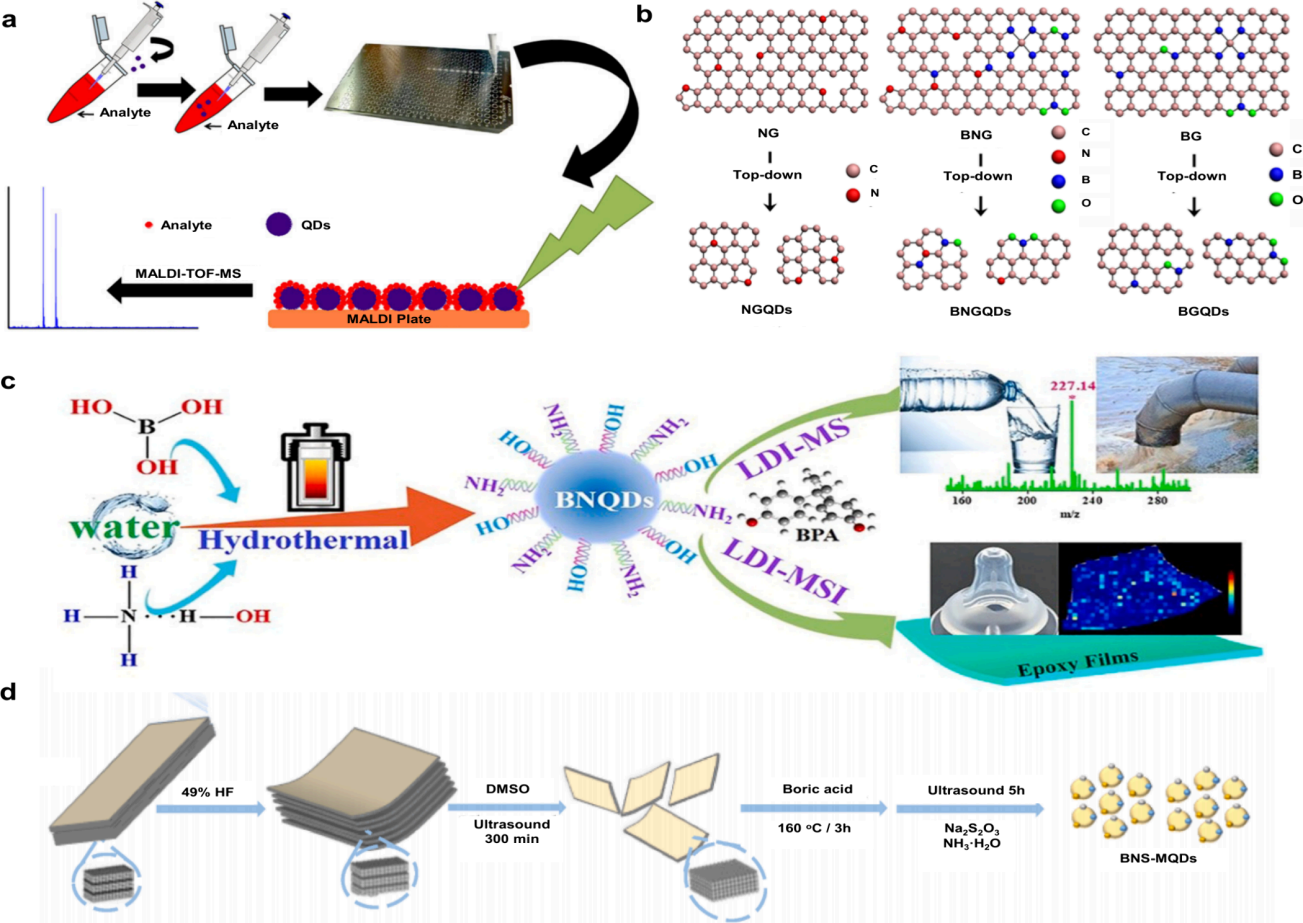
Quantum dots as matrices for MALDI-MS. (a) Schematic illustration of QDs as a matrix for the QDA-LDI-MS analysis of carbohydrates. Adapted and reproduced with permission from ref 959. Copyright 2016 John Wiley & Sons. (b) Schematic illustrations of the top-down strategy for the preparation of NGQDs, BNGQDs, and BGQDs from diverse HGs. Adapted and reproduced with permission from ref 961. Copyright 2022 American Chemical Society. (c) Synthesis of BNQDs and their application as an inorganic matrix for BPA analysis by MALDI-MS. Adapted and reproduced with permission from ref 962. Copyright 2023 Elsevier BV. (d) Synthesis pathway of BNS-MQDs. Adapted and reproduced with permission from ref 963. Copyright 2025 Elsevier BV.

In addition, Seino et al. created a novel ionization platform for MALDI-MS analysis using self-assembled germanium nanodots (GeNDs) of uniform size grown on a silicon wafer.^960^ GeNDs are nanocrystalline dots (*i.e.*, QDs) made by molecular beam epitaxy and form stable nanodots on the silicon single crystal surface because of lattice mismatch. They investigated the performance of MALDI-MS using GeNDs by measuring a wide range of analytes, including peptides, proteins, synthetic oligomers, and polymer additives.^960^ Jin et al. developed a “top-down” strategy to create heteroatom-doped graphene QDs (HGQDs) for small molecule MALDI-MS detection and imaging (Figure 79b). These HGQDs leverage the π-conjugated network of their parent material, enhancing energy transfer and negative ion generation, while their small size promotes uniformity and reproducibility in MALDI-MSI.^961^ Compared with other QDs, HGQDs exhibit superior laser D/I capabilities for a range of small molecules, such as AAs, FAs, saccharides, small peptides, nucleobases, anticancer drugs, and bisphenol pollutants. Zhao et al. investigated the application of boron nitride QDs (BNQDs) as an inorganic matrix in MALDI MS and MSI for the detection of bisphenol A and demonstrated their effectiveness in analyzing environmental water, plastic, and pacifier samples.^962^ A schematic diagram of the synthesis of BNQDs and their application as an inorganic matrix for the MALDI-MS analysis of bisphenol A is shown in Figure 79c. Compared with traditional organic matrices, the abundant hydroxyl and amino groups on BNQDs enhance hydrogen bonding with bisphenol A, improving its ionization and selectivity, while also offering superior sensitivity, lower background interference, excellent salt tolerance, and high reproducibility, thus making them ideal for analyzing small molecular pollutants. Additionally, Peng et al. successfully synthesized a novel ternary-doped MXene QDs (BNS-MQDs) incorporating boron, nitrogen, and sulfur, which were integrated into a photocurable polymer resin to produce a cost-effective and efficient 3D printed matrix for the MALDI target plate (3D-MTP) for direct multidimensional MALDI-TOF MSI analysis of small molecular environmental pollutants (Figure 79d).^963^ This target plate was successfully used to investigate the distribution of the emerging antioxidant pollutant *N*,*N*′-di-2-naphthyl-*p*-phenylenediamine in female zebrafish, revealing its accumulation in organs such as the eyes, gills, and liver for the first time.

QDs are valuable matrices for MALDI-TOF-MS because of their unique structure and electronic properties, but most current studies focus on water-soluble NMs.^957^ Some surface-modified QDs are hydrophobic and insoluble in water, making their interactions with biomolecules reliant on surface modifications. High-quality water-soluble QDs are needed, and the toxicity of certain QDs, particularly those containing cadmium, is important to consider.

#### 3.2.6 Outlook

The rapid advances in nanotechnology have led to the increasing use of inorganic NPs and nanostructured surfaces to complement traditional organic matrices in MALDI-MS analysis. This review section summarizes the applications of various NMs, including metal-based NMs, carbon-based NMs, silicon-based NMs, organic frameworks, and QDs.

While traditional organic matrices in MALDI-MS provide good sensitivity and resolution, they often lead to interference from fragment ions, molecular ions, and matrix adducts, particularly for small molecules less than 700 Da. The high abundance of matrix-related ions can create spatial charge effects and saturate the detector, reducing analyte signal sensitivity. Consequently, the development of high-sensitivity inorganic MALDI matrices is crucial for minimizing these interference peaks. In comparison with traditional MALDI techniques utilizing organic matrices (such as CHCA, DHB, and SA), MALDI-MS with inorganic matrices, offers distinct advantages for the analysis of small molecules. First, inorganic matrices generally yield background-free mass spectra in the LMW range (<700 Da), enhancing the clarity of the results. Second, MALDI-MS using inorganic matrices demonstrates superior single-particle reproducibility, as it circumvents the issues of uneven mixing and co-crystallization commonly associated with organic matrices. Third, NMs function not only as energy transfer matrices for analytes but also as adsorbents that effectively clean and enrich analytes in complex samples, thereby improving detection limits in MALDI-MS analysis. Fourth, compared with its traditional counterparts, MALDI-MS with inorganic matrices frequently results in higher signal intensity, as the inorganic matrix mitigates competition between analytes and matrix ions.

Moreover, the performance of inorganic matrices in MALDI-MS is largely influenced by the inherent properties of the NMs, including size, morphology, composition, concentration, and surface functionalization. While remarkable progress has been made in addressing the challenges faced by traditional MALDI-MS through the development of new NMs as matrices, several challenges still need to be addressed for practical applications.

I. The design and synthesis of inorganic NMs for matrix applications is often complex and expensive, necessitating the development of simpler, more versatile, and cost-effective methods to enable large-scale production for clinical applications.
II. To address the complexity and heterogeneity of biological samples, it is essential to develop multifunctional NMs and hybrid matrices with increased sensitivity, selectivity, and speed of analysis.
III. Although MALDI-MS using inorganic matrices minimizes the “sweet spot” effect seen with organic matrices, it still falls short in accurate quantification compared with traditional tandem MS. To enhance data authenticity, reproducibility, and quantitative accuracy, it is crucial to develop optimized substrates and matrix deposition methods, and to integrate and automate these processes with emerging technologies such as microarrays.
IV. Designing multifunctional matrices for multidimensional detection can facilitate the integration of proteomics, peptidomics, and metabolomics, thereby enriching our understanding of molecular interactions and biological processes. Further development is needed to advance clinical applications of high-throughput multi-omics studies based on MALDI-MS, including matrix optimization, analysis workflow design, and data mining.
V. Current research on inorganic matrices for MALDI-MS mainly emphasizes the development of new matrices, while the understanding of D/I processes remains limited, leading to inefficient and random analyte selection. Investigating these mechanisms in novel materials is crucial for guiding effective material design and enhancing practical applications.

Overall, NMs usable as MALDI matrices need to meet a series of basic requirements, including high adsorption efficiency in the UV range (220–350 nm), chemical stability, high energy transfer efficiency, and low background signals in the low mass region. Compared with traditional organic matrix MALDI-MS techniques, MALDI-MS techniques based on inorganic matrices have advantages such as clear background, fewer interference peaks, high salt tolerance, and good detection reproducibility. Therefore, they have strong potential applications in fields such as forensic science, pharmaceutical science, environmental monitoring, biomedical analysis, and quantitative analysis. However, there is currently a lack of systematic research and consensus theory on how to select suitable NMs as MALDI matrices. Furthermore, substantial efforts are needed to facilitate the transition of new MALDI-MS technology into clinical and industrial settings, necessitating the continued investment of resources and expertise.

### 3.3 Organic–Inorganic Binary and Hybrid Matrices

Conventional organic matrices are typically mixed with the samples of interest to facilitate the absorption of the incident UV laser beam and mediate the desorption and ionization of the analyte,^23^ thereby generating ions in the gas phase. However, the presence of background signals produced by these matrices can negatively impact the performance of MALDI-MS, particularly in the analysis of small molecules under 500 Da, thus hindering the effective and sensitive detection of LMW compounds.^964^ The use of inorganic matrices (various different NMs) that are not ionized under UV radiation offers a potential solution to this limitation. However, inorganic matrices also pose significant challenges. The lack of proton donors in inorganic matrices results in the dominance of alkali adduct ions in MALDI-MS spectra, which complicates the identification of target analytes in complex samples.^965^ Furthermore, while the exact mechanisms by which matrices influence D/I remain incompletely understood, it is evident that different matrices selectively ionize distinct classes of compounds.^966^ For instance, THAP is particularly effective for the analysis of oligosaccharides, whereas CHCA is frequently employed for peptides and small proteins. In addition to conventional organic matrices, various NMs have emerged as promising alternatives; however, they also demonstrate selectivity in their ionization properties.^966^ To overcome these limitations, recent studies have begun to combine inorganic and organic matrices, aiming to achieve more comprehensive analyte coverage and enhance detection efficiency. Figure 2 and Table S7 present organic–inorganic binary and hybrid matrices, highlighting their preferred ionization mode and applications for MALDI-MS/MSI

#### 3.3.1 Organic–Inorganic Binary Matrices

Organic–inorganic binary mixed matrices facilitate uniform sample-matrix co-crystallization by suppressing the formation of matrix clusters and fragments, thereby broadening the detection range, increasing sensitivity, and improving the overall quality of the D/I process. Notably, Feenstra et al. introduced a simple binary matrix mixture comprising nonfunctionalized Fe_3_O_4_ NPs and the organic matrix DHB (Fe_3_O_4_/DHB) to effectively mitigate TAG ion suppression by PCs.^966^ This matrix not only reduced TAG ion suppression but also facilitated the desorption and ionization of various lipids, including cationic PC, anionic PE and PI, and neutral digalactosyldiacylglycerol (DGDG), while demonstrating remarkable efficiency for detecting large polysaccharides that were undetectable with individual matrices.^966^ Chen et al. developed a binary matrix composed of CDs and 9-AA (CDs/9-AA), which demonstrated high sensitivity and reproducibility for the analysis of small molecules, including nucleosides, AAs, oligosaccharides, peptides, and anticancer drugs in positive ion mode.^967^ As depicted in Figure 80a, a proposed mechanism by which 9-AA/CDs increase the sensitivity of positive ion MALDI-TOF MS for small molecule detection is illustrated. The improved analytical performance of 9-AA/CDs is hypothesized to arise from their π-conjugated network, the abundant carboxyl and hydroxyl groups on the surface of the CDs, the formation of thin matrix crystals, and the potential ionization suppression effect of CDs on 9-AA. Moreover, Hou et al. employed a MgO/NEDC matrix for the qualitative and quantitative analysis of high-concentrations of glucose and Pb^2+^ in complex metal solutions, highlighting the matrix's significant resistance to interference.^968^ Wang et al. developed a simple MALDI-TOF-MS method to enhance the uniformity of sample crystals and facilitate the ionization of three sweeteners (aspartame, neotame, and advantame), using a binary matrix composed of NDs and DHB.^969^ Through the optimization of ND concentrations, sample deposition methods, and ionization dopants, effective ionization conditions were established, and the ND/DHB binary matrix demonstrated reliable detection of sweeteners in commercial beverages.

**Figure 80:**
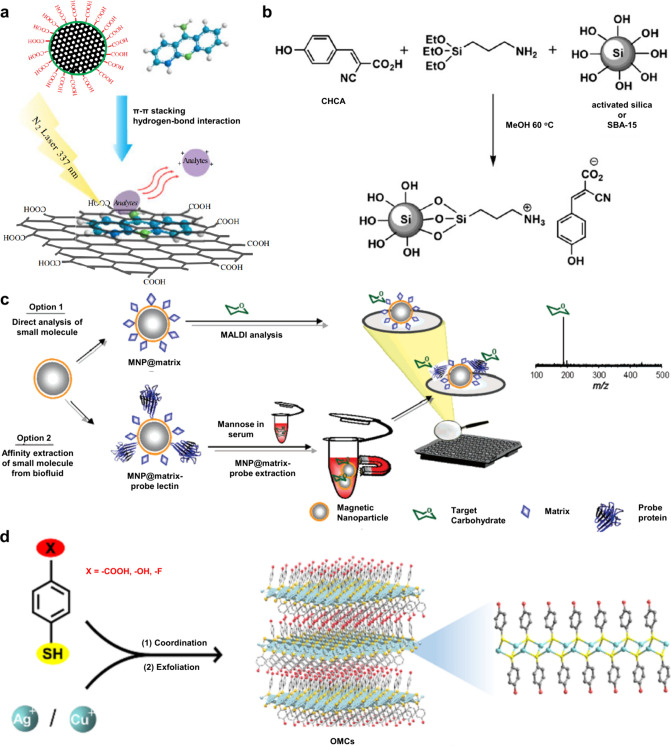
Organic–inorganic binary matrices for MALDI-MS. (a) Schematic of the mechanism by which 9AA/CDs, as a MALDI matrix, improve the detection sensitivity for small molecules in positive ion mode MALDI-TOF MS. Adapted and reproduced with permission from ref 967. Copyright 2016 American Society for Mass Spectrometry. (b) Synthetic route to ionic macrocomplexes of [silica gel-Si-NH_3_^+^]­[CHC^–^] and [SBA-15-Si-NH_3_^+^]­[CHC^–^]. Adapted and reproduced with permission from ref 971. Copyright 2011 Elsevier BV. (c) Workflow of functionalized MNP-assisted MALDI-TOF MS. Option 1: NP@matrix serves as a matrix-free additive. Option 2: Bifunctional MNP@matrix-protein serves as an affinity matrix-free additive. Adapted and reproduced with permission from ref 972. Copyright 2007 American Chemical Society. (d) Scheme of preparation for few-layered OMCs (organic metal chalcogenides). Adapted and reproduced with permission from ref 973. Copyright 2024 The Authors under a Creative Commons Attribution 3.0 Unported Licence.

Additionally, Dufresne et al. introduced a method for constructing MALDI substrates by pre-coating CHCA and potassium salt layers onto NAPA, which significantly improved the detection capabilities for lipid signals.^970^ Zhao et al. mixed Fe_3_O_4_ magnetic NPs (Fe_3_O_4_ MNPs) with humic acids (HAs) to create magnetic HAs (MHAs), allowing rapid and convenient separation from organic solvents. This mixed matrix demonstrated potential applications in the separation and enrichment of illegal additives in food, as the MHAs, after adsorbing the analytes, can be directly applied to the MALDI plate for swift analysis, facilitating a fast and straightforward operation while minimizing analyte loss.^387^

#### 3.3.2 Organic–Inorganic Hybrid Matrices

Recent studies have focused not only on simply mixing matrices, but also on combining the properties of organic and inorganic matrices to create hybrid matrices for MALDI-MS analysis.^964^ These hybrid matrices possess unique characteristics, with the organic components covalently anchored to an inorganic inert framework. For instance, Tang et al. reported a novel matrix that combines CHCA with Santa Barbara amorphous-15 (SBA-15) using 3-aminopropyltriethoxysilane (APTES) (SBA-15@APTES@CHCA).^974^ This matrix exhibits enhanced energy absorption and sample crystallization capabilities, and it is more stable than other SBA-15 modified ionic macrocomplex matrices, making it suitable for the analysis of small molecules such as pesticides and quinolone antibiotics. Additionally, Sun et al. synthesized TiO_2_ composite particles (TiO_2_ CPs) containing different anchoring groups based on dihydroxybenzoic acid (DHB) analogs to improve D/I performance.^975^ Among these, 3,4-DHB-TiO_2_ CPs exhibited the highest UV absorption capacity and superior detection sensitivity while maintaining a low background signal. These composite particles can serve as a new matrix for detecting and imaging various secondary metabolites in different aged roots of *Scutellaria baicalensis* Georgi (baical skullcap). Zhang et al. modified multilayer Ti_3_C_2_T_X_ (MXene) using *p*-aminoazobenzene (*p*-AAB) to create a novel material, *p*-AAB/MXene, as a matrix for MALDI-TOF MS to facilitate the rapid analysis of small-molecule emerging pollutants.^976^ The innovative *p*-AAB/MXene material functions as both an adsorbent and a matrix for direct mass spectrometric detection, enabling high sensitivity and precision in analyzing *p*-phenylenediamine-quinones and diamide insecticides in food (beverage) and environmental (PM2.5) samples, while effectively enhancing laser energy absorption and ionization of target compounds for trace pollutant monitoring. Chitanda et al. introduced the application of functionalized NDs as MALDI-MS matrices for LMW pharmaceuticals/metabolites. They covalently attached conventional matrices (or a lysine moiety) onto the surface of detonated NDs, designing novel matrices covalently linked to detonation NDs, *i.e.*, ND-COOH, ND-CHCA, ND-DHB, and ND-SA.^977^ The results showed that these novel matrices achieved ionization of various LMW drugs/metabolites in negative ion mode, generating strong ion signals and increasing S/N ratio. Additional studies have explored CHCA-modified SiO_2_ hybrid materials (CHCA-SiO_2_),^964^ CHCA-functionalized AuNPs,^978^ THAP–zeolite complexes,^979^ and Cu_2_O PS combined with DHB (Cu_2_O PS@DHB).^980^ These materials have shown excellent performance in MALDI-MS analysis, and are applicable for the detection and imaging of small molecular compounds and biomacromolecules.

Liu et al. conducted MALDI-TOF-MS analysis on small molecules using a mixed ILM material ([TiO_2_-Si-NH_3_^+^]­[CHC^–^]), which significantly improved the S/N ratio and reduced interference from matrix background ions.^981^ Mullens et al. synthesized the matrix by covalently linking an ionic liquid precursor (a silylated amine) to silica, followed by reacting it with the anion of the conventional MALDI matrix, CHCA, to form an ionic macrocomplex [SBA-15-Si-NH_3_^+^]­[CHC^–^] (Figure 80b).^971^ Their research demonstrated that the modified mesoporous silica-based matrix exhibits excellent signal intensity and minimal chemical noise in the analysis of DA and serotonin, which can be attributed to its increased surface area and enhanced uniformity. Su et al. synthesized a modified SBA-15 matrix ([CHC^–^]­[NH_3_^+^-Si-SBA-15-Si-NH_3_^+^]­[CHC^–^]), using CHCA and modified mesoporous silica (SBA-15-Si-NH_3_^+^), which exhibited minimal background interference, high homogeneity, and heightened sensitivity and reproducibility for melamine analysis. This novel matrix enabled the development of a high-throughput screening method for detecting melamine in milk samples using MALDI-TOF MS.^982^

Additionally, organic matrix functionalized Fe_3_O_4_ MNPs (matrix@MNP) represent an important category within organic–inorganic hybrid materials. For example, Lin et al. coupled the organic matrices CHCA and DHB to Fe_3_O_4_ MNPs (CHCA@MNPs and DHB@MNPs), which enhanced the signal by enriching the samples within the matrix through magnetic interactions.^972^ Figure 80c illustrates the workflow of MALDI-TOF-MS assisted by functionalized MNPs. This strategy created a targeted biomolecular probe capable of rapidly enriching and detecting low-abundance small molecules in human serum. Furthermore, Tseng et al. explored the broad applicability of DHB@MNP as an ionization matrix, demonstrating their utility for the rapid screening and identification of various types of small molecules, characterized by prominent, background-free, and easily identifiable protonated ions.^983^ Ho et al. utilized DHB@MNPs as a new matrix, combining liquid–liquid extraction and seed-layer surface preparation with MALDI-MS technology to successfully identify and quantify small molecules in urine.^984^ Obena et al. prepared three types of matrix@MNPs (CHCA@MNP, DHB@MNP, and SA@MNP) for the detection of metal ions, such as Cu in tap water.^985^ The results indicated that all three types of matrix@MNPs, particularly DHB@MNP, significantly enhanced the detection sensitivity of metal ions and provided clear identification through characteristic isotopic patterns and precise mass measurements, suggesting a mechanism for the NP-mediated MALDI metal ionization process.

Organic metal chalcogenides (OMCs) are a novel class of 2D organic–inorganic hybrid materials, featuring metal chalcogenide layers covalently bonded to organic functional groups.^986^ The high designability of inorganic layers and organic functional elements allows OMCs to exhibit tunable optical absorption, carrier types, and mobility, enhancing their photothermal conversion and ionic transfer efficiency.^987^ Moreover, OMCs demonstrate improved dispersion in various solvents, leading to uniform substrate solutions that enhance the sensitivity and reproducibility of MALDI-MS. Ouyang et al. synthesized four highly designed low-layer OMCs (Figure 80d) and investigated their potential as promising MALDI-MS matrices, revealing significant improvements in UV absorption, energy transfer, and ionization efficiency.^973^ Notably, the optimized OMC Cu­(SPh-COOH) showed an enhanced signal response and a clean background, successfully detecting various metabolites within the molecular weight range of 180–828 Da and demonstrated high precision in diagnosing conditions like central precocious puberty (one of the most common endocrine disorders in children) based on serum metabolic fingerprints.^973^

#### 3.3.3 Outlooks

The use of organic matrices in MALDI-MS can result in uneven co-crystallization and interference from low *m*/*z* peaks, whereas inorganic matrices may suffer from inadequate protonation due to a lack of proton donors. To address these challenges, researchers have combined the properties of organic and inorganic matrices to design various organic–inorganic binary matrices through either direct mixing or hybrid/synthetic approaches, to achieve background-free and high-performance MS measurements, complementing other MS methods for small molecule analysis. For example, Chen et al. identified four key factors affecting the ionization of mixed binary matrices like 9-AA/CDs:^967^ (1) the strong UV absorption of the π-conjugated network of 9-AA/CDs, (2) carboxyl groups modified on the surface of the CDs acting as protonation sites, (3) thin crystal layers of 9-AA/CDs quickly reach high surface temperatures, reducing energy transfer during MALDI-MS, and (4) the inhibitory effect of CDs as dopants on the ionization of 9-AA. Obena et al. proposed a matrix@MNP-mediated ionization mechanism by which metal cations are chelated by salicylate or carboxylate groups on DHB, leading to the preconcentration of analytes. Upon UV irradiation, the excited DHB heats the matrix, promoting energy transfer from the MNPs to ionize and release the metal ions into the gas phase.^985^ Currently, research on the mechanisms of organic–inorganic binary/hybrid matrices is limited, and further investigation into their ionization processes could significantly improve MALDI-MS performance and advance the field.

## 4 MALDI Matrices for Multi-Omics Analysis

For the purposes of this review, targeted analysis is defined as the investigation of specific types of molecules, whereas untargeted analysis refers to the comprehensive analysis of a multitude of component types within a sample, without being restricted to a single molecular category.

### 4.1 MALDI Matrices for Proteomic Analysis

The proteome represents the entirety of the proteins encoded by the genome at a specific developmental or cellular stage^988^ and is a complex microcosm of the network of structural and regulatory proteins at a given moment in time.^989^ Proteomics is the scientific field of proteome research, including protein-related studies such as protein identification, quantification, structural analysis, functional studies, and their interactions. Especially in the context of “multi-omics,” the focus of proteomics research has shifted from merely establishing a protein list in a specific tissue to exploring the protein spatial heterogeneity and co-localization in biological samples to explain their potential function in normal or diseased cells and tissues.^990^ Among the available strategies, MS-based proteomics has become indispensable in molecular and cellular biology as well as in the emerging field of systems biology. Although ESI and MALDI are the most commonly used ionization techniques in proteomics, tandem MS based on the MALDI source has the advantages of simplicity and rapidity for the qualitative and quantitative analysis of AAs, peptides, proteins, protein–protein interactions, and post-translational modifications.^991^ For instance, sodium dodecyl sulfate polyacrylamide gel electrophoresis (SDS-PAGE), as the cornerstone of protein separation, provides purified samples for MALDI-TOF MS based on molecular weight differences. The extraction of target bands from the gel, followed by enzymatic digestion, leverages MALDI-TOF MS's high sensitivity and accuracy to compensate for the limitations of SDS-PAGE in terms of identity confirmation. This process ultimately yields the protein's molecular fingerprint.^992^ Collectively, these elements constitute a closed-loop separation-identification system, thereby facilitating advancements in both qualitative and quantitative analysis within the domain of proteomics research. Over the past decades, the successful use of MALDI-MS/MS for proteomics analysis has enabled researchers and clinicians to decipher the molecular functions, biological processes, cellular compositions, and associated pathways of numerous proteins, as well as their roles in the pathogenesis of diseases.

AAs are indispensable constituents of protein synthesis and essential substrates in cellular energy metabolism, which are ubiquitous in all living organisms and participate in a multitude of critical biochemical processes. To date, a total of 22 protein AAs have been identified in living organisms, of which 20 are common AAs and the remaining two are classified as specialized AAs.^359^ These AAs are important for nutritional intake, health maintenance, and disease occurrence in living organisms. Peptides are compounds formed by the linkage of α-AAs via peptide bonds, as well as intermediate products of protein hydrolysis. In comparison to peptides, proteins possess longer AA sequences and more sophisticated spatial structures. They are involved in nearly all biological processes, acting as the primary agents in many life activities. Concurrently, proteins constitute a significant proportion of cellular mass, representing approximately half of the total dry mass, three-quarters of the inner mitochondrial membrane, one-quarter of the myelin membrane, and one-half of the plasma membrane.^993^ In summary, AAs, peptides, and proteins are indispensable functional molecules in living organisms, and they together constitute an integral component of proteomics, which is becoming increasingly significant within the field of life sciences.

Despite the increasing application of MALDI-MS to the detection and *in situ* analysis of AAs, peptides, and proteins, the inherent complexity and diversity of these compounds present a significant challenge to achieving comprehensive and effective characterization. On the one hand, it is challenging to identify a universal MALDI matrix that can effectively ionize these three molecules simultaneously, given their significant differences in molecular weight and structure; on the other hand, these molecules require distinct consideration in MALDI-MS analysis, including the choice of ionization mode and mass resolution. It is therefore crucial to ascertain the most suitable analytical technique for the particular object under investigation. The selection of suitable matrices is highly important for enhancing MALDI-based proteomics research, as it allows for the effective ionization of specific analytes.

#### 4.1.1 MALDI Matrices for Untargeted Proteomics Analysis

With continuous screening and development by researchers, a variety of MALDI matrices have been used for the analysis of AAs, peptides, and proteins, most of which are nontargeting matrices that enable the detection of different types of molecules (Table S8). Although DHB and CHCA are effective conventional organic matrices for AA detection, they are susceptible to ionic interference in the low mass region, which presents a challenge in the detection of all protein AAs. CHCA and SA are established matrices for peptide and protein analysis, however, their relatively low ionization efficiency has limited their application to a range of tissue samples, particularly for the detection of HMW proteins. Consequently, new matrices with reduced matrix-associated ion interference and enhanced ionization efficiency coupled with a more expansive detection range have been continually explored and proposed.

DMAN has been reported as a novel matrix for MALDI-TOF-MS analysis of anions, demonstrating the ability to distinguish deprotonated analyte signals with clarity.^52^ The effective measurement of several strong and weakly acidic LMW analytes, including AAs, fatty acid–AA conjugates, and short peptides, at physiologically relevant concentrations has been shown to be a viable application of DMAN. Moreover, DMAN displayed excellent linearity for analytes, even at low picomolar/femtomolar LODs, particularly for peptides over three orders of magnitude. A study by Gu et al. revealed that HNBN was a highly versatile organic matrix for peptide and protein analysis.^56^ It exhibited strong UV-absorption properties and provided a clean spectral background in the low mass range. Moreover, compared with CHCA, HNBN demonstrated superior performance in peptide detection and comparable protein coverage in *E. coli* analysis. In addition, HMPPA and IPA were recognized as effective matrices for peptide and protein analysis.^450^ Among these, IPA was more effective for relatively small proteins (less than 20 kDa), whereas HMPPA exhibited superior detection sensitivity and tolerability. However, neither HMPPA nor IPA offered a substantial advantage over DHB or CHCA in protein analysis, although either could be employed as an alternative matrix in instances where DHB or CHCA was ineffective. Tang et al. first proposed the use of HZN as a matrix for the MALDI-MSI analysis of endogenous proteins.^443^ Compared with conventional matrices such as DHB, CHCA, and 9-AA, the HZN matrix exhibited higher sensitivity, improved signal intensities, and broader molecular coverage (50–200,000 Da). The utilization of HZN has been shown to facilitate the visualization of tissue-specific distributions and alterations in protein expression within the kidney and liver sections of obese *ob*/*ob* and diabetic *db*/*db* mice, respectively. Through the innovative addition of BP particles to standard MALDI matrices such as SA and CHCA, Mandal and coworkers effectively enhanced the intensity of MS detection of specific AAs and peptides.^687^ The incorporation of supplementary BP prompted the formation of uniform co-crystallized particles with the matrix on the MALDI target plate. This decreased the discrepancies in matrix/analyte deposition and increased the intensity and reproducibility of the ion signals. Furthermore, AAs containing aromatic rings (*e.g.*, phenylalanine (Phe) and tryptophan (Trp)) preferentially interact with the surface of BP particles, whereas AAs without aromatic rings (*e.g.*, glycine (Gly) and leucine (Leu)) do not. This observation led to the hypothesis that BP may function by interacting with aromatic groups within the molecule. In addition to the single matrix previously discussed, binary or combined matrices have been employed with the objective of enhancing analyte ionization efficiency and obtaining superior mass spectral signals for a diverse range of molecules. The combination of two commonly used matrices, DHB and CHCA, has led to a significant improvement in the point-to-point reproducibility and sequence coverage of peptide mass mapping.^636^ This hybrid matrix displayed increased tolerance to salts and impurities, obviating the need for sample prepurification, and facilitating the analysis of intact proteins, particularly glycoproteins.

As MALDI matrices have evolved, a new class of ILMs has gradually been used for MS detection of AAs, peptides, and proteins.^580^ Mank and coworkers evaluated a novel ILM, DHBB, and compared it with previously available ILMs such as CHCAB and SinTri.^585^ Their findings indicated that DHBB was efficacious for the detection of peptides and proteins, whereas CHCAB was more appropriate for the characterization of peptides. In comparison, SinTri was identified as the most suitable ILM for the analysis of HMW proteins, such as IgG. Shrivas et al. subsequently demonstrated the superiority of CHCAB for peptide detection.^598^ Compared with the CHCA matrix the use of CHCAB as a matrix for the detection of enzymatically cleaved cytochrome c peptides effectively increased the detected signal intensity (10-fold) and the number of peptide sequences. Furthermore, Fukuyama et al. developed a novel ILM, TMG salt of G_3_CA, an advance on previous research.^609^ The G_3_CA matrix facilitated the preferential ionization of glycopeptides derived from the enzymatic degradation product of ribonuclease B (RNase B) in both positive and negative ion modes, with a particular emphasis on the latter. Thereafter, inspired by the work of Kolli et al.,^994^ they proposed a novel liquid matrix, 3-AQ/G_3_CA, for the detection of glycopeptides and phosphopeptides.^660^ The results demonstrated that 3-AQ/G_3_CA exhibited enhanced or comparable detection sensitivity relative to that of the DHB and 3-AQ/CHCA matrices. All analytes were detected as [M+H]^+^ or [M–H]^−^, and the matrix was observed to inhibit the dissociation of unstable regions, including sialylated and phosphorylated modifications in peptides. Calvano et al. evaluated the performance of an ILM composed of CHCA and ANI.^594^ Compared with CHCA, the CHCA/ANI combination yielded comparable or superior S/N ratios for MALDI-MS detection. It demonstrated efficacy for a range of analytes, including AAs, peptides, proteins, and other classes, which suggested its potential as a general-purpose matrix. Moreover, Crank and Armstrong systematically demonstrated that modifying the cationic and anionic constituents of IL could impart distinct properties to ILMs.^63^ Their findings revealed that DIEA/CHCA and IMTBA/CHCA were the most effective for protein and peptide detection.

The development of novel inorganic matrices for the application of AAs, peptides, and proteins is occurring at a remarkable pace in comparison with that of traditional organic matrices and ILMs. For example, Sazbo et al. utilized derivatized fullerenes that were covalently bound to the surface of silica particles with varying pore sizes as an efficacious matrix for MALDI analysis of AAs and peptides, as well as other biomolecules (<1500 Da).^995^ The findings demonstrated that the developed fullerene–silica matrix was effective at reducing or even eliminating the interference of matrix ions. Furthermore, the D/I capacity of the matrix was found to be influenced primarily by three key factors: the applied laser power, sample preparation, and pore size of the silica particles. Kuo et al. constructed a rapid and efficacious MALDI-TOF-MS analysis platform based on the layered structure of reduced GO (rGO) and AuNPs.^996^ The multi-layer thin film prepared by alternate layer-by-layer deposition of rGO and AuNPs (LBL rGO/AuNP) could be employed as both a sample plate and a matrix in MALDI-TOF MS, which has been instrumental in facilitating the successful analysis of small molecules, including AAs and peptides. By altering the number of layers in the film, the MS signals could be optimized. Compared with the use of AuNPs or CHCA as a matrix alone, the incorporation of LBL rGO/AuNP markedly increased the signal intensity, S/N ratio, and reproducibility of the analytes detected by MALDI-TOF-MS. In addition, Lin et al. introduced ultrathin g-C_3_N_4_ nanosheets as a novel matrix for the detection of small molecules by MALDI-TOF MS in the negative ion mode.^851^ Compared with conventional organic matrices (*e.g.*, CHCA and 3-AQ) and graphene, g-C_3_N_4_ nanosheets exhibit advantages such as an absence of matrix background interference and enhanced signal intensity in the analysis of AAs and peptides. The potential ionization mechanisms associated with the g-C_3_N_4_ nanosheet matrix are discussed further in their paper. Chen et al., demonstrated that CNTs generated from reactive anodic aluminum oxide templates could be employed as suitable MALDI matrices for the analysis of peptides and small proteins.^787^ The incorporation of citric acid buffer into the CNT matrix system diminished the signal intensity of the basic cation adducts while simultaneously extending the detectable mass range. Notably, the new generation of materials based on QDs^997^ and MOFs^998^ has also been expanded into the field of proteomics research. These materials were used as novel matrices for the MALDI-MS analysis of AAs, peptides (*e.g.*, phosphopeptidesglycopeptides), and proteins. Related studies have been systematically summarized.

The aforementioned matrices are suited primarily for UV-MALDI-MS analysis; however, for IR-MALDI analysis, other appropriate matrices are necessary. Pirkl et al. developed a temperature-controlled sample stage and successfully applied it to orthogonal TOF-MS with the objective of enhancing the suitability of water ice as an IR-MALD matrix.^999^ The water–ice matrix enabled the analysis of large molecules, including noncovalently bound holo-myoglobin and RNase B, and was employed for the first time in the analysis of IgG monoclonal antibodies (MW: 150 kDa). Unusually for MALDI-MS, this approach enabled the observation of high charge states of multiple protonated species, such as the investigated peptides and even for lacto-*N*-fucopentaose II oligosaccharides.

#### 4.1.2 MALDI Matrices for Targeted Amino Acid, Peptide and Protein Analysis

##### 4.1.2.1 Amino Acids and Derivatives

MALDI-MS has been successfully used for qualitative and quantitative analysis of free AAs and protein hydrolysis products. While the aforementioned nontargeted MALDI matrices can effectively address the shortcomings of conventional matrices in AA detection, namely the overlap of matrix fragmentation peaks in the low-mass region of the spectra with those of the analytes, there is still scope for improvement in the selective detection of AAs by these matrices. It is therefore important to develop novel matrices with favorable targeting properties to increase the efficiency and accuracy of AA detection. The performance of two surfactants, namely, sodium dodecyl sulfate (SDS) and sodium octyl sulfate (SOS), as MALDI matrices (laser wavelength: 1064 nm) for the detection of several AAs (Phe, valine (Val), proline (Pro), alanine (Ala), and tyrosine (Tyr)), was investigated by Yazdabadi et al.^1000^ Furthermore, the impact of the repeller plate material on AA detection during the ionization stage of the mass spectrometer was examined, and the results were compared with those obtained from direct-LDI TOF spectra. The results demonstrated that Na^+^ tended to be transferred to Phe and Val when SDS was employed as a matrix. Moreover, the peak intensities of [M+Na]^+^ and [M–H+2Na]^+^ markedly increased when Ag was utilized as a repeller plate material. However, other AAs responded poorly to the SDS matrix. In addition, the carbon chain length of the SOS matrix affected the MALDI spectrum of Phe, resulting in the disappearance of the crucial sodium addition peak. To investigate the formation mechanism of the sodium addition peak, researchers employed density functional theory calculations to characterize the structures of [M+Na]^+^ and [M–H+2Na]^+^ ions, identifying the amino acid interaction sites where Na^+^ binds. Meanwhile, the standard Gibbs free energy changes (ΔG°) for the reactions M+Na^+^ → [M+Na]^+^ and [M+Na]^+^+Na^+^ → [M–H+2Na]^+^+H^+^ were simultaneously calculated. According to the Δ*G*° values, the attachment of the first Na^+^ to the amino acid occurs in the gas phase, while the attachment of the second Na^+^ to [M+Na]^+^ does not represent a favorable process in the gas phase.^1000^ A series of AIE matrices based on the Schiff base reaction have been synthesized for the analysis of small-molecule compounds by MALDI.^576^ The development of this matrix decreased spectral background interference, improved detection sensitivity, and modulated ionization efficiency for specific analytes by changing the aggregation state. This method has been successfully applied to the direct detection of Gly in human urine. Shanta et al. developed a new combined matrix of 3-HC and ATT that is capable of efficiently ionizing small molecules, including single AAs (*e.g.*, Ala, methionine (Met), threonine (Thr), and Trp) and some drugs.^641^ It is also expected to be used for tissue surface MSI, as the combined matrix produced less background signal in the LMW range and had higher vacuum stability. In a recent publication, Fu et al. described a novel organic matrix, DHT, for the selective detection of AAs in biological samples.^359^ The results demonstrated that DHT exhibited strong UV-vis absorption, uniform matrix deposition, and high vacuum stability, which resulted in significantly fewer matrix-associated ions at *m*/*z* < 500 compared to the conventional DHB and CHCA matrices. The detection of four AAs (*i.e.*, Arg, glutamic acid (Glu), glutamine (Gln), and Pro) was achieved at concentrations as low as 6, 4, 6, and 4 ng/mL, respectively, when DHT was used as the matrix. In addition, as shown in Figure 81a, DHT enabled the successful detection and imaging of 20 protein AAs and taurine in human serum samples and edible oyster (*Crassostrea gigas*) tissue sections, thereby opening up new avenues for using *in situ* MALDI-MSI to detect and image AAs in biological samples. Zhou et al. subsequently designed, synthesized, and tested a series of pyridine salt probes, and their screen identified 1-(4-(((2,5-dioxopyrrolidin-1-yl)­oxy)­carbonyl)­phenyl)-2,4,6-triphenylpyridin-1-ium (DCT) as the candidate with the highest reaction efficiency and best detection performance.^1001^ Using the DCT probe with a CHCA matrix, 20 common AA patterns were discovered for the first time in normal mouse tissue (Figure 81b), thereby distinguishing the distribution of AA in normal, normal interstitial, tumor, and tumor interstitial regions, providing potential mechanistic insights for cancer research and other types of biomedical research.

**Figure 81:**
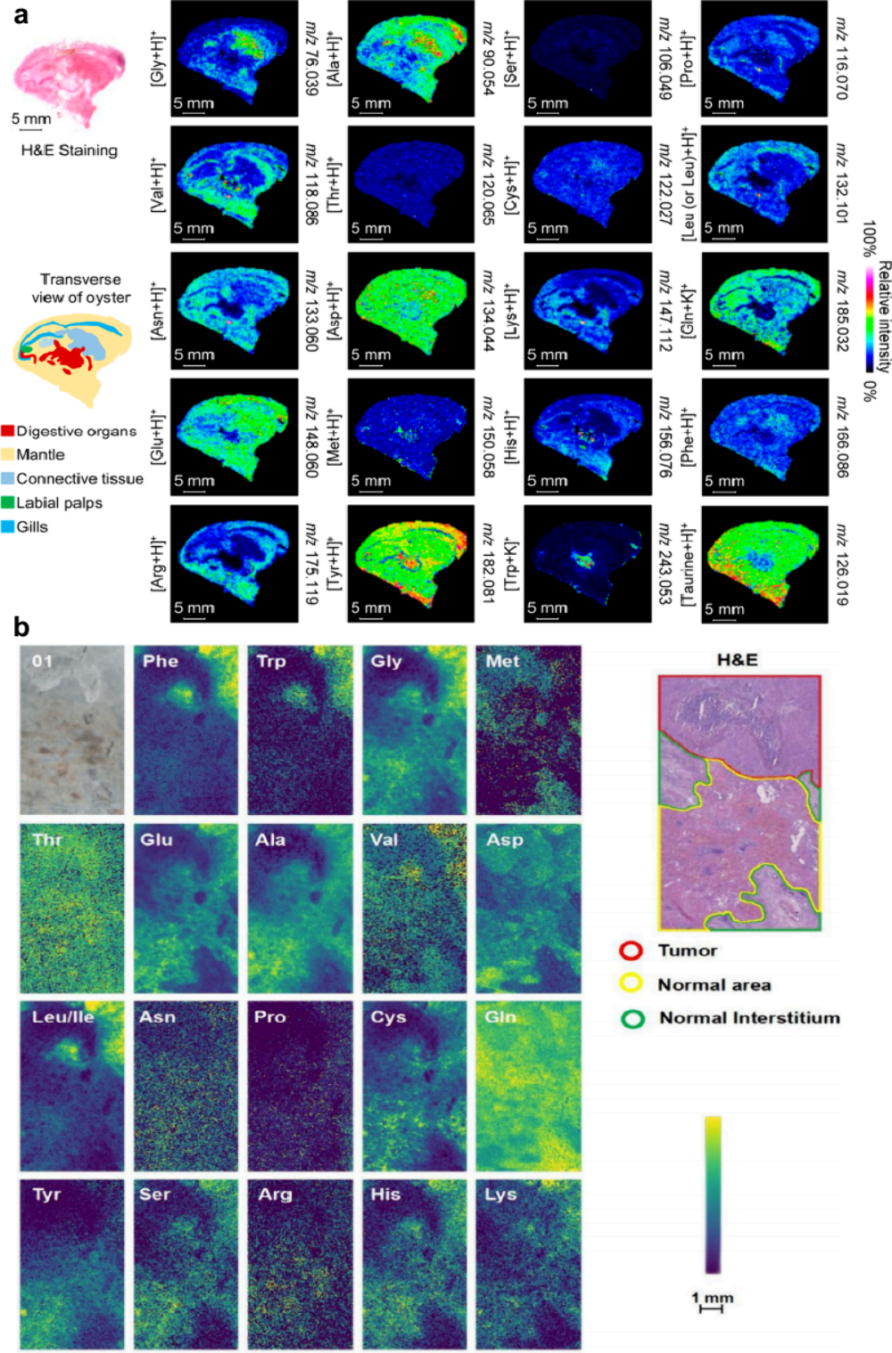
(a) Ion images of AAs from edible oyster tissue sections using DHT as the matrix. H&E staining was performed after MALDI-MSI. MSI achieved 275 μm spatial resolution. Adapted and reproduced with permission from ref 359. Copyright 2023 American Chemical Society. (b) MALDI-MSI visualization of common AAs extracted from human hepatocellular carcinoma tissue sample No. 1 after DCT derivatization. Adapted and reproduced with permission from ref 1001. Copyright 2024 American Chemical Society.

Wang et al. prepared oxidized CNTs with short and open-ended structures on PAA templates via chemical vapor deposition in 2007.^793^ The CNTs were subsequently employed as a valuable matrix for MALDI-FTMS analysis, which resulted in a notable increase in the sensitivity of AA detection, with successful applications observed in the analysis of leucine and isoleucine in corn roots. Similarly, Zhang et al. successfully analyzed 20 common AAs using CNT as the matrix by MALDI-FTMS.^1002^ Their findings revealed that CNT possessed the properties of dispersing analytes, absorbing UV light, and transmitting energy with minimal background interference and analyte fragmentation. Furthermore, all the AAs exhibited the most robust signals for sodium ion adducts (*i.e.*, [M+2Na–H]^+^), presumably because of the replacement of a proton in the carboxyl group by a sodium ion, whereas another sodium ion was introduced to the corresponding molecule through electrostatic forces. Moreover, Dong et al. used graphene as a matrix for the first time in MALDI-TOF MS to analyze LMW compounds such as AAs.^68^ Compared with previously available matrices, graphene had enhanced D/I efficiency for nonpolar compounds. The use of graphene as a matrix effectively circumvented the interference of matrix ions on the spectra and the fragmentation of analytes. In addition, it exhibited high salt resistance and satisfactory reproducibility. Qiu et al. synthesized Co_3_O_4_ nanocrystals enriched with surface hydroxyl groups as a novel matrix for the detection of small molecules by MALDI-TOF-MS.^692^ In comparison with reported organic matrices, the prepared Co_3_O_4_ nanocrystal matrices were characterized by low matrix background interference, excellent reproducibility, and high signal intensity in AA analysis. The majority of the AAs displayed favorable detection performance when analyzed using the Co_3_O_4_ matrix, in both positive and negative ion modes. Furthermore, a distinctive decarboxylation peak was easily produced in positive ion mode, which facilitated the identification of AAs. In a separate study, Marsico et al. employed monolayer-protected AuNPs as a matrix for the detection of LMW compounds.^1003^ By optimizing the appropriate deposition method, density, size, and monolayer surface chemistry of the AuNPs, it was possible to detect AAs at very low concentrations (fmol) with minimal interference. The AuNPs deposited using the inkjet-printing technique exhibited a more uniform distribution, thereby enabling the detection of AAs by MALDI-MS at any location on the printed surface, which in turn facilitated the attainment of reproducible results. Moreover, the aggregation of AuNPs was a requisite step for achieving the lowest detection limit, indicating not only that there was an optimal surface density of AuNPs but also that their aggregation state significantly influenced the ionization mechanism of the analyte. O'Neill et al. employed a chemical derivatization strategy utilizing 4-hydroxy-3-cinnamaldehyde to increase the ionization efficiency and detection performance of AAs. By employing AuNPs as a matrix, they effectively demonstrated the abundance and distribution of AAs within the root systems of B73 and Mo17 corn, indicating that the hybrid retains characteristics inherited from both parental lines.^1004^ The results of this study revealed that B73 has a relatively low abundance of AAs, primarily located in the root center, whereas the abundance of Mo17 is relatively high, mainly in the root cortex. Additionally, the presence of ^15^N-ammonium ions provided insights into amino acid synthesis, showing that the localization of specific AAs, such as leucine/isoleucine and Gln, remained unaffected by the nitrogen source.

##### 4.1.2.2 General Peptides and Proteins

Peptides are ubiquitous in bodily fluids, cells, and tissues and perform a multitude of physiological functions, acting as hormone messengers, cytokines, antimicrobial agents, and protease inhibitors, among others.^1005^ The molecular forms of these bioactive peptides are typically not directly predictable from genomic sequences because they are released through specific multistep processing pathways within the cell. In contrast, proteins are more complex in structure than peptides and fulfill a wide range of biological roles. The human genome comprises approximately 20,000 protein-coding genes, which collectively produce approximately 1.8 million protein species, forms, and variants. These include the expression of individual proteins, protein isoforms, and post translational modifications of proteins,^1006^ such as phosphorylation, glycosylation, acetylation, methylation, ubiquitination, glycosylation, and oxidation/reduction.^1007^ However, these diverse modifications of proteins (such as oxidation) may reduce their susceptibility to degradation by specific proteases such as trypsin to some extent, thereby affecting subsequent studies based on enzymatic hydrolysis reactions.^1008,1009^ First, the introduction of modified groups may directly shield or alter trypsin's recognition sites (lysine and arginine), hindering effective enzyme-substrate binding. Second, modifications may alter protein tertiary structure, inducing molecular cross-linking and aggregation. This form aggregates that are difficult for proteases to access, significantly reducing the availability of cleavage sites. Finally, when modifications occur directly on residues at cleavage sites, they render these sites unrecognizable by proteases, leading to complete inactivation of the site. Consequently, the combined use of multiple proteases and targeted optimization strategies may serve as complementary approaches. As the main initiators of biological activities, proteins are the most important surface molecules affecting the function of living cells and are the main targets for cancer therapy. Given the pivotal role of peptides and proteins in biological processes, researchers have devoted considerable effort and achieved notable advancements in related fields of study. In particular, MALDI-MS-based peptide and protein studies have advanced significantly. In addition to the conventional nontargeted matrices, an increasing number of targeted matrices are being employed for the biological characterization of peptides and proteins.

To date, CHCA remains the most commonly used matrix for detecting peptides using MALDI-MS. Ucal et al. demonstrated that incorporating varying concentrations of ADP into the CHCA matrix significantly diminished matrix cluster formation while increasing peptide signal intensity.^1010^ The study further revealed that ADP improved the spectral quality of MALDI-MSI, enhancing detection sensitivity and mass accuracy, thus highlighting the potential of this combination for clearer peptide imaging in FFPE tissues. Qin et al. used CHCA as a matrix to detect and image peptide biomarkers in castor beans, significantly enhancing detection sensitivity and spatial distribution through optimized sample preparation.^1011^ The study demonstrated the effectiveness of the matrix in identifying castor bean-specific biomarkers (*Ricinus communis* biomarkers, RCBs), essential for determining the geographical origin and variety of castor bean seeds. MS imaging as shown in Figure 82a, revealed that RCB-1 to -3 were distributed throughout the endosperm but enriched in the testa and embryo tissues, particularly in Ethiopia and Pakistan. Notably, the compartmentalization of RCB-1 to -3 varied among samples from different countries, and RCB-4 or RCB-5 were not detected in any castor bean sections from nine geographic origins. Claes et al. integrated MALDI-MSI with immunohistochemistry (IHC) to conduct both targeted and untargeted spatial proteomics analyses of breast cancer tissues, utilizing CHCA as the matrix to achieve high multiplexity in imaging multiple biomarkers.^1012^ This approach offers new insights for molecular pathology research in breast cancer. Similarly, Bindi et al. successfully identified numerous peptide features in kidney tissue samples fixed with BFPE and FFPE using CHCA as the matrix, revealing significant overlap between the two fixation methods.^1013^ This finding indicates that the CHCA matrix effectively enhances peptide ionization and detection, which supports the results of subsequent imaging analyses. Additionally, the CHCA matrix effectively distinguished major kidney regions and substructures, such as glomeruli, tubules, and blood vessels, further confirming its effectiveness in peptide imaging. Grgic et al. developed an optimized MALDI-MSI strategy that achieves high spatial resolution at the single-cell level by sublimating the CHCA matrix followed by immersion in ice-cold ammonium phosphate monobasic (AmP).^1014^ The AmP immersion step was found to be critical for preventing matrix aggregation and enhancing peptide signal intensity. This method achieved an impressive pixel resolution of 5 μm while preserving the matrix crystal size at approximately 400 nm. Using this method, researchers detected 89 peptide peaks in a single MDA-MB-231 breast cancer cell and successfully identified 24 peptides corresponding to 17 proteins, including actin, wave protein, and transglutaminase-2, through LC-MS/MS analysis. Additionally, Macdonald et al. highlighted that using collagenase for tissue digestion improves peptide detection with the CHCA matrix, as it targets the triple helix structure of collagen, generating distinctive peptides that enhance MALDI-MSI capabilities.^1015^ Optimized sample preparation methods, such as liquid surface extraction after collagenase digestion on tissue sections, may make it possible to obtain richer peptide information and improve the peptide identification capability of MALDI-MSI.

**Figure 82:**
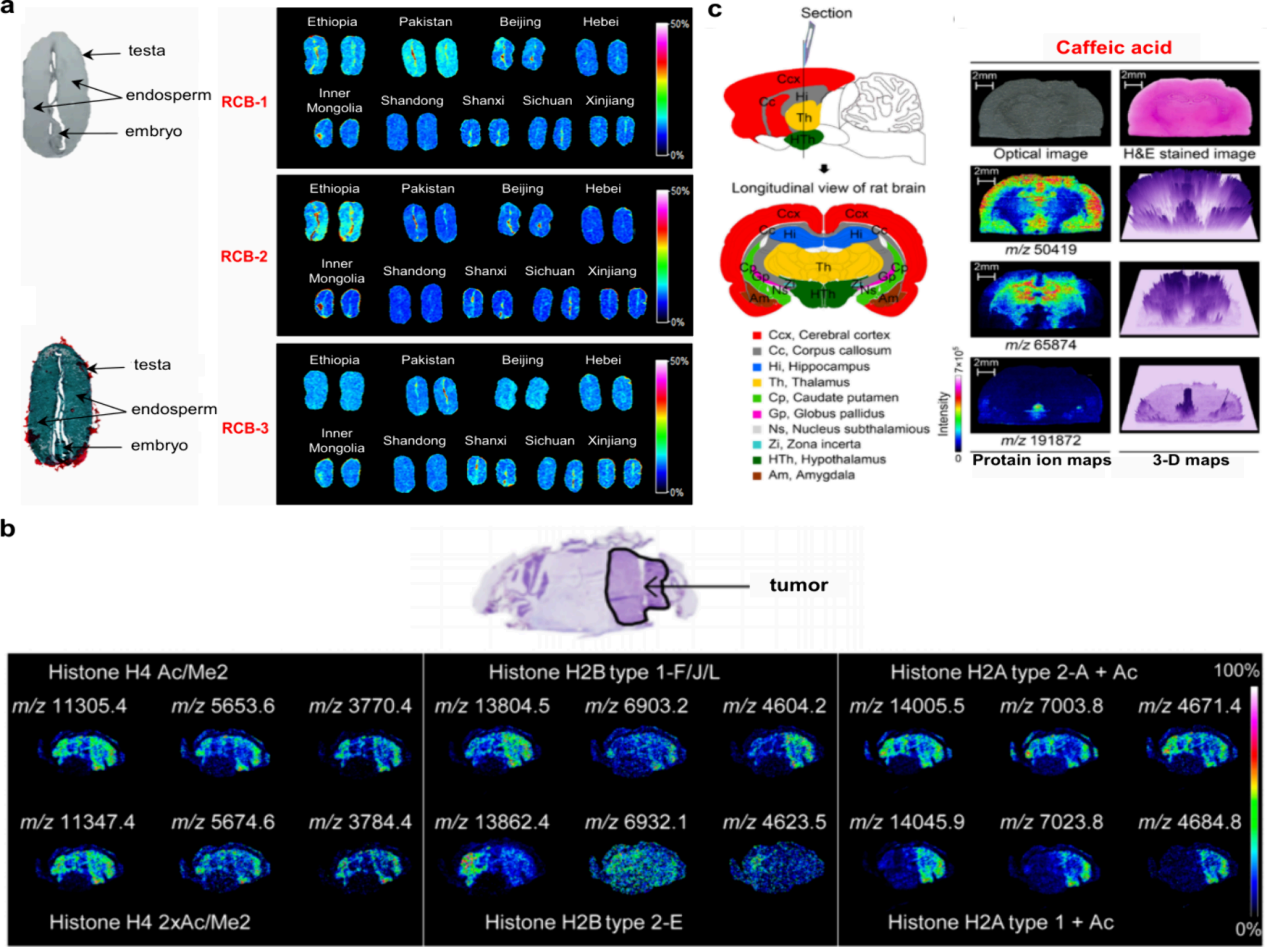
(a) MALDI-MSI analysis of RCBs in positive ion mode in castor bean sections. Distribution of RCB-1, -2, and -3 in castor beans from different geographical sources. Color scales encode arbitrary ion relative strength. Adapted and reproduced with permission from ref 1011. Copyright 2022 The Authors under the terms of the Creative Commons CC BY license. (b) MS images of the differential regulation of histone proteoforms. Protein assignments were made via alignment with the LC-MS/MS results for microdissected samples of tumor and healthy regions of tissue. Adapted and reproduced with permission from ref 1022. Copyright 2017 The Authors under the terms of the Creative Commons CC BY license. (c) *In situ* detection and imaging of endogenous proteins from rat brain sections by MALDI-TOF MS using CA as a matrix. MS imaging was acquired at a 200 μm spatial resolution. The three-dimensional maps of these protein ions are included. ND, protein ion signals that cannot be detected and imaged. Adapted and reproduced with permission from ref 35. Copyright 2021 American Chemical Society.

In terms of peptide detection, in addition to CHCA, which exhibits excellent detection performance and is widely used, the DHB matrix also has relatively widespread application. For instance, Chen et al. successfully utilized DHB to detect and map the distribution of melanotan II and its metabolites across various mouse tissues, such as the heart, lungs, liver, muscles, and kidneys, thereby affirming its suitability for MALDI-MSI in drug distribution studies.^1016^ Cintron-Diaz et al. utilized the DHB matrix for imaging neuropeptides in human pituitary FFPE tissue sections using MALDI-MSI and ultra-high-resolution FT-ICR MS technology. They successfully detected Arg vasopressin and oxytocin, revealing distinct isotopic patterns and elemental distributions with approximately 1 ppm mass accuracy, and confirmed their amino acid sequences through MS/MS analysis.^1017^ Dunne et al. demonstrated that combining antibody-guided single-cell imaging with MALDI-MSI using a DHB matrix effectively detects and images extracellular matrix proteins in FFPE tissues, facilitating the study of cellular and extracellular components in the tissue microenvironment.^1018^ Uras et al. used a DHB matrix for MALDI-MSI analysis of newborn brain tissue from AD transgenic mouse models, identifying expression differences in several AD-related proteins linked to synaptic plasticity, axonal degeneration, and autophagy, some of which correlate with the disease’s pathological features.^1019^ Additionally, Rampal and collaborators developed a MALDI-MSI imaging method that enhances the detection of extracellular matrix proteins through decellularization and sequential enzymatic digestion (PNGase F and trypsin), using DHB as a matrix to visualize the spatial distribution of protein-related peptides.^1020^ Their results revealed that decellularization significantly increased the variety of detectable proteins, achieving high-resolution imaging at 100 μm and 50 μm, whereas combining accurate mass measurement with binary colocalization methods further improved protein identification accuracy, supporting the use of MALDI-MSI for ECM protein distribution studies.

In addition to CHCA and DHB, some researchers have used 2-MBT as a matrix for peptide detection and imaging characterization. For example, Liu et al. employed 2-MBT as a matrix for peptide detection and imaging in *Bombyx batryticatus*, a traditional Chinese medicine derived from the larvae of *Bombyx mori* silkworms infected with *Beauveria bassiana.* ^1021^ Their findings revealed distinct spatial distribution patterns of various AAs and peptides in *Bombyx batryticatus* tissues such as the silk gland, digestive tract, mesenchyme, and epidermis. Notably, beauvericin was concentrated in the peripheral regions of silk glands, whereas bassianolide was found predominantly in the epidermal layer. The study also revealed significant changes in the AA and peptide contents following high-temperature roasting, with increases in homoserine and decreases in gamma-aminobutyric acid (GABA) and L-Arg, likely due to protein degradation and thermal effects.

Similarly, SA is the most commonly used and relatively effective matrix for MALDI-MS analysis of proteins. Dilillo et al. achieved ultra-high-resolution imaging of proteins in mouse glioma tissue using the SA matrix, enabling MALDI-FTICR-MSI to detect intact proteins in the 3.5–16 kDa mass range with full isotopic resolution.^1022^ Selected MS images, as illustrated in Figure 82b, reveal multiple post translational modifications of histones H2A, H2B, and H4, which were detected as the singly, doubly, triply charged ions, with each charge state exhibiting similar distributions. This method facilitated the differentiation of overlapping protein ions and, when integrated with laser capture microdissection, provided confirmation of changes in protein expression associated with tumor metabolism. Liu et al. used SA as the matrix to enhance protein detection in 3D-cultured colon cancer cell spheroids via MALDI-MSI, achieving the clear visualization of cetuximab light chain fragments.^1023^ Their method involved *in situ* reduction and alkylation, which broke down large cetuximab molecules into smaller fragments, overcoming traditional MALDI-MSI limitations for high molecular weight proteins. This approach improved detection sensitivity and resolution while preserving the spatial distribution of the drug, proving effective in patient-derived colorectal tumor organoids. Moreover, Yin et al. utilized MALDI-MSI to analyze protein distribution in transgenic glyphosate- and glufosinate-resistant soybeans, wild soybeans, and F2 hybrid seeds, demonstrating that the SA matrix enhanced signal intensity and revealed significant spatial differences in proteins within the *m*/*z* range of 700–6,000.^1024^ The results indicated that wild soybean-specific proteins, particularly proteins associated with defense and stress responses, were more abundant in the seed margins, whereas key proteins such as glycogen synthase showed variations linked to differences in seed germination and growth adaptability among the soybean types.

While traditional matrices such as CHCA, DHB, and SA remain pivotal in MALDI-MS-based peptide and protein detection, new members of the matrix family need to be explored to address the complexities of proteomic analysis. Notably, a series of novel and effective matrices have been successfully developed on the basis of CHCA. In MALDI-TOF MS analysis, matrix clusters and their alkali metal ion adducts have the effect of suppressing peptide signals, particularly in the low mass range (500–1400 Da), which poses a challenge for peptide fingerprinting analysis and protein identification. Kim’s team employed NTA as a dopant to CHCA with the objective of increasing peptide signal strength.^666^ This approach involved the use of a non-UV-absorbing chelator that inhibited matrix clustering and enhanced the peptide signal. Their results demonstrated that the addition of 7 mM NTA led to a notable reduction in matrix clustering and an improvement in the S/N ratio by a factor of 5 to 20, ensuring the reliability of protein identification and sequence coverage. In quantitative proteomics, the NBS method represents a stable isotope labeling technique for Try residues. Matsuo et al. reported that 3H4NBA exhibited selective ionization and detection capabilities for NBS-labelled peptides in comparison to DHB.^337^ In addition, 3H4NBA was capable of selectively detecting nitrotyrosine-containing peptides, which was a crucial aspect for the study of reactive nitrogen species and associated pathological conditions. Lascoux et al. devised a novel peptide assay that employs the *N*-hydroxysuccinimide ester (NHS) derivative of CHCA for the labeling of lysine (Lys) residues.^554^ The use of the CHCA matrix for MALDI MS analysis demonstrated a proportional relationship between the signal intensity of labeled and unlabeled peptides and peptide abundance. However, when the neutral matrix CHCE was used, the signals of the labeled peptides were markedly amplified, whereas the signals of the unlabeled peptides were almost undetectable. Consequently, the combination of CHCA-derived tags and the CHCE matrix could markedly increase the sensitivity of MALDI detection in the context of labeled compounds, thereby mitigating the potential for protein structure interference. Wang et al. synthesized a series of carboxyl-esterified derivatives of CHCA and evaluated them individually as MALDI-MS matrices for protein analysis.^555^ Among these derivatives, CHCA-C3 demonstrated the most promising detection performance for intact proteins, resulting in a 10-fold increase in detection sensitivity and high salt tolerance. This was primarily attributable to the enhanced ionization efficiency of the CHCA-C3 matrix, which was achieved through optimized stripping capability and increased hydrophobicity or affinity for proteins in comparison to those of conventional matrices. Additionally, the CHCA-C3 matrix exhibited exceptional efficacy in the analysis of low-abundance proteins within complex biological samples. The Jaskolla team developed ClCCA as a novel matrix for MALDI-MS.^54^ This matrix demonstrated remarkable utility in proteomics, achieving enhanced peptide detection sensitivity and a more uniform reaction with peptides of varying alkalinity. The utilization of ClCCA as a matrix resulted in enhanced sequence coverage in 1 fmol of BSA solution enzyme-digested samples, reaching up to 48% compared with 4% in CHCA matrices. Furthermore, ClCCA facilitated the accurate identification of proteins in gel bands containing 25 fmol of BSA. Copper ions play pivotal roles in numerous biological processes, particularly in the context of their interactions with peptides and proteins, which are instrumental in the pathogenesis of neurodegenerative diseases such as AD. Wu et al. innovatively synthesized a copper-containing MALDI matrix, namely, the dinuclear copper complex ((CHCA)_4_Cu_2_), through the reaction of CHCA with copper oxide (CuO).^1025^ This matrix significantly increased the signals of the copper-binding peptide ions ([M+*x*Cu+(*x*–1)­H]^+^), thus improving the signal quality of the MS/MS, which was beneficial for the study of peptide–copper complexes in terms of their binding and cleavage chemistries.

Given the shortcomings of conventional SA and CHCA matrices in protein and peptide detection, Salum et al. sought to develop a novel MALDI matrix, *Z*-SA, by considering the cis–trans isomers of existing SA.^268^ They then evaluated its peptide detection performance relative to that of *E*-SA and CHCA. The experimental results demonstrated that compared with the conventional matrix, the *Z*-SA matrix yielded fewer cluster signals in the low *m*/*z* region and exhibited superior detection of hydrophobic and hydrophilic peptides. This proved advantageous in addressing the constraints associated with the conventional matrix in the analysis of LMW peptides. Kato and coworkers synthesized a range of halogenated FAs as MALDI matrices and compared them with CHCA and DHB.^273^ The results established that the introduction of Br at the 6-position of FA was the optimal position, and the resulting 6-BFA could conveniently detect a wide range of peptides, particularly those in the 3–5 kDa range and those containing acidic AAs or proline. Concurrently, 6-BFA was shown to be an appropriate matrix for MS/MS analysis using a MALDI-TOF-MS. In addition, Yang et al. facilitated the integration of peptide extraction, concentration, and MALDI analysis by using a three-component system consisting of CHCA, 3-AQ, and quinoline as a liquid matrix, which could rapidly enter the aqueous phase with the help of ultrasonic dispersion.^1026^ The application of this matrix decreased the detection limit of peptides such as angiotensin I in aqueous solution to as low as 1.25 nM, providing better coverage of the peptide sequences compared with conventional MALDI matrices. This method was successfully applied to sample cleanup, preconcentration, and the *in situ* analysis of peptides in the presence of signal inhibitors such as Tris buffer and urea.

With the advancement of research, a plethora of innovative organic matrices have been developed for the analysis of peptides and proteins. The SA matrix is a prevalent choice in UV MALDI protein analysis; however, it cannot form a diverse range of distinctive protein charge states. In contrast, the 2-NPG matrix possesses the capacity to detect multiple protein charge states, offering a distinct advantage in accuracy for the MALDI-MS analysis of proteins. In a comparative study of mass spectra, Choi et al. examined cytochrome c, myoglobin, BSA, and IgG in diverse matrices, including DHB, CHCA, SA, and 2-NPG.^658^ Their findings revealed that 2-NPG exhibited the most extensive range of multiple charge states, while the HCl-bound 2-NPG matrix yielded the most robust protein peaks. In general, low sample abundance and salt interference have constituted significant challenges for MALDI protein profiling. The Monopoli team demonstrated the effectiveness of the synthetic matrix CPPA in analyzing proteins by MALDI-TOF MS, particularly for complex samples such as foods and bacterial extracts.^561^ The CPPA matrix, which significantly increased protein signals, and diminished point-to-point variability, was successfully deployed for protein profiling of microorganisms, milk, and seed extracts. Compared with conventional matrices such as SA, CHCA, and CClCA, CPPA exhibited a superior S/N ratio and uniform responses for the majority of proteins examined, including those present in milk, hazelnut, and intact bacterial cells of *E. coli*. Liu et al. assessed the potential of CA as a MALDI matrix, achieving successful *in situ* protein detection and imaging in three biological tissue sections: rat brain, *Capparis masaikai* seed, and germinated soybean seeds.^35^ The results demonstrated that CA exhibited strong UV absorption, a wide mass detection range (close to 200,000 Da), micrometer-sized matrix crystals, uniform deposition, and high ionization efficiency. *In situ* detection and imaging of endogenous proteins in rat brain slices were performed using MALDI-TOF-MSI. The use of the CA matrix allowed the clear identification of a greater number of high molecular weight protein ions, which exhibited exceptional in situ detection capabilities within biological tissues at *m*/*z* values exceeding 80,000. Furthermore, the application of the CA matrix significantly enhanced the imaging of surface proteins in selected biological tissues, showing promise for the tissue imaging of high molecular weight proteins in both animal and plant specimens (Figure 82c). Denti et al. reported that ATT is an effective alternative matrix for MALDI-MSI in spatial proteomics analysis, enabling the detection and *in situ* imaging of trypsin-digested BSA peptides in FFPE tissue sections.^1027^ Compared with the traditional CHCA matrix, ATT exhibited superior ionization efficiency across a wide *m*/*z* range, resulting in enhanced BSA sequence coverage (47%) and a clearer spatial distribution of analytes. Additionally, ATT produced fewer matrix peaks in the low *m*/*z* range, minimizing adduct formation and simplifying spectrum interpretation. Although its stability is lower under high vacuum conditions, ATT maintains the analyte signal intensity for at least 7.5 hours without compromising the visualization of biologically relevant molecules, highlighting its potential to advance spatial proteomics.

ISD is a fragmentation method in MALDI-MS, which occurring in the MALDI source, rapidly after the laser shot and before the ion extraction. ISD facilitates rapid peptide sequencing through the detection of *c*- and *z*-series ions, or *a*- and *x*-series ions with *d*-series ions, and its effectiveness dependent on the choice of matrix.^338^ DAN, the most commonly used reduced matrix in ISD, is of interest because of its high sensitivity to fragment ions. However, the *c*- and *z*-series of ions produced by DAN upon N-Cα bond breaking resulted in challenges in differentiating between Leu and Ile and the inability to generate the *c*_(*n*–1)_-series of ions when Pro is at residue *n*.^338^ While oxidized matrices can address this problem by facilitating the generation of both α- and β-series ions through Cα–C bond cleavage, they exhibit reduced sensitivity to ISD fragment ions in comparison with reduced matrices, such as DAN. To this end, Fukuyama et al. developed a novel MALDI oxidative matrix, 3H2NBA, for the ISD analysis of peptides.^338^ Their results demonstrated that 3H2NBA possesses oxidative matrix properties that facilitate the generation of both α- and β-series ions through Cα–C bond cleavage. Furthermore, the ions produced by 3H2NBA exhibited peak intensities comparable to or higher than those produced by DAN, while also demonstrating significantly enhanced resolution in some instances. Concurrently, 3H2NBA proved effective in resolving several complications present in DAN, including the differentiation between Leu and Ile through the generation of *d*-series ions.

In contrast, numerous inorganic matrices have been developed with the specific intention of being used for MALDI-MS analysis of peptides and proteins. To illustrate, the study conducted by McLean et al. employed AuNPs as a low-concentration, selective matrix for the ionization of biomolecules.^690^ The effectiveness of AuNPs of varying sizes as LDI matrices was investigated, and the ionization of a range of peptides and small proteins, including post-translationally modified peptides, was successfully achieved. Additionally, it was demonstrated that AuNPs can selectively ionize pTyr-containing peptides, which is crucial for investigating their functions in cells. Similarly, Duan et al. achieved successful improvements in peptide ionization by modifying self-assembled monolayers terminated with CHCA on the surface of AuNPs.^978^ This resulted in effective inhibition of Au cluster ions and analyte fragment ions, as well as a significant increase in peptide desorption ions upon combining glycerol and citric acid. Moreover, Fleith’s team devised a novel MALDI matrix by covalently attaching CHCA to an amorphous silicon hybrid material (CHCA-SiO_2_), which showed enhanced UV absorption during laser D/I and was employed for the MALDI-MS analysis of peptide mixtures (550–1300 Da).^964^ Compared with conventional matrices, the novel matrix demonstrated superior detection sensitivity and relative ionization difference. Its ability to form a homogeneous film contributed to an enhanced peptide detection response. The ratio of specific peptide molecules, such as the tripeptide GSH to glutathione disulfide (GSSG), is closely correlated with the oxidative stress and antioxidant levels of organisms and is frequently considered an indicator of cellular redox status. Fang et al. designed a comprehensive scheme for the rapid, efficient, and cost-effective determination of the antioxidant capacity of GSH.^1028^

Fang et al. developed a comprehensive scheme for the rapid, efficient, and cost-effective detection of the antioxidant capacity of GSH. They synthesized titanium dioxide (TiO_2_)-Au/graphene nanocomposites and proposed a strategy for evaluating the antioxidant properties of beverages/fruits using MALDI-MS. This material had the capacity to oxidize GSH to produce GSSG through the generation of OH radicals under UV–visible light irradiation. Their results revealed that the composite was efficacious in extracting 0.01 mg/mL of GSH, resulting in a high S/N ratio for MS detection. Furthermore, the method was employed to determine the antioxidant capacity of various antioxidants, including vitamin C, vitamin E, and β-carotene, as well as a range of beverages and fruits. Mesoporous WTiO was also employed as a novel inorganic matrix for MALDI-TOF MS to analyze short peptides.^763^ Among the matrices tested, those with an ordered 2D or 3D structure performed better than those with a disordered WTiO matrix did. Semiconductor QDs were introduced as dopants to MALDI matrices. For example, the addition of CdSe/ZnS QDs to conventional CHCA matrices has been demonstrated to significantly improve the S/N ratio, peak quality, and number of peptides detected by MALDI-MS.^1029^ This, in turn, has been shown to improve the accuracy and sequence coverage of overall protein identification. Common pencil lead has also been shown to serve as an effective matrix for the MALDI detection of peptides and other substances.^832^ As a cost-effective, rapid, and straightforward matrix, it has certain advantages in terms of easy application method, hydrophobicity increasing sensitivity, and solvent-less preparation (directly scribbling pencil lead onto the target spot). On the other hand, Wen and coworkers used silicon nanopowder (5-nm) as a matrix to detect peptides in both positive and negative ion modes.^876^ The principal benefits of this matrix were the reduction in matrix background interference in the LMW range and the optimization of the spectral background and analyte signal intensity through improvements in particle size and sample preparation. In parallel, Piret et al. employed TiO_2_ nanotube layers as a sensitive substrate for peptide detection by SALDI-MS.^753^ The preparation of the optimized nanotube layer via electrochemical anodic oxidation, in conjunction with its controlled surface chemistry, facilitated the detection of the neurotensin peptide in peptide mixtures at limits as low as 10 fmol.

##### 4.1.2.3 Phosphopeptides

In order to gain a deeper understanding of protein function, it is necessary to elucidate post-translational modifications such as protein hydrolytic cleavage, acylation, carboxylation, glycosylation, lipidation, sulfonation, and phosphorylation in detail.^1030^ Acidic modifications of proteins (*e.g.*, phosphorylation and sulfonation) play important roles in many cellular processes, including signaling and protein–protein interactions. Among these modifications, phosphorylation represents the most fundamental, pervasive, and important mechanism for regulating and controlling protein viability and function. Conventional techniques for the analysis of phosphorylated proteins include the labeling of AAs with 32p-phosphate, followed by chromatographic or electrophoretic separation and Edman degradation.^1030^ Furthermore, phosphorylated peptides have significant implications in the fields of proteomics and post-translational modification studies. However, there are several limitations in their study because of low ionization efficiency during MALDI-MS.

To enhance the detection efficiency of phosphorylated peptides, Kjellstrom et al. discovered that the incorporation of PA into the DHB matrix led to a notable intensification of the ion signals associated with phosphorylated peptides by MALDI-MS, which was also validated in phosphorylated proteins.^659^ Similarly, this phenomenon was observed when the trypsin digest products of phosphorylated proteins of PrkC (*Bacillus subtilis*) were analyzed using LC-MALDI-MS. The results indicated that the addition of PA to the matrix not only enhanced the detection signals of the phosphorylated peptides but also reduced the interference of sodium ions, which further improved the resolution and provided a new strategy for the MALDI-MS analysis of phosphorylated proteins.^659^ In addition, Nabetani and coworkers effectively increased the ionization intensity of acidic modified peptides, such as phosphorylated and sulfated peptides, by adding ammonium salts to the matrix solution, which provided a simple and effective method for detecting these modified proteins.^1031^ The method was also successfully applied to two-dimensional electrophoresis (2-DE)-isolated proteins from *Caenorhabditis elegans*, resulting in the identification of 42 spots as modified proteins, 34 of which were non-overlapping unique proteins. In parallel, it was revealed that the *p*I shift of DIM-1 and MLC-1 proteins in 2-DE gels was found to be attributable to the presence of acidic modifications.

In addition to the previously discussed dopants, Osaka et al. investigated a novel organic matrix, 5,1-ANL, for the MALDI-MS ISD analysis of phosphopeptides.^462^ The utilization of 5,1-ANL as a matrix resulted in elevated ISD fragment ion yields for mono-, di-, and tetraphosphorylated peptides. Furthermore, the method demonstrated the capacity to achieve specific cleavage of peptide bonds while maintaining the integrity of phosphorylated modifying groups. 5-ASA has also been employed as a novel matrix for the MALDI ISD analysis of peptides.^351^ Compared with other frequently used matrices, 5-ASA has superior hydrogen-supplying capabilities. Additionally, it displays enhanced clarity in protonated molecules and fragment ions and can circumvent the formation of interfering peaks, including multiply-protonated ions and metastable ions.

CHCA can provide corroborative information regarding phosphorylation sites during the analysis of phosphorylated peptides; however, it is a “hot” matrix that frequently results in excessive fragmentation of the analyte.^1032^ To address this issue, Chen et al. performed MALDI MS detection of phosphorylated peptides by combining DHB and CHCA as a binary matrix system.^1032^ Magnetic NPs comprising Fe_3_O_4_ and Al_2_O_3_ were employed as affinity probes for the enrichment of serine (Ser) phosphopeptides from tryptic digests of proteins. The binary matrix system proved effective in determining the number of phosphorylation sites in the peptides while simultaneously reducing the detection limit to 2.5 fmol. Since then, Zhou and coworkers have further reported a novel binary matrix system that significantly improves the detection efficiency of phosphorylated peptides and mitigates reliance on laser power, obtained by mixing 3-HPA and CHCA in a 1:1 volume ratio.^634^ Compared with pure CHCA, the binary matrix not only alleviated the loss of neutrality (*e.g.*, loss of phosphate groups) and crystal morphology in the analysis of phosphorylated peptides but also provided greater sensitivity for the detection of mono- and diphosphorylated peptides. Concurrently, a wide range of phosphorylated peptides were also successfully identified in the enzymatic digestion of casein from commercially available low-fat milk samples using this matrix. Consequently, Hou et al. devised a novel binary matrix combination of DHAP and DAHC, which markedly increased the ionization efficiency of phosphorylated peptides, particularly at low ratios of DHAP/DAHC.^1033^ The binary matrix demonstrated the ability to detect phosphorylated peptides at femtomolar levels and, compared with the DHB/PA matrix, exhibited superior performance in terms of sample homogeneity and the sensitivity of phosphorylated peptide measurements. Additionally, it has the potential to identify phosphorylation sites in α-casein and β-casein, and has successfully characterized two phosphorylation sites in cyclin-dependent kinase-1 (CDK1)-treated human histone H1. Figure 83a shows the results of the MALDI-TOF-MS analysis of the tryptic digest of phosphorylated human histone H1 detected directly using the optimized DHAP/DAHC matrix in positive ion mode (without the desalting step). A total of 31 peptides were obtained, covering 81% of the sequence, including two identified phosphopeptides. The disappearance of these phosphopeptide peaks was verified by treatment with alkaline phosphatase, confirming their phosphorylation status (Figure 83b). Subsequent MS/MS analysis (Figure 83c,d) revealed only one neutral-loss peak corresponding to [MH-H_3_PO_4_]^+^, indicating that both peptides were monophosphorylated and that their phosphorylation sites were consistent with the consensus sequence recognized by CDK1, highlighting the effectiveness of the DHAP/DAHC matrix in analyzing protein phosphorylation using MALDI-MS.

**Figure 83:**
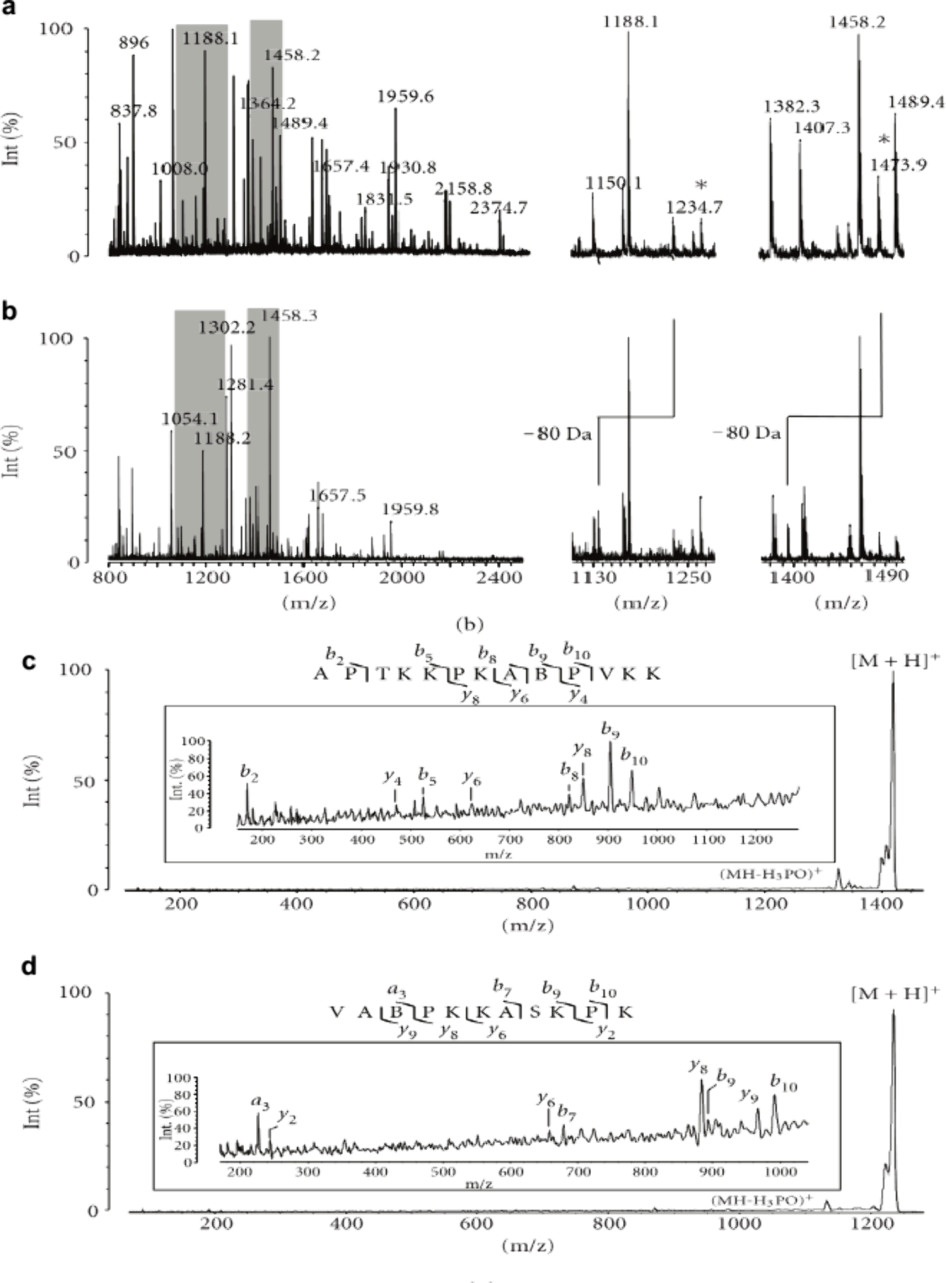
MALDI-MS analysis of phosphopeptides from CDK1-treated human histone H1. MS spectra of the tryptic peptides from (a) CDK1-treated histone H1 with the untreated and (b) alkaline phosphatase-treated histone H1. The two panels on the right show the magnified spectra to indicate the two phosphopeptides labeled with asterisks (*). Compared to the CDK-1-treated group, phosphatopeptides derived from alkaline phosphatase-treated histone H1 undergo dephosphorylation, resulting in a mass loss of 80 (HPO_3_) in the spectrum. The MALDI-TOF/TOF-MS analysis of phosphorylation sites on the two monophosphopeptides V ApTPKKASKPK, *m*/*z* 1234.7 (c), APTKKPKApTPVKK, *m*/*z* 1473.9 (d) from the CDK1-treated human histone H1. The neutral-loss peak of the phosphopeptide was noted as [MH-H_3_PO_4_]^+^. The amide bond in the peptide backbone breaks upon energy acquisition, differences in the breaking site and charge retention patterns result in the formation of *b*-ions and *y*-ions. Fragment ions carrying the N-terminal end of the peptide segment are *b*-ions, while those carrying the C-terminal end are *y*-ions. The fragment patterns of the peptides are shown in the magnified MS/MS spectra. “B” in the amino sequence indicates a dehydroamino-2-butyric acid residue converted from a phosphothreonine residue by beta-elimination of H_3_PO_4_. All the spectra were detected using a DHAP/DAHC matrix in positive ion mode. Adapted and reproduced with permission from ref 1033. Copyright 2010 The Authors under exclusive license to John Wiley & Sons.

The continuous development of liquid matrices has led to the gradual emergence of hybrid multiliquid matrices as a research focus. A notable example is the work of Fukuyama et al., who enhanced the detection sensitivity of phosphorylated peptides by optimizing the sample preparation method of the 3-AQ/CHCA matrix.^1034^ The sensitivity of the matrix was evaluated for six phosphorylated peptides using a MALDI-QIT-TOF mass spectrometer. Their results demonstrated that the novel sample preparation method, which involved the addition of diammonium hydrogen phosphate and alteration of the analytical solvent composition, led to a 10- to 10,000-fold increase in sensitivity for the detection of mono- and tetra-phosphorylated peptides in comparison to the conventional DHB matrix. The method is based on the enrichment of hydrophilic peptides within the matrix droplets, offering a novel approach for the highly sensitive analysis of phosphopeptides by MALDI-MS.

##### 4.1.2.4 Hydrophobic Peptides

Hydrophobic peptides typically contain a relatively high proportion of hydrophobic AAs, such as Ala and Ile. Additionally, their molecular structures often feature hydrophobic groups, including methyl and ethyl groups, which increase their hydrophobicity. Moreover, the presence of phenolic groups and specific ring structures can also influence the hydrophobic characteristics of these compounds. In general, the majority of cancer markers are glycoproteins that are located on the surface of cell membranes and, comprise both hydrophilic and hydrophobic regions.^1035–1037^ However, due to the inherent difficulty in detecting hydrophobic peptides, they are challenging to analyze as potential targets following glycoproteolytic digestion. To date, the primary challenge in detecting hydrophobic peptides using MALDI technology can be attributed to the fact that conventional MALDI matrices are typically hydrophilic and exhibit low affinity for hydrophobic peptides.

To address the above challenge, Fukuyama's team reported a novel dopant, ADHB, for improving the sensitivity of MALDI-MS for the detection of hydrophobic peptides.^570^ By introducing hydrophobic alkyl chains into the DHB matrix, ADHB enhances the affinity for hydrophobic peptides, allowing their accumulation at the edge of the matrix/analyte dry spot. Figure 84 displays the MALDI-MS results for phosphorylase b tryptic digestion analysis using CHCA+ADHB (Figure 84a) or CHCA alone (Figure 84b). Within the *m*/*z* range of 700–2500, both matrices detected the same peptide ions; however, in the range above *m*/*z* 2500, ADHB showed higher ion intensities, particularly at *m*/*z* 3715.2 and *m*/*z* 4899.3. Additionally, both peptides were also detected at the edge of the matrix/analyte dry spot. These findings suggest that mixing ADHB with the CHCA matrix significantly improves the detection sensitivity of hydrophobic peptides (by 10 to 100 times) without compromising the MALDI-MS detection performance and helps to increase protein sequence coverage. Furthermore, this approach also contributed to an increase in protein sequence coverage. In light of the findings of ADHB studies, Fukuyama et al. developed a novel matrix, ATHAP, which can detect peptides uniformly and enhance the sensitivity to hydrophobic peptides, solving the problem of finding “sweet spots” encountered when using ADHB.^384^ In contrast to ADHB, ATHAP can function as a matrix in isolation. In addition, ATHAP has been demonstrated to enhance the sensitivity of hydrophobic peptides by a factor of 10 compared with CHCA, facilitating the detection of [M+H]^+^, while simultaneously inhibiting the detection of [M+Na]^+^ and [M+K]^+^ and streamlining the process of spectral analysis. Subsequently, Fukuyama's team employed the ATHAP matrix to effectively detect membrane proteins containing membrane-expanded regions, which are frequently challenging to analyze by MALDI-MS with conventional matrices.^1038^ The results showed that the ATHAP matrix was capable of detecting the intact molecular ion of bacteriorhodopsin, which contains seven transmembrane structural domains. This represents a significant challenge for conventional matrices, such as SA. Furthermore, compared with the CHCA matrix, the ATHAP matrix exhibited superior sensitivity in the detection of hydrophobic enzymatic ions with single transmembrane structural domains, including cadherin 1 (CDH1), fibroblast growth factor receptor 4 (FGFR4), epithelial cell adhesion molecule (EPCAM) recombinant proteins, and human epidermal growth factor receptor type 2 (HER2). This highlights the enhanced ability of the ATHAP matrix to analyze membrane proteins, particularly those belonging to the hydrophobic region, such as those with transmembrane structural domains.^1038^ In contrast, Ghafly et al. used 1-AP and 1-AP-based GUMBOS as MALDI matrices for the analysis of hydrophobic and hydrophilic peptides.^573^ By varying the counterions, they synthesized a series of solid-phase 1-AP-based GUMBOS with variable hydrophobicity, such as [1-AP]­[Cl], [1-AP]­[Asc], and [1-AP]­[NTf_2_]. Compared with the conventional matrix CHCA, the novel matrices exhibited increased hydrophobicity. There was a discernible correlation between the signal intensity of the hydrophobic peptides and the hydrophobicity of the matrix. With these matrices, the sensitivity of the detection of hydrophobic peptides markedly increased, whereas the detection of hydrophilic peptides increased in intensity in more hydrophilic matrices.

**Figure 84:**
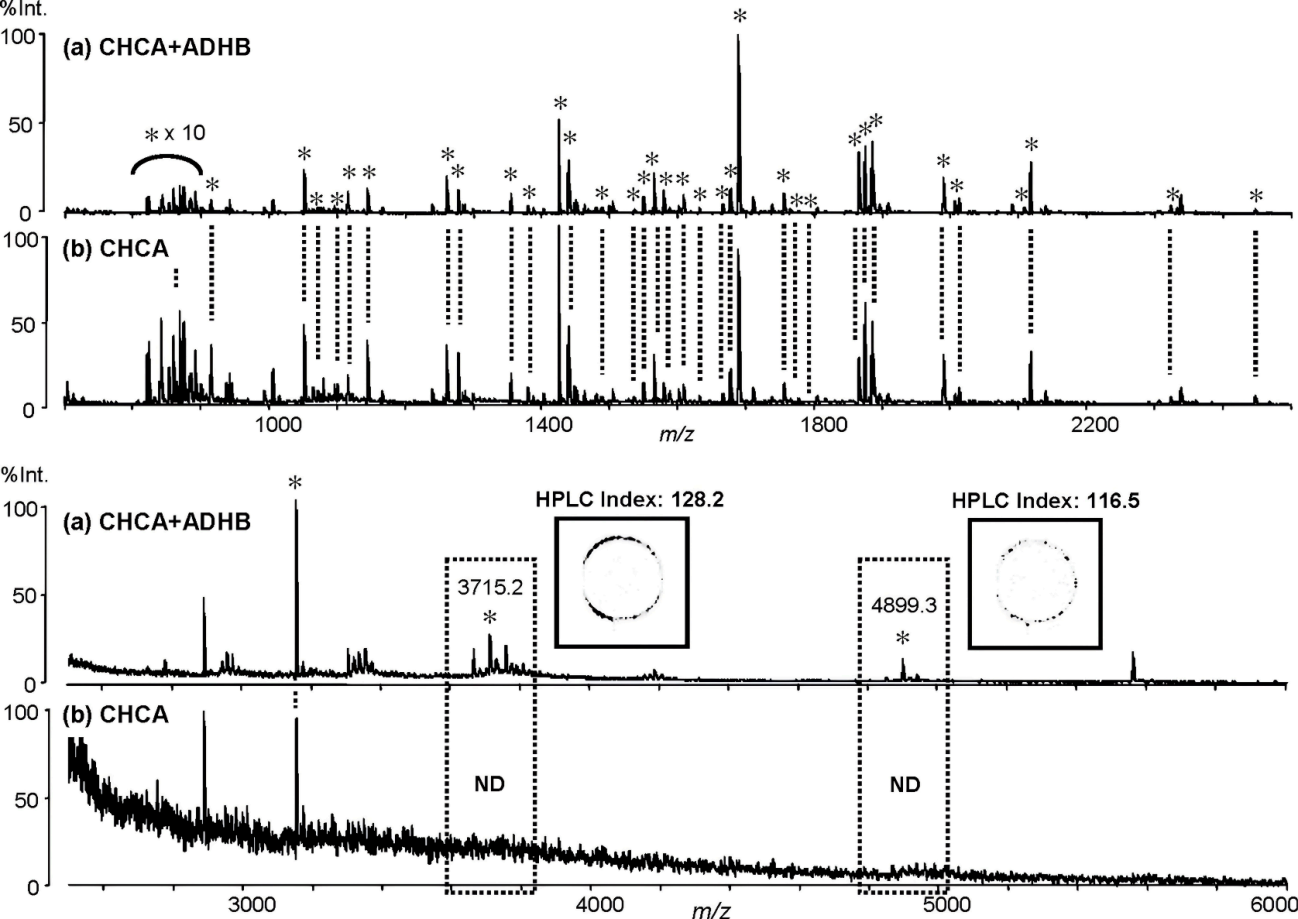
Alkylated dihydroxybenzoic acid (ADHB) as a MALDI dopant for hydrophobic peptide analysis. Positive-ion mass spectra of 100 fmol phosphorylase b digests using (a) CHCA + ADHB or (b) CHCA alone at *m*/*z* 700–2,500 (top) and *m*/*z* 2500–6,000 (bottom). The digested ion peaks are annotated with asterisks. ND indicates that ion peaks were not detected. Insets are MS images of the ion peaks at *m*/*z* 3715.2 and *m*/*z* 4899.3 obtained using CHCA + ADHB. The HPLC index is a hydrophobicity scale, where higher values indicate greater hydrophobicity. The ion at *m*/*z* 3715.2, which has a relatively high HPLC Index of 128.2, and *m*/*z* 4899.3, which has a relatively high HPLC Index of 116.5. Reproduced with permission from ref 570. Copyright 2012 American Chemical Society.

##### 4.1.2.5 Disulfide-Linked Proteins/Peptides

Disulfide bonds in proteins represent prominent post-translational modifications, whereby the sulfhydryl groups present in cysteine residues are linked to one another. These bonds play a crucial role in maintaining the structural integrity of proteins, yet they cannot be accurately predicted based on the AA sequence alone. The resolution of the location of disulfide bond formation is of critical importance for the comprehension of the 3D structure and stability of proteins, as well as for the elucidation of their structure–function relationships. However, determining the location of disulfide bonds between multiple cysteine residues has constituted a significant challenge in the structural resolution of natural proteins and peptides.

In a review of MALDI-MS for the determination of peptide and protein disulfide bonds, Yang et al. described partial reduction and alkylation methods as well as strategies for the identification of disulfide bonds and/or cysteine residues in proteins using MALDI-MS.^1039^ The majority of MALDI-MS used for the characterization of proteins containing disulfides and free cysteines involve the reduction of disulfides and the alkylation of hydrosulfide groups with a variety of reagents, including commonly used reducing agents such as dithiothreitol (DTT)^1040^ and tris­(2-carboxyethyl)­phosphine (TCEP).^1041^ The reactions of disulfide proteins/peptides with DTT (Figure 85a) and TCEP (Figure 85b) are shown in Figure 85. Furthermore, reagents such as indoacetamide,^1042^ *N*-ethylmaleimide,^1043^ and acrylamide^1044^ are frequently employed to facilitate the alkylation of cysteine. As a result, derivatized disulfides facilitate expanded MALDI-MS detection, as observed by Goman et al. The use of DHAP and DAHC in MALDI-MS circumvented the fragmentation of susceptible peptides and curtailed peptide cation endocytosis. Furthermore, it was compatible with the direct analysis of TCEP-reduced disulfide-linked peptides, which enabled expedient determination of the molecular weight of small proteins.^305^

**Figure 85:**
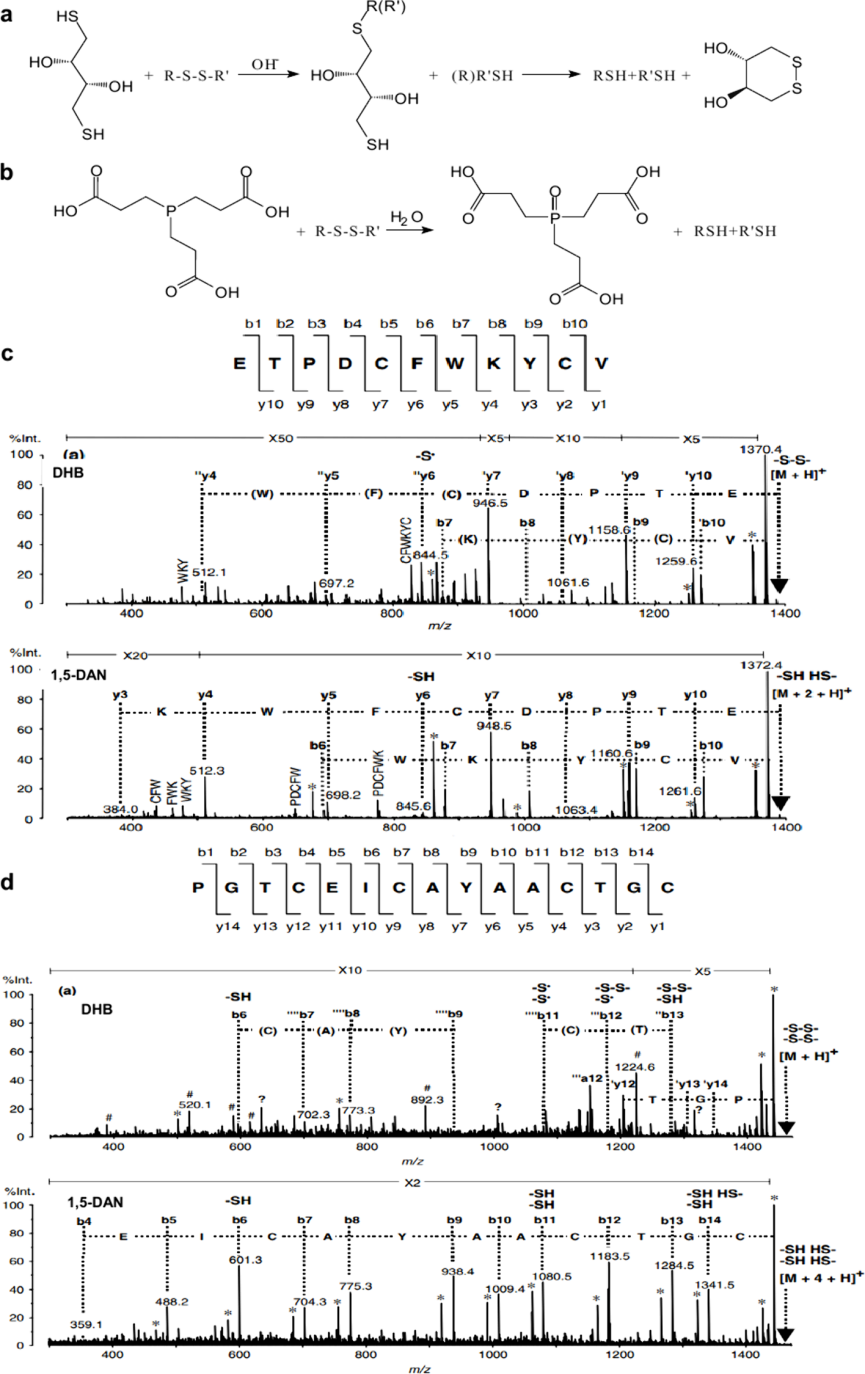
(a,b) Reactions of disulfide-containing proteins/peptides with (a) DTT and (b) TCEP. Adapted and reproduced with permission from ref 1039. Copyright 2012 Springer-Verlag. (c,d) MS/MS spectra of urotensin II (c) and guanylin (d) obtained by using DHB and DAN as matrices with AXIMA-QIT TOF MS in positive ion mode. The ion peaks indicated by (*) are derived from dehydrated ion species. The ion peaks indicated by (#) are derived from multiple internal cleavages. Superscript symbols on *y*-ions are as follows: (′), *y*-ions in which the disulfide bonds are retained; (′′), *y*-ions in which the terminus of the half-cysteine residues is ‘S·’; and other *y*-ions in which the terminus of the half-cysteine residues is ‘SH’, *i.e.*, the thiol group. (′′′), *b*-ions in which a disulfide bond is retained and the terminus of the half-cysteine residues is ‘S·’; (′′′′), *b*-ions in which a disulfide bond is cleaved and the terminus of the half-cysteine residues is ‘S·’; other *b*-ions show that all disulfide bonds are cleaved and that the terminus of each half-cysteine residues are ‘SH’, *i.e.*, a thiol group. Symbols of amino acids in parentheses mean that they are usually impossible to obtain *de novo* sequencing because a disulfide bond is retained at the base, and the terminal of half-cysteine residue is ‘S·’ even though the disulfide bond is cleaved. Adapted and reproduced with permission from ref 352. Copyright 2005 John Wiley & Sons.

In addition to reagent-assisted methods, a limited number of MALDI matrices have been reported for direct disulfide bond detection of peptides or proteins. For example, Fukuyama et al. employed AA sequencing and disulfide bond localization of human urotensin II (Figure 85c) and human guanylin (Figure 85d), which contain one and two disulfide bonds, respectively, utilizing DAN as a MALDI matrix.^352^ DAN was used in a manner analogous to that of a conventional MALDI matrix, without any prior treatment of the peptides. MS was then conducted directly using MALDI-QIT-TOF. Their outcomes demonstrated that the tandem mass spectra of the molecular ions obtained through reduction with DAN yielded a notable series of *b*-/*y*-series product ions, enabled the successful identification of AA residues and narrowed the potential candidates for disulfide bond arrangement. Moreover, Molin et al. conducted a comprehensive investigation into the reaction mechanism of DAN, elucidating the structural characteristics of the reactive species generated by DAN through the use of precise mass measurements and MS/MS experiments. DAN was found to generate the anomalous molecular ion M^+•^, which could be attributed to a two-photon pooling process. Their findings further suggested that the M^+•^ ion of DAN played a pivotal role in the protonation of the analyte and the reduction of the disulfide bond.^543^ Furthermore, Asakawa and colleagues investigated the potential of TSA as a reactive matrix for MALDI-MS.^491^ Despite the reducing properties of TSA, its weak interaction with the carboxyl oxygen in peptides rendered it incapable of inducing reductive MALDI-ISD. However, when peptides containing disulfide bonds were analyzed, the reaction of the TSA matrix resulted in partial cleavage of the disulfide bonds, yielding TSA-endocyclized peptides. Consequently, a comparison of conventional matrices and TSA in MALDI matrices allowed the number of disulfide bonds in the peptides to be calculated.

#### 4.1.3 Outlook

The advance of MALDI-MS has led to the development of a versatile, reliable, and sensitive analytical tool for protein identification in sequence databases. As the foundation of proteomics research, MALDI-MS analysis offers the benefits of high throughput and the capacity to analyze limited sample sizes (less than 1 μL). The selection of suitable organic matrices can result in the formation of homogeneous co-crystals with analytes, thereby effectively enhancing the detection of low-abundance proteins/peptides in complex samples. Furthermore, NMs are widely employed as matrices, affinity probes, and enrichment probes in proteomics analysis due to their distinctive optical and physicochemical properties, rendering them optimal candidates for the pre-enrichment of trace biomolecules prior to MALDI-MS analysis. The matrices used for protein assays are currently undergoing a period of significant evolution to facilitate the application of appropriate matrices for a range of research objects and purposes. Given the considerable advances that have been made in chemical synthesis and nanoscience in the development of MALDI-MS bioanalytical methods, more effective matrices and analytical methods will likely be developed in the future for the high-throughput proteomic characterization of biological samples.

### 4.2 MALDI Matrices for Metabolomic Analysis

In the past decade, metabolomics has played an increasingly important role in the study of functional genomics and systems biology, providing practical information on metabolic networks/pathways in biological systems. Metabolomics, one of the latest omics technologies widely used in the post-genome era, can be traced back to the study of “metabolic profiles” in the 1970s.^1045^ The study of metabolic profiles refers mainly to the initial clinical diagnosis of metabolites in patients’ body fluids based on chromatography-MS techniques.^1046^ Among them, LC-MS and GC-MS, *etc.* are considered effective tools for metabolomics analysis, with LC-MS particularly noteworthy for its combination of LC's separation capabilities and MS's detection specificity, thereby providing crucial molecular weight and structural information.^1047^ In comparison, MALDI-MS technology is prone to the presence of matrix-related background signals in the low mass range, complicating the mass spectral annotation of LMW compounds. Additionally, during the simultaneous analysis of metabolite mixtures, the dominance of a particular analyte can lead to ion suppression effects, and the low reproducibility of signal intensity often poses challenges for quantitative applications.^1048^ Consequently, the number of metabolites detected in a single MALDI-MS experiment is typically lower than that achieved by LC-MS. Nevertheless, MALDI-MS possesses several advantageous features that make it suitable for LMW compound analysis, such as high tolerance to salts and buffers, rapid analysis, high sensitivity, minimal sample consumption, and the ability to store samples on the target plate for extended periods.^1048^ Furthermore, the reliance of LC-MS on metabolite extraction from tissues inevitably leads to the loss of the *in situ* spatial distribution information, which directly affects the exploration of the biological value of tissue metabolites.^1049^ In comparison, MALDI-MSI is widely used for *in situ* detection and imaging of endogenous metabolites in biological tissues with superior detection throughput and sensitivity. In recent years, MALDI-MS has not only become the first choice for the analysis of high molecular weight compounds but also has provided a constant stream of powerful driving force for LMW compound analysis with the development of new MALDI matrices.

LMW metabolites, whose molecular weight is usually less than 1500 Da, refer mainly to a class of functional entities that participate in controlling cellular metabolism and the physiological, pathological, or developmental state of biological systems. Compared with genomics, transcriptomics, and proteomics, metabolomics can better reflect the “compound phenotype” of an organism under specific conditions and can provide more comprehensive information on the actual metabolic profile of a tested biological sample in different conditions.^1050^ Metabolites are diverse, including small peptides, carbohydrates, polar and nonpolar vitamins, organic acids, amine chelating compounds, and drug compounds. Small peptides have been introduced in proteomics. Lipidomics and glycomics are important subsets of metabolomics, and due to their significance, the application of MALDI-MS in lipidomics and glycomics will be highlighted in subsequent sections. This section mainly highlights the introduction of special or highly specific metabolites from different species and the matrices suitable for detecting these metabolites (Table S9), such as the MALDI analysis of important metabolites (*e.g.*, NTs, vitamins, alkaloids, plant hormones, and flavonoid compounds, *etc*.).

#### 4.2.1 MALDI Matrices for Untargeted Metabolomic Analysis

There is no doubt that metabolites encompass various compounds with complex properties. However, several factors limit the detection of metabolites, such as the preference of MALDI matrices for metabolite detection, mass analysers for different MALDI-MS, and the complexity/heterogeneity of the biological tissue surface and the internal environment, *etc.* ^1047^ Therefore, further efforts are needed to detect and identify a wide range of metabolites in biological tissues in the field of analytical chemistry.^1051^ Since the first generation of classic matrices was developed, MALDI-MS has shown unprecedented potential for nontargeted metabolite detection. For example, classic matrices such as DHB, CHCA, and 2-MBT were initially inclined to detect high molecular weight compounds.^394^ Novel matrices were subsequently developed based on the “core structure” of first-generation classical organic matrices, including derivatives of benzoic acid and cinnamic acid, as well as ILMs and inorganic graphene-based matrices.^566^ In fact, some scholars have stated that MALDI technology is no longer the primary choice for detecting high molecular weight compounds (although there is still interference from matrix signals in the LMW region). Duncan et al. also demonstrated that UV-MALDI based on CHCA and DHB matrices can be used for detecting LMW compounds in the positive-ion mode (quaternary ammonium salts, steroids, nucleosides, purines, pyrimidines, AAs, choline, opioids, antibiotics, prostaglandins, porphyrin macrocyclic metal complexes, and phthalocyanine IX).^1052^ However, it is clear that detecting metabolites in real tissue/biological samples is often far less accurate than detecting standard compounds. The primary factor is that the abundance of some metabolites in the tissue microenvironment is very low, and ionic inhibition effects may occur between metabolites. The background interference of the matrix itself can indirectly minimize the ionic inhibition effects by optimizing laser intensity and the molar ratio of the matrix/analyte, etc.^234^ Overall, the development of new matrices provides a valuable direction for uncovering nontargeted metabolic information.

In the various MALDI processes for nontargeted metabolite detection, matrices often exhibit a preference for polarity. Notably, the matrices utilized for negative ion mode analysis offer new perspectives for the discovery of additional metabolites. Among the various MALDI matrices, 9-AA is one of the earliest and most effective for analyzing LMW metabolites, including phenols, carboxylic acids, sulfonates, aldehydes, plant hormones, and bile acids.^1053,1054^ Calvano et al. also successfully applied the binary matrix DMAN/9-AA for the analysis of bacterial cell membrane components.^642^ Additionally, Fagerer et al. evaluated 20 MALDI matrices and reported that 9-AA demonstrated exceptional performance in detecting phosphorylated compounds. They also considered 4-amino-2-methylquinoline to be a suitable matrix when detecting AAs, organic acids, and nucleotide phosphates mixed in sufficient molar amounts.^1055^ Similarly, Krivosheina et al. identified 9-AA as a highly effective commercial MALDI matrix. They noted that compared to 9-AA, 4-dimethylaminobenzaldehyde (DMABA) is more effective for detecting LMW carboxylic compounds, substituted phenols, and naphthenic acid mixtures in the negative ion mode.^1056^ Beyond 9-AA, other matrices such as norharmane,^455^ DAN,^182^ and DMAN^425^ have also been reported for metabolite detection in the negative ion mode. Notably, DAN has been utilized for the analysis of PLs and LMW metabolites in maize leaves. Furthermore, DMAN has demonstrated excellent performance in the detection of various LMW acidic metabolites, including carboxylic acids, FAs, AAs, vitamins, and both plant and animal hormones in negative ion mode.

In recent years, to further improve the detection range of biological tissue metabolites, MALDI matrices capable of “broad spectrum” detection have been reported. Tang et al. reported the use of HZN as a universal MALDI matrix for the detection and imaging of small molecule metabolites, lipids, and proteins.^443^ Gu et al. reported that HNBN, as a universal matrix, has strong UV absorption and a clean background in the low mass range. It has demonstrated improved detection performance for different types of analytes, including organic drugs, peptides, proteins, mouse brain tissue, and bacteria.^56^ Liu et al. proposed that P2NA, as a novel matrix, facilitates the MALDI analysis and imaging of various small-molecule metabolites, including free FAs, AAs, peptides, antioxidants, and PLs, and succeeded in identifying and quantifying metabolites in the brain tissue of rats subjected to middle cerebral artery occlusion.^415^ Zhu et al. innovatively used GO as an auxiliary matrix for traditional matrices such as DHB, CHCA, THAP, and SA. The results show that the mixed binary matrix has better detection performance than the single conventional matrix does.^1057^ For example, the DHB-GO composite matrix forms uniform matrix deposition and exhibits stable point-to-point reproducibility, sensitivity, and linearity in statin dry drop analysis. The THAP-GO composite matrix is expected to be widely used in lipid research, and CHCA-GO and SA-GO have good detection effects for peptides and proteins. This study also revealed that low concentrations (0.1 mg·mL^–1^) of GO caused little contamination to the MALDI-MS. Notably, if instrument safety is a critical consideration, inorganic matrices are unsuitable for long-term, high-frequency use in MALDI-MS, as they may lead to unforeseen failures or damage to the mass spectrometer, such as contamination of the ion source or the risk of short circuits.^33^ Wang et al. made numerous original achievements in the field of new MALDI matrix research. For example, DMCA was reported as a MALDI matrix in 2019 to detect endogenous LMW compounds in biological tissue. It was able to detect more LMW compounds than conventional matrices.^33^ The same team also reported novel MALDI matrices for endogenous metabolite detection in biological tissues, such as (+) 4-NC,^420^ (+) HNTP,^394^ and (−) AAB,^185^ providing new insights for non-targeted metabolite detection in different detection modes.

Reports on these broad-spectrum matrices warrant further investigation, as they not only significantly mitigate interference from background signals associated with LMW compounds but also enable the simultaneous detection of small metabolites and large biomolecules such as proteins. This capability enhances the comprehensive coverage of high-throughput analyses of biological tissues, facilitating more integrated multi-omics studies.

#### 4.2.2 MALDI Matrices for Targeted Metabolite Analysis

##### 4.2.2.1 Neurotransmitters

NTs act as “small-molecule signal messengers” in chemical outburst transmission. They are released through the presynaptic membrane and act on the postsynaptic membrane through the synaptic gap to realize signal transmission between neurons. Common NTs include biogenic amines (DA, norepinephrine (NE), and 5-hydroxytryptamine (5-HT)), some special AAs (GABA, acetylcholine (Ach), and Gly), peptide transmitters (substance P, angiotensin, and oxytocin), and other classic NTs.^1058^ These NTs are vital in the nervous system and abnormal release or regulation often leads to physiological diseases, such as Parkinson’s disease^1059^ and AD.^1060^ At present, the methods commonly used for NT detection include electrophysiology, electrochemistry, microdialysis, and fluorescence imaging, but these techniques have several limitations. Traditional electrophysiological and electrochemical methods are only suitable for NTs^1061^ which can undergo redox reactions. Microdialysis technology can detect the concentration of NTs around the target area, but it is time-consuming and difficult to track the dynamic changes in NTs.^1062^ Fluorescence imaging methods typically have high spatio-temporal resolution, but specific antibodies or probes need to be developed for complex NTs. Therefore, the number and variety that can be detected at one time are limited.^1063^ In contrast, MALDI-MS shows unprecedented progress in NT detection because the detection only needs an appropriate matrix. However, conventional matrices have been unable to meet the needs of detection for various types of NTs, so it is necessary to further develop new matrices.

NTs have low abundance and complex chemical properties which lead to the poor performance of traditional matrices in detecting NTs. Further research on MALDI matrices revealed that several reactive matrices improved the ability to detect NTs. Interestingly, most of the organic matrix compounds that successfully detected NTs contained an “F” group or fragment. In 2014, Shariatgorji et al. introduced an in situ derivatization method utilizing pyranylium salts (2,4-diphenyl-pyranylium tetrafluoroborate, DPP-TFB), which selectively reacts with primary amines to generate *N*-alkyl or *N*-aryl pyridinium derivatives (Figure 86a). DPP-TFB derivatives of endogenous primary amines, including NTs as well as their metabolites and precursors, can undergo self-assisted laser desorption ionization. The results showed that the use of DPP-TFB can enhance the simultaneous imaging of various NTs in brain tissue, including tyrosine, tryptamine, tyramine, phenylethylamine, DA, *etc.* This method offers valuable insights into the dynamic spatial changes in NTs in specific brain structures during disease and drug response; however, it currently cannot detect the downstream products resulting from neurotransmitter deamination.^519^ In 2019, Shariatgorji’s group developed a reactive matrix based on FMP. The use of this matrix enables *in situ* spatial visualization of low-abundance NTs (such as DA, serotonin, *etc.*) and their upstream and downstream molecules in the brain with a lateral resolution of 10 μm.^165^ The FMP matrix achieves covalent charge labeling of phenolic hydroxyl and/or primary/secondary amine molecules through selective bromination (Figure 86b), successfully revealing NT biosynthesis and degradation pathways in brain tissue. It not only provides key insights into the role of NTs in the cellular location in the nervous system and complex reactions after drug administration, but is also an important reference for the study of basic neural processes and disease states. Kasai et al. found that 3-CF_3_-BTD detects moore orders of magnitude skin neurotransmitter standards (*i.e.*, DA, serotonin, histamine, and adrenaline) more effectively compared to CHCA.^322^ Additionally, the Cao team utilized DPP-TFB as a pyrylium salt for the derivatization of primary amines, resulting in significant signal enhancement for various NTs. When used in conjunction with DHB, this approach successfully detected key primary amine-containing NTs such as DA, serotonin, γ-aminobutyric acid, and histamine in crab brain sections by MALDI-MSI. Furthermore, metabolites like Ach and phosphocholine were also identified through direct application of the DHB matrix.^1064^

**Figure 86:**
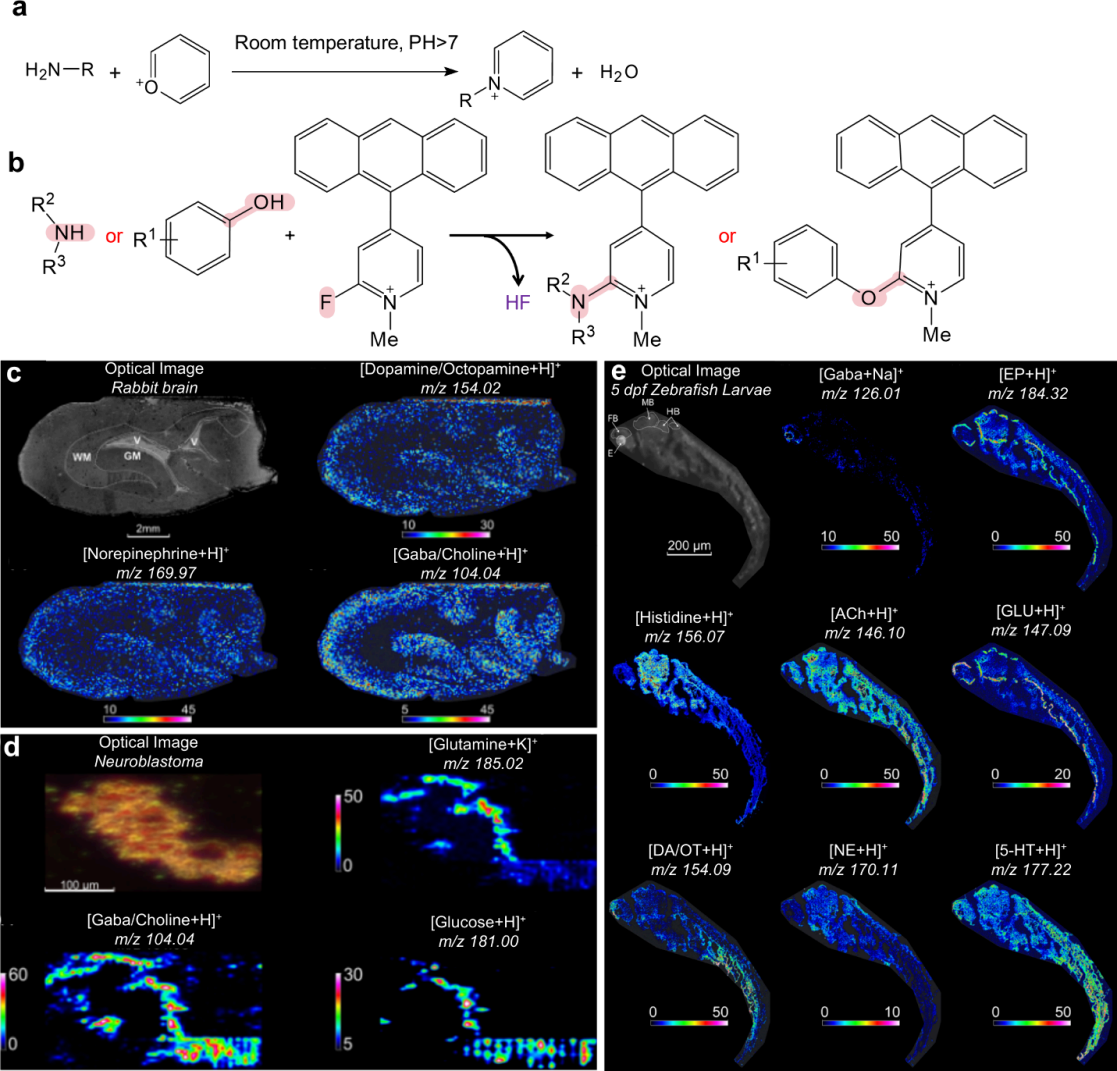
Neurotransmitters (NTs) derivatization scheme and MALDI-MSI of NTs from different biological samples. (a) Reaction of primary amines with pyrylium ion occurs at ambient temperature and pressure in 50% methanol solution at pH > 7. The reaction produces water and charged *N*-aryl or *N*-alkyl pyridinium ions that are readily detected with high sensitivity in MALDI-MS and MALDI-MSI. Adapted and reproduced with permission from ref 519. Copyright 2014 Elsevier. (b) Schematic of the reaction between the FMP reactive matrix and phenolic hydroxyls and amines. R1, R2, and R3 denote variable substituents. Adapted and reproduced with permission from ref 165. Copyright 2019 Authors, under exclusive license to Springer Nature America. (c–e) Molecular histological analysis of NTs from different biological samples by MALDI-MSI using AuNPs as the matrix. (c) MSI of a coronal rabbit brain tissue section at 20 μm lateral spatial resolution. WM, white matter; DM, gray matter; V, ventricles. (d) MSI of neuroblastoma cells at 5 μm lateral spatial resolution. (e) MSI of a sagittal zebrafish tissue section at 5 μm lateral spatial resolution. E, eye; FB, forebrain; MB, midbrain; HB, hindbrain. Adapted and reproduced with permission from ref 703. Copyright 2020 American Society for Mass Spectrometry.

To address the interference in the detection of target molecules caused by organic matrices (matrix clusters/adduction ions) in low molecular mass regions, inorganic matrices such as metal-based NPs including Au, Ag, Pt, Zn, and Ti, have been used for the detection of small molecule metabolites. Tang's team utilized solvent-free argon ion sputtering to coat animal tissue with a uniform layer of AuNPs, enabling the detection and imaging of metabolites in both normal and tumor mouse brain tissues, such as NTs and FAs.^1065^ This method offers a clean background, high sensitivity, and the added benefit of electrical conductivity, and can provide Supporting Information on tissue slices through scanning electron microscopy. McLaughlin et al. used AuNPs covered with citrate to successfully detect physiologically relevant concentrations of Ach, DA, epinephrine, Gln, 4-aminobutyric acid, and NE in serum and homogenate tissue through MALDI-MS.^703^ In addition, they successfully pneumatically sprayed AuNPs onto animal tissue slices, achieving imaging of rabbit brain tissue slices with a lateral spatial resolution of 20 μm (Figure 86c) and imaging of several precursor amine NTs in neuroblastoma cells (Figure 86d) and zebrafish embryos (Figure 86e) with a lateral spatial resolution of 5 μm. This method provides a time- and cost-effective preparation approach, significantly lower in cost and more efficient compared to traditional chemical derivatization strategies and organic matrices.^703^ Similarly, Chen et al. compared the newly developed ZnO NPs with TiO_2_ NPs. They found that the use of ZnO NPs was able to obtain high-quality images of mouse sagittal and rat coronal tissue sections, but there was no significant difference in the number of detected LMW compounds compared to TiO_2_ NPs.^1066^ The partially reactive matrix developed by researchers, as well as the inorganic nanomatrix, provides entirely new insights into the rapid and efficient detection of NTs. These findings help neuroscientists to further elucidate neural processes and disease mechanisms by studying the changes in the abundance and spatial distribution of these NTs in the brain.

##### 4.2.2.2 Vitamin

Vitamins are essential nutrients that animals generally cannot synthesize on their own, requiring dietary intake to prevent health issues. Therefore, a lack of vitamins in the human body usually leads to health problems.^1067^ Vitamins are classified into two categories: water-soluble and fat-soluble vitamins. Water-soluble vitamins dissolve in water but not in nonpolar solvents, with limited storage in the body and the excess excreted in the urine. In contrast, fat-soluble vitamins dissolve in nonpolar solvents, can be absorbed with dietary fats, and are stored in the body with lower excretion rates.^1068^ Such vitamins play crucial roles as enzyme cofactors in various biochemical reactions, contributing to the overall homeostasis of the organism.

Shikano et al. used CHCA matrix-based MADLI-MSI to analyze vitamins A_1_, B_6_, and C in dried persimmons (*Diospyros kaki*) and revealed the influence of drying on the distribution and content of various vitamins in persimmons. The results, as shown in Figure 87a–k, indicate that lipophilic vitamin A_1_ was distributed mainly in the dry outer peel, whose content was 3.4 times higher than that of fresh persimmons. Moreover, vitamin B_1_ and B_6_ were concentrated in the moist middle peel, and the drying process reduced the content of vitamin C in persimmons.^1069^ While few studies have reported the use of classical matrices for vitamin analysis, novel organic, ionic liquid, and inorganic matrices have been thoroughly explored for the same purpose. Chen et al. evaluated 17 porphyrin compounds as MALDI-TOF MS matrices for detecting water-soluble vitamins B_1_, B_2_, B_6_, B_12_, and C, and reported that porphyrin matrices with hydroxyl or carboxyl groups are effective for this purpose.^1070^ This study revealed that vitamins B_2_ and B_6_ can be ionized under 337 nm laser irradiation with the help of these matrices, and that a higher molecular weight of vitamins requires an increased optimal matrix/analyte molar ratio (M/A). To address the uneven distribution of solid matrices, researchers have investigated ILMs for vitamin detection in MALDI-MS, finding that some ILMs, which combine classical MALDI matrices with organic bases, effectively avoid the sweet spot effect and are suitable for LMW compounds such as vitamins.^583^ Additionally, Chen et al. designed and synthesized a novel compound, DHPT, which serves as an efficient MALDI matrix for analyzing various LMW amines.^325^ Given that all vitamin B compounds are amines, DHPT has also been reported for the detection of vitamin B compounds. Figure 87l illustrates the MALDI-TOF mass spectra of these vitamin B compounds, each analyzed at a concentration of 2.5 nmol: thiamine (VB_1_), riboflavin (VB_2_), nicotinamide (VB_3_), and pyridoxol (VB_6_). The results indicate that DHPT offers several advantages for mass spectrometric analysis in the low mass range, including high sensitivity, excellent selectivity, broad applicability, and minimal background interference.

**Figure 87:**
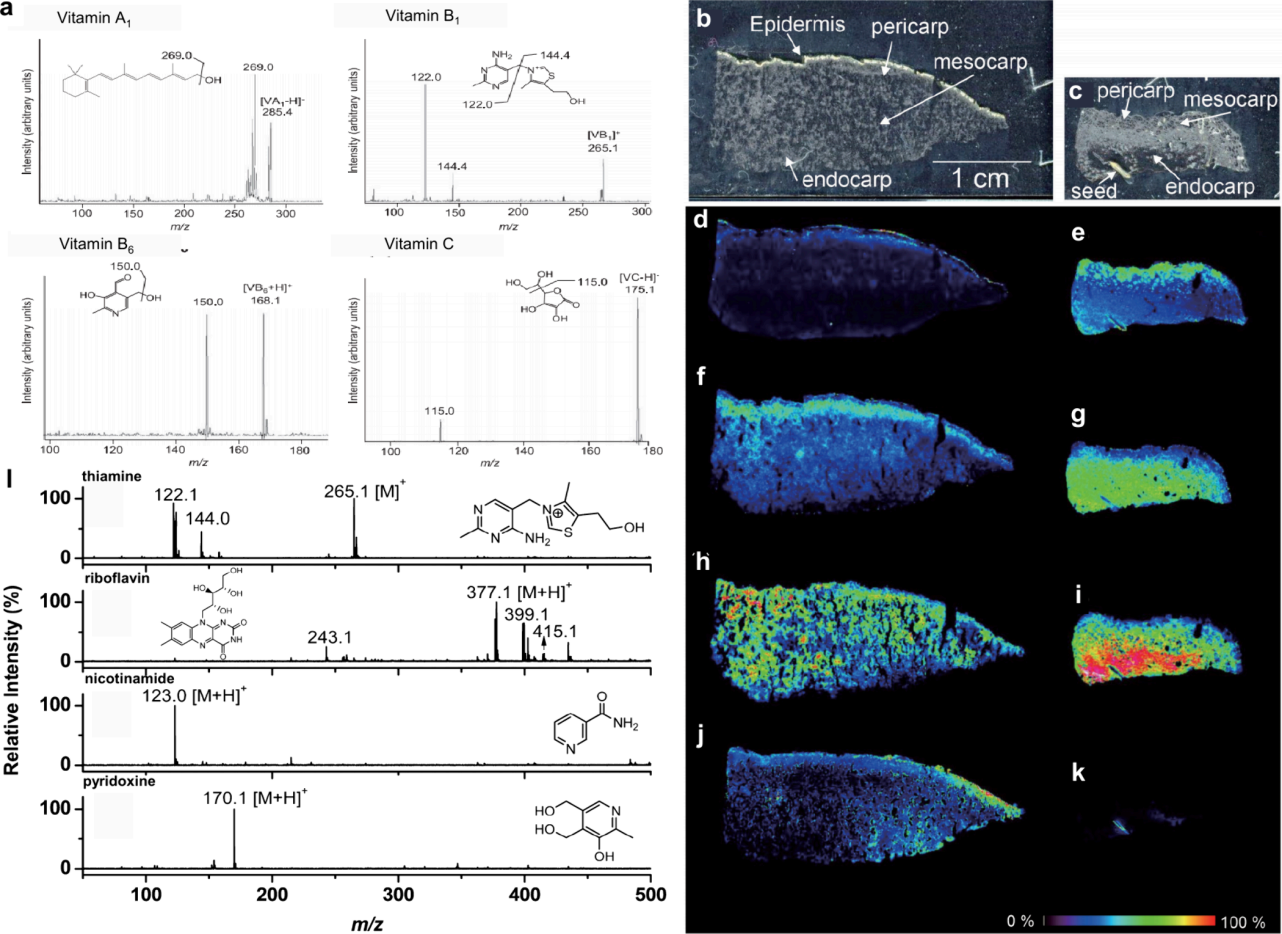
(a) MALDI tandem mass spectra of vitamin-related analytes. Structures of analytes and their fragmentation patterns: vitamin A_1_, vitamin B_1_, vitamin B_6_, and vitamin C. The spectra of vitamin A_1_, vitamin B_1_ and vitamin B_6_ are from dried persimmon cross-sections, whereas the spectra of vitamin C are from raw persimmon cross-sections. (b–k) MALDI-MS imaging of vitamins in persimmon sections. Optical images of (b) raw and (c) dried persimmon sections. MS image of vitamin A_1_ in (d) raw and (e) dried samples, vitamin B_1_ in (f) raw and (g) dried samples, vitamin B_6_ in (h) raw and (i) dried samples, and vitamin C in (j) raw and (k) dried samples. The vitamin related signals from the cross sections were normalized to the total ion count. Adapted and reproduced with permission from ref 1069. Copyright 2020 Japan Oil Chemists’ Society. (l) MALDI-TOF mass spectra for vitamin B compounds with DHPT as the matrix: thiamine (VB_1_) *m*/*z* 265.1, [M]^+^; *m*/*z* 122.1 and 144.0 corresponds to the fragmentation products of the C–N bond between the ethyl and nitrogen cations; riboflavin (VB_2_) *m*/*z* 377.1, [M+H]^+^; *m*/*z* 399.1, [M+Na]^+^; *m*/*z* 415.1, [M+K]^+^; *m/z* 243.1 corresponds to the loss of one 2,3,4,5-tetrahydroxyl-1-pentene molecule from [M+H]^+^; nicotinamide (VB_3_) *m/z* 123.0, [M+H]^+^; pyridoxine (VB_6_) *m*/*z* 170.1, [M+H]^+^. The amount of each compound was 2.5 nmol. Adapted and reproduced with permission from ref 325. Copyright 2012 American Chemical Society.

Vitamin D is a class of lipophilic steroid compounds, including vitamin D_3_ in mammals and vitamin D_2_ in plants (especially mushrooms). Vitamin D_3_ is synthesized mainly in skin exposed to UV light, and is crucial for bone metabolism. Deficiencies in vitamin D can lead to diabetes, cancer, depression, neurodegenerative diseases, and cardiovascular diseases.^1071^ In the analysis of vitamin D, 25-hydroxyvitamin D_3_ (25­(OH)­D_3_) is often used as an indicator due to its longer half-life, relatively higher blood concentration, and direct correlation with vitamin D levels. Qi et al. determined 25­(OH)­D_3_ in human serum using chemical derivatization^1072^ and MALDI-MS (CHCA as the matrix) as rapid quantitative methods,^1073^ and compared the results with those of LC-MS/MS. They reported that this approach not only improved accuracy while circumventing matrix effects but also offered several advantages over LC-MS/MS, including the elimination of time-consuming chromatographic separation, enhanced speed, reduced sample consumption, the absence of organic solvents, and greater ease of automation. Additionally, Noh et al. used a parylene matrix chip for MALDI-TOF MS analysis of vitamin D with low chip noise and uniform matrix distribution, which is suitable for the quantitative analysis of 25­(OH)­D_3_. 25­(OH)­D_3_ was modified by the nucleophilic addition of betaine aldehyde (BA) to form a hemiacetal salt, allowing it to be detected by MALDI-TOF MS.^1074^

At present, dozens of different vitamin compounds have been reported, and most of these vitamins cannot be detected by conventional means. The advancement of derivatization techniques and new matrices has improved vitamin detection, and future efforts should focus on expanding methods for commonly studied vitamins based on existing research.

##### 4.2.2.3 Alkaloids

Alkaloids are a class of nitrogenous basic organic compounds that are usually found in certain parts or organs of plants. They have complex ring structures and important biological activities, making them among the effective components in medicinal plants.^1075^ Alkaloids are one of the secondary metabolites of plants and over 10,000 kinds of alkaloids have been identified. Due to their large number, complex structures, and widespread sources, no detailed classification of alkaloids is provided here.^1076^

Despite the diversity of alkaloids, many studies have been conducted using both classical and novel organic matrices. Cai et al. assessed the ability of various matrices, including SA, THAP, 3-AQ, 3-HPA, CHCA, and DHB, to detect alkaloids from Chinese medicine *Aconitum carmichaelii* Debx. tissue sections using MALDI-MSI, finding that DHB and CHCA exhibited the highest detection performance.^1077^ Dos Santos et al. analyzed the distribution of alkaloids on the surface of *Erythroxylum coca* leaves using MALDI­(+)-FT-ICR MSI technology with CHCA, 2-MBT, and DHB matrices. Among them, DHB was ultimately selected as the optimal matrix and was used in subsequent experiments at a concentration of 2 mg·mL^–1^.^1078^ Additionally, Ha et al. indicated that the sensitivity of the DHB matrix was superior to that of THAP. They successfully detected and differentiated the relative concentrations of α-solanine, α-chaconine, dehydrochaconine, and dehydrobatatinine in potato tubers and visualized the relative concentrations of sugar alkaloids accumulated through exposure during potato tuber storage using DHB matrix-based MALDI-MSI.^1079^

In addition to classical matrices for the detection of various alkaloids, many organic MALDI matrices with novel structures have been reported. Among them, compounds with “thiophene” structures are particularly favored. Schinkovitz et al. reported a synthetic matrix MT3P, which has a core of aromatic bithiophene and an introduced methylthio group. It exhibited good spectral performance in the detection of 25 alkaloids, in which 23 standard compounds were successfully detected.^324^ The bithiophene core moiety of MT3P suggests strong absorption at a laser wavelength of 337 nm. The introduction of two methylthio groups into the vicinity of the bithiophene formed 5,5′-bis­(methylthio)-[2,2′]­bithiophene (MetS2BT), which significantly enhanced the ionization ability of the alkaloids. By replacing one of the methylthio groups in MetS2BT with a 2-cyanoethyl group, the final structure of MT3P was obtained. The introduction of these moieties further enhanced the ionization ability and selectivity of MT3P for alkaloids and resulted in high-quality MALDI spectra at low laser energy levels.^324^ In 2017, the team further compared four novel bithiophene-based matrix molecules with conventional matrices CHCA and DHB and found that 3-(5′-pentafluorophenylmethylsulfanyl-[2,2′]­bithiophenyl-5-ylsulfanyl)­propionitrile (PFPT3P) showed the most promising performance. Further results revealed that the use of PFPT3P matrix provided a direct and rapid method for detecting trace alkaloids in highly complex samples such as crude extracts of colchicine, herbs containing smilagenine and berberine, and human plasma containing strychnine.^1080^ Moreover, DHPT demonstrated excellent selectivity for amine analysis with minimal matrix interference and enabled low picomolar to femtomolar detection limits in positive ion mode, along with the selective detection of standard alkaloids.^325^

Relevant literature reports on alkaloids are more concentrated in botanical research. MMA has been reported by Feng and Lu as a matrix for the MALDI detection and imaging of arecoline and arecaidine in areca nut (Figure 88a), while also demonstrating its feasibility for trace analysis of arecoline in human plasma at concentrations below 3 μM.^386^ The Wu’s team conducted MALDI-MSI analysis of the principal alkaloids in areca fruit using a novel organic matrix, DMCA. They performed *in situ* imaging of 10 alkaloid types across three developmental stages of areca fruit (*Areca catechu*), including arecoline, arecaidine, caffeine, cotinine, guvacine, guvacoline, hordenine, sophoridine, trigonelline, and vicine. The ion images obtained by MALDI-MSI detailed the spatial distribution of alkaloids in different tissues of the areca fruit (*i.e.*, exocarp, mesocarp, endocarp, exotesta, endosperm, and embryo) and the identity and abundance of alkaloids were further confirmed by LC-MS/MS.^1081^ Additionally, several studies have reported the use of inorganic nanomatrices for the detection of alkaloids. Ewelina, P. et al. employed functionalized TiO_2_ nanowire deposition to visualize vinca alkaloids in the petals of *Catharanthus roseus* using MALDI-MS.^738^ As illustrated in Figure 88b, the spatial distribution of several vinca alkaloids within the deep pink and white petals of *Catharanthus roseus* catharanthine, catharanthine, vindolinine isomers, serpentine, vindoline, and anhydrovinblastine. The similarity in the relative quantities of vinca alkaloids obtained through SALDI-MS^2^ and standard LC-QQQ-MS analyses validated the accuracy of the imprinting method for semi-quantitative purposes. Furthermore, the study explored the differences in the vinca alkaloid profiles among five varieties of *C. roseus*, presenting a semi-quantitative comparison of all the discussed vinca alkaloids across different varieties. In that study, TiO_2_ nanowires were introduced as a solid matrix for MSI of LMW compounds in plant tissues, significantly enhancing the detection limits by modifying the chemical properties of the matrix surface to improve sample imprinting selectivity. Yang’s team successfully utilized AuNPs as a matrix for MALDI-TOF MSI to map the distribution of AAs and various alkaloids in different parts of *Lepidium meyenii* Walp root, offering valuable insights for *in situ* investigations of active ingredients in natural products. Their results identified several imidazole alkaloids produced during the drying process and enabled clear visualization of AAs, amide alkaloids, and saccharides throughout different sections of the root tissue.^1082^ In conclusion, the detection of various alkaloids by different MALDI matrices has essentially been realized, and reports of new organic matrices have further promoted the study of different types of alkaloids.

**Figure 88:**
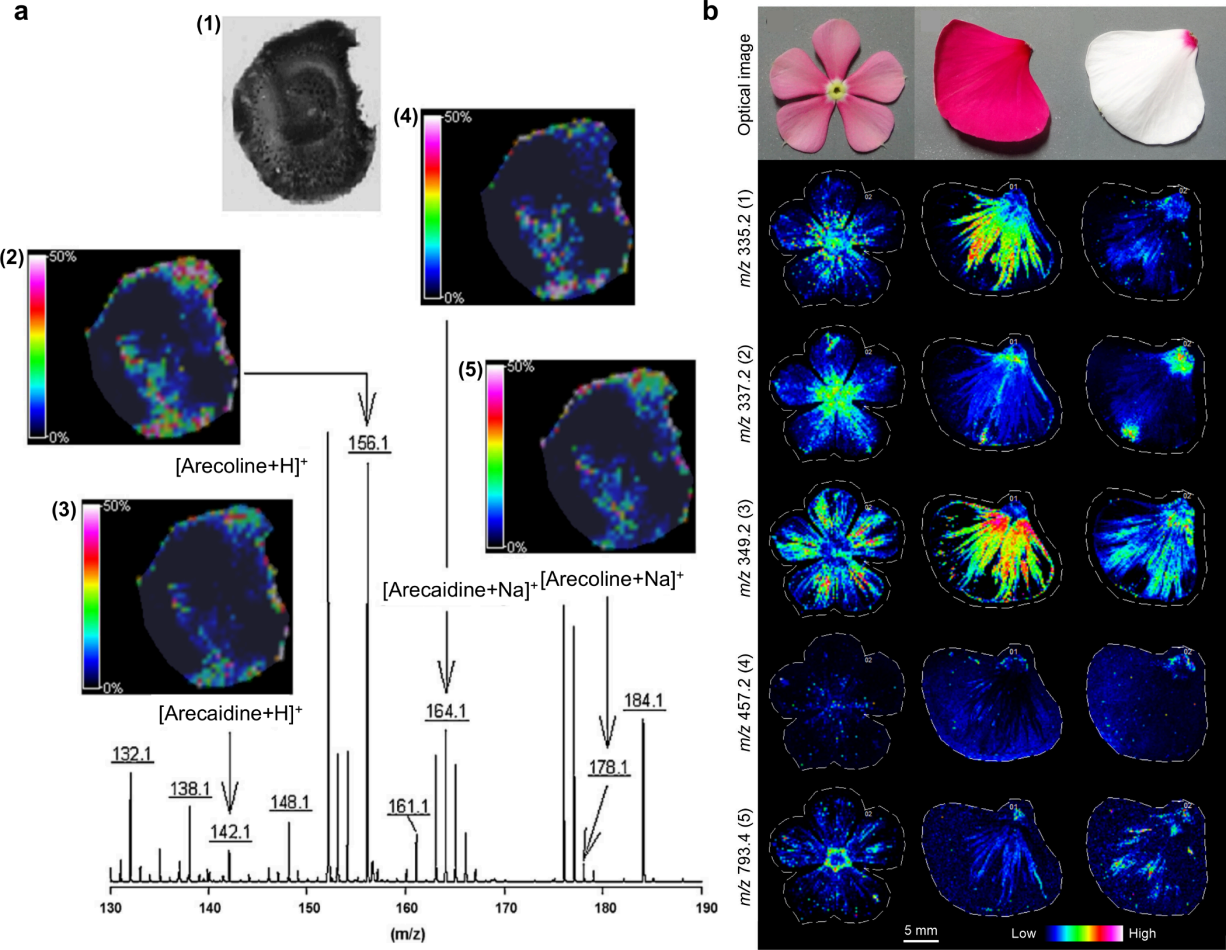
(a) The MALDI-TOF mass signals were transformed into images. The distribution of arecoline and arecaidine in fresh areca nut section was tested by MALDI imaging: (1) image of fresh nut tissue, (2) [M+H]^+^ of arecoline, (3) [M+H]^+^ of arecaidine, (4) [M+H]^+^ of arecaidine, and (5) [M+H]^+^ of arecoline. Rainbow colors represent the different intensities of arecoline and arecaidine in the tissue. Adapted and reproduced with permission from ref 386. Copyright 2009 Elsevier BV. (b) Spatial distribution of vinca alkaloids in *Catharanthus roseus* petals. Distribution of alkaloids in intact small flowers, single dark pink petals and single white petals of a larger flower. Signals at (1) *m*/*z* 335.2, (2) 337.2, (3) 349.2, (4) 457.2, and (5) 793.4 correspond to (1) iminium catharanthine, (2) catharanthine and vindolinine isomers, (3) serpentine, (4) vindoline, and (5) anhydrovinblastine, respectively. Petals were imprinted on a TiO_2_ plate. Raster step was 250 μm. The color bar represents the MS signal intensity. Adapted and reproduced with permission from ref 738. Copyright 2020 Society for Experimental Biology and John Wiley & Sons.

##### 4.2.2.4 Phytohormones

Plant hormones are trace small molecule metabolites in plants, with key roles in physiological processes such as plant growth, development, and stress. They mediate signal transmission that affects gene expression in response to light, temperature, water and other conditions, so that plants can adapt to changes in the external environment.^1083^ Plant hormones can be divided into endogenous and exogenous compounds. Endogenous hormones, such as auxin, gibberellin (GA), cytokinin (CK), abscisic acid (ABA), ethylene (ETH) and brassinosteroid (BR), are involved in biological processes such as the growth, division, differentiation, and dormancy shedding of plant reproductive organs through interconnected response networks.^1084^ Exogenous hormones, such as naphthalene acetic acid (NAA), buxin, ethethylene, *etc.*, are artificially synthesized compounds used to regulate endogenous hormone levels in plants, and are therefore known as plant growth regulators.^1085^ The amounts of plant hormones secreted in the plant are low, and these compounds usually need to be transported to other parts of the plant to play a regulatory role. Therefore, the *in situ* visualization of plant hormones is highly important for understanding the dynamic changes in their spatial location. Currently, techniques such as immunohistochemistry and fluorescence microscopy are commonly used to visualize plant hormones, but these methods are limited by the lack of specific antibodies^1086^ and the inability to visualize multiple hormones^1087^ simultaneously. Therefore, the search for more efficient and specific visualization techniques is crucial for studying plant hormones.

It is encouraging that MALDI-MSI can facilitate the *in situ* visualization of various plant hormones with an appropriate MALDI matrix. Several studies have indicated that conventional organic matrices, such as DHB, 2-MBT, CHCA, and DMAN, as well as certain inorganic nanomatrices, have been utilized to investigate a limited number of plant hormones.^52,1088,1089^ In 2017, Shiono et al. employed MALDI-TOF-MSI technology with CHCA as the matrix to conduct imaging analyses of two plant hormones, CK (specifically *trans*-zeatin (*t*Z)) and ABA, in rice (*Oryza sativa*) root tissues.^1088^ The choice of *t*Z and ABA for this study was informed by their well-established functions and distributions in plants, as well as the scarcity of similar compounds in nature. The results revealed that *t*Z is predominantly localized approximately 40 mm behind the root tip, whereas it is nearly undetectable near the root apex; in contrast, ABA was primarily detected at the root tip. To mitigate the interference caused by the self-ionization of organic matrices on the ionization of plant hormones, Shiono’s team further investigated the potential of using Fe-NPs as an inorganic matrix in MALDI-MS for multi-hormone imaging and the study of plant hormone signaling.^1090^ For comparison, the researchers prepared seven plant hormones [ABA, 3-indoleacetic acid (IAA), BR, two CKs (*t*Z and 6-(γ,γ-dimethylallyl amino)­purine (iP)), jasmonic acid (JA), and salicylic acid], an ethylene precursor (1-aminocyclopropane-1-carboxylic acid (ACC), and a heavy hydrogen-labeled ABA (D_6_-ABA)]. This inorganic nanoparticle-based MALDI-MS technology successfully identified all nine compounds, including various hormones and ACC, in rice root cross sections after a 2-hour incubation in a hormone mixture, whereas conventional MALDI-MS detected all the compounds except for IAA, BR, and D_6_-ABA. The imaging results revealed distinct distribution patterns of these hormones, with ABA and CKs located in the outer root region and IAA found in the epidermis, cortex, and stele, highlighting the method's potential for exploring hormone signaling in crop development and stress responses.^1090^

Zhang et al. reported a new matrix PNA with heightened ionization efficiency in the MALDI-MSI analysis of plant hormones and lipid molecules.^418^ Compared with traditional matrices (DHB, CHCA and 9-AA), PNA has strong UV absorption and uniform co-crystallization, and it achieved MALDI-MSI analysis of eight plant hormones (*i.e.*, ABA, IAA, *t*Z, gibberellin A_3_ (GA_3_), 2,4-dichlorophenoxyacetic acid (2,4-D), 6-benzylaminopurine (6-BA), SA, and NAA) and lipids such as LPCs, PCs and TAGs. Recently, Chen et al. introduced a novel organic matrix, DHNBA, which significantly improved the detection and imaging of multiple hormones in plant tissues.^184^ DHNBA's strong UV absorption, uniform matrix deposition, negligible background interference, and high ionization efficiency enhance the sensitivity of various standard compounds, including isoprenoid cytokinins (*t*Z, dihy-drozeatin (DHZ), meta-topolin (*m*T), and iP), JA, ABA, and ACC. Additionally, researchers have employed DHNBA in MALDI-MSI to analyze compounds (such as *t*Z, DHZ, ABA, IAA, and ACC) in various plant tissues, for example germinating seeds, primary/lateral roots, and nodules, thereby demonstrating its effectiveness in tracking complex plant hormone biosynthesis pathways. As shown in Figure 89, the researchers employed DHNBA in MALDI-MSI to analyze germinating seeds (Figure 89a), primary roots (Figure 89b), primary and lateral roots (Figure 89c), and root nodules (Figure 89d) in soybean, revealing the distribution of endogenous plant hormones across different tissue sections through *in situ* detection and imaging. These findings demonstrate that the DHNBA matrix significantly improves the ability to simultaneously monitor complex biosynthetic pathways of these hormones.

**Figure 89:**
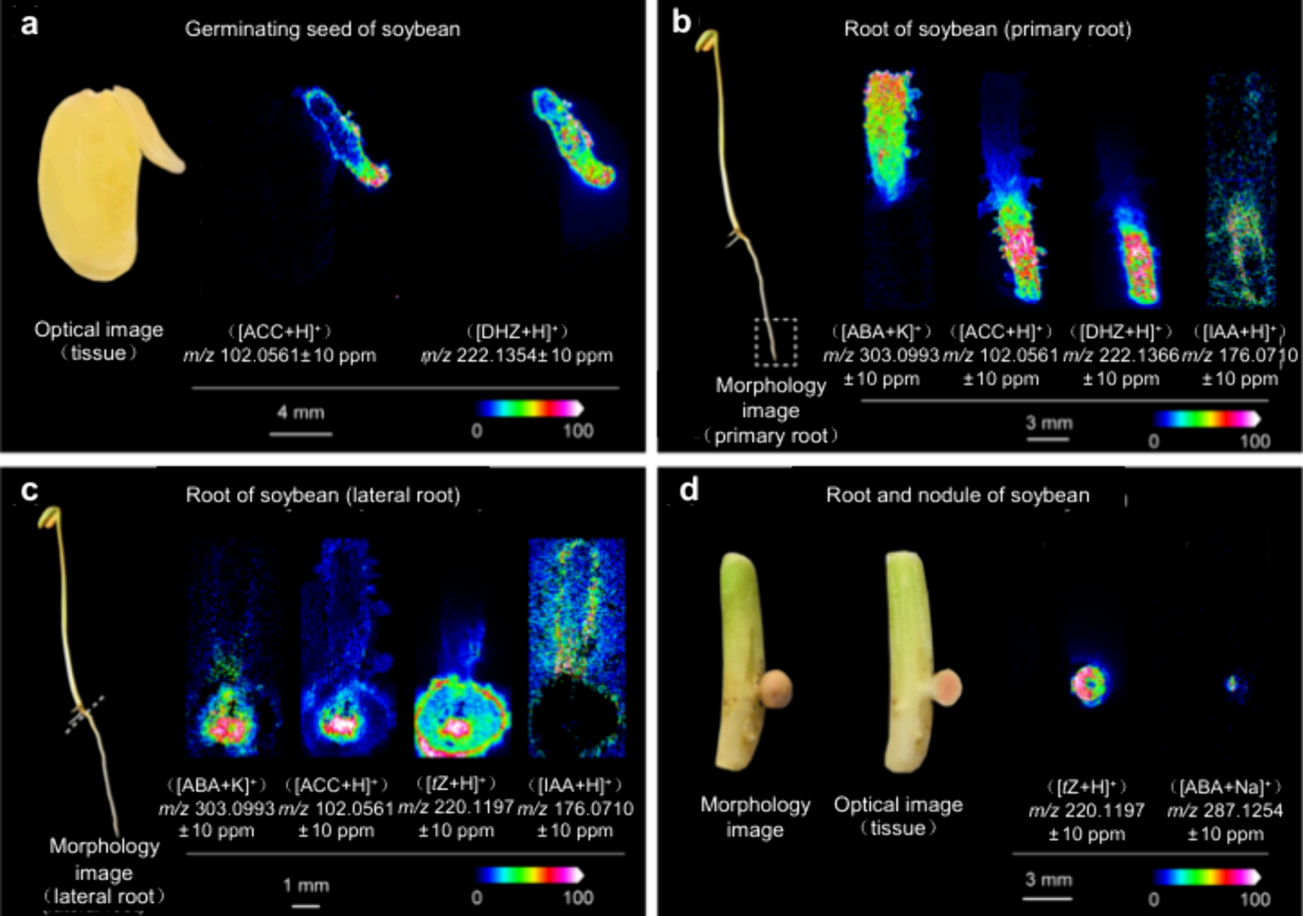
*In situ* detection and imaging of endogenous phytohormones from different soybean tissue sections by timsTOF flex MALDI-2 using 2,4-dihydroxy5-nitrobenzoic acid (DHNBA) as the matrix. (a) Reconstructed ion maps of 1-aminocyclo-propane-1-carboxylic acid (ACC) and dihydrozeatin (DHZ) in soybean germinating seed tissue sections indicate that these phytohormones are distributed in the radicle. MS imaging was acquired at 100 μm spatial resolution. (b) Reconstructed ion maps of abscisic acid (ABA), ACC, DHZ, and indole-3-acetic acid (IAA) in soybean primary root longitudinal sections reveal distinct phytohormone distribution patterns. ACC, DHZ, and IAA are distributed in the root tip, whereas ABA is predominantly distributed in the elongation zone of primary roots. MS imaging was acquired at a 50 μm spatial resolution. (c) Reconstructed ion maps of ABA, ACC, *trans*-zeatin (*t*Z), and IAA in soybean primary and lateral root sections reveal distinct phytohormone distribution patterns. MS imaging was acquired at a 50 μm spatial resolution. (d) Reconstructed ion maps of ABA and *t*Z in soybean root and nodule tissue sections. ABA and *t*Z are distributed in the nodules. MS imaging was acquired at an 80 μm spatial resolution. Reproduced with permission from ref 184. Copyright 2024 The Authors, under exclusive license to New Phytologist.

The limited sensitivity and comprehensiveness of MALDI-MS analysis for common plant hormones may be attributed to low hormone concentrations or suboptimal matrix selection.^184^ Furthermore, it is worth noting that not all phytohormones can be detected by high-vacuum mass spectrometry, as phytohormones can exert their effects at trace levels but are unstable and sensitive to extreme environmental conditions, such as temperature, humidity, and light.^1091^ In general, the development of new MALDI matrices has significantly expanded the *in situ* visualization and comprehensive study of plant hormones, facilitating improved interpretation of their spatial distribution and abundance.

##### 4.2.2.5 Flavonoids

Flavonoids are polyphenolic secondary metabolites characterized by a carbon skeleton of C6–C3–C6, consisting of two aromatic rings (A and B) and one heterocyclic ring (C). The general structures of the flavonoids are illustrated in Figure 90a. Based on the oxidation level of the C ring and differences in substitution positions, flavonoids can be divided into flavonols, flavanols, flavones, dihydrochalcones, isoflavonoids, anthocyanins, and biflavonoids.^1092^ Flavonoids have a variety of biological activities, such as antioxidant activity and some potential antiviral properties.^1093^ Moreover, as one of the representative secondary metabolites of plants, flavonoids play important roles in the growth of plant bodies and resistance to adverse external environments.^1094^ Interestingly, in addition to being a particularly rare biologically active component of plants, some structurally similar compounds to flavonoids are used as MALDI matrices because they contain multiple hydroxyl radicals. For example, polyhydroxyflavone compounds such as quercetin have been reported as excellent MALDI matrices for lipid analysis in dual-polarity mode.^51^ Moreover, Zhao et al. used N-doped luteolin-based carbon dots (N-CDs) as a matrix to analyze LMW compounds, and the results showed that the N-CDs matrix had lower background interference, higher sensitivity, higher efficiency, and better reproducibility compared with N-free CDs.^1095^

**Figure 90:**
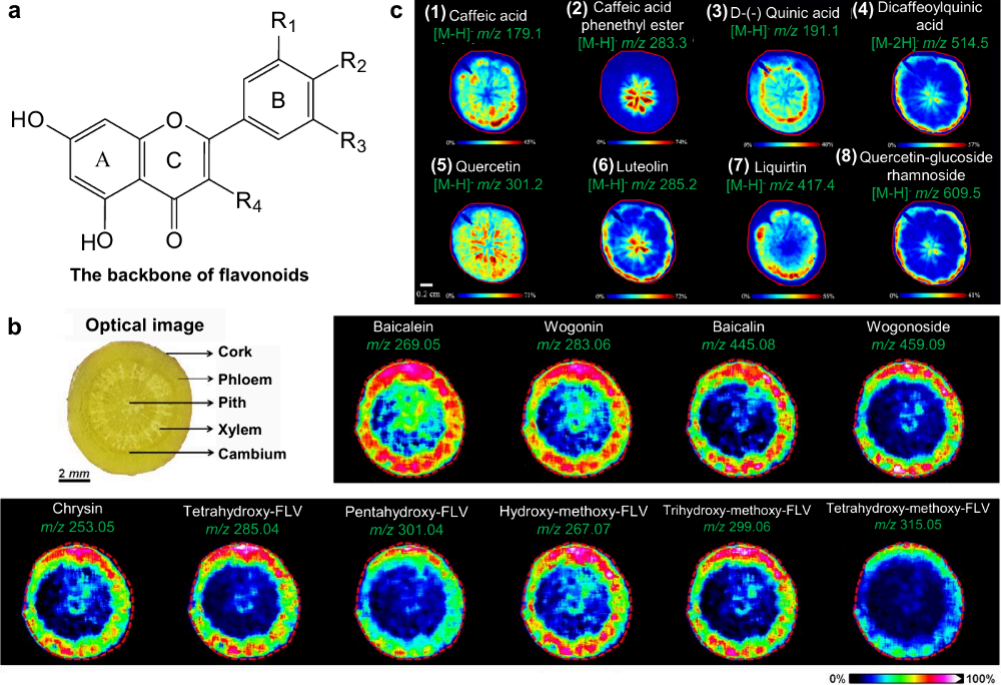
(a) General structures of flavonoids. Kaempferol: R_1_=R_3_=H, R_2_=R_4_=OH. Quercetin: R_1_=R_2_=R_4_=OH, R_3_=H. Myricetin: R_1_=R_2_=R_3_=R_4_=OH. Isorhamnetin: R_1_=OCH_3_, R_2_=R_4_=OH, R_3_=H. Luteolin: R_1_=R_2_=OH, R_3_=R_4_=H. (b) Optical image of the root section of *S. baicalensis* and MALDI-MS images of representative flavones and flavone glycosides in the root of *S. baicalensis*. Reproduced with permission from ref 1100. Copyright 2020 Elsevier BV. (c) MALDI MS images of caffeoylquinic acids (1–4) and flavonoids (5–8) in *A. lappa* roots. Data were collected from the *Baiji* variety in negative ion reflector mode. Adapted and reproduced with permission from ref 1101. Copyright 2022 The Authors, under exclusive license to MDPI, Basel, Switzerland.

Some classic matrices such as DHB, 9-AA, and THAP have also been used for the detection of flavonoids.^1096^ Madeira et al. studied the fragmentation behavior of quercetin, myricetin, kaempferol, and naringenin in MALDI using DHB as the matrix. The results showed that these flavonoids generated large flavonoid-DHB agglomerated ions (protonated, sodium- and potassium-charged clusters) in MALDI-MS.^1097^ Wang et al. employed MALDI-TOF MS with THAP and DHB matrices to identify isoflavones in soybean samples, ultimately favoring DHB for its reproducibility. Their findings indicated that isoflavones predominantly existed in the protonated form, with very little sodium and potassium complex ions present.^1098^ This adduct pattern suggests that, for these specific analytes under the experimental conditions, proton transfer was the thermodynamically favored pathway over alkali metal cation adduction. This team subsequently chose THAP in dual-polarity mode for MALDI-MSI detection of flavonol glycosides in crude food extracts.^1099^ Sun et al. detected flavonoids and flavonol glycosides in the roots of *Scutellaria baicalensis* using various conventional matrices (*i.e.*, NEDC, BNDM, DAN, and 9-AA) in negative ion mode, finding that 9-AA yielded the highest ion intensity and the optimal matrix.^1100^ A total of 11 flavones, 5 flavone glycosides, 6 carbohydrates, and various flavone synthesis-related metabolites were imaged and identified, primarily in the root phloem, where these metabolites exhibited stronger ion intensities. Moreover, using an optimized MALDI matrix, the research team conducted a visual analysis of the spatial distribution of flavonoids, flavonoid glycosides, and other metabolites in *Scutellaria baicalensis* roots. Figure 90b showed the optical image of *Scutellaria baicalensis* root slices and the distribution and relative abundance of flavonoids and their glycosides in the cross-section of the root. Notably, MALDI-MSI results revealed that the concentrations of flavonoids and flavonoid glycosides in the phloem of *S. baicalensis* roots were significantly higher than those in the xylem and pith, underscoring their crucial role in the plant's defense response to environmental stress. Li et al. conducted an *in situ* visualization of various endogenous components in *Arctium lappa* root using MALDI-TOF-MSI technology.^1101^ The results demonstrate that DHB serves as the optimal matrix for the analysis of AAs and sugars in positive ion mode, while DHAP is as the best matrix for the analysis of flavonoids and caffeoylquinic acids in *astragalus* roots. Imaging results (Figure 90c) showed a clear spatial distribution of these compounds, *e.g.*, caffeoylquinic acid and flavonoids predominantly in the epidermis and cortex.^1101^

In addition to organic matrices, researchers have used inorganic materials as matrices to carry out studies on flavonoids. For example, Yin et al. prepared a novel MOF@HOF composite material for the analysis of small molecules. The results have shown that it has a high D/I capacity, low background interference, strong salt tolerance, and good signal reproducibility and is suitable for the analysis of flavonoid compounds.^945^ Li et al. developed novel boric acid-functionalized magnetic multiwalled carbon nanotubes with the flexible branched polymer Fe_3_O_4_@MWCNTs@ε-PL@BA and applied them to detect LMW flavonoids.^1102^ Compared with conventional organic matrices, MWCNTs have higher ionization efficiency, clean background signals, good sensitivity, and high salt tolerance due to the unique electron–phonon interaction and high introduction density of boric acid functional groups, making them suitable for the direct and quantitative analysis of target flavonoids in complex food samples. Liu et al. developed a graphene-based LDI-TOF MS to detect coumarin flavonoids and their derivatives.^810^ Their evaluation showed that GO, particularly with larger transverse sheet sizes, was the most effective matrix for analyzing flavonoids and coumarin derivatives in negative ion mode due to its superior D/I efficiency. Cha et al. have also used colloidal graphite-assisted LDI-MS to successfully image secondary metabolites such as small flavones.^1103^ The dispersed colloidal graphite particles exhibited high spatial homogeneity, facilitating precise imaging. Furthermore, the study demonstrated tissue-specific accumulation of flavonoids in Arabidopsis flowers and elucidated the spatial distribution and extent of light-induced flavonoid accumulation within Arabidopsis stem segments.

A comprehensive analysis of the structure and function of flavonoids, along with their spatial positional changes during various physiological activities in plants, is invaluable for advancing our understanding of the molecular mechanisms through which flavonoids mitigate external stressors. Moreover, the recently reported MALDI matrices demonstrate significant potential for the detection of flavonoids, particularly the novel MALDI matrices, which offer critical insights into the identification of specific flavonoid classes.

#### 4.2.3 Outlook

Metabolomics, a key component of systems biology, has garnered significant attention in life sciences for its ability to provide functional insights at the ultimate level of omics research. Recent advances in MALDI-MS have greatly advanced metabolomics, providing several beneficial properties for the analysis of small molecules. These properties include high tolerance to salts and buffers, rapid analysis, high absolute sensitivity, low sample consumption, and the possibility of sample storage. At present, many classical and novel matrices have been used for the detection and imaging of broad spectrum and specific categories of small molecules, especially important categories of metabolites such as NTs, vitamins, alkaloids, plant hormones, and flavonoids. However, the analysis of metabolites (or small molecules) via MALDI-MS remains challenging. These challenges are most pronounced when using widely used SOMs, which produce substantial background noise in the LMW region, overlapping with the *m*/*z* range of typical metabolites, thereby obstructing the detection of target ions. Furthermore, the “sweet-spot” effect impairs reproducibility in low-signal-intensity regimes, ultimately compromising quantitative reliability. Emerging matrices, such as ILMs and inorganic matrices, show promise in mitigating certain background interferences. However, in the absence of a universal “perfect” matrix capable of addressing all analytical challenges, selection remains contingent on the specific analyte class under investigation. Consequently, future efforts should prioritize the systematic development and investigation of novel matrices designed to suppress background interference, enhance crystallization homogeneity, improve ionization reproducibility, and strengthen performance in the LMW range.

### 4.3 MALDI Matrices for Lipidomic Analysis

While MALDI-MS remains the gold standard for MS-based proteomics,^1104^ its emerging role as a powerful tool for MSI is also being increasingly recognized.^1105^ Recent years have seen a shift in research focus from “proteomics” to “lipidomics”, with the spatially resolved analysis of lipid distributions within tissues, termed “spatial lipidomics”, becoming a crucial application of MSI.^32^ This shift is due to the superior detectability of lipids by MSI, their ubiquitous presence at similar levels in vertebrates,^1106^ and the scarcity of alternative techniques for spatial lipid analysis.^1107^

Lipids are amphipathic biomolecules characterized by hydrophobic structural features, synthesized through an initial anionic thioester condensation reaction (fatty acid synthase) or the carbocation condensation of branched chain pyrophosphate intermediates (isoprene pathway).^1108^ LIPID MAPS (www.lipidmaps.org) has established a classification system that divides individual lipid species into eight categories (Figure 91),^1108,1109^ including FAs, glycerolipids (GLs), glycerophospholipids (GPLs), SLs, sterol lipids (STs), prenol lipids, saccharolipids, and polyketides, with further distinctions based on their polar head groups.^1110^ The complexity of the effects of lipids on biological processes lies in their unique physical and chemical properties, which are essential for fundamental cellular functions.^1111^ For example, the hydrophobicity of lipids enables the formation of cell membranes that create boundaries within organisms, separating them from the hydrophilic environment and establishing essential membrane potentials.^1112^ Additionally, lipids are crucial for energy storage and signal transduction and participate in autocrine, paracrine, and endocrine regulatory processes both intermolecularly and intramolecularly.^1112^ Extensive research indicates that abnormalities in lipid metabolism and homeostasis are linked to various human diseases, including diabetes and obesity, atherosclerosis and stroke, cancer, mental disorders, neurodegenerative diseases and neurological disorders, as well as autoimmune diseases.^1110,1113^ Owing to these critical functions of lipids in organisms, the importance of lipidomics in the biological sciences is increasingly recognized.

**Figure 91:**
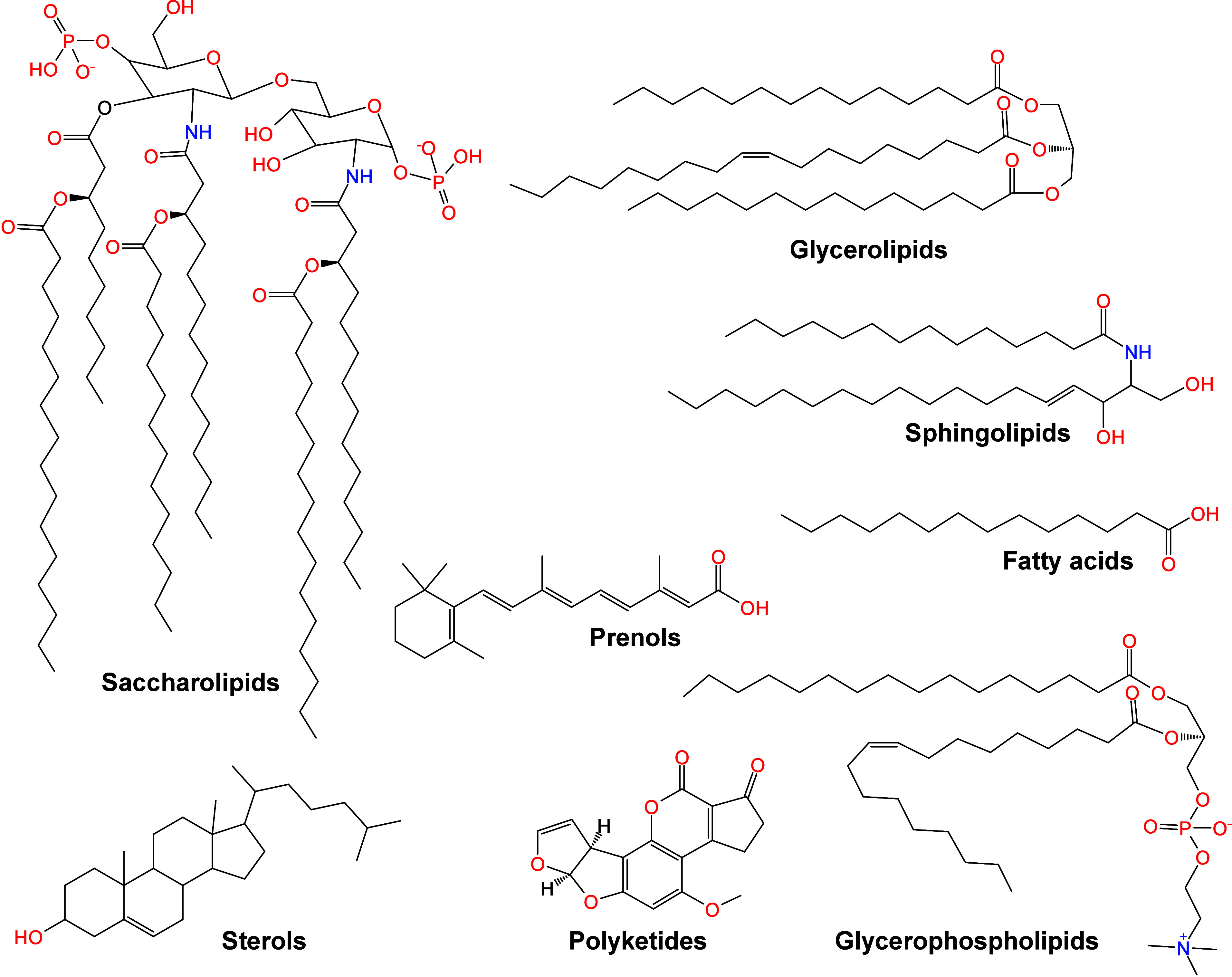
Example structures for the eight lipid categories as defined by the International Lipids Classification and Nomenclature Committee (ILCNC) in 2005 and implemented in the LIPID MAPS Structure Database (LMSD). Reproduced with permission from ref 1112. Copyright 2020 Authors. Mass Spectrometry Reviews published by John Wiley & Sons.

Despite the importance of lipids, research on these molecules has been limited by difficulties in analytical techniques. The selection of matrices for *in vitro* and *in situ* lipid analyses by MALDI-MS is not straightforward because of several factors, including the LMW of lipids (<1,500 Da), considerable molecular diversity, closely related *m*/*z* values, relative insolubility in aqueous systems, and significant concentration variations within crude samples.^1114,1115^ Therefore, it is imperative to select appropriate matrices tailored to distinct research objectives and targets. These matrices should either selectively enrich specific lipid classes or enhance the efficient ionization of a broad spectrum of lipids, independent of their varying physicochemical properties (Table S10). Furthermore, employing a simple detection strategy that incorporates both positive and negative ion modes allows for a more comprehensive capture of lipid information from the sample, thereby significantly increasing data coverage and reliability.

#### 4.3.1 MALDI Matrices for Untargeted Lipidomic Analysis

Various matrices have been screened and identified as suitable for lipid analysis. Key prerequisites for matrices utilized in this context include low chemical noise and interference from matrix-related ions, adequate absorbance at the specified laser wavelength, high solubility in the analyte solvent, appropriate acidity and basicity, and enhanced ionization efficiency for the analyte molecules.^1114^ Matrices commonly used for lipid analysis include DHB, which remains the most widely accepted matrix,^1116^ as well as CHCA,^1117^ PNA,^1118^ 9-AA,^469,1119^ 2-MBT,^315^ DMAN,^425^ and DAN.^574^

Lipids possess diverse chemical structures, many of which are more readily detectable under one ionization polarity than under the other (positive or negative ion mode). For instance, PCs and SMs are typically observed as positive ions, whereas other lipids such as PAs, PEs, PSs, PIs, and STs, are typically detected as negative ions in MALDI-MSI.^1120^ DHB is one of the widely used matrices for positive ion mode lipid analysis, and offers high vacuum stability and the ability to produce clearer spectra with reduced analyte ion fragmentation without interfering with analyte identification.^1116^ However, as a MALDI matrix, DHB also has several drawbacks, such as the tendency to form large crystals, leading to uneven analyte distribution, poor point-to-point reproducibility, and reduced mass resolution.^1121^ Additionally, the relatively high acidity of DHB can limit its sensitivity to certain PLs, such as PAs, PSs, PIs, PGs, and PEs, especially in negative ion mode.^1118^ To address challenges involving crystal size and uniformity, Niehaus et al. successfully achieved subcellular-level MALDI-MSI by sublimating DHB onto tissues to form smaller crystals.^103^ Stoyanovsky et al. explored the use of *O*-alkylation derivatives of DHB (*i.e.*, DHB-C*_n_* H_2*n*+1_(C_6_H_13_, C_9_H_19_, and C_12_H_25_)) for MALDI-MSI of PLs in brain tissue.^1122^ Compared with traditional DHB, these modified DHB facilitated the formation of finer crystals and improved spatial resolution by enhancing the incorporation of DHB-C_*n*_H_2*n*+1_ into hydrophobic PL bilayers. CHCA is another matrix used for lipid imaging in positive ion mode, but its application in tissue imaging is limited by high-abundance matrix-related background signals.^54^ Recent studies suggest that CHCA can be transformed into more efficient matrix compounds, such as CNAA for PL analysis and CNDA for facilitating the detection of neutral lipids, such as DAGs and TAGs, in positive ion mode.^264^ The CHCA-derived ionic matrix CHCAB enabled the successful imaging and identification of approximately 30 lipids in the mouse cerebellum using MALDI-TOF/TOF-MS.^598^

Several novel and promising matrices have been proposed to facilitate negative mode ionization. Among these, 9-AA and DMAN are particularly effective for acidic analytes such as LMW organic acids and PLs. 9-AA, a moderately strong base (p*K*_a_ = 9.99), is used for the rapid and sensitive analysis of lipids in metabolites or cell extracts.^1053^ Sun et al. demonstrated the use of 9-AA in negative ion mode for analyzing PLs and cardiolipins (CLs) from cardiac lipid extracts.^1119^ Additionally, the small ISD of 9-AA in MALDI-MS is important for the orthogonal selectivity of lipid ionization and analysis of complex biological samples. Wang et al. identified the optimal molar ratio of NEDC and 9-AA (NEDC/9-AA) as 1.35 through systematic testing of various mixtures. This optimized mixture enhances lipidomics analysis and MALDI-MS imaging in a high-throughput, semiquantitative manner, enabling the analysis and mapping of all major classes of PLs and sulfatides from lipid extracts or tissue slides.^643^ Despite its extensive application in negative ion mode for the detection of LMW compounds, including various lipids, the utility of 9-AA can be hindered by the interference of matrix-ion clusters.^33^ Furthermore, 9-AA demonstrates significant solvent dependency, with signal intensities exhibiting considerable variation based on the solvent system employed.^1123^ For instance, the signal intensity of peaks are markedly amplified when chloroform is utilized as the solvent, whereas this enhancement is considerably less pronounced with alternative solvents such as 2-propanol or acetonitrile. DMAN, a highly basic “proton sponge” with a p*K*_a_ of approximately 12, effectively extracts protons from weak acids, reducing matrix-associated interfering ions and making this matrix ideal for the MALDI-MS analysis of metabolites and lipids under 800 Da.^52^ However, DMAN is unstable under high vacuum conditions, with deposited matrices being sufficiently volatile at ambient temperatures to sublime under MALDI-MS vacuum.^363^ Calvano et al. successfully developed a binary matrix of DMAN/9-AA, which allows for the rapid and easy detection of approximately 50 major membrane components (such as FAs, monoglycerides, DAGs, TAGs, PLs, glycolipids, and CLs) in both gram-positive (*Lactobacillus sanfranciscensis*) and gram-negative (*Escherichia coli*) microorganisms.^642^

Most commercial MALDI matrices are designed for unipolar detection, but dual-polarity MALDI matrices offer a method for the comprehensive mass spectrometric analysis of both positively and negatively charged lipids within a single sample. Researchers have recently identified several dual-polarity matrices suitable for high-spatial-resolution MALDI-MSI. An early example is DAN, introduced by Thomas et al., which is effective for lipid detection in both positive and negative ion modes.^1124^ Studies have shown that sublimated DAN is highly efficient in MALDI-MSI, providing abundant lipid features under both positive and negative polarities, along with low background interference, high resolution, and excellent vacuum stability.^363^ However, the high toxicity of DAN, including its potential carcinogenic and mutagenic properties, limits its widespread use in the MALDI tissue imaging of lipids.^51^ In 2004, PNA was reported as a useful matrix for the MALDI imaging of endogenous lipids, particularly in porcine and bovine lens tissues.^1118^ Steven compared the performance of PNA and CHCA in positive ion mode, and reported that PNA is as effective as, or even better than, CHCA in terms of lipid ion intensity, leading to reduced ion fragmentation and analyte migration when air spray matrix deposition is used.^448^ Unfortunately, PNA is prone to sublimation under typical MALDI-MSI vacuum conditions, which makes them unsuitable for imaging larger tissue samples at high or ultrahigh spatial resolution.^315^ Astigarraga et al. developed 2-MBT as an effective alternative to DHB for PLs MALDI-MS of PLs in brain and liver tissues.^315^ 2-MBT enables direct analysis and imaging of PLs in liver and brain samples, with characteristics such as low vapor pressure and low acidity, allowing prolonged acquisition times, the identification of more lipid species, and good spatial resolution with high detection reproducibility.

Recently, novel matrices have continuously emerged for lipid MS analysis and imaging, including DT, quercetin, DPH, DCH, MEK, AuNPs, and AgNPs. DT has emerged as a promising MALDI matrix that enhances the detection of endogenous lipids, particularly polar lipids, in positive ion mode. During the imaging of rat liver and bovine lens tissue sections, more than 70 lipid species were detected, including PCs, PEs, SMs, PSs, PGs, PAs, STs, TAGs, ceramide phosphates (CerPs), and acylcarnitines (ALCs).^362^ Wang et al. demonstrated that the use of quercetin as a MALDI matrix improves lipid imaging, successfully imaging 212 lipid species from rat brains in a single experiment for the first time, and revealing asymmetrical lipid distributions in the left and right hippocampus^51^. DPH serves as an effective MALDI matrix that minimizes background signals and enables high-sensitivity detection of small FAs (*m/z* 200-350) and larger lipids (*m/z* up to 1,000) in negative ion mode, facilitating MALDI-MSI of various lipids, including FAs, LPLs, PLs, and STs, in rat and mouse brain tissues.^472^ In dual-polarity MALDI-MSI, 3-APH has exhibited superior performance with higher sensitivity, broader molecular coverage, and lower background noise, showing the potential for detecting metabolites related to mouse brain metabolism and ischemic responses, including nucleotides, FAs, glycerides, GPLs, SLs, and glycolipids.^442^ The unique structure of plant tissue samples (such as cell walls and waxy cuticles) further restricts the comprehensive characterization of endogenous molecules by MALDI-MSI, impacting its broader application in botany. The development of matrices such as DCH and MEK extends the coverage of lipid detection and imaging in both animal and plant tissues. DCH offers advantages such as low vacuum volatility, good chemical stability, the formation of millimeter-sized crystals, and uniform matrix coatings, making it suitable as a MALDI-MSI matrix for lipid detection in both animal and plant tissues in positive ion mode.^459^ MEK, as a negative-ion MALDI matrix, has outperformed commonly used matrices like 2-MBT in terms of quantity and sensitivity of lipid detection and imaging, complementing other positive ion MALDI matrices to provide broader coverage for both animal and plant tissues.^414^ Recent studies have also highlighted the significant potential of inorganic matrices in lipid analysis. For example, AgNPs have been utilized in negative ion mode to map the distribution of FAs in mouse retinal sections,^720^ whereas AuNPs have been employed to image sulfolipids and gangliosides in mouse brain sections.^708^

#### 4.3.2 MALDI Matrices for Targeted Lipid Analysis

##### 4.3.2.1 Fatty Acids

FAs are saturated or unsaturated fatty carboxylic acids derived from biosynthesis, dietary intake, intestinal absorption, and adipose tissue release.^1125^ FAs are a major energy source^1126^ and serve as precursors of prostaglandins,^1127^ essential components of complex lipids,^1128^ and substrates for the synthesis of fats, lipoproteins, liposaccharides, and eicosanoids.^1129,1130^ The composition of lipids or FAs in response to dietary intake or nutrients is analyzed primarily in the liver, heart, endothelium, kidneys, adipose tissue, red blood cells, plasma, brain, and retina.^720^ Visualizing the distribution of FAs is crucial for understanding their role in biological systems. As acidic compounds, FAs are more easily detected in negative ion mode.^1107^ However, a significant challenge in performing MALDI-TOF-MS on free FAs, especially mixtures, is the overlap of FA peaks with matrix signals, which are particularly abundant in the low-mass range, potentially leading to the suppression of small peaks.^1131^ Additionally, the high intensity of matrix signals reduces the sensitivity of FA detection.

To address these issues, several efforts have been made. In addition to nontargeted matrices such as 9-AA, DMCA, and DPH, various other matrices have been developed to improve the detection and imaging of FAs. One potential approach involves using the matrix meso-tetrakis porphyrin (F20TPP), which has a large molar mass (M = 974.57 g/mol) and is less prone to gas phase fragmentation, thereby preventing matrix overlap issues. F20TPP, when mixed with saponified oil solutions, allows reproducible MALDI-TOF spectral analysis of various plant oils, with ions predominantly in the form of sodiated carboxylate salts [RCOONa+Na]^+^.^1132^ However, despite its effectiveness, this method is no longer widely used because of its relatively low sensitivity and its ability to accurately identify only saturated FAs.^566^ Napagoda et al. proposed using 1,14-diaza[5]­helicene(1)­[9c,10] as an effective MALDI matrix for analyzing FAs and organic acids and successfully detected signals from two FAs (PA and SA) and the plant hormone ABA, with clear deprotonated signals observed for arachidic and ricinoleic acids.^433^ Another method employs ammonia-treated NEDC (ATNEDC) treated with ammonia in negative ion mode as a novel MALDI matrix for the direct quantification of serum FAs through MALDI-FTICR-MS, identifying free FAs present in serum.^1131^ Additionally, Ling et al. synthesized a new matrix, DBDA, for the negative ion MALDI-TOF-MS analysis of small molecules, particularly FAs.^474^ DBDA was used to accurately quantify FAs in serum and successfully obtained MALDI-MS images of FAs in mouse brain tissue (Figure 92a).^1133^

**Figure 92:**
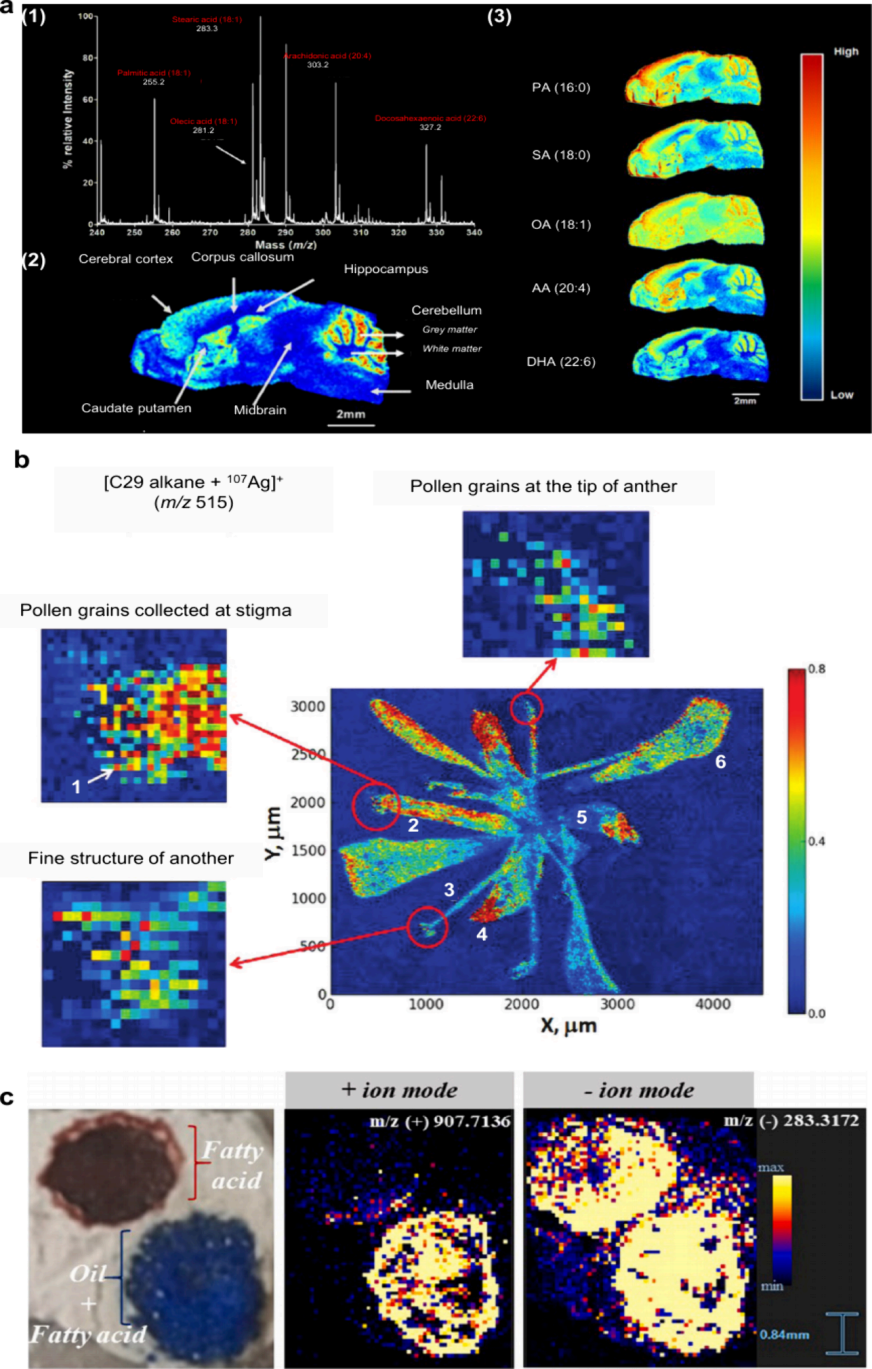
(a) DBDA as a matrix for the MALDI analysis of fatty acids in murine brain tissues. (1) On-tissue MALDI-TOF mass spectrum for *m/z* 240–340. (2) Ion density map of DHA (*m*/*z* 327.2) showing distinct brain regions. (3) MALDI-MSI analysis was performed on sagittal cryosections from 10-week-old mice. Ion density maps for palmitic (PA), stearic (SA), oleic (OA), docosahexaenoic (DHA), and arachidonic acid (AA) are shown. Adapted and reproduced with permission from ref 1133. Copyright 2023 American Society for Mass Spectrometry. (b) MS imaging of an Arabidopsis flower at the single-cell level at high spatial resolution. MS image of a silver ion adduct of C29 alkane (*m*/*z* 515) on an Arabidopsis flower obtained with a spatial resolution of 12 μm (12 μm for both laser spot size and raster size). The letters indicate the positions of single pixels for the mass spectra, (1) pollen grain, (2) side of carpel, (3) stamen filament, (4) tip of sepal, (5) middle of sepal, and (6) side of petal. Adapted and reproduced with permission from ref 1134. Copyright 2010 American Chemical Society. (c) MALDI IMS of linseed oil (positive mode, TAG, *m*/*z* 907.7136) and stearic acid (negative mode, [M–H]^−^, *m*/*z* 283.3172) using G-CN (1 mg mL^–1^). The blue area contains linseed oil and stearic acid mixed with Egyptian blue, and the red area contains stearic acid mixed with red hematite. Reproduced with permission from ref 1135. Copyright 2022 Elsevier BV.

Numerous efforts have been made to explore the use of inorganic matrices, which are advantageous because they provide a clean background. In MALDI-MS, silver cores exhibit specificity for FAs, leading multiple research teams to utilize AgNPs as a matrix for MALDI-MS analysis of FAs in mouse liver and retinal tissues^720^ to investigate the impact of FA distribution on fundamental biological mechanisms and various diseases. Furthermore, by utilizing AgNPs as the matrix and combining high spatial resolution with high mass resolution techniques, successful MALDI-MS imaging of epidermal lipids in *Arabidopsis thaliana* was achieved at the single-cell level. This approach enabled detailed chemical imaging of entire flowers at a high spatial resolution of approximately 12 μm, allowing the visualization of anther and stigma substructures, as well as individual pollen grains (Figure 92b), and the direct identification of lipid metabolites on root surfaces.^1134^ In recent studies, researchers proposed the use of cyanographene (G-CN) as a matrix to detect FAs by MALDI-MS/MSI at both polarities. This approach enables the detection of lipids below 100 ng with high quality and demonstrates minimal interference from inorganic pigments, ensuring high-sensitivity detection and visualization (Figure 92c).^1135^ Another study demonstrated that G and GO effectively enriched five long-chain fatty acid models because of their high surface area and strong interaction forces, enhancing ionization efficiency and enabling sensitive detection of these FAs at low concentrations in real biological samples.^1136^ Moreover, using chemically modified nanometer-scale silicon as a matrix enables the laser desorption/negative ionization of small-molecule acids, achieving detection limits of 50 *p*mol/μL for sulfonic acids and 200 *p*mol/μL for FAs without detecting polymers or cationic adducts, and has been successfully applied to analyze FAs in milk and tick samples.^1137^

In summary, the analysis of FAs has garnered increasing scientific interest, necessitating fast and reliable analytical methods. While established GC-MS methods remain the predominant choice for FA detection, MALDI-MS offers the potential to investigate the spatial distribution of FAs. However, the matrices currently employed in MALDI analysis have inherent limitations, and to date, no universally accepted “best” matrix for FA analysis has been established, warranting further investigation.

##### 4.3.2.2 Glycerolipids

TAGs serve as the primary form of excess energy storage in animals and are primarily found as compounds such as galactolipids mono- and digalactosyldiacylglycerol (MGDG, DGDG) in plants.^1138^ TAGs are crucial for energy storage and exist predominantly as lipid droplets in the adipose tissue of organisms. Under the action of phospholipase C, DAGs are generated from GPLs by cleaving the polar head groups, and they serve as important lipid-derived second messengers.^1139^ Recent studies have linked specific TAGs with various diseases, especially cardiovascular diseases and metabolic syndromes,^819^ highlighting their potential as preclinical biomarkers for future clinical applications.^1140^ Current enzyme-based clinical assays generally measure only the total TAG levels in plasma (or serum), neglecting the profiles of individual TAG species or subclasses.^1140^ Understanding the diversity of TAG species is essential for elucidating the physiological functions of individual TAG molecules and for developing potential biomarkers for related diseases. TAGs can be readily detected by traditional MALDI-MS when they are present in samples such as edible oils or exist independently.^1141^ However, the varying ionization efficiencies of lipids often lead to the suppression of nonpolar lipids, such as DAGs, by more polar lipids such as PCs. Although TAGs are usually present at sufficiently high concentrations to avoid significant signal suppression, low-abundance TAGs may still be overshadowed by the signals of other lipid species.^702^ As a result, MALDI-MS analysis of TAGs in crude lipid extracts typically requires only simple chromatographic purification methods, such as TLC or solid-phase extraction.^1119,1142^ To date, there have been few reports on matrices that facilitate the direct detection of low-abundance TAGs within complex mixtures.

Interestingly, regardless of the matrix used, DAGs and TAGs consistently appear as adducts with alkali metal ions or other alkali metal ions, while H^+^ adducts are not detectable at all.^566^ When standard DHB is used as a matrix, DAGs^1143^ and TAGs^1144^ can be conveniently and sensitively detected by positive ion MALDI-TOF-MS. Further investigations have demonstrated that the addition of NaCl or other sodium salts to DHB can increase the sensitivity of TAG analysis.^1145^ TAGs are prone to in-source fragmentation; adding a reinforcing alkaline substance such as NaOH can reduce this occurrence, thereby improving signal intensity.^1146^ LiDHB has also been shown to be an effective MALDI matrix. Under standard conditions, it can generate [M+Li]^+^ ions through lithiation, making it suitable for the analysis of nonpolar long-chain lipids such as DAGs, TAGs, and long-chain esters, as well as hydrocarbons and polymers.^569^ In 2015, Lou et al. demonstrated that the use of CHCA dissolved in tetrahydrofuran can increase the detectability of TAGs in the presence of PLs and mitigate the ion suppression effects of PLs in known complex mixtures.^1147^ Additionally, CNDA, a derivative of CHCA, has been developed as a lipid MALDI matrix. Studies on lipid profiling in milk, plasma, blood, and fish samples have shown that CNDA has excellent ionization properties, particularly for the analysis of neutral lipids such as DAGs and TAGs.^264^ Utilizing AGO as both a lipid extractor and a MALDI matrix allows for the efficient extraction and quantification of TAGs in plasma.^819^ This method facilitates monitoring changes in TAG species across different samples and through collision-induced dissociation tandem MS (CID–MS^2^), provides detailed information on the FA composition of plasma TAGs. As shown in Figure 93a, while the use of DHB as a matrix primarily resulted in the detection of PL signals, the use of AGO significantly increased the detection of TAGs in plasma, thereby providing a more comprehensive analysis of plasma TAG composition. Son et al. demonstrated that citrate-capped AuNPs (AuNPs-CBS) with a 12 nm diameter can serve as an effective matrix for directly profiling TAGs from crude beef lipid extracts and detecting trace levels of TAGs in total brain lipid extracts, free from interference from polar lipids.^702^ Another study employing Au-CBS conducted MSI of TAGs in fresh-frozen slices of mouse liver and rabbit adrenal tissue, and this approach achieved spatial resolution between 10 and 25 μm (Figure 93b).^1148^

**Figure 93:**
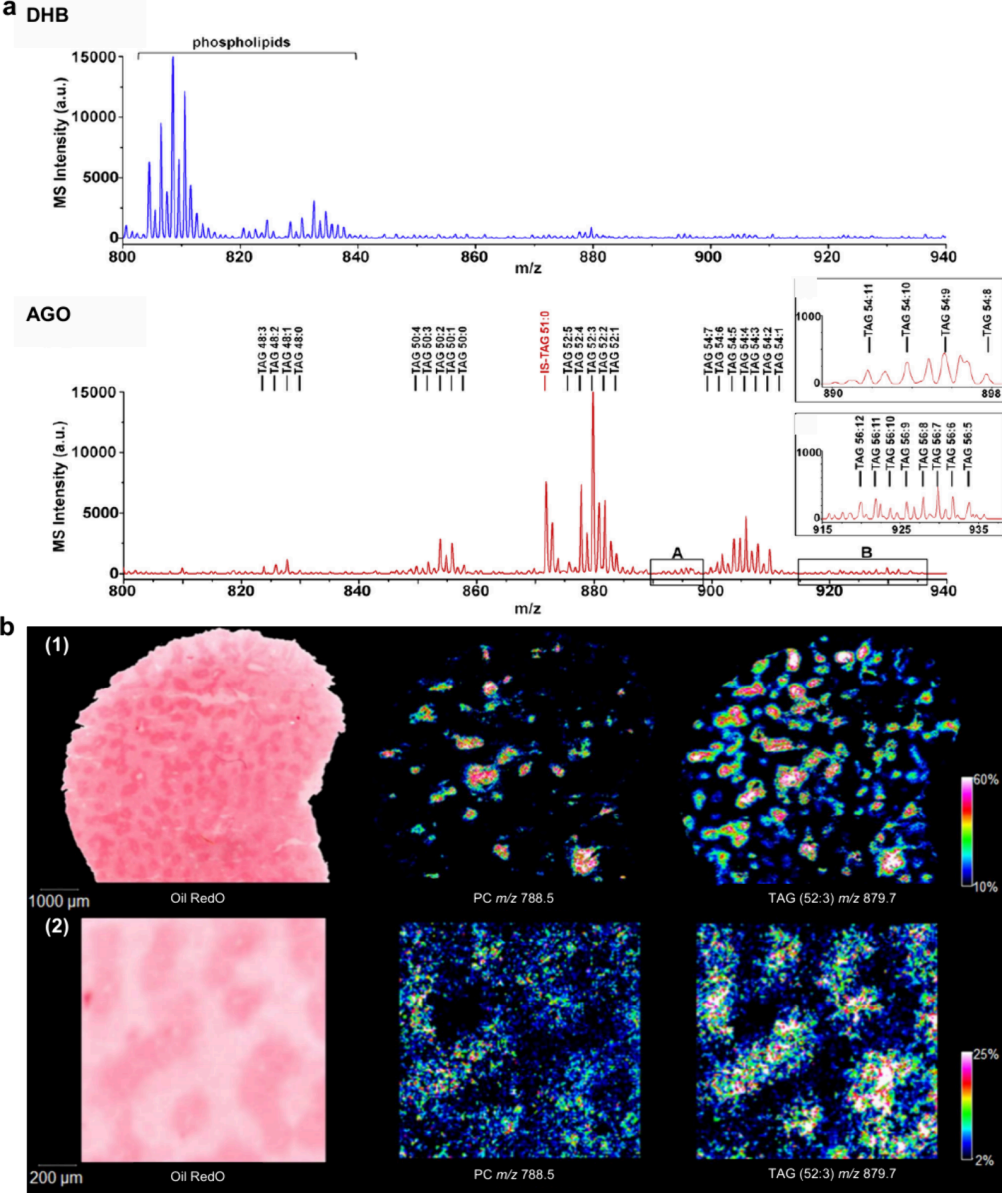
(a) The lipids were detected mainly as [M+Na]^+^ ions and the *m*MS spectra of a plasma sample analyzed by using routine MALDI-MS (with organic solvent to extract lipids and DHB as the matrix and graphene oxide (AGO) as the matrix); *m*/*z* peaks corresponding to the detected TAGs were marked. Adapted and reproduced with permission from ref 819. Copyright 2018 Elsevier BV. (b) CBS-Au-LDI IMS of a mouse liver section acquired with lateral resolutions of (1) 25 μm and (2) 10 μm. The distribution of TAG (52:3) at *m*/*z* 879.7 is associated mainly with lipid islands (darker red Oil RedO areas). Adapted and reproduced with permission from ref 1148. Copyright 2016 American Chemical Society.

##### 4.3.2.3 Glycerophospholipids

PLs are essential components of cells, playing numerous critical roles in cellular functions. Most cellular PLs form bilayer membrane structures, whose integrity and physical properties are vital for life processes.^47^ GPLs represent one of the most significant subclasses of PLs, serving not only as critical structural lipids, such as components of cell membranes, but also as secondary messengers, including PAs, PIs, and LPLs, that play regulatory roles in metabolism.^1149^ GPLs are composed of glycerol, FAs, and PAs, with various head groups attached to the phosphate group, resulting in complex structures and diverse chemical properties^1150^. As shown in Figure 94, the most common GPLs with a single phosphate group include PC, PE, PI, PS, PG, and PA.^1109^ A subclass of GPLs are LPLs, which contain a single fatty acid predominantly at the sn-1 position of the glycerol main chain. Examples of LPLs include LPCs, lysophosphatidic acids (LPAs), lysophosphatidylethanolamines (LPEs), lysophosphatidylinositols (LPIs), lysophosphatidylglycerols (LPGs), and lysophosphatidylserines (LPSs) (Figure 94). To date, various matrices have been reported for the identification and characterization of GPLs by MALDI-MS.^1150^

**Figure 94:**
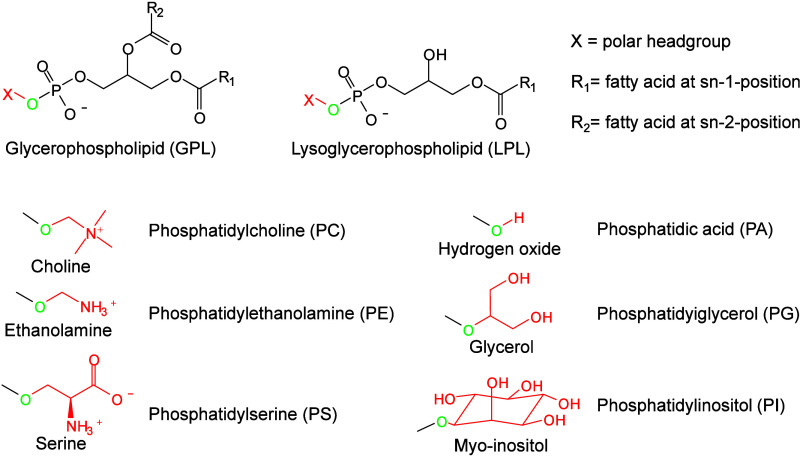
Chemical structures of headgroups of major glycerophospholipids (GPLs). The polar moiety (X), which is connected to the phosphate group, defines the individual class of GPLs. The identities at R_1_ and R_2_, which vary with different numbers of carbon atoms, numbers of double bonds, locations of the double bonds, etc., define the individual molecular species of each lipid class.

Different types of GPLs have varying detection properties, with PCs being the most extensively studied. This is largely due to its abundance in cell membranes and the presence of a quaternary ammonium group that maintains a permanent positive charge regardless of pH,^1107^ making PC the most sensitive target for detection in positive ion mode within lipid mixtures. In contrast, PE is among the more challenging lipids to detect. In positive ion mode, PE has a significantly lower detection sensitivity than PC because of its easily deprotonated amino group, which can result in a negatively charged phosphate group, unlike PC’s permanent positive charge.^1151^ In negative ion mode, the detection of PEs is often hindered by more acidic lipids such as PIs or sulfatides, which can suppress their signals.^1152^ Additionally, acidic GPLs such as PIs, PSs, and PAs generally exhibit lower sensitivity in positive ion mode^1153^ because of their charge characteristics and increased polarity, which reduces their interactions with the polar matrices essential for ionization.

DHB is among the most established and well-characterized MALDI matrices and has facilitated the acquisition of extensive data on GPLs.^1116^ However, the acidic nature of DHB (pH ∼ 2.5) leads to the protonation of anionic headgroups, making it challenging for detection in negative ion mode. This limitation can be partially addressed by using less acidic matrices such as PNA (pH ∼ 6.3)^1118^ and 9-AA,^466^ thereby improving the detection and quantification of PLs in both positive and negative ion modes. In addition to these commonly used matrices, the basic compound AAN has been identified as an alternative matrix for the negative ion MALDI-MS of single-class GPLs (Figure 95a): PAs from soybean, PGs from egg-yolk lecithin, PSs from the bovine brain, and PIs from soybean.^404^ Using AAN in negative ion mode offers higher sensitivity and better uniformity, enhancing the detection capabilities for GPLs such as PEs, PSs, PGs, and PIs. Sublimed CMBT has been used for imaging in both positive and negative ion modes, providing a preliminary identification and spatial distribution of various lipids in mouse kidney tissues, with PCs and PGs highlighted in positive ion mode and PIs in negative ion mode (Figure 95b)^319^. Stubiger and Belgacem evaluated THAP as a MALDI matrix for the sensitive detection of PLs in positive ion mode. Salt doping (with sodium/lithium acetate or ammonium citrate) reduced spectral complexity and enabled the detection of PLs in the presence of PCs.^310^ The ILMs exhibit excellent solubility for various analytes, form homogeneous crystals with the analytes, and possess high vacuum stability, making them suitable for lipid analysis by MALDI-MS. Ham et al. developed a novel solid ionic crystal MALDI matrix composed of PNA and butyric acid, which reduced the complexity of positive ion spectra by generating protonated forms of each PL in normal human tear fluid for study^616^. Similarly, Li et al. synthesized a series of ILMs of CHCA for positive ion detection of PLs.^58^ Compared with the crystalline matrix DHB, these ILMs demonstrated higher signal intensity, smaller spot size, better spot uniformity, enhanced signal reproducibility, and comparable or improved detection limits for PLs. In addition, the ILM is based on two commonly used matrices (DHB or CHCA) and consists of a binary matrix ionic liquid composed of TFA and piperine,^637^ and a CHCAB ILM, *etc.* ^1154^ Both have been synthesized for improving the MALDI-MSI of PLs in tissues. In organic matrix-based MALDI-MS, PCs and PAs are detected mainly in positive ion mode: silicon NAPA, as an inorganic matrix, helps to detect difficult compounds such as PEs and glycosylated Cers, whereas in the negative ion mode, NAPA is dominated by the signal of PEs.^885^ Nanoflake-capped silicon nanowire (NGQD@MoS_2_/SiNW) material, with its high photothermal conversion and photoinduced charge transfer efficiency, significantly increases the LDI efficiency of lipids with varying polarities on its substrate.^1155^ This platform allows for MALDI-MS imaging to accurately reflect the abundance and distribution of PLs in non-small cell lung cancer tissue slices and adjacent normal tissues (Figure 95c). In addition, HOA-modified BaTiO_3_ NPs have been employed as liquid–liquid microextraction probes and identified by MALDI-MS, facilitating the efficient extraction and analysis of hydrophobic molecules in biological samples. This method demonstrated a good linear concentration range and low detection limits for PSs and PAs.^764^

**Figure 95:**
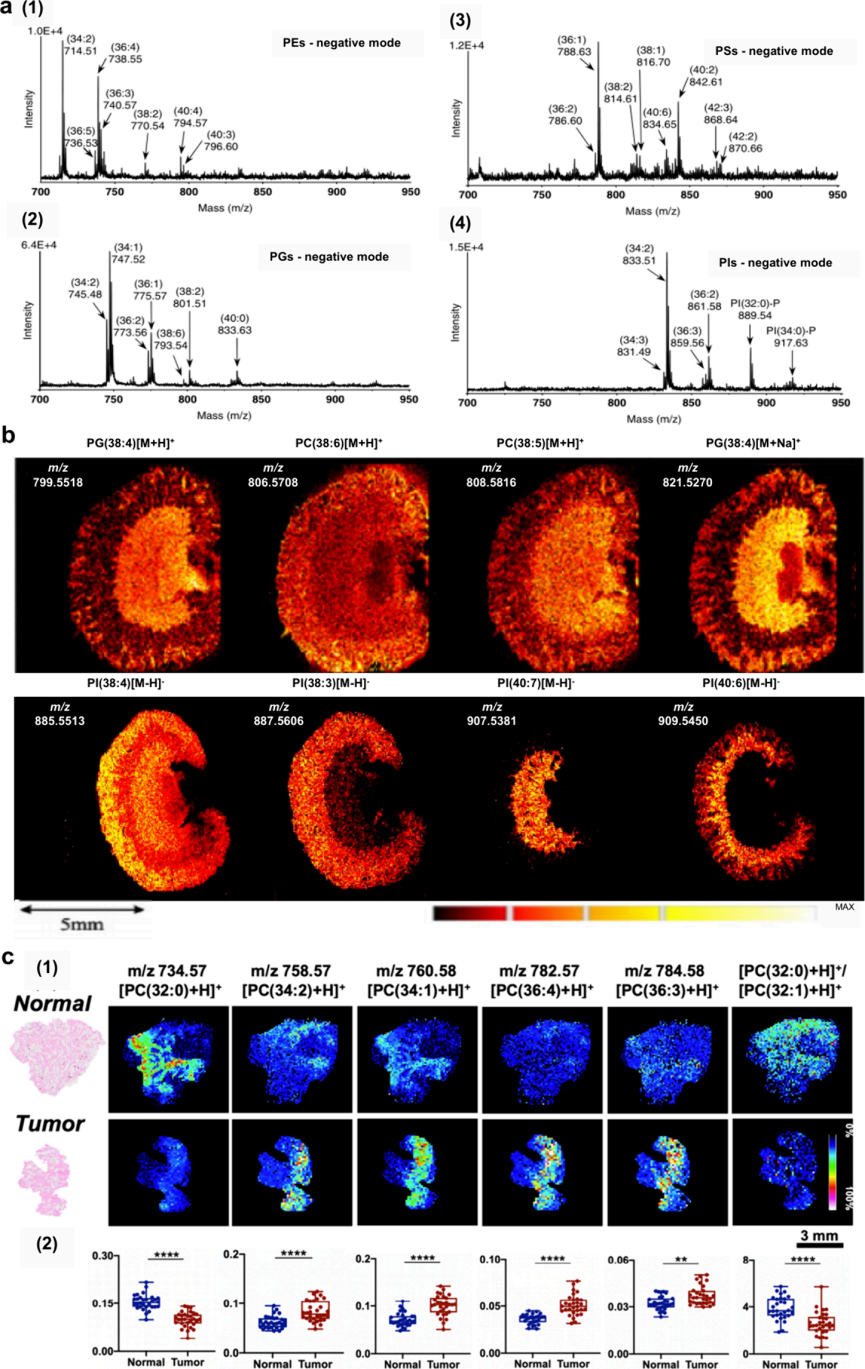
(a) Negative-ion MALDI mass spectra of single PL classes using 2-(2-aminoethylamino)-5-nitropyridine (AAN) as a basic matrix: (1) PEs from soybean, (2) PGs derived from egg-yolk lecithin, (3) PSs from the bovine brain, and (4) PIs from soybean. Adapted and reproduced with permission from ref 404. Copyright 2008 John Wiley & Sons. (b) Results of MALDI-MSI analysis of kidney phospholipids using a sublimated CMBT matrix in positive ion mode and negative ion mode. The analysis was conducted at a spatial resolution of 50 μm. Adapted and reproduced with permission from ref 319. Copyright 2023 The Authors, Rapid Communications in Mass Spectrometry published by John Wiley & Sons Ltd. (c) Distribution of representative biomarkers in adjacent normal and tumorous tissues from NSCLC tissue by MALDI-MSI using NGQD@MoS_2_/SiNWs as the matrix. (1) Distribution of representative potential biomarkers across tissue sections from NSCLC tissue. (2) Statistical box plots showing the ion intensity of the tumorous and adjacent normal tissue sections with the degree of differentiation. (**, *p* < 0.01; ****, *p* < 0.0001). Adapted and reproduced with permission from ref 1155. Copyright 2022 American Chemical Society.

To effectively analyze complex mixtures of GPLs, it is often necessary to separate them into individual lipid species for analysis or to conduct both positive and negative ion detection for comparative results. Therefore, the sample preparation method is crucial for the separation and detection of GPLs. For instance, to alleviate ion suppression issues with PE, recent studies have indicated that sample preparation methods can reduce ion suppression in PEs. A mixture of *n*-hexane/2-propanol (3:2, v/v) and pure CHCl_3_ has been recommended as the optimal solvent for extracting PSs, with slight acidification to minimize extraction losses.^1156^ PIs are relatively rare lipids in eukaryotic cell membranes. To minimize interference from other PLs, a selective two-step extraction method can be employed. This involves initially extracting cell materials with a neutral solvent mixture of CHCl_3_ and MeOH, followed by extraction of the pellet with an acidic solvent containing HCl or citric acid, thereby allowing the quantitative recovery of PIs.^1114^ PAs and LPAs are present in very small amounts in biological samples, and significant losses can occur during extraction. Alternatively, poor sample preparation might artificially induce PA formation, resulting in artifact.^1107^ To address these challenges, Morishige et al. utilized inorganic zinc complexes as matrices to capture LPAs and enhance their detectability.^1157^ The second challenge in the MALDI-MS analysis of PL mixtures is the presence of numerous possible cations that can be used to induce cationization of the PLs under investigation. Under physiological conditions, the most relevant cations are protons, sodium ions, and potassium ions, which are all at least partially soluble in organic solvents.^676^ Since the ionization pattern of an unknown sample reflects the ion distribution in solution, peak overlap can occur between cationic adducts of the same PL class containing different FAs. To resolve this issue, three approaches can be considered:^1152^ (i) desalting the sample, (ii) separating different lipid classes using chromatographic techniques, or (iii) adding a significant amount of an auxiliary salt such as CsCl. The addition of CsCl, which provides high concentrations of Cs^+^, increases the mass of the PLs sufficiently to prevent further adduct formation from causing peak overlap. This ensures that all observed mass differences are due to variations in the fatty acyl composition of different lipid species.^676^

Diphosphatidylglycerols, also known as CLs, are unique mitochondrial PLs characterized by a higher molecular weight than typical PLs and possessing two negatively charged phosphate groups. The compound DHAP can be used for negative ion mode MALDI-MS analysis of PLs,^1158^ particularly CLs.^1159^ Wang et al. used DHAP as a matrix for *in situ* detection of CLs by MALDI-MS, demonstrating that it generates the highest abundance of CL ions with minimal interference and S/N ratios. Furthermore, when combined with Cs^+^, DHAP further enhances detection sensitivity and provides valuable fragmentation information for structural characterization of CLs in rat organs.^1159^ Yang et al. have employed NRM as a promising matrix in MS imaging studies to detect CLs, achieving a sensitivity of approximately 4.7 pg/mm^2^ (equivalent to ∼0.47 ng/μL, assuming a 10 μm tissue thickness) for spotted synthetic CL standards.^1160^ Their approach also enabled the successful detection and mapping of endogenous CLs in complex biological samples such as bacterial arrays and mouse tissue sections. Phosphatidylinositol phosphates (PIPs) contain phosphate residues with three or more negative charges, which produce excellent signals in negative ion mode. However, MALDI-MS investigations of PIPs have been limited, with liquid chromatography coupled with LC-ESI-MS remaining the preferred method for detecting PIs and their phosphorylated derivatives, such as PIPs.^1161^

To date, MALDI-MSI has revolutionized the comprehensive imaging of PLs in complex biological tissues in both 2D and 3D.^1150^ The variability in the detectability of individual PLs using positive or negative ion mode detection complicates the analysis of mixtures. In positive ion mode, PCs tend to suppress the detection of other GPLs. Therefore, in the presence of abundant PCs, negative ion mode may be the optimal method for detecting all other GPLs.^566^ However, evidence suggests that PCs might also interfere with negative ion data, depending on the matrix used in MALDI-MS analysis.^1162^ Moreover, comprehensive studies have demonstrated that the detection of PIPs is highly challenging and lacking. Given the biological importance of these compounds, such investigations are urgently needed.

##### 4.3.2.4 Sphingolipids

SLs and GSLs are currently topics of intense research interest because of their potential as biomarkers for aging and diseases such as Niemann–Pick disease and Fabry disease.^1107^ SP molecular diversity arises from variations in fatty acyl groups during *N*-acylation and the formation of long-chain bases,^47^ making their structure more complex than that of GPLs because of the presence of numerous carbohydrate-based headgroups. GSLs refer to a broad subclass of SLs in which the carbohydrate portion is either singularly linked or multiply linked to the ceramide backbone^47^ and will be described in detail in the next section on glycomic applications. This section focuses primarily on the analysis of the most important SLs, such as SMs and Cers.

SMs are structural components of the plasma membrane and constitute 5–10% of the total PLs in most mammalian cells, with higher levels found in erythrocytes, ocular lenses, and the brain.^1163^ The chemical structure of SM backbone is illustrated in Figure 96a. The metabolite products of SMs, such as Cers, sphingosine, and sphingosine-1-phosphate, participate in cell signaling and physiological biochemical functions, including apoptosis, aging, and development.^1164^ SMs contain choline, which facilitates the detection of their [M+H]^+^ ions in MALDI-MS, even in the presence of other PLs.^1165^ However, their typical MALDI-MS positive ion signals are weaker than those of PC molecules, possibly because of the differing tendencies of the two compounds to produce negative ions:^1166^ SMs have an increased tendency to generate negative ions, while positive ions are produced to a lesser extent. Cers, which are biosynthetic intermediates of nervous lipids, play crucial roles in regulating signal transduction, apoptosis, cell differentiation, and inflammatory responses.^1167^ In MALDI-MSI experiments, Cers are often observed as positive ions that are dehydrated and appear as [M+H-H_2_O]^+^.^1168^

**Figure 96:**
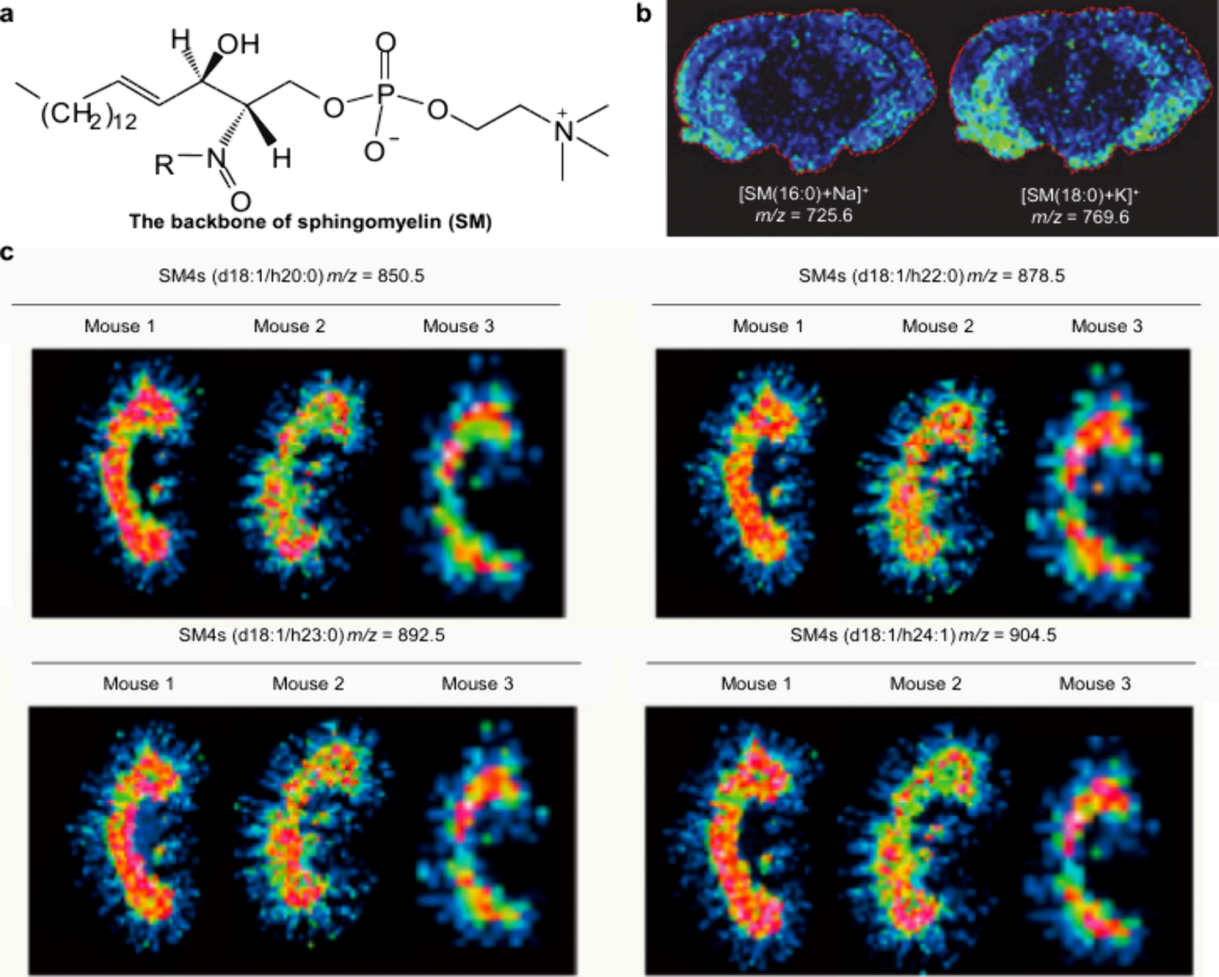
(a) Chemical structure of the backbone of sphingomyelin (SM). “R” represents a variable alkyl chain. (b) MSI of phosphosphingolipids from mouse brain sections sublimated with COOH-NHMe (IV) in positive ion mode at 150 μm spatial resolution. Adapted and reproduced with permission from ref 571. Copyright 2020 American Chemical Society. (c) Spatial distributions of various metabolites observed in three mice kidneys in negative ion mode, using 6-GAQ as a matrix. The step size is 200 mm for mice 1 and 2, and 400 mm for mouse 3. The above images were obtained from three db/m mice at 16 weeks old. Adapted and reproduced with permission from ref 1169. Copyright 2022 Elsevier BV.

MALDI matrices suitable for SLs analysis encompass a range of organic and inorganic materials such as DHB, 9-AA, 2-MBT, DT,^362^ 3-APH,^442^ and AgNPs.^708^ Some novel synthetic matrices, particularly those combining organic and inorganic components, have also shown promise in enhancing SP analysis. For instance, the Huang et al. demonstrated enhanced sensitivity to PLs by designing and synthesizing a series of novel MALDI matrices, especially the COOH-NHMe­(IV) matrix, and successfully observed PCs and SMs in mouse brain sections in positive ion mode (Figure 96b), as well as several GPLs in negative ion mode.^571^ Ma et al. synthesized a series of glycosylated matrices by linking glucose to existing amine matrices, among which 6-GAQ exhibited superior detection capabilities.^1169^ This matrix enabled the MALDI-MS detection of more endogenous metabolites in mouse kidneys and showed good selectivity for hydrophilic metabolites (Figure 96c), such as the successful detection of strong signals of hydrophilic sulfogalactoceramide. Han et al. synthesized AgNPs@PDA as a matrix for the analysis of lipids in both positive and negative ion modes. The PDA surrounding the silver core reduced silver cluster ions, overcoming the ion suppression effect of PCs and enhancing the analytical performance for other lipids, including PEs, HexCers, PSs, PIs, PIPs, and STs.^1170^ Moreover, Luo et al. employed PGC materials to imprint brain tissue sections, selectively enriching SLs while removing polar PLs. This MSI method demonstrated heightened detection sensitivity for low-response/low-abundance lipids in rat cerebellum imaging, enabling the detection of more lipid signals, particularly DAGs, Cers, and SMs, with clearer spatial distributions.^850^

##### 4.3.2.5 Sterol Lipids

The term “sterol" refers to molecules based on the cyclopentanoperhydrophenanthrene skeleton, among which mammalian sterols are primarily derived from cholesterol and its precursors, while plants and yeasts can also serve as sources of sterols through the food chain.^1109^ Cholesterol plays a crucial role as a component of membrane proteins and GPLs, and the emerging field of “cholesterolomics” highlights its importance in biological systems.^1171^ Recent studies have indicated that disruptions in cholesterol homeostasis are linked to several central nervous system disorders,^1172^ such as AD, Niemann–Pick disease, Rett syndrome, and Smith–Lemli–Opitz syndrome. Despite the growing body of related research, there has been relatively little interest in the MALDI-MS characterization of cholesterols, as this metabolite can be quantified from various biological samples using LC-MS^1173^ or can be measured using commercially available enzymatic reagent kits,^1174^ which are simple and relatively inexpensive.

The low proton affinity and acidity of cholesterol and cholesterol esters (CEs),^1172^ along with their relatively small molecular weight^566^, present major challenges for their detection using MALDI-MS, particularly because of interference from the matrix background. When using common organic matrices such as DHB are used, cholesterol cannot be detected in the expected form of H^+^ or Na^+^ adducts; instead, it can only be identified only at *m*/*z* = 369.3 ([m+H-H_2_O]^+^) through a water elimination process.^1175^ In a study conducted in 2011, Zaima et al. used DHB as the matrix for MALDI-MSI to identify two potential CE biomarkers, cholesterol linoleate (CE 18:2) and cholesterol oleate (CE 18:1), in lipid-rich regions of aortic atherosclerotic lesions in both mouse and human samples.^1176^ CEs are typically detected as adducts with alkali metal ions, particularly Na^+^ or K^+^, whereas H^+^ adducts remain undetectable.^1107^ Although further investigations have not been conducted, this may be attributable to similar reasons to those with TAGs,^1177^ namely that the H^+^ adducts of CEs are more unstable than Na^+^ adducts and cannot survive in the time-of-flight mass analyzer of MALDI equipment without fragmentation.

Various methods, including the use of synthetic organic and inorganic matrices, have been developed to increase the ionization of STs in MALDI-MS. For instance, coumarin with hydroxyl or carboxylic acid groups, especially DCA, exhibit improved sensitivity and stability for detecting hydrophobic compounds such as surface-active agents, androstenedione, and cholesterol in complex matrices without the need for chemical derivatization.^385^ Superbasic proton sponges based on PN, especially TPPN, can be used as a matrix to enhance the effectiveness of MALDI-MS detection for hardly ionizable compounds, particularly for sterols, steroids, fatty alcohols and saccharides.^432^ Yang et al. developed a reactive matrix, *N*-methylpyridinium-2-carbaldehyde iodide (MP2CA), suitable for the detection of cholesterol and other hydroxyl-containing sterols by MALDI-MS.^551^ As demonstrated in Figure 97a–c, the reaction of cholesterol with MP2CA (Figure 97a) enables the effective detection of cholesterol derivatives using a MALDI mass spectrometer, resulting in clearer spectral outcomes without the need to use other matrices such as CHCA and thereby circumventing peak overlap issues (Figure 97b,c). Modified polyvinylidene fluoride (PVDF), characterized by sp^2^ carbon, π–π conjugation, and oxygen-containing functional groups, acts as a highly effective ionization matrix for nonpolar lipids such as TAGs, FAs and cholesterol, enhancing their signals by a factor of approximately 10–20 and eliminating background interference compared with conventional MALDI methods.^1178^ The Lu team developed a simple method using composite materials of mesoporous graphene and zirconium-based MOFs (MG@UiO-66) combined with MALDI-TOF-MS for the effective detection of steroids in environmental water samples and successfully evaluated its performance with model steroids such as testosterone, methyltestosterone and androsterone.^911^ Several techniques incorporate salt doping and metal sputtering to improve analytical outcomes. For instance, Dufresne et al. employed a sodium acetate and carbonate buffer mixture at pH 10.3 to facilitate the sputtering of a gold layer (Au-CBS), which enabled the detection of sodium adducts of cholesterol (Figure 97d).^1148^ Similarly, Patti et al. directly deposited silver onto NIMS surfaces prior to tissue deposition and later introduced an aqueous AgNO_3_ solution for the analysis of cholesterol.^871^ Additionally, on-tissue chemical derivatization (OTCD) has been employed for MALDI-MSI of cholesterol, such as the two-step OTCD method using cholesterol oxidase and Girard-P hydrazine^167^, or using betaine aldehyde as an OTCD reagent^1172^. However, this topic lies beyond the scope of the present article and is not discussed in detail. Although some advances have been made, further research is needed to develop alternative and more straightforward methods for cholesterol detection using MALDI-MS.

**Figure 97:**
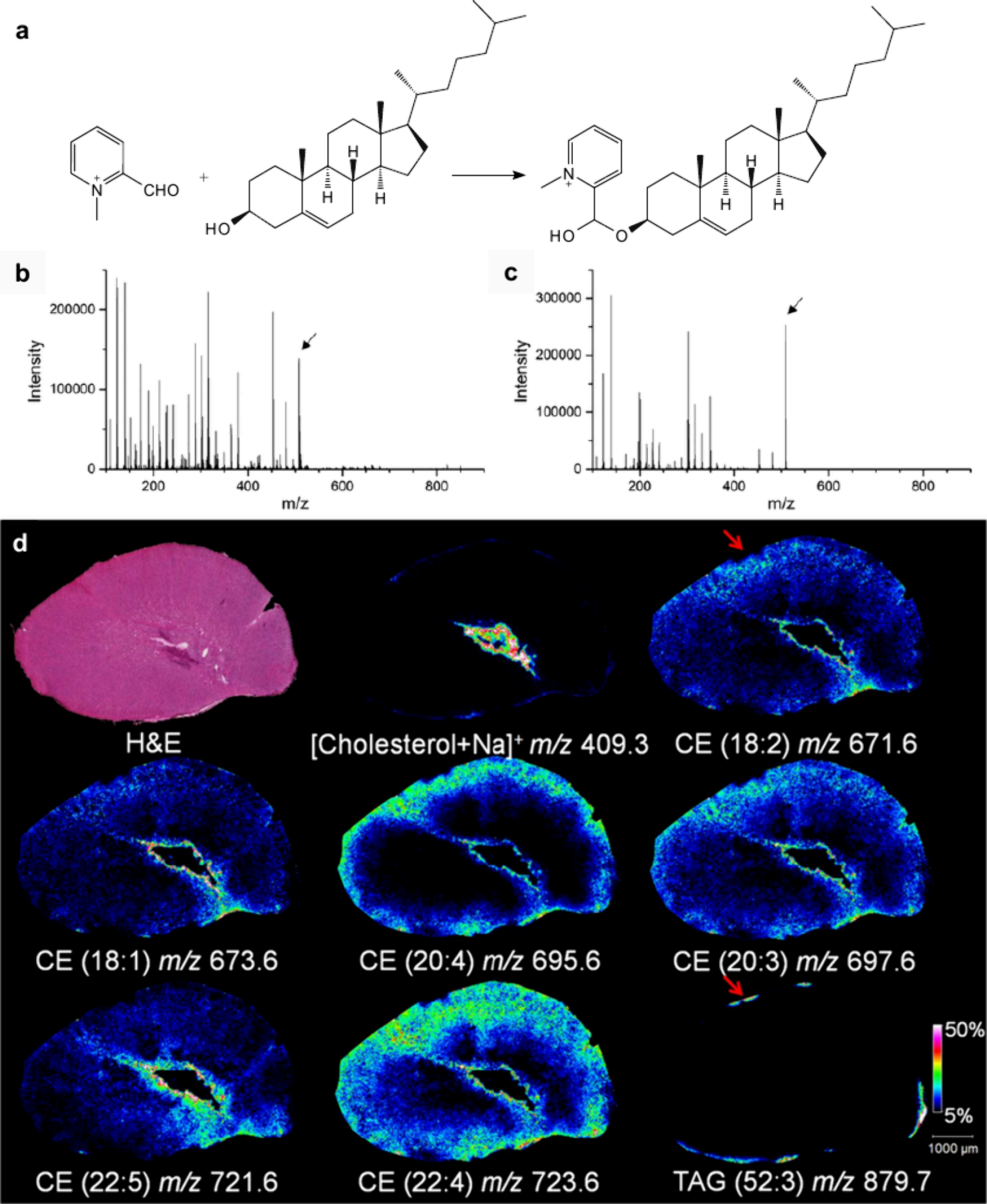
(a–c) MP2CA as a reactive matrix for cholesterol. (a) Reaction scheme. (b,c) MALDI-MS spectra of the MP2CA derivative of cholesterol (b) with or (c) without the assistance of the CHCA matrix. Arrows indicate the [M+MP2CA]^+^ peak of cholesterol. Reproduced with permission from ref 551. Copyright 2023 American Chemical Society. (d) CBS-Au-LDI-MSI with a lateral resolution of 25 μm, acquired from a rabbit AD tissue section. Based on exact mass and on-tissue MS/MS, intact cholesterol as a sodium adduct (*m*/*z* 409.3) along with several different CEs (*m*/*z* 671.5, 673.5, 695.5, 697.5, 721.5, and 723.5) and TAGs (*m*/*z* 879.7) were found to be the dominant signals within the adrenal gland. H&E staining of the next serial section is provided in the top left corner. The red arrow highlights one of the ion suppression sites of CEs by TAGs. Adapted and reproduced with permission from ref 1148. Copyright 2016 American Chemical Society.

##### 4.3.2.6 Lipid Oxidation Products

Oxidized phospholipids (oxPLs) are pivotal bioactive molecules involved in inflammation, infection, and immune responses, and abundant in body fluids, cells, and tissues.^1179^ The formation of oxPLs is initiated by the action of oxygen-centered free radicals, which abstract hydrogen atoms from unsaturated fatty acid chains, generating oxidatively modified products.^1179^ OxPLs encompass hundreds of different PL structures, and the field of “oxylipidomics” is currently garnering increasing interest. Most studies on oxidized lipids utilize ESI-MS,^1180^ as the ESI ionization process is softer than MALDI, thereby improving the detection of unstable compounds (such as easily dehydrated hydroperoxides).^1181^

Despite the growing interest in oxPLs, progress in improving MALDI matrices has been minimal. The use of ATT as a matrix, combined with specific dopants, can significantly increase the ionization efficiency of unstable OxPL species and improve their tolerance to alkali ions in biological samples. This approach facilitates the high-sensitivity detection of OxPLs in both positive and negative ion modes of MALDI-MS, particularly for trace amounts of OxPLs, such as OxPC, OxPE, and OxPS.^1182^ Given the relatively low concentrations of OxPLs in biological systems, chemical derivatization remains a convenient method for improving the detection of these compounds (*e.g.*, short-chain aldehydes). DNPH is a useful reactive MALDI matrix for detecting lipid oxidation products. DNPH reacts as a derivatizing agent with carbonyl compounds to convert aldehydes into stable crystalline hydrazones, facilitating their characterization by MALDI-MS under high vacuum conditions.^508^ Shigeri et al. investigated the reactions of 19 hydrazide and 14 hydrazine reagents with gaseous aldehydes (including formaldehyde, acetaldehyde, and propionaldehyde) and identified two hydrazide (2-hydroxybenzohydrazide and 3-HNAH) and two hydrazine reagents (2-hydrazinoquinoline and DNPH)-that significantly increased D/I efficiency in MALDI-MS.^511^ This represents the first report of using new reactive matrices for the direct detection of gaseous molecules by MALDI-MS.

Overall, the detection of lipid oxidation products using MALDI-TOF-MS necessitates significant enhancement, particularly in the context of MSI, where research on the detection of oxidized lipids remains limited. Currently, the sensitivity of the detection of peroxides by MALDI-TOF-MS is generally low or necessitates harsh oxidative conditions that differ from those in physiological environments.^1183^ Furthermore, investigations of the MSI of oxidized lipids are markedly lacking. Consequently, MALDI-MS detection of lipid oxidation products requires further refinement.

##### 4.3.2.7 Carbon–Carbon Double Bond (C=C) Positional Isomers

A comprehensive understanding of lipids necessitates the determination of carbon chain length, degree of unsaturation (*i.e.*, the number of carbon–carbon DBs (C=C)), and the specific positions of these DBs within the chain.^1184^ Recent research highlights the growing interest in lipid isomers, as certain isomers may be linked to various diseases,^172^ thereby underscoring the importance of identifying DB positions. However, determining the positions of DBs in unsaturated FAs remains a challenging task that often requires selective cleavage of the DBs (for example, through ozone^172^ or the PB reaction^1185^) to generate derivatives of carbonyl compounds for identification. The Wäldchen et al. team proposed using BPh as a MALDI matrix to achieve *in situ* derivatization and DB position assignment at pixel sizes as low as 15 μm through PB reactions with lipids, thereby facilitating lipid imaging in mouse brain sections and *Schistosoma mansoni* tegument.^529^ As depicted in Figure 98a, only n-9- and n-7-related fragment ions were detected in mouse cerebellar tissue, with distinct distribution patterns observed for these DB position isomers. Figure 98b illustrates that while n-7 and n-9 isomers are distributed across the surface of *S. mansoni*, the abundance of n-13 related fragment ions increases at the head and tail regions compared with the intermediate area of the parasite. These findings demonstrate that BPh facilitates the generation of a wide range of PB product ions from various PL classes, allowing the precise determination of DB positions. Additionally, a derivative of BPh, 2-benzoylpyridine (BzPy), was utilized as a multifunctional MALDI-MSI matrix to trace diagnostic lipid DB position fragment ions in mouse pancreatic tissue, achieving imaging with a lateral resolution of 7 μm (Figure 98c–e), which allowed for the identification of the islets of Langerhans associated with the upregulation and depletion of lipid isomers.^530^ Furthermore, Koktavá et al. successfully detected FAs and their C=C positional isomers in biological tissues by combining CeO_2_ and TiO_2_ nanopowder-based MALDI-MS techniques with off-line lipid derivatization by ozone and validated their method through MSI in mouse brains and human colorectal cancer tissues.^1184^

**Figure 98:**
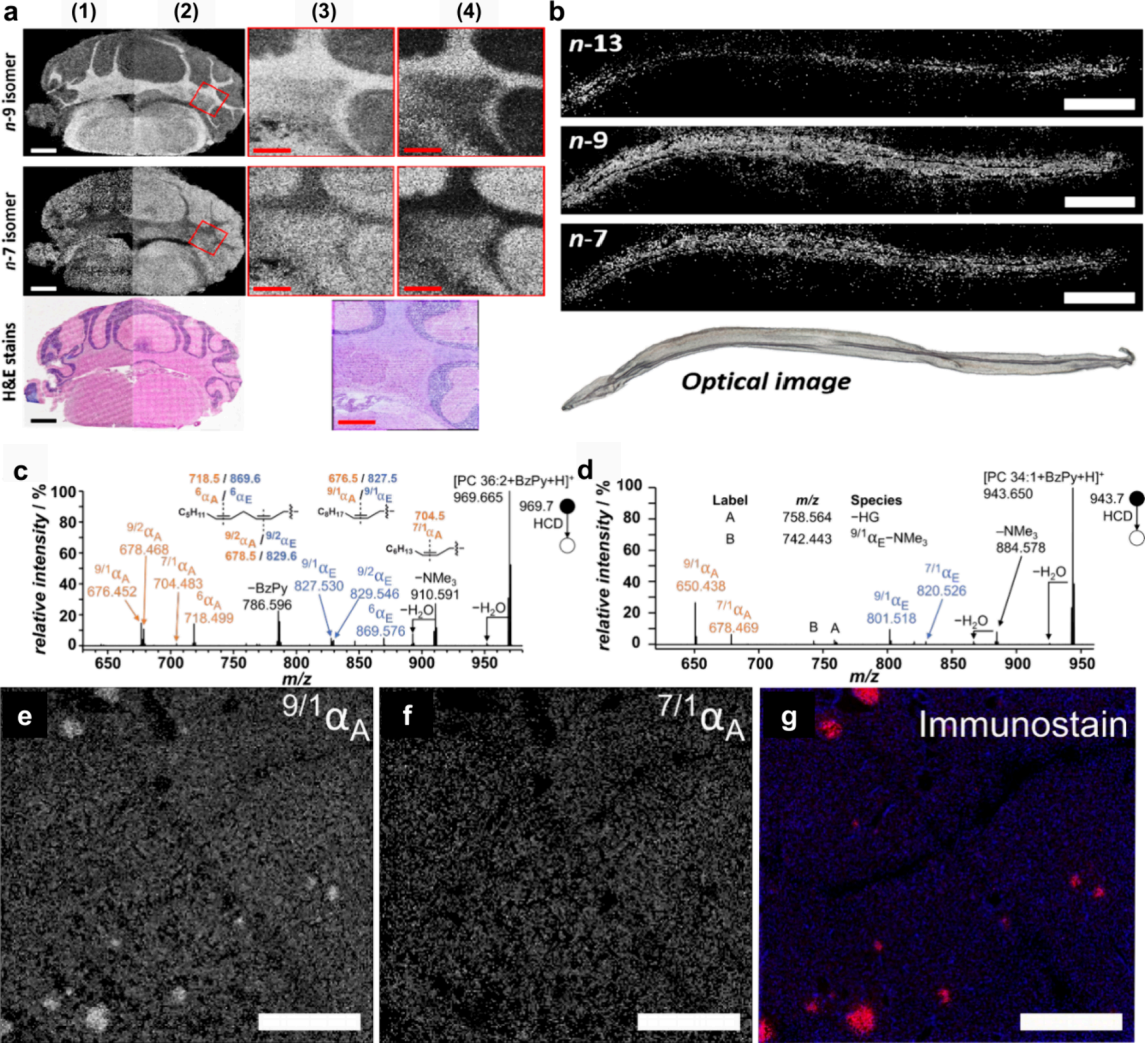
(a) MALDI-MS^2^ images of diagnostic retro-PB aldehyde fragment ions of (1) PC 36:1 and (2–4) PC 34:1 acquired from mouse cerebellum sections with (1,2) 25 μm and (3,4) 15 μm pixel sizes. TIC normalized (1–3) *n*-9, (1–3) *n*-7, and relative intensity (4) *n*-9/*n*-7 and (4) *n*-7/*n*-9 MS images. The red square in column b highlights the region measured in columns 3+4. (lower row) Microscope images of H&E stained tissue sections after MALDI-MS^2^ I. Scale bars are (1,2) 1 mm and (3,4) 400 μm. TIC-total ion current. (b) MALDI-MS^2^ images of diagnostic retro-PB aldehyde fragment ions of PC 34:1 acquired from male *S. mansoni* tegument with a 20 μm pixel size (TIC normalized). (lower row) Optical microscopy image before measurement. Scale bars: 1 mm. Adapted and reproduced with permission from ref 529. Copyright 2019 American Chemical Society. (c,d) MALDI-MS^2^ spectra of (c) [PC 36:2+BzPy+H]^+^ and (d) [PC 34:1+BzPy+H]^+^ desorbed from mouse cerebellum and mouse pancreas tissue, respectively. (e–g) TIC normalized MALDI-MS^2^ images of diagnostic fragment ions (e) ^9/1^αA (*m/z* 650.439) and (f) ^7/1^αA (*m*/*z* 678.470) of DB position isomers of [PC 34:1+BzPy+H]^+^ were acquired from mouse pancreas tissue with 10 μm pixel size. (g) Fluorescence microscopy image of post-MALDI-MSI-immunofluorescence stained pancreas tissue sections. Red areas in E indicate insulin-positive β-cells in Langerhans islets. Scale bars: 600 μm. Adapted and reproduced with permission from ref 530. Copyright 2020 American Chemical Society.

#### 4.3.3 Outlook

A primary objective of medical and clinical lipidomics research is a comprehensive analysis of lipid types and their biological roles in health and disease. Currently, the field of MALDI-MS lipid analysis remains vibrant and rapidly evolving. The discovery of new matrix materials, along with ongoing improvements in sensitivity and reproducibility, opens up possibilities for the establishment of new lipidomics methodologies. Certain lipids can only be effectively detected by MALDI-MS only when the most appropriate matrix is employed; however, the criteria for determining the “most suitable” matrix have yet to be fully elucidated. It appears that the choice of matrix is contingent upon the specific lipids being studied and the corresponding scientific questions. Although new matrices have been reported to enable a broader range of lipid analyses, their application in actual biological samples is still limited compared with that of classical matrices and has not yet gained widespread acceptance. We look forward to further expanding the application of MALDI-MS imaging in spatial lipidomics in the future, including the use of these new matrices. The application of MALDI-MS imaging in spatial lipidomics is likely to continue to expand in the future, including the use of these new matrices.

### 4.4 MALDI Matrices for Glycomic Analysis

Saccharides or carbohydrates are fundamental components in organisms, serving as primary energy sources and playing crucial roles in cell structure, biosynthesis, and cellular activity regulation.^1186^ Saccharides can be divided into monosaccharides, oligosaccharides, polysaccharides, and glycoconjugates. Monosaccharides represent the fundamental building blocks of sugars, including glucose and fructose, and predominantly function as the primary energy reservoir in biological systems. Oligosaccharides, which are composed of a limited number of monosaccharide units, participate in crucial cellular processes such as cell recognition and signal transduction. Polysaccharides, on the other hand, are derived from a substantial assembly of monosaccharides linked by glycosidic bonds, encompassing vital molecules such as starch, cellulose, and glycogen, primarily facilitating energy storage and structural reinforcement within biological matrices. Last, glycoconjugates are complexes formed by various sugar molecules (usually including monosaccharides, oligosaccharides, or polysaccharides) covalently linked to other types of biomolecules (such as proteins, lipids, or other biomacromolecules), and they perform which can exert their biological functions through complex molecular mechanisms.^1187^

Research on carbohydrates involves not only their functions in metabolic pathways but also their dynamic changes under various physiological and pathological conditions. Their metabolism involves the oxidation of glucose, generating energy through glycolysis and the citric acid cycle while providing precursor molecules for other metabolic pathways.^1188^ Modern glycobiology studies have shown that polysaccharide structures are abundant in higher animals and play specific roles in almost all biological regulatory pathways; moreover, abnormalities in the synthesis or metabolism of glycoconjugates are closely linked to hundreds of human diseases and conditions such as congenital glycosylation disorders and muscular dystrophy.^1189–1192^ The rapid advancement of metabolomics techniques has propelled research on glycobiology into the spotlight, enabling scientists to uncover how carbohydrate metabolism affects organismal changes, fostering novel perspectives for diagnosing and treating associated diseases.

Owing to the diversity and complexity of carbohydrates, the characterization of their chemical structures has always been challenging. Traditional methods combine chemical techniques and instrumental analyses such as high performance gel permeation chromatography (HPGPC), methylation with GC-MS, and nuclear magnetic resonance (NMR).^588^ However, the polar differences in carbohydrate molecules make selecting appropriate separation and MS analysis methods complex and time-consuming, and large sample amounts are often needed. Recently, MALDI-MS has emerged as a powerful tool for high-resolution and sensitive glycomic analysis through carbohydrate molecular profiling and imaging.^1193^ Studies have demonstrated its efficacy in detecting synthetic carbohydrates with high *m*/*z* values, with spectra primarily featuring single-charged ions that fragment to provide valuable insights into monosaccharides, linkage types, and sequences, facilitating the swift inference of carbohydrate composition and structure. On the other hand, compared with peptides or proteins, carbohydrates generally exhibit lower ionization efficiency owing to their abundant hydroxyl groups and the general absence of readily protonatable or deprotonatable functional groups. In selecting the ionization mode, it is essential to consider the chemical characteristics of the target carbohydrate analytes, specifically, carbohydrates containing acidic functional groups such as sulfate, carboxyl, or phosphate groups are more suitable for analysis in negative ion mode.^1194^ Consequently, in practical applications, systematically optimizing matrix selection on the basis of the structural characteristics of various carbohydrates is crucial for enhancing ionization efficiency, improving detection sensitivity, and increasing the quality of MALDI-MS. Currently, a variety of MALDI matrices have been successfully developed and widely applied for the detection and imaging of various carbohydrates. However, it is regrettable that there remains a relative scarcity of nontargeted matrices that encompass the entire glycomics spectrum. Therefore, a brief overview of commonly used carbohydrate detection matrices is provided here (Table S11), with more detailed descriptions of the practical applications of carbohydrate compounds in the subsequent sections.

The matrices commonly used in the MALDI-MS analysis of carbohydrates can be broadly classified into three major categories: solid matrices, ILMs, and inorganic matrices. Among traditional solid matrices, a few matrices such as DHB, CHCA, and THAP, are also commonly used for peptides, glycopeptides, and polysaccharides; other solid matrices used for carbohydrates include CMBT,^314^ gentisic acid,^438^ AMT,^323^ HABA,^354^ ISL,^373^ and HYNIC,^403^ among others. A common method to increase the ionization efficiency of natural oligosaccharides is the derivatization of reducing carbohydrates with aminoaromatic compounds. For example, the reaction of the 3-AQ matrix with the reducing end of oligosaccharides, combined with efficient MALDI-MS and targeted derivatization, has enabled the analysis of oligosaccharides.^501^ Furthermore, the reactive matrices that have been used for carbohydrate analysis include DHBB,^585^ 3-hydrazinobenzoic acid plus DHB (DHB/3HBA), and quinoline-3-carbohydrazide plus DHB (DHB/Q3CH).^1195^ However, a common drawback of using solid matrices is the tendency to form non-uniform crystals on the target surface, where uneven co-crystallization often leads to “hot spots” accompanied by issues of poor reproducibility between samples.

The use of ILs as matrices enhances sample homogeneity, with a growing number of ILs serving as effective MALDI matrices for carbohydrate analysis. These ionic liquids, derived from conventional acidic crystalline MALDI matrices and organic bases such as aliphatic or aromatic amines,^373^ not only aid in ionizing analytes but also facilitate the uniform co-crystallization of analyte–matrix blends. Commonly used ILMs for carbohydrate analysis include DHB-based matrices such as DHB/*N*-MA, DHB/*N*-EA, and BOA/DHB/Na,^588,589^ as well as 3-AQ-based matrices like 3-AQ/G_3_CA, and 3-AQ/CHCA,^660,1196^ and GTHAP,^614^ among others. Compared with traditional solid matrices, ILMs exhibit higher signal reproducibility, but show poorer performance for high-resolution and HMW compound applications.^1197^ Additionally, the use of inorganic matrices based on NMs provides a new approach for detecting LMW carbohydrates and improving sample homogeneity. NPs such as AuNPs,^695^ CNPs, and WCS NPs^847^ are typically used for monosaccharide, disaccharide, and oligosaccharide analysis and quantification, whereas composite materials such as UiO-66­(SH)_2_@Pd NPs^913^ and TpPa-1@Ag@GSH^935^ are typically used for oligosaccharide and glycopeptide analysis. Although inorganic matrices have advantages in small molecule analysis because of less interference from matrix-related ions, their limited universality restricts their widespread application. In conclusion, matrix selection is critical as each matrix has distinct advantages and disadvantages, with the suitability varying depending on the analyte type.

#### 4.4.1 MALDI Matrices for Targeted Carbohydrate Analysis

##### 4.4.1.1 Monosaccharides

Monosaccharides are the fundamental units of carbohydrates and consist of carbon chains with three or more carbon atoms, where each carbon atom, except one, bears a hydroxyl group. They can be classified as aldoses or ketoses, and owing to variations in the arrangement of atoms within the sugar molecules, many monosaccharides exist in various stereoisomeric forms.^1198^ The common symbols, abbreviations, and masses for some important monosaccharides are listed in Table 2, following the nomenclature guidelines set by the Nomenclature Committee of the Consortium for Functional Glycomics (CFG). Monosaccharides are widely present in living organisms and play significant roles in various biological processes such as energy metabolism^1199^ and signal transduction.^1200^ In recent years, an imbalance in monosaccharide metabolism has been closely linked to the occurrence of several diseases, including diabetes^1201^ and hereditary fructose intolerance,^1202^ reflecting the critical role of monosaccharides in maintaining normal physiological functions. Accurate measurement of the relative abundance and spatial distribution of monosaccharides in biological samples is crucial for understanding their biological roles, yet challenges remain in direct analysis using MALDI-MS because of their hydrophilicity and low ionization efficiency. Despite these challenges, researchers are actively working on developing efficient MALDI matrices to improve the detection of endogenous monosaccharides in tissues.

**Table 2:**
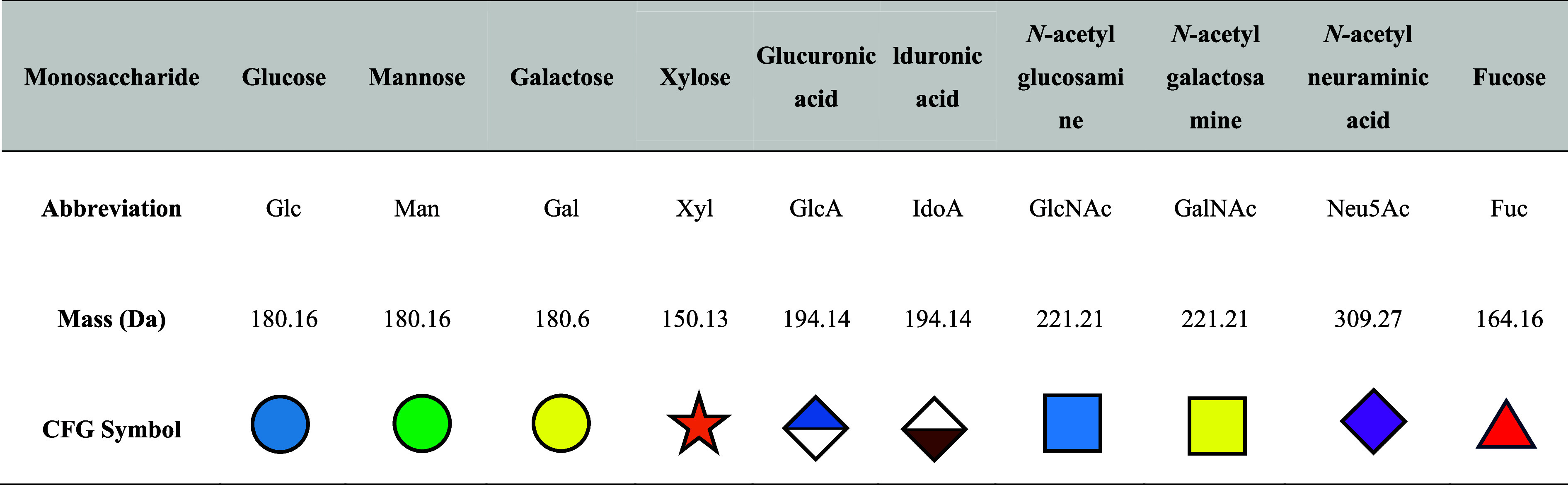
Abbreviations, Masses, and Symbols of Common Monosaccharides

Glucose is among the most important monosaccharides. Chen et al. reported that NEDC is suitable for use as a MALDI-MS matrix because of its excellent UV-absorption ability and high salt tolerance.^435^ The research team utilized this matrix in conjunction with microdialysis to measure glucose levels in rat brain microdialysates, and successfully detected glucose concentrations as low as 10 μM at a high ionic strength (126 mM NaCl) without sample purification, thereby demonstrating the sensitivity and rapidity of the NEDC in glucose detection. Additionally, He et al. proposed that NHHC is an ideal negative-ion MALDI matrix with high salt tolerance that is accordingly suitable for high-sensitivity glucose analysis.^438^ The research team successfully measured glucose levels in serum using the NHHC in negative ion mode, revealing its potential for rapid and high-throughput glucose detection, particularly for clinical blood glucose monitoring. Ma et al. prepared a series of MOFs using a one-pot method and post-synthetic modification strategies, with a maltose-functionalized MOF (MIL-101-maltose) serving as the matrix, and the results showed ultrahigh ionization efficiency and good dispersibility.^920^ The LDI-MS rapid detection platform established with this matrix features low sample consumption, short analysis times, and high salt tolerance, rendering it particularly suitable for rapid glucose monitoring in the serum of both diabetic patients and healthy controls, thereby facilitating accurate differentiation between the two groups. Furthermore, Wang et al. reported that WCS NPs (∼48 nm) can serve as an effective matrix for analyzing various molecules with high sensitivity and reproducibility, achieving a glucose detection limit of 1 pmol, which is suitable for quantitative urinary glucose determination using clinical samples from diabetic patients.^847^ It should be noted that this specific application relies on the defined clinical background. For the general identification of hexoses, MALDI-MS alone is insufficient due to isomer ambiguity, requiring complementary techniques like tandem MS, chromatography, or derivatization.

Fructose is another common monosaccharide that belongs to the ketohexose class and is an isomer of glucose. Naturally occurring fructose is primarily found in its free form as d-fructose, which is widely distributed in honey and fruits. Unlike glucose, fructose typically exists as an oily viscous liquid under natural conditions rather than in crystalline form, which gives it distinct physicochemical properties in various natural foods. Zhao et al. synthesized O-P and NC/G with excellent properties, including a large specific surface area, high electrical conductivity, and strong UV absorption properties.^815^ As a MALDI matrix, O-P and NC/G successfully enabled MALDI-MS analysis and quantitative detection of fructose and glucose in human serum and soft drinks without the need for standards.

Galactose is a significant monosaccharide classified into the aldose and hexose categories. It is found predominantly in dairy products and sugar beets, constitutes a key component of lactose in mammalian milk,^1203^ and is present in polysaccharides within biological tissues such as snails, frog eggs, and bovine lungs.^1204,1205^ In the brain and neural tissues, galactose often exists as a d-galactoside and serves as an essential component of various glycoproteins.^1206^ However, there is currently a dearth of reported MALDI matrices suitable for the detection and analysis of other free monosaccharides, including free galactose, in addition to glucose and fructose.

##### 4.4.1.2 Oligosaccharides

In the classification of oligosaccharides, they are traditionally defined as molecules comprising 2–10 monosaccharide units linked by glycosidic bonds. However, this classification is not strictly adhered to, as structures with a greater number of monosaccharide units can also be observed. While polysaccharides are typically defined as carbohydrate molecules whose degree of polymerization exceeds 20–25, this boundary is not rigid; indeed, molecules with a degree of polymerization of 25 or higher may be classified as oligosaccharides under certain circumstances.^1207^ This flexibility in definition accounts for the categorization of some LMW cellulose fragments as oligosaccharides. Recently, interest in oligosaccharide research has increased because of the rapid development of biotechnology and food science. Owing to the low ionization efficiency of oligosaccharides, analyzing them using MALDI-TOF MS remains a challenge.^403^ To address this, many researchers have developed various types of MALDI matrices to optimize the detection performance of oligosaccharides, increase ionization efficiency, and suppress background noise, thereby improving signal intensity and clarity.

Wang et al. demonstrated that THAP is an effective MALDI matrix for the analysis and detection of oligosaccharides in food.^1208^ By exploring the interplay between laser intensity, resolution, and the response factors of individual oligosaccharides, they developed a standard addition method that enabled the successful quantitative analysis of fructooligosaccharides. To increase the sensitivity and selectivity in the analysis of oligosaccharides via MALDI-MS, Jiao et al. introduced a novel matrix, HYNIC, which achieves a detection limit for maltoheptaose as low as 1 amol, five orders of magnitude lower than that of the traditional matrix, DHB.^403^ Furthermore, HYNIC exhibits notable selectivity for oligosaccharide ionization and allows direct detection in human serum without the need for pre-separation of proteins or peptides. Yang et al. reported that the flavonoid ISL, which has a chalcone structure, can serve as an excellent matrix for the MALDI-MS analysis of neutral oligosaccharides, exhibiting superior sensitivity, crystallinity, and salt resistance to those of traditional matrices such as DHB and THAP.^373^ Notably, the use of ISL in analyses revealed a robust quantitative linear relationship and excellent reproducibility within a concentration range of 1–100 pmol·L^–1^. Furthermore, ISL is effective across a broader concentration range, yielding strong signals even at lower matrix concentrations and laser intensities. These attributes underscore the promising potential of ISL for the rapid analysis of oligosaccharides. Moreover, Calvano et al. successfully employed MALDI-MS/MS technology to utilize the superbasic proton sponge TPPN for the structural characterization of neutral saccharides, cyclodextrins, and saccharide alditols.^441^ Owing to its inherent high basicity, TPPN effectively facilitates the deprotonation of neutral carbohydrates, providing a straightforward and efficient method for generating gas-phase [M–H]^−^ ions.

In addition to solid substrates, ILMs and inorganic matrices have been used in the MALDI-MS analysis of oligosaccharides. For example, Zhao et al. developed a series of nonderivatized ionic ILMs for carbohydrate MALDI analysis, with DHB/*N*-MA and DHB/*N*-EA demonstrating the best detection performance, achieving oligosaccharide detection limits as low as 10 fmol.^588^ They also implemented an internal standard strategy using DHB/*N*-MA to sensitively quantify fructooligosaccharide extracts. Additionally, the Mernie research team developed a method combining DHB@MNPs with TLC-MALDI-MS for the rapid separation, detection, and identification of oligosaccharides.^1209^ Without the need for chemical derivatization, this integrated platform successfully identified 25 different oligosaccharides from human milk through TLC and tandem MS visualization, revealing variations in oligosaccharide abundance during different breastfeeding periods.

For specific oligosaccharides characterized by low ionization efficiency and nonvolatility, derivatization is frequently essential to facilitate effective detection and analysis using MALDI matrices. Rohmer et al. developed a targeted derivatization method using a 3-AQ matrix that avoided the sample purification step, and enabled the measurement of Schiff bases in both positive and negative ion modes.^501^ This method allowed the detection of oligosaccharide derivative anions at concentrations as low as 1 fmol and provided detailed insights into their sequences and structures through enhanced PSD. By optimizing the reaction conditions, they achieved complete and reproducible derivatization of all the tested oligosaccharides, successfully identifying and differentiating two isomers (trifucosyllacto-*N*-hexaose (TFLNH) and trifucosyl-*para*-lacto-*N*-hexaose (TFpLNH)) in human milk samples.^501^ As shown in Figure 99a, both the oligosaccharides TFLNH and TFpLNH consist of one lactose and two *N*-acetyl-lactosamine units, and carry three fucose (Fuc) residues, but they differ in structure. MALDI-TOF/TOF MS^2^ analysis indicates that the fragmentation pattern in positive ion mode is dominated primarily by the loss of fucose residues, making it difficult to locate these residues in the context of structural characterization. In the derivatized 3-AQ-HMOS, all original fucoses are retained, significantly enhancing the acquisition of structural information. Fragmentation in negative ion mode has different characteristics, allowing sequence information to be obtained through consecutive C-cleavages. These results demonstrate that fragmentation analysis based on 3-AQ derivatives can effectively distinguish the structural features of these two isomers. Owing to its amino heterocyclic structure, AP is considered an ideal derivatization reagent to enhance the hydrophobicity of oligosaccharides and can be mixed with DHB to optimize the analysis of carbohydrates.^500^ Cai et al. utilized AP as a derivatization agent and co-matrix to significantly enhance the MALDI analysis of oligosaccharides through a convenient and complete nonreductive amination reaction, eliminating the need for desalting and allowing the effective evaluation of MS/MS fragmentation patterns.

**Figure 99:**
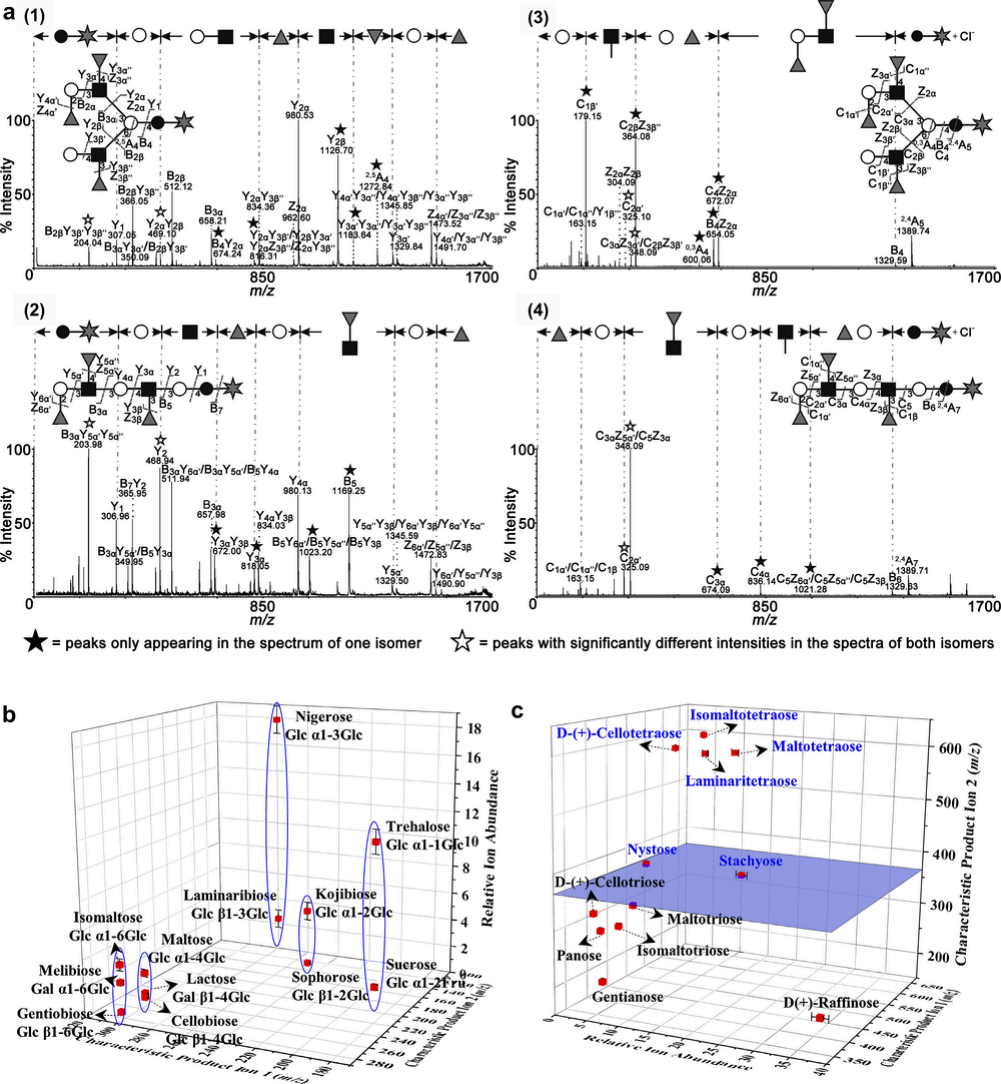
(a) Two human milk oligosaccharides (HMOS), trifucosyllacto-*N*-hexaose (TFLNH) and trifucosyl-*para*-lacto-*N*-hexaose (TFpLNH): MS^2^ spectra of 3-AQ-HMOS in positive and negative ion mode. On the left, MALDI-TOF/TOF MS^2^ spectra in positive ion mode are displayed for (1) 3-AQ-TFLNH and (2) 3-AQ-TFpLNH ([M+H]^+^ as the precursor). On the right, MALDI-TOF/TOF MS2 spectra in negative ion mode are shown for (3) 3-AQ-TFLNH and (4) 3-AQ-TFpLNH ([M+Cl]^−^ as the precursor). Adapted and reproduced with permission from ref 501. Copyright 2010 American Chemical Society. (b,c) 3D diagrams for (b) 12 disaccharides and (c) six trisaccharides and six tetrasaccharides. Adapted and reproduced with permission from ref 913. Copyright 2022 Elsevier BV.

The specific properties of oligosaccharides depend on the type of glycosidic bond and the specificity of the monosaccharides involved. For example, sucrose is formed by the dehydration condensation of glucose and fructose. Paek et al. investigated the MALDI-MS analysis of sucrose using a charcoal matrix with different cationizing agents (Li^+^, Na^+^, K^+^, Rb^+^, Ag^+^, and Cs^+^).^1210^ Their study revealed that compared with traditional matrices such as DHB and CHCA, an activated charcoal matrix significantly enhanced the cation adduction peaks of sucrose while concurrently reducing background interference. However, the use of the charcoal matrix also led to the cleavage of glycosidic bonds, generating fragment peaks whose degree of fragmentation was inversely related to the size of the cationizing agents, with smaller cations (Li^+^, Na^+^, Ag^+^) resulting in greater fragmentation and larger cations (K^+^, Rb^+^, Cs^+^) leading to less fragmentation.^1210^ Luo et al. employed UiO-66-(SH)_2_@Pd NPs as a matrix, significantly increasing the ionization efficiency of oligosaccharides for MALDI-MS analysis.^913^ By employing MALDI-LIFT-TOF/TOF technology, they successfully distinguished 24 isomers of oligosaccharides, including disaccharides, trisaccharides, and tetrasaccharides, while establishing relative quantification curves. The 3D plot in Figure 99b visually illustrates the differences among the studied oligosaccharide isomers, where the *x*- and *y*-axes represent the characteristic product ions of a specific oligosaccharide, and the *z*-axis indicates the relative ion abundance of the designated ion pairs. Furthermore, this method has been successfully applied to the identification and quantification of sucrose and maltose in Asian ginseng and American ginseng. Additionally, Lee et al. employed GO as a MALDI matrix to analyze seven disaccharide isomers by MALDI-MS and accurately determined the relative contents of maltose and sucrose in four honey samples.^808^

Overall, researchers are developing innovative MALDI matrices to enhance the detection and analysis of a broader range of oligosaccharides, thereby expanding the utility of MALDI-MS in oligosaccharide research across various sample types. By optimizing matrix selection, researchers can enhance the sensitivity, selectivity, and reproducibility of analyses, thereby promoting the in-depth study of oligosaccharide molecules in biological samples and other complex matrices.

##### 4.4.1.3 Polysaccharides

Polysaccharides are among the most common carbohydrates and play a critical role in living organisms.^1211^ Polysaccharides are typically formed by the dehydration condensation of 10 or more monosaccharides linked by glycosidic bonds, and their composition, molecular weight, and attachment methods significantly influence their structure, properties, and functional mechanisms.^1212^ Polysaccharides can be classified as natural or semisynthetic, where semisynthetic forms are obtained from natural forms through chemical or enzymatic modifications.^1213^ They exhibit various biological activities particularly noteworthy for their anticancer properties, as some can inhibit tumor growth and induce apoptosis in cancer cells.^1214^ Additionally, chemically modified polysaccharides have significant potential in drug delivery systems, effectively targeting therapeutic agents to tumor tissues.^1215^ Overall, polysaccharides possess inherent anticancer activity and can be further enhanced for cancer treatment through modifications and nanotechnology.

However, the quality precision and polydispersity of polysaccharides are relatively poor, making the qualitative identification of polymer ions challenging. To date, only a few matrices have been reported for detecting polysaccharides with molecular weights greater than 3,000 Da using MALDI-MS. The Mirza’s team reported that AMT can be used as a MALDI matrix for neutral carbohydrates, enabling the effective analysis of compounds such as β-cyclodextrin, maltodextrin, and dextran-5000.^323^ Compared with alkali metal cations, AMT has superior proton affinity, making it a more effective cationizing agent, and its uniform surface morphology further suggests better ion yield. THAP has been confirmed to be an efficient matrix suitable for the MALDI analysis of polysaccharides. By using THAP, it is possible to effectively detect linear neutral polysaccharides with molecular weights exceeding 47,000, providing high-quality spectra and excellent reproducibility, and it is also applicable for the analysis of dextran, polysialic acids, and glycoproteins.^309^ Additionally, López-García et al. developed a method using THAP as a matrix that eliminates the need for derivatization or purification pre-treatment to characterize the glucans extracted from various commercial mushroom supplement extracts.^1216^ They utilized THAP as the matrix, with DMSO and water as solvents. The resulting MALDI mass spectra are presented in Figure 100, *i.e.*, (a) Shiitake (*Lentinula edodes*), (b) Maitake (*Grifola frondosa*), (c) *Hericium erinaceus*, (d) *Cordyceps*, and (e) *Polyporus*. The study found that the MALDI polysaccharide spectra of five different fungal species were similar, all with a signal for the characteristic [glucan+Na]^+^ cation and with a mass difference of 16 Da between peaks, which is associated with β-(1→3)-glucan. Despite the similar qualitative analysis results, statistical analysis revealed differences in molecular weight among different species under the same experimental conditions.

**Figure 100:**
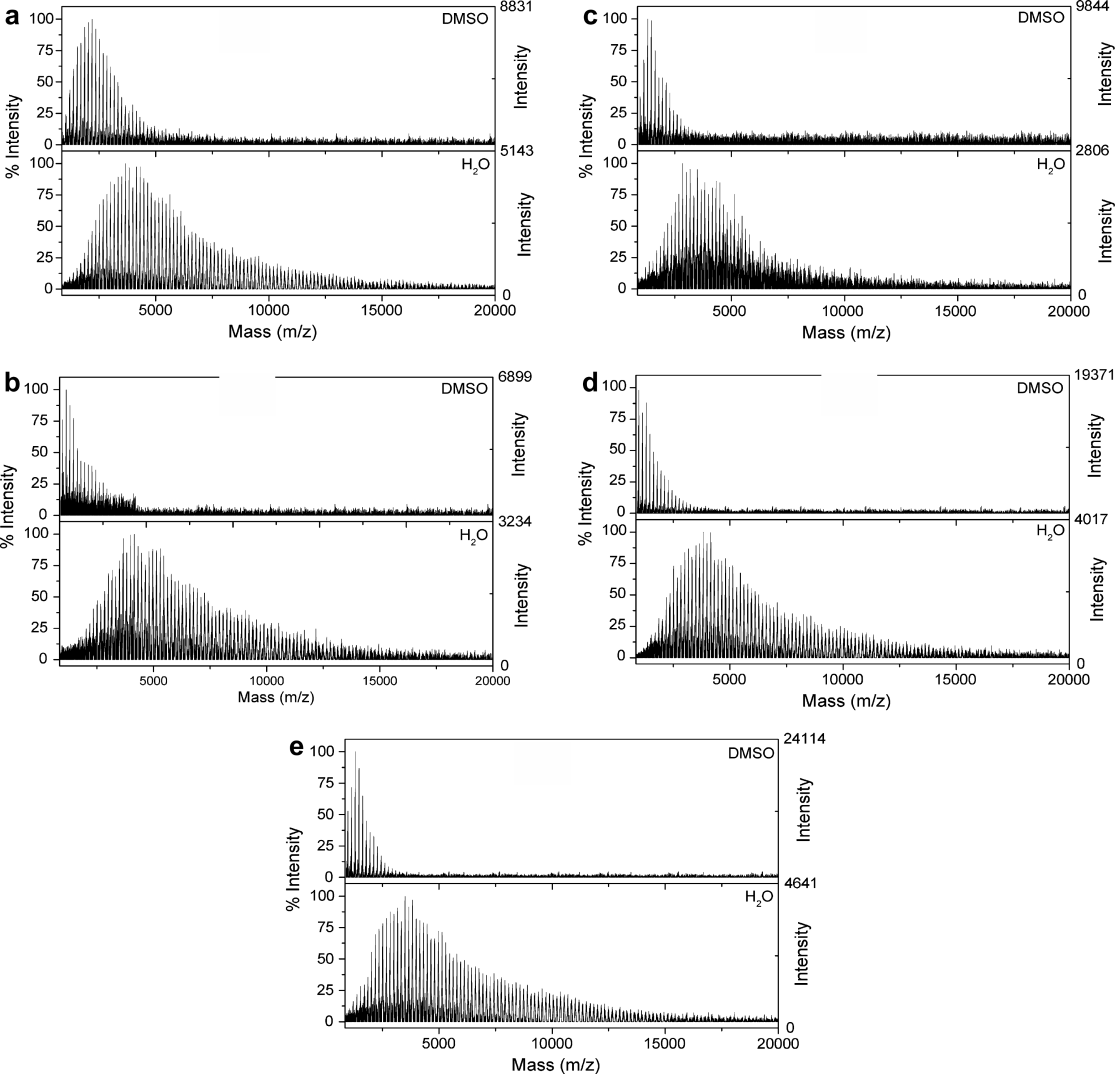
Comparison of MALDI profiles for commercial mushroom extracts acquired in linear mode using DMSO and water as sample solvents and THAP as the matrix; the sample concentration was 4,000 mg·L^–1^, and the sample:matrix ratio was 1:75. (a) M1 shiitake, (b) M2 maitake, (c) M3 *Hericium*, (d) M4 *Cordyceps*, (e) M5 *Polyporus*. Reproduced with permission from ref 1216. Copyright 2015 Elsevier Ltd.

To improve the ionization efficiency of MALDI-MS and simplify the preparation process, Lin et al. demonstrated that the reactive matrix 2-HQ is a novel MALDI matrix capable of sensitively detecting LMW polysaccharides in both positive and negative ion modes.^552^ The derivatization process, which involves the reaction of 2-HQ with the reducing end of polysaccharides to form stable hydrazones, significantly enhances ionization efficiency; moreover, the 2-HQ matrix exhibits good reproducibility, facilitating the quantitative analysis of various carbohydrates. Additionally, Wang et al. proposed a method of on-target derivatization using liquid matrix 3-AQ/CHCA to analyze the structures of oligosaccharides and polysaccharides via MALDI-TOF-MS and explored their fragmentation patterns.^1217^ In their study, polysaccharides (arabinoxylan, xylan, arabinogalactan, and dextran) were first depolymerized through enzymatic or acid hydrolysis. Following enzymatic treatment, xylan and arabinoxylan were successfully degraded into oligosaccharides. However, acid hydrolysis may lead to unwanted products; most of the arabinogalactan and dextran were already converted to oligosaccharides in this study when the hydrolysis time was extended to 2 hours or longer. Mass spectrometric analysis showed that targeted derivatization significantly improved the S/N ratio and sensitivity, making 3-AQ-labeled glycan ions easier to identify and providing important information for structural elucidation.^1217^

Overall, the development of novel MALDI matrices has significantly expanded the applicability of MALDI-MS in polysaccharide research by improving ionization efficiency, sensitivity, and reproducibility for reliable quantitative and qualitative analyses in complex biological samples.

##### 4.4.1.4 Glycoproteins

Protein glycosylation is an important post-translational modification of proteins that is widely present in all forms of life, ranging from prokaryotes to eukaryotes.^1218^ Glycosylation, defined as the covalent attachment of glycans to proteins, plays a crucial role in determining protein localization, activity, and function. This modification influences essential processes such as protein folding, stability, and transport, as well as key biological functions, including cell recognition, signal transduction, and immune responses, ultimately shaping cellular physiology and pathology.^1219,1220^ Aberrant glycosylation can result in protein dysfunction and disrupted cell signaling, potentially initiating a range of diseases, including cancer, neurodegenerative disorders, congenital disorders of glycosylation, infectious diseases, and chronic inflammatory responses.^1221,1222^

Glycoproteins, which are proteins modified by enzymatic glycosylation, represent a major class of glycoconjugates. The complexity and heterogeneity of glycoproteins primarily arise from the diversity of monosaccharide components and their linkage methods, differences in glycan branching structures, variations in glycosylation sites, and the diversity of glycosidic bonds between glycans and proteins. Based on the types of amino acid residues to which oligosaccharide chains are attached, glycosylation is mainly categorized into four types:^1223^ (i) *N*-glycosylation, where glycans are linked to asparagine (Asn) residues via *N*-acetylglucosamine, (ii) *O*-glycosylation, which attaches glycans to Ser or Thr residues, (iii) glycosylphosphatidylinositol anchors, which are attached to the carboxy-terminal of certain membrane-associated proteins, and (iv) *C*-glycosylation, which primarily occurs on tryptophan residues in certain membrane-associated and secretory proteins. In rare cases, other amino acid residues such as cysteine or lysine may also be glycosylated. Among these, *N*-glycosylation and *O*-glycosylation are the two most common forms, and both exhibit highly diverse branched 3D structures. *N*-Glycosylation involves the attachment of glycan chains to the amide group of asparagine residues, with the specific sequence being Asn-X-Ser or Asn-X-Thr, where X can be any amino acid other than proline.^1224,1225^ However, in bacteria, an acidic residue is required at position-2 (where position 0 indicates that the receptor is an asparagine), resulting in the extended sequence D/E-X-N-X-S/T.^1224^ *N*-Glycosylation is a highly conserved glycan modification that begins in the endoplasmic reticulum, is further processed in the Golgi apparatus, and is regulated by a series of glycosyltransferases and glycosidases.^1226^ After glycan attachment, glycoproteins fold correctly; therefore, *N*-glycosylation affects the tertiary structure and stability of glycoproteins.^1225,1227^ In contrast, *O*-glycosylation refers to the attachment of oligosaccharide chains via their oxygen atoms to the hydroxyl groups of amino acid residues, typically occurring on Ser or Thr residues.^1228^ *O*-glycosylation does not involve any specific motif and usually occurs after protein folding is complete. Each glycosylation site may possess multiple different glycan structures. For instance, at a single glycosylation site on the CD59 glycoprotein, more than 100 different glycan structures have been identified, demonstrating significant microheterogeneity.^1229^

The study of protein glycosylation is limited by the low abundance of glycosylation sites, and by the microheterogeneity and low ionization efficiency of glycopeptides, highlighting the need for sensitive and accurate characterization methods. Consequently, MALDI-MS has emerged as a pivotal technique for effectively characterizing glycoproteins. The analysis of glycoproteins is a significant application area of MALDI technology; however, due to its complexity, a complete analysis involves multiple steps.^347^ Enzymatic digestion, typically utilizing trypsin, facilitates the analysis of glycopeptides, allowing the elucidation of glycan structures and their attachment sites, while subsequent structural analysis of released glycans is conducted in detail. High-resolution instruments can effectively differentiate glycoproteins with fewer glycosylation sites and provide precise glycan mass measurements. Ultimately, the integration of data concerning glycan composition and linkage patterns is crucial for constructing a comprehensive structural profile. The specific sample preparation methods, such as the enrichment and purification of glycoproteins, glycopeptides, and released glycans, are not the main focus of this section; interested readers can refer to several excellent review articles for an in-depth understanding of the relevant techniques and methods.^1230,1231^ In this section, we explore the matrices related to glycoproteins, enzymatically digested glycopeptides, and glycans in MALDI-MS from the perspective of the matrix and their applications.

###### 4.4.1.4.1 *N*-Glycans

*N*-Glycans play various important biological roles in living organisms. However, the low abundance, high structural heterogeneity, poor ionization efficiency, and unevenness during co-crystallization of *N*-glycans with conventional matrices pose significant challenges for their qualitative and quantitative analysis using MALDI-MS.

In the MALDI-MS analysis of glycoproteins, AHB was one of the first matrices reported to effectively detect oligosaccharide mixtures released from glycoproteins.^345^ However, due to its lower ionization efficiency and multifunctionality compared to DHB, AHB was quickly replaced by the latter.^346^ Dunne et al. successfully detected and imaged *N*-glycans in FFPE tissues using DHB as a MALDI matrix through MALDI-MSI technology, demonstrating the potential of DHB for *N*-glycan analysis in complex biological tissue samples.^1018^ Malaker et al. performed MALDI imaging analysis of *N*-glycans in canine glioma tissue sections using a DHB matrix (Figure 101a–c), and reported that a sialylated glycan (HexNAc4-Hex5-NeuAc2) was significantly upregulated in the necrotic regions of high-grade gliomas.^1232^ They effectively mapped the distribution of this glycan in both tumor and necrotic areas, providing vital spatial insights into glycoproteinome changes. Additionally, the researchers identified the glycoprotein linked to this glycan chain, haptoglobin, by employing spatially resolved glycoproteomics technology on adjacent tissue sections, thereby establishing a direct connection between glycan imaging and complete glycopeptide identification. Recently, Urakami et al. developed several matrices containing mixed components, such as ANI/DHB/Na^1233^ and DAN/DHB/Na,^1234^ to enhance the direct analysis of *N*-glycans on glycoproteins. They improved the direct analysis of *N*-glycans on glycoproteins using the MALDI-ISD method to achieve practical MALDI detection. These doped matrices enhanced glycosidic bond degradation under high laser energy, improving sensitivity and reproducibility in the direct analysis of polysaccharide fragments, thereby making them suitable for use as biomarkers in biological studies. In addition, Zhao et al. proposed a parallel targeting derivatization strategy based on two combinations of matrices, DHB/3HBA and DHB/Q3CH, for the rapid detection of reducing *N*-glycans.^1195^ These matrices exhibit high derivatization efficiency and significantly improved ionization efficiency, allowing precise MS and MS^2^ calibration in dual-polarity mode. This method was successfully applied to the assessment of serum *N*-glycans from patients with hepatocellular carcinoma, demonstrating its potential in the discovery of clinical biomarkers while simultaneously supporting rapid and automated *N*-glycan identification with a good linear relationship (*R* ^2^ > 0.998) and accuracy (RSD ≤ 10%).^1195^

**Figure 101:**
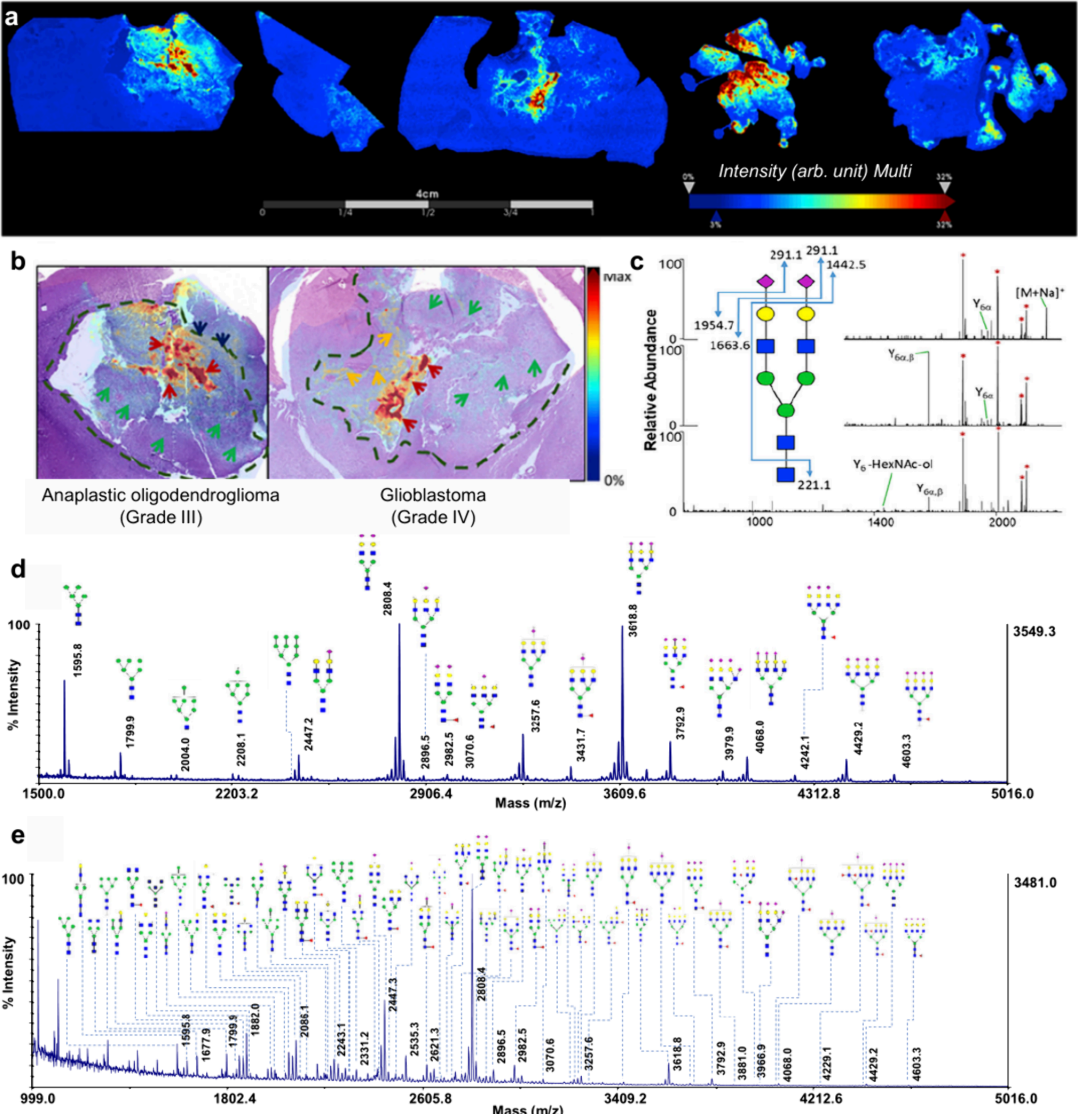
(a–c) MALDI-MSI of *N*-glycans using DHB as a matrix. (a) Summed ion images of Na^+^ and K^+^ adducts of HexNAc4-Hex5-NeuAc2 on canine glioma biopsies. (b) Superposition of MALDI-MSI glycan images with H&E-stained adjacent sections. Arrows and dashed lines indicate regions annotated by the pathologist. Legend: red, green, yellow, and dark blue arrows represent necrotic regions, tumor regions, pseudoglomerular vessels, and pseudopalisading necroses, respectively. Dashed lines indicate tumor margins. (c) MS^n^ spectra of HexNAc4-Hex5-NeuAc2 confirming its structure. Adapted and reproduced with permission from ref 1232. Copyright 2021 Elsevier Ltd. (d,e) MALDI-TOF-MS profiles of purified and extracted permethylated glycans using CNPs, derived from (d) 0.8 μg mixture of RNase B, fetuin, and AGP at a 1:2:5 weight ratio and (e) 10 μL of human blood serum. Adapted and reproduced with permission from ref 849. Copyright 2018 American Society for Mass Spectrometry.

HABA has been proposed as an efficient MALDI matrix suitable for the analysis of peptides, proteins, and glycoproteins, demonstrating excellent sensitivity and reproducibility, especially better sensitivity and mass resolution for larger proteins and glycoproteins; however, it has poor solubility in water.^354^ Additionally, CHCA is also a commonly used matrix in MALDI-MS. Stanback et al. effectively utilized CHCA in MALDI-MSI to visualize *N*-glycans in fresh frozen tissues, *i.e.*, *N*-glycans from the brain tissues of wild-type mice.^1235^ Liao et al. developed a novel reactive matrix for MALDI, namely, 3-CACA, which co-crystallizes with the conventional matrix CHCA to significantly enhance derivatization efficiency. Their study demonstrated that this 3-CACA/CHCA matrix achieved exceptionally low detection limits, reaching femtomole levels in *N*-glycan analysis, while simultaneously exhibiting excellent sensitivity, qualitative performance, and robust linear quantification capabilities (*R* ^2^ > 0.998).^282^ Wang et al. designed and synthesized a reactive MALDI matrix, 2-HTA, which co-crystallizes uniformly with oligosaccharides and facilitates the generation of high-intensity deprotonated ions in negative ion mode.^518^ By combining 2-HTA with SA (SA/2-HTA), the research team established a rapid high-throughput method for the targeted analysis of free oligosaccharides, thereby demonstrating exceptional detection sensitivity and linearity (*R* ^2^ > 0.999), with detection limits at the femtomole level. This method was successfully applied to the quantitative analysis of *N*-glycans in various peach *Prunus persica* (L.) Batsch cultivars, suggesting the potential of *N*-glycans as biomarkers for food-related allergies.^518^ Ullmer et al. investigated a novel ILM, specifically GTHAP, for its application in MALDI-MS analysis of glycopeptides and glycans.^614^ The experimental results indicate that GTHAP effectively overcomes the ionization suppression of carbohydrates in the presence of peptides, thereby significantly increasing the signal intensity. Comparisons with the THAP matrix reveal that GTHAP demonstrates superior performance in analyzing glycoprotein digests and *N*-glycans, particularly in glycosylation analysis, which in turn results in lower levels of metastable decay.

Banazadeh et al. investigated GNs and CNPs as MALDI matrices and co-matrices (with DHB) and found that they significantly enhanced the signal intensity of *N*-glycans in various biological samples and facilitated the generation of ISD fragments, offering detailed insights into glycosidic and cross-ring cleavages.^849^ These NMs displayed good salt tolerance in high-salt solutions, improving the uniformity and reproducibility of DHB crystals, and were effectively utilized for the glycan profiling of three glycoproteins mixture (Figure 101d) and human serum (Figure 101e) by adjusting their concentrations to control the extent of cleavage. Furthermore, Ma et al. employed MIL-101­(NH_2_)@Au-Cys as a MALDI-MS matrix, achieving detection limits at the femtomole level and good intersample reproducibility.^919^ This material’s high surface area, abundance of hydrophilic groups, and size-exclusion effect make it a highly sensitive and selective adsorbent for *N*-glycan enrichment, opening new avenues for the design of multifunctional probes.

###### 4.4.1.4.2 *O*-Glycans

In recent years, an increasing body of research has demonstrated that abnormalities in *O*-glycosylation are closely linked to the occurrence of various diseases.^1220,1236^ and are recognized as potential biomarkers in clinical settings.^1237^ Moreover, *O*-glycosylation is critical for the adaptation of polar fish to low-temperature environments.^1238^ A thorough exploration of the molecular mechanisms of *O*-glycosylation and its biological significance holds important scientific value for understanding cell communication, signal transduction, and disease treatment.

As a gold standard matrix for glycan analysis, DHB has also been widely used in *O*-glycan research.^1239^ However, organic salt-type matrices often introduce matrix-derived noise in MALDI-MS, making direct analysis of *O*-glycans on small glycoproteins with a molecular weight less than 1,000 difficult. To address this problem, the Urakami team reported a new matrix combination, AHB/Na, that allows the glycan-selective MALDI-ISD analysis of intact *O*-linked glycopeptides and glycoproteins without the need for preprocessing.^349^ AHB/Na promotes the release of free *O*-glycans through the β-elimination of Ser/Thr residues, enhancing the resolution and S/N ratio of ISD ion peaks. This method can detect glycan patterns from mixtures without digestion or purification, such as multiple *O*-linked glycoproteins in gastric mucin (Figure 102a), and perform pseudo-MS^3^ analysis on ISD ions derived from *O*-glycans to provide more detailed structural information.^349^ Barada and Hinou evaluated and optimized a novel solid ion matrix BOA/DHB/Na, which successfully labeled the reducing ends of glycans by forming a uniform solid salt mixture with glycans on the MALDI target plate.^589^ To validate this method, the researchers performed base excision and hydrophilic interaction chromatography (HILIC) to separate the released polysaccharides, followed by MALDI-TOF-MS analysis. The experimental design and results are illustrated in Figure 102b,c. This matrix exhibited excellent aggregation performance and decay suppression, significantly enhancing ionization efficiency, and showed good sensitivity and peak pattern reproducibility when analyzing *O*-glycans from porcine stomach mucin. Pouria et al. utilized THAP-ammonium citrate as a matrix to analyze a pool of *O*-glycopeptides from human serum IgA1 that had been digested, reduced, and alkylated by trypsin, using MALDI-TOF-MS in positive ion mode.^1240^ Serum IgA1 samples from both healthy individuals and patients with disease exhibited identical glycopeptide amino acid sequences, but variations in glycan substitutions resulted in distinct spectral baseline fragmentation and demonstrated reproducibility in the relative quantification of different glycan types. Selman et al. assessed ClCCA as a matrix for MALDI-TOF-MS analysis of unstable salivary trypsin *N*-glycopeptides and released *N*- and *O*-glycans, finding that it demonstrated strong analytical performance in negative ion mode.^281^ ClCCA facilitated a uniform microcrystal distribution and exhibited high reproducibility, thereby streamlining automated measurements while enhancing resolution and mass accuracy. Additionally, when PMP-labeled *O*-glycans were analyzed in reflective positive ion mode with ClCCA, their integrity significantly surpassed that of *O*-glycans labeled with DHB and CHCA.^281^

**Figure 102:**
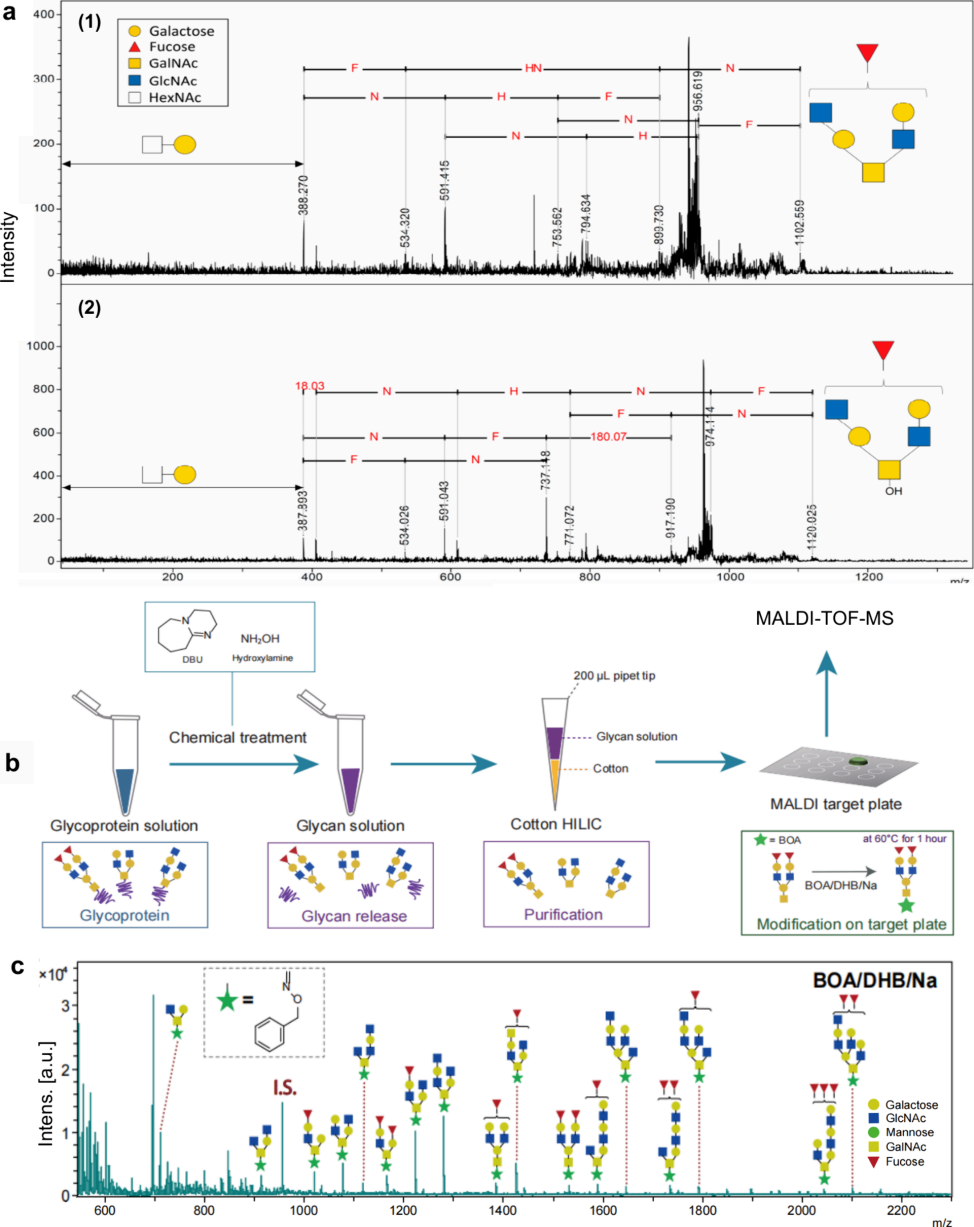
(a) Pseudo-Ms^3^ spectrum of PS (1 μg·μL^–1^) with AHB/Na (1) at *m*/*z* 1102 for the precursor ion and (2) at *m*/*z* 1120 for the precursor ion. *N*,*N*-Acetyl hexosamine; H, hexose; F, fucose (deoxyhexose). Adapted and reproduced with permission from ref 349. Copyright 2023 The Authors. Licensee MDPI, Basel, Switzerland. (b,c) *O*-Linked glycan profiles obtained from mucins from the porcine stomach (MPS). (b) Workflow for profiling of *O*-linked glycans using a pure BOA matrix. IS, internal standard; DBU, 1,8-diazabicyclo[5.4.0]­undec-7-ene; HILIC, hydrophilic interaction chromatography. (c) MALDI-TOF-MS spectra of mucin from MPS with the BOA/DHB/Na matrix. Compositions of the *O*-linked glycan structure were assigned by GlycoMod using experimental masses (mass tolerance 0.2 Da). Adapted and reproduced with permission from ref 589. Copyright 2022 The Authors. Licensee MDPI, Basel, Switzerland.

##### 4.4.1.5 Proteoglycans

Proteoglycans are a significant class of glycoconjugates and represent a specialized subclass of glycoproteins. As essential components of the extracellular matrix and basement membranes, proteoglycans not only provide structural support but also regulate numerous biological processes, including morphogenesis, cell growth, and cell differentiation.^1241^ A defining structural feature of proteoglycans is the covalent attachment of linear, highly sulfated carbohydrate chains, known as glycosaminoglycans (GAGs), to specific serine residues on the core protein.^1242^ This linkage occurs via a characteristic tetrasaccharide bridge (GlcAβ1-3Galβ1-3Galβ1-4Xylβ1-O-Ser). GAGs are linear polysaccharides with strongly acidic properties and represent a prominent family of sulfated carbohydrates that are widely distributed in the extracellular matrix and proteoglycan complexes.^1243^ Structurally, GAGs consist of repeating disaccharide units, typically composed of a hexosamine and a hexuronic acid (most commonly glucuronic acid). On the basis of the type of hexosamine, GAGs are classified into two main categories:^1244^ glucosaminoglycans, including keratan sulfate (KS), heparin (HP), hyaluronic acid (HA), and heparan sulfate (HS), and galactosaminoglycans, such as chondroitin sulfate (CS) and dermatan sulfate (DS). The most notable biological properties of GAGs arise from their extensive sulfation, which imparts a strong negative charge, enabling them to bind to cationic sites on proteins through specific sequence recognition or through electrostatic interactions.^1245^ These molecules are critically involved in regulating key biological events, such as the localization of cell surface receptors, proteolytic processes, tumor angiogenesis and metastasis, and the oligomerization of growth factors.

Intact GAG polysaccharides are not amenable to direct mass spectrometric analysis due to their extremely low volatility. This property stems from their high molecular weight and the presence of highly charged functional groups, particularly sulfate residues, which severely impede evaporation under vacuum conditions. Consequently, it is often necessary to reduce their molecular weight through controlled enzymatic digestion or chemical digestion. Enzymatic digestion can be achieved via exolytic cleavage by lyases and hydrolytic cleavage by glycosidases.^1246^ Chemical degradation methods include acid or base hydrolysis, oxidative degradation, and radical degradation.^1247^ It is essential to recognize that the enzymes employed for polysaccharide degradation may be inhibited by oversulfated GAGs, preventing the breakdown of those that contain an excess of sulfate residues or exhibit unusual sulfation patterns. Furthermore, chemical degradation is typically accompanied by site-specific reactions, particularly the cleavage of sulfate esters and/or amide linkages, complicating subsequent analyses.^1248^ Therefore, the appropriate depolymerization method must be carefully selected. Despite these strategies, the MALDI-MS analysis of GAG oligosaccharide fragments and other sulfated oligosaccharides remains challenging. These sulfated carbohydrates are prone to extensive desulfation (loss of SO_3_, ∼80 Da) under gas-phase conditions, compounded by their inherent structural heterogeneity, high polarity, and the insufficient ionization efficiency of conventional MALDI matrices.^613,1249^ Together, these factors complicate spectral interpretation and introduce biases in molecular weight determination, severely limiting the application of MALDI-MS for the high-precision characterization of such sulfated carbohydrates.

In previous MALDI-MS investigations, particularly from the perspective of matrix design, numerous matrices have been proposed to address the analytical challenges associated with sulfated carbohydrates. Initially, the use of “cold” matrices aimed at suppressing ISD has been explored. Conventional matrices such as DHB were widely employed for sulfated oligosaccharides.^1246^ For instance, Nimptsch et al. demonstrated that while the sensitivity of detecting highly acidic oligosaccharides in positive ion mode using DHB was slightly reduced, it did significantly minimize the loss of sulfate residues, thereby enhancing the reliability of the analysis.^1250^ Additionally, Dai et al. identified coumarin 120 as an ideal matrix for analyzing monosulfated disaccharides and reported a notable improvement in the ionization efficiency of sulfated trisaccharides and tetrasaccharides containing *N*-acetylneuraminic acid when used in conjunction with ATT, ultimately achieving subpicomole sensitivity in MALDI-MS for a variety of sulfated oligosaccharides.^1251^ Nonami et al. reported that β-carboline alkaloids (*e.g.*, NRM) served as an effective matrix for the MALDI-MS detection of sulfated oligosaccharides, such as λ-carrageenans.^451^ Furthermore, through rational design of the stereochemical structure of cinnamic acid derivatives, Salum et al. developed a novel “cold” matrix, Z-SA, that efficiently detects sulfate oligosaccharides, including neocarratetraose 41,43-disulfate disodium salt, neocarrahexaose 41,43,45-trisulfate trisodium salt, and neocarraoctaose 41,43,45,47-tetrasulfate tetrasodium salt, in negative ion mode.^269^ This matrix nearly completely suppresses the dissociation of sulfate groups, resulting in excellent ionization stability and signal integrity.

Significant advancements have been made in the application of ILMs for MALDI-MS analysis of sulfated carbohydrates. Laremore et al. demonstrated that ILMs, specifically ImCHCA and DHBB, enable the robust detection of highly sulfated oligosaccharides without prior complexation.^603^ Their sample preparation protocol is straightforward, involving the simple mixing of an aqueous analyte solution with the ILMs. This approach enabled the analysis of minimal sample quantities while yielding uniformly distributed spots on the MALDI target, successfully detecting picomolar amounts of disaccharides, octasulfated sucrose, and the sodium salt of octasulfated pentasaccharide (Arixtra). Furthermore, the same team developed a novel ILM, G_2_CHCA, that facilitates direct MALDI-MS analysis of DS and CS oligosaccharides.^608^ This matrix is suitable for the direct detection of purified polysulfated oligosaccharides (up to decasaccharides) as well as complex glycan mixtures by MALDI-MS, efficiently ionizing unsaturated, nonacidic polysulfated/multicarboxylated oligosaccharides, which typically exist in a sodium salt form. The study revealed that G_2_CHCA exhibits excellent tolerance to buffer salts and enables real-time monitoring of the partial enzymatic digestion of chondroitin 4-sulfate. The mass spectrum obtained in positive ion mode was significantly superior to that in negative ion mode, with the spectra of DS and CS oligosaccharides predominantly consisting of sodium-ionized molecular species, exhibiting minimal fragmentation peaks.^608^ Fukuyama et al. optimized an ILM for the MALDI-MS analysis of sulfated, acylated, and neutral oligosaccharides and glycans.^609^ The newly synthesized G_3_CA and the existing G_2_CHCA demonstrated high sensitivity for detecting these compounds in both positive and negative ion dual-polarity modes. Notably, the use of G_3_CA effectively suppressed the dissociation of sulfate groups, markedly enhancing the detection sensitivity of sulfated oligosaccharides and both sialylated and neutral oligosaccharides, achieving sensitivities of 1 femtomole in both ion modes. Additionally, Przybylski et al. synthesized two novel ILMs based on HABA (HABA/TMG and HABA/SPM) and successfully applied them to the MALDI-MS analysis of HP and HS.^613^ Compared with the conventional matrix CHCA, HABA promotes the cation binding of oligosaccharide sulfate groups, primarily by limiting the cleavage of N–S and O–S bonds, thus reducing desulfation rates. The use of counterions such as TMG and spermidine further curtails decomposition; spermidine, in particular, reduces alkali metal exchange and improves signal-to-noise ratios, peak intensities, and spectral profiles, although its utility diminishes for sulfated oligosaccharides larger than octamers.^613^ Additionally, Schmidt et al. reported that CHCA·nHo ILMs (norharmane combined with CHCA) perform exceptionally well for LMW carbohydrates in both positive and negative ion modes (linear and reflective modes).^610^ Although sulfate dissociation was not fully eliminated, a prominent [M–Na]^−^ signal was observed for sulfated oligosaccharides such as neocarratetraose-41,43-disulfate disodium salt in negative-ion mode.

Compared with UV-MALDI-MS, IR-MALDI-MS provides milder D/I conditions, thereby better preserving labile molecular structures. Witt et al. investigated the use of glycerol and water ice as IR-MALDI matrices for the structural characterization of unstable GAGs.^1241^ Their findings revealed that GAG oligosaccharides with varying sulfation levels underwent ionization primarily as intact deprotonated singly charged ions, though doubly and even triply charged disaccharide ions could also be observed depending on sulfation degree, accompanied by minimal loss of sulfate groups. Notably, in tandem MS experiments, the collision-induced dissociation of these multiply deprotonated, sodiated GAG ions yielded informative fragment spectra that allowed precise localization of sulfate group positions. This study particularly highlights that water ice as a matrix enables highly effective and gentle IR-MALDI-MS analysis of highly sulfated GAG disaccharides.^1241^

##### 4.4.1.6 Glycolipids

Glycolipids are a class of glycoconjugates and are glycosyl derivatives of lipids that serve as key components of cell membranes and signaling molecules throughout life. These amphipathic molecules consist of hydrophilic sugar groups linked to hydrophobic regions via glycosidic bonds, contributing to membrane structure and function. They exhibit a diverse range of forms, from simple monosaccharides to complex polysaccharide chains, including glycoglycerolipids and GSLs, among others. Despite their structural variations, they all contain at least one sugar moiety linked to a lipid portion via a glycosidic bond, possess one or two fatty acyl residues, and lack phosphate groups. Glycolipids, particularly GSLs, are vital for cell surface interactions and play a significant role in immune responses,^1252^ as well as being implicated in various diseases, including cancer^1253^ and infectious diseases.^1254^ Recent research has highlighted the diverse biological functions of glycolipids and their potential applications in disease research, drug development, and biotechnology, identifying them as promising targets for cancer immunotherapy.^1255^

MALDI-MS and MALDI-MSI techniques have been extensively validated as powerful tools for the sensitive and efficient detection and analysis of glycolipids, providing critical insights into their molecular structures and spatial distribution within complex biological samples. Various matrices have shown strong performance in glycolipid analysis; for example, DHB is widely used as a MALDI matrix for glycolipid analysis because of its high ionization efficiency, strong signal intensity, versatility, and enhanced ionization efficiency in positive ion mode.^1256–1258^ Additionally, the 9-AA matrix, known for its high ionization efficiency and excellent affinity for acidic glycolipids, effectively enhances the signals of acidic glycolipids in MALDI-MS, especially in negative ion mode. Consequently, in negative ion detection mode, 9-AA is regarded as the preferred matrix for detecting certain acidic glycolipids, such as acidic GSLs.^1259,1260^ The combination of exoglycosidase digestion with MALDI-TOF MS has been shown to be an effective method for characterizing picomole quantities of glycoconjugates and oligosaccharide structures. Geyer et al. developed an efficient sample preparation method using ATT as a matrix coupled with a low concentration of volatile buffer such as ammonium acetate to maintain exoglycosidase activity, enabling rapid analysis of carbohydrates and their mixtures without complex desalting.^1261^ This approach provides high sensitivity and enables the direct analysis of nonderivatized polysaccharides, glycopeptides, and glycolipids, circumventing the need for complex desalting or sample separation procedures, thereby streamlining functional studies of glycoconjugates. CMBT is an effective MALDI-MS matrix that enhances the analysis of peptides, LMW proteins, and glycolipids through uniform crystallization, resulting in superior reproducibility and consistent ion generation compared to the traditional matrix DHB.^314^ CMBT demonstrates good sensitivity and resolution when analyzing gangliosides, particularly in negative ion mode, effectively reducing the loss of sialic acid residues. Additionally, compared with DHB, CMBT performs better in the analysis of complex carbohydrates, such as peptides extracted from the structural component peptidoglycan of bacterial cell walls. Itonori et al. employed MALDI-TOF MS technology with CHCA as a matrix to analyze differences in purified GSLs from the larvae and pupae of the silkworm (*Bombyx mori*).^1262^ The results indicated that the GSLs from the larvae contained four ceramide species, while those from the pupae contained only two. Their structural analysis also revealed several novel GSL molecules and highlighted significant differences in ceramide composition between the two developmental stages. Furthermore, Caughlin et al. developed a sublimation method based on DAN, which successfully visualized gangliosides in rat brains through MALDI–MSI, optimizing its use for the precise localization and detection of ganglioside spatial distribution.^1263^ Goto-Inoue et al. developed and tested a novel matrix utilizing alkylamine-modified AuNPs for the high-sensitivity visualization of the distribution of GSLs in biological tissue sections.^708^ This approach demonstrated an approximately 20-fold increase in sensitivity to sulfatides in mouse brain slices compared with the conventional matrix, DHB, and successfully highlighted the visualization of sulfatides in mouse brain samples.

##### 4.4.1.7 Glycoside

Glycosides are secondary metabolites found in plants and are formed by the attachment of sugar molecules to nonsugar aglycones through glycosidic bonds.^1264^ Their amphipathic nature, with a hydrophilic glycosyl component and a hydrophobic aglycone component, enables them to interact with both water and lipids, playing essential roles in cellular signaling, transmembrane transport, and plant defense mechanisms. Glycosides can be classified into various types based on the structural characteristics and functions of their aglycones, such as cardiac glycosides, flavonoid glycosides, and saponins.^1265^ They play significant roles in plant metabolism and exhibit a wide range of biological activities, such as anti-inflammatory, antioxidant, and antibacterial effects. This chemical diversity makes glycosides valuable for drug development, the food industry, and agriculture. For instance, cardiac glycosides are used to treat heart diseases, steviol glycosides act as natural sweeteners, and saponins serve as surfactants in emulsifiers. In summary, glycosides are important not only for basic research but also for their extensive applications.

Flavonoids and flavonoid glycosides are the most researched glycosides in MALDI-MS. Wang et al. successfully identified flavonol glycosides in green tea using MALDI-TOF MS and THAP as the optimal matrix for ionization.^1099^ In positive ion mode, these glycosides exhibited multiple ionic forms, including [M+H]^+^, [M+Na]^+^, [M+K]^+^, and [M–H+Na+K]^+^, and showed fragmentation, providing detailed structural information, while the negative ion mode primarily produced [M–H]^−^ ions without fragmentation data. The findings indicated that flavonol glycosides generally had more consistent signal intensity in positive mode, whereas notable response variations were observed among different glycosides in negative mode, such as kaempferol glycosides, whose responses were significantly lower than those of quercetin glycosides. Frison-Norrie et al. employed THAP as the matrix for the qualitative and quantitative analysis of flavonol glycosides in almond seedcoats using MALDI-TOF MS.^1266^ The study successfully identified four flavonol glycosides: isorhamnetin rutinoside, isorhamnetin glucoside, kaempferol rutinoside, and kaempferol glucoside. Furthermore, the researchers utilized rutin (quercetin-3-rutinoside) as an internal standard and established an efficient MALDI-TOF MS method for the precise quantification of these four flavonol glycosides. Beck and Stengel employed MALDI-MSI technology using DHB as the matrix to facilitate the specific identification and localization of flavonoid glycosides and bioflavonoids, enabling the generation of mass spectrometric images of plant constituents from thin sections.^1267^ By utilizing a MALDI source with high lateral resolution (as low as 3 μm), they successfully revealed the detailed distribution patterns of flavonoid diglycosides and biflavonoids in *Ginkgo biloba* L. leaves.

Cardiac glycosides are one of the most extensively studied classes of glycosides and are widely used for the treatment of heart failure and atrial arrhythmias because of their significant positive inotropic effects. They enhance myocardial contractility by inhibiting the activity of the sodium–potassium pump (Na^+^/K^+^-ATPase), which increases intracellular calcium ion concentrations, making them important drugs for the treatment of cardiovascular diseases.^1268^ In molecular-level investigations of plant chemical defense, Dreisbach et al. employed atmospheric-pressure 3D-surface MALDI-MSI to explore the phytochemical defense mechanisms of *Asclepias curassavica*. Their study revealed that the DHB matrix yielded optimal analytical results for cardiac glycosides and other secondary plant metabolites.^1269^ Spatial imaging (Figure 103a) revealed the localized accumulation of cardenolides like calotoxin (*m*/*z* 587.2250, [M+K]^+^, red) exclusively in the injured areas, while dihydroxyflavone (*m*/*z* 253.2639, [M+H]^+^, green) were uniformly distributed on the entire leaf surface. These results demonstrated that upon the simulation of mechanical damage analogous to herbivore attacks, cardenolides and other defensive metabolites were exclusively detected within the damaged leaf tissue, with an increased flow rate of latex facilitating the accumulation of these compounds.

**Figure 103:**
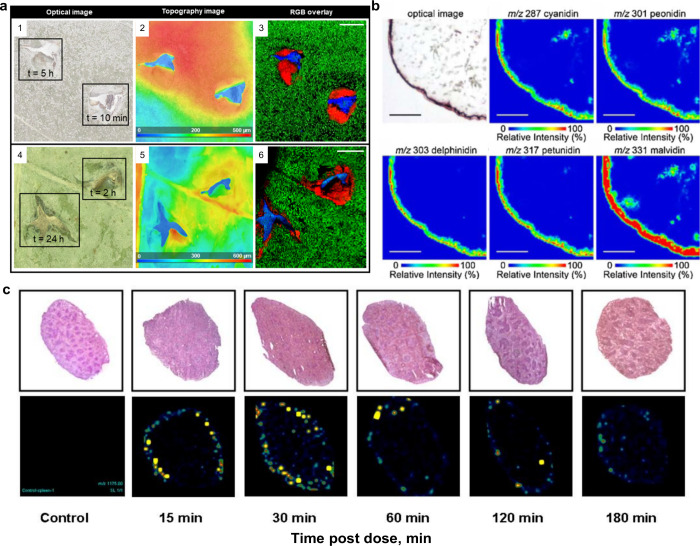
(a) 3D-surface MALDI-MS imaging of *A. curassavica* leaves. Both samples were injured twice at different time intervals before they were harvested. (1,4) Optical microscopy image of the leaf surface after measurement. The injured areas are marked and the associated time intervals are indicated. (2,5) Topographical image of the leaf surface showing height differences up to 500 μm and 600 μm, respectively. (3,6) RGB overlay of ion images showing the spatial distribution of calotoxin (*m*/*z* 587.2250, [M+K]^+^, red), dihydroxyflavone (*m*/*z* 255.0652, [M+H]^+^, green) and *m*/*z* 255.2110 (blue). MS images were generated with c 111 × 111 pixels, 45 μm pixel size, f 112 × 103 pixels; 35 μm pixel size; (c,f) *m*/*z* bin width: Δ­(*m*/*z*)/*m*/*z* = ± 5 ppm. Scale bars: 1 mm. Adapted and reproduced with permission from ref 1269. Copyright 2022 The Authors, under exclusive license to Springer Nature. (b) MALDI-IMS analysis of anthocyanidins, the aglycones of anthocyanin, in a rabbiteye blueberry section. Optical image of the rabbiteye blueberry cross-section used in the following MALDI-MSI analyses. MALDI-MSI images of the ions at *m*/*z* 287, 301, 303, 317, and 331 corresponding to cyanidin, peonidin, delphinidin, petunidin, and malvidin, respectively. Scale bar: 2.0 mm. Adapted and reproduced with permission from ref 1270. Copyright 2012 Springer-Verlag. (c) Cucumarioside A_2–2_ MALDI images of acutely dosed mouse spleen samples. The top-most row displays the H&E stained section for the representative spleens. The bottom row shows the spatial distribution of cucumarioside A_2–2_ for a representative spleen at each acute time point. Adapted and reproduced with permission from ref 1271. Copyright 2013 Elsevier BV.

Yoshimura et al. analyzed 10 anthocyanin molecular species in rabbiteye blueberry (*Vaccinium ashei*) using DHB as the matrix and MALDI-IMS-MS to study the effects of MSI on the identification and visualization of anthocyanins.^1270^ The results, depicted in Figure 103b, present an optical image of the cross-section of the rabbiteye blueberry and distinct distribution patterns of various anthocyanins within the exocarp and mesocarp of the blueberry slices. Specifically, cyanidin, onidin, and malvidin are distributed in both the exocarp and mesocarp, whereas other anthocyanins, such as delphinidin and petunidin, are primarily concentrated in the exocarp. Additionally, the distribution patterns were more closely associated with their respective aglycones than with their sugar moieties. The Pislyagin team employed radioisotopic tracing (using a ^3^H-labeled compound and liquid scintillation counting), MALDI-MS, and MALDI-MSI technologies (with CHCA as the matrix) to systematically analyze the stability and dynamic changes of the triterpene glycoside cucumarioside A_2_-2, the main compound of the medical lead compound Cumaside for use in immunodeficiency diseases, in the spleen tissue of mice.^1271^ As shown in Figure 103c, cucumarioside A_2_-2 remained stable for 24 hours post-injection, with no metabolic conversion, and reached its maximum concentration (*C*_max_) within 30 minutes. Additionally, MALDI-MSI analysis revealed that this glycoside primarily accumulated in the serosal region of the spleen, with a significant increase in drug concentration around the organ 15–30 minutes after administration, followed by a gradual decrease in surface concentration, while the internal redistribution changes were relatively limited^1271^. Furthermore, Petroselli et al. analyzed the saponin fingerprint profile of leaves and stems in commercial yerba mate (*Ilex paraguariensis*) using MALDI-MS with DHB as the matrix, and conducted an in-depth analysis of the typical saponin fingerprint profiles of *Ilex paraguariensis* and *Ilex dumosa*.^1272^

#### 4.4.2 Outlook

Carbohydrates are the most abundant biomolecules in nature, with various forms, including monosaccharides, oligosaccharides, polysaccharides, and glycoconjugates (such as glycoproteins and glycolipids). They play critical roles in numerous physiological functions. However, despite rapid advances in genomics and proteomics, progress in glycobiology has been hampered by the vast diversity of carbohydrate isomers and their low abundance, which present significant challenges for both qualitative and quantitative analysis. Furthermore, glycosylation is crucial in biological processes, with modification status closely linked to the regulation of protein function, cellular communication, and the pathogenesis of diseases. Nevertheless, due to the complexity of glycosylation modifications and their low abundance in samples, current detection and analytical techniques still face certain limitations. Given the difficulty of achieving complete derivatization and the potential for increased peak numbers to complicate resolution, developing effective matrices to prevent derivatization has become an alternative approach for carbohydrate detection. Therefore, the development of more efficient and sensitive matrices to meet the needs of carbohydrate research has become an important issue that the scientific community urgently needs to address. Future research should focus on optimizing the properties of existing matrices, such as enhancing ionization efficiency, reducing background noise and increasing the sensitivity of detecting low-abundance glycosylation modifications. Future research should focus on optimizing the properties of existing matrices, such as enhancing ionization efficiency, reducing background noise, and increasing the sensitivity of detecting low-abundance glycosylation modifications. Additionally, designing specialized matrices that can selectively enhance the ionization of glycoconjugates or effectively distinguish between carbohydrate isomers will help expand the application of MALDI-MS technology in carbohydrate analysis.

### 4.5 MALDI Matrices for Nucleic Acid Analysis

Nucleic acids are biomolecules composed of polymerized nucleotides. Each nucleotide consists of a basic group, a pentose sugar, and a phosphate group. Based on the type of pentose sugar in the nucleotide, either d-ribose or d-deoxyribose, nucleic acids are classified as DNA and RNA. Single-stranded DNA consists of nucleotides linked to form a chain, where each pentose sugar is attached to one of four bases, including cytosine (C), guanine (G), adenine (A), or thymine (T). Double-stranded DNA forms via specific base pairings between two antiparallel strands, C pairs with G and A with T. The primary function of DNA is the long-term storage of genetic information encoded by the sequence of its bases. RNA, while structured similarly to DNA, features uracil (U) instead of T. Some RNA molecules carry genetic information like DNA, whereas others exhibit enzymatic capabilities akin to those of proteins. Nucleotide sequence differentiation renders each DNA or RNA molecule unique and essential, carrying heritable information critical to all life forms. Genetic information, contained within specific segments of nucleic acid sequences called genes, encodes the vital instructions necessary for the synthesis and organization of all biomolecular components, thereby regulating cellular and organismal behaviors.

Here, some matrices used for nucleic acid analysis in MALDI-MS are introduced (Table S12). Compared with its use to analyze proteins and peptides, the application of MALDI-MS for nucleic acid analysis has lagged behind.^397^ As shown in Figure 104, the performance comparison of biochemical/MALDI-TOF MS/nucleic acid amplification testing (NAAT) demonstrates that MALDI-TOF MS has unique advantages in nucleic acid detection.^1273^ In addition, MALDI-TOF MS can be used for the identification of *Mycobacterium* tuberculosis and the analysis of drug resistance profiles.^1274^ This changed significantly in 1993 with the groundbreaking discovery by Becker’s group, which demonstrated that 3-HPA could serve as an effective matrix. One pivotal breakthrough was its application in single nucleotide polymorphism (SNP) detection. In 1997, Haff and Smirnov from Applied Biosystems innovated a spectral method combining single-base primer extension chemistry with MALDI-TOF for SNP identification.^1275^ A key advantage of this method is its ability to concurrently detect dozens of SNPs within a single sample reaction system. Compared with traditional nucleic acid analytical techniques, MALDI-TOF-MS not only allows the precise identification of oligonucleotide sequences but also enables rapid and accurate determination of the types and locations of their modifications. This capability is crucial for diagnosing genetic disorders, conducting screenings, and guiding therapeutic dosing decisions. Shuai et al. demonstrated the detection results of the MALDI-TOF MS technology for single-target viral standard plasmids, clearly presenting the specific peak positions of unextended probes and single-base extension products in each viral plasmid sample through mass spectra, verifying the accuracy of single-target detection. Additionally, they validated the specificity of MALDI-TOF MS by detecting target viruses and nontarget viruses (Figure 105).^1276^ The most common clinical SNP detection methods include Sanger sequencing, fluorescent quantitative polymerase chain reaction (PCR), low-density gene chips, and pyrosequencing; however, these methods cannot involve multigene or multisite analysis. Conversely, MALDI-TOF-MS allows the simultaneous detection of up to 52 SNP sites, significantly enhancing the efficiency of multigene and multisite detection.

**Figure 104:**
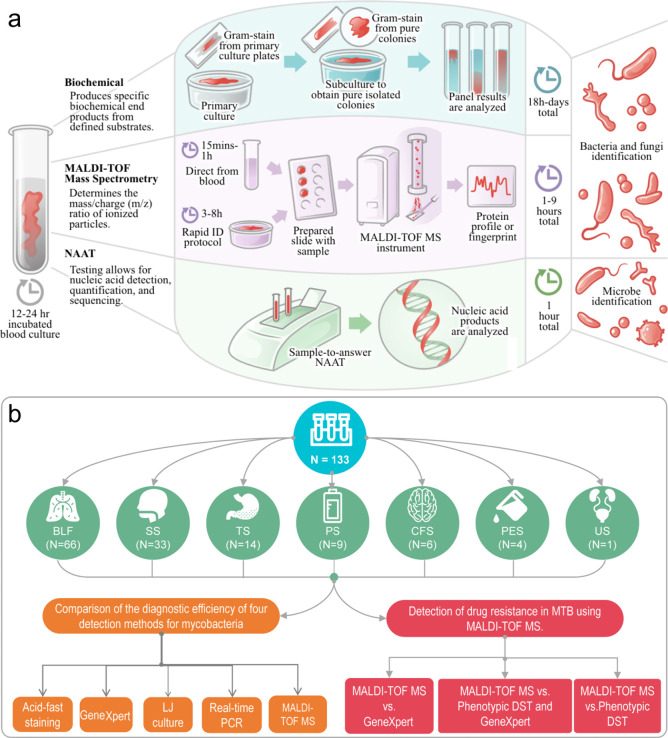
Biochemical/MALDI-TOF MS/NAAT performance metrics and mycobacterial identification with drug resistance profiling. (a) Biochemical assays, MALDI-TOF MS, and NAAT identification processes and performance turnaround times. NAAT, nucleic acid amplification test. Reproduced with permission from ref 1273. Copyright 2024 Oxford University Press. (b) Schematic overview of the study design for evaluating the diagnostic performance of nucleic acid MALDI-TOF MS in mycobacterial identification and drug resistance profiling. This schematic diagram illustrates the comprehensive study design employed to assess the diagnostic capabilities of nucleic acid MALDI-TOF MS in the identification of MTB and NTM, as well as in profiling their drug resistance. Reproduced with permission from ref 1274. Copyright 2025 ASM Journals.

**Figure 105:**
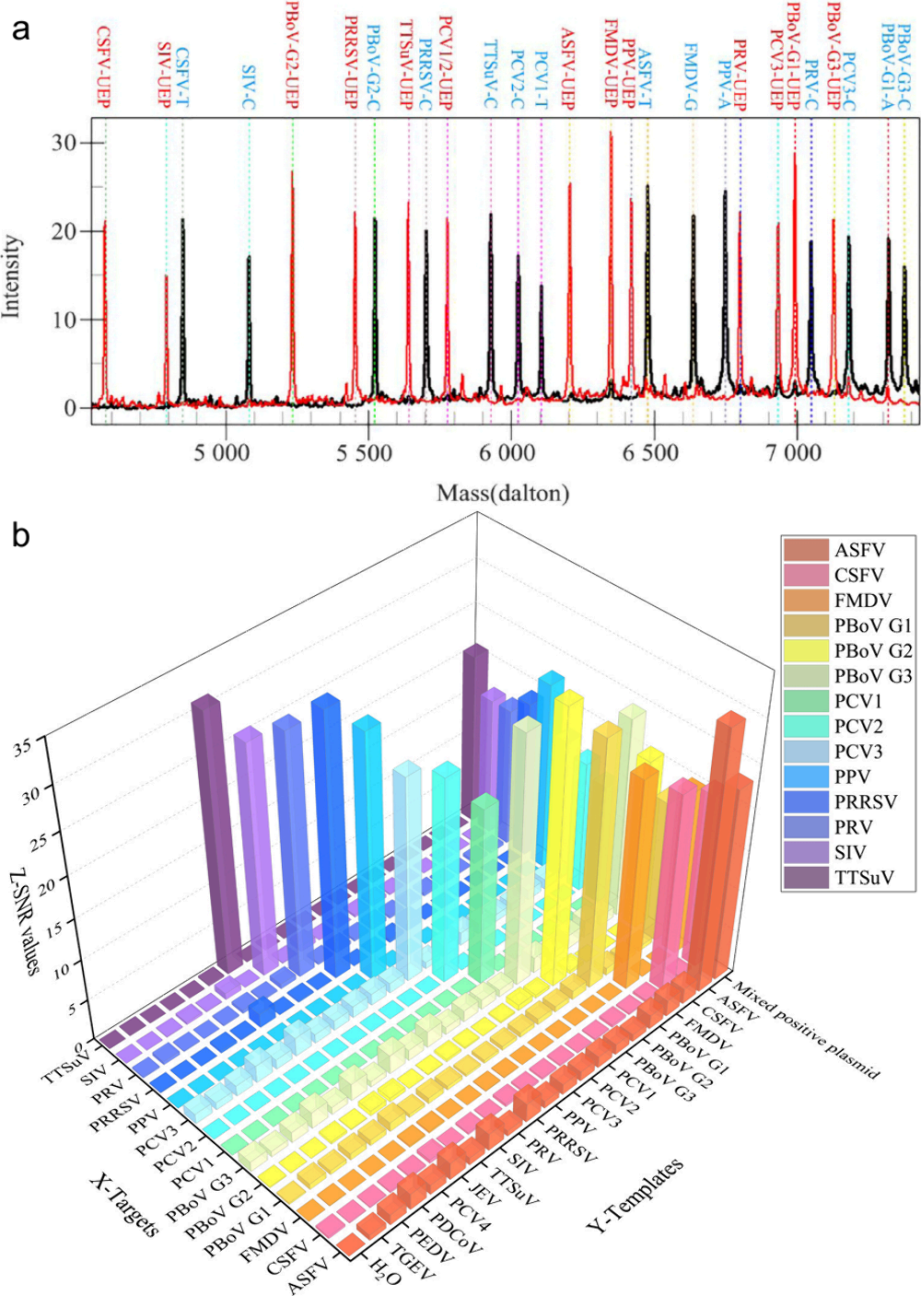
Specific detection of MALDI-TOF MS for nucleic acid. (a) The MALDI-TOF MS assay of multiple viral standard plasmids. In the mass spectrograms, the black peak represents the mass spectrometry result of mixed plasmids, and the red peak represents the mass spectrometry result of the blank control. (b) Validation of the specificity of MALDI-TOF MS by detection of target and non-target viruses. *X*-axis: Specific target of the primers and probes used in this assay. *Y*-Axis: The mixed standard plasmids, 14 target viruses, and four non-target viruses. *Z*-Axis: Signal-to-noise ratio (SNR) value. SNR ≥ 6 is considered positive. Reproduced with permission from ref 1276. Copyright 2024 Elsevier BV.

The analysis of oligonucleotides using MALDI-TOF-MS was first reported in 1990.^1277–1279^ Initially, researchers primarily focused on adapting matrices and laser wavelengths traditionally used for protein and peptide analysis. However, they soon encountered unique challenges with nucleic acids, such as significant ion fragmentation and the formation of metal cation adducts on analytes, which resulted in lower mass spectral quality than that obtained for proteins and peptides. These challenges necessitated the development of novel MALDI conditions, particularly the use of new matrices, to achieve better ionization efficiency. In 1992, Tang et al. reported the use of a mixture of 3-methylsalicylic acid and 3-hydroxy-4-methoxybenzaldehyde as matrices, enabling the detection of oligonucleotides up to 34-mers in length.^398^ Nordhoff et al. reported the detection of tRNAs and 5S rRNA in *Escherichia coli* using succinic acid, urea, and NA as matrices, combined with an IR laser at a wavelength of 2.94 μm.^1280^ In 1994, Wu et al. analyzed single-stranded DNA oligomers up to 67 nucleotides in length using 3-HPA as the matrix.^397^ Although other compounds such as 2,4,6-THAP,^308^ 2,3,4-THAP,^306^ picolinic acid,^399,1281^ 3-aminopicolinic acid (3-APA),^1282^ ATT,^476^ MSA,^353^ QA,^478^ PCA,^640^ 3-HC,^382^ and DABP,^410^ were found to be effective matrices, 3-HPA remains the most commonly used matrix for nucleic acid analysis. In addition, glycerol serves as an ideal matrix in IR-MALDI, generating molecular ions of RNA transcripts up to 2180 nucleotides long.^1283^

As the most commonly used matrix for nucleic acid analysis, 3-HPA is suitable for detecting single-stranded DNA oligomers in both positive and negative ion modes.^60^ Studies have demonstrated that oligonucleotides larger than 25-mers achieve stronger signal intensities and higher mass resolution in linear TOF systems than in reflection TOF systems. Although 3-HPA has been proven to be a very effective MALDI matrix for the mass spectrometric analysis of ssDNA, problems still persist. To improve the performance of 3-HPA, many scientists have begun to explore various approaches. For example, Carda-Broch et al. reported the practical use of ionic matrices for DNA oligomer detection with MALDI-TOF-MS.^620^ Their studies tested oligonucleotides using several ionic matrix compounds synthesized with two acids (3-HPA and DHB). The results showed that compared with traditional organic matrices, ionic matrices can increase the ion peak intensity of oligonucleotides. Additionally, Zhou et al. discovered a mixed matrix system of 3-HPA and PCA for analyzing DNA segments, showing improvements in reproducibility, resolution, the S/N ratio, and tolerance to metal salts compared to using 3-HPA alone.^640^

Furthermore, interactions between nucleic acids and proteins play a vital role in many biochemical processes, including DNA replication, recombination, and repair. Therefore, analyzing these interactions is crucial. Thiede et al. demonstrated that using 2,4,6-THAP in negative ion mode effectively permits MALDI-MS to analyze noncovalent RNA–peptide complexes, enabling the detection of peptides, RNA, and RNA-peptide complexes simultaneously.^307^ Additionally, Zhu et al. found that a mixed matrix of 2,3,4-THAP and 2,4,6-THAP enhances the performance of MALDI-TOF-MS in detecting DNA, which is especially suitable for samples containing low DNA concentrations like polymerase chain reaction products. This mixed matrix had better resolution, reproducibility, and sensitivity than the 3-HPA and picolinic acid matrices.^306^ In another study, Keiichiro et al. utilized MALDI to characterize the specific noncovalent complexes between guanidine derivatives and single-stranded DNA, using ATT as a matrix in the presence of DHC in positive ion mode and verifying the ability of ATT to characterize the noncovalent interactions between guanidine probes and ssDNA.^477^

In addition, Tang et al. discovered that pyridine acid is an ideal matrix for the MALDI-MS analysis of nucleic acids, successfully detecting single-stranded DNA with 190 nucleotides and double-stranded DNA with 190 base pairs.^399^ However, using pyridine acid as a matrix may result in more dimer ions, making it challenging to distinguish between dimers and doubly-charged parent ions when analyzing mixtures of DNA fragments. Nevertheless, pyridine acid overall remains an excellent matrix for obtaining mass spectra of larger DNA fragments. Concurrently, Viladkar et al. found that using FA as a matrix for MALDI-MS analysis of oligonucleotide–fluorophore conjugates significantly increased signal intensity, mass accuracy, and S/N ratio compared to the 3-HPA matrix.^271^ The results indicated that FA is the preferred matrix for analyzing oligonucleotide–fluorophore conjugates and can be effectively used for rapid screening before further applications. Song et al. conducted research demonstrating that QA serves as a novel matrix for MALDI-MS nucleic acid analysis under specific conditions. This matrix provided uniform crystals and reproducible signals, thus optimizing the spectral resolution of DNA^478^. In addition, Zhang et al. identified 3-HC as a new matrix for MALDI-MS analysis of DNA, especially effective for oligonucleotides from 3 to 70 bases in length.^382^ Compared to traditional matrices, using 3-HC significantly enhances the resolution, S/N ratio, dot pair distinction, and reproducibility between samples of DNA fragments. Fu et al. demonstrated that using DABP as a MALDI-MS matrix for oligonucleotides enables the production of intact ions at lower laser power and improves detection limits. Compared with traditional matrices, DABP reduces fragmentation and alkali metal ion adducts, and provides excellent reproducibility, resolution, and S/N ratio.^409,410^

In recent years, new matrices for nucleic acid MALDI-MS analysis, such as anthranilic acid, DAN, 6-TG, and ANP, have continually emerged (Figure 106). Zhang et al. first utilized AA as a matrix by forming Schiff bases from a mixture of AA, niacin, and ammonium citrate, which then react with the basic sites of modified oligodeoxynucleotides for analysis.^499^ Although MALDI-TOF-MS methods are applied to short oligonucleotide sequencing, the low fragmentation efficiency during ISD or PSD requires the acquisition of numerous spectra, thereby limiting throughput. To overcome the throughput limitations of prior short oligonucleotide sequencing methods, Hagan et al. utilized DAN to enhance in-source fragmentation analysis of oligonucleotides by inducing ISD of DNA and RNA molecular anions through DAN for rapid sequence confirmation, especially during the D/I process in negative ion mode.^1284^ Satoshi et al. discovered that nucleobase derivatives can serve as new MALDI matrices for the ISD of oligonucleotides. Compared with existing oligonucleotide matrices, nucleobase derivatives exhibit enhanced interactions with analytes because of their structural similarities, with 6-TG particularly demonstrating a marked effect in inducing ISD fragments.^473^ They successfully used 6-TG as a MALDI matrix to characterize 10 single-stranded RNA and DNA sequences, showcasing the potential of nucleobase derivatives as novel matrices in oligonucleotide sequencing. To examine the importance of pH in the MALDI analysis of oligonucleotides, Michael et al. screened 37 highly substituted pyrimidine, pyridine, and benzene derivatives containing basic amino groups as potential matrices.^406^ They found that ANP can effectively analyze mixed-base, single-stranded oligonucleotides (<20 basic groups) and thymine homopolymers. This development significantly extends the application range of MALDI for acid-sensitive species, although limitations remain in oligonucleotide sample analysis. Additionally, Sau-wan et al. employed ANP with ammonium halides as co-matrices for the MALDI analysis of oligonucleotides, determining that all ammonium halides greatly enhance the signal intensity of intact molecular ions for DNA homopolymers.^407^ The results indicated that among the halides employed, NH4F provided the most effective enhancement. Furthermore, the ANP matrix exhibited strong inhibition of alkali metal adduct formation during oligonucleotide analysis.

**Figure 106:**
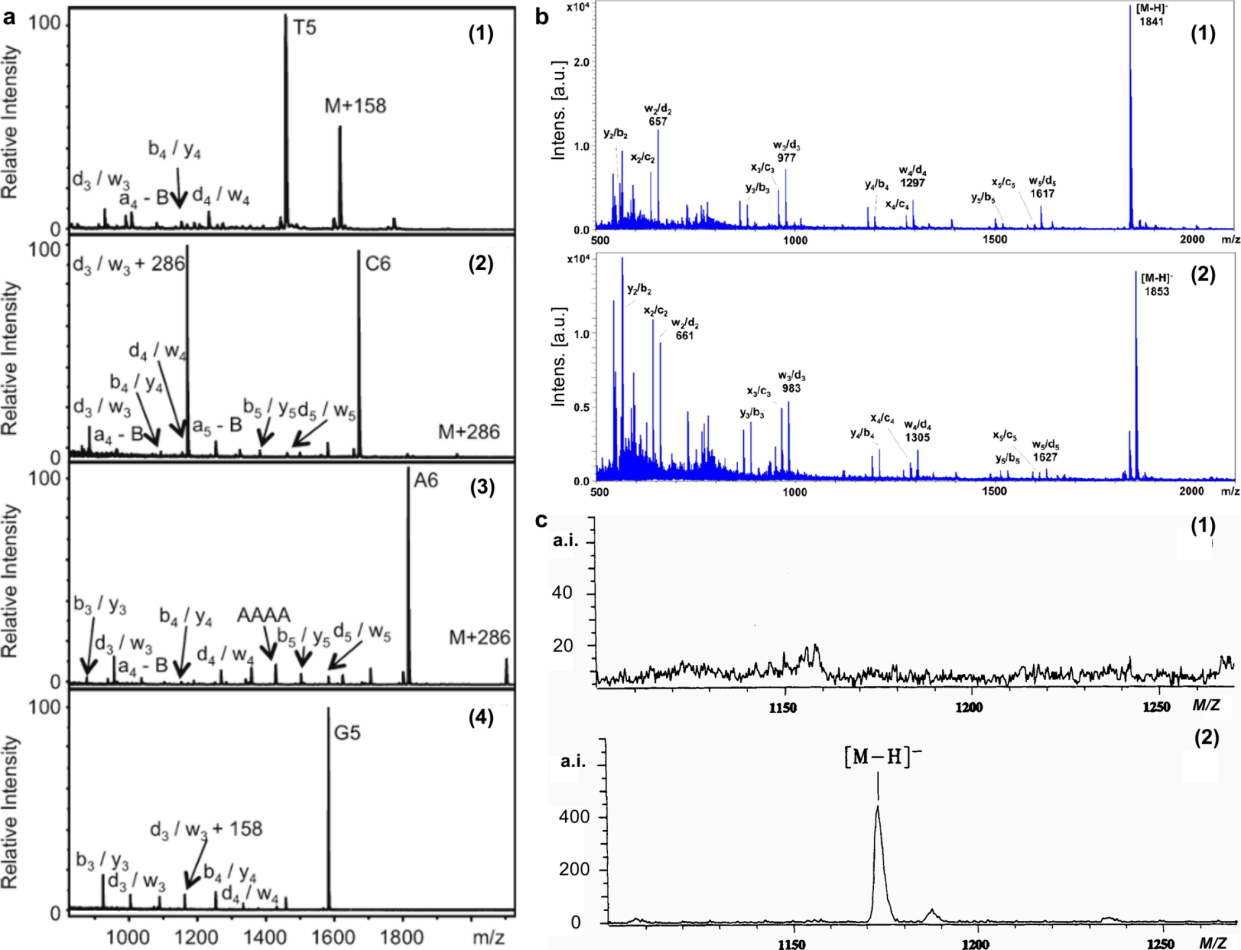
Comparative MALDI-TOF MS analysis of nucleic acid using DAN, 6-TG, and ANP matrices. (a) Negative ion MALDI TOF ISD spectra using DAN matrix: (1) dT_5_, (2) dC_6_, (3) dA_6_, and (4) dG_5_ homopolymeric oligonucleotides. Reproduced with permission from ref 1284. Copyright 2012 American Society for Mass Spectrometry. (b) Mass spectra of 6-mer phosphorothioate DNA or RNA using 6-TG as the matrix: (1) dT_6_-PS and (2) rU_6_-PS. Reproduced with permission from ref 473. Copyright 2019 John Wiley & Sons. (c) MALDI mass spectra of pd­(C)_4_ in (1) ANP matrix and (2) ANP/NH_4_F matrix. Reproduced with permission from ref 407. Copyright 1996 John Wiley & Sons.

Beyond the use of organic matrices, numerous studies have explored inorganic matrices because of their effectiveness in providing a clean background. For instance, Kong et al. discovered that polylysine-coated diamond nanocrystals can serve as solid-phase extraction supports to concentrate diluted oligonucleotide solutions, facilitating the separation of oligonucleotides from proteins in highly contaminated solutions.^1285^ This method results in the formation of stable complexes between amine-functionalized diamond nanocrystals and DNA, enabling the large-scale analysis of DNA in more complex protein solutions and even cell lysates. Furthermore, Hong et al. designed and fabricated various nanoscale structures for surface-localized DNA, consisting of a layer of negatively charged silica NPs and a layer of positively charged AuNPs, to allow the covalent binding (Au-S bond) and hybridization of oligonucleotides, thereby achieving unprecedented detection capabilities.^1286^

Due to the highly negative charge of the phosphodiester backbone of oligonucleotides, issues such as cation adsorption and high detection limits arise, which limit the widespread application of MALDI-MS for oligonucleotide analysis. Dopants have proven to be crucial in the MALDI analysis of nucleic acids. Ammonium salts such as ammonium acetate,^1287^ ammonium citrate, ammonium tartrate,^308^ and ammonium fluoride^1288^ have been identified as useful dopants that significantly suppress the adsorption of metal cations. Spermine, which acts as a cation-exchange agent, has been utilized as a dopant in various matrices,^353,648^ albeit not 3-HPA.^648^ Other compounds, such as 2-PA,^1289^ various sugars, and PCA,^640^ have been added to 3-HPA to enhance its performance. Specifically, TETA-SPM as a dopant can enhance UV-MALDI-MS detection capabilities for oligonucleotides by eliminating cation adsorption and reducing the detection limit of DNA without requiring desalting steps.^648^ Moreover, Vandell et al. found that polyamine co-matrices demonstrated successful performance in MALDI-MS analysis of oligonucleotides because of their amino functional sites.^652^ They evaluated the efficacy of tetrahydrochloride spermine, spermine, and trihydrochloride spermidine in enhancing mass spectral quality in MALDI-MS analysis of oligonucleotides. The spectrometric data indicated that spermine and spermidine were highly effective co-matrices, increasing both the resolution of molecular ions and their abundance. To improve DNA detection performance, Shahgholi et al. demonstrated that sugar-based dopants can enhance DNA signal intensity and quality resolution.^1289^ The addition of fructose or fucose to the 3-HPA matrix improved DNA mass spectral resolution and reduced the degree of metastable decay caused by extraneous energy from laser irradiation. Distler et al. found that using fucose as a dopant could improve the MALDI-MS analysis of oligonucleotides by increasing the uniformity, signal intensity, and duration of experiments, thereby enhancing the detection capability of complex oligonucleotide mixtures and reducing the degree of oligonucleotide fragmentation.^654^ Furthermore, combining SPM with MSA as a matrix for detecting nucleotides in the 12–20 base pair range yielded better resolution, lower fragmentation levels, and reduced alkali ion adduct peak intensity in MALDI-MS analysis than in matrices such as 3-HPA and ATT with added SPM or DHC.^353^

As several matrices capable of ionizing DNA and RNA have been discovered, MALDI-MS is becoming a platform for studying nucleic acid sequence variations (such as mutations, SNPs, insertions/deletions, alternative splicing), quantitative changes (such as copy number variations, gene expression, and allele expression), and modifications (such as genomic DNA methylation, post-transcriptional modifications of tRNA and rRNA). Although some MALDI-MS technologies for nucleic acid analysis have been reported, their application in real samples lags behind other nucleic acid analysis techniques (Sanger sequencing, quantitative PCR), and they have not yet been widely adopted. Therefore, most MALDI matrices for nucleic acid detection were developed before 2000. Nonetheless, the high throughput and high resolution characteristics of MALDI-MS make it a valuable complementary analytical technique for modern nucleic acid analysis, offering significant applications in revealing nucleic acid variations and functions within complex biological systems.

## 5 MALDI Matrices for Quantitative Analysis

MALDI-MS has emerged as a pivotal analytical tool in quantitative studies across diverse fields including proteomics, metabolomics, lipidomics, glycomics, and drug discovery, owing to its high throughput, exceptional sensitivity, and capability for spatial imaging.^1048,1290,1291^ However, the quantitative performance of MALDI-MS is inherently constrained by matrix-related factors, such as matrix–analyte co-crystallization homogeneity, ion suppression effects, background interference in the LMW mass region, and inconsistent D/I efficiency.^1048,1292,1293^ Matrix selection, therefore, stands as a fundamental determinant of the accuracy, precision, and robustness of MALDI-MS quantitative analysis.^1291^ In this section, we provide an overview of the necessity and current status of MALDI-MS quantitative analysis, emphasizing the advance in MALDI matrix development.

### 5.1 Necessity of MALDI-MS Quantitative Analysis

MALDI-MS has become a powerful analytical technique since its inception, enabling the rapid analysis of a wide spectrum of analytes ranging from small molecules (*e.g.*, metabolites, lipids, and drugs) to large biomacromolecules (*e.g.*, proteins and polymers).^1048,1290,1294,1295^ While early applications predominantly focused on qualitative identification, the demand for quantitative MALDI-MS has surged in recent years, driven by its potential in clinical diagnosis, drug development, environmental monitoring, and systems biology.^32,1048,1290,1296^ In clinical settings, for instance, accurate quantification of serum free FAs via paper-array plate based MALDI-MS can serve as a sensitive and potentially specific marker for gout, thereby facilitating the precise diagnosis of gout.^1297^ In spatial metabolomics, quantitative MALDI-MSI can visually reveal the microscale metabolic gradient changes in tissues, revealing region-specific metabolic activities that underpin disease pathogenesis.^32,1293^ In environmental monitoring, by harnessing the power of MALDI-MS quantification and implementing standardized practices, researchers can effectively monitor environmental pollution, identify and quantify emerging pollutants, and contribute to the preservation of our environment.^1296^ Similarly, in polymer science, quantitative MALDI-MS enables precise determination of MW distributions and copolymer compositions, critical for material performance optimization.^1298,1299^

The necessity of MALDI-MS quantitative analysis stems from its unique advantages over conventional quantitative techniques. Compared to LC-MS, MALDI-MS eliminates the need for tedious chromatographic separation, reducing analysis time from several tens of minutes to just a few minutes or even seconds and enabling high-throughput screening of hundreds to thousands of samples.^1048,1294^ For spatial quantitative analysis, MALDI-MSI provides pixel-level quantification of various analytes in intact tissues or cells, offering spatial information that is unattainable with traditional analysis methods.^1291^ Moreover, MALDI-MS exhibits exceptional sensitivity, capable of detecting analytes at femtomolar to attomolar concentrations, making it ideal for trace analysis of biomarkers in complex biological matrices.^1048,1290,1291,1293^ However, the translation of MALDI-MS from qualitative to quantitative analysis is hindered by inherent technical challenges. Unlike ESI, MALDI relies on a matrix to absorb laser energy, facilitate desorption, and promote ion formation. The matrix–analyte interaction is highly complex, and subtle variations in matrix properties can lead to significant deviations in quantitation.^8^. For example, the use of different matrices (norharman vs DHB) can result in drastically different peak intensity distributions of lipids in MALDI-MSI images, even when the same lipid internal standard (IS, PE15:0/18:1 (d7)) is applied.^1300,1301^ In small molecule analysis, traditional organic matrices produce intense background peaks in the LMW region (typically, *m*/*z* < 500), masking analyte signals and impairing quantification accuracy.^1302^ These challenges underscore the critical need for rigorous matrix selection and optimization to unlock the full potential of MALDI-MS quantitative analysis.

### 5.2 Current Status of MALDI-MS Quantitative Analysis

Over the past decade, significant advancements have been made in MALDI-MS quantitative methodologies, including the development of internal standard strategies, matrix modification techniques, and data normalization algorithms.^32,602,1291,1300,1303^ Isotope-labeled ISs are the most widely used approach to correct for matrix effects and ionization variability.^1300,1301^ For example, Wang et al. developed a spatial quantitative metabolomics method using ^13^C-labeled yeast extract as a universal IS, enabling pixel-level normalization and quantification of over 200 metabolic features in mouse brain and kidney tissues, with some achieving absolute quantification.^1300^ In lipid analysis, isotope-labeled lipid ISs have been successfully applied to MALDI-MS quantitative analysis.^1301^ In spatial quantitative analysis, MALDI-MSI has achieved remarkable progress in resolving tissue metabolic heterogeneity. A recent study reported an integrated experimental–computational workflow using high-resolution MALDI-MSI, isotope tracing, and deep-learning artificial intelligence.^1304^ Most measured metabolites (>90%) showed significant spatial concentration gradients in the liver lobules and intestinal villi. Despite the enormous progress that has been made in MALDI-MS quantitative analysis, several limitations still persist. For water-soluble metabolites with diverse physicochemical properties, the lack of universal ISs remains a major bottleneck, as individual ISs are often required for each metabolite class, leading to high costs and limited feasibility. In small molecule analysis, traditional organic matrices continue to pose challenges due to background interference^1305^ and poor co-crystallization with analytes.^282,518^ Therefore, the development of new matrices, standardized matrix application protocols, and new calibration strategies, are therefore essential for the widespread adoption of MALDI-MS in quantitative study.

### 5.3 Matrix Development for MALDI-MS Quantitative Analysis

Matrix selection is a pivotal step in MALDI-MS quantitative analysis, as the matrix directly influences every stage of the analytical process, from sample preparation to ion detection.^1291^ An ideal matrix should possess several key properties: strong absorption at the laser wavelength, high ionization efficiency, good miscibility with analytes, homogeneous co-crystallization, minimal background interference, and the ability to mitigate ion suppression. Deviations from these properties can lead to reduced sensitivity, poor reproducibility, and inaccurate quantification.

One of the primary challenges associated with matrix selection is matrix-induced ion suppression. The matrix competes with analytes for protonation/deprotonation, and high matrix concentrations can suppress analyte ionization, particularly in complex biological samples.^657^ For metabolite analysis, excess traditional SOM matrix can produce intense ion adduct peaks in the low MW region, masking metabolite ion signals and limiting quantification sensitivity. The choice of matrix can also affect co-crystallization homogeneity; uneven crystal formation leads to inconsistent laser energy absorption and ion generation, resulting in high signal variability. Traditional matrices, *e.g.*, DHB, often form large needlelike crystals with analytes, resulting in inhomogeneous co-crystallization and poor ionization efficiency stability.^555^ Background interference is another critical issue, especially for LMW molecule quantitative analysis (ref 1306). Conventional organic matrices (*e.g.*, 2-MBT, DHB, CHCA) ionize readily, producing intense background peaks in the *m*/*z* < 500 region, which overlap with small molecule analytes such as metabolites, vitamins, and drugs (refs 459, 1307 ). This interference significantly impairs the detection and quantification of LMW analytes. To address the issues mentioned above, many novel matrix systems have been developed, including nanomaterial-based matrices (*e.g.*, MOFs (ref 906) and COFs (ref 1308)), carbon-based nanomaterial matrices (refs 68, 825 ), metallic nanostructure matrices (refs 1309–1311 ), QD matrices (ref 955), reactive matrices (refs 529, 1291 ), ionic liquid matrices (refs 18, 588, 1312 ), matrix with dopants (ref 328, 1313, 1314 ), and binary-/hybrid-based matrices (Table S13) (ref 627, 1195 ). For instance, Ti-based MOF nanosheets have demonstrated low background interference, high signal stability, and superior ionization efficiency for small molecule MALDI-MS quantitative analysis (ref 1315). Two MOF matrices, *i.e.*, UiO-66-(SH)_2_@PdNP and CuO/Cu_2_O@ZnO-CN, show excellent sensitivity, ionization efficiency and specificity for carbohydrate quantitative analysis by MALDI-MS, along with low background interference, high salt and protein tolerance, good signal stability, and good reproducibility (ref 913, 1316 ). The latter also demonstrated excellent discrimination performance for oligosaccharide isomers (ref 1316). Fluoro-functionalized ionic COF (F-iCOF) has been used as a new matrix for highly selective enrichment and sensitive quantitative analysis of potassium perfluorinated sulfonate (PFS) by MALDI-MS (ref 1317). TAPB-DMTP-COF (refs 931, 1318 ) and COF_HD_ (ref 1319) have been successfully reported as new COF matrices for small molecules in MALDI-MS quantitative analysis. Similarly, carbon-based nanomaterial matrices (*e.g.*, G (graphene) (ref 68) and Bi_2_O_3_@GO (ref 825)) and metallic nanostructure matrices (*e.g.*, Fe_3_O_4_NPs (refs 1305, 1320 ), AgNPs (ref 1309), AuNPs (refs 1310, 1311 ), Au@TiO_2_NSs (ref 1321), AuNPs-FPTDF (ref 1322), and so on) have also been developed and used for small molecule MALDI-MS quantitative analysis. Obviously, compared with commonly-used SOM matrices (*e.g.*, DHB, CHCA, etc.), these highly salt-tolerant matrices also demonstrate significant advantages in terms of high detection sensitivity, high signal stability, high repeatability, and low background signal interference for analyte MALDI-MS detection. Taking Bi_2_O_3_@GO matrix for an example, Bi2O_3_@GO exhibits improved S/N response and eliminated matrix-related interferences compared to traditional SOM matrices and it was successfully applied for the quantitative analysis of glucose in human serum and soft drinks (ref 825). AuNPs-FPTDF was also reported as a metallic nanostructure matrix for sensitive and quantitative small-molecule analysis via MALDI-MS and MALDI-MSI (ref 1322). The use of AuNPs-FPTDF enables dual-polarity detection with ultrahigh sensitivity: LOD reaches 10 amol for histidine, 0.8 amol for glucose, 0.2 amol for oxymatrine, 4.0 pmol for dopamine, and 2 pmol for stearic acid, alongside excellent repeatability (coefficient of variation <2%) and salt tolerance (stable signals up to 5 mM NaCl). For clinical applications, it quantifies rat blood glucose across 0.5-9 mM (*R* ^2^ = 0.9999) with results matching commercial kits. Moreover, it enables imprint MALDI-MSI of *Catharanthus roseus* flowers, visualizing tissue-specific distributions of alkaloids and flavonoids, highlighting its versatility for both quantitative bioanalysis and spatial metabolomics. Furthermore, the development of DHB@MNP (organic–inorganic hybrid matrix, ref 984) and QDs-DDTC (quantum dot matrix, ref 955) completely eliminates the interference of background signals on MALDI-MS quantitative analysis, thereby enhancing the sensitivity, stability, repeatability, and reliability of quantitative detection of analytes. In addition, reactive matrices (*e.g.*, 2-HQ (ref 552), 4-HQ (refs 445, 1323 ), 3-AQ (ref 501), AP (ref 500), PAPAN (ref 502), 2-HTA (ref 518), 3-CACA (ref 282), TMNTA (ref 528), DAN (refs 1324–1327 ), and so on), ionic liquid matrices (*e.g.*, 3-AQ/CHCA (ref 1312), 3-AQ/THAP (ref 1328), 3-QA/HCOONH_4_ (ref 1329), DHB/*N*-MA (refs 588, 1305 ), DHB/*N*-EA (ref 588), DHB/DMA (ref 1330, DHB/Pyr (ref 583), CHCA/AP (ref 1331). CHCA/ANI (ref 1332), 1,5-DAN/NH_4_F (ref 1333), and so on), matrix with dopants (*e.g.*, THAPNaSm (ref 1334), NaDHB (ref 328), DHB/MSA/fucose (ref 1313), FA/fucose (ref 1313), DHB/AS (ref 1314), and so on), and binary-/hybrid-based matrices (*e.g.*, DHB/DHBH (ref 627), (DHB/3HBA)/(DHB/Q3CH) (ref 1195), and so on) have also been developed to overcome the aforementioned shortcomings of traditional SOM matrices and to be used for quantitative analysis of various analytes (such as small molecules, metabolites, carbohydrates, lipids, amino acids, proteins, drugs, etc.) by MALDI-MS (Table S13). For example, 2-HQ was successfully reported as a novel reactive matrix for sensitive and quantitative carbohydrate analysis via MALDI-MS (ref 552). It efficiently reacts with carbohydrates’ reducing ends to form stable hydrazones, boosting ionization efficiency 10-fold vs 3-AQ and 100-fold vs. DHB. With excellent reproducibility, 2-HQ enables quantitative analysis of neutral, acidic, and LMW carbohydrates over wide linear ranges, highlighting its potential for high-throughput glycomics quantification in complex samples. A boronic acid-modified reactive matrix, *i.e.*, TMNTA has been also successfully developed, enabling high-throughput absolute quantification of monosaccharide isomers (*i.e.*, glucose and fructose) in honey.^528^ Recently, 3-AQ/THAP was successfully developed as a novel ionic liquid matrix for sensitive and accurate quantitative detection of miRNA via MALDI-MS, outperforming conventional matrices like DHB and 3-HPA (ref 1328). The use of 3-AQ/THAP minimizes alkali metal adduct peaks, reduces the “sweet spot” effect (RSD <7% across 36 single-spot analyses), and achieves broad mass coverage (3-mer to 50-mer oligonucleotides) with single-base resolution. For quantitation, it delivers LOD of 2.5-50 fmol for 6-mer to 31-mer oligonucleotides, a linear range of 0.4-40 μM (*R* ^2^ > 0.988) for 11, 16, 21, and 26-mer oligonucleotides, and high consistency with UHPLC-UV (*R* ^2^ = 0.969) at 1/7th the analysis time. Applied to complex samples, 3-AQ/THAP quantifies has-miR-575, has-miR-656, and has-miR-7-5p in human plasma, fetal bovine serum, and fetal equine serum with recoveries of 89.1–104.9%, demonstrating robust utility of 3-AQ/THAP for miRNA biomarker screening and oligonucleotide biopharmaceutical quality control. In matrix with dopants, AS (ammonium sulfate) is a unique matrix dopant that improves the signal of various types of biomolecules including oligonucleotides (ref 308), proteins (ref 1335). and glycolipids (ref 1336) in MALDI-MS. In addition, a study has shown that AS, as an dopant to DHB matrix, drastically enhances the quantitative detection of hydrophilic quaternary ammonium compounds (QACs) by MALDI-MS and MALDI-IMS, overcoming inherent ion suppression for these bioactive metabolites (ref 1314). In mouse brain homogenates, AS boosts signal intensities of carnitine (Car), acetylcarnitine (AcCar), and glycerophosphocholine (GPC) by *ca*. 300-, 700-, and 2500-fold, respectively, with a higher AS concentration (≥63 mM) required for this improvement than that (≤31 mM) for suppressing potassium adduction on phosphatidylcholine and DHB matrix clusters. For MALDI-MSI, AS elevates these QAC signals by 10–40-fold, enabling simultaneous visualization of five QACs (Car, AcCar, GPC, choline, and phosphocholine) while preserving their native tissue distributions. The salting-out effect of AS reduces ion suppression from biological matrices, establishing DHB/AS as a robust platform for sensitive, accurate QAC quantification and spatial mapping in complex biosamples.

It is worth noting that the development of mentioned above new matrix systems has significantly advanced the progress of MALDI-MS quantitative analysis, but it is undeniable that up to now, most studies still choose SOM matrices for quantitative analysis. Therefore, to enhance the reliability and accuracy of MALDI-MS quantitative analysis studies, two main effort aspects are involved, (i) Strict quality control of experimental conditions (*e.g.*, matrix solvent optimization to form homogeneous analyte–matrix co-crystallization, matrix spotting/coating optimization to improve the matrix crystallization homogeneity (refs 1337–1339 ), combined with the use of isotope labeling technology (refs 1340–1344 ), immuno-MALDI technology (ref 1345–1347 ), internal standard control (refs 1301, 1348–1352 ), automatic MS acquisition (ref 1353, 1354 , etc.) based on the use of traditional SOM matrices (*e.g.*, DHB (refs 516, 1348–1350, 1353–1357 ), CHCA (refs 1343–1347, 1351, 1352, 1358–1372 ), SA (refs 1337, 1373–1375 ), 9-AA (refs 1297, 1339, 1376, 1377 , and so on); (ii) novel SOM matrix development (*e.g.*, DHT (ref 359), HFMC (ref 1378), HYNIC (ref 403), NEDC (refs 435, 1379, 1380 ), ATNEDC (ref 1131), NHHC (ref 438), 2-NPG (ref 421), NRM (ref 1301), 3-HPA (ref 1332, 1381 ), and so on) (Table S13). These advanced matrices exhibit superior properties, including low background interference, high signal stability, enhanced salt tolerance, improved ionization efficiency, and good reproducibility, particularly for various analytes from complex biological samples. Taking four new SOM matrices, *i.e.*, DHT, NEDC, ATNEDC, and 2-NPG, for example, these matrices have been successfully developed to address distinct analytical challenges for AAs, carbohydrates, FFAs, and the fungicide pyrimethanil (PYM), respectively, forming a tiered technology pipeline from endogenous biomolecule profiling to exogenous pesticide spatial quantitation. For AAs, DHT was engineered as the first AA-specific MALDI matrix, overcoming the limitations of conventional matrices (DHB and CHCA) that produce high background noise in the LMW range and low AA detection coverage. DHT exhibits strong UV absorption at 355 nm (molar extinction coefficient 13,800 L·mol^–1^·cm^–1^, 1.8–9.2× higher than CHCA/DHB), uniform crystallization, and high vacuum stability, with matrix-related ion signals reduced by 50% and 71.8% relative to DHB and CHCA at *m*/*z* < 500. Quantitatively, DHT achieves LOD of 4–6 ng/mL for arginine, glutamic acid, glutamine, and proline, and detects all 20 proteinogenic AAs in human serum (vs 7 and 10 AAs for DHB and CHCA). For MALDI-MSI, DHT enabled the first *in situ* imaging of 20 protein AAs and taurine in edible oyster tissues, resolving heterogeneous spatial distributions (*e.g.*, alanine enriched in gills, glycine concentrated in connective tissue) that were undetectable with conventional matrices. NEDC addresses the longstanding challenge of carbohydrate isomer differentiation and relative quantitation by forming stable chloride-adducted ions ([M+Cl]^−^) of underivatized disaccharides, which undergo diagnostic fragmentation by MALDI-TOF/TOF-MS/MS.^1379^ The matrix eliminates low-mass-range interference, yielding characteristic fragment patterns that distinguish disaccharide isomers differing in composition (cellobiose vs lactose), glycosidic linkage (maltose vs isomaltose), and anomeric configuration (gentiobiose vs isomaltose). Relative quantitation of binary isomer mixtures achieves linearity with *R* ^2^ > 0.988, and the method was applied to quantify maltose (0.06 molar ratio relative to sucrose) in *Medicago* leaf extracts without complex sample pretreatment, outperforming ion mobility–MS approaches that fail to baseline-separate underivatized disaccharide isomers. Building on NEDC’s core properties, ATNEDC (ammonia-modified NEDC) was screened and optimized for sensitive FFA quantitation by MALDI-FTICR MS.^1131^ ATNEDC minimizes ion suppression and matrix interference, enabling quantification of nine FFAs (*i.e.*, C_14:0_, C_16:1_, C_16:0_, C_18:0_, C_18:1_, C_18:2_, C_18:3_, C_20:4_, and C_22:6_) with excellent linearity (*R* ^2^ = 0.991–0.999), LOD (0.2–5.4 μM), and a linear dynamic range exceeding two orders of magnitude. It exhibits robust precision (interday RSD < 19%) and rapid analysis (<30 s per sample), outperforming conventional GC/LC-MS workflows. Applied to 339 serum samples, it successfully differentiates hyperglycemic patients from healthy controls and those without hyperglycemia by detecting elevated FFA levels associated with increased fasting blood glucose, highlighting its clinical utility for metabolic syndrome diagnosis and FFA biomarker screening. Extending MALDI-MS’s utility to agrochemical analysis, 2-NPG was developed for PYM quantitation, outperforming DHB and CHCA in sensitivity and precision.^421^ For MALDI-MS, 2-NPG achieves a LOD of 0.013 ppm (comparable to UPLC-MRM’s 0.008 ppm), excellent linearity (*R* ^2^ > 0.999) in matrix-matched solutions, and high recoveries with minimal matrix effects. For MALDI-MSI, it enables the first absolute quantitative imaging of PYM in heterogeneous strawberry tissues, combined with quartz crystal microbalance for localized tissue mass measurement. The matrix resolves PYM’s progressive penetration (2–10 mm depth over 4 days) and yields concentrations linearly correlated with MALDI-MS and UPLC-MRM, providing a robust tool for pesticide residue spatial quantitation in food safety assessment. Collectively, these new SOM matrices advance MALDI-MS technology by tailoring chemistries to analyte-specific properties, with each innovation addressing unmet needs across biological metabolomics and agricultural contaminant analysis, while maintaining compatibility with high-throughput and spatial imaging workflows.

Undoubtedly, the development of new matrix systems, the continuous creation or discovery of new SOM matrices, and the proposal of stricter and better experimental conditions are all crucial for further promoting the continuous wide application of MALDI-MS quantitative analysis.

## 6 Conclusions and Outlook

MALDI matrices play a crucial and multifaceted role in MALDI-MS and MALDI-MSI analyses. The selection and application of an optimal matrix are paramount for achieving efficient detection of specific compounds. In particular, the inherent properties of the matrix directly influence the sensitivity of detecting target compounds within complex mixtures.^133,174^ Over the past four decades, the continuous screening and development of novel MALDI matrices have propelled the rapid advancement of MALDI-MS technology and significantly broadened its application spectrum. Consequently, a systematic review of this topic is both timely and necessary. In this review, we first introduce the fundamental principles and experimental procedures of MALDI-MS. We then examine the latest progress in matrix research, encompassing the development and application of both traditional and novel matrices. Key aspects are highlighted as follows: (I) First-generation organic matrices: This category includes both traditional and novel SOMs, which are suitable for (+)­MALDI-MS, (-)­MALDI-MS, and (+/-)­MALDI-MS analyses. (II) Second-generation organic matrices: These matrices are designed and synthesized employing advanced strategies, including matrix derivatization, reactive matrices, ILMs, binary matrices, and the incorporation of dopants. (III) Inorganic matrices based on inorganic materials: This category encompasses metal-based, carbon-based, and silicon-based NPs, MOFs, COFs, and QDs. (IV) Organic–inorganic binary and hybrid matrices. Traditional MALDI matrices are primarily composed of small organic molecules. Through the screening of new SOMs, the addition of dopants, structural modification, polymerization, or transformation into synthetic matrices, along with the rapid development and application of inorganic NMs in MALDI-MS, MALDI matrices can now meet diverse analytical requirements. Classical matrices are widely adopted because of their broad recognition and extensive application. In contrast, novel matrices exhibit remarkable characteristics, including enhanced analyte ionization efficiency and sensitivity, increased MW to reduce matrix ion interference, reduced volatility of small molecules, and improved ability to analyze compound structures by MALDI-MS.^175–177^ Owing to the application of suitable matrices, the detectable mass range of MALDI-MS has expanded from the HMW region (for macromolecules) to the LMW region, encompassing proteins, peptides, lipids, and carbohydrates. Accordingly, this review also provides a comprehensive and systematic overview of progress in matrix applications for MALDI-MS analysis, with a specific focus on the widespread use of spatial MALDI-MS detection and imaging in proteomics, lipidomics, glycomics, and metabolomics.

Over the past four decades, with the continuous development of novel matrices and the advancement of related instrumental methods, MALDI-MSI technology has gradually emerged as a key tool for molecular visualization and high-throughput analysis in spatial multi-omics. However, this technology still faces a series of challenges. These include insufficient detection sensitivity for post-translational modifications of proteins (*e.g.*, glycosylated and phosphorylated proteins) and certain lipids (*e.g.*, PIPs, CLs, and oxPLs), difficulty in effectively distinguishing isomers, the presence of ion suppression effects, and lower quantitative accuracy than that provided by traditional tandem MS methods.^688^ Additionally, the matrices themselves present multiple challenges during application, such as unclear ionization mechanisms, low screening efficiency, and application limitations. Therefore, future study should systematically focus on elucidating matrix ionization mechanism, developing novel high-performance matrices, and expanding the application potential of MALDI-MS in quantitative detection, single-cell imaging, and the integration of spatial multi-omics.

The ionization mechanism of matrices in MALDI technology has not yet been comprehensively and uniformly explained, which is likely the core issue hindering their systematic development and design. An ideal matrix typically needs to meet several basic requirements:^841^ efficient UV laser light absorbance at a specific wavelength (*e.g.*, 355 nm) with a low tendency for photofragmentation; long-term stability under vacuum to meet the requirements of high-precision imaging; significant enhancement of the ionization efficiency of the analyte without generating interference or causing analyte aggregation; extremely low background noise in the LMW region; good chemical stability and reproducibility; and practical conditions such as simple preparation, controllable cost, and low toxicity. Other parameters such as pKa values,^52^ GB,^1382^ and proton affinity^281^ are frequently utilized to account for the differences in the ionization performance of matrices and to elucidate the final forms of analyte ions. However, there is a lack of systematic research exploring the relationship between these properties, particularly in the context of novel matrices, which merits further investigation. Although several possible ionization mechanisms have been proposed to explain the ionization behavior of matrices, such as the cluster model for organic matrices,^44^ the photoexcitation/pooling model,^212^ and the thermally driven ionization mechanism for inorganic NMs,^71,243^ there is still a lack of a universal theory to comprehensively describe the D/I physicochemical processes on the matrix surface under laser irradiation, especially how the surface properties of NMs induce ionization effects similar to those of chemical matrices.^774^ The lag in mechanism research has created a significant gap between basic theory and practical application guidelines, resulting in a lack of rational design principles for method development. This gap has also led to the long-standing reliance on empirical trials and serendipitous discoveries in matrix screening, which consume a great deal of time and manpower.^566^ Therefore, systematic and in-depth research on the D/I micromechanisms of matrices, especially organic molecules and NMs under laser irradiation, is not only an inevitable requirement for revealing the core physicochemical processes of MALDI technology but also provides key theoretical guidance for the rational design and application of next-generation high-performance matrix materials.

With ongoing progress in organic chemistry, inorganic chemistry, and materials science, the development of matrices shows infinite potential and will continue to be an important research direction in the future. To overcome existing performance bottlenecks, future research should focus on the following aspects: (I) Organic matrices: There is a need to screen or develop carefully designed matrices to enhance their ionization and detection capabilities, thereby improving the authenticity, reproducibility, and quantitative accuracy of data, and enabling the accurate determination of various important endogenous and exogenous compounds and their metabolites. (II) Inorganic matrices: Conjugated NPs with high specificity may simplify sample preparation and analysis procedures.^77^ The preparation of functionalized NPs with high specificity and efficient energy transfer can further expand NP applications in the analysis of complex samples. (III) Hybrid systems: Given the complexity and heterogeneity of biological samples, the development of multifunctional combinations of organic matrices, NMs, and mixed media is urgently needed to meet the increasing demand for analysis sensitivity, selectivity, and rapidity. (IV) Specialized matrices and complementary technologies: To overcome the challenges of low ionization yield and limited spatial resolution for some difficult-to-detect analytes and the insufficient detection of isomers, new specialized matrices (*e.g.*, reactive matrices) should be developed, tissue derivatization methods applied, or other complementary analytical techniques combined to enhance ionization effects and improve imaging quality.^1383–1385^ Recently, the introduction of artificial intelligence (AI) and machine learning (ML) technologies has injected new impetus into matrix study and development. Most matrices have been discovered by chance or through empirical methods, and the traditional “trial-and-error” approach is constrained by its time-consuming and labor-intensive nature. In contrast, goal-oriented design based on computational chemistry data and AI-generated models is gradually enabling the prediction of matrix structure performance and functional optimization. A recent study by Padilla et al. successfully used AI-guided MALDI matrix design.^1386^ They proposed a goal-oriented AI-generated model based on computational chemistry data to explore electron-transfer matrices for the mass spectrometry analysis of LMW compounds. Integrating computational chemistry and ML methods to predict matrix structure and function is expected to significantly accelerate the screening and synthesis of high-performance matrices.

At the application level, the selection of matrices and the optimization of their performance are directly related to improvements in the quantitative accuracy and spatial resolution of MALDI-MS. The ion suppression effect is the main factor limiting quantification. It manifests as a significant reduction in the signal of the target analyte and quantitative deviation. The main causes include ionization competition (*e.g.*, differences in proton affinity between the matrix and the analyte), charge competition from endogenous/exogenous interferents (*e.g.*, lipids, salts, and metal ions), and abnormal energy transfer caused by uneven matrix crystallization morphology or unsuitable ratios.^31^ Adjusting the matrix–analyte stoichiometric ratio (*e.g.*, insulin to nicotinic acid 1:600 ^1387^), using novel matrices (*e.g.*, NAPA^890^ and AgNPs@PDA^1170^), introducing dopants (*e.g.*, 2-PA^1388^, NTA^666^, AmP^1014^), and optimizing sample pretreatment (*e.g.*, tissue washing protocols^151–153^ ) can effectively alleviate the suppression effect, enhancing the reproducibility of detection in complex biological samples. In terms of spatial resolution, the uniformity of matrix crystallization and the energy transfer efficiency have a decisive impact on imaging quality, which is directly related to the uniformity of D/I and signal intensity. Achieving cellular and subcellular resolution in MALDI-MSI technology represents a key goal that depends on the coordinated progress of matrix application and instrumentation.^1152^ Recent technological innovations have demonstrated this progress: the CHCA matrix prepared by sublimation combined with AmP soaking treatment has successfully achieved high-spatial-resolution molecular imaging at the single-cell level,^1014^ the t-MALDI-2 MSI system has achieved a high pixel size of 600 nm in brain tissue,^103^ and the use of a fiber-based nanolaser probe equipped with a microlens has improved the spatial resolution to the nanometer level (subcellular level).^1389^ To further achieve robust single-cell and 3D tissue imaging in the future, breakthroughs are still needed in terms of detection sensitivity, spatial resolution, and sample preparation methods to fully realize the application potential of high-spatial-resolution MALDI-MSI in the biological sciences.^1390^

In addition, MALDI-MS and MALDI-MSI show significant potential for expanding spatial multi-omics applications because of their high-throughput characteristics and *in situ* analysis capabilities.^47,133^ With the increasing emphasis on multi-omics research, the spatial multi-omics data obtained by MALDI-MSI technology can be integrated with other transcriptomic, proteomic, and metabolomic data to provide a new perspective for systems biology that should reveal complex biological networks and assist in the discovery of potential biomarkers.^1089,1391,1392^ However, the translational potential of this technology is largely limited by a core factor: the performance and practical applicability of the matrix system. Although many newly developed matrices show good potential in a controlled laboratory environment, establishing robustness in complex clinical samples (*e.g.*, heterogeneous tissues or body fluids) still calls for rigorous validation and optimization. Future study should not only focus on developing novel MALDI matrix systems but also emphasize rigorous optimization and clinical validation, as these are the foundation for releasing the full potential of MALDI-MS technology in multi-omics and driving its in-depth application in life sciences. Firstly, systematic optimization and comprehensive validation of emerging matrix systems across a wide range of complex clinical specimens should be prioritized. This encompasses not only heterogeneous solid tissues (*e.g.*, tumor tissues) but also biofluids (*e.g.*, plasma, cerebrospinal fluid, and urine) that often contain interfering substances (*e.g.*, salts) and low-abundance analytes crucial for disease diagnosis. Validation criteria should extend beyond basic analytical performance (sensitivity, specificity, and reproducibility) to include clinical practicality metrics, such as compatibility with routine clinical sample processing workflows, rapid matrix application protocols, and scalability for large-scale clinical cohorts. Significantly, the development of novel matrix systems, such as designed and synthesized specific matrices, nanocomposites matrices, and binary or hybrid matrices, should be incorporated into this optimization and validation process to overcome the inherent limitations of current matrices, such as uneven crystallization, matrix-induced ion suppression, and poor compatibility with tissue types. Secondly, leveraging these well-optimized and clinically validated novel matrix systems to expand the application potential of MALDI-MSI in spatial multi-omics study is essential for promoting the development of MALDI-MS technology. Specifically, the high-quality spatial multi-omic data generated by these advanced matrix systems can serve as the key factors for seamless complementary integration with spatial transcriptomics and spatial epigenomics (*e.g.*, DNA methylation and histone modifications). These multidimensional spatial multi-omics networks, centered around high-performance matrix-enabled MALDI-MSI data, are poised to offer profound insights into the tissue-specific molecular heterogeneity inherent in complex diseases, including but not limited to cancer, neurodegenerative disorders, and autoimmune diseases. Moreover, they will expedite the discovery of spatially resolved biomarkers that hold substantial clinical translational value. Therefore, the development, optimization, and validation of novel matrix systems are not only pivotal steps but also among the core driving forces for the further advancement of MALDI-MS technology in life sciences. These endeavors will accelerate the translation of MALDI-based spatial multi-omics from preclinical investigations to clinical applications, thereby facilitating precision diagnosis, personalized treatment selection, and therapeutic response monitoring. Moreover, they will unlock new horizons in basic study, such as elucidating the molecular-level spatial regulation of embryonic development, tissue regeneration, and the maintenance of tissue homeostasis.

Finally, establishing a standardized MALDI matrix database, along with shared repositories for matrix-related protocols and spectral data, will significantly enhance data accessibility, improve research consistency, and accelerate the development of quantitative imaging technologies. Multiple studies have emphasized the importance of standardization and universal guidelines, calling for the establishment of centralized hubs to share imaging data sharing,^1393,1394^ repositories that curate raw data, validated results, and experimental metadata to maximize data utility. A dedicated MALDI matrix database would serve as a key resource, enabling researchers to systematically compare matrix performance, select optimal matrices for specific analytes and applications, and standardize preparation protocols. Rendering MALDI-MS a more reliable and reproducible technique, by fostering global collaboration and consensus among researchers,^1395–1397^ is critical. Concrete steps include: developing a widely accessible database of MALDI matrices, including their physicochemical properties, application notes, and reference spectral data; establishing consensus best practices for matrix selection and sample preparation to ensure technical reliability and reproducibility; and expanding online resources (*e.g.*, matrix tutorials, application guides) to lower barriers to adoption.

Overall, this review synthesizes advances in matrix development and their implications for MALDI-MS analytical strategies and applications. In addition to classical matrices, hundreds of new matrices are being developed through the screening of new SOM matrices, matrix derivatization, the design and synthesis of ILMs, and the application of NMs. Although these novel matrices may not completely replace the classical ones, the rational design of matrices with molecular recognition properties will extend the applicability of MALDI ionization mechanisms to a wider range of compounds and is expected to find new and interesting applications in targeted MALDI-MS or MALDI-MSI analysis. More excitingly, matrix studies are gradually moving from empirical exploration to a new stage that combines mechanism-driven exploration and intelligent design. By systematically elucidating ionization mechanisms, accelerating the development of high-performance matrices with the help of AI, optimizing matrix crystallization processes, and establishing standardized databases and sharing platforms, MALDI technology is expected to undergo breakthroughs in quantitative accuracy, spatial resolution, and multi-omics applicability. To date, the application of MALDI-MS technology in spatial proteomics, spatial metabolomics, spatial lipidomics, and spatial glycomics has significantly enhanced our understanding of molecular interactions and complex biological problems. Looking ahead, with continued breakthroughs in matrix performance and the ongoing refinement of multi-omics integration paradigms, MALDI-MS will evolve into a comprehensive, high-precision, high-throughput core platform for chemical analysis and molecular imaging, which will play an increasingly critical and transformative role in systems biology research, the exploration of physiological/biochemical mechanisms, and clinical translational applications.

## Supplementary Material



## Notes

Overview of preferred ionization modes and applications for various MALDI matrices, including small organic molecules, designed and synthesized organic matrices, common-used dopants, inorganic matrices, and organic–inorganic binary/hybrid matrices, and the summaries of representative MALDI matrices for specific analytical fields, namely proteomics, metabolomics, lipidomics, glycomics, nucleic acid analysis, and quantitative analysis (PDF)

## Abbreviations

1-AP: 1-aminopyrene
2,3,4-DMNI: 2,3-dimethyl-4-nitro-1*H*-indole
2,3,6-DMNI: 2,3-dimethyl-6-nitro-1*H*-indole
2,4,6-THAP (or THAP): 2,4,6-trihydroxyacetophenone
2,4-D: 2,4-dichlorophenoxyacetic acid
2,4-DHAP: 2,4-dihydroxyacetophenone
2,4-DHBA: 2,4-dihydroxybenzaldehyde
2,5-cDHA: (*E*)-4-(2,5-dihydroxyphenyl)­but-3-en-2-one
2,5-DHAP: 2,5-dihydroxyacetophenone
2,6-DHAP (or DHAP): 2,6-dihydroxyacetophenone
2A4M5NP: 2-amino-4-methyl-5-nitropyridine
2D: two-dimensional
2-DHBA: 2,4-dihydroxybenzaldehyde
2-HPM: 2-hydrazinopyrimidine
2-HQ: 2-hydrazinoquinoline
2-HTA: 2-hydrazinoterephthalic acid
2-MBT: 2-mercaptobenzothiazole
2-NPG: 2-nitrophloroglucinol
2-P: 2-pyridine
2-PA: 2-pyridinecarboxylic acid
3,4-MNI: 3-methyl-4-nitro-1*H*-indole
3,6-MNI: 3-methyl-6-nitro-1*H*-indole
3,7-DHF: 3,7-dihydroxyflavone
3-APA: 3-aminopicolinic acid
3-APH: 3-aminophthalhydrazide
3-AQ: 3-aminoquinoline
3-CACA: α-cyano-3-aminocinnamic acid
3-CF3-BTD: 5-(3-trifluoromethylbenzylidene)­thiazolidine-2,4-dione
3D: three-dimensional
3D-MG: 3D mesoporous graphene
3D-MTP: 3D printed MALDI target plate
3H2NBA: 3-hydroxy-2-nitrobenzoic acid
3H4NBA: 3-hydroxy-4-nitrobenzoic acid
3-HC: 3-hydroxycoumarin
3-HF: 3-hydroxyflavone
3-HNAH: 3-hydroxy-2-naphthoic acid hydrazide
3-HPA: 3-hydroxycholanic acid
3M4HBA: 3-methoxy-4-hydroxybenzoic acid
3-MPA: 3-mercaptopropionic acid
3-NPH: 3-nitrophthalhydrazide
3-P: 3-pyridine
4-AC: 4-aminoisoquinoline-3-carboxamide
4-APH: 4-aminophthalhydrazide
4-HQ: 4-hydrazinoquinoline
4-NC: 4-nitrocatechol
4-NI: 4-nitro-1*H*-indole
5,1-ANL: 5-amino-1-naphthol
5-ASA: 5-aminosalicylic acid
5-HF: 5-hydroxyflavone
5-HT: 5-hydroxytryptamine
6-BA: 6-benzylaminopurine
6-BFA: 6-bromo-ferulic acid
6-TG: 6-thioguanine
7,3′,4′-THF: 7,3′,4′-trihydroxyflavone
9,10-DPA: 9,10-diphenylanthracene
9-AA: 9-aminoacridine
9-NA: 9-nitroanthracene
A: adenine
AA: anthranilic acid
AAB: 4-aminoazobenzene
AAN: 2-(2-aminoethyloamino)-5-nitropyridine
AAs: amino acids
ABA: abscisic acid
AC: ammonium citrate
ACA: 4-aminocinnamic acid
ACC: 1-aminocyclopropane-1-carboxylic acid
AcCar: acetylcarnitine
Ach: acetylcholine
AD: Alzheimer’s disease
ADCN: 1-amino-2,4-dichloronaphthalene
ADFA: 2-amino-4,5-diphenylfuran-3-carboxylic acid
ADHB: alkylated dihydroxybenzoic acid
ADP: ammonium dihydrogen phosphate
AFA: aquatic fulvic acid
AgBz: silver benzoate
AgNO_3_: silver nitrate
AGO: aggregated GO
AgTFA: silver trifluoroacetate
AgTS: silver *p*-toluenesulfonate
AHB: 3-amino-4-hydroxybenzoic acid
AHC: alkylated hydroxychalcone
AIE: aggregation-induced luminescence
Al: aluminum
Ala: alanine
ALCs: acylcarnitines
AmP: ammonium phosphate monobasic
AMT: 5-amino-2-mercapto-1,3,4-thiadiazole
AN: ammonium nitrate
AnCCA: (2*E*)-3-(anthracen-9-yl)-2-cyano-propan-2-enoic acid
ANI: aniline
ANP: 2-amino-5-nitropyridine
AOG: nitric acid oxidized G
AP: aminopyrazine
APCI: atmospheric pressure chemical ionization
API: atmospheric pressure ionization
AP-MALDI: atmospheric pressure MALDI
Apt-AuNPs: aptamer-modified AuNPs
APTES: 3-aminopropyltriethoxysilane
AP-TM-MALDI-MSI: atmospheric pressure transmission-mode MALDI-MSI
Apt-SWNHs: aptamer-modified single-walled carbon nanocones
Arg: arginine
Asc: ascorbate
ASE: analyte suppression effect
ATHAP: alkylated trihydroxyacetophenone
ATNEDC: ammonia-treated *N*-(1-naphthyl)­ethylenediamine dihydrochloride
ATP: adenosine triphosphate
ATRP: atom-transfer radical polymerization
ATT: 6-aza-2-thiothymine
Au@MnO: AuNPs on MnO
AUC: area under the curve
AuNPs-CBS: citrate-capped AuNPs
BaTiO_3_ NPs: barium titanate NPs
BB7: Basic Blue 7
B-CNWs: boron-doped CNWs
B-COFs: boronic acid-functionalized COFs
Bi_2_O_3_@GO: bismuth oxide-GO
BMQI: 6-borono-1-methylquinoline-1-ium
BNDM: 1,1′-binaphthyl-2,2′-diamine
BNQDs: boron nitride QDs
BNS-MQDs: ternary-doped MXene QDs
BOA: *o*-benzylhydroxylamine
BP: black phosphorus
BP@Au: black phosphorus nanosheet-gold
BPh: benzophenone
BR: brassinosteroid
Br-C60: bromoacetyl functionalized C60
BSA: bovine serum albumin
BSPD: *N*,*N*′-bis­(salicylidene)*p*-phenylenediamine
BSPD-OH: *N*,*N*′-bis­(4-hydroxysalicylidene)*p*-phenylenediamine
BSPD-OMe: *N*,*N*′-bis­(4-methoxysalicylidene)­p-phenylenediamine
BzPy: 2-benzoylpyridine
C: cytosine
HSBF: hexa­(sulfonbutyl)­fullerene (C60­[(CH_2_)_4_SO_3_^–^]_6_)
CA: caffeic acid
Car: carnitine
CCPBA: [(*E*)-4-(2-cyano-2-carboxyethenyl)­phenyl]­boronic acid
ccRCC: clear cell renal cell carcinoma
CDH1: cadherin 1
CDK1: cyclin-dependent kinase-1
CDs: carbon dots
CdS: cadmium sulfide
CdSe: cadmium selinide
CdTe: cadmium telluride
CeO_2_: cerium oxide
CerPs: ceramide phosphates
Cers: ceramides
CEs: cholesterol esters
CFG: Consortium for Functional Glycomics
CHCA: α-cyano-4-hydroxycinnamic acid
CHCAB: α-cyano-4-hydroxycinnamic acid butylamine
CHCA-C3: (*E*)-α-cyano-4-hydroxycinnamic acid propyl ester
CHCA-SiO_2_: CHCA-modified SiO_2_ hybrid materials
CHCE: α-cyano-4-hydroxycinnamic methyl ester
CHC-Mal: (*E*)-2-cyano-*N*-(2-(2,5-dioxo-2,5-dihydro-1*H*-pyrrol-1-yl)­ethyl)-3-(4-hydroxyphenyl)­acrylamide
CK: cytokinin
ClCCA: 4-chloro α-cyanocinnamic acid
CLs: cardiolipins
CMBT: 5-chloro-2-mercaptobenzothiazole
CMC: carboxymethyl cellulose
CNAA: (*E*)-2-cyano-3-(naphthalen-2-yl)­acrylic acid
CNCs: cellulose nanocrystals
CNDA: (2*E*,4*E*)-2-cyano-5-(4-nitrophenyl)­penta-2,4-dienoic acid
CNFs: carbon nanofibers
CNHs: carbon nanohornes
CNTs: carbon nanotubes
CNWs: carbon nanowalls
Co: cobalt
Co­(OH)_2_: cobalt hydroxide
Co_3_O_4_: tricobalt tetroxide
COFs: covalent organic frameworks
COPD: chronic obstructive pulmonary disease
CPCD: coupled photophysical and chemical dynamics
CPPA: α-cyano-5-phenyl-2,4-pentadienoic acid
CPT-11: irinotecan
CS: chondroitin sulfate
CsCl: cesium chloride
CSNPs: core-shell NPs
CTAB: hexadecyltrimethylammonium bromide
CTICL: charge transfer-induced chemiluminescence
Cu_2_O PS@DHB: Cu_2_O PS combined with DHB
CuCl: copper chloride
CuO: copper oxide
CVD: chemical vapor deposition
Cys: cysteine
D/I: desorption/ionization
D4-CHCA: D4-α-cyano-4-hydroxycinnamic acid
DA: dopamine
D-A COFs: donor-acceptor COFs
DAA: 9-(3,4-diaminophenyl)­acridine
DABP: 3,4-diaminobenzophenone
DAGs: diacylglycerols
DAHC: diammonium hydrogen citrate
DAN: 1,5-diaminonaphthalene
DB: double bond
DBDA: *N*1,*N*4-dibenzylidenebenzene-1,4-diamine
DC: dansylcadaverine
DCA: 6,7-dihydroxycoumarin-3-carboxylic acid
DCBTA: 2-[5-(2,4-dichlorobenzoyl)-2-thienyl]­acetic acid
DCH: 2,3-dicyanohydroquinone
DCHA: dicyclohexylamine
DCPH: 2,4-dicarboxylphenylhydrazine
DCTB: 2-[(2*E*)-3-(4-*tert*-butylphenyl)-2-methylprop-2-enyl]­propanedinitrile
DDTC-AuNPs: dithiocarbamate-functionalized AuNPs
DEA: *N*,*N*-diethylaniline
DH: dansyl hydrazine
DHB: 2,5-dihydroxybenzoic acid
DHB/3HBA: 3-hydrazinobenzoic acid plus DHB
DHB/Q3CH: quinoline-3-carbohydrazide plus DHB
DHBB: 2,5-dihydroxybenzoate butylamine
DHNA: 1,4-dihydroxy-2-naphthoic acid
DHNBA: 2,4-dihydroxy-5-nitrobenzoic acid
DHPT: 2,3,4,5-tetrakis­(3′,4′-dihydroxyphenyl)­thiophene
DHT: 2,5-dihydroxyterephthalic acid
DHZ: dihydrozeatin
Di-FCCA: α-cyano-2,4-difluorocinnamic acid
DIOS: desorption/ionization on porous silicon
DIPEA: *N*,*N*-diisopropylethylammonium
DIT: 1,8,9-trihydroxyanthracene
DIUTHAME: hole alumina membrane
DMA: *N*,*N*-dimethylaniline
DMABA: 4-dimethylaminobenzaldehyde
DMACA: 4-(dimethylamino)­cinnamic acid
DMAN: 1,8-bis­(dimethylamino)­naphthalene
DMAPA: 3-(dimethylamino)-1-propylamine
DMCA: 3,4-dimethoxycinnamic acid
DMED: *N*,*N*-dimethylethylenediamine
DMNB: 4,5-dimethoxy-2-nitrobenzene
DMNB-2,5-DHAP: 4,5-dimethoxy-2-nitrobenzyl-2,5-dihydroxyacetophenone
DMNTH: 4-dimethylamino-6-(4-methoxy-1-naphthyl)-1,3,5-triazine-2-hydrazine
DMSA: *meso*-2,3-dimercaptosuccinic acid
DNA: deoxyribonucleic acid
DNPH: 2,4-dinitrophenylhydrazine
DPH: 1,6-diphenyl-1,3,5-hexatriene
DPN: 1,8-di­(piperidinyl)-naphthalene
DPP: 2,4-diphenylpyridinium
DPP-TFB: 2,4-diphenyl-pyranylium tetrafluoroborate
DQ: diquat
DS: dermatan sulfate
DSP: 3,3′-dithiodipropionic acid di­(*N*-hydroxysuccinimide ester)
DT: dithranol, 1,8-dihydroxy-9-anthrone
DTCA: 1,4-dioxo-1,2,3,4-tetrahydrophthalazine-6-carboxylic acid
DTT: dithiothreitol
DUA: 11-(dansylamino)­undecanoic acid
EBT: elevated bowtie
EDTA: ethylenediamine tetraacetic acid
EMT: 5-ethyl-2-mercaptothiazole
EPCAM: epithelial cell adhesion molecule
ESI: electrospray ionization
ET: electron transfer
ETH: ethyne
F20TPP: *meso*-tetrakis porphyrin
FA: ferulic acid
FAB: fast atom bombardment
FAs: fatty acids
f-CNTs: functionalized CNTs
FD: field desorption
FDTS: fluorocarbon-based hydrophobic perfluorodecyltrichlorosilane
Fe_2_O_3_: iron­(III) oxide
Fe_3_O_4_: magnetic iron oxide
FeNPs: ferric nanoparticles
FF: flufenamic acid
FFPE: formalin-fixed and paraffin-embedded
FGFR4: fibroblast growth factor receptor 4
F-iCOF: fluoro-functionalized ionic COF
FMA: formic acid
FMP: 2-fluoro-1-methyl pyridinium
FPhBA: 4-formylphenylboronic acid
FT-ICR: fourier transform ion cyclotron resonance
G: graphene
G/SiO_2_: graphene-coated silicon wafer
G_2_CHCA: bis-1,1,3,3-tetramethylguanidinium α-cyano-4-hydroxycinnamate
G_3_CA: bis-1,1,3,3-tetramethylguanidinium *p*-coumaric acid
GA: gibberellin
GA3: gibberellin A3
GABA: gamma-aminobutyric acid
GB: gas-phase basicity
GC: gas chromatography
g-C_3_N_4_: graphitic carbon nitride
GCB: graphite black
G-CN: cyanographene
GD: graphdiyne
GeNDs: germanium nanodots
Gln: glutamine
GLs: glycerolipids
Glu: glutamic acid
Gly: glycine
Gly-3AQ: glycosyl-3-aminoquinoline
gNG: gas-phase nitrogen-doped graphene
GNs: graphene nanosheets
GO: graphene oxide
GO-VPBA: 4-vinylphenylboronic acid-functionalized GO
GPC: glycerophosphocholine
GPLs: glycerophospholipids
GSH: glutathione
GSLs: glycosphingolipids
GSSG: glutathione disulphide
GTHAP: 1,1,3,3-tetramethylguanidine-2,4,6-trihydroxyacetophenone
HA: hyaluronic acid
HABA: 2-(4-hydroxyphenylazo)­benzoic acid
HAs: humic acids
h-BNNs: hexagonal boron nitride nanosheets
HCA: 7-hydroxycoumarin-3-carboxylic acid
H-COF-SO_3_H: sulfonic acid-functionalized hierarchical porous COFs
HCys: homocysteine
HER2: human epidermal growth factor receptor type 2
HexCers: hexosylceramides
HGQDs: heteroatom-doped graphene QDs
HILIC: hydrophilic interaction chromatography
His: histidine
HMPPA: 4-hydroxy-3-methoxyphenylpyruvic acid
HMW: high-molecular-weight
HNBN: 4-hydroxy-3-nitrobenzonitrile
HNE: 4-hydroxy-2-nonenal
HNTP: 2-hydroxy-5-nitro-3-(trifluoromethyl)­pyridine
HOA: 2-hydroxy octadecanoic acid
HOFs: hydrogen-bonded organic frameworks
Ho-TiO_2_: holmium-modified titanium dioxide nanocomposites
HP: heparin
HPGPC: high performance gel permeation chromatography
HPLC: high-performance liquid chromatography
HS: heparan sulfate
HYNIC: hydrazinonicotinic acid
HZN: hydralazine
IAA: *trans*-3-indoleacrylic acid/3-indoleacetic acid
IgM: immunoglobulins
ILM: ionic liquid matrix
ILs: ionic liquids
IM: 1-methylimidazole
IMAC: immobilized metal ion affinity chromatography
IMTBA: *N*-isopropyl-*N*-methyl-*t*-butylammonium
InAA: indoleacrylic acid
iP: 6-(γ γ-dimethylallyl amino)­purine
IPA: indole-3-pyruvic acid
ISD: in-source decay
ISL: isoliquiritigenin, 4,2′,4′-trihydroxychalcone
IT: ion trap
JA: jasmonic acid
KS: keratan sulfate
LA-LDI-MS: label-assisted LDI-MS
Leu: leucine
LFPs: latent fingerprints
Li_2_DHT: lithium 2,5-dihydroxyterephthalate
LiBA: lithium benzoate
LiDHB: lithium 2,5-dihydroxybenzoate
LiDMB: lithium 2,5-dimethoxybenzoate
LiSA: lithium sinapate
LiSalA: lithium salicylic acid
LISMAs: silicon microcolumn arrays
LiVA: lithium vanillate
LMW: low-molecular-weight
LPAs: lysophosphatidic acids
LPCs: lysophosphatidylcholines
LPEs: lysophosphatidylethanolamines
LPGs: lysophosphatidylglycerols
LPIs: lysophosphatidylinositols
LPLs: lysophospholipids
LPSs: lysophosphatidylserines
LSIMS: liquid secondary ion mass spectrometry
Lumazine: 1*H*-pteridine-2,4-dione
Lys: lysine
MA: mefenamic acid
MALDI: matrix-assisted laser desorption/ionization
MAOG: G composite material
MAPS: 4-maleic anhydride proton sponge
matrix@MNP: matrix functionalized Fe_3_O_4_ MNPs
MBA: 4-mercaptobenzoic acid
MCF: mesoporous silica foam
mCOF@PEI@B­(OH)_2_: boric acid-functionalized magnetic COF
MDPNA: methanediphosphonic acid
MEK: Michler’s ethyl ketone
ME-SIMS: matrix-enhanced SIMS
Met: methionine
MetS2BT: 5,5′-bis­(methylthio)-[2,2′]­bithiophene
MHAs: magnetic HAs
MMA: 7-mercapto-4-methylcoumarin
Mn: manganese
MOFs: metal-organic frameworks
MP2CA: *N*-methylpyridinium-2-carbaldehyde iodide
MPA: mercaptopropionic acid
MPLA: monophosphoryl lipid A
MPNs: metal-polyphenol networks
MS: mass spectrometry
MSA: 5-methoxysalicylic acid
MSE: matrix suppression effect
mT: meta-topolin
MT3P: 3-[5'-(methylthio)-2,2'-bithiophen-5-ylthio]­propanenitrile
MUA: 11-mercaptoundecanoic acid
MWCNTs@PANI: multi-walled CNTs (MWCNTs) and polyaniline (PANI) composite
MXenes: transition metal carbides
NA: nicotinic acid
NAA: naphthalene acetic acid
NAPAs: nanopost arrays
NBS: 2-nitrobenzenesulfenyl
N-CDs: N-doped luteolin-based carbon dots
NCPH: 2-nitro-4-carboxyphenylhydrazine
ND: nanodiamond
NE: norepinephrine
N-EA: *N*-ethylaniline
NEDC: *N*-(1-naphthyl) ethylenediamine dihydrochloride
NEDN: *N*-(1-naphthyl) ethylenediamine dinitrate
NHHC: 1-naphthylhydrazine hydrochloride
NHS: *N*-hydroxysuccinimide ester
NI: nitroindole
NIMS: nanostructure-initiator MS
NiO: nickel oxide
N-MA: *N*-methylaniline
NMR: nuclear magnetic resonance
NMs: nanomaterials
NPFs: nanoporous films
NPs: nanoparticles
NR: nanorod
NR-AuNPs: Nile Red-adsorbed AuNPs
NRM: nor-harmane (9*H*-pyrido­[3,4-*b*]­indole)
nSi: nanofilament silicon
NTA: nitrilotriacetic acid
NTf2: bis­(trifluoromethane)­sulfonimide
NTs: neurotransmitters
nZVI: nano-zero-valent iron
OCT: optimal cutting temperature
ODT: octadecanethiol
OGP: *n*-octyl-β-d-glucopyranoside
OMCs: organic metal chalcogenides
O-P: N-C/G, O-P, and N-doped C/G composites
oxPLs: oxidized phospholipids
P2NA: *N*-phenyl-2-naphthylamine
P3OT: poly­(3-octylthiophene-2,5-diyl)
PA: phosphoric acid
PAA: porous anodic alumina
*p*-AAB: *p*-aminoazobenzene
PAAG: polyacrylamide gel
PAHs: polycyclic aromatic hydrocarbons
PAPAN: 2-phenyl-3-(*p*-aminophenyl) acrylonitrile
PAs: phosphatidic acids
PB: Paternò–Büchi
PBDPP: 1,4-phenylene-4,4'-bis­(2,6-diphenyl-4-pyrylium)
PCA: pyrazinecarboxylic acid
PCR: polymerase chain reaction
PCs: phosphatidylcholines
PD: phenylenediamine
Pd: palladium
PDA: polydopamine
PEG 200: polyethylene glycol 200
PEs: phosphatidylethanolamines
PFCs: perfluorinated compounds
PFOA: perfluorooctanoic acid
PFOS: perfluorooctane sulfonate
PFPT3P: 3-(5′-pentafluorophenylmethylsulfanyl-[2,2′]­bithiophenyl-5-ylsulfanyl)­propionitrile
PFS: potassium perfluorinated sulfonate
PGC: porous graphitic carbon
PGs: phosphatidylglycerols
Ph-CCA-NH_2_: 4-phenyl-α-cyano-cinnamic acid amide
Phe: phenylalanine
PIPs: phosphatidylinositol phosphates
PIs: phosphatidylinositols
PLs: phospholipids
PMC: *p*-methoxy cinnamaldehyde
PN: 1,8-bisphosphazenylnaphthalene
PNA: *p*-nitroaniline
PPD: *p*-phenylenediamine
PPG2000: polypropylene glycol 2000
ppt: parts per trillion
PQ: paraquat
Pro: proline
PRs: polyoxymethylenes
PSD: post-source decay
pSer: phosphoserine
pSi: porous silicon
PSs: phosphatidylserines
Pt: platinum
pThr: phosphothreonine
pTyr: phosphotyrosine
PV: phenylenevinylene
PVDF: polyvinylidene fluoride
PVP: polyvinylpyrrolidone
PYM: pyrimethanil
Pyr: pyridine
QA: quinaldic acid
QACs: quaternary ammonium compounds
QDs: quantum dots
Q-TOF: quadrupole-TOF
R: rhodamine
RCBs: ricinus communis biomarkers
*r*-CD: *r*-cyclodextrin
rGO: reduced GO
RNA: ribonucleic acid
RNase B: ribonuclease B
RTILs: room temperature ILs
S/N: signal-to-noise
SA: sinapinic acid
SALDI-MS: surface-assisted LDI-MS
SBA-15: Santa Barbara amorphous-15
SDS: sodium dodecyl sulfate
Si: silicon
SinTri: 3,5-dimethoxycinnamic acid triethylamine
SiNWs: silicon nanowires
Si-O: siloxane backbone
SiO_2_ NPs: silica nanoparticles
siRNA: small interfering RNA
SLs: sphingolipids
SMALDI: scanning microprobe MALDI
SMs: sphingomyelins
Sn: tin
SNP: single nucleotide polymorphism
SOMs: small organic molecules
SOS: sodium octyl sulfate
SPD: spermidine
STs: sterol lipids
SWNHs: single-walled carbon NHs
T: thymine
TA: tannic acid
TAGs: triacylglycerols
TBA: tributylamine
TBBPA: tetrabromobisphenol A
TBBPS: tetrabromobisphenol S
TCEP: tris­(2-carboxyethyl)­phosphine
TCNQ: 7,7,8,8-tetracyanoquinodimethane
TETA-SPM: tetraamine spermine
Tf: transferrin
TFA: trifluoroacetic acid
TFLNH: trifucosyllacto-*N*-hexaose
TFpLNH: trifucosyl-*para*-lacto-*N*-hexaose
TGA: hioglycolic acid
THF: tetrahydrofuran
Thr: threonine
TIMS: trapped ion mobility spectrometry
TiO_2_: titanium dioxide
TiO_2_-DA: DA-functionalized TiO_2_ NPs
TLC: thin-layer chromatography
TMG: 1,1,3,3-tetramethylguanidine
TMGN: 1,8-bis­(tetramethylguanidino)-naphthalene
TMNTA: 4-trimethylamino-6-(4-methoxy-1-naphthyl)-1,3,5-triazine-2-(3-aminophenylboronic acid
TMP: 2,4,6-trimethylpyrylium
TPPN: 1,8-bis­(tris-pyrrolidinyl-phosphazenyl)­naphthalene
Trip: tripropylammonium α-hydroxycinnamate
Trp: tryptophan
TSA: thiosalicylic acid
TTF: tetrathiafulvalene
Tyr: tyrosine
*t*Z: *trans*-zeatin
U: uracil
UHMW: ultra-high molecular weight
UV: ultraviolet
Val: valine
W: tungsten
WCS NPs: washed candle soot nanoparticles
WTiO: tungsten-titanium oxide
Zn: zinc
ZnO: zinc oxide
ZnSe: zinc selenium
