# Supplementary material for: MALDI Matrix: Origins, Innovations, and Frontiers
Source: Chem Rev. 2026 Feb 3;126(5):3324–528. doi: 10.1021/acs.chemrev.5c00786 (PMC12983221; doi:10.1021/acs.chemrev.5c00786)
Supplement: Supplementary file 1 [file cr5c00786_si_001.pdf]

Supporting Information

MALDI Matrix: Origins, Innovations, and Frontiers

Ran Wu,[†,#] Rui Liu,[‡,#] Hao Hu,[§,#] Liang Qin,[ǁ] Lulu Chen,[ǁ] Zhibin Bao,[ǁ] Jinxiang Fu,[‡] Hua Guo,[‡] Lei Wang,[⁑] Anna Wang,[‡] Zihan Wang,[‡] Chenyu Yang,[†] Xiangrui Cheng,[‡] Difan Chen,[‡] Haiqiang Liu,[¶] Yanping Jing,[†] Shuai Guo,[††] Yujie Fu,[O,*] and Xiaodong Wang[†,‡,*]

[†] College of Biological Sciences and Biotechnology, Beijing Forestry University, Beijing 100083, China.

[‡] College of Life and Environmental Sciences, Minzu University of China, Beijing 100081, China.

[§] State Key Laboratory of Natural and Biomimetic Drugs School of Pharmaceutical Sciences, Peking University, Beijing 100191, China.

[ǁ] HIT Research Center, Ltd, Beijing 100094, China.

[⁑] State Key Laboratory of Biotherapy and Cancer Center, West China Hospital, Sichuan University, Chengdu 610064, China

[¶] Xiangtan Environmental Science Research Institute, Xiangtan 411100, China.

[††] School of Basic Medicine and Clinical Pharmacy, China Pharmaceutical University, Nanjing 210009, China.

[O] State Key Laboratory of Efficient Production of Forest Resources, Beijing Forestry University, Beijing 100083, China

[#]These authors contributed equally to this work.

***Corresponding authors:**

Prof. Xiaodong Wang, Ph.D

College of Biological Sciences and Biotechnology, Beijing Forestry University, Beijing 100083, China

#35 Qinghua East Road, Haidian District, Beijing, 100083, China

College of Life and Environmental Sciences, Minzu University of China, Beijing 100081, China

#27 Zhongguancun South Avenue, Beijing, 100081, China

Email: Xiaodong@muc.edu.cn; xiaodongwang@bjfu.edu.cn

Prof. Yujie Fu, Ph.D

State Key Laboratory of Efficient Production of Forest Resources, Beijing Forestry University, Beijing 100083, China

#35 Qinghua East Road, Haidian District, Beijing, 100083, China

Email: yujie_fu@163.com

Table of Contents

Supporting Information Table S1. Properties and applications of commonly used organic matrices in MALDI-MS S-3

Supporting Information Table S2. Properties and applications of commonly used inorganic matrices in MALDI-MS. S-7

Supporting Information Table S3. Small organic molecule (SOM) matrices with their preferred ionization mode and applications for MALDI-MS/MSI........ S-9

Supporting Information Table S4. Designed and synthesized organic matrices with their preferred ionization mode and applications for MALDI-MS/MSI........ S-17

Supporting Information Table S5. Common-used dopants in organic matrices for MALDI-MS/MSI. S-27

Supporting Information Table S6. Inorganic matrices with their preferred ionization mode and applications for MALDI-MS/MSI. S-29

Supporting Information Table S7. Organic-inorganic binary and hybrid matrices with their preferred ionization mode and applications for MALDI-MS/MSI........ S-41

Supporting Information Table S8. Representative MALDI matrices for proteomic analysis S-43

Supporting Information Table S9. Representative MALDI matrices for metabolomic analysis S-48

Supporting Information Table S10. Representative MALDI matrices for lipidomic analysis S-52

Supporting Information Table S11. Representative MALDI matrices for glycomic analysis........ S-56

Supporting Information Table S12. Representative MALDI matrices for nucleic acid analysis S-60

Supporting Information Table S13. Representative MALDI matrices for MALDI-MS quantitative analysis S-62

REFERENCES S-72

Supporting Information Table S1. Properties and applications of commonly used organic matrices in MALDI-MS.

Matrix class	Matrix names	Acronyms	pKa*	GB(M) (kJ·mol^{-1})*	Matrix source	Ionization mode	Analytes	Ref.
SOM matrices	2,5-Dihydroxybenzoic acid	DHB	2.97[a]	822.5 ± 15,5[a]	Commercially purchased	Positive	Proteins	1
						Dual-Polarity	Lipids	2
						Positive	Quercetin, myricetin, luteolin and kaempferol	3
						Positive	*N*-glycans and extracellular matrix peptides	4
						Negative	Oligonucleotides	5
SOM matrices	Sinapinic acid	SA	4.47[a]	867.4 ± 1.4[a]	Commercially purchased	Positive	Monoclonal human immunoglobulin IgM	6
						Positive	Immune complexes	7
						Negative	Neutral/sulfated carbohydrates	8
						Positive	Commercial peptides and model peptides with different amino acid side chains	9
						Positive	Cysteine-containing peptides	10
SOM matrices	α-Cyano-4-hydroxycinnamic acid	CHCA	1.17[b]	908.1 ± 0.8[a]	Commercially purchased	Positive	Peptides and proteins	11
						Positive	Thymosin β4	12
						Dual-Polarity	Vitamins A1, B6, and C	13
						Positive	Phospholipids	14
						Positive	*N*-glycans and lipids	15
SOM matrices	9-Aminoacridine	9-AA	9.99[a]	989.10[a]	Commercially purchased	Negative	LMW compounds	16
						Negative	Phenols, carboxylic acids, sulfonates, aldehydes, plant hormones, and bile acids	17,18
						Negative	Flavones	19
						Negative	Cellular glycerophospholipids	20
						Negative	Rat brain lipids	21

Matrix class	Matrix names	Acronyms	pKa*	GB(M) (kJ·mol^{-1})*	Matrix source	Ionization mode	Analytes	Ref.
SOM matrices	2,4,6-Trihydroxyacetophenone	THAP	7.80[a]	864.2 ± 7.6[a]	Commercially purchased	Dual-Polarity	Noncovalent RNA-peptide complexes	22
						Negative	Natural and modified oligonucleotides	23
						Positive	Polysaccharides	24
						Positive	Neutral storage lipid, polar membrane lipid, and glycosphingolipids	25
						Positive	Fructooligosaccharides in red onions, shallots, and elephant garlic	26
Reactive matrices	1,5-Diaminonaphthalene	DAN	2.60[a]	875 ± 8[a]	Commercially purchased	Positive	Proteins	27
						Positive	Human urotensin II, human guanylin, and bovine insulin	28
						Negative	Phospholipids and low molecular weight metabolites in maize leaves	29
						Dual-Polarity	Lipidome	30
						Positive	Gangliosides in rat brain tissue	31
SOM matrices	3-Hydroxypicolinic acid	3-HPA	1.14[b]	876.3 ± 10.2[a]	Commercially purchased	Dual-Polarity	Underivatized single-stranded DNA oligomers	32
						Dual-Polarity	Oligonucleotides	33
						Negative	Oligonucleotides	5
						Negative	Oligonucleotides	34
						Dual-Polarity	MicroRNA biomarkers	35
SOM matrices	Dithranol	DT	7.16[b]	N/A	Commercially purchased	Positive	Lipids	36
						Positive	Lipids in rat brain	37
						Positive	Protected synthetic peptides	38
						Positive	Phytosterol	39
						Positive	Synthetic polymers	40
SOM matrices	6-Aza-2-thiothymine	ATT	7.50[a]	835.0[a]	Commercially purchased	Negative	Oligonucleotides	41
						Positive	Intact double-stranded DNA	42

Matrix class	Matrix names	Acronyms	pKa*	GB(M) (kJ·mol^{-1})*	Matrix source	Ionization mode	Analytes	Ref.
						Positive	Specific noncovalent complexes between guanidinium derivatives and single-stranded DNA	43
						Positive	Tryptic peptides and map in situdigested peptides	44
						Positive	Underivatized glycans, oligosaccharide derivatives, glycopeptides, and glycolipids	45
Reactive matrices	3-Aminoquinoline	3-AQ	4.95[a]	N/A	Commercially purchased	Dual-Polarity	Oligosaccharides	46
						Dual-Polarity	Glycopeptides and phosphopeptides	47
						Positive	Angiotensin I and protein enzymatic digest products	48
						Positive	Glycoproteins	49
						Positive	N-Glycans	50
Reactive matrices	2-[(2E)-3-(4-tert-Butylphenyl)-2-methylprop-2-enylidene] malononitrile	DCTB	N/A	N/A	Commercially purchased	Dual-Polarity	Coordination compounds, organometal lics, and conjugated organic compounds	51
						Positive	Polythiophene	52
						Negative	Very labile compounds	53
						Positive	Amino groups	54
						Positive	Noninflammatory Type III Rotaxane Dendrimers	55
SOM matrices	2,5-Dihydroxyacetophenone	2,5-DHAP	9.86[b]	N/A	Commercially purchased	Dual-Polarity	Proteins and oligonucleotide	56
						Positive	Peptides, proteins, and glycoproteins	57
						Positive	Primary amines	58
						Negative	phosphatidylethanolamine	59
						Dual-Polarity	Lipid species	60
SOM matrices	2-Mercaptobenzothiazole	2-MBT	7.69[a]	N/A	Commercially purchased	Dual-Polarity	Peptides and proteins	61

Matrix class	Matrix names	Acronyms	pKa*	GB(M) (kJ·mol^{-1})*	Matrix source	Ionization mode	Analytes	Ref.
Reactive matrices	2,4-Dinitrophenylhydrazine	DNPH	1.55[a]	N/A	Commercially purchased	Dual-Polarity	Lipids on brain and liver tissue	62
						Positive	Flavonoids	63
						Positive	Alkaloids and flavonoid	64
						Positive	Lipid in human colon	65
						Positive	Small molecules	66
						Positive	4-hydroxy-2-nonenal-modified peptides	67
						Positive	Oxidized phospholipids	68
						Positive	Gaseous aldehydes	69
						Positive	Carbonyl containing compounds	70
SOM matrices	9H-pyrido[3,4-b]indole	Nor-harmane	14.5[a]	N/A	Commercially purchased	Dual-Polarity	Proteins and sulfated oligosaccharides	71
						Dual-Polarity	Native high-methoxylated pectin	72
						Dual-Polarity	Oligosaccharides	73
						Dual-Polarity	Bacterial lipid A (endotoxin)	74
						Dual-Polarity	Sulfated neocarrabiose oligosaccharides	75

*pKa: the pKa value of the matrix in solution; GB(M): gas-phase basicity for matrix molecules, a thermochemical parameter commonly used to describe proton transfer reactions. N/A: Information not available. [a]Measured values, from references 76, 77, 78, 79, 80, 81, 82, 83, 84, 85, 86, 87, 88, and 89; [b]Calculated values, from references 90, 91, and 38.

Supporting Information Table S2. Properties and applications of commonly used inorganic matrices in MALDI-MS.

Matrix class	Matrix names	Acronyms	Size or structure*	Matrix source	Ionization mode	Analytes	Ref.
Metallic nanostructures	Gold nanoparticles	AuNPs	Dia.: 2, 5 and 10 nm	Commercially purchased	Dual-Polarity	Peptides and proteins	92
			Dia.: 12 ± 1.9 nm	Synthetically prepared	Positive	Small neutral carbohydrates	93
			Dia.: ~3 and ~10 nm	Commercially purchased	Positive	Endogenous and exogenous compounds	94
			Dia.: 3.5 nm	Synthetically prepared	Positive	Aminothiols	95
			Dia.: 12.2 ± 1.4 nm	Synthetically prepared	Positive	Small metabolites	96
Metallic nanostructures	Silver nanoparticles	AgNPs	Dia.: 34 ± 3 nm	Synthetically prepared	Negative	Estrogens	97
			Dia.: 0.5-15 nm	Synthetically prepared	Dual-Polarity	Glycerophospholipids	98
			Dia.: 0.5-16 nm	Commercially purchased	Dual-Polarity	Neutral lipids, such as cerebrosides	99
Metallic nanostructures	Titanium dioxide nanoparticles	TiO_2 NPs	Dia.: 1 μm	Commercially purchased	Positive	PEG 200 and methyl stearate	100
			Dia.: < 10 nm	Synthetically prepared	Positive	LMW metabolites	101
			Dia.: < 50 nm	Commercially purchased	Positive	Lipids	102
Metallic nanostructures	Magnetic iron oxide nanoparticles	Fe_3O_4 NPs	Dia.: 25.49-49.51 nm	Commercially purchased	Positive	Asphaltene molecules	103
Carbon-based Nanomaterials	Carbon nanotubes	CNTs	OD/ID: ~20/2 nm	Synthetically prepared	Positive	Peptides, organic compounds, and β-cyclodextrin	104
			N/A	Commercially purchased	Positive	Neutral small carbohydrates	105
			Dia.: ~250 nm × L: ~60 μm	Synthetically prepared	Positive	Peptides and proteins	106
Carbon-based Nanomaterials	Oxidized carbon nanotubes	Oxidized CNTs	OD/ID: ~10/3-5 nm × L: >several µm	Synthetically prepared	Positive	Low-mass compounds in environmental samples	107
			N/A	Synthetically prepared	Positive	Small molecules	108
Carbon-based Nanomaterials	Graphene	G	N/A	Synthetically prepared	Positive	Small molecules	109

Matrix class	Matrix names	Acronyms	Size or structure*	Matrix source	Ionization mode	Analytes	Ref.
			Thickness: ~8-10 nm × Lateral size: ~600 nm	Commercially purchased	Negative	Small molecules	110
			Lane width: ~250 µm	Commercially purchased	Dual-Polarity	Endogenous caffeine and theanine	111
			N/A	Commercially purchased	Dual-Polarity	Flavonoids	112
Carbon-based Nanomaterials	Graphene oxide	GO	wrinkled single-layer graphene structure	Synthetically prepared	Positive	Disaccharides	113
			Thickness: 0.8-1.2 nm	Commercially purchased	Negative	Small molecules	114
			N/A	Commercially purchased	Positive	Small molecular components	115
Silicon-based nanostructures	Porous silicon	pSi, DIOS	Micropores: <2 nm; mesopores: 2-50 nm	Synthetically prepared	Positive	Peptides, small molecules, and WIN antiviral drug	116
			N/A	Synthetically prepared	Positive	Small molecules	117
			Sponge-like, high porosity: ~600 $m^2 \cdot cm^{-3}$;	Synthetically prepared	Positive	Secondary metabolites	118
			N/A	Synthetically prepared	Positive	Protein characterization	119
			N/A	Synthetically prepared	Positive	Low-mass components of polyesters	120
Silicon-based nanostructures	Nanostructure-initiator mass spectrometry	NIMS	Nanostructured surface, pore size: ~10 nm	Synthetically prepared	Positive	Peptides and metabolites	121
			N/A	Synthetically prepared	Positive	Xenobiotics and endogenous metabolites	122
			Surface pore size: ~10-20 nm	Synthetically prepared	Negative	Phosphorylated metabolites	123

*N/A: information not available; Dia.: diameter; OD: outer diameter; ID: inner diameter; L: length.

Supporting Information Table S3. Small organic molecule (SOM) matrices with their preferred ionization mode and applications for MALDI-MS/MSI.

Matrix class	Matrix names	Acronyms	Chemical Formula	Monoisotopic MW (Da)*	Average MW (Da)	Ionization mode	Targets	Ref.
Cinnamic acid based matrices	Sinapinic acid	SA	$C_{11}H_{12}O_5$	224.068473	224.21	Positive	Monoclonal human immunoglobulin IgM	6
						Positive	Immune complexes	7
						Positive	Short peptides	8
						Positive	Carbohydrate	9
	Ferulic acid	FA	$C_{10}H_{10}O_4$	194.057909	194.18	Negative	Fluorophore-labeled oligonucleotides	124
						Positive	Proteins	125
						Positive	Cyanocobalamin and peptides	126
	Caffeic acid	CA	$C_9H_8O_4$	180.042259	180.16	Positive	Proteins	127 - 129
	α-Cyano-4-hydroxycinnamic acid	CHCA	$C_{10}H_7NO_3$	189.042593	189.17	Positive	Peptides and proteins	11
						Positive	Thymosin β4	12
	3,4-Dimethoxycinnamic acid	DMCA	$C_{11}H_{12}O_4$	208.073559	208.21	Positive	Low molecular weight (LMW) compounds	130
	Curcumin		$C_{21}H_{20}O_6$	368.125988	368.38	Positive	Drugs, lipids, and fingermarks	131
Acetophenone based matrices	2,4-Dihydroxyacetophenone	2,4-DHAP	$C_8H_8O_3$	152.047344	152.15	Negative	SiRNA oligonucleotide	132
	2,5-Dihydroxyacetophenone	2,5-DHAP	$C_8H_8O_3$	152.047344	152.15	Dual-Polarity	Proteins and oligonucleotide	56
						Positive	Peptides, proteins, and glycoproteins	57
						Positive	Primary amines	58
	2,6-Dihydroxyacetophenone	2,6-DHAP	$C_8H_8O_3$	152.047344	152.15	Positive	Hydrophobic proteins and peptides	133
						Positive	Fragile peptides, disulphide bonding, and small proteins	134
	2,4,6-Trihydroxyacetophenone	THAP	$C_8H_{10}O_5$	186.052823	186.16	Dual-Polarity	Noncovalent RNA-peptide complexes	22
						Positive	Lipids	135
						Negative	Natural and modified oligonucleotides	23
						Positive	Polysaccharides	24
Thiazole based matrices	2-Mercaptobenzothiazole	2-MBT	$C_7H_5NS_2$	166.986341	167.25	Dual-Polarity	Peptides and proteins	136
						Positive	Lipids	62

Matrix class	Matrix names	Acronyms	Chemical Formula	Monoisotopic MW (Da)*	Average MW (Da)	Ionization mode	Targets	Ref.
	5-Chloro-2-mercaptobenzothiazole	CMBT	$C_7H_4ClNS_2$	166.986341	201.70	Positive	Peptides, LMW proteins, oligosaccharides, and glycolipids	136
						Dual-Polarity	Peptidoglycan muropeptides	61
						Dual-Polarity	Phospholipids	137
	5-Ethyl-2-mercaptothiazole	EMT	$C_5H_7NS_2$	145.001991	145.30	Dual-Polarity	Substance P, insulin, beta-cyclodextrin, triacylglycerols of coconut oil, and polypropylene glycol 2000	138
	5-Amino-2-mercapto-1,3,4-thiadiazole	AMT	$C_2H_3N_3S_2$	132.976839	133.20	Positive	Neutral carbohydrates	139
	3-[5'-(Methylthio)-2,2'-bithiophen-5-ylthio]propanenitrile	MT3P	$C_{12}H_{11}NS_4$	296.977433	297.50	Positive	Alkaloids	140
	2,3,4,5-Tetrakis(3',4'-dihydroxylphenyl)thiophene	DHPT	$C_{28}H_{20}O_8S$	516.087889	516.5	Positive	LMW amines and creatinine	141
	2-[5-(2,4-Dichlorobenzoyl)-2-thienyl]acetic acid	DCBTA	$C_{13}H_8Cl_2O_3S$	313.95712	315.17	Positive	Peptides, peptide mixtures, α-cyclodextrin, testosterone, and caffeine	142
Benzoic acid based matrices	2,5-Dihydroxybenzoic acid	DHB	$C_7H_6O_4$	154.026609	154.12	Positive	Proteins	1
						Dual-Polarity	Lipidomics	2
	3-Hydroxy-4-nitrobenzoic acid	3H4NBA	$C_7H_5NO_5$	183.016772	183.12	Positive	2-nitrobenzenesulfenyl-labeled peptides	143
	3-Hydroxy-2-nitrobenzoic Acid	3H2NBA	$C_7H_5NO_5$	183.016772	183.12	Positive	Peptides	144
	2,4-Dihydroxy-5-nitrobenzoic acid	DHNBA	$C_7H_5NO_6$	199.011687	199.12	Positive	Phytohormones	145
	Mefenamic acid	MA	$C_{15}H_{15}NO_2$	241.110279	241.29	Positive	Polypeptides, proteins, and drugs	146
	4-Mercaptobenzoic acid	MBA	$C_7H_6O_2S$	154.00885	154.19	Positive	Metals	147
						Positive	Cesium and strontium	148
	3-Amino-4-hydroxybenzoic	3,4-AHB	$C_7H_7NO_3$	153.042593	153.14	Positive	Underivatised oligosaccharides	149

Matrix class	Matrix names	Acronyms	Chemical Formula	Monoisotopic MW (Da)*	Average MW (Da)	Ionization mode	Targets	Ref.
	acid					Positive	Glycotyping of *O*-Linked glycopeptides and intact mucins	150
	3-Methoxy-4-hydroxybenzoic acid	3M4HBA	$C_8H_8O_4$	168.042259	168.15	Positive	Proteins	151
	5-Aminosalicylic acid	5-ASA	$C_7H_7NO_3$	153.042593	153.04	Positive	Peptides	152
	5-Methoxysalicylic acid	5-MSA	$C_8H_8O_4$	168.042259	168.15	Negative	Oligonucleotides	153
	2-(4-Hydroxyphenylazo) benzoic acid	HABA	$C_{13}H_{10}N_2O_3$	242.069142	242.23	Positive	Peptides, proteins, and glycoproteins	154
	2,5-Dihydroxyterephthalic acid	DHT	$C_8H_6O_6$	198.016438	198.13	Positive	Amino acids	155
Anthracene based matrices	Dithranol	DT	$C_{14}H_{10}O_3$	226.062994	226.23	Positive	Lipids	36
	1,8,9-Trihydroxyanthracene	DIT	$C_{14}H_{10}O_3$	226.062994	226.23	Dual-Polarity	Lipids	30
	9-Nitroanthracene	9-NA	$C_{14}H_9NO_2$	223.063329	223.23	Negative	Fluorinated fullerenes	156
						Negative	Derivatized fullerenes	157
Flavonoid based matrices	Isoliquiritigenin	ISL	$C_{15}H_{12}O_4$	256.073559	256.25	Positive	Neutral oligosaccharides	158
	Quercetin		$C_{15}H_{10}O_7$	302.042653	302.24	Positive	Pt(II) and Pd(II) complexes	159
						Positive	Endogenous lipids	37
						Dual-Polarity	Porcine adrenal gland lipids	160
	Rutin		$C_{27}H_{30}O_{16}$	610.153385	610.52	Positive	Pt(II) and Pd(II) complexes	159
	Apigenin		$C_{15}H_{10}O_5$	270.052823	270.24	Positive	Transition metal complexes	161
	Kaempferol		$C_{15}H_{10}O_6$	286.047738	286.24	Positive		
	Luteolin		$C_{15}H_{10}O_6$	286.047738	286.24	Positive	Endogenous lipids	37
						Positive	Transition metal complexes	161
	3-Hydroxyflavone	3-HF	$C_{15}H_{10}O_3$	238.062994	238.24	Positive	Endogenous lipids	37
	5-Hydroxyflavone	5-HF	$C_{15}H_{10}O_3$	238.062994	238.24	Positive		
	3,7-Dihydroxyflavone	3,7-DHF	$C_{15}H_{10}O_4$	254.057909	254.24	Positive		
	Chrysin		$C_{15}H_{10}O_4$	254.057909	254.24	Positive		
	7,3',4'-Trihydroxyflavone	7,3',4'-THF	$C_{15}H_{10}O_5$	270.052823	270.24	Positive		
	Fisetin		$C_{15}H_{10}O_6$	286.047738	286.24	Positive		
	Morin		$C_{15}H_{12}O_8$	320.053217	320.25	Positive		

Matrix class	Matrix names	Acronyms	Chemical Formula	Monoisotopic MW (Da)*	Average MW (Da)	Ionization mode	Targets	Ref.
Coumarin based matrices	7-Mercapto-4-methylcoumarin	MMA	$C_{10}H_8O_2S$	192.0245	192.23	Positive	LMW compounds and alkaloids	162
	Aquatic fulvic acid	AFA	$C_{14}H_{12}O_8$	308.053217	308.24	Positive	Carbohydrates, cyclodextrins, and peptides	163
	Usnic acid	UA	$C_{18}H_{16}O_7$	344.089603	344.32	Positive	Small molecules	164
	3-Hydroxycoumarin	3-HC	$C_9H_6O_3$	162.031694	162.14	Dual-Polarity	DNA	165
	Esculetin		$C_9H_6O_4$	178.026609	178.14	Positive<br>Positive	Olanzapine	166
	Coumarin		$C_9H_6O_2$	146.036779	146.14	Positive		
	Umbelliferone		$C_9H_6O_3$	162.031694	162.14	Positive		
	7-Hydroxycoumarin-3-carboxylic acid	HCA	$C_{10}H_6O_5$	206.021523	206.15	Positive	Hydrophobic compounds	167
	6,7-Dihydroxycoumarin-3-carboxylic acid	DCA	$C_{10}H_6O_6$	222.016438	222.15	Positive		
Pyridine based matrices	3-Hydroxypicolinic acid	3-HPA	$C_6H_5NO_3$	139.026943	139.11	Dual-Polarity	Underivatized single-stranded DNA oligomers	32
						Dual-Polarity	Oligonucleotides	33
						Negative		5
	2-Picolinic acid	2-PA	$C_6H_5NO_2$	123.032028	123.11	Negative	Nucleic acids and proteins	168
	Nicotinic acid	NA	$C_6H_5NO_2$	123.032028	123.11	Positive	Proteins	169
	Hydrazinonicotinic acid	HYNIC	$C_6H_7N_3O_2$	153.053826	153.14	Positive	Oligosaccharides	170
	2-(2-Aminoethyloamino)-5-nitropyridine	AAN	$C_7H_{10}N_4O_2$	N/A	182.18	Dual-Polarity	Phospholipids	171
	Nifedipine		$C_{17}H_{18}N_2O_6$	346.116486	346.33	Negative	Polyphenols	172
	2-Amino-4-methyl-5-nitropyridine	AMNP	$C_6H_7N_3O_2$	153.053826	153.14	Dual-Polarity	Proteins and oligonucleotides	173
	2-Amino-5-nitropyridine	ANP	$C_5H_5N_3O_2$	139.038176	139.11	Negative	Oligonucleotides	174
	2-Hydroxy-5-nitro-3-(trifluoromethyl)pyridine	HNTP	$C_6H_3F_3N_2O_3$	208.009577	208.09	Positive	Metabolites	175
Benzophenone based matrices	3,4-Diaminobenzophenone	DABP	$C_{13}H_{12}N_2O$	212.094963	212.25	Positive	Peptide and protein samples	176
						Negative	Oligonucleotides	177
	Michler's ethyl ketone	MEK	$C_{21}H_{28}N_2O$	324.220164	324.46	Negative	Lipids	178

Matrix class	Matrix names	Acronyms	Chemical Formula	Monoisotopic MW (Da)*	Average MW (Da)	Ionization mode	Targets	Ref.
Nitrobenzene based matrices	4-Hydroxy-3-nitrobenzonitrile	HNBN	$C_7H_4N_2O_3$	164.022192	164.12	Positive	Small organic, peptide, and protein molecules	179
	p-Nitroaniline	PNA	$C_6H_6N_2O_2$	138.042927	138.12	Positive	Taxus breyifolia extracts	180
						Dual-Polarity	Lipids and phytohormones	181
	2-Amino-3-nitrophenol	ANP	$C_6H_6N_2O_3$	154.037842	154.12	Dual-Polarity	Peptides, small proteins, and polymers	182
	4-Nitrocatechol	4-NC	$C_6H_5NO_4$	155.021858	155.11	Positive	LMW compounds	183
	2-Nitrophloroglucinol	2-NPG	$C_6H_5NO_5$	171.016772	171.11	Positive	Fungicide pyrimethanil	184
Naphthalene based matrices	1,8-Bis(dimethylamino) naphthalene	DMAN	$C_{14}H_{18}N_2$	214.146999	214.31	Negative	Fatty acids	185
	1,8-Bis(tetramethylguanidino)-naphthalene	TMGN	$C_{20}H_{30}N_6$	354.253195	354.49	Negative	Trace perfluorinated compounds in environmental water samples	186
	N-phenyl-2-naphthylamine	P2NA	$C_{16}H_{13}N$	219.104799	219.28	Negative	Small molecules	187
	1,14-Diaza[5]helicene		$C_{20}H_{12}N_2$	280.100048	280.32	Negative	Acidic analytes	188
	Maleic anhydride proton sponge	MAPS	$C_{18}H_{20}N_2O_3$	N/A	N/A	Negative	Small molecules	189
	1,8-Bis(trispyrrolidinophosphazenyl)naphthalene	TPPN	$C_{36}H_{57}N_9P_2$	677.421216	677.80	Negative	Cholesterol	190
	N-(1-naphthyl) ethylenediamine dihydrochloride	NEDC	$C_{12}H_{16}Cl_2N_2$	258.069054	259.17	Negative	Glucose	191
						Negative	Metal ions	192
	N-(1-naphthyl) ethylenediamine dinitrate	NEDN	$C_{12}H_{12}N_4O_6$	308.075684	308.25	Negative	Small molecules	193
	1-Naphthylhydrazine hydrochloride	NHHC	$C_{10}H_{11}ClN_2$	194.061076	194.66	Negative	Glucose and homogentisic acid	194
	1,4-Dihydroxy-2-naphthoic acid	DHNA	$C_{11}H_8O_4$	204.042259	204.18	Positive	Polymer	195
						Positive	Lipids	196
Pyridazine based matrices	3-Aminophthalhydrazide	3-APH	$C_8H_7N_3O_2$	177.053826	177.16	Dual-Polarity	Metabolites	197
	Hydralazine	HZN	$C_8H_8N_4$	160.074896	160.18	Dual-Polarity	Small molecular, lipids, and proteins	198

Matrix class	Matrix names	Acronyms	Chemical Formula	Monoisotopic MW (Da)*	Average MW (Da)	Ionization mode	Targets	Ref.
	4-Hydrazinoquinazoline	4-HQ	$C_8H_8N_4$	160.074896	160.18	Dual-Polarity	Neutral and sialylated glycans	199
	4-Aminocinnoline-3-carboxamide	4-AC	$C_9H_8N_4O$	188.069811	188.19	Dual-Polarity	Metabolites	200
Indole based matrices	Indole-3 pyruvic acid	IPA	$C_{11}H_9NO_3$	203.058243	203.19	Dual-Polarity	Proteins and peptides	201
	Trans-3-indoleacrylic acid	IAA	$C_{11}H_9NO_2$	187.063329	187.19	Dual-Polarity	Anabolic steroids	202
	9H-pyrido[3,4-b]indole	Nor-harmane	$C_{11}H_8N_2$	168.068748	168.19	Dual-Polarity	Proteins and sulfated oligosaccharides	71
						Dual-Polarity	Native high-methoxylated pectin	72
						Dual-Polarity	Oligosaccharides	73
						Dual-Polarity	Bacterial lipid A (endotoxin)	74
	1-Methyl-9H-pyrido[3,4-b]indole	Harmane	$C_{12}H_{10}N_2$	182.084398	182.22	Dual-Polarity	Proteins and sulfated oligosaccharides	71
	7-Methoxy-1-methyl-9H-pyrido[3,4-b]indole	Harmine	$C_{13}H_{12}N_2O$	212.094963	212.25	Negative	Anionic adducts of oligosaccharides	79
						Dual-Polarity		
	1-Methyl-9H-pyrido[3,4-b]indol-7-ol	Harmol	$C_{12}H_{10}N_2O$	198.079313	198.22	Dual-Polarity	Proteins and sulfated oligosaccharides	71
	3,4-Dihydro-7-methoxy-1-methyl-9H-pyrido[3,4-b]indole	Harmaline	$C_{13}H_{14}N_2O$	214.110613	214.26	Dual-Polarity		
	3,4-Dihydro-1-methyl-9H-pyrido[3,4-b]indol-7-ol	Harmalol	$C_{12}H_{10}N_2O$	198.079313	198.22	Dual-Polarity		
	Pyridoindoles		N/A	N/A	N/A	Dual-Polarity	Proteins, carbohydrates, and synthetic polymers	203
	Pyridylindoles		N/A	N/A	N/A	Dual-Polarity		
	Pyridylpyridoindoles		N/A	N/A	N/A	Dual-Polarity		
	3-Methyl-4-nitro-1H-indole	3,4-MNI	$C_9H_8N_2O_2$	176.058578	176.17	Dual-Polarity	Lipid, peptide, protein, glycan, and perfluorooctanesulfonic acid compounds	204
	2,3-Dimethyl-6-nitro-1H-indole	2,3,6-DMNI	$C_{10}H_{10}N_2O_2$	190.074228	190.20	Dual-Polarity		
	3-Methyl-6-nitro-1H-indole	3,6-MNI	$C_8H_7N_3O_2$	177.053826	177.16	Dual-Polarity		
	2,3-Dimethyl-4-nitro-1H-indole	2,3,4-DMNI	$C_{10}H_{10}N_2O_2$	190.074228	190.20	Dual-Polarity		
	4-Nitro-1H-indole	4-NI	$C_8H_6N_2O_2$	162.042927	162.15	Negative		

Matrix class	Matrix names	Acronyms	Chemical Formula	Monoisotopic MW (Da)*	Average MW (Da)	Ionization mode	Targets	Ref.
Others	4-Hydroxy-3-methoxyphenylpyruvic acid	HMPPA	$C_{10}H_{10}O_5$	210.052823	210.18	Positive	Peptides and proteins	201
	D-arabinosazone	DAZ	$C_{17}H_{20}N_4O_3$	328.153541	328.37	Positive	Carbohydrates	205
	2-Amino-4,5-diphenylfuran-3-carboxylic acid	ADFA	$C_{17}H_{13}NO_3$	279.089543	279.29	Positive	Polypeptides, proteins, and drugs	146
	2,3-Dicyanohydroquinone	DCH	$C_8H_4N_2O_2$	160.027277	160.13	Positive	Lipids	206
	5-Amino-1-naphthol	5,1-ANL	$C_{10}H_9NO$	159.068414	159.18	Positive	Phosphorylated peptides	207
	1H-pteridine-2,4-dione	Lumazine	$C_6H_4N_4O_2$	164.033425		Positive	Complex (phospho) lipid mixtures	208
	Olanzapine	OLZ	$C_{17}H_{20}N_4S$	312.140868	312.43	Positive	Peptides	209
	1,4-Dioxo-1,2,3,4-tetrahydrophthalazine-6-carboxylic acid	DTCA	$C_9H_6N_2O_4$	206.032757	206.15	Negative	LMW compounds	210
	9-Aminoacridine	9-AA	$C_{13}H_{10}N_2$	194.084398	194.23	Negative	LMW compounds	16
	1,6-Diphenyl-1,3,5-hexatriene	DPH	$C_{18}H_{16}$	232.125201	232.32	Negative	Lipids	211
	6-Thioguanine	6-TG	$C_5H_5N_5S$	167.026566	167.19	Negative	Oligonucleotides	212
	N1,N4-dibenzylidenebenzene-1,4-diamine	DBDA	$C_{20}H_{16}N_2$	284.131349	284.35	Negative	Small molecules and fatty acids	213
	4-Aminoazobenzene	AAB	$C_{12}H_{11}N_3$	197.095297	197.24	Negative	Metabolites	214
	6-Aza-2-thiothymine	ATT	$C_4H_5N_3OS$	143.015333	143.17	Negative	Oligonucleotides	41
						Positive	Intact double-stranded DNA	42
						Positive	Specific noncovalent complexes between guanidinium derivatives and single-stranded DNA	43
	Quinaldic acid	QA	$C_{10}H_7NO_2$	173.047678	173.17	Dual-Polarity	Nucleic acids	215
	Lignin		N/A	N/A	N/A	Dual-Polarity	Small molecules	216
	1,1'-Binaphthyl-2,2'-diamine	BNDM	$C_{20}H_{16}N_2$	284.131349	284.35	Dual-Polarity	Metabolites	217
	Dansylcadaverine	DC	$C_{17}H_{25}N_3O_2S$	335.166748	335.46	Dual-Polarity	Proteomics	218
	Dansylhydrazine	DH	$C_{12}H_{15}N_3O_2S$	265.088498	265.33	Dual-Polarity		
	Dansyl-DL-a-amino caprylic acid	DDCA	$C_{26}H_{41}N_3O_4S$	491.281778	491.69	Dual-Polarity		

Matrix class	Matrix names	Acronyms	Chemical Formula	Monoisotopic MW (Da)*	Average MW (Da)	Ionization mode	Targets	Ref.
	11-(Dansylamino)undecanoic acid	DUA	$C_{23}H_{34}N_2O_4S$	434.223929	434.59	Dual-Polarity		
	Basic Blue 7	BB7	$C_{33}H_{40}ClN_3$	513.291076	514.15	Dual-Polarity	Lipids	219

*MW: molecule weight; N/A: information not available.

Supporting Information Table S4. Designed and synthesized organic matrices with their preferred ionization mode and applications for MALDI-MS/MSI.

Matrix class	Sub class	Matrix names	Acronyms	Ionization mode	Targets	Ref.
Reactive matrices	Schiff base synthesis reaction	Anthranilic acid	AA	Negative	Oligodeoxynucleotides	220
		Aminopyrazine	AP	Positive	Oligosaccharides	84
		3-Aminoquinoline	3-AQ	Dual-Polarity	Oligosaccharides	46
		2-Phenyl-3-(p-aminophenyl) acrylonitrile	PAPAN	Positive	Glycans	221
		9-(3,4-Diaminophenyl)acridine	DAA	Positive	α-Dicarbonyl compounds	222
		α-Cyano-3-aminocinnamic acid	3-CACA	Positive	Plant *N*-glycans	223
		2,4-Dihydroxybenzaldehyde	2,4-DHBA	Positive	Primary amines	58
		Tryptamine		Positive	Non-polar carbonyl compounds	224
	Wolff-Kishner reduction reaction	2,4-Dinitrophenylhydrazine	DNPH	Positive	Small molecules	66
				Positive	4-Hydroxy-2-nonenal-modified peptides	67
				Positive	Oxidized phospholipids	68
				Positive	Gaseous aldehydes	69
		4-Dimethylamino-6-(4-methoxy-1-naphthyl)-1,3,5-triazine-2-hydrazine	DMNTH	Positive	Small carbonyl compounds	163
				Positive	Carbonyl containing compounds	70
		2-Nitro-4-carboxyphenylhydrazine	NCPH	Dual-Polarity	*N*-glycan	225
		2,4-Dicarboxylphenylhydrazine	DCPH	Dual-Polarity		
		2-Hydroxybenzohydrazide	HBH	Positive	Gaseous aldehydes	69
		3-Hydroxy-2-naphthoic acid hydrazide	3-HNAH	Positive	Gaseous aldehydes	69
				Positive	Steroids with carbonyl groups	226
		2-Hydrazinoquinoline	2-HQ	Positive	Gaseous carbonyl compounds	227
				Positive	Gaseous aldehydes	69
		2-Hydrazinopyrimidine	2-HPM	Positive	Cholesterol and fatty alcohols	228
				Positive	Oligosaccharides	229
		2-Hydrazinoterephthalic acid	2-HTA	Negative	*N*-glycans	230
	Condensation	2,4-Diphenyl-pyranylium	DPP	Positive	Biologically active primary	231

Matrix class	Sub class	Matrix names	Acronyms	Ionization mode	Targets	Ref.
	reaction	1,4-Phenylene-4,4'-bis（2,6-diphenyl-4-pyrylium）	PBDPP	Positive	amines (catecholamine neurotransmitters)	
		2,4,6-Trimethylpyrylium	TMP	Positive		
		2-Fluoro-1-methyl pyridinium	FMP	Positive	Phenolic hydroxyl and/or primary or secondary amine groups	232
		1-Pyrenylboronic acid	1-PBA	Positive	Polyfunctional compounds	233
				Positive	Ubiquitous cis-1,2-diol functionality	234
		4-(N-methyl)pyridinium boronic acid	4-(N-Me)$Py^{+}B(OH)_2$	Positive	Catecholamines	235
		[(E)-4-(2-cyano-2-carboxyvinyl) phenyl]boronic acid	CCPBA	Negative	LMW compounds (such as 1,2 and 1,3-diols, a-hydroxyacids and aminols or, even, inorganic anions as fluoride)	236
		6-Borono-1-methylquinoline-1-ium	BMQI	Positive	*cis*-diol compounds	237
		p-Methoxy cinnamaldehyde	PMC	Positive	primary amines, amino acids and neurotransmitters	238
		4-Trimethylamino-6-(4-methoxy-1-naphthyl)-1,3,5-triazine-2-(3-aminophenylboronic acid	TMNTA	Positive	glucose and fructose isomers	239
	Addition reaction	Benzophenone	BPh	Positive	Unsaturated phospholipids（PLs）	240
		2-Benzoylpyridine	BzPy	Dual-Polarity	Lipids	241
		Thiosalicylic acid	TSA	Positive	The disulfide bonds in peptide	242
	Electron transfer reaction	2-[(2E)-3-(4-tert-Butylphenyl)-2-methylprop-2-enylidene] malononitrile	DCTB	Dual-Polarity	Coordination compounds, organometallics, and conjugated organic compounds	51
				Positive	Polythiophene	52
				Negative	Very labile compounds	53

Matrix class	Sub class	Matrix names	Acronyms	Ionization mode	Targets	Ref.
		9,10-Diphenylanthracene	9,10-DPA	Dual-Polarity	9-Methylanthracene, fluoranthene, *trans*-stilbene, pyrene, chlorophyll (a), vitamin A, vitamin K1 (phylloquinone), vitamin K2 (menaquinone) and 4-chromanol	243
		Poly(3-octylthiophene-2,5-diyl	P3OT	Negative	1,2-Dioxetanes	244
		7,7,8,8-Tetracyanoquinodimethane	TCNQ	Negative	Insoluble giant polycyclic aromatic hydrocarbons	245
		Tetrathiafulvalene	TTF	Negative	Industrial pigments	246
		1,5-Diaminonaphthalene	DAN	Positive	Proteins	27
				Positive	Chlorophyll identification	247
				Dual-Polarity	Bacteriochlorophyll a in rhodobacter sphaeroides and its zinc and copper analogue pigments	248
		Phenylenevinylene	PV	Positive	Porphyrins, polyaromatics and Pphthalocyanine	249
		1-Amino-2,4-dichloronaphthalene	ADCN	Negative	Metabolomics	250
		Anthracene		Positive	LMW non-polar polymers	251
		Pyrene		Positive		
		Acenaphthene		Positive		
		Terthiophene		Positive	Metallocenes and a methylene-bridged bisphenol	252
		Anthracene-*d10*		Positive		
	Special chemical reaction	Glycosyl-3-aminoquinoline	Gly-3AQ	Positive	PH change	253
		Nicotinic acid chlorides	NAC	Positive	Alcohols	224
		Quinoline-6-carboxylic acid chlorides	Quinoline-6CAC	Positive		
		(E)-2-cyano-N-(2-(2,5-dioxo-2,5-dihydro-1H-pyrrol-1-yl)ethyl)-3-(4-hydroxyphenyl)acrylamide	CHC-Mal	Positive	Free thiol-groups in metabolites and proteins	254
Matrix derivatization	Cinnamic acid derivatives	α-Cyano-4-hydroxycinnamic methyl ester	CHCE	Positive	Peptides	255
		(E)-α-cyano-4-hydroxycinnamic acid propyl ester	CHCA-C3	Positive	Proteins	256

Matrix class	Sub class	Matrix names	Acronyms	Ionization mode	Targets	Ref.
		4-Chloro-α-cyanocinnamic acid	ClCCA	Positive	Bovine serum albumin	257
				Dual-Polarity	Phosphatidylethanolamine chloramines	258
				Dual-Polarity	Sialylated glycans and glycopeptides	259
				Positive	Cyanocobalamin	260
				Positive	Cyanocobalamin conjugates of cisplatin and diaminocyclohexane-platinum(II)	261
		(2E)-3-(anthracen-9-yl)-2-cyanoprop-2enoic acid	AnCCA	Positive	LMWcompounds	262
		(E)-2-cyano-3-(naphthalen-2-yl)acrylic acid	CNAA	Positive		
				Dual-Polarity	Lipids	263
		(2E,4E)-2-cyano-5-(4-nitrophenyl)penta-2,4-dienoic acid	CNDA	Dual-Polarity		
		α-Cyano-2,4-difluorocinnamic acid	Di-FCCA	Positive	Lipids	264
		α-Cyano-5-phenyl-2,4-pentadienic Acid	CPPA	Positive	Intact proteins	265
		D^4-α-cyano-4-hydroxycinnamic acid	D^4-CHCA	Positive	synthetic small molecule pharmaceutical, isoquinoline alkaloid, and endogenous compounds	266
		4-Phenyl-α-cyanocinnamic acid amide	Ph-CCA-NH_2	Negative	Multiple lipid classes	267
		p-Phenyl-α-cyanocinnamic acid amide	p-Ph-CCAA	Negative	Sulfatide	268
		4-Aminocinnamic acid	ACA	Dual-Polarity	Lipids	269
		4-(Dimethylamino)cinnamic acid	DMACA	Dual-Polarity		
	Benzoic acid derivatives	Lithium 2,5-dihydroxybenzoate	LiDHB	Positive	Lipids and HMW hydrocarbons	270
		Alkylated trihydroxyacetophenone	ATHAP	Positive	Hydrophobic peptides	271
		COOH-NHMe (IV)		Dual-Polarity	Lipids and proteins	272
	Others	Lithium salicylic acid	LiSalA	Positive	Hydrocarbons and wax esters	273
		Lithium vanillate	LiVA	Positive		
		Lithium sinapate	LiSA	Positive		

Matrix class	Sub class	Matrix names	Acronyms	Ionization mode	Targets	Ref.
		1-AP-derived group of uniform materials based on organic salts	1-AP-based GUMBOS	Positive	Hydrophobic peptides	274
		1,5-Diaminonaphthalene hydrochloride	DAN hydrochloride	Negative	Small molecules	275
		N,N'-bis(4-hydroxylsalicylidene)-p-phenylenediamine	BSPD-OH	Positive	LMW compounds	276
		N,N '-bis(4-methoxysalicylidene)p-phenylenediamine	BSPD-OMe	Positive		
		N,N'-bis(salicylidene)p-phenylenediamine	BSPD	Positive		
		1,8-Di(piperidinyl)naphthalene	DPN	Negative	Metabolomics	277
		5-(3-Trifluoromethylbenzvldene)thiazoldine-2.4-dione	3-CF_3-BTD	Positive	Biogenic monoamine transmitters	278
		(E)-4-(2,5-dihydroxyphenyl)but-3-en-2-one	2,5-cDHA	Positive	Proteins and LMW analytes	279
		4,5-Dimethoxy-2-nitrobenzyl-2,5-dihydroxyacetophenone	DMNB-2,5-DHAP	Dual-Polarity	Lipids	280
		Functional melanin nanoparticles	COOH-MNP-COOH	Negative	Tetrabromobisphenol A and tetrabromobisphenol S	281
Ionic liquid matrices	Ionic liquids based on DHB	2,5-Dihydroxybenzoic acid/pyridine	DHB/Pyr	Dual-Polarity	LMW compounds	282
				Dual-Polarity	Lipids	283
				Positive	Pathogenic bacteria	284
		2,5-Dihydroxy benzoic acid butylamine	DHBB	Positive	Pullulans	285
				Positive	Glycoconjugates	286
		2,5-Dihydroxybenzoic acid/aniline	DHB/ANI	Dual-Polarity	Lipids	283
				Positive	Pathogenic bacteria	284
		2,5-Dihydroxybenzoic acid/*N,N*-diethylamine	DHB/DEA	Positive	Pathogenic bacteria	284
		2,5-Dihydroxybenzoic acid/3-acetylpyridine	DHB/3-AP	Dual-Polarity	Lipids	283
		2,5-Dihydroxybenzoic acid/*N,N*-dimethylaniline	DHB/DMA	Positive	Pathogenic bacteria	284
				Positive	Oligosaccharides	287

Matrix class	Sub class	Matrix names	Acronyms	Ionization mode	Targets	Ref.
		2,5-Dihydroxybenzoic acid/*N*-methylaniline	DHB/ *N*-MA	Positive	Carbohydrates	288
		2,5-Dihydroxybenzoic acid/*N*-ethylaniline	DHB/*N*-EA	Positive		
		O-benzylhydroxylamine/2,5-dihydroxybenzoic acid/Na	BOA/DHB/Na	Positive	Glycans	289
	Ionic liquids based on CHCA	α-Cyano-4-hydroxy-cinnamic acid/*N,N*-diethylaniline	CHCA/DEA	Positive	Peptides, oligonucleotides, and phospholipids	290
		α-Cyano-4-hydroxy-cinnamic acid/3-(dimethylamino)-1-propylamine	CHCA/DMAPA	Positive	Peptides	291
		α-Cyano-4-hydroxycinnamic acid/2-amino-4-methyl-5-nitropyridine	CHCA/2A4M5NP	Positive	Peptides	292
		α-Cyano-4-hydroxy cinnamic acid/*N,N*-dimethylaniline	CHCA/DMA	Positive		
		α-Cyano-4-hydroxycinnamic acid/aniline	CHCA/ANI	Positive	Tissue samples	293
				Positive	Amino acids, peptides, proteins, lipids, phospholipids, synthetic polymers, and sugars	294
				Positive		
		α-Cyano-4-hydroxycinnamic acid/3-acetylpyridine	CHCA/3-AP	Positive	Peptides and proteins	295
		α-Cyano-4-hydroxycinnamic acid/phenylenediamine	CHCA/PD	Positive		
		α-Cyano-4-hydroxycinnamic acid/1-methylimidazole	CHCA/IM	Dual-Polarity	Phospholipids	296
				Negative	Gangliosides	297
				Positive	Polyhexamethylene guanidine oligomers	298
				Dual-Polarity	Uncomplexed highly sulfated oligosaccharides	299
		α-Cycno-4-hydroxy-cinnamic acid/tripropylamine	CHCA/Trip	Positive	Phospholipids, cholesterol, and peptides	300
				Positive		
		N-isopropyl-N-methyl-N-tert-butylammonium α-cyano-4-hydroxycinnamate	CHCA/IMTBA	Positive	Peptides, proteins	301

Matrix class	Sub class	Matrix names	Acronyms	Ionization mode	Targets	Ref.
		N,N-diisopropylethylammonium α-cyano-4-hydroxycinnamate	CHCA/DIPEA	Positive<br>Positive	Biodegradable polymers	302
		N,N-diisopropylethylammonium ferulate	FA/DIPEA	Positive	Peptides, proteins, and carbohydrates	301
		α-Cycno-4-hydroxy-cinnamic acid/tributylamine	CHCA/TBA	Positive	Phospholipids	303
		α-Cyano-4-hydroxycinnamic acid butylamine	CHCAB	Positive<br>Positive	Peptides and proteins<br>Phospholipids	286<br>304
		3,5-Dimethoxycinnamic acid triethylamine	SinTri	Positive	Peptides and proteins	286
		α-Cycno-4-hydroxy-cinnamic acid/pyridine	CHCA/Pyr	Positive	Proteins	291
		1-Butylamine-α-cyano-4-hydroxycinnamic acid	1-Butylamine-CHCA	Dual-Polarity	Sulfoglycolipids	305
		α-Cycno-4-hydroxy-cinnamic acid/3-aminoquinoline	CHCA/3-AQ	Positive<br>Positive<br>Positive	Peptides<br>HMW polyrotaxanes<br>Peptide mass mapping	306<br>307<br>308
		α-Cycno-4-hydroxy-cinnamic acid/3-aminoquinoline/ammonium dihydrogen phosphate	CHCA/3-AQ/ADP	Positive	Phosphopeptide	309
		Bis-1,1,3,3-tetramethylguanidinium α-cyano-4-hydroxycinnamate	G2CHCA	Positive	Dermatan sulfate and chondroitin sulfate oligosaccharides	310
		Bis-1,1,3,3-tetramethylguanidinium p-coumaric acid	G_3CA	Positive	Sulfated/sialylated/neutral oligosaccharides and glycopeptides	311
		α-Cyano-4-hydroxycinnamate/norharmane	CHCA.nHo	Dual-Polarity	Carbohydrate	312
		Et3N-α-cyano-4-hydroxycinnamic acid	Et3N-alpha-CHCA	Positive	LMW aflatoxins	313
	Others	2-(4-Hydroxyphenylazo)benzoic acid/1,1,3,3-tetramethylguanidine	HABA/TMG2	Negative	Heparin and heparan sulfate oligosaccharides	314
		2-(4-Hydroxyphenylazo)benzoic acid/spermine	HABA/SPM	Negative		

Matrix class	Sub class	Matrix names	Acronyms	Ionization mode	Targets	Ref.
		1,1,3,3-Tetramethylguanidinium 2,4,6-trihydroxyacetophenone	GTHAP	Positive	Glycopeptides and glycans out of total tryptic digests	315
		N,N-diisopropylethylammonium 3-oxocoumarate	3-HC/DIPEA	Positive	Aliphatic biodegradable photoluminescent polymers	316
		N,N-diisopropylethylammonium/dihydroxymonooxoacetophenoate	THAP/DIPEA	Positive		
		p-Nitroaniline/butyric acid	PNA/butyric acid	Positive	Phosphorylated lipids in biological fluids	317
		1-Butyl-3-methylimidazolium tetrafluoroborate	$[BMIM]BF_4$	Positive	Low-polar small molecules	318
		2,5-Dihydroxybenzoic acid/n-butylamine	DHB/n-butylamine	Positive	Synthetic polymer	319
		Sinapinic acid/triethylamine	SA/triethylamine	Positive		
		Trans-2-(3-(4-t-butylphenyl)-2-methyl-2-propenylidene) malononitrile/triethylamine	DCTB/triethyl amine	Positive		
		Mefenamic acid/aniline	MA/ANI	Positive	Drugs, carbohydrate, and amino acids	320
		Mefenamic acid/*N,N*-dimethylaniline	MA/DMA	Positive		
		Mefenamic acid/pyridine	MA/Pyr	Positive		
		Mefenamic acid/2-methyl picoline	MA/2-P	Positive		
		6-Aza-2-thiothymine/pyridine	ATT/Pyr	Dual-Polarity	MicroRNA biomarkers	35
		3-Hydroxypicolinic acid/base	3-HPA/base	Positive	DNA oligomers	321
		α-Cyanohydroxycinnamic/base	CHCA/base	Positive	Lignin	322
		2,5-Dihydroxybenzoic acid/base	DHB/base	Positive		
		Ferulic/base	FA/base	Positive		
Binary and hybrid-based matrices	Binary and hybrid based on DHB	2,5-Dihydroxybenzoic acid/spermine	DHB/SPM	Negative	Acidic glycoconjugates	323
		2,5-Dihydroxybenzoic acid/glycerol	DHB/glycerol	Positive	Peptides, proteins, and oligosaccharides	324

Matrix class	Sub class	Matrix names	Acronyms	Ionization mode	Targets	Ref.
		2,5-Dihydroxybenzoic acid/2,6-dihydroxybenzoic acid	DHB/2,6-DHB	Positive	Glycans	325
		2,5-Dihydroxybenzoic acid/2,5-dihydroxybenzohydrazide	DHB/DHBH	Dual-Polarity	N-glycans	326
		2,5-Dihydroxybenzoic acid/2,5-dihydroxyacetophenone	DHB/2,5-DHAP	Dual-Polarity	Phosphatidylethanolamine	59
		2,5-Dihydroxybenzoic acid/procaine	DHB/procaine	Positive	Oligosaccharides	327
		2,5-Dihydroxybenzoic acid/1,5-diaminonaphthalene/potassium ion	DHB/DAN/K^+	Positive	O-antigen glycan	328
		2,5-Dihydroxybenzoic acid/alkylated hydroxychalcone	DHB/AHC	Positive	Hydrophobic peptide	329
	Binary and hybrid based on CHCA	α-Cyano-4-hydroxycinnamic acid/nitrocellulose	CHCA/nitrocellulose	Positive	Dimethylarginines in protein hydrolysates	330
		α-Cyano-4-hydroxycinnamic acid/9-aminoacridine	CHCA/9-AA	Dual-Polarity	Small molecules	331
		α-Cyano-4-hydroxycinnamic acid/3-hydroxypicolinic acid	CHCA/3-HPA	Dual-Polarity	Phosphopeptides	332
		α-Cyano-4-hydroxycinnamic acid/p-phenylenediamine	CHCA/PPD	Positive	Antibiotics	333
		α-Cyano-4-hydroxycinnamic acid/2,5-dihydroxybenzoic acid	CHCA/DHB	Positive	Peptides, proteins	334
				Dual-Polarity	Phospholipids	335
		Nonionic detergent n-octyl-β-D-glucopyranoside(OGP)/ phosphoric acid(PA)/α-cyano-4-hydroxycinnamic acid(CHCA)	TOPAC	Positive	Phosphorylated amyloid-β peptides	336
	Others	2,3,4-Trihydroxyacetophenone/2,4,6-trihydroxyacetophenone/ammonium citrate	2,3,4-THAP/2,4,6-THAP/AC	Negative	DNA	337
		3-Hydroxypicolinic acid/pyrazinecarboxylic acid	3-HPA/PCA	Positive	Oligodeoxynucleotides	338

Matrix class	Sub class	Matrix names	Acronyms	Ionization mode	Targets	Ref.
		3-Hydroxycoumarin/6-aza-2-thiothymine	3-HC/ATT	Positive	Small molecules	339
		1,8-Bis(dimethylamino)naphthalene/9-aminoacridine	DMAN/9-AA	Negative	Lipid fingerprinting of intact bacteria	340
		N-(1-naphthyl) ethylenediamine dihydrochloride/9-aminoacridine	NEDC/9-AA	Dual-Polarity	Lipids	341
		Rhodamine (R) 575/α-cyano-4-hydroxycinnamic acid	R575/CHCA	Positive	LMW analytes	342

Supporting Information Table S5. Common-used dopants in organic matrices for MALDI-MS/MSI.

Matrix class	Matrix names	Acronyms	Ionization mode	Targets	Ref.
Ammonium salts compounds	NH_4F		Negative	Oligonucleotides	174
			Dual-Polarity	Lipids	343
	NH_4Cl		Negative	Oligonucleotides	174
			Negative	Neutral oligosaccharides	344
	NH_4Br		Negative	Oligonucleotides	174
	NH_4I		Negative		
	Diammonium citrate	DAC	Dual-Polarity	Phosphorylated peptides	345
Organic amine compounds	Spermine tetrahydrochloride	SPM-4HCl	Negative	Oligonucleotides	346
	Spermine	SPM	Negative		
	Spermidine trihydrochloride	SPD-3HCl	Negative		
	Spermidine	SPD	Negative		
	Tetraamine Spermine	TETA-SPM	Negative	Oligonucleotides	347
	Dimethylforamide	DMF	Positive	Substance P (SP) and betacyclodextrin (BCD)	348
Carbohydrates	Fucose		Negative	Oligonucleotides	349
			Positive	Recombinant proteins	350
	Sorbitol		Positive	Hydrophobic proteins	351
	Cyclodextrin		Positive	Substance P and adenosine	352
Acid compounds	Ethylenediaminetetraacetic acid	EDTA	Negative	Bacterial endotoxins (lipid A)	353
	Trifluoroacetic acid	TFA	Negative	Phosphorylated and ketodeoxyoctonic acid lipids A	354
			Positive	Protein	355
	Phosphoric acid	PA	Dual-Polarity	Phosphopeptides and phosphoproteins	356
			Positive	Phosphopeptides and non-phosphopeptides	357
	Methanediphosphonic acid	MDPNA	Positive	Phosphopeptides	358
	Nitrilotriacetic Acid	NTA	Positive	Peptide	359
	Alkylated dihydroxybenzoic acid	ADHB	Dual-Polarity	Hydrophobic peptide	360
	Formic acid	FMA	Positive	Hydrophobic proteins	351
	Perfluorooctanoic acid	PFOA	Positive		
Salt compounds	Silver trifluoroacetate	AgTFA	Positive	Oligomeric carbon, siloxane series	361
			Positive	Polybutadiene	362
			Positive	Polystyrene	363
	Silver benzoate	AgBz	Positive	Polybutadiene	362
	Silver nitrate	$AgNO_3$	Positive	Polybutadiene	362
			Positive	Unsaturated lipids	364
	Silver p-toluenesulfonate	AgTS	Positive	Polybutadiene	362
	Copper(II) chloride	$CuCl_2$	Positive	Polystyrene	363

Matrix class	Matrix names	Acronyms	Ionization mode	Targets	Ref.
			Positive	Ultra-high molecular weight polystyrene	365
			Positive	Polymers	366
	Copper(I) chloride	CuCl	Positive	Polystyrene	367
			Positive		
	Copper(I) bromide	CuBr	Positive		
	Copper(II) bromide	$CuBr_2$	Positive	Ultra-high molecular weight polystyrene	365
	Copper(I) iodide	CuI	Positive		
	Copper(II) acetate	$Cu(OAc)_2$	Positive		
	Caesium chloride	CsCl	Positive	Phosphatidylcholine mixtures	368
	Lithium citrate	LiCitrate	Positive		
	Lithium acetate	LiAc	Positive		
	Lithium trifluoroacetate	LiTFA	Positive	Lipids	369
	Lithium iodide	LiI	Positive		
	Lithium chloride	LiCl	Positive		
	Ammonium nitrate	AN	Negative	Neutral N-linked carbohydrates	370
	Acetates		Positive		
	Chlorides		Positive	Lipids	371
	Nitrates		Positive		
	Alkylsulfonates		Negative	Neutral oligosaccharides	372
	Transferrin	Tf	Positive	Insulin	373
Others	Cucurbituril	CB[n]	Positive	Polyamines	374
	Black phosphorus	BP	Positive	Intact cells, peptides, and amino acids	375

Supporting Information Table S6. Inorganic matrices with their preferred ionization mode and applications for MALDI-MS/MSI.

Matrix class	Sub class	Matrix names	Acronyms	Ionization mode	Targets	Ref.
Metallic nanostructures	Metal Nanomaterials	Bare gold nanoparticles	AuNPs	Dual-Polarity	Peptides and proteins	92
				Positive	Small neutral carbohydrates	93
				Positive	Endogenous and exogenous compounds embedded in latent fingerprints	94
				Positive	Aminothiols	95
				Positive	Small metabolites	96
				Positive	Neutral steroids	376
		Citrate-capped gold nanoparticles	Citrate-capped AuNPs	Positive	Triacylglycerols in crude lipid mixtures	377
				Positive	Small aminergic neurotransmitters	378
		Hexadecyltrimethylammonium bromide-adsorbed gold nanoparticles	CTAB-AuNPs	Negative	Nucleoside monophosphate	379
		Nile Red-adsorbed gold nanoparticles	NR-AuNPs	Positive	Aminothiols	380
		Aptamer-modified gold nanoparticles	Apt-AuNPs	Dual-Polarity	ATP and glutathione	381
		Dopamine dithiocarbamate-functionalized gold nanoparticles	DDTC-AuNPs	Positive	Small molecules and phosphopeptides	382
		Gold nanoparticles modified with alkylamine	AuNPs-alkylamine	Negative	Glycosphingolipids	383
		Cysteine modified small ligament Au nanoporous film	Cysteine modified Au NPFs	Positive	Amino acids, drug, cyclodextrins, peptides, and polyethylene glycols	384
		Gold nanoparticles with hexagonal boron nitride nanosheets	Au@BN	Positive	Fipronil and other small molecules in strawberries and zebrafish	385
		Gold nanoparticles/cellulose nanocrystals	AuNPs/CNC	Positive	Intact proteins extracted from serum exosomes of non-small cell lung cancer patients	386
		Gold nanoparticle enhanced target	AuNPET	Positive	LMW compounds	387
				Positive	Potential clear cell RCC biomarkers	388
		Silver nanoparticles	AgNPs	Negative	Estrogens	97
				Dual-Polarity	Glycerophospholipids	98
				Dual-Polarity	Neutral lipids, such as cerebrosides	99

Matrix class	Sub class	Matrix names	Acronyms	Ionization mode	Targets	Ref.
		Silver-109 Nanoparticles	^{109}AgNPs	Positive	Fentanyl and other illicit drugs	389
		Citrate-capped silver nanoparticles	Citrate-capped AgNPs	Positive	Peptides	390
		Silver nanoparticles modified with alkylcarboxylate and alkylamine	C_mAgNP/C_nNH_2s	Negative	Fatty acids	60
		Surface modified silver selinide nanoparticles	Ag_2Se NPs	Positive	Peptide, protein	391
		Polyvinylpyrrolidone capped silver nanoparticles	PVP-AgNPs	Positive	Lipids	392
		Sol-gel-derived silver-nanoparticle-embedded thin film	AgNPs thin film	Positive	Peptides, triacylglycerols, and phospholipids	393
		Highly porous silver foils	Ag foils	Positive	Cuticular extracts of fruit flies (Drosophila melanogaster) and worker bees (Apis mellifera)	394
		Silver-109 nanoparticle enhanced steel target	^{109}AgNPs	Positive	Drugs and many other compounds	395
				Positive	Amino acids	396
				Dual-Polarity	Metabolites	397
		Silver nanoparticle target	AgNP targets	Positive	Bacterial lipids profiles	398
				Positive	LMW compounds such as triglycerides, saccharides, amino acids, and carboxylic acids	399
		Aluminum powder	AlNPs	Positive	Poly(ethylene glycol) 200 (PEG 200) and methyl stearate	100
		Manganese powder	MnNPs	Positive		
		Molybdenum powder	MoNPs	Positive		
		Silicon powder	SiNPs	Positive		
		Tin powder	SnNPs	Positive		
		Tungsten powder	WNPs	Positive		
		Zinc powder	ZnNPs	Positive		
		Platinum nanoparticles	PtNPs	Positive	Small biomolecules and microwave digested proteins	400
		Fluorocarbon-based hydrophobic perfluorodecyltrichlorosilane-Pt Nf substrates	FDTS-PtNfs	Negative	Amino acids and peptides	401
		Prefabricated platinum nanomaterial matrix	Prefabricated Pt NMs	Positive	Oligosaccharides and lipids in plant tissues	402
		Palladium nanoparticles	PdNPs	Positive	Fatty acids, triacylglycerols, carbohydrates, and antibiotics	403

Matrix class	Sub class	Matrix names	Acronyms	Ionization mode	Targets	Ref.
		Ferric nanoparticles	FeNPs	Positive	Metabolic fingerprinting of diabetic retinopathy	404
		Nano-zero-valent iron	nZVI	Negative	Serum metabolic fingerprinting	405
	Metal Oxide Nanomaterials	Titanium dioxide nanoparticles	TiO_2 NPs	Positive	PEG 200 and methyl stearate	100
				Positive	Peptide and protein	406
				Positive	LMW metabolites	101
				Positive	Lipids	102
		Holmium modified titanium dioxide nanocomposites	Ho-TiO_2	Positive	Bisphenol S and Indigo	407
		Dopamine-modified titanium dioxide nanoparticles	TiO_2-DA	Positive	Small metabolites and lipids in mouse brain tissue	408
					Serum lipids	409
		Titanium dioxide sol-gel-deposited thin film	TiO_2 sol-gel film	Positive	α-Cyclodextrin	410
		Photocatalytically patterned titanium oxide arrays	PPTA	Positive	Phosphopeptides	411
		Nanostructured titanium dioxide plate	TiO_2 plate	Positive	Phosphopeptides	412
		Titanium dioxide films	TiO_2 films	Positive	Phosphopeptides	413, 414
		Chemically functionalized titanium dioxide nanotube layers	TiO_2 nanotube layers	Positive	Peptides and small molecules	415
		Functionalized titanium oxide nanowire substrate	TiO_2 nanowire	Positive	Vinca alkaloids in the petal	416
		Zinc oxide nanoparticles	ZnO NPs	Positive	Low molecular weight organic compounds and synthetic polymers	417
				Positive	PEG 200 and methyl stearate	100
		Surface modified zinc oxide nanorod array chip	Surface modified ZnO NR array	Positive	Protein	418
		Silane-immobilized magnetic iron oxide particles	Silane-immobilized Fe_3O_4 NPs	Positive	Peptide, protein and cytochrome C	419
		Magnetic iron oxide nanoparticles	Fe_3O_4 NPs	Positive	Asphaltene molecules	103
		Nickel oxide nanoparticles	NiO NPs	Positive		
		Tricobalt tetraoxide nanoparticles	Co_3O_4 NPs	Positive		

Matrix class	Sub class	Matrix names	Acronyms	Ionization mode	Targets	Ref.
		Desorption ionization using through hole alumina membrane	DIUTHAME	Positive	Phospholipid	420
		Tricobalt tetraoxide nanocrystals	Co_3O_4 nanocrystals	Dual-Polarity	Amino acids, harmful additives and pesticide	421
		Tricobalt tetraoxide/cobalt hydroxide heterojunctions	$Co_3O_4/Co(OH)_2$	Positive	Environmental pollutant	422
		Cerium oxide nanoparticles	CeO_2 NPs	Positive	Phosphopeptides	423
		Microstructured tungsten oxide	WO_3 substrate	Positive	Polypeptides and drug species	424
		Tungsten oxide powder	WO_3 NPs	Positive	PEG 200 and methyl stearate	100
	Other metallic nanomaterials	Multi-component mesoporous tungsten titanate	WTiO	Positive	Short peptides	425
		2-Hydroxy octadecanoic acid-modified barium titanate nanoparticles	HOA-modified $BaTiO_3$ NPs	Positive	Phospholipids	426
		Gold manganese oxide hybrid nanoflowers	Au@MnO	Dual-Polarity	ATP	427
		Mesoporous NiO@ZnO nanofiber membranes	NiO@ZnO	Positive	Urine metabolism	428
		Nitrogen-rich $Ag@Ti_3C_2$	$Ag@N-Ti_3C_2$	Positive	Xenobiotic pesticides and other endogenous small molecules	429
		Trimetallic alloy	PdPtAu	Positive	Noble metal-thiol hybridization	430
		Porous platinum-copper alloy	Porous PtCu alloy	Positive	Plasma metabolic fingerprints	431
		Mesoporous palladium-platinum alloys	Mesoporous PdPt alloys	Positive	Metabolic fingerprints in plasma	432
		Plasmonic two-dimensional black phosphorus nanosheet-gold nanomatrix	Plasmonic 2D BP@Au	Positive	Anticancer drugs include irinotecan (CPT-11) with its metabolically active derivative	433
		Metal-polyphenol network nanocomposite	Au-MPN@Ag nanocomposite	Positive	Metabolites	434
		Metal-phenolic network functionalized AuNPs	M-TA@AuNPs	Positive	Amino acids	435
Carbon-based Nanomaterials	Fullerenes	Fullerene	C60	Dual-Polarity	Phosphotungstic acid	436,437
		C70 fullerene	C70	Dual-Polarity	Nonderivatized steroids	438

Matrix class	Sub class	Matrix names	Acronyms	Ionization mode	Targets	Ref.
		Starlike water-soluble fullerene derivative, hexa(sulfonbutyl)fullerene	$C60[(CH_2)_4SO_3^-]_6$; HSBF	Positive	Amino acids, peptides, and proteins.	439
		Bromoacetyl functionalized C60	Br-C60	Negative	LMW thiols in serum	440
		Water-soluble fullerenol	$C60(OH)_{24-26}$	Dual-Polarity	Small molecules, and saccharin sodium in foods	441
		Functionalized fullerenes	$C60((CH_2)_2COOH)n$	Positive	Peptides and phospholipids	442
	Carbon nanotubes	Carbon nanotubes	CNTs	Positive	Peptides, organic compounds, and β-cyclodextrin	104
				Positive	Neutral small carbohydrates	105
				Positive	Peptides and proteins	106
		Oxidized carbon nanotubes	Oxidized CNTs	Positive	Low-mass compounds in environmental samples	107
				Positive	Small molecules	108
				Positive	Small carbohydrates and amino acids	443
		Water-soluble multi-wall carbon nanotubes and polyaniline composites	MWCNTs@PANI	Dual-Polarity	Small molecular-weight metabolites	444
		Highly water-dispersible multiwalled carbon nanotubes@polydopamine	MWCNTs@PDA	Dual-Polarity	Small molecules	445
		Transition metal carbides with multi-walled carbon nanotubes	Mxene/MWCNTs	Positive	Dysregulated leukotriene metabolism	446
	Graphene and graphene oxide	Graphene	G	Positive	Small molecules	109
				Negative	Small molecules	110
				Positive	Hydrophobic compounds	447
				Dual-Polarity	Endogenous caffeine and theanine	111
				Positive	Probe the interactions between transition metals of Fe(II), Fe(III), Cu(II) with a non steroidal anti-inflammatory drug (NSAID), flufenamic acid (FF)	448
				Dual-Polarity	Flavonoids	112
		Graphene oxide	GO	Positive	Disaccharides	113
				Negative	Small molecules	114
				Positive	Small molecular components	115
		Fluorographene	FG	Dual-Polarity	Quaternary ammonium halides	449

Matrix class	Sub class	Matrix names	Acronyms	Ionization mode	Targets	Ref.
		Gas-phase N-doped graphene	gNG	Negative	Small molecule	450
		O-P,N-doped carbon/graphene	O-P,N-C/G	Dual-Polarity	Small molecules	451
		Three-dimensional mesoporous graphene	3D-MG	Dual-Polarity	Polyphenols	452
		Acid-oxidized graphene	AOG	Negative	Nonpolar analytes	453
		4-vinylphenylboronic acid-functionalized graphene oxide	GO-VPBA	Positive	Small molecule compounds with vicinal diols	454
		Aggregated graphene oxide	AGO	Positive	Triacylglycerols	455
		Magnetic graphene	Fe_3O_4/G	Dual-Polarity	Small molecules, traditional Chinese medicine and nicotine metabolites	456
		Magnetic graphene composites	MAOG	Negative	Nitropolycyclic hydrocarbons (nitro-PAHs)	454
		Fe_3O_4 nanoparticle/graphene oxide magnetic nanocomposite	Fe_3O_4/GO	Positive	Low-abundance peptides and phosphopeptides	457
					Pesticides (i.e., carbofuran and carbendazim)	458
		Graphene oxide-functionalized magnetic composites	GO@NH_2@Fe_3O_4	Positive	Quinolone antibiotics	459
		Hybrid bismuth oxide-graphine oxide nanomaterials	Bi_2O_3@GO	Negative	Small molecules	460
	Graphite	Graphite/liquid mixed matrices	Graphite particles/glycerol	Positive	Peptides and proteins, as well as of lower molecular weight ana-lytes	461
				Positive	Intermediate weight analytes	462
		Colloidal graphite		Positive	Cerebrosides and sulfatides	463
		Graphite		Positive	Radionuclides	464
		Graphite plate		Positive	Small macromolecules	465
				Positive	Fatty acids	466
				Positive	LMW poly(methylsilsesquioxane)s	467
		Pencil lead		Positive	Peptides, polymers and actinide metals	468
		Graphite sheet		Positive	Amino acids, peptides, and polyethylene glycol polymers	469
		Graphite carbon black	GCB	Dual-Polarity	Taste- and odor-active compounds	470
					Chemical patterns of soy sauce products	471
	Carbon nanofibers	Derivatized graphitic nanofibres	GNFs	Positive	Peptides and proteins	472

Matrix class	Sub class	Matrix names	Acronyms	Ionization mode	Targets	Ref.
	Nanodiamond	Nanodiamond	ND	Positive	Proteins	473
		Functionalized diamond nanopowder	diamond-IDA-Fe^{3+}/La^{3+}	Positive	Phosphopeptides	474
	Carbon nanohorns	Functional single-walled carbon nanohorns	Apt-SWNHs	Dual-Polarity	Amino acids, peptides, and fatty acids	475
	Carbon nanodots	Carbon nanodots	CDs	Dual-Polarity	Glucose and uric acid	476
				Dual-Polarity	Anti-inflammatory drug	477
	Graphdiyne	Graphdiyne	GD	Negative	Fatty acids, amino acids, peptides and drugs	478
		Gold nanoparticles-decorated graphdiyne	Au/GDY	Positive	Exogenous/endogenous components in biological tissues	479
	Carbon Nanoparticles	Washed carbon soot nanoparticles	WCS NPs	Dual-Polarity	Carbohydrates, polymers, peptides, drugs, dyes and fatty acids	480
		Carbon nanoparticles	CNPs	Positive	N-glycans	481
	Graphitic carbon	Porous graphitic carbon	PGC	Positive	Neutral lipids	482
		Graphitic carbon nitride nanosheets	g-C3N4 nanosheets	Dual-Polarity	Amino acids, nucleobases, peptides, bisphenols (BPs), and nitropolycyclic aromatic hydrocarbons (nitro-PAHs)	483
	Carbon nanowalls	Boron-doped carbon nanowalls	B-CNWs	Positive	Different types of compounds such as fatty acids, lipids, metabolites, saccharides and peptides	484
					Cytochrome C and ricin	485
Silicon-based nanostructures	Porous Silicon-DIOS	Porous silicon	pSi/DIOS	Positive	Peptides, small molecules, and WIN antiviral drug	116
				Positive	Small molecules	117
				Positive	Secondary metabolites	118
				Positive	Protein characterization	119
				Positive	Low-mass components of polyesters	120
		Porous silicon dioxide	DIOSD	Positive	Catecholamines	486
	Porous Silicon-NIMS	Nanostructure-initiator mass spectrometry	NIMS	Positive	Peptides and metabolites	121
				Positive	Xenobiotics and endogenous metabolites	122
				Negative	Phosphorylated metabolites	123
		Cation-enhanced nanostructure-initiator mass spectrometry	Cation-enhanced NIMS	Positive	Carbohydrates and steroids	487
		Black silicon based nanostructure-initiator mass spectrometry	Black silicon based NIMS	Positive	Small molecules	488
		Nanostructure-initiator	Nimzyme	Positive	β-1,4-galactosidase	489

Matrix class	Sub class	Matrix names	Acronyms	Ionization mode	Targets	Ref.
		mass spectrometry based enzyme assay		Positive	Three stereoisomers (maltose, lactose and cellobiose)	490
	Silicon/Silica Nanoparticles	Silicon nanoparticles	SiO_2 NPs	Dual-Polarity	Small molecules	491
				Negative	Lignin	492
		Gold-silica core-shell nanoparticles with ultrathin silica shell of 2-4 nm	$Au@SiO_2$ CSNPs	Positive	Small molecules	493
		Au coated SiO2 nanoshells	$SiO_2@Au$ nanoshell	Positive	Metabolomics	494
		Graphene-coated silicon wafer plate	G/SiO_2 plate	Positive	Proteins	495
	Silicon Nanowires	Silicon nanowires	SiNWs	Positive	Small molecules	496
				Positive	Lipids	497
				Positive	Small molecules, peptides, and a bovine serum albumin (BSA) digest	498
				Negative	Metabolites	499
		Fluorinated ethylene propylene coated silicon nanowires chips	FEP@SiNWs	Negative	Metabolomics and peptidomics	500
	Nanopost arrays	Silicon nanopost arrays	NAPAs	Dual-Polarity	Microbial single cell metabolomics	501
				Positive	Hexosylceramides (HexCers) and phosphatidylethanolamines (PEs), neutral lipids, such as TGs	502
				Dual-Polarity	Lipids, (+) phosphatidylcholines and phosphatidic acids, (-) sulfatides and free fatty acids	503
				Positive	Neutral lipids and biooligomers	504
	Orther silicon-based nanostructure	Nanoscale calcinated silicate film on a gold substrate	SiOx/Au	Positive	Peptides	505
		Octadecyltrichlorosilane monolayer-calcinated nanofilm on a gold substrate	OST-SiOx/Au	Positive	Peptides	506
		Mesocellular siliceous foams	MCF	Positive	Fingermarks	507
		Nanostructured silicon	nSi	Positive	Peptides	508
		Diatom *Thalassiosira pseudonana* cell walls	*T. pseudonana* cell walls	Dual-Polarity	PEG600, D-sphingosine and raffinose	509

Matrix class	Sub class	Matrix names	Acronyms	Ionization mode	Targets	Ref.
		Dinoflagellate *Prorocentrum minimum* cell walls	*P. minimum cell* walls	Dual-Polarity		
		Celite		Dual-Polarity		
		1H,1H,2H,2H-perfluorooctyldimethlychlorosilane modified T. pseudonana cell walls	PFOS-diatom cell walls	Positive	Small analytes	510
		Pentafluorophenylpropyl dimethylchlorosilane modified T. pseudonana cell walls	PPS-diatom cell walls	Positive		
Organic frameworks	Metal-organic frameworks	Material of Institute Lavoisier-100(Fe)	MIL-100(Fe)	Positive	Polycyclic aromatic hydrocarbons (PAHs)	511
				Positive	Mono-/di-saccharides, peptides and complex starch digests	512
		Material of Institute Lavoisier-100(Cr)	MIL-101(Cr)	Positive	Quercetin	513
		Ti-based metal-organic frameworks nanosheets	NTU-9 nanosheets	Dual-Polarity	Saccharides	514
		Zeolitic imidazolate framework nanocrystals	ZIF-8	Negative	Environmental pollutant	515
		Three-dimensional mesoporous graphene and zirconium-based metal-organic frameworks	MG@UiO-66	Positive	Steroids	516
		Nanoporous carbons derived from metal-organic frameworks	cMIL-53, cCYCU-3	Positive	Biomolecules, synthetic polymers, and even small organic compounds	517
		Palladium nanoparticles decorated thiol-functionalized metal organic framework nanocomposite	UiO-66-$(SH)_2$@Pd NPs	Positive	Oligosaccharide isomers	518
		Boric-acid-modified multifunctional Zr-based metal-organic frameworks	Fe_3O_4@PDA @B-UiO-66	Positive	Glucose	519
		Magnetic Zr-based metal-organic frameworks	Fe_3O_4@PDA @ZrMOF	Negative	PM2.5 samples	520
		Zeolitic imidazolate framework-8 coated magnetic nanocomposites	Fe_3O_4@ZIF-8 MNCs	Negative	Small molecules	521

Matrix class	Sub class	Matrix names	Acronyms	Ionization mode	Targets	Ref.
		Core-shell structured magnetic nanosphere	Fe_3O_4@NTU-9	Positive	Urinary exosome metabolite fingerprints	522
		Metal-organic framework-derived metal oxide nanomaterial	CoFeNMOF-D	Positive	Serum metabolic fingerprints	523
		Core-shell MOF@MOF nanoparticle	UiO-66-$(OH)_2$@UiO-66-NH_2	Positive	Small-molecule compounds in biological samples	524
		Functional metal-organic frameworks	MIL-101(NH_2)@Au-Cys	Positive	*N*-glycans	525
		Maltose-functional metal-organic frameworks	MIL-101-maltose	Dual-Polarity	Small biomolecule	526
		Reactive metal-organic framework	NH_2NH-MOF	Positive	Small aldehydes	527
	Covalent-organic frameworks	Covalent organic framework TpBD	spherical COF TpBD	Positive	Amino acids and fatty acids, environmental pollutants like bisphenol S (BPS) and pyrene	528
		The condensation of 1,3,5-triformylbenzene and p-phenylenediamine	COF-LZU1	Negative	Fluorochemicals	529
		Spherical vinyl-functionalized covalent-organic framework	COF-V	Positive	Small molecules	530
		Covalent organic framework nanofilm	COF film	Negative	5-Fluorouracil	531
				Negative	Small molecules such as amino acids, bisphenols (Bps), estrogens, and drugs homocysteine	532
		Boric-acid-functionalized covalent organic framework	B-COFs	Negative	Cis-Diol-Containing Compounds	533
		Sulfonic acid functionalized hierarchical porous covalent organic frameworks	H-COF-SO_3H	Positive	Quaternary ammonium herbicides paraquat (PQ) and diquat (DQ)	534
		Magnetic covalent organic framework	Fe_3O_4@COFs	Positive	PAHs and their derivatives in PM2.5	535
		Glutathione-functionalized silver nanoparticle-grafted covalent organic framework	TpPa-1@Ag@GSH	Positive	*N*-linked glycopeptides	536
		Layered imine-based covalent organic polymer with mesopores	p-TpBDH-OH	Positive	*N*-glycopeptides	537

Matrix class	Sub class	Matrix names	Acronyms	Ionization mode	Targets	Ref.
		Boric acid-functionalized magnetic covalent organic framework with polyethyleneimine	mCOF@PEI @$B(OH)_2$	Positive	*N*-glycopeptides	538
		Spherical covalent organic framework	TPB-BPTP-COF	Positive	Triphenyl phosphate on breast cancer in mice	539
		Gold-modified covalent organic frameworks	COF-S@Au NP	Positive	Metabolites induced by triclosan exposure	540
		Donor-acceptor covalent organic framework nanofilm	D-A COF nanofilm	Negative	Low-weight molecules, such as amino acids, bisphenols, and estrogens creatinine in human serum	541
		Covalent organic frameworks-2,5-dihydroxyterephthalaldehyde and 1,3,5-tris (4-aminophenyl) benzene film	COF-DhaTab film	Negative	PFOS in zebrafish, rat kidney and liver tissues	542
	Hydrogen-bonded organic frameworks	Porous metal-organic frameworks@Hydrogen-bonded organic frameworks	MOF@HOF	Positive	Flavonoids in kumquat and honey orange	543
Quantum dots		Functionalized cadmium selinide quantum dots	CdSe QDs	Positive	Amino acids and peptides	544
		Cysteine-capped zinc selenium quantum dots	ZnSe-Cys QDs	Positive	Protein	545
		Zinc selenium quantum dots modified with 3-mercaptopropionic acid	ZnSe-3MPA	Positive	Peptides and proteins from sodium salt solution	546
		Meso-2,3-dimercaptosuccinic acid modified cadmium telluride quantum dots	DMSA-CdTe QD	Positive	Carbohydrates	547
		3,3′-dithiodipropionic acid di(N-hydroxysuccinimide ester) modified cadmium telluride quantum dots	DSP-CdTe QDs	Positive		
		Mercaptopropionic acid modified cadmium sulfide quantum dots	MPA-CdS QDs	Positive		
		Thioglycolic acid modified cadmium sulfide quantum dots	TGA-CdS QDs	Positive		
		Germanium nanodots	GeNDs	Positive	Poly(ethylene glycol) oligomer	548

**Matrix class**	**Sub class**	**Matrix names**	**Acronyms**	**Ionization mode**	**Targets**	**Ref.**
		Heteroatom-doped graphene quantum dots	HGQDs	Negative	Small Biomolecules	549
		Boron nitride quantum dots	BNQDs	Negative	Bisphenol A	550
		Ternary-doped boron, nitrogen, sulfur-MXene quantum dots-based target plate	3D-MTP (BNS-MQDs)	Positive	Small-molecule environmental pollutants	551

Supporting Information Table S7. Organic-inorganic binary and hybrid matrices with their preferred ionization mode and applications for MALDI-MS/MSI.

Matrix class	Matrix names	Acronyms	Ionization mode	Targets	Ref.
Organic-inorganic binary matrices	2,5-Dihydroxybenzoic acid on Fe_3O_4 nanoparticles	Fe_3O_4/DHB	Positive	Triglycerides, PL in maize	552
	Carbon dots/9-aminoacridine	CDs/9-AA	Positive	Nucleosides, amino acids, oligosaccharides, peptides, and drugs	553
	Magnesium oxide mixed with N-naphthylethylenediamine dihydrochloride	MgO/NEDC	Negative	Lead (Pb) exposure	554
	Nanodiamonds/2,5-dihydroxybenzoic acid	NDs/DHB	Positive	Sweeteners in commercial beverages	555
	α-Cyano-4-hydroxycinnamic acid and tri-potassium citrate salt pre-coated silicon nanopost array	CHCA/NAPA	Positive	Lipids	556
	Humic acids mixed with magnetic iron oxide nanoparticles	MHAs	Positive	Rhodamine B (RdB)	557
Organic-inorganic hybrid matrices	SBA-15 modified α-cyano-4-hydroxycinnamic acid	SBA-15@APTES@CHCA	Positive	Quinolone antibiotics	558
	Submicron 3,4-dihydroxybenzoic acid-titanium dioxide composite particles	3,4-DHB-TiO_2 CPs	Positive	Secondary metabolites	559
	Multilayer Ti_3C_2TX using p-aminoazobenzene	p-AAB/MXene	Positive	Emerging environmental organic pollutants in beverages and PM2.5	560
	Lysine moiety onto the surface of detonated nanodiamonds	ND-COOH	Dual-Polarity	Small organic pharmaceuticals	561
	α-Cyano-4-hydroxycinnamic acid onto the surface of detonated nanodiamonds	ND-CHCA	Dual-Polarity		
	2,5-Dihydroxybenzoic acid onto the surface of detonated nanodiamonds	ND-DHB	Dual-Polarity		
	Sinapinic acid onto the surface of detonated nanodiamonds	ND-SA	Dual-Polarity		
	Amorphous α-cyano-4-hydroxycinnamic acid-functionalized silica	CHCA-SiO_2	Positive	Peptides	562
	α-Cyano-4-hydroxycinnamic acid-modified Au nanoparticles	CHCA-functionalized AuNPs	Negative	Peptides	563
	Alkali-metal cation-substituted zeolites and 2,4,6-trihydroxyacetophenone	THAP-zeolite complexes	Positive	Maltohexaose and acetylsalicylic acid	564

Matrix class	Matrix names	Acronyms	Ionization mode	Targets	Ref.
	Copper oxide particles in combination with low amounts of 2,5-dihydroxybenzoic acid	Cu_2O PS@DHB	Positive	Proteins and glycopeptides	565
	Titanium dioxide nanoparticle was modified with an ionic liquid formed by α-cyano-4-hydroxycinnamic acid and 3-APTES	$[TiO_2–Si–NH_3^+][CHC^-]$	Positive	Small molecules	566
	Combines cyano-4-hydroxy cinnamic acid and mesoporous silica	$[CHC^-]$ $[NH_3^+$-Si-SBA-15-Si-$NH_3^+]$ $[CHC^-]$	Dual-Polarity	Small molecules	567
	Ionic macro-complex between α-cyano-4-hydroxycinnamic acid and a modified mesoporous silica	[SBA-15-Si-$NH_3^+][CHC^-]$	Positive	Two neurotransmitters, dopamine and serotonin	568
	α-Cyano-4-hydroxycinnamic acid-conjugated magnetic nanoparticles	CHCA@MNP	Positive	Small molecules	569
			Positive	Metal ions	570
	2,5-Dihydroxybenzoic acid-conjugated magnetic nanoparticles	DHB@MNP	Positive	Small molecules	569
			Positive	Small molecules	571
			Positive	Metal ions	570
	Sinapinic acid-conjugated magnetic nanoparticles	SA@MNP	Positive	Metal ions	570
	Organic metal chalcogenides	OMC Cu(SPh-COOH)	Positive	Metabolic molecular diagnosis of central precocious puberty	572

Supporting Information Table S8. Representative MALDI matrices for proteomic analysis.

Targets	Matrix names	Acronyms	Ionization mode	Analyte entities	Ref.
Untargeted proteomics	1,8-Bis(dimethylamino)naphthalene	DMAN	Negative	Amino acids, fatty acid-amino acid conjugates (FACs)	573
	Addition of black phosphorus to sinapinic/α-cyano-4-hydroxycinnamic acids	Addition of BP to SA/CHCA	Positive	Amino acids and peptides	375
	4-Hydroxy-3-nitrobenzonitrile	HNBN	Positive	Peptides and proteins	179
	4-hydroxy-3-methoxyphenylpyruvic acid	HMPPA	Positive	Peptides and proteins	201
	Indole-3-pyruvic acid	IPA	Positive	Peptides and proteins（<20kDa）	201
	Hydralazine	HZN	Positive	Proteins	198
	2,5-Dihydroxybenzoic acid and α-cyano-4-hydroxycinnamic acid	DHB/CHCA	Positive	Peptides and proteins	334
	2,5-Dihydroxybenzoate butylamine	DHBB	Positive	Peptides and proteins	286
	α-Cyano-4-hydroxycinnamic acid butylamine	CHCAB	Positive	Peptides and protein enzymatic digest products	304
	Bis-1,1,3,3-tetramethylguanidinium p-coumaric acid	G_3CA	Dual-Polarity	Glycopeptides	311
	3-Aminoquinoline/Bis-1,1,3,3-tetramethylguanidinium p-coumaric acid	3-AQ/G_3CA	Dual-Polarity	Glycopeptides and phosphopeptides	47
	α-Cyano-4-hydroxycinnamic acid and aniline	CHCA/ANI	Positive	Amino acids, peptides, proteins and protein enzymatic digest products	294
	N,N-diisopropylethylammonium/α-cyano-4-hydroxycinnamate N-isopropyl-N-methyl-N-tert-butylammonium/α-cyano-4-hydroxycinnamate	DIEA/CHCA IMTBA/CHCA	Positive	Peptides and proteins	301
	Fullerene–silica		Positive	Amino acids and peptides	574
	Layer-by-layer depositions of rGO and gold nanoparticles	LBL rGO/AuNP	Positive	Amino acids and peptides	575
	Ultrathin graphitic carbon nitride	g-C_3N_4	Negative	Amino acids and peptides	483
	Carbon nanotubes	CNTs	Positive	Small proteins, peptides, and protein enzymatic digest products	106
	Quantum dots	QDs	Dual-Polarity	Amino acids, peptides, and proteins	576
	Metal–organic frameworks	MOFs	Dual-Polarity	Amino acids, peptides, proteins	577
	Water ice		Positive	Peptides and proteins	578
Amino acids	Sodium dodecyl sulfate and sodium octyl sulfate	SDS and SOS	Positive	Phe, Val, Pro, Ala, and Tyr	579

Targets	Matrix names	Acronyms	Ionization mode	Analyte entities	Ref.
	Aggregation-induced emission compounds (*N,N'*-bis(4-hydroxylsalicylidene)-p-phenylenediamine, *N,N'*-bis(4-methoxylsalicyli-dene)-p-phenylenediamine and *N,N'*-bis(salicylidene)-p-phenylenediamine)	BSPD-OH, BSPD-OMe and BSPD	Positive	Arg, His, Pro and Leu; Gly in human urine	276
	3-Hydroxycoumarin and 6-aza-2-thiothymine	3-HC/ATT	Positive	Ala, Thr, Met, and Trp	339
	2,5-Dihydroxyterephthalic acid	DHT	Positive	20 protein amino acids and taurine	155
	Oxidized carbon nanotubes	oxidized CNTs	Positive	Try, Leu, Ile and Arg; Leu and Ile in corn root	443
	Carbon nanotubes	CNTs	Positive	20 protein amino acids	580
	Graphene		Positive	Glu, His, and Trp	109
	Tricobalt tetraoxide nanocrystals	Co_3O_4	Positive	Ala, Asn, His, Pro, Ser and Val	421
	Monolayer-protected gold nanoparticles	AuNPs	Positive	Arg, His, Gly, Glu, Leu, Met, Phe, and Ser	581
Peptides and proteins	Addition of nitrilotriacetic acid to α-cyano-4-hydroxycinnamic acid	Addition of NTA to CHCA	Positive	In-gel digested bovine serum albumin (BSA) trypyic peptides and Rubisco trypyic peptides	359
	3-Hydroxy-4-nitrobenzoic acid	3H4NBA	Positive	2-Nitrobenzenesulfenyl (NBS)-labeled model peptides (ACTH (5–10) peptide), proteins (bovine a-lactalbumin, rabbit glyceraldehyde-3-phosphate dehydrogenase, chicken lysozyme, and chicken ovalbumin) followed by tryptic digestion	143
	α-Cyano-4-hydroxycinnamic methyl ester	CHCE	Positive	CHCA-labeled peptides	255
	(E)-α-cyano-4-hydroxycinnamic acid propyl ester	CHCA-C3	Positive	Porcine insulin, lysozyme, myoglobin, growth hormone, trypsin TPCK treated from bovine pancreas, pepsin, bovine serum albumin (BSA), transferrin from human, IgG from rabbit	256
	4-Chloro-α-cyanocinnamic acid	Cl-CCA	Positive	Bovine serum albumin (BSA) in-solution and in-gel digest products	257
	Dinuclear copper complex (α-cyano-4-hydroxycinnamic acid (CHCA) copper salt	$(CHCA)_4Cu_2$	Positive	Site-specific copper binding of several peptides	582
	Z-sinapinic acid	Z-SA	Positive	Commercial peptides (bradykinin (1-7), bradykinin (1-8), angiotensin I, angiotensin II) and model peptides with different amino acid side chains	9

Targets	Matrix names	Acronyms	Ionization mode	Analyte entities	Ref.
	6-Bromo-ferulic acid	6-BFA	Positive	Large peptides (3–5 kDa) and peptides containing acidic amino acids or proline.	126
	α-Cyano-4-hydroxycinnamicacid/3-aminoquinoline/quinoline	CHCA/3-AQ/Q	Positive	Angiotensin I and protein enzymatic digest products	48
	2-Nitrophloroglucinol	2-NPG	Positive	Cytochrome c,myoglobin, bovine serum albumin (BSA), and immunoglobulin G (IgG)	583
	α-Cyano-5-phenyl-2,4-pentadienic acid	CPPA	Positive	Most examined proteins occurring in milk, hazelnut and in intact bacterial cells of *E. coli*.	265
	Caffeic acid	CA	Positive	Protein from rat brain, *Capparis masaikai* seed, and germinating soybean seed	129
	3-Hydroxy-2-nitrobenzoic acid	3H2NBA	Positive	Angiotensin I, Amyloid β 1–16, amyloid β 1–40, N-acetyl renin substrate, angiotensin I, and adrenocorticotropic hormone fragment 18–39 (ACTH 18–39)	144
	Gold nanoparticles	AuNPs	Positive	Peptide systems and small proteins (e.g., bovine insulin) including posttranslationally modified peptides (e.g., phosphorylated).	92
	α-Cyano-4-hydroxycinnamic acid-modified gold nanoparticles	CHCA-modified AuNPs	Positive	Cytochrome c, TPCK-treated trypsin, [Sar1, Thr8]-angiotensin II, and neurotensin	563
	Hybrid organic–inorganic amorphous α-cyano-4-hydroxycinnamic acid–silica	CHCA-SiO_2	Positive	Small synthetic peptide mixtures (550–1300 Da)	562
	Titanium dioxide-gold/graphene nanocomposites	TiO_2-Au/G nanocomposite s	Positive	Tripeptide glutathione (GSH)	584
	Mesoporous tungsten titanate	WTiO	Positive	Short peptides: tri-peptide (Gly-Leu-Ala), tetra-peptide (Val-Gly-Ser-Glu), and horse heart myoglobin (MYO) trypyic peptides	425
	Highly fluorescent CdSe/ZnS quantum dots	CdSe/ZnS QDs	Positive	Crude tryptic digests of bovine serum albumin (BSA)	585
	Pencil lead		Positive	Substance P	468
	Silicon nanopowder (5-50 nm)		Dual-Polarity	Peptides Arg-Gly, YGGFL	491
	Titanium oxide nanotube layer	TiO_2 nanotube layers	Positive	Desarg1-Bradykinin, angiotensin I, Glu1-Fibrinopeptide B, and neurotensin	415

Targets	Matrix names	Acronyms	Ionization mode	Analyte entities	Ref.
	6-Aza-2-thiothymine	ATT	Positive	Tryptic peptides from Bovine Serum Albumin (BSA) and map in situdigested peptides from formalin-fixed paraffin-embedded (FFPE) tissue sections	44
	Addition of phosphoric acid to 2,5-dihydroxybenzoic acid	Addition of PA to DHB	Positive	Tryptic digests of α/β-casein, *B. subtilis* PrkC phosphoprotein	356
	Addition of monoammonium phosphate to α-cyano-4-hydroxycinnamic acid	Addition of monoammonium phosphate to CHCA	Negative	Bradykinin fragment 2–9, human angiotensin I, human [Glu^{1}]-fibrinopeptide B, human adrenocorticotropic hormone fragment 18–39, triphosphorylated peptide, heat shock protein fragment phosphopeptide, hirudin 55–65 sulfopeptide, acetyl-hirudin 53–65 sulfopeptide, and 2-DE separated proteins of *Caenorhabditis elegans*	586
	5-Amino-1-naphthol	5,1-ANL	Positive	Adrenocorticotropic hormone fragment 18–35 (ACTH18-35), monophosphorylated peptide [$pTyr^{6}$]-ACTH 18–35, diphosphorylated peptide [$pTyr^{6}$, $pSer^{14}$]-ACTH 18–35, and bovine b-casein 1–29 fragment	207
	5-Aminosalicylic acid	5-ASA	Positive	Adrenocorticotropic hormone fragment 18-35 (ACTH18-35), monophosphorylated peptide [$pTyr^{6}$]-ACTH18-35, di-phosphorylated peptide [$pTyr^{6}$, $pSer^{14}$]-ACTH18-35, [Arg^{36}]-ACTH18-36, ACTH19-36: , [Arg^{36}]-ACTH19-36, [Arg^{22}]-ACTH22-39, deuterium-labeled dodecapeptide), and [Arg^{8}]-vasopressin	152
	2,5-Dihydroxybenzoic acid/α-cyano-4-hydroxycinnamic acid	DHB/CHCA	Positive	Tryptic digest of β-casein	587
	3-Hydroxypicolinic acid and α-cyano-4-hydroxycinnamic acid	3-HPA/CHCA	Dual-Polarity	ERK^{WT}, ERK^{pT}, ERK^{pTpY}, $Fus3^{WT}$, $Fus3^{pY}$ and $Fus3^{pTpY}$; in-solution tryptic digest of the crude casein extracted from commercially available low fat milk sample	332
	2, 6-Dihydroxyacetophenone and diammonium hydrogen citrate	DHAP/DAHC	Dual-Polarity	The tryptic digests of α-casein and β-casein, human histone H1 treated with Cyclin-Dependent Kinase-1 (CDK1)	588

Targets	Matrix names	Acronyms	Ionization mode	Analyte entities	Ref.
	3-Aminoquinoline and α-cyano-4-hydroxycinnamic acid	3-AQ/CHCA	Dual-Polarity	β-casein 33-48, β-casein 1-25, ovalbumin 341-360, ovalbumin 60-85, kinase domain of insulin receptor-3 (IRK-3), kinase domain of insulin receptor-1 (IRK-1)	589
Hydrophobic peptides	Addition of o-alkylated dihydroxybenzoic acid to α-cyano-4-hydroxycinnamic acid	Addition of ADHB to CHCA	Positive	Temporin A amide, NF-kB inhibitor SN50, OVA-BIP hybrid peptide, melittin honey bee, β-amyloid 22-42, MPGΔNLS, β-amyloid 1-11, β-amyloid 1-28, GPHRSTPESRAAV, β-conglycinin hydrolysate 165-178 FAS inhibitor thioesterase antagonist soy, humanin, [Gly14]-humanin, and β-amyloid 1-42, ACTH 18-39, and MassPREP phosphorylase b digestion	360
	1-(2,4,6-Trihydroxyphenyl)octan-1-one (alkylated trihydroxyacetophenone	ATHAP	Positive	Tryptic digests of bacteriorhodopsin (BR), human cadhelin 1 (CDH1), human fibroblast growth factor receptor 4 (FGFR4), human epithelial cell adhesion molecule (EPCAM), human epidermal growth factor receptor type 2 (HER2)	590
	1-Aminopyrene	1-AP	Positive	Bradykinin fragment 1-7, angiotensin II human, valinomycin, gramicidin from *Bacillus aneurinolyticus*	274
	1-AP-derived group of uniform materials based on organic salts:[1-AP][chloride], [1-AP][ascorbate], [1-AP][bis(trifluoromethane)sulfonimide]	GUMBOS:[1-AP][Cl], [1-AP][Asc], [1-AP][NTf_2]	Positive		
Disulfide-linked proteins/peptides	1,5-Diaminonaphthalene	DAN	Positive	Human urotensin II, human guanylin, and bovine insulin	28
	Thiosalicylic acid	TSA	Positive	Substance P, [Arg^8]-vasopressin, α-conotoxin SI, HSA31-39	242

Supporting Information Table S9. Representative MALDI matrices for metabolomic analysis.

Targets	Matrix names	Acronyms	Ionization mode	Analyte entities	Ref.
Untargeted metabolites	2,5-Dihydroxybenzoic acid	DHB	Positive	Quaternary ammonium salts, steroids, nucleosides, purines, pyrimidines, amino acids, choline, opioids, antibiotics, prostaglandins, porphyrin macrocyclic metal complexes, and phthalocyanine IX	591
	α-Cyano-4-hydroxycinnamic acid	CHCA			
	9-Aminoacridine	9-AA	Negative	Phenols, carboxylic acids, sulfonates, aldehydes, plant hormones, and bile acids	17,18
	1,8-Bis(dimethylamino)naphthalene/9-aminoacridine	DMAN/9-AA	Negative	Bacterial cell membrane components	340
	4-Amino-2-methylquinoline		Negative	Amino acids, organic acids, and nucleotide phosphates	592
	4-Dimethylaminobenzaldehyde	DMABA	Negative	Low molecular weight carboxyl-containing compounds, substituted phenols, and mixtures of naphthenic acids	593
	Norharmane	NRM	Dual-Polarity	Bacterial lipid A (endotoxin)	74
	1,5-Diaminonaphthalene	DAN	Negative	Phospholipids and low molecular weight metabolites in maize leaves	29
	1,8-Bis(dimethylamino)naphthalene	DMAN	Negative	Carboxylic acids, fatty acids, amino acids, vitamins, and both plant and animal hormones	573
	Hydralazine	HZN	Dual-Polarity	Small molecule metabolites, lipids, and proteins	198
	4-Hydroxy-3-nitrobenzonitrile	HNBA	Positive	Organic drugs, peptides, proteins, mouse brain tissue, and bacterial	179
	N-phenyl-2-naphthylamine	P2NA	Negative	Small-molecule metabolites including free fatty acids, amino acids, peptides, antioxidants, and phospholipids	187
	2,5-Dihydroxybenzoic acid-graphene oxide	DHB-GO	Positive	Statin dry drop	594
	2,4,6-trihydroxyacetophenone-graphene oxide	THAP-GO	Positive	Lipid	594
	3,4-Dimethoxycinnamic Acid	DMCA	Positive	Endogenous low molecular weight compounds	130
	4-Nitrocatechol	4-NC	Positive	Low-molecular-weight compounds	183
	2-Hydroxy-5-nitro-3-(trifluoromethyl)pyridine	HNTP	Negative	Tissue metabolites	175
	4-Aminoazobenzene	AAB	Negative	Tissue metabolites	214
Neurotransmitters	2,4-Diphenyl-pyranylium tetrafluoroborate	DPP-TFB	Positive	Tyrosine, tryptamine, tyramine, phenylethylamine, dopamine	595

Targets	Matrix names	Acronyms	Ionization mode	Analyte entities	Ref.
	2-Fluoro-1-methyl pyridinium	FMP	Positive	Low-abundance NTs (such as dopamine, serotonin, etc.) and their upstream and downstream molecules	232
	5-(3-Trifluoromethylbenzylidene)thiazolidine-2,4-dione	3-CF_3-BTD	Positive	Biogenic monoamine transmitters *i.e.*, dopamine, serotonin, histamine, and adrenaline	278
	2,4-Diphenyl-pyranylium tetrafluoroborate/2,5-dihydroxybenzoic acid	DPP-TFB/DHB	Positive	Neurotransmitters in crustacean brain	596
	Gold nanoparticles	AuNPs	Dual-Polarity	Metabolites from mouse brain tissue, including neurotransmitters, fatty acids and nucleobases	597
	Citrate-capped gold nanoparticles	AuNPs-CBS	Positive	Neurotransmitters in serum and homogenized tissue, including, acetylcholine, dopamine, epinephrine, glutamine, 4-aminobutyric acid, norepinephrine, octopamine, and serotonin	378
	Zinc oxide nanoparticles	ZnO NPs	Positive	Neurotransmitters and small molecules in brain tissues	598
Vitamins	α-Cyano-4-hydroxycinnamic acid	CHCA	Dual-Polarity	Vitamins A1, B6, and C in dried persimmons (*Diospyros kaki*)	13
			Positive	25-Hydroxyvitamin D_3 (25(OH)D_3)	599
	Porphyrin compounds		Dual-Polarity	Water-soluble vitamins (B1, B2, B6, B12, and C)	600
	2,3,4,5-Tetrakis(3′,4′-dihydroxylphenyl) thiophene	DHPT	Positive	Vitamin B (B1, B2, B3, B6)	141
	Parylene matrix chip		Positive	25-Hydroxyvitamin D_3 (25(OH)D_3) in serum	601
Alkaloids	α-Cyano-4-hydroxycinnamic acid	CHCA	Positive	Alkaloid components in *Aconitum Carmichaeli* Debx. (Fuzi in Chinese)	602
	2,5-Dihydroxybenzoic acid	DHB	Positive	Alkaloids on the surface of *Erythroxylum coca* leaves	64
				Toxic glycoalkaloids (α-solanine, α-chaconine, dehydrochaconine, and dehydrobatatinine) in potato tuber tissue	603
	3-[5′-(Methylthio)-2,2′-bithiophen-5-ylthio]propanenitrile	MT3P	Dual-Polarity	Alkaloids	140

Targets	Matrix names	Acronyms	Ionization mode	Analyte entities	Ref.
	3-(5'-Pentafluorophenylmethylsulfanyl-[2,2']bithiophenyl-5-ylsulfanyl)propionitrile	PFPT3P	Positive	Alkaloids from highly complex samples such as the crude extract of Cholchicum autumnale (Colchicaceae), strychnine-spiked human plasma, as well as RYTMOPASC ® solution	604
	2,3,4,5-Tetrakis(3′,4′-dihydroxylphenyl) thiophene	DHPT	Positive	The metabolites including creatinine, glycine, alloxan, allantoin, and 3-hydroxyhippuric acid in human urine	141
	7-Mercapto-4-methylcoumarin	MMA	Positive	Arecoline and arecaidine in areca nut and arecoline in human plasma	162
	3,4-Dimethoxycinnamic acid	DMCA	Positive	10 Physiologically active alkaloids in situ (arecoline, arecaidine, caffeine, cotinine, guvacine, guvacoline, hordenine, sophoridine, trigonelline, and vicine) in areca fruits at three different developmental stages	605
	Titanium dioxide nanowire substrate	TiO_2 nanowire substrate	Positive	Vinca alkaloids in the petal of Catharanthus roseus	416
	Gold nanoparticles	AuNPs	Positive	Amino acids, amide alkaloids, imidazolium alkaloids and saccharide in the upper, middle, and lower parts of Lepidium meyenii Walp (Maca) root tissue	606
Phytohormones	α-Cyano-4-hydroxycinnamic acid	CHCA	Positive	Cytokinin and abscisic acid on the roots of rice (*Oryza sativa*)	607
	Ferrum nanoparticles	Fe-NPs	Positive	Multiple plant hormones in roots of rice (*Oryza sativa*)	608
	p-Nitroaniline	PNA	Dual-Polarity	Multiple lipids and phytohormones	181
	2,4-Dihydroxy-5-nitrobenzoic acid	DHNBA	Positive	Phytohormones in plant tissues	145
Flavonoids	2,5-Dihydroxybenzoic acid	DHB	Positive	Quercetin, myricetin, luteolin and kaempferol	3
				Isoflavones from soy samples	609
	2,4,6-Trihydroxyacetophenone	THAP	Dual-Polarity	Flavonol glycosides in food crude extracts	26
	9-Aminoacridine	9-AA	Negative	Flavones in *Scutellaria baicalensis* Georgi	19
	2,6-Dihydroxyacetophenone	DHAP	Negative	Caffeoylquinic acids and flavonoids in *Arctium lappa* L. root	610
	Metal-organic framework@hydrogen-bond framework	MOF@HOF composite material	Positive	Falvonoids	543

Targets	Matrix names	Acronyms	Ionization mode	Analyte entities	Ref.
	Boronic acid-functionalized magnetic multi-walled carbon nanotubes with flexible branched polymers	Fe_3O_4@MWCNTs@ε-PL@BA	Dual-Polarity	Cis-diol-flavonoid compounds	611
	Graphene oxide	GO	Dual-Polarity	Flavonoids and the derivatives of coumarin	112
	Colloidal graphite		Negative	Small molecules such as phospholipids, cerebrosides, oligosaccharides, flavonoids, and other secondary metabolites	612

Supporting Information Table S10. Representative MALDI matrices for lipidomic analysis.

Targets	Matrix names	Acronyms	Ionization mode	Analyte entities	Ref.
Untargeted lipidomics	2,5-Dihydroxybenzoic acid	DHB	Positive	Diacylglycerols, phosphatidylcholines, and (poly)phosphoinositides	613
				Phospholipids and glycolipids	614
	2,5-Dihydroxybenzoic acid-CnH2n+1	DHB-C_nH_{2n+1}(C_6H_{13}, C_9H_{19}, $C_{12}H_{25}$)	Positive	Lipids in the brain	615
	α-Cyano-4-hydroxycinnamic acid	CHCA	Positive	Phospholipids in formalin fixed rat brain sections	14
	α-Cyano-4-hydroxycinnamic acid butylamine	CHCAB	Positive	Phospholipids in mouse liver and cerebellum tissue sections	304
	9-Aminoacridine	9-AA	Negative	LMW organic acids including aliphatic (from acetic to palmitic acid), aromatic acids, phytohormones (*e.g.*, Jasmonic and salicylic acids), and amino acids	18
				Cellular glycerophospholipids	20
				Rat brain lipid	21
	N-(1-naphthyl) ethylenediamine dihydrochloride and 9-aminoacridine	NEDC/9-AA	Dual-Polarity	All the major classes of phospholipids and sulfatide from mouse brain slides	341
	1,8-Bis(dimethylamino)naphthalene	DMAN	Negative	LMW analytes (fatty acids, amino acids, fatty acid-amino acid conjugates, plant and animal hormones, vitamins, and short peptides)	185
	1,8-Bis(dimethylamino)naphthalene and 9-aminoacridine	DMAN/9-AA	Negative	Lipid fingerprinting of intact bacteria	340
	1,5-Diaminonaphthalene	DAN	Dual-Polarity	Plasma phospholipid content between atherosclerotic and healthy mice	616
				Lipidome	30
				Small molecules in tissues following focal cerebral ischemia	275
	p-Nitroaniline	PNA	Dual-Polarity	Various phospholipid classes	617
			Positive	Lipids	618
	2-Mercaptobenzothiazole	2-MBT	Dual-Polarity	Lipids on brain and liver tissue	62
	Dithranol	DT	Positive	Lipids in rat liver and bovine calf lens	36
	Quercetin		Positive	Lipids in rat brain	37
	1,6-Diphenyl-1,3,5-hexatriene	DPH	Negative	Fatty acids, phospholipids, and sulfatides in brain tissues	211

Targets	Matrix names	Acronyms	Ionization mode	Analyte entities	Ref.
	2,3-Dicyanohydroquinone	DCH	Positive	Lipids in biological tissues	206
	Michler's ethylketone	MEK	Negative	Lipid MALDI tissue imaging	178
	3-Aminophthalhydrazide	3-APH; luminol	Dual-Polarity	Fatty acids, glycerolipids, glycerophospholipids, sphingolipids, and saccharolipids in mouse brain subjected to middle cerebral artery occlusion (MCAO)	197
Fatty acids	Meso-tetrakis porphyrin	F20TPP	Positive	FFAs in rat plasma	619
	1,14-Diaza[5]helicene		Negative	FAs and organic acids in a wide range of samples	188
	Ammonia-treated N-(1-naphthyl) ethylenediamine dihydrochloride	ATNEDC	Negative	Serum FFAs	620
	N1,N4-dihbenzylidenebene-1,4-diamine	DBDA	Negative	Serum FFAs; FAs and sulfatides in brain tissue sections	213,621
	Silver nanoparticles	AgNPs	Negative	FAs in mouse retinal sections; surface FAs of Arabidopsis thaliana	60,622
	Cyanographene	G-CN	Dual-Polarity	Oil binders and FAs in artworks	623
	Graphene or graphene oxide	G, GO	Positive	Long-chain FAs	624
	Chemically modified nanometer-scale silicon		Negative	Substituted sulfonic acids and FAs from milk and tick nymph samples	625
Glycerolipids	Lithium 2,5-dihydroxybenzoate	LiDHB	Positive	Hydrocarbons (C24–C40), diverse lipids (triglycerides, diglycerides, wax esters from leaves) and saturated polymers	270
	Cyanonitrophenyl dienoic acid	CNDA	Positive	Neutral lipids such as DAGs and TAGs	263
	Aggregated graphene oxide	AGO	Positive	TAGs in complex biological samples	455
	Citrate-capped gold nanoparticles	AuNPs-CBS	Positive	TAGs from crude lipid mixtures	377
				TAGs from thin tissue sections	626
Glycerophospholipids	2-(2-Aminoethyloamino)-5-nitropyridine	AAN	Negative	Phospholipids in simple mixtures and in a crude methanolic soybean extract	171
	5-Chloro-2-mercaptobenzothiazole	CMBT	Dual-Polarity	Phospholipids in mouse kidney	137
	2',4',6'-Trihydroxyacetophenone	THAP	Positive	Neutral storage lipids (triacylglycerols), polar membrane lipids (glycerophospho- and sphingolipids), and glycosphingolipids	135
	p-Nitroaniline with the protonating agent butyric acid	PNA/butyric acid	Positive	Phosphorylated lipids in biological fluids	317
	Ionic-liquid matricess of α-cyano-4-hydroxycinnamic acid	ILMs of CHCA	Dual-Polarity	Phospholipids	296

Targets	Matrix names	Acronyms	Ionization mode	Analyte entities	Ref.
	2,5-Dihydroxybenzoic acid and α-cyano-4-hydroxycinnamic acid with a mixture of trifluoroacetic acid and piperidine	DHB/CHCA/TFA or piperidine	Dual-Polarity	Phospholipids	335
	Cyno-4-hydroxycinnamic acid butylamine	CHCAB	Positive	Phospholipids in soybean	627
	Silicon nanopost arrays	NAPA	Dual-Polarity	Lipids, (+) phosphatidylcholines and phosphatidic acids, (-) sulfatides and free fatty acids	503
	Nanoflakes-capped silicon nanowires	NGQD@MoS2/SiNWs	Dual-Polarity	Phospholipids on sliced non small cell lung cancer	628
	2-hydroxy octadecanoic acid-modified barium titanate nanoparticles	HOA-modified BaTiO3 NPs	Positive	Phospholipids	426
	2,6-Dihydroxyacetophenone	DHAP	Negative	Glycerophospholipids and sulfatides in brain tissue; cardiolipin from rat organs section	629,630
	Norharmane	NRM	Negative	Cardiolipins in prokaryotic and eukaryotic samples	631
Sphingolipids	2-(Methylamino)benzoic acid	COOH-NHMe (IV)	Dual-Polarity	Phospholipids in mouse brain sections	272
	6-Glycosylaminoquinoline	6-GAQ	Negative	Hydrophilic lipids in the mouse kidney	632
	Polydopamine-capped AgNPs	AgNPs@PDA	Dual-Polarity	Glycerophospholipids and sphingolipids in impact-induced injured brain	633
	Porous graphitic carbon	PGC	Positive	Glycerolipids and sphingolipids in brain tissue	482
Sterol lipids	6,7-Dihydroxycoumarin-3-carboxylic acid	DCA	Positive	Sterols in yeast cells	167
	1,8-Bis(Trispyrrolidinophosphazenyl)PS	TPPN	Negative	Cholesterol	190
	N-methylpyridinium-2-carboxaldehyde	MP2CA	Positive	Cholesterol and other sterols	634
	Modified polyvinylidene fluoride membrane	m-PVDF-m	Dual-Polarity	TAGs and cholesteryl esters on mouse kidney section	635
	Three-dimensional mesoporous graphene and zirconium-based metal-organic frameworks	MG@UiO-66	Positive	Steroids	516
	Sodium-doped gold	Au-CBS	Positive	Intact cholesterol, TAG and cholesterol esters (CE) from a rabbit adrenal gland section	626
	Cation-enhanced nanostructure-initiator mass spectrometry	cation-enhanced NIMS	Positive	Carbohydrates or steroids in human serum, a Gerbera jamesonii flower stem and a mouse brain	487

Targets	Matrix names	Acronyms	Ionization mode	Analyte entities	Ref.
Lipid oxidation products	6-Aza-2-thiothymine	ATT	Dual-Polarity	Oxidized phospholipids (OxPLs)	25
	2,4-Dinitrophenylhydrazine	DNPH	Positive	Lipid oxidation products, gaseous aldehydes and ketones	68
				Gaseous aldehydes	69
Carbon-carbon double bond (C=C) positional isomers	Benzophenone	BPh	Positive	C═C double-bond (DB) positions in isomeric phospholipids	240
	2-Benzoylpyridine	BzPy	Dual-Polarity	Localization of C═C double bonds (DBs) in lipids	241
	Metal oxide laser ionization mass spectrometry imaging technique with off-line lipid derivatization by ozone	CeO_2 and TiO_2 nanopowders	Negative	Fatty acids and their carbon-carbon double bond (C═C) positional isomers	636

Supporting Information Table S11. Representative MALDI matrices for glycomic analysis.

Targets	Matrix names	Acronyms	Ionization mode	Analyte entities	Ref.
Monosaccharides	N-(1-naphthyl)ethylenediamine dihydrochloride	NEDC	Negative	Glucose in rat brain microdialysates	191
	1-Naphthylhydrazine hydrochloride	NHHC	Negative	Quantification of glucose and homogentisic acid in real samples	194
	Maltose-functionalized metal-organic framework	MIL-101-maltose	Positive	Eight saccharide standards from monosaccharide to octasaccharide including glucose, sucrose, maltotriose, maltotetraose, maltopentaose, α-CD, β-CD and γ-CD and serum glucose	526
	Washed carbon soot nanoparticles	WCS NPs	Positive	Quantitative urinary glucose determination	480
	O-P,N-doped carbon/graphene	O-P, N-C/G	Positive	Fructose and glucose in human serum and soft drinks	451
Oligosaccharides	2',4',6'-trihydroxyacetophenone	THAP	Positive	Fructooligosaccharides in red onions, shallots, and elephant garlic	637
	Hydrazinonicotinic acid	HYNIC	Positive	Oligosaccharides from human serum	170
	Isoliquiritigenin	ISL	Positive	Neutral oligosaccharides	158
	1,8-bis(tripyrrolidinylphosphazenyl)naphthalene	TPPN	Negative	Neutral saccharides, cyclodextrins, and saccharide alditols	638
	2,5-dihydroxybenzoic acid/N-methylaniline	DHB/*N*-MA	Positive	Oligosaccharide, fructo-oligosaccharide mixtures extracted and identified from rice noodles	288
	Dihydroxybenzoic acid-functionalized magnetic nanoparticles	DHB@MNPs	Positive	Oligosaccharides from human milk	639
	3-aminoquinoline	3-AQ	Dual-Polarity	Oligosaccharides	46
	Aminopyrazine/2,5-dihydroxybenzoic acid	AP/DHB	Positive	Oligosaccharides, the mixture of glycans and deglycosylated tryptic digest of glycoproteins	84
	Charcoal		Positive	Sucrose	640
	Palladium nanoparticles decorated thiol-functionalized metal organic framework nanocomposite	UiO-66$(SH)_2$@Pd NPs	Positive	Oligosaccharide isomers, including disaccharides, trisaccharides, and tetrasaccharides	518
	Graphene oxide	GO	Positive	Seven disaccharide isomers (gentiobiose, isomaltose, melibiose, lactose, maltose, cellobiose, and sucrose), and maltose and sucrose in four different honey samples	113

Targets	Matrix names	Acronyms	Ionization mode	Analyte entities	Ref.
Polysaccharides	5-Amino-2-mercapto-1,3,4-thiadiazole	AMT	Positive	Neutral carbohydrates of molecular weights up to approximately 5000 Da	139
	2',4',6'-trihydroxyacetophenone	THAP	Positive	Large polysaccharides, dextrans, glycoproteins and polysialic acids<br>α and β-glucans form commercial mushroom extracts	24<br>641
	2-Hydrazinequinoline	2-HQ	Dual-Polarity	LMW polysaccharides	642
	3-Aminoquinoline/α-cyano-4-hydroxycinnamic acid	3-AQ/CHCA	Positive	Various oligosaccharides and polysaccharides, such as (A)XOS, malto-oligosaccharides and cyclodextrins	643
Glycoproteins	3-Amino-4-hydroxybenzoic acid	AHB	Positive	Oligosaccharide mixtures released from glycoproteins	149
	2,5-Dihydroxybenzoic acid	DHB	Positive	*N*-glycans and extracellular matrix peptides on formalin-fixed paraffin-embedded tissues	4
	2,5-Dihydroxybenzoic acid/aniline/sodium	ANI/DHB/Na	Positive	The structural pattern of glycan on glycoproteins	644
	1,5-Diaminonaphthalene/2,5-dihydroxybenzoic acid/Na	DAN/DHB/Na	Positive	Glycoforms in the femtomolar range of intact glycoproteins	644
	3-Hydrazinobenzoic acid plus DHB and quinoline-3-carbohydrazide plus DHB	DHB/3HBA and DHB/Q3CH	Dual-Polarity	*N*-glycans of serum from hepatocellular carcinoma (HCC) patients	645
	2-(4-Hydroxyphenylazo)benzoic acid	HABA	Positive	Peptides, proteins, and glycoproteins up to approximately 250 kDa	154
	α-Cyano-4-hydroxycinnamic acid	CHCA	Positive	*N*-glycans and lipids from tissues	15
	α-Cyano-3-amino-cinnamic acid/α-cyano-4-hydroxycinnamic acid	3-CACA/CHCA	Positive	Plant *N*-glycan	223
	2-Hydrazinoterephthalic acid and sinapic acid/2-hydrazinoterephthalic acid	2-HTA and SA/2-HTA	Negative	*N*-glycans in peach allergy	230
	1,1,3,3-Tetramethylguanidine salt of 2,4,6-trihydroxyacetophenone	GTHAP	Positive	Glycopeptides and glycans	315
	Graphene nanosheets and **c**arbon nanoparticles	GNs and CNPs	Dual-Polarity	Glycan profiling of three diverse glycoproteins and human blood serum	481
	Cysteine-functionalized metal-organic framework	MIL-101(NH_2)@Au-Cys	Positive	Saccharides, lipids, small peptides, polymers, drugs and amino acids	525
	Sodium-doped 3-amino-4-hydroxybenzoic acid	AHB/Na	Positive	*O*-glycan fragments generated by the ISD of glycopeptides and glycoproteins containing *O*-glycans	150

Targets	Matrix names	Acronyms	Ionization mode	Analyte entities	Ref.
	O-Benzylhydroxylamine/2,5-dihydroxybenzoic acid/Na	BOA/DHB/Na	Positive	*O*-glycan from porcine stomach mucin	289
	2,4,6-Trihydroxy acetophenone–ammonium citrate	THAP-ammonium citrate	Positive	*O*-glycopeptides derived from human serum IgA1	646
	4-Chloro-α-cyanocinnamic acid	Cl-CCA	Dual-Polarity	Labile sialylated tryptic *N*-glycopeptides and released *N*- and *O*-glycans	259
Proteoglycans	2,5-Dihydroxybenzoic acid	DHB	Positive	Sulphated oligosaccharides of the chondroitin sulphate	647
	Coumarin 120 and coumarin 120/6-aza-2-thiothymine	Coumarin 120 and coumarin 120/ATT	Dual-Polarity	Sulfated disaccharides to tetrasaccharides, including those containing sialic acid	648
	9H-pyrido[3,4-b]indole	Nor-harmane	Dual-Polarity	Sulfated oligosaccharides (λ-carrageenans)	71
	Z-Sinapinic acid	Z-SA	Negative	Neutral/sulfated carbohydrates	8
	1-Methylimidazolium α-cyano-4-hydroxycinnamate<br>butylammonium 2,5-dihydroxybenzoate	ImCHCA<br>DHBB	Dual-Polarity	The sodium salts of a disaccharide, sucrose octasulfate (SOS), and an octasulfated pentasaccharide (Arixtra)	299
	Bis-1,1,3,3-tetramethylguanidinium α-cyano-4-hydroxycinnamate	G_2CHCA	Positive	Purified single-component samples and mixtures of several dermatan sulfate (DS) and chondroitin sulfate (CS) oligosaccharides	310
	Bis-1,1,3,3-tetramethylguanidinium p-coumaric acid	G_3CA	Dual-Polarity	Sulfated/sialylated/neutral oligosaccharides and glycopeptides	311
	2-(4-Hydroxyphenylazo)benzoic acid/1,1,3,3-tetramethylguanidine<br>2-(4-Hydroxyphenylazo)benzoic acid/spermine	HABA/TMG<br>HABA/SPM	Negative	Heparin (HP) and heparan sulfate (HS) oligosaccharides	314
	α-Cyano-4-hydroxycinnamate/norharmane	CHCA·nHo	Negative	Sulfated oligosaccharides such as neocarratetraose-41,43-disulfate disodium salt	312
	Water ice		Negative	Enzymatically treated glycosaminoglycan samples	649
Glycolipids	2,5-Dihydroxybenzoic acid	DHB	Positive	Glycolipids	650-652
	6-Aza-2-thiothymine	ATT	Positive	Underivatized glycans, oligosaccharide derivatives, glycopeptides, and glycolipids	45

Targets	Matrix names	Acronyms	Ionization mode	Analyte entities	Ref.
	Chloro-2-mercaptobenzothiazole	CMBT	Dual-Polarity	Peptides and low-mass proteins, oligosaccharides, glycolipids, and muropeptides derived from peptidoglycans	136
	α-Cyano-4-hydroxycinnamic acid	CHCA	Positive	Neutral glycosphingolipids from the silkworm *Bombyx moria*	653
	1,5-Diaminonaphthalene	DAN	Positive	Gangliosides in rat brain tissue	31
Glycosides	2′,4′,6′-Trihydroxyacetophenone	THAP	Dual-Polarity	Flavonol glycosides in food samples	26
			Positive	Flavonol glycosides in almond seedcoats	654
	2,5-Dihydroxybenzoic acid	DHB	Positive	Flavonoid glycosides and biflavonoids in *Ginkgo biloba* L.	655
				Plant defensive cardiac glycosides in *Asclepias curassavica*	656
				Anthocyanin species in rabbiteye blueberry *Vaccinium ashei*	657
				Saponin fingerprint profile of leaves and stems in commercial yerba mate (*Ilex paraguariensis*)	658
	α-Cyano-4-hydroxycinnamic acid	CHCA	Negative	Cucumarioside A_2-2 in mouse spleen	659

Supporting Information Table S12. Representative MALDI matrices for nucleic acid analysis.

Matrix names	Acronyms	Ionization mode	Analyte entities	Ref.
3-Hydroxypicolinic acid	3-HPA	Negative	Oligonucleotides	33<br>5
		Dual-Polarity	Underivatized single-stranded DNA oligomers	32
		Negative	500-nucleotide DNA	660
2,5-Dihydroxybenzoic acid	DHB	Negative	Oligonucleotides	5
Ferulic acid	FA	Negative	Oligonucleotides	5
			Fluorophore-labeled oligonucleotides	124
5-Methoxysalicylic acid/spermine	5-MSA/SPM	Negative	Oligonucleotides	153
3,4-Diaminobenzophenone	DABP	Negative	Oligonucleotides	177<br>661
1,5-Diaminonapthalene	DAN	Negative	Oligonucleotides	662
6-Thioguanine	TG	Negative	Oligonucleotides	212
2-Amino-3-nitropyridine	ANP	Negative	Oligonucleotides	173
2-Amino-5-nitropyridine/ammonium fluoride	ANP/NH_4F	Negative	Oligonucleotides	174
3-Hydroxypicolinic acid/ammonium fluoride	HPA/NH_4F	Negative	Oligonucleotides	663
Tetraamine spermine	TETA-SPM	Negative	Oligonucleotides	347
Polyamine		Negative	Oligonucleotides	346
Fucose		Negative	Oligonucleotides	349
2',4',6'-Trihydroxyacetophenone/ammonium tartrate	THAP/ammonium tartrate	Negative	Natural and modified oligonucleotides	23
Anthranilic acid	AA	Negative	Oligodeoxynucleotides (ODNs)	220
3-Hydroxypicolinic acid/pyrazinecarboxylic acid	3-HPA/PCA	Positive	Oligodeoxynucleotides	338
2,5-Dihydroxybenzoic acid/ammonium acetate	DHB/ammonium acetate	Negative	Oligodeoxynucleotides	664
Nicotinic acid	NA	Positive	Underivatized oligodeoxyribonucleotides	665
Succinic acid<br>Urea<br>Nicotinic acid		Negative<br>Negative<br>Negative	Nucleic acids	666
Picolinic acid	PA	Negative	Nucleic acids	168
Quinaldic acid	QA	Dual-Polarity	Nucleic acids	215
Glycerol		Negative	Large nucleic acids	667
3-Aminopicolinic acid	3-APA	Negative	DNA	668

Matrix names	Acronyms	Ionization mode	Analyte entities	Ref.
2',4',6'-Trihydroxyacetophenone/2',3',4'-trihydroxyacetophenone/ammonium citrate	2,4,6-THAP/2,3,4-THAP/ammonium citrate	Negative	DNA	337
3-Hydroxycoumarin	3-HC	Positive	DNA	165
3-Hydroxypicolinic acid/base	3-HPA/base	Positive	DNA oligomers	321
2,5-Dihydroxybenzoic acid/base	2,5-DHB/base	Positive		
6-Aza-2-thiothymine	ATT	Negative	Intact double-stranded DNA	41
		Positive	Specific noncovalent complexes between guanidinium derivatives and single-stranded DNA	43
Polylysine-coated diamond nanocrystals		Negative	DNA oligonucleotides	669
Various nanoscale architectures		Positive	Surface-confined DNA	670
2',4',6'-Trihydroxyacetophenone	THAP	Negative	Noncovalent RNA-peptide complexes	22
3-Hydroxycholanic acid/sugar dopants	3-HPA/sugar dopants	Positive	The smallest nucleotide change (A:T)	671

Supporting Information Table S13. Representative MALDI matrices for MALDI-MS quantitative analysis.

Matrix class	Matrix names	Acronyms	Analyte	Ionization mode	Performance and notes	Ref.
Organic framework matrix (MOF)	Titanium based MOF nanosheets	Ti-based MOF nanosheets	Small molecules	Negative	Excellent ionization efficiency, less background interference, significant stability, and high salt resistance for quantitative analysis.	672
Organic framework matrix (MOF)	Pd nanoparticles decorated thiol-functionalized MOF	UiO-66-$(SH)_2$@Pd NP	Carbohydrates (*e.g.*, oligosaccharide isomer)	Positive	High ionization efficiency, high sensitivity, high oligosaccharide isomer discrimination performance, and good for quantitative analysis.	518
Organic framework matrix (MOF)	MOF-Derived erythrocyte-like CuO/Cu_2O@ZnO-CN nanomaterials	CuO/Cu_2O@ZnO-CN	Carbohydrates (*e.g.*, saccharides and glucose)	Positive	Excellent sensitivity, ionization efficiency and specificity, low background interference, high salt and protein tolerance and, good stability and reproducibility for quantitative analysis.	673
Organic framework matrix (COF)	Fluoro-functionalized ionic covalent organic framework	F-iCOF	Perfluorinated sulfonate	Negative	High ionization efficiency, without ion interferences in LMW region, high selectivity, and high sensitivity for quantitative analysis.	674
Organic framework matrix (COF)	1,3,5-Tris(4-aminophenyl)benzene-2,5-dimethoxyterephthalaldehyde-covalent organic frameworks	TAPB-DMTP-COF	Small molecules (*e.g.*, benzophenone derivatives)	Negative	High ionization efficiency, without ion interferences in LMW region, high selectivity, and high sensitivity for quantitative analysis.	532,675
Organic framework matrix (COF)	Carboxyl-functionalized COFs	COF_{HD}	Cationic dyes	Positive	High ionization efficiency, without ion interferences in LMW region, high selectivity, and high sensitivity for quantitative analysis.	676
Carbon-based nanomaterial matrix	Graphene	G	Small molecules	Positive	High sensitivity, high reproducibility, high salt tolerance, low-background ion interference, and good for quantitative analysis.	109
Carbon-based nanomaterial matrix	Hybrid bismuth oxide-graphene oxide	Bi_2O_3@GO	Small molecules (*e.g.*, glucose, maltose, raffinose, fatty acids, ascorbic acid, gibberellic acid, sulfaquinoxaline, bisphenol A, etc.)	Negative	Low background interferences, high sensitivity and stability, and good repeatability for quantitative analysis.	460

Matrix class	Matrix names	Acronyms	Analyte	Ionization mode	Performance and notes	Ref.
Metallic nanostructure matrix	Ferroferric oxide nanoparticles	Fe_3O_4 NPs	Small molecules (*e.g.*, LMW adulterations: enrofloxacin, acetaminophen, ribavirin, lincomycin hydrochloride, moroxydine hydrochloride, antipyrine, glucose, etc.)	Positive	High sensitivity, high reproducibility, low-background ion interference, and good for quantitative analysis.	677,678
Metallic nanostructure matrix	Silver nanoparticles	AgNPs	Cholesterol	Positive	High sensitivity, high reproducibility, low-background ion interference, and good for quantitative analysis.	679
Metallic nanostructure matrix	Gold nanoparticles	AuNPs	Small molecules (*e.g.*, glutathione)	Positive	High sensitivity, high reproducibility, low-background ion interference, and good for quantitative analysis.	680,681
Metallic nanostructure matrix	Core@shell nanostars composed of star-like Au nanocores with TiO2 shells	Au@TiO_2 NSs	Small molecules	Positive	High sensitivity, high reproducibility, low-background ion interference, and good for quantitative analysis.	682
Metallic nanostructure matrix	Porous TiO2 film immobilized with gold nanoparticles	AuNPs-FPTDF	Small molecules	Dual-Polarity	High sensitivity, high reproducibility, low-background ion interference, and good for quantitative analysis.	683
Quantum dot matrix	Functionalized quantum dots with dopamine dithiocarbamate	QDs-DDTC	Tryptic digest proteins (*e.g.*, cytochrome c, lysozyme, BSA, etc.)	Positive	High ionization efficiency, high sensitivity, background-free detection, good reproducibility, and good linearity for quantitative analysis.	684
			Small molecules (*e.g.*, efavirenz, tobramycin, aspartame, etc.)	Positive	High ionization efficiency, high sensitivity, background-free detection, good reproducibility, and good linearity for quantitative analysis.	684
Organic-inorganic hybrid matrix	2,5-Dihydroxybenzoic acid conjugated magnetic nanoparticles	DHB@MNP	Small molecules (*e.g.*, morphine, 7-aminoflunitrazepam, etc.)	Positive	High ionization efficiency, high sensitivity, background-free detection, good reproducibility, and good linearity for quantitative analysis.	685

Matrix class	Matrix names	Acronyms	Analyte	Ionization mode	Performance and notes	Ref.
Reactive matrix	2-Hydrazinoquinoline	2-HQ	Carbohydrates	Dual-Polarity	The sensitivity for analyzing glycans has been greatly improved vs. 3-AQ and DHB. The good reproducibility of 2-HQ allowed for quantitative analysis of neutral, acidic, and LMW carbohydrates.	642
Reactive matrix	4-Hydrazinoquinazoline	4-HQ	Cholesterol	Positive	High sensitivity, high specificity, and good reproducibility for quantitative analysis.	686
			Carbohydrates (*e.g.*, glycans)	Dual-Polarity	High sensitivity, high specificity, and good reproducibility for quantitative analysis.	199
Reactive matrix	3-Aminoquinoline	3-AQ	Carbohydrates (*e.g.*, oligosaccharides)	Dual-Polarity	Good performance for oligosaccharide derivatized quantitatively and reproducibly.	46
Reactive matrix	Aminopyrazine	AP	Carbohydrates (*e.g.*, oligosaccharides)	Positive	Good performance for oligosaccharide derivatized quantitatively and reproducibly.	84
Reactive matrix	2-Phenyl-3-(*p*-aminophenyl) acrylonitrile	PAPAN	Carbohydrates (*e.g.*, glycans)	Positive	The detection sensitivity for glycans was improved 100-fold vs. DHB and the peptide ionization can be significantly suppressed. It has potential for glycans quantitative analysis.	221
Reactive matrix	2-Hydrazinoterephthalic acid	2-HTA	Carbohydrates (*e.g.*, glycans)	Negative	Homogeneous co-crystallization and high ionization efficiency for glycans quantitative analysis	230
Reactive matrix	*α*-Cyano-3-aminocinnamic acid	3-CACA	Carbohydrates (*e.g.*, glycans)	Positive	The use of 3-CACA combined with CHCA shows high homogeneous co-crystallization, high derivatization efficiency, high sensitivity, and high reproducible quantitation with good linearity.	223
Reactive matrix	4-Trimethylamino-6-(4-methoxy-1-naphthyl)-1,3,5-triazine-2-(3-aminophenylboronic acid)	TMNTA	Carbohydrate isomers (*e.g.*, glucose and fructose)	Positive	High ionization efficiency, high sensitivity, high isomer discrimination performance, low matrix interferences, and good for quantitative analysis.	239
Reactive matrix	1,5-Naphthalenediamine	DAN	Short-chain fatty acids	Positive	The use of 1,5-DAN combined with isotope-coded on-tissue derivatization is suitable for short-chain fatty acid quantitative analysis.	687

Matrix class	Matrix names	Acronyms	Analyte	Ionization mode	Performance and notes	Ref.
			Lipids (*e.g.*, phospatidylcholines)	Positive	The use of 1,5-DAN combined with the internal standard control is suitable for phospatidylcholine quantitative analysis.	688,689
Reactive matrix	2-[(2*E*)-3-(4-*tert*-butylphenyl)-2-methylprop-2-enylidene]malononitrile	DCTB	CNS drugs (*e.g.*, Xylazine)	Positive	High sensitivity and high ionization efficiency for xylazine on tissue quantitative analysis.	87
Ionic liquid matrix	3-Aminoquinoline/α-cyano-4-hydroxycinnamic acid	3-AQ/CHCA	Carbohydrates (*e.g.*, glycans)	Negative	High sensitivity with good quantitative capability for glycans	690
Ionic liquid matrix	3-Aminoquinoline/2′,4′,6′-trihydroxyacetophenone monohydrate	3-AQ/THAP	Oligonucleotides (*e.g.*, miRNA)	Negative	High sensitivity and high accuracy for miRNA quantitative detection.	691
Ionic liquid matrix	3-Aminoquinoline/ammonium formate	3-AQ/ $HCOONH_4$	Gangliosides	Negative	High sensitivity and high ionization efficiency for quantitative analysis	692
Ionic liquid matrix	DHB/N-methylaniline	DHB/*N*-MA	Carbohydrates	Positive	High sensitivity and high ionization efficiency for carbohydrate quantitative analysis.	288,677
Ionic liquid matrix	DHB/*N*-ethylaniline	DHB/*N*-EA	Carbohydrates	Positive	High sensitivity and high ionization efficiency for carbohydrate quantitative analysis.	288
Ionic liquid matrix	DHB/*N,N*-dimethylaniline	DHB/DMA	Carbohydrates (*e.g.*, glycans)	Positive	High sensitivity and precision for glycan quantitative analysis.	693
Ionic liquid matrix	DHB/pyridine	DHB/Pyr	Amino acids	Dual-Polarity	High homogeneous co-crystallization, high sensitivity, and high reproducible quantitation with good linearity.	282
Ionic liquid matrix	α-Cyano-4-hydroxycinnamic acid/2-aminopentane	CHCA/AP	Metabolites (*e.g.*, *N*-acyl homoserine lactones)	Positive	The use of CHCA/AP combined with the isotope labeling technology shows high sensitivity and ionization efficiency for quantitative analysis.	694
Ionic liquid matrix	α-Cyano-4-hydroxycinnamic acid/aniline	CHCA/ANI	Proteins	Positive	The use of CHCA/ANI combined with immunosorbent assay-MS (ISA-MS) method shows high sensitivity and ionization efficiency for quantitative analysis.	695

Matrix class	Matrix names	Acronyms	Analyte	Ionization mode	Performance and notes	Ref.
Ionic liquid matrix	1,5-Diaminonaphthalene/ammonium fluoride	DAN/ NH_4F	Metabolites and lipids	Negative	The use of 1,5-DAN/ NH_4F combined with isotopically labeled standard control shows high sensitivity and ionization efficiency for quantitative analysis.	696
Matrix with dopant	2,4,6-Trihydroxyacetophenone intercalated Na^+-smectite	THAPNaSm	Monosaccharides (*e.g.*, glucose)	Positive	High sensitivity, high reproducibility, analyte size dependent Ionization, low-background ion interference, and good for quantitative analysis.	697
Matrix with dopant	Sodium 2,5-dihydroxybenzoate	NaDHB	Lipids (*e.g.*, sterol lipids, PCs, PEs, acylglycerols, etc.)	Positive	Almost completely suppresses analyte protonation and corresponding fragmentation reactions and has potential for quantitative analysis.	698
Matrix with dopant	2,5-Dihydroxybenzoic acid/5-methoxysalicylic acid/fucose	DHB/MSA/fucose	Proteins (*e.g.*, cyclosporin A, insulin, arg-insulin, cytochrome C, etc.)	Positive	Good accuracy and linearity for quantitative analysis.	699
Matrix with dopant	Ferulic acid/fucose	FA/fucose	Proteins (*e.g.*, cyclosporin A, insulin, arg-insulin, cytochrome C, etc.)	Positive	Good accuracy and linearity for quantitative analysis.	699
Matrix with dopant	2,5-Dihydroxybenzoic acid/ammonium sulfate	DHB/AS	Hydrophilic quaternary ammonium compounds (*e.g.*, carnitine, choline, acetylcarnitine, glycerophosphocholine, etc.)	Positive	High sensitivity, high ionization efficiency, low interference of potassium, and suitable for quantitative analysis	700
Binary and hybrid-based matrix	2,5-Dihydroxybenzoic acid/2,5-dihydroxybenzohydrazide	DHB/DHBH	Carbohydrates (*e.g.*, glycans)	Positive	The use of DHBH combined with DHB significantly improves the ionization efficiency of carbohydrates and leads to a uniform co-crystallization of analytes-matrix mixtures, providing accurate quantitation for N-glycans with high derivatization efficiency and good linearity.	326

Matrix class	Matrix names	Acronyms	Analyte	Ionization mode	Performance and notes	Ref.
Binary and hybrid-based matrices	(3-Hydrazinobenzoic acid/DHB)/(quinoline-3-carbohydrazide/DHB)	(DHB/3HBA)/ (DHB/Q3CH)	Carbohydrates (*e.g.*, glycans)	Dual-Polarity	High ionization efficiency, high sensitivity, and good linearity and accuracy for glycans quantitative analysis.	645
SOM matrix	2,5-Dihydroxybenzoic acid	DHB	Metabolites	Positive	Strong background interference and ion suppression, inhomogeneous co-crystallization, and usually poor for LMW molecule quantitative analysis.	155,206
			Carbohydrates	Positive	High sensitivity. Under strict quality control (*e.g.*, isotope labeling technology control, internal standard control), it can be used for oligosaccharide (or glycan) quantitative analysis.	642,693,701-704
			Lipids (*e.g.*, sphingomyelins, glucosylceramides, phosphatidylcholines, lysophosphatidylcholines, phosphatidylinositols, etc.)	Positive	Under strict quality control (*e.g.*, chloroform-methanol (2:1 (v/v)) to prepare the matrix solution, internal standard control, polystyrene colloidal spheres assisted matrix-analyte co-crystallization, and so on), it can be used for quantitative analysis.	705-708
			Fatty alcohols (*e.g.*, cholesterol, hexadecanol, octadecanol, etc.)	Positive	The use of DHB combined with *N*-alkylpyridinium isotope quaternization is suitable for fatty alcohol quantitative analysis.	228
			Drugs	Positive	The use of DHB combined with automatic MS acquisition is better choice for drug quantitative analysis.	709-711
			Coccidiostats	Positive	Predominantly $[M+Na]^+$ ions and high sensitivity for quantitative analysis.	712
			Octreotide	Positive	High sensitivity and precision. Under strict quality control, it can be used for octreotide on tissue quantitative analysis.	713,714

Matrix class	Matrix names	Acronyms	Analyte	Ionization mode	Performance and notes	Ref.
			Peptides (*e.g.*, glycopeptides, phosphopeptides, etc.)	Positive	Under strict quality control (*e.g.*, matrix micro-spotting to improve the homogeneity, or conjunction with the isotope labeling technology), it can be used for peptide quantitative analysis.	715-718
			Cordycepin	Positive	High ionization efficiency. Under strict quality control (*e.g.*, isotope-labeled internal standard control), it can be used for cordycepin quantitative analysis.	719
			Blended oils (*e.g.*, sunflower seed oils, olive oils, canola oil, grapeseed oils, etc.)	Positive	High sensitivity and precision. Under strict quality control, it can be used for quantitative analysis.	720,721
			Cocaine	Positive	Under strict quality control (*e.g.*, internal standard control), it can be used for quantitative analysis.	722
			Carbohydrates (*e.g.*, disaccharide isomers)	Positive	Suitable for the relatively quantitative analysis of 2-pyridinecarbohydrazide derivatized disaccharides	723
			Carnitines and organic acids	Positive	The use of CHCA combined with isotopically labeled internal standards is suitable for quantitative analysis.	724
			Lipids	Dual-Polarity	High sensitivity combined with internal standard calibration for quantitative analysis.	725
SOM matrix	α-Cyano-4-hydroxycinnamic acid	CHCA	Sulfatide	Negative	Good sensitivity. Under strict quality control, it can be used for sulfatide quantitative analysis.	726
			Peptides (*e.g.*, neuropeptides, peptide cyanotoxins, hepcidin, etc.)	Positive	Under strict quality control (*e.g.*, matrix micro-spotting to improve the homogeneity, or conjunction with the isotope labeling technology, or self-aliquoting microarray plates to obtain homogeneous analyte/matrix co-crystallization, or combined with immuno-MALDI technology, or internal standard control), it can be used for peptide quantitative analysis.	715,727-737

Matrix class	Matrix names	Acronyms	Analyte	Ionization mode	Performance and notes	Ref.
			Proteins (*e.g.*, β-2-microglobulin (β_2M), advanced glycosylated end-products of β_2M, BSA, β-lactoglobulin, etc.)	Positive	Good sensitivity. Under strict quality control (*e.g.*, internal standard control, or acid hydrolysis combined with isotopically labeled standards, or microfluidics, or iTRAQ/TMT isotope labeling strategies), it can be used for quantitative analysis.	738-743
			Perfluorooctanesulfonic acid (*e.g.*, PFOS)	Negative	High sensitivity, high selectivity, and high repeatability for PFOS on tissue quantitative analysis.	744
			Peptides	Positive	Under strict quality control (*i.e.*, matrix micro-spotting to improve the homogeneity), it can be used for peptide quantitative analysis.	715
SOM matrix	Sinapinic acid	SA	Proteins (*e.g.*, HMW proteins, protein-protein interactions (PPIs), etc.)	Positive	The use of saturated SA combined with sandwich deposition (saturated SA as the top layer) and internal standard control shows high signal stability for high mass protein quantitative analysis. Combined with chemical cross-linking, above quantitative strategy can be used to evaluate the affinity of PPIs. The use of SA combined with PVDF affinity probe is suitable for protein quantitative analysis	745-747
			Glucosinolates	Negative	High ionization efficiency, high sensitivity, low matrix interferences, and combined with sublimation for matrix coating is good for quantitative analysis.	748
SOM matrix	9-Aminoacridine	9-AA	Lipids (*e.g.*, phosphatidylcholines, phosphatidylethanolamines, triglycerides, etc.)	Dual-Polarity	High ionization efficiency, high sensitivity, low matrix interferences, good reproducibility and linearity, and combined with normalization method is good for quantitative analysis.	749,750
			Free fatty acids	Negative	The use of 9-AA combined with the disposable paper-array plate shows high sensitivity, and good reproducibility, and good linearity for quantitative analysis.	751

Matrix class	Matrix names	Acronyms	Analyte	Ionization mode	Performance and notes	Ref.
SOM matrix	2,5-Dihydroxyterephthalic acid	DHT	Amino acids	Positive	Excellent performance for quantifying the amino acids.	155
SOM matrix	7-Hydroxy-4-(trifluoromethyl) coumarin	HFMC	Drugs	Positive	Good crystallization behavior, low background signals, good reproducibility, and good linearity for quantitative analysis (HFMC matrix was spotted on the strongly hydrophobic fluoropolymer-coated target plate).	752
SOM matrix	Hydrazinonicotinic acid	HYNIC	Carbohydrates (*e.g.*, oligosaccharides)	Positive	High sensitivity and selective analysis of oligosaccharides. It has good potential for quantitative analysis.	170
			Carbohydrates (*e.g.*, glucose)	Negative	High salt tolerance, without matrix interferences in LMW region, and high sensitivity for glucose quantitative analysis.	191
SOM matrix	*N*-(1-Naphthyl) ethylenediamine dihydrochloride	NEDC	Carbohydrates (*e.g.*, disaccharide isomers)	Negative	High salt tolerance, without matrix interferences in LMW region, high disaccharide isomer discrimination performance, and good for relatively quantitative analysis.	753
			N-Acetylaspartate	Negative	High salt tolerance, without matrix interferences in LMW region, and high sensitivity for quantitative analysis.	754
SOM matrix	Ammonia-treated N-(1-naphthyl) ethylenediamine dihydrochloride	ATNEDC	Fatty acids	Negative	High sensitivity, high specificity, and good reproducibility for quantitative analysis.	620
SOM matrix	1-Naphthylhydrazine hydrochloride	NHHC	Carbohydrates (*e.g.*, glucose)<br>Homogentisic acid	Negative<br>Negative	Little interference in LMW region, and high sensitivity for glucose and homogentisic acid quantitative analysis.	194
SOM matrix	2-Nitrophloroglucinol	2-NPG	Pesticides (*e.g.*, Fungicide pyrimethanil)	Positive	High sensitivity, high precision, good LOD/LOQ, low matrix effect, and high recovery for pyrimethanil quantitative analysis.	184
SOM matrix	Norharmane	NRM	Lipids	Negative	The use of NRM combined with the internal standard control shows high sensitivity, high specificity, and good reproducibility for lipid quantitative analysis.	708

Matrix class	Matrix names	Acronyms	Analyte	Ionization mode	Performance and notes	Ref.
SOM matrix	3-Hydroxypicolinic acid	3-HPA	Oligonucleotides	Dual-Polarity	The use of 3-HPA combined with ammonium citrate or polymerase chain reaction-MS (PCR-MS) shows high sensitivity, high specificity, and good reproducibility for quantitative analysis.	695,755

REFERENCES

(1) Strupat, K.; Karas, M.; Hillenkamp, F. 2,5-Dihydroxybenzoic acid: a new matrix for laser desorption—ionization mass spectrometry. *Int. J. Mass Spectrom. Ion Processes* **1991,** *111*, 89-102.

(2) Wei, Y.; Zhang, Y.; Lin, Y.; Li, L.; Liu, J.; Wang, Z.; Xiong, S.; Zhao, Z. A uniform 2,5-dihydroxybenzoic acid layer as a matrix for MALDI-FTICR MS-based lipidomics. *Analyst* **2015,** *140* (4), 1298-1305.

(3) Madeira, P. J.; Florencio, M. H. Flavonoid-matrix cluster ions in MALDI mass spectrometry. *J. Mass Spectrom.* **2009,** *44* (7), 1105-1113.

(4) Dunne, J.; Griner, J.; Romeo, M.; Macdonald, J.; Krieg, C.; Lim, M.; Yagnik, G.; Rothschild, K. J.; Drake, R. R.; Mehta, A. S.et al. Evaluation of antibody-based single cell type imaging techniques coupled to multiplexed imaging of N-glycans and collagen peptides by matrix-assisted laser desorption/ionization mass spectrometry imaging. *Anal. Bioanal. Chem.* **2023,** *415* (28), 7011-7024.

(5) Tang, K.; Allman, S. L.; Chen, C. H. Matrix-assisted laser desorption ionization of oligonucleotides with various matrices. *Rapid Commun. Mass Spectrom.* **1993,** *7* (10), 943-948.

(6) Nelson, R. W.; Dogruel, D.; Williams, P. Mass determination of human immunoglobulin IgM using matrix-assisted laser desorption/ionization time-of-flight mass spectrometry. *Rapid Commun. Mass Spectrom.* **1994,** *8* (8), 627-631.

(7) Schlosser, G.; Pocsfalvi, G.; Malorni, A.; Puerta, A.; de Frutos, M.; Vekey, K. Detection of immune complexes by matrix-assisted laser desorption/ionization mass spectrometry. *Rapid Commun. Mass Spectrom.* **2003,** *17* (24), 2741-2747.

(8) Salum, M. L.; Itovich, L. M.; Erra-Balsells, R. Z-sinapinic acid: the change of the stereochemistry of cinnamic acids as rational synthesis of a new matrix for carbohydrate MALDI-MS analysis. *J. Mass Spectrom.* **2013,** *48* (11), 1160-1169.

(9) Salum, M. L.; Giudicessi, S. L.; Schmidt De Leon, T.; Camperi, S. A.; Erra-Balsells, R. Application of Z-sinapinic matrix in peptide MALDI-MS analysis. *J. Mass Spectrom.* **2017,** *52* (3), 182-186.

(10) Yang, H.; Liu, N.; Liu, S. Determination of peptide and protein disulfide linkages by MALDI mass spectrometry. *Top. Curr. Chem.* **2013,** *331*, 79-116.

(11) Beavis, R. C.; Chaudhary, T.; Chait, B. T. α-Cyano-4-hydroxycinnamic acid as a matrix for matrixassisted laser desorption mass spectromtry. *Org. Mass Spectrom.* **1992,** *27* (2), 156-158.

(12) Urso, E.; Le Pera, M.; Bossio, S.; Sprovieri, T.; Qualtieri, A. Quantification of thymosin beta(4) in human cerebrospinal fluid using matrix-assisted laser desorption/ionization time-of-flight mass spectrometry. *Anal. Biochem.* **2010,** *402* (1), 13-19.

(13) Shikano, H.; Miyama, Y.; Ikeda, R.; Takeshi, H.; Suda, J.; Yoshinaga, K.; Taira, S. Localization Analysis of Multiple Vitamins in Dried Persimmon (Diospyros kaki) Using Matrix-assisted Laser Desorption/ionization Mass Spectrometry Imaging. *J. Oleo Sci.* **2020,** *69* (8), 959-964.

(14) Carter, C. L.; McLeod, C. W.; Bunch, J. Imaging of phospholipids in formalin fixed rat brain sections by matrix assisted laser desorption/ionization mass spectrometry. *J. Am. Soc. Mass Spectrom.* **2011,** *22* (11), 1991-1998.

(15) Stanback, A. E.; Conroy, L. R.; Young, L. E. A.; Hawkinson, T. R.; Markussen, K. H.; Clarke, H. A.; Allison, D. B.; Sun, R. C. Regional N-glycan and lipid analysis from tissues using MALDI-mass spectrometry imaging. *STAR Protoc.* **2021,** *2* (1), 100304.

(16) Vermillion-Salsbury, R. L.; Hercules, D. M. 9-Aminoacridine as a matrix for negative mode matrix-assisted laser desorption/ionization. *Rapid Commun. Mass Spectrom.* **2002,** *16* (16), 1575-1581.

(17) Mims, D.; Hercules, D. Quantification of bile acids directly from urine by MALDI-TOF-MS. *Anal.*

Bioanal. Chem. **2003,** *375* (5), 609-616.

(18) Shroff, R.; Muck, A.; Svatos, A. Analysis of low molecular weight acids by negative mode matrix-assisted laser desorption/ionization time-of-flight mass spectrometry. *Rapid Commun. Mass Spectrom.* **2007,** *21* (20), 3295-3300.

(19) Sun, C.; Zhang, M.; Dong, H.; Liu, W.; Guo, L.; Wang, X. A spatially-resolved approach to visualize the distribution and biosynthesis of flavones in Scutellaria baicalensis Georgi. *J. Pharm. Biomed. Anal.* **2020,** *179*, 113014.

(20) Sun, G.; Yang, K.; Zhao, Z.; Guan, S.; Han, X.; Gross, R. W. Matrix-assisted laser desorption/ionization time-of-flight mass spectrometric analysis of cellular glycerophospholipids enabled by multiplexed solvent dependent analyte-matrix interactions. *Anal. Chem.* **2008,** *80* (19), 7576-7585.

(21) Cerruti, C. D.; Benabdellah, F.; Laprevote, O.; Touboul, D.; Brunelle, A. MALDI imaging and structural analysis of rat brain lipid negative ions with 9-aminoacridine matrix. *Anal. Chem.* **2012,** *84* (5), 2164-2171.

(22) Thiede, B.; von Janta-Lipinski, M. Noncovalent RNA-peptide complexes detected by matrix-assisted laser desorption/ionization mass spectrometry. *Rapid Commun. Mass Spectrom.* **1998,** *12* (23), 1889-1894.

(23) Pieles, U.; Zurcher, W.; Schar, M.; Moser, H. E. Matrix-assisted laser desorption ionization time-of-flight mass spectrometry: a powerful tool for the mass and sequence analysis of natural and modified oligonucleotides. *Nucleic Acids Res.* **1993,** *21* (14), 3191-3196.

(24) Hsu, N. Y.; Yang, W. B.; Wong, C. H.; Lee, Y. C.; Lee, R. T.; Wang, Y. S.; Chen, C. H. Matrix-assisted laser desorption/ionization mass spectrometry of polysaccharides with 2',4',6'-trihydroxyacetophenone as matrix. *Rapid Commun. Mass Spectrom.* **2007,** *21* (13), 2137-2146.

(25) Stubiger, G.; Belgacem, O.; Rehulka, P.; Bicker, W.; Binder, B. R.; Bochkov, V. Analysis of oxidized phospholipids by MALDI mass spectrometry using 6-aza-2-thiothymine together with matrix additives and disposable target surfaces. *Anal. Chem.* **2010,** *82* (13), 5502-5510.

(26) Wang, J.; Sporns, P. MALDI-TOF MS analysis of food flavonol glycosides. *J. Agric. Food Chem.* **2000,** *48* (5), 1657-1662.

(27) Molin, L.; Seraglia, R.; Dani, F. R.; Moneti, G.; Traldi, P. The double nature of 1,5-diaminonaphthalene as matrix-assisted laser desorption/ionization matrix: some experimental evidence of the protonation and reduction mechanisms. *Rapid Commun. Mass Spectrom.* **2011,** *25* (20), 3091-3096.

(28) Fukuyama, Y.; Iwamoto, S.; Tanaka, K. Rapid sequencing and disulfide mapping of peptides containing disulfide bonds by using 1,5-diaminonaphthalene as a reductive matrix. *J. Mass Spectrom.* **2006,** *41* (2), 191-201.

(29) Strnad, S.; Prazienkova, V.; Sykora, D.; Cvacka, J.; Maletinska, L.; Popelova, A.; Vrkoslav, V. The use of 1,5-diaminonaphthalene for matrix-assisted laser desorption/ionization mass spectrometry imaging of brain in neurodegenerative disorders. *Talanta* **2019,** *201*, 364-372.

(30) Thomas, A.; Charbonneau, J. L.; Fournaise, E.; Chaurand, P. Sublimation of new matrix candidates for high spatial resolution imaging mass spectrometry of lipids: enhanced information in both positive and negative polarities after 1,5-diaminonapthalene deposition. *Anal. Chem.* **2012,** *84* (4), 2048-2054.

(31) Caughlin, S.; Park, D. H.; Yeung, K. K.; Cechetto, D. F.; Whitehead, S. N. Sublimation of DAN Matrix for the Detection and Visualization of Gangliosides in Rat Brain Tissue for MALDI Imaging Mass Spectrometry. *J. Vis. Exp.* **2017,** (121), 55254.

(32) Wu, K. J.; Shaler, T. A.; Becker, C. H. Time-of-flight mass spectrometry of underivatized single-stranded DNA oligomers by matrix-assisted laser desorption. *Anal. Chem.* **1994,** *66* (10), 1637-1645.

(33) Wu, K. J.; Steding, A.; Becker, C. H. Matrix-assisted laser desorption time-of-flight mass spectrometry of oligonucleotides using 3-hydroxypicolinic acid as an ultraviolet-sensitive matrix. *Rapid Commun. Mass Spectrom.* **1993,** *7* (2), 142-146.

(34) Streletskii, A. V.; Kozlova, A. Y.; Esipov, D. S.; Kayushin, A. L.; Korosteleva, M. D.; Esipov, S. E. Determination of oligonucleotide molecular masses by MALDI mass spectrometry. *uss. J. Bioorg. Chem.* **2005,** *31* (2), 139-145.

(35) Salim, H.; Gimenez, E.; Sanz-Nebot, V.; Benavente, F. Ionic matrices for matrix-assisted laser desorption/ionization mass spectrometry analysis of microRNA biomarkers. *Anal. Chim. Acta* **2020,** *1139*, 169-177.

(36) Le, C. H.; Han, J.; Borchers, C. H. Dithranol as a MALDI matrix for tissue imaging of lipids by Fourier transform ion cyclotron resonance mass spectrometry. *Anal. Chem.* **2012,** *84* (19), 8391-8398.

(37) Wang, X.; Han, J.; Chou, A.; Yang, J.; Pan, J.; Borchers, C. H. Hydroxyflavones as a new family of matrices for MALDI tissue imaging. *Anal. Chem.* **2013,** *85* (15), 7566-7573.

(38) Schaiberger, A. M.; Moss, J. A. Optimized sample preparation for MALDI mass spectrometry analysis of protected synthetic peptides. *J. Am. Soc. Mass Spectrom.* **2008,** *19* (4), 614-619.

(39) Li, Y.; Liang, M.; Shu, J.; Zhang, P.; Sun, W. Analysis of phytosterol by MALDI-TOF mass spectrometry. *Anal. Methods* **2014,** *6* (13), 4754-4760.

(40) Yoo, H.-J.; Kim, D.-H.; Shin, D.; Oh, Y.; Lee, S.; Lee, J. Y.; Choi, Y.-J.; Lee, S. H.; Lee, K.-S.; Kim, Y.et al. Recent developments in pre-treatment and analytical techniques for synthetic polymers by MALDI-TOF mass spectrometry. *Anal. Methods* **2020,** *12* (48), 5767-5800.

(41) Lecchi, P.; Le, H. M.; Pannell, L. K. 6-Aza-2-thiothymine: a matrix for MALDI spectra of oligonucleotides. *Nucleic Acids Res.* **1995,** *23* (7), 1276-1277.

(42) Lecchi, P.; Pannell, L. K. The detection of intact double-stranded DNA by MALDI. *J. Am. Soc. Mass Spectrom.* **1995,** *6* (10), 972-975.

(43) Ohara, K.; Smietana, M.; Vasseur, J. J. Characterization of specific noncovalent complexes between guanidinium derivatives and single-stranded DNA by MALDI. *J. Am. Soc. Mass Spectrom.* **2006,** *17* (3), 283-291.

(44) Denti, V.; Monza, N.; Bindi, G.; Porto, N. S.; L'Imperio, V.; Pagni, F.; Piga, I.; Smith, A. 6-Aza-2-Thiothymine as an Alternative Matrix for Spatial Proteomics with MALDI-MSI. *Int. J. Mol. Sci.* **2024,** *25* (24), 13678.

(45) Geyer, H.; Schmitt, S.; Wuhrer, M.; Geyer, R. Structural analysis of glycoconjugates by on-target enzymatic digestion and MALDI-TOF-MS. *Anal. Chem.* **1999,** *71* (2), 476-482.

(46) Rohmer, M.; Meyer, B.; Mank, M.; Stahl, B.; Bahr, U.; Karas, M. 3-Aminoquinoline acting as matrix and derivatizing agent for MALDI MS analysis of oligosaccharides. *Anal. Chem.* **2010,** *82* (9), 3719-3726.

(47) Fukuyama, Y.; Funakoshi, N.; Takeyama, K.; Hioki, Y.; Nishikaze, T.; Kaneshiro, K.; Kawabata, S.; Iwamoto, S.; Tanaka, K. 3-Aminoquinoline/p-coumaric acid as a MALDI matrix for glycopeptides, carbohydrates, and phosphopeptides. *Anal. Chem.* **2014,** *86* (4), 1937-1942.

(48) Yang, S. H.; Reddy, P. M.; Ho, Y. P. Concentration and in situ detection of peptides using liquid matrix-assisted laser desorption ionization matrixes. *Anal. Chem.* **2010,** *82* (1), 44-48.

(49) Watanabe, M.; Terasawa, K.; Kaneshiro, K.; Uchimura, H.; Yamamoto, R.; Fukuyama, Y.; Shimizu, K.; Sato, T.-A.; Tanaka, K. Improvement of mass spectrometry analysis of glycoproteins by MALDI-MS using 3-aminoquinoline/α-cyano-4-hydroxycinnamic acid. *Anal. Bioanal. Chem.* **2013,** *405* (12), 4289-4293.

(50) Nishikaze, T.; Kaneshiro, K.; Kawabata, S.-i.; Tanaka, K. Structural Analysis of N-Glycans by the Glycan-Labeling Method Using 3-Aminoquinoline-Based Liquid Matrix in Negative-Ion MALDI-MS. *Anal. Chem.* **2012,** *84* (21), 9453-9461.

(51) Wyatt, M. F.; Stein, B. K.; Brenton, A. G. Characterization of various analytes using matrix-assisted laser desorption/ionization time-of-flight mass spectrometry and 2-[(2E)-3-(4-tert-butylphenyl)-2-methylprop-2-enylidene]malononitrile matrix. *Anal. Chem.* **2006,** *78* (1), 199-206.

(52) De Winter, J.; Deshayes, G.; Boon, F.; Coulembier, O.; Dubois, P.; Gerbaux, P. MALDI-ToF analysis of polythiophene: use of trans-2-[3-(4-t-butyl-phenyl)-2-methyl- 2-propenylidene]malononitrile-DCTB-as matrix. *J. Mass Spectrom.* **2011,** *46* (3), 237-246.

(53) Ulmer, L.; Mattay, J.; Torres-Garcia, H. G.; Luftmann, H. Letter: The Use of 2-[(2E)-3-(4-Tert-Butylphenyl)-2-Methylprop-2-Enylidene]Malononitrile as a Matrix for Matrix-Assisted Laser Desorption/Ionization Mass Spectrometry. *Eur. J. Mass Spectrom.* **2000,** *6* (1), 49-52.

(54) Lou, X.; de Waal, B. F.; Milroy, L. G.; van Dongen, J. L. A sample preparation method for recovering suppressed analyte ions in MALDI TOF MS. *J. Mass Spectrom.* **2015,** *50* (5), 766-770.

(55) Wang, T.; Cai, Z.; Chen, Y.; Lee, W. K.; Kwan, C.-S.; Li, M.; Chan, A. S. C.; Chen, Z.-F.; Cheung, A. K. L.; Leung, K. C.-F. MALDI-MS Imaging Analysis of Noninflammatory Type III Rotaxane Dendrimers. *J. Am. Soc. Mass Spectrom.* **2020,** *31* (12), 2488-2494.

(56) Krause, J.; Stoeckli, M.; Schlunegger, U. P. Studies on the selection of new matrices for ultraviolet matrix-assisted laser desorption/ionization time-of-flight mass spectrometry. *Rapid Commun. Mass Spectrom.* **1996,** *10* (15), 1927-1933.

(57) Wenzel, T.; Sparbier, K.; Mieruch, T.; Kostrzewa, M. 2,5-Dihydroxyacetophenone: a matrix for highly sensitive matrix-assisted laser desorption/ionization time-of-flight mass spectrometric analysis of proteins using manual and automated preparation techniques. *Rapid Commun. Mass Spectrom.* **2006,** *20* (5), 785-789.

(58) Zaikin, V. G.; Borisov, R. S.; Polovkov, N. Y.; Slyundina, M. S. Reactive matrices for matrix-assisted laser desorption/ionization mass spectrometry of primary amines. *Eur. J. Mass Spectrom. (Chichester)* **2015,** *21* (3), 403-411.

(59) Schroter, J.; Fulop, A.; Hopf, C.; Schiller, J. The combination of 2,5-dihydroxybenzoic acid and 2,5-dihydroxyacetophenone matrices for unequivocal assignment of phosphatidylethanolamine species in complex mixtures. *Anal. Bioanal. Chem.* **2018,** *410* (9), 2437-2447.

(60) Hayasaka, T.; Goto-Inoue, N.; Zaima, N.; Shrivas, K.; Kashiwagi, Y.; Yamamoto, M.; Nakamoto, M.; Setou, M. Imaging mass spectrometry with silver nanoparticles reveals the distribution of fatty acids in mouse retinal sections. *J. Am. Soc. Mass Spectrom.* **2010,** *21* (8), 1446-1454.

(61) Xu, N.; Huang, Z. H.; de Jonge, B. L.; Gage, D. A. Structural characterization of peptidoglycan muropeptides by matrix-assisted laser desorption ionization mass spectrometry and postsource decay analysis. *Anal. Biochem.* **1997,** *248* (1), 7-14.

(62) Astigarraga, E.; Barreda-Gomez, G.; Lombardero, L.; Fresnedo, O.; Castano, F.; Giralt, M. T.; Ochoa, B.; Rodriguez-Puertas, R.; Fernandez, J. A. Profiling and imaging of lipids on brain and liver tissue by matrix-assisted laser desorption/ ionization mass spectrometry using 2-mercaptobenzothiazole as a matrix. *Anal. Chem.* **2008,** *80* (23), 9105-9114.

(63) Liu, Y.; Nie, X.; Wang, J.; Zhao, Z.; Wang, Z.; Ju, F. Visualizing the distribution of flavonoids in litchi (Litchi chinenis) seeds through matrix-assisted laser desorption/ionization mass spectrometry imaging. *Front. Plant Sci.* **2023,** *14*, 1144449.

(64) Dos Santos, N. A.; de Almeida, C. M.; Goncalves, F. F.; Ortiz, R. S.; Kuster, R. M.; Saquetto, D.; Romao,

W. Analysis of Erythroxylum coca Leaves by Imaging Mass Spectrometry (MALDI-FT-ICR IMS). *J. Am. Soc. Mass Spectrom.* **2021,** *32* (4), 946-955.

(65) Garate, J.; Fernández, R.; Lage, S.; Bestard-Escalas, J.; Lopez, D. H.; Reigada, R.; Khorrami, S.; Ginard, D.; Reyes, J.; Amengual, I.et al. Imaging mass spectrometry increased resolution using 2-mercaptobenzothiazole and 2,5-diaminonaphtalene matrices: application to lipid distribution in human colon. *Anal. Bioanal. Chem.* **2015,** *407* (16), 4697-4708.

(66) Brombacher, S.; Owen, S. J.; Volmer, D. A. Automated coupling of capillary-HPLC to matrix-assisted laser desorption/ionization mass spectrometry for the analysis of small molecules utilizing a reactive matrix. *Anal. Bioanal. Chem.* **2003,** *376* (6), 773-779.

(67) Fenaille, F.; Tabet, J. C.; Guy, P. A. Identification of 4-hydroxy-2-nonenal-modified peptides within unfractionated digests using matrix-assisted laser desorption/ionization time-of-flight mass spectrometry. *Anal. Chem.* **2004,** *76* (4), 867-873.

(68) Teuber, K.; Fedorova, M.; Hoffmann, R.; Schiller, J. 2,4-Dinitrophenylhydrazine as a New Reactive Matrix to Analyze Oxidized Phospholipids by MALDI-TOF Mass Spectrometry. *Anal. Lett.* **2012,** *45* (9), 968-976.

(69) Shigeri, Y.; Ikeda, S.; Yasuda, A.; Ando, M.; Sato, H.; Kinumi, T. Hydrazide and hydrazine reagents as reactive matrices for MALDI-MS to detect gaseous aldehydes. *J. Mass Spectrom.* **2014,** *49* (8), 742-749.

(70) Flinders, B.; Morrell, J.; Marshall, P. S.; Ranshaw, L. E.; Clench, M. R. The use of hydrazine-based derivatization reagents for improved sensitivity and detection of carbonyl containing compounds using MALDI-MSI. *Anal. Bioanal. Chem.* **2015,** *407* (8), 2085-2094.

(71) Nonami, H.; Fukui, S.; Erra-Balsells, R. β-Carboline alkaloids as matrices for matrix-assisted ultraviolet laser desorption time-of-flight mass spectrometry of proteins and sulfated oligosaccharides: a comparative study using phenylcarbonyl compounds, carbazoles and classical matrices. *J. Mass Spectrom.* **1997,** *32* (3), 287-296.

(72) Monge, M. E.; Negri, R. M.; Kolender, A. A.; Erra-Balsells, R. Structural characterization of native high-methoxylated pectin using nuclear magnetic resonance spectroscopy and ultraviolet matrix-assisted laser desorption/ionization time-of-flight mass spectrometry. Comparative use of 2,5-dihydroxybenzoic acid and nor-harmane as UV-MALDI matrices. *Rapid Commun. Mass Spectrom.* **2007,** *21* (16), 2638-2646.

(73) Fukuyama, Y.; Kolender, A. A.; Nishioka, M.; Nonami, H.; Matulewicz, M. C.; Erra-Balsells, R.; Cerezo, A. S. Matrix-assisted ultraviolet laser desorption/ionization time-of-flight mass spectrometry of beta-(1 --> 3), beta-(1 --> 4)-xylans from Nothogenia fastigiata using nor-harmane as matrix. *Rapid Commun. Mass Spectrom.* **2005,** *19* (3), 349-358.

(74) Scott, A. J.; Flinders, B.; Cappell, J.; Liang, T.; Pelc, R. S.; Tran, B.; Kilgour, D. P.; Heeren, R. M.; Goodlett, D. R.; Ernst, R. K. Norharmane Matrix Enhances Detection of Endotoxin by MALDI-MS for Simultaneous Profiling of Pathogen, Host, and Vector Systems. *Pathog. Dis.* **2016,** *74* (8), ftw097.

(75) Fukuyama, Y.; Ciancia, M.; Nonami, H.; Cerezo, A. S.; Erra-Balsells, R.; Matulewicz, M. a. C. Matrix-assisted ultraviolet laser-desorption ionization and electrospray-ionization time-of-flight mass spectrometry of sulfated neocarrabiose oligosaccharides. *Carbohydr. Res.* **2002,** *337* (17), 1553-1562.

(76) Casadio, R.; Melandri, B. A. The behavior of 9-aminoacridine as an indicator of transmembrane pH difference in liposomes of natural bacterial phospholipids. *J. Bioenerg. Biomembr.* **1977,** *9* (1), 17-29.

(77) Gut, I. G.; Jeffery, W. A.; Pappin, D. J. C.; Beck, S. Analysis of DNA by 'Charge Tagging' and Matrix-assisted Laser Desorption/Ionization Mass Spectrometry. *Rapid Commun. Mass Spectrom.* **1997,** *11* (1), 43-50.

(78) Zhang, J.; Knochenmuss, R.; Stevenson, E.; Zenobi, R. The gas-phase sodium basicities of common

matrix-assisted laser desorption/ionization matrices. *Int. J. Mass spectrom.* **2002,** *213* (2), 237-250.

(79) Cai, Y.; Jiang, Y.; Cole, R. B. Anionic adducts of oligosaccharides by matrix-assisted laser desorption/ionization time-of-flight mass spectrometry. *Anal. Chem.* **2003,** *75* (7), 1638-1644.

(80) Smyk, B. Fluorescence Study of Sinapic Acid Interaction with Bovine Serum Albumin and Egg Albumin. *J. Fluoresc.* **2003,** *13* (4), 349-356.

(81) Takehira, K.; Sugawara, Y.; Kowase, S.; Tobita, S. A picosecond time-resolved study on prototropic reactions of electronically excited 1,5- and 1,8-diaminonaphthalenes in aqueous solution. *Photochemical & Photobiological Sciences* **2005,** *4* (3), 287-293.

(82) Barylyuk, K.; Fritsche, L.; Balabin, R. M.; Nieckarz, R.; Zenobi, R. Gas-phase basicity of several common MALDI matrices measured by a simple experimental approach. *RSC Adv.* **2012,** *2* (5), 1962-1969.

(83) Jing, P.; Hou, M.; Zhao, P.; Tang, X.; Wan, H. Adsorption of 2-mercaptobenzothiazole from aqueous solution by organo-bentonite. *Journal of Environmental Sciences* **2013,** *25* (6), 1139-1144.

(84) Cai, Y.; Zhang, Y.; Yang, P.; Lu, H. Improved analysis of oligosaccharides for matrix-assisted laser desorption/ionization time-of-flight mass spectrometry using aminopyrazine as a derivatization reagent and a co-matrix. *Analyst* **2013,** *138* (21), 6270-6276.

(85) Zhang, H.; Smith, M. A.; Purves, R. W. Optimization of Triacylglycerol-estolide Analysis by Matrix-Assisted Laser Desorption/Ionization-Mass Spectrometry. *J. Am. Oil Chem. Soc.* **2014,** *91* (6), 905-915.

(86) Georgiou, C. D.; Zisimopoulos, D.; Argyropoulou, V.; Kalaitzopoulou, E.; Salachas, G.; Grune, T. Protein and cell wall polysaccharide carbonyl determination by a neutral pH 2,4-dinitrophenylhydrazine-based photometric assay. *Redox Biology* **2018,** *17*, 128-142.

(87) Rzagalinski, I.; Kovacevic, B.; Hainz, N.; Meier, C.; Tschernig, T.; Volmer, D. A. Toward Higher Sensitivity in Quantitative MALDI Imaging Mass Spectrometry of CNS Drugs Using a Nonpolar Matrix. *Anal. Chem.* **2018,** *90* (21), 12592-12600.

(88) Adhikari, M.; Joshi, N. K.; Joshi, H. C.; Mehata, M. S.; Mishra, H.; Pant, S. Revisiting the photochemistry 2,5-dihydroxy benzoic acid (gentisic acid): Solvent and pH effect. *J. Phys. Org. Chem.* **2021,** *34* (4), e4168.

(89) Tammekivi, E.; Ghiami-Shomami, A.; Tshepelevitsh, S.; Trummal, A.; Ilisson, M.; Selberg, S.; Vahur, S.; Teearu, A.; Lõkov, M.; Peets, P.et al. Experimental and Computational Study of Aminoacridines as MALDI(−)-MS Matrix Materials for the Analysis of Complex Samples. *J. Am. Soc. Mass Spectrom.* **2021,** *32* (4), 1080-1095.

(90) Hillenkamp, F.; Wäfler, E.; Jecklin, M. C.; Zenobi, R. Positive and negative analyte ion yield in matrix-assisted laser desorption/ionization revisited. *Int. J. Mass spectrom.* **2009,** *285* (3), 114-119.

(91) Rein, F. N.; Rocha, R. C.; Toma, H. E. Redox and pH-induced switching of the coordination sites in the 3-hydroxypicolinate ruthenium (III)-edta complex. *J. Coord. Chem.* **2001,** *53* (2), 99-123.

(92) McLean, J. A.; Stumpo, K. A.; Russell, D. H. Size-selected (2-10 nm) gold nanoparticles for matrix assisted laser desorption ionization of peptides. *J. Am. Chem. Soc.* **2005,** *127* (15), 5304-5305.

(93) Su, C. L.; Tseng, W. L. Gold nanoparticles as assisted matrix for determining neutral small carbohydrates through laser desorption/ionization time-of-flight mass spectrometry. *Anal. Chem.* **2007,** *79* (4), 1626-1633.

(94) Tang, H. W.; Lu, W.; Che, C. M.; Ng, K. M. Gold nanoparticles and imaging mass spectrometry: double imaging of latent fingerprints. *Anal. Chem.* **2010,** *82* (5), 1589-1593.

(95) Chiang, N. C.; Chiang, C. K.; Lin, Z. H.; Chiu, T. C.; Chang, H. T. Detection of aminothiols through surface-assisted laser desorption/ionization mass spectrometry using mixed gold nanoparticles. *Rapid*

Commun. Mass Spectrom. **2009,** *23* (19), 3063-3068.

(96) Wei, X.; Liu, Z.; Jin, X.; Huang, L.; Gurav, D. D.; Sun, X.; Liu, B.; Ye, J.; Qian, K. Plasmonic nanoshells enhanced laser desorption/ionization mass spectrometry for detection of serum metabolites. *Anal. Chim. Acta* **2017,** *950*, 147-155.

(97) Chiu, T. C.; Chang, L. C.; Chiang, C. K.; Chang, H. T. Determining estrogens using surface-assisted laser desorption/ionization mass spectrometry with silver nanoparticles as the matrix. *J. Am. Soc. Mass Spectrom.* **2008,** *19* (9), 1343-1346.

(98) Jackson, S. N.; Baldwin, K.; Muller, L.; Womack, V. M.; Schultz, J. A.; Balaban, C.; Woods, A. S. Imaging of lipids in rat heart by MALDI-MS with silver nanoparticles. *Anal. Bioanal. Chem.* **2014,** *406* (5), 1377-1386.

(99) Muller, L.; Kailas, A.; Jackson, S. N.; Roux, A.; Barbacci, D. C.; Schultz, J. A.; Balaban, C. D.; Woods, A. S. Lipid imaging within the normal rat kidney using silver nanoparticles by matrix-assisted laser desorption/ionization mass spectrometry. *Kidney Int.* **2015,** *88* (1), 186-192.

(100) Kinumi, T.; Saisu, T.; Takayama, M.; Niwa, H. Matrix-assisted laser desorption/ionization time-of-flight mass spectrometry using an inorganic particle matrix for small molecule analysis. *J. Mass Spectrom.* **2000,** *35* (3), 417-422.

(101) Shrivas, K.; Hayasaka, T.; Sugiura, Y.; Setou, M. Method for simultaneous imaging of endogenous low molecular weight metabolites in mouse brain using TiO2 nanoparticles in nanoparticle-assisted laser desorption/ionization-imaging mass spectrometry. *Anal. Chem.* **2011,** *83* (19), 7283-7289.

(102) Peng, C.; Zhang, Q.; Liu, J.-a.; Wang, Z.-p.; Zhao, Z.-w.; Kang, N.; Chen, Y.; Huo, Q. Study on titanium dioxide nanoparticles as MALDI MS matrix for the determination of lipids in the brain. *Green Process. Synth.* **2021,** *10* (1), 700-710.

(103) Olaitan, A. D.; Reyes, K. A.; Barnes, L. F.; Yount, J. R.; Ward, S.; Hamilton, H. S. C.; King, K. E.; Van Leeuwen, C. J.; Stepherson, J. R.; Vargas, T. K.et al. Transition metal oxide nanoparticles as surfaces for surface-assisted laser desorption/ionization mass spectrometry of asphaltenes. *Pet. Sci. Technol.* **2017,** *35* (19), 1917-1924.

(104) Xu, S.; Li, Y.; Zou, H.; Qiu, J.; Guo, Z.; Guo, B. Carbon nanotubes as assisted matrix for laser desorption/ionization time-of-flight mass spectrometry. *Anal. Chem.* **2003,** *75* (22), 6191-6195.

(105) Ren, S. F.; Zhang, L.; Cheng, Z. H.; Guo, Y. L. Immobilized carbon nanotubes as matrix for MALDI-TOF-MS analysis: applications to neutral small carbohydrates. *J. Am. Soc. Mass Spectrom.* **2005,** *16* (3), 333-339.

(106) Chen, W. Y.; Wang, L. S.; Chiu, H. T.; Chen, Y. C.; Lee, C. Y. Carbon nanotubes as affinity probes for peptides and proteins in MALDI MS analysis. *J. Am. Soc. Mass Spectrom.* **2004,** *15* (11), 1629-1635.

(107) Hu, L.; Xu, S.; Pan, C.; Yuan, C.; Zou, H.; Jiang, G. Matrix-assisted laser desorption/ionization time-of-flight mass spectrometry with a matrix of carbon nanotubes for the analysis of low-mass compounds in environmental samples. *Environ. Sci. Technol.* **2005,** *39* (21), 8442-8447.

(108) Pan, C.; Xu, S.; Hu, L.; Su, X.; Ou, J.; Zou, H.; Guo, Z.; Zhang, Y.; Guo, B. Using oxidized carbon nanotubes as matrix for analysis of small molecules by MALDI-TOF MS. *J. Am. Soc. Mass Spectrom.* **2005,** *16* (6), 883-892.

(109) Dong, X.; Cheng, J.; Li, J.; Wang, Y. Graphene as a novel matrix for the analysis of small molecules by MALDI-TOF MS. *Anal. Chem.* **2010,** *82* (14), 6208-6214.

(110) Lu, M.; Lai, Y.; Chen, G.; Cai, Z. Matrix interference-free method for the analysis of small molecules by using negative ion laser desorption/ionization on graphene flakes. *Anal. Chem.* **2011,** *83* (8), 3161-3169.

(111) Chang, C.; Li, X.; Bai, Y.; Xu, G.; Feng, B.; Liao, Y.; Liu, H. Graphene matrix for signal enhancement

in ambient plasma assisted laser desorption ionization mass spectrometry. *Talanta* **2013,** *114*, 54-59.

(112) Liu, C. W.; Chien, M. W.; Su, C. Y.; Chen, H. Y.; Li, L. J.; Lai, C. C. Analysis of flavonoids by graphene-based surface-assisted laser desorption/ionization time-of-flight mass spectrometry. *Analyst* **2012,** *137* (24), 5809-5816.

(113) Lee, D.; Kim, Y.; Jalaludin, I.; Nguyen, H. Q.; Kim, M.; Seo, J.; Jang, K. S.; Kim, J. MALDI-MS analysis of disaccharide isomers using graphene oxide as MALDI matrix. *Food Chem.* **2021,** *342*, 128356.

(114) Zhou, D.; Guo, S.; Zhang, M.; Liu, Y.; Chen, T.; Li, Z. Mass spectrometry imaging of small molecules in biological tissues using graphene oxide as a matrix. *Anal. Chim. Acta* **2017,** *962*, 52-59.

(115) Liu, Y.; Liu, J.; Yin, P.; Gao, M.; Deng, C.; Zhang, X. High throughput identification of components from traditional Chinese medicine herbs by utilizing graphene or graphene oxide as MALDI-TOF-MS matrix. *J. Mass Spectrom.* **2011,** *46* (8), 804-815.

(116) Wei, J.; Buriak, J. M.; Siuzdak, G. Desorption-ionization mass spectrometry on porous silicon. *Nature* **1999,** *399* (6733), 243-246.

(117) Liu, Q.; Guo, Z.; He, L. Mass spectrometry imaging of small molecules using desorption/ionization on silicon. *Anal. Chem.* **2007,** *79* (10), 3535-3541.

(118) Rudd, D.; Ronci, M.; Johnston, M. R.; Guinan, T.; Voelcker, N. H.; Benkendorff, K. Mass spectrometry imaging reveals new biological roles for choline esters and Tyrian purple precursors in muricid molluscs. *Sci. Rep.* **2015,** *5*, 13408.

(119) Thomas, J. J.; Shen, Z.; Crowell, J. E.; Finn, M. G.; Siuzdak, G. Desorption/ionization on silicon (DIOS): a diverse mass spectrometry platform for protein characterization. *Proc. Natl. Acad. Sci. U. S. A.* **2001,** *98* (9), 4932-4937.

(120) Arakawa, R.; Shimomae, Y.; Morikawa, H.; Ohara, K.; Okuno, S. Mass spectrometric analysis of low molecular mass polyesters by laser desorption/ionization on porous silicon. *J. Mass Spectrom.* **2004,** *39* (8), 961-965.

(121) Northen, T. R.; Yanes, O.; Northen, M. T.; Marrinucci, D.; Uritboonthai, W.; Apon, J.; Golledge, S. L.; Nordstrom, A.; Siuzdak, G. Clathrate nanostructures for mass spectrometry. *Nature* **2007,** *449* (7165), 1033-1036.

(122) Yanes, O.; Woo, H. K.; Northen, T. R.; Oppenheimer, S. R.; Shriver, L.; Apon, J.; Estrada, M. N.; Potchoiba, M. J.; Steenwyk, R.; Manchester, M.et al. Nanostructure initiator mass spectrometry: tissue imaging and direct biofluid analysis. *Anal. Chem.* **2009,** *81* (8), 2969-2975.

(123) Amantonico, A.; Flamigni, L.; Glaus, R.; Zenobi, R. Negative mode nanostructure-initiator mass spectrometry for detection of phosphorylated metabolites. *Metabolomics* **2009,** *5* (3), 346-353.

(124) Viladkar, S. Matrix-assisted laser desorption/ionization mass spectrometry analysis of fluorophore-labeled oligonucleotides using ferulic acid. *J. Mass Spectrom.* **2001,** *36* (8), 973-974.

(125) Stverakova, D.; Sedo, O.; Benesik, M.; Zdrahal, Z.; Doskar, J.; Pantucek, R. Rapid Identification of Intact Staphylococcal Bacteriophages Using Matrix-Assisted Laser Desorption Ionization-Time-of-Flight Mass Spectrometry. *Viruses* **2018,** *10* (4), 176.

(126) Kato, Y.; Hirosawa, N.; Sakamoto, T.; Moriguchi, T.; Takayama, J.; Xuan, M.; Okazaki, M.; Suzuki, Y.; Hoshi, A.; Sakamoto, Y. Characterization of 6-bromoferulic acid as a novel common-use matrix for matrix-assisted laser desorption/ionization time-of-flight mass spectrometry. *Rapid Commun. Mass Spectrom.* **2020,** *34* (7), e8636.

(127) Beavis, R. C.; Chait, B. T. Cinnamic acid derivatives as matrices for ultraviolet laser desorption mass spectrometry of proteins. *Rapid Commun. Mass Spectrom.* **1989,** *3* (12), 432-435.

(128) Bucknall, M.; Fung, K. Y.; Duncan, M. W. Practical quantitative biomedical applications of MALDI-

TOF mass spectrometry. *J. Am. Soc. Mass Spectrom.* **2002,** *13* (9), 1015-1027.

(129) Liu, H.; Han, M.; Li, J.; Qin, L.; Chen, L.; Hao, Q.; Jiang, D.; Chen, D.; Ji, Y.; Han, H.et al. A Caffeic Acid Matrix Improves In Situ Detection and Imaging of Proteins with High Molecular Weight Close to 200,000 Da in Tissues by Matrix-Assisted Laser Desorption/Ionization Mass Spectrometry Imaging. *Anal. Chem.* **2021,** *93* (35), 11920-11928.

(130) He, H.; Qin, L.; Zhang, Y.; Han, M.; Li, J.; Liu, Y.; Qiu, K.; Dai, X.; Li, Y.; Zeng, M.et al. 3,4-Dimethoxycinnamic Acid as a Novel Matrix for Enhanced In Situ Detection and Imaging of Low-Molecular-Weight Compounds in Biological Tissues by MALDI-MSI. *Anal. Chem.* **2019,** *91* (4), 2634-2643.

(131) Francese, S.; Bradshaw, R.; Flinders, B.; Mitchell, C.; Bleay, S.; Cicero, L.; Clench, M. R. Curcumin: a multipurpose matrix for MALDI mass spectrometry imaging applications. *Anal. Chem.* **2013,** *85* (10), 5240-5248.

(132) Shimizu, H.; Jinno, F.; Morohashi, A.; Yamazaki, Y.; Yamada, M.; Kondo, T.; Asahi, S. Application of high-resolution ESI and MALDI mass spectrometry to metabolite profiling of small interfering RNA duplex. *J. Mass Spectrom.* **2012,** *47* (8), 1015-1022.

(133) Jacksen, J.; Emmer, A. Evaluation of 2,6-dihydroxyacetophenone as matrix-assisted laser desorption/ionization matrix for analysis of hydrophobic proteins and peptides. *Anal. Biochem.* **2012,** *425* (1), 18-20.

(134) Gorman, J. J.; Ferguson, B. L.; Nguyen, T. B. Use of 2,6-dihydroxyacetophenone for analysis of fragile peptides, disulphide bonding and small proteins by matrix-assisted laser desorption/ionization. *Rapid Commun. Mass Spectrom.* **1996,** *10* (5), 529-536.

(135) Stubiger, G.; Belgacem, O. Analysis of lipids using 2,4,6-trihydroxyacetophenone as a matrix for MALDI mass spectrometry. *Anal. Chem.* **2007,** *79* (8), 3206-3213.

(136) Xu, N.; Huang, Z.-H.; Watson, J. T.; Gagecor, D. A. Mercaptobenzothiazoles: A new class of matrices for laser desorption ionization mass spectrometry. *J. Am. Soc. Mass Spectrom.* **1997,** *8* (2), 116-124.

(137) Yousefi-Taemeh, M.; Duli, E.; Dabija, L. G.; Lemaire, M.; Ifa, D. R. Sublimation application of 5-chloro-2-mercaptobenzothiazole matrix for matrix-assisted laser desorption/ionization mass spectrometry imaging of mouse kidney. *Rapid Commun. Mass Spectrom.* **2023,** *37* (16), e9594.

(138) Raju, N. P.; Mirza, S. P.; Vairamani, M.; Ramulu, A. R.; Pardhasaradhi, M. 5-Ethyl-2-mercaptothiazole as matrix for matrix-assisted laser desorption/ionization of a broad spectrum of analytes in positive and negative ion mode. *Rapid Commun. Mass Spectrom.* **2001,** *15* (19), 1879-1884.

(139) Mirza, S. P.; Raju, N. P.; Madhavendra, S. S.; Vairamani, M. 5-Amino-2-mercapto-1,3,4-thiadiazole: a new matrix for the efficient matrix-assisted laser desorption/ionization of neutral carbohydrates. *Rapid Commun. Mass Spectrom.* **2004,** *18* (14), 1666-1674.

(140) Schinkovitz, A.; Kenfack, G. T.; Seraphin, D.; Levillain, E.; Dias, M.; Richomme, P. Selective detection of alkaloids in MALDI-TOF: the introduction of a novel matrix molecule. *Anal. Bioanal. Chem.* **2012,** *403* (6), 1697-1705.

(141) Chen, S.; Chen, L.; Wang, J.; Hou, J.; He, Q.; Liu, J.; Wang, J.; Xiong, S.; Yang, G.; Nie, Z. 2,3,4,5-Tetrakis(3',4'-dihydroxylphenyl)thiophene: a new matrix for the selective analysis of low molecular weight amines and direct determination of creatinine in urine by MALDI-TOF MS. *Anal. Chem.* **2012,** *84* (23), 10291-10297.

(142) Yasuda, A.; Ishimaru, T.; Nishihara, S.; Sakai, M.; Kawasaki, H.; Arakawa, R.; Shigeri, Y. A thiophene-containing compound as a matrix for matrix-assisted laser desorption/ ionization mass spectrometry and the electrical conductivity of matrix crystals. *Eur. J. Mass Spectrom. (Chichester)* **2013,** *19* (1), 29-37.

(143) Matsuo, E.; Toda, C.; Watanabe, M.; Ojima, N.; Izumi, S.; Tanaka, K.; Tsunasawa, S.; Nishimura, O. Selective detection of 2-nitrobenzenesulfenyl-labeled peptides by matrix-assisted laser desorption/ionization-time of flight mass spectrometry using a novel matrix. *Proteomics* **2006,** *6* (7), 2042-2049.

(144) Fukuyama, Y.; Izumi, S.; Tanaka, K. 3-Hydroxy-2-Nitrobenzoic Acid as a MALDI Matrix for In-Source Decay and Evaluation of the Isomers. *J. Am. Soc. Mass Spectrom.* **2018,** *29* (11), 2227-2236.

(145) Chen, L.; Zhang, Y.; Hao, Q.; Fu, J.; Bao, Z.; Bu, Y.; Sun, N.; Wu, X.; Lu, L.; Kong, Z.et al. Enhancement of in situ detection and imaging of phytohormones in plant tissues by MALDI-MSI using 2,4-dihydroxy-5-nitrobenzoic acid as a novel matrix. *New Phytol.* **2024,** *243* (5), 2021-2036.

(146) Abdelhamid, H. N.; Wu, H. F. Furoic and mefenamic acids as new matrices for matrix assisted laser desorption/ionization-(MALDI)-mass spectrometry. *Talanta* **2013,** *115*, 442-450.

(147) Sun, Q.; Zhang, S.; Huang, W.; Wang, R.; Chen, Z.; Cai, Z.; Lin, Z. 4-Mercaptobenzoic acid as a MALDI matrix for highly sensitive analysis of metals. *Analyst* **2021,** *146* (5), 1543-1547.

(148) Shao, W.; Sun, Y.; Su, H.; Sun, Q.; Lin, Z. 4-Mercaptobenzoic acid-assisted laser desorption/ionization mass spectrometry for sensitive quantification of cesium and strontium in drinking water. *Rapid Commun. Mass Spectrom.* **2022,** *36* (17), e9342.

(149) Mock, K. K.; Davey, M.; Cottrell, J. S. The analysis of underivatized oligosaccharides by matrix-assisted laser desorption mass spectrometry. *Biochem. Biophys. Res. Commun.* **1991,** *177* (2), 644-651.

(150) Urakami, S.; Hinou, H. Sodium-Doped 3-Amino-4-hydroxybenzoic Acid: Rediscovered Matrix for Direct MALDI Glycotyping of O-Linked Glycopeptides and Intact Mucins. *Int. J. Mol. Sci.* **2023,** *24* (23), 16836.

(151) Beavis, R. C.; Chait, B. T. Matrix-assisted laser desorption ionization mass-spectrometry of proteins. *Methods Enzymol.* **1996,** *270*, 519-551.

(152) Sakakura, M.; Takayama, M. In-source decay and fragmentation characteristics of peptides using 5-aminosalicylic acid as a matrix in matrix-assisted laser desorption/ionization mass spectrometry. *J. Am. Soc. Mass Spectrom.* **2010,** *21* (6), 979-988.

(153) Distler, A. M.; Allison, J. 5-Methoxysalicylic acid and spermine: a new matrix for the matrix-assisted laser desorption/ionization mass spectrometry analysis of oligonucleotides. *J. Am. Soc. Mass Spectrom.* **2001,** *12* (4), 456-462.

(154) Juhasz, P.; Costello, C. E.; Biemann, K. Matrix-assisted laser desorption ionization mass spectrometry with 2-(4-hydroxyphenylazo)benzoic acid matrix. *J. Am. Soc. Mass Spectrom.* **1993,** *4* (5), 399-409.

(155) Fu, J.; Gu, J.; Bao, Z.; Zhou, Y.; Hu, H.; Yang, C.; Wu, R.; Liu, H.; Qin, L.; Xu, H.et al. 2,5-Dihydroxyterephthalic Acid: A Matrix for Improved Detection and Imaging of Amino Acids. *Anal. Chem.* **2023,** *95* (51), 18709-18718.

(156) Streletskiy, A. V.; Goldt, I. V.; Kuvychko, I. V.; Ioffe, I. N.; Sidorov, L. N.; Drewello, T.; Strauss, S. H.; Boltalina, O. V. Application of 9-nitroanthracene as a matrix for laser desorption/ionization analysis of fluorinated fullerenes. *Rapid Commun. Mass Spectrom.* **2004,** *18* (3), 360-362.

(157) Kotsiris, S. G.; Vasil'ev, Y. V.; Streletskii, A. V.; Han, M.; Mark, L. P.; Boltalina, O. V.; Chronakis, N.; Orfanopoulos, M.; Hungerbuhler, H.; Drewello, T. Application and evaluation of solvent-free matrix-assisted laser desorption/ionization mass spectrometry for the analysis of derivatized fullerenes. *Eur. J. Mass Spectrom. (Chichester)* **2006,** *12* (6), 397-408.

(158) Yang, H.; Wang, J.; Song, F.; Zhou, Y.; Liu, S. Isoliquiritigenin (4,2',4'-trihydroxychalcone): a new matrix-assisted laser desorption/ionization matrix with outstanding properties for the analysis of neutral oligosaccharides. *Anal. Chim. Acta* **2011,** *701* (1), 45-51.

(159) Petkovic, M.; Vujacic, A.; Schiller, J.; Bugarcic, Z.; Savic, J.; Vasic, V. Application of flavonoids - quercetin and rutin - as new matrices for matrix-assisted laser desorption/ionization time-of-flight mass spectrometric analysis of Pt(II) and Pd(II) complexes. *Rapid Commun. Mass Spectrom.* **2009,** *23* (10), 1467-1475.

(160) Wang, X.; Han, J.; Pan, J.; Borchers, C. H. Comprehensive imaging of porcine adrenal gland lipids by MALDI-FTMS using quercetin as a matrix. *Anal. Chem.* **2014,** *86* (1), 638-646.

(161) Petković, M.; Petrović, B.; Savić, J.; Bugarčić, Ž. D.; Dimitrić-Marković, J.; Momić, T.; Vasić, V. Flavonoids as matrices for MALDI-TOF mass spectrometric analysis of transition metal complexes. *Int. J. Mass spectrom.* **2010,** *290* (1), 39-46.

(162) Feng, C. H.; Lu, C. Y. A new matrix for analyzing low molecular mass compounds and its application for determination of carcinogenic areca alkaloids by matrix-assisted laser desorption ionization time-of-flight mass spectrometry. *Anal. Chim. Acta* **2009,** *649* (2), 230-235.

(163) Mugo, S. M.; Bottaro, C. S. Rapid on-plate and one-pot derivatization of carbonyl compounds for enhanced detection by reactive matrix LDI-TOF MS using the tailor-made reactive matrix, 4-dimethylamino-6-(4-methoxy-1-naphthyl)-1,3,5-triazine-2-hydrazine (DMNTH). *J. Mass Spectrom.* **2007,** *42* (2), 206-217.

(164) Schinkovitz, A.; Richomme, P. Usnic acid and its versatility as MALDI matrix. *J. Mass Spectrom.* **2015,** *50* (1), 270-274.

(165) Zhang, Z.; Zhou, L.; Zhao, S.; Deng, H.; Deng, Q. 3-Hydroxycoumarin as a new matrix for matrix-assisted laser desorption/ionization time-of-flight mass spectrometry of DNA. *J. Am. Soc. Mass Spectrom.* **2006,** *17* (12), 1665-1668.

(166) Wang, H.; Wang, Y.; Wang, G.; Hong, L. Matrix-assisted laser-desorption/ionization mass spectrometric imaging of olanzapine in a single hair using esculetin as a matrix. *J. Pharm. Biomed. Anal.* **2017,** *141*, 123-131.

(167) Wang, H.; Dai, B.; Liu, B.; Lu, H. Coumarins as new matrices for matrix-assisted laser-desorption/ionization Fourier transform ion cyclotron resonance mass spectrometric analysis of hydrophobic compounds. *Anal. Chim. Acta* **2015,** *882*, 49-57.

(168) Tang, K.; Taranenko, N. I.; Allman, S. L.; Chen, C. H.; Chang, L. Y.; Jacobson, K. B. Picolinic acid as a matrix for laser mass spectrometry of nucleic acids and proteins. *Rapid Commun. Mass Spectrom.* **1994,** *8* (9), 673-677.

(169) Karas, M.; Hillenkamp, F. Laser desorption ionization of proteins with molecular masses exceeding 10,000 daltons. *Anal. Chem.* **1988,** *60* (20), 2299-2301.

(170) Jiao, J.; Zhang, Y.; Yang, P.; Lu, H. Hydrazinonicotinic acid as a novel matrix for highly sensitive and selective MALDI-MS analysis of oligosaccharides. *Analyst* **2015,** *140* (1), 156-161.

(171) Lorkiewicz, P.; Cecilia Yappert, M. 2-(2-aminoethylamino)-5-nitropyridine as a basic matrix for negative-mode matrix-assisted laser desorption/ionization analysis of phospholipids. *J. Mass Spectrom.* **2009,** *44* (1), 137-143.

(172) Nguyen, H. N.; Tanaka, M.; Komabayashi, G.; Matsui, T. The photobase generator nifedipine as a novel matrix for the detection of polyphenols in matrix-assisted laser desorption/ionization mass spectrometry. *J. Mass Spectrom.* **2016,** *51* (10), 938-946.

(173) Fitzgerald, M. C.; Parr, G. R.; Smith, L. M. Basic matrices for the matrix-assisted laser desorption/ionization mass spectrometry of proteins and oligonucleotides. *Anal. Chem.* **1993,** *65* (22), 3204-3211.

(174) Cheng, S. W.; Chan, T. W. Use of ammonium halides as co-matrices for matrix-assisted laser

desorption/ionization studies of oligonucleotides. *Rapid Commun. Mass Spectrom.* **1996,** *10* (8), 907-910.

(175) Bao, Z.; Yu, D.; Fu, J.; Gu, J.; Xu, J.; Qin, L.; Hu, H.; Yang, C.; Liu, W.; Chen, L.et al. 2-Hydroxy-5-nitro-3-(trifluoromethyl)pyridine as a Novel Matrix for Enhanced MALDI Imaging of Tissue Metabolites. *Anal. Chem.* **2024,** *96* (13), 5160-5169.

(176) Xu, S.; Ye, M.; Xu, D.; Li, X.; Pan, C.; Zou, H. Matrix with high salt tolerance for the analysis of peptide and protein samples by desorption/ionization time-of-flight mass spectrometry. *Anal. Chem.* **2006,** *78* (8), 2593-2599.

(177) Fu, Y.; Xu, S.; Pan, C.; Ye, M.; Zou, H.; Guo, B. A matrix of 3,4-diaminobenzophenone for the analysis of oligonucleotides by matrix-assisted laser desorption/ionization time-of-flight mass spectrometry. *Nucleic Acids Res.* **2006,** *34* (13), e94.

(178) Shi, Y.; Hu, H.; Hao, Q.; Wu, R.; Wang, L.; Qin, L.; Gu, W.; Liu, H.; Jiang, D.; Hong, L.et al. Michler's ethylketone as a novel negative-ion matrix for the enhancement of lipid MALDI tissue imaging. *Chem. Commun. (Camb.)* **2022,** *58* (5), 633-636.

(179) Gu, H.; Ma, K.; Zhao, W.; Qiu, L.; Xu, W. A general purpose MALDI matrix for the analyses of small organic, peptide and protein molecules. *Analyst* **2021,** *146* (12), 4080-4086.

(180) Gimon, M. E.; Kinsel, G. R.; Edmondson, R. D.; Russell, D. H.; Prout, T. R.; Ewald, H. A. Matrix-assisted laser desorption/ionization time-of-flight mass spectrometry of paclitaxel and related taxanes. *J. Nat. Prod.* **1994,** *57* (10), 1404-1410.

(181) Zhang, Y. X.; Zhang, Y. D.; Shi, Y. P. Novel Small Molecule Matrix Screening for Simultaneous MALDI Mass Spectrometry Imaging of Multiple Lipids and Phytohormones. *J. Agric. Food Chem.* **2024,** *72* (12), 6762-6771.

(182) Chen, L. C.; Asakawa, D.; Hori, H.; Hiraoka, K. Matrix-assisted laser desorption/ionization mass spectrometry using a visible laser. *Rapid Commun. Mass Spectrom.* **2007,** *21* (24), 4129-4134.

(183) Xu, H.; Han, M.; Liu, H.; Qin, L.; Chen, L.; Hu, H.; Wu, R.; Yang, C.; Guo, H.; Li, J.et al. 4-Nitrocatechol as a novel matrix for low-molecular-weight compounds in situ detection and imaging in biological tissues by MALDI-MSI. *Chin. Chem. Lett.* **2024,** *35* (6), 109095.

(184) McDonald, H.; Li, Q.; Ashaduzzaman, M.; Zhao, C.; Pan, S.; Szulczewski, G. J.; Liang, Q. Quantitative MALDI-MS and Imaging of Fungicide Pyrimethanil in Strawberries with 2-Nitrophloroglucinol as an Effective Matrix. *J. Am. Soc. Mass Spectrom.* **2024,** *35* (6), 1272-1281.

(185) Shroff, R.; Svatos, A. 1,8-Bis(dimethylamino)naphthalene: a novel superbasic matrix for matrix-assisted laser desorption/ionization time-of-flight mass spectrometric analysis of fatty acids. *Rapid Commun. Mass Spectrom.* **2009,** *23* (15), 2380-2382.

(186) Cao, D.; Wang, Z.; Han, C.; Cui, L.; Hu, M.; Wu, J.; Liu, Y.; Cai, Y.; Wang, H.; Kang, Y. Quantitative detection of trace perfluorinated compounds in environmental water samples by Matrix-assisted Laser Desorption/Ionization-Time of Flight Mass Spectrometry with 1,8-bis(tetramethylguanidino)-naphthalene as matrix. *Talanta* **2011,** *85* (1), 345-352.

(187) Liu, H.; Zhou, Y.; Wang, J.; Xiong, C.; Xue, J.; Zhan, L.; Nie, Z. N-Phenyl-2-naphthylamine as a Novel MALDI Matrix for Analysis and in Situ Imaging of Small Molecules. *Anal. Chem.* **2018,** *90* (1), 729-736.

(188) Napagoda, M.; Rulisek, L.; Jancarik, A.; Klivar, J.; Samal, M.; Stara, I. G.; Stary, I.; Solinova, V.; Kasicka, V.; Svatos, A. Azahelicene Superbases as MAILD Matrices for Acidic Analytes. *Chempluschem* **2013,** *78* (9), 937-942.

(189) Giampa, M.; Lissel, M. B.; Patschkowski, T.; Fuchser, J.; Hans, V. H.; Gembruch, O.; Bednarz, H.;

Niehaus, K. Maleic anhydride proton sponge as a novel MALDI matrix for the visualization of small molecules (<250 m/z) in brain tumors by routine MALDI ToF imaging mass spectrometry. *Chem. Commun. (Camb.)* **2016,** *52* (63), 9801-9804.

(190) Calvano, C. D.; Cataldi, T. R.; Kogel, J. F.; Monopoli, A.; Palmisano, F.; Sundermeyer, J. Superbasic alkyl-substituted bisphosphazene proton sponges: a new class of deprotonating matrices for negative ion matrix-assisted ionization/laser desorption mass spectrometry of low molecular weight hardly ionizable analytes. *Rapid Commun. Mass Spectrom.* **2016,** *30* (14), 1680-1686.

(191) Chen, R.; Xu, W.; Xiong, C.; Zhou, X.; Xiong, S.; Nie, Z.; Mao, L.; Chen, Y.; Chang, H. C. High-salt-tolerance matrix for facile detection of glucose in rat brain microdialysates by MALDI mass spectrometry. *Anal. Chem.* **2012,** *84* (1), 465-469.

(192) Hou, J.; Chen, S.; Zhang, N.; Liu, H.; Wang, J.; He, Q.; Wang, J.; Xiong, S.; Nie, Z. Organic salt NEDC (N-naphthylethylenediamine dihydrochloride) assisted laser desorption ionization mass spectrometry for identification of metal ions in real samples. *Analyst* **2014,** *139* (13), 3469-3475.

(193) Chen, R.; Chen, S.; Xiong, C.; Ding, X.; Wu, C. C.; Chang, H. C.; Xiong, S.; Nie, Z. N-(1-naphthyl) ethylenediamine dinitrate: a new matrix for negative ion MALDI-TOF MS analysis of small molecules. *J. Am. Soc. Mass Spectrom.* **2012,** *23* (9), 1454-1460.

(194) He, Q.; Chen, S.; Wang, J.; Hou, J.; Wang, J.; Xiong, S.; Nie, Z. 1-naphthylhydrazine hydrochloride: a new matrix for the quantification of glucose and homogentisic acid in real samples by MALDI-TOF MS. *Clin. Chim. Acta* **2013,** *420*, 94-98.

(195) Tang, X.; Vertes, A.; Dreifuss, P. A. New matrices and accelerating voltage effects in matrix-assisted laser desorption/ionization of synthetic polymers. *Rapid Commun. Mass Spectrom.* **1995,** *9* (12), 1141-1147.

(196) Liu, R.; Long, L.; Yan, X.; Guo, H.; Wu, R.; Hu, H.; Xu, H.; Hao, Q.; Qin, L.; Chen, L.et al. Enhancement of lipid MALDI tissue imaging using 1,4-dihydroxy-2-naphthoic acid as a novel matrix. *Sci. China Chem.* **2025**.

(197) Li, B.; Sun, R.; Gordon, A.; Ge, J.; Zhang, Y.; Li, P.; Yang, H. 3-Aminophthalhydrazide (Luminol) As a Matrix for Dual-Polarity MALDI MS Imaging. *Anal. Chem.* **2019,** *91* (13), 8221-8228.

(198) Tang, W.; Gordon, A.; Wang, F.; Chen, Y.; Li, B. Hydralazine as a Versatile and Universal Matrix for High-Molecular Coverage and Dual-Polarity Matrix-Assisted Laser Desorption/Ionization Mass Spectrometry Imaging. *Anal. Chem.* **2021,** *93* (26), 9083-9093.

(199) Ling, L.; Yu, S.; Ding, C. 4-Hydrazinoquinazoline acting as a reactive matrix for the rapid and sensitive analysis of neutral and sialylated glycans using MALDI MS. *Analyst* **2021,** *146* (22), 6840-6845.

(200) Chen, Y.; Hu, D.; Zhao, L.; Tang, W.; Li, B. Unraveling metabolic alterations in transgenic mouse model of Alzheimer's disease using MALDI MS imaging with 4-aminocinnoline-3-carboxamide matrix. *Anal. Chim. Acta* **2022,** *1192*, 339337.

(201) Bai, J.; Liang, X.; Liu, Y. H.; Zhu, Y.; Lubman, D. M. Characterization of two new matrices for matrix-assisted laser desorption/ionization mass spectrometry. *Rapid Commun. Mass Spectrom.* **1996,** *10* (7), 839-844.

(202) Galesio, M.; Rial-Otero, R.; Capelo-Martinez, J. L. Comparative study of matrices for their use in the rapid screening of anabolic steroids by matrix-assisted laser desorption/ionisation time-of-flight mass spectrometry. *Rapid Commun. Mass Spectrom.* **2009,** *23* (12), 1783-1791.

(203) Nonami, H.; Wu, F.; Thummel, R. P.; Fukuyama, Y.; Yamaoka, H.; Erra-Balsells, R. Evaluation of pyridoindoles, pyridylindoles and pyridylpyridoindoles as matrices for ultraviolet matrix-assisted laser desorption/ionization time-of-flight mass spectrometry. *Rapid Commun. Mass Spectrom.* **2001,** *15* (23), 2354-2373.

(204) Liang, Q.; Mondal, P.; Li, Q.; Maqbool, T.; Zhao, C.; Jiang, D.; Szulczewski, G. J.; Wijeratne, G. B. Nitro Indole Derivatives as Novel Dual-Polarity Matrices for MALDI Mass Spectrometry and Imaging with Broad Applications. *Anal. Chem.* **2024,** *96* (4), 1668-1677.

(205) Chen, P.; Baker, A. G.; Novotny, M. V. The use of osazones as matrices for the matrix-assisted laser desorption/ionization mass spectrometry of carbohydrates. *Anal. Biochem.* **1997,** *244* (1), 144-151.

(206) Liu, Y.; Chen, L.; Qin, L.; Han, M.; Li, J.; Luo, F.; Xue, K.; Feng, J.; Zhou, Y.; Wang, X. Enhanced in situ detection and imaging of lipids in biological tissues by using 2,3-dicyanohydroquinone as a novel matrix for positive-ion MALDI-MS imaging. *Chem. Commun. (Camb.)* **2019,** *55* (83), 12559-12562.

(207) Osaka, I.; Sakai, M.; Takayama, M. 5-Amino-1-naphthol, a novel 1,5-naphthalene derivative matrix suitable for matrix-assisted laser desorption/ionization in-source decay of phosphorylated peptides. *Rapid Commun. Mass Spectrom.* **2013,** *27* (1), 103-108.

(208) Calvano, C. D.; Carulli, S.; Palmisano, F. 1H-pteridine-2,4-dione (lumazine): a new MALDI matrix for complex (phospho)lipid mixtures analysis. *Anal. Bioanal. Chem.* **2010,** *398* (1), 499-507.

(209) Musharraf, S. G.; Ameer, M.; Ali, A. MALDI-MS analysis and theoretical evaluation of olanzapine as a UV laser desorption ionization (LDI) matrix. *J. Pharm. Biomed. Anal.* **2017,** *132*, 190-194.

(210) Yan, J.; Xie, W.; Jin, Y.; Cai, Z.; Lin, Z. 1,4-Dioxo-1,2,3,4-tetrahydrophthalazine-6-carboxylic acid as a novel MALDI matrix for enhanced analysis of metabolites induced by imidacloprid exposure. *Talanta* **2025,** *286*, 127536.

(211) Ibrahim, H.; Jurcic, K.; Wang, J. S.; Whitehead, S. N.; Yeung, K. K. 1,6-Diphenyl-1,3,5-hexatriene (DPH) as a Novel Matrix for MALDI MS Imaging of Fatty Acids, Phospholipids, and Sulfatides in Brain Tissues. *Anal. Chem.* **2017,** *89* (23), 12828-12836.

(212) Kimura, S.; Fujisaka, A.; Obika, S. Nucleobase derivatives induce in-source decay of oligonucleotides as new matrix-assisted laser desorption/ionization matrices. *Rapid Commun. Mass Spectrom.* **2020,** *34* (6), e8620.

(213) Ling, L.; Li, Y.; Wang, S.; Guo, L.; Xiao, C.; Chen, X.; Guo, X. DBDA as a Novel Matrix for the Analyses of Small Molecules and Quantification of Fatty Acids by Negative Ion MALDI-TOF MS. *J. Am. Soc. Mass Spectrom.* **2018,** *29* (4), 704-710.

(214) Wu, R.; Jiang, D.; Hu, H.; Yang, C.; Qin, L.; Chen, L.; Hu, Z.; Xu, H.; Li, J.; Liu, H.et al. 4-Aminoazobenzene: A novel negative ion matrix for enhanced MALDI tissue imaging of metabolites. *Chin. Chem. Lett.* **2024,** *35* (11), 109624.

(215) Song, F. Quinaldic acid as a new matrix for matrix-assisted laser desorption/ionization of nucleic acids. *Rapid Commun. Mass Spectrom.* **2003,** *17* (15), 1802-1807.

(216) Zhao, X.; Wang, H.; Liu, Y.; Ou, R.; Liu, Y.; Li, X.; Pan, Y. Lignin as a MALDI matrix for small molecules: a proof of concept. *Analyst* **2021,** *146* (24), 7573-7582.

(217) Sun, C.; Liu, W.; Mu, Y.; Wang, X. 1,1'-binaphthyl-2,2'-diamine as a novel MALDI matrix to enhance the in situ imaging of metabolic heterogeneity in lung cancer. *Talanta* **2020,** *209*, 120557.

(218) Godfrey, A. R.; Brenton, A. G.; Forbes-Robertson, S. Investigating dansyl compounds as novel matrices for matrix-assisted laser desorption/ionisation proteomics. *Rapid Commun. Mass Spectrom.* **2010,** *24* (1), 160-164.

(219) Javorek, M.; Hendrych, M.; Ondrakova, K.; Preisler, J.; Bednarik, A. Staining Tissues with Basic Blue 7: A New Dual-Polarity Matrix for MALDI Mass Spectrometry Imaging. *Anal. Chem.* **2025,** *97* (5), 2828-2836.

(220) Zhang, L. K.; Gross, M. L. Location of abasic sites in oligodeoxynucleotides by tandem mass spectrometry and by a chemical cleavage initiated by an unusual reaction of the ODN with MALDI

matrix. *J. Am. Soc. Mass Spectrom.* **2002,** *13* (12), 1418-1426.
(221) Ling, L.; Xiao, C.; Ma, Y.; Jiang, L.; Wang, S.; Guo, L.; Jiang, S.; Guo, X. 2-Phenyl-3-(p-aminophenyl) Acrylonitrile: A Reactive Matrix for Sensitive and Selective Analysis of Glycans by MALDI-MS. *Anal. Chem.* **2019,** *91* (14), 8801-8807.
(222) Mugo, S. M.; Bottaro, C. S. Rapid analysis of alpha-dicarbonyl compounds by laser desorption/ionization mass spectrometry using 9-(3,4-diaminophenyl)acridine (DAA) as a reactive matrix. *Rapid Commun. Mass Spectrom.* **2008,** *22* (8), 1087-1093.
(223) Liao, J.; Wang, H.; Zhou, S.; Liu, Y.; Zhao, X.; Pan, Y. alpha-Cyano-3-aminocinnamic acid: A novel reactive matrix for qualitative and quantitative analysis of plant N-glycans by MALDI-MS. *Anal. Chim. Acta* **2023,** *1283*, 341970.
(224) Slyundina, M. S.; Polovkov, N. Y.; Borisov, R. S.; Zaikin, V. G. Tryptamine: a Reactive Matrix for MALDI Mass Spectrometry. *J. Anal. Chem.* **2018,** *72* (13), 1295-1299.
(225) Liao, J.; Wang, H.; Zhou, S.; Feng, H.; Liu, Y.; Zhao, X.; Pan, Y. 2-Nitro-4-Carboxyphenylhydrazine and 2,4-Dicarboxylphenylhydrazine as a Pair of Novel Reactive MALDI Matrices for Rapid and Accurate Profiling of N-Glycome in Dual Ion Modes. *Anal. Chem.* **2024,** *96* (41), 16145-16153.
(226) Shigeri, Y.; Yasuda, A.; Sakai, M.; Ikeda, S.; Arakawa, R.; Sato, H.; Kinumi, T. Hydrazide and hydrazine reagents as reactive matrices for matrix-assisted laser desorption/ionization mass spectrometry to detect steroids with carbonyl groups. *Eur. J. Mass Spectrom. (Chichester)* **2015,** *21* (2), 79-90.
(227) Shigeri, Y.; Kamimura, T.; Ando, M.; Uegaki, K.; Sato, H.; Tani, F.; Arakawa, R.; Kinumi, T. 2-Hydrazinoquinoline: a reactive matrix for matrix-assisted laser desorption/ionization mass spectrometry to detect gaseous carbonyl compounds. *Eur. J. Mass Spectrom. (Chichester)* **2016,** *22* (2), 83-90.
(228) Wang, H.; Wang, H.; Zhang, L.; Zhang, J.; Guo, Y. N-Alkylpyridinium isotope quaternization for matrix-assisted laser desorption/ionization Fourier transform mass spectrometric analysis of cholesterol and fatty alcohols in human hair. *Anal. Chim. Acta* **2011,** *690* (1), 1-9.
(229) Jiang, K.; Aloor, A.; Qu, J.; Xiao, C.; Wu, Z.; Ma, C.; Zhang, L.; Wang, P. G. Rapid and sensitive MALDI MS analysis of oligosaccharides by using 2-hydrazinopyrimidine as a derivative reagent and co-matrix. *Anal. Bioanal. Chem.* **2017,** *409* (2), 421-429.
(230) Wang, H.; Gao, Y.; He, Q.; Liao, J.; Zhou, S.; Liu, Y.; Guo, C.; Li, X.; Zhao, X.; Pan, Y. 2-Hydrazinoterephthalic Acid as a Novel Negative-Ion Matrix-Assisted Laser Desorption/Ionization Matrix for Qualitative and Quantitative Matrix-Assisted Laser Desorption/Ionization-Mass Spectrometry Analysis of N-Glycans in Peach Allergy Research. *J. Agric. Food Chem.* **2023,** *71* (1), 952-962.
(231) Shariatgorji, M.; Nilsson, A.; Kallback, P.; Karlsson, O.; Zhang, X.; Svenningsson, P.; Andren, P. E. Pyrylium Salts as Reactive Matrices for MALDI-MS Imaging of Biologically Active Primary Amines. *J. Am. Soc. Mass Spectrom.* **2015,** *26* (6), 934-939.
(232) Shariatgorji, M.; Nilsson, A.; Fridjonsdottir, E.; Vallianatou, T.; Kallback, P.; Katan, L.; Savmarker, J.; Mantas, I.; Zhang, X.; Bezard, E.et al. Comprehensive mapping of neurotransmitter networks by MALDI-MS imaging. *Nat. Methods* **2019,** *16* (10), 1021-1028.
(233) Matveeva, M. D.; Zimens, M. E.; Topolyan, A. P.; Zhilyaev, D. I.; Voskressenskaya, D. L.; Krivosheina, M. S.; Borisov, R. S.; Zaikin, V. G. 1-Pyrenylboronic Acid as a Reactive Matrix for the Analysis of Polyfunctional Compounds by MALDI Mass Spectrometry: New Possibilities. *J. Anal. Chem.* **2023,** *77* (14), 1729-1736.
(234) Addy, P. S.; Bhattacharya, A.; Mandal, S. M.; Basak, A. Label-assisted laser desorption/ionization mass spectrometry (LA-LDI-MS): an emerging technique for rapid detection of ubiquitous cis-1,2-diol functionality. *RSC Adv.* **2014,** *4* (87), 46555-46560.

(235) Kaya, I.; Brulls, S. M.; Dunevall, J.; Jennische, E.; Lange, S.; Martensson, J.; Ewing, A. G.; Malmberg, P.; Fletcher, J. S. On-Tissue Chemical Derivatization of Catecholamines Using 4-(N-Methyl)pyridinium Boronic Acid for ToF-SIMS and LDI-ToF Mass Spectrometry Imaging. *Anal. Chem.* **2018,** *90* (22), 13580-13590.

(236) Monopoli, A.; Calvano, C. D.; Nacci, A.; Palmisano, F. Boronic acid chemistry in MALDI MS: a step forward in designing a reactive matrix with molecular recognition capabilities. *Chem. Commun. (Camb.)* **2014,** *50* (33), 4322-4324.

(237) Chen, J.; Huang, H.; Ouyang, D.; Lin, J.; Chen, Z.; Cai, Z.; Lin, Z. A reactive matrix for in situ chemical derivatisation and specific detection of cis-diol compounds by matrix-assisted laser desorption/ionisation mass spectrometry. *Analyst* **2023,** *148* (21), 5402-5406.

(238) Cheema, E. K.; Bhattacharya, P.; Singha, M.; Basak, A. Use of p-methoxy cinnamaldehyde in label-assisted laser desorption/ionization mass spectrometry (LALDI-MS) for detection of different classes of primary amines, amino acids and neurotransmitters. *Int. J. Mass spectrom.* **2024,** *505*, 117307.

(239) Qin, Z. N.; Chen, J. M.; Li, J. W.; Zhen, Z. P.; Chen, Q. Z.; Zhang, Z. Q.; Wang, G. H.; Gao, Y. F. Rapid absolute quantification of glucose and fructose isomers in honey using a boronic acid-based reactive matrix by MALDI-TOF/TOF tandem mass spectrometry. *Food Chem.* **2025,** *477*, 143623.

(240) Waldchen, F.; Spengler, B.; Heiles, S. Reactive Matrix-Assisted Laser Desorption/Ionization Mass Spectrometry Imaging Using an Intrinsically Photoreactive Paterno-Buchi Matrix for Double-Bond Localization in Isomeric Phospholipids. *J. Am. Chem. Soc.* **2019,** *141* (30), 11816-11820.

(241) Waldchen, F.; Mohr, F.; Wagner, A. H.; Heiles, S. Multifunctional Reactive MALDI Matrix Enabling High-Lateral Resolution Dual Polarity MS Imaging and Lipid C horizontal lineC Position-Resolved MS(2) Imaging. *Anal. Chem.* **2020,** *92* (20), 14130-14138.

(242) Asakawa, D.; Osaka, I. Direct MALDI-MS analysis of the disulfide bonds in peptide using thiosalicylic acid as a reactive matrix. *J. Mass Spectrom.* **2017,** *52* (2), 127-131.

(243) Boutaghou, M. N.; Cole, R. B. 9,10-Diphenylanthracene as a matrix for MALDI-MS electron transfer secondary reactions. *J. Mass Spectrom.* **2012,** *47* (8), 995-1003.

(244) Ijuin, H. K.; Yamada, M.; Ohashi, M.; Watanabe, N.; Matsumoto, M. Electron-transfer-induced decomposition of 1,2-dioxetanes in negative-mode matrix- assisted laser desorption/ionization time-of-flight mass spectrometry. *Eur. J. Mass Spectrom. (Chichester)* **2008,** *14* (1), 17-25.

(245) Przybilla, L.; Brand, J. D.; Yoshimura, K.; Rader, H. J.; Mullen, K. MALDI-TOF mass spectrometry of insoluble giant polycyclic aromatic hydrocarbons by a new method of sample preparation. *Anal. Chem.* **2000,** *72* (19), 4591-4597.

(246) Asakawa, D.; Chen, L. C.; Hiraoka, K. Negative-mode MALDI mass spectrometry for the analysis of pigments using tetrathiafulvalene as a matrix. *J. Mass Spectrom.* **2008,** *43* (11), 1494-1501.

(247) Calvano, C. D.; Ventura, G.; Cataldi, T. R.; Palmisano, F. Improvement of chlorophyll identification in foodstuffs by MALDI ToF/ToF mass spectrometry using 1,5-diaminonaphthalene electron transfer secondary reaction matrix. *Anal. Bioanal. Chem.* **2015,** *407* (21), 6369-6379.

(248) Calvano, C. D.; Ventura, G.; Trotta, M.; Bianco, G.; Cataldi, T. R.; Palmisano, F. Electron-Transfer Secondary Reaction Matrices for MALDI MS Analysis of Bacteriochlorophyll a in Rhodobacter sphaeroides and Its Zinc and Copper Analogue Pigments. *J. Am. Soc. Mass Spectrom.* **2017,** *28* (1), 125-135.

(249) Castellanos-Garcia, L. J.; Agudelo, B. C.; Rosales, H. F.; Cely, M.; Ochoa-Puentes, C.; Blanco-Tirado, C.; Sierra, C. A.; Combariza, M. Y. Oligo p-Phenylenevinylene Derivatives as Electron Transfer Matrices for UV-MALDI. *J. Am. Soc. Mass Spectrom.* **2017,** *28* (12), 2548-2560.

(250) Luo, Y.; Gao, A.; Zhou, X.; Hu, X.; Chang, S.; Liu, L.; Zhang, L.; Gui, Z.; Yan, X.; Huang, X.et al. Imaging the Redox Network of Metabolic Acids with Matrix Assisted Laser Desorption and Photoinduced Electron Transfer Ionization Mass Spectrometry. *Anal. Chem.* **2025,** *97* (33), 18167-18176.

(251) Macha, S. F.; Limbach, P. A.; Savickas, P. J. Application of nonpolar matrices for the analysis of low molecular weight nonpolar synthetic polymers by matrix-assisted laser desorption/ionization time-of-flight mass spectrometry. *J. Am. Soc. Mass Spectrom.* **2000,** *11* (8), 731-737.

(252) McCarley, T. D.; McCarley, R. L.; Limbach, P. A. Electron-Transfer Ionization in Matrix-Assisted Laser Desorption/Ionization Mass Spectrometry. *Anal. Chem.* **1998,** *70* (20), 4376-4379.

(253) Ma, G.; Zhao, X.; Guo, C.; Li, S.; Liu, Y.; He, Q.; Chen, K.; Pan, Y. Glycosylamines-based reactive matrix designed for imaging acidity in Ponkan fruit using matrix assisted laser desorption/ionization mass spectrometry imaging. *Anal. Chim. Acta* **2018,** *1041*, 78-86.

(254) Fulop, A.; Bausbacher, T.; Rizzo, S.; Zhou, Q.; Gillandt, H.; Hopf, C.; Rittner, M. New Derivatization Reagent for Detection of free Thiol-groups in Metabolites and Proteins in Matrix-Assisted Laser Desorption/Ionization Mass Spectrometry Imaging. *Anal. Chem.* **2020,** *92* (9), 6224-6228.

(255) Lascoux, D.; Paramelle, D.; Subra, G.; Heymann, M.; Geourjon, C.; Martinez, J.; Forest, E. Discrimination and selective enhancement of signals in the MALDI mass spectrum of a protein by combining a matrix-based label for lysine residues with a neutral matrix. *Angew. Chem. Int. Ed. Engl.* **2007,** *46* (29), 5594-5597.

(256) Wang, S.; Xiao, Z.; Xiao, C.; Wang, H.; Wang, B.; Li, Y.; Chen, X.; Guo, X. (E)-Propyl alpha-Cyano-4-Hydroxyl Cinnamylate: A High Sensitive and Salt Tolerant Matrix for Intact Protein Profiling by MALDI Mass Spectrometry. *J. Am. Soc. Mass Spectrom.* **2016,** *27* (4), 709-718.

(257) Jaskolla, T. W.; Lehmann, W. D.; Karas, M. 4-Chloro-alpha-cyanocinnamic acid is an advanced, rationally designed MALDI matrix. *Proc. Natl. Acad. Sci. U. S. A.* **2008,** *105* (34), 12200-12205.

(258) Jaskolla, T.; Fuchs, B.; Karas, M.; Schiller, J. The new matrix 4-chloro-alpha-cyanocinnamic acid allows the detection of phosphatidylethanolamine chloramines by MALDI-TOF mass spectrometry. *J. Am. Soc. Mass Spectrom.* **2009,** *20* (5), 867-874.

(259) Selman, M. H.; Hoffmann, M.; Zauner, G.; McDonnell, L. A.; Balog, C. I.; Rapp, E.; Deelder, A. M.; Wuhrer, M. MALDI-TOF-MS analysis of sialylated glycans and glycopeptides using 4-chloro-alpha-cyanocinnamic acid matrix. *Proteomics* **2012,** *12* (9), 1337-1348.

(260) Calvano, C. D.; Ventura, G.; Palmisano, F.; Cataldi, T. R. 4-Chloro-alpha-cyanocinnamic acid is an efficient soft matrix for cyanocobalamin detection in foodstuffs by matrix-assisted laser desorption/ionization mass spectrometry (MALDI MS). *J. Mass Spectrom.* **2016,** *51* (9), 841-848.

(261) Ventura, G.; Arnesano, F.; Calvano, C. D.; Palmisano, F.; Cataldi, T. R. I. Cyanocobalamin conjugates of cisplatin and diaminocyclohexane-platinum(ii): matrix-assisted laser desorption ionization mass spectrometry characterization using 4-chloro-α-cyanocinnamic acid as the matrix. *RSC Adv.* **2017,** *7* (85), 53658-53666.

(262) Porta, T.; Grivet, C.; Knochenmuss, R.; Varesio, E.; Hopfgartner, G. Alternative CHCA-based matrices for the analysis of low molecular weight compounds by UV-MALDI-tandem mass spectrometry. *J. Mass Spectrom.* **2011,** *46* (2), 144-152.

(263) Monopoli, A.; Ventura, G.; Aloia, A.; Ciriaco, F.; Nacci, A.; Cataldi, T. R. I.; Calvano, C. D. Synthesis and Investigation of Novel CHCA-Derived Matrices for Matrix-Assisted Laser Desorption/Ionization Mass Spectrometric Analysis of Lipids. *Molecules* **2022,** *27* (8), 2565.

(264) Teuber, K.; Schiller, J.; Fuchs, B.; Karas, M.; Jaskolla, T. W. Significant sensitivity improvements by matrix optimization: a MALDI-TOF mass spectrometric study of lipids from hen egg yolk. *Chem. Phys.*

Lipids **2010,** *163* (6), 552-560.
(265) Monopoli, A.; Nacci, A.; Cataldi, T. R. I.; Calvano, C. D. Synthesis and Matrix Properties of alpha-Cyano-5-phenyl-2,4-pentadienic Acid (CPPA) for Intact Proteins Analysis by Matrix-Assisted Laser Desorption/Ionization Mass Spectrometry. *Molecules* **2020,** *25* (24), 6054.
(266) Shariatgorji, M.; Nilsson, A.; Goodwin, R. J.; Svenningsson, P.; Schintu, N.; Banka, Z.; Kladni, L.; Hasko, T.; Szabo, A.; Andren, P. E. Deuterated matrix-assisted laser desorption ionization matrix uncovers masked mass spectrometry imaging signals of small molecules. *Anal. Chem.* **2012,** *84* (16), 7152-7157.
(267) Fulop, A.; Porada, M. B.; Marsching, C.; Blott, H.; Meyer, B.; Tambe, S.; Sandhoff, R.; Junker, H. D.; Hopf, C. 4-Phenyl-alpha-cyanocinnamic acid amide: screening for a negative ion matrix for MALDI-MS imaging of multiple lipid classes. *Anal. Chem.* **2013,** *85* (19), 9156-9163.
(268) Tambe, S.; Blott, H.; Fulop, A.; Spang, N.; Flottmann, D.; Brase, S.; Hopf, C.; Junker, H. D. Structure-performance relationships of phenyl cinnamic acid derivatives as MALDI-MS matrices for sulfatide detection. *Anal. Bioanal. Chem.* **2017,** *409* (6), 1569-1580.
(269) Dufresne, M.; Migas, L. G.; Djambazova, K. V.; Colley, M. E.; Van de Plas, R.; Spraggins, J. M. Aminated cinnamic acid analogs as dual polarity matrices for high spatial resolution MALDI imaging mass spectrometry. *Anal. Chim. Acta* **2025,** *1371*, 344423.
(270) Cvacka, J.; Svatos, A. Matrix-assisted laser desorption/ionization analysis of lipids and high molecular weight hydrocarbons with lithium 2,5-dihydroxybenzoate matrix. *Rapid Commun. Mass Spectrom.* **2003,** *17* (19), 2203-2207.
(271) Fukuyama, Y.; Nakajima, C.; Furuichi, K.; Taniguchi, K.; Kawabata, S.; Izumi, S.; Tanaka, K. Alkylated trihydroxyacetophenone as a MALDI matrix for hydrophobic peptides. *Anal. Chem.* **2013,** *85* (20), 9444-9448.
(272) Huang, P.; Huang, C. Y.; Lin, T. C.; Lin, L. E.; Yang, E.; Lee, C.; Hsu, C. C.; Chou, P. T. Toward the Rational Design of Universal Dual Polarity Matrix for MALDI Mass Spectrometry. *Anal. Chem.* **2020,** *92* (10), 7139-7145.
(273) Horka, P.; Vrkoslav, V.; Hanus, R.; Peckova, K.; Cvacka, J. New MALDI matrices based on lithium salts for the analysis of hydrocarbons and wax esters. *J. Mass Spectrom.* **2014,** *49* (7), 628-638.
(274) Al Ghafly, H.; Siraj, N.; Das, S.; Regmi, B. P.; Magut, P. K.; Galpothdeniya, W. I.; Murray, K. K.; Warner, I. M. GUMBOS matrices of variable hydrophobicity for matrix-assisted laser desorption/ionization mass spectrometry. *Rapid Commun. Mass Spectrom.* **2014,** *28* (21), 2307-2314.
(275) Liu, H.; Chen, R.; Wang, J.; Chen, S.; Xiong, C.; Wang, J.; Hou, J.; He, Q.; Zhang, N.; Nie, Z.et al. 1,5-Diaminonaphthalene hydrochloride assisted laser desorption/ionization mass spectrometry imaging of small molecules in tissues following focal cerebral ischemia. *Anal. Chem.* **2014,** *86* (20), 10114-10121.
(276) Yao, C.; Niu, C.; Na, N.; He, D.; Ouyang, J. Aggregation-induced emission compounds as new assisted matrices for laser desorption/ionization time-of-flight mass spectrometry. *Anal. Chim. Acta* **2015,** *853*, 375-383.
(277) Weißflog, J.; Svatoš, A. 1,8-Di(piperidinyl)-naphthalene – rationally designed MAILD/MALDI matrix for metabolomics and imaging mass spectrometry. *RSC Adv.* **2016,** *6* (79), 75073-75081.
(278) Kasai, H.; Nakakoshi, M.; Sugita, T.; Matsuoka, M.; Yamazaki, Y.; Unno, Y.; Nakajima, H.; Fujiwake, H.; Tsubuki, M. Investigation of 5-(3-Trifluoromethylbenzylidene)thiazolidine-2,4-dione as a Matrix for Analyses of Biogenic Monoamine Transmitters Using MALDI-MS. *Anal. Sci.* **2016,** *32* (8), 907-910.
(279) Yang, J.; Norris, J. L.; Caprioli, R. Novel vacuum stable ketone-based matrices for high spatial resolution MALDI imaging mass spectrometry. *J. Mass Spectrom.* **2018,** *53* (10), 1005-1012.
(280) Zhou, Q.; Rizzo, S.; Oetjen, J.; Fulop, A.; Rittner, M.; Gillandt, H.; Hopf, C. A Caged In-Source Laser-

Cleavable MALDI Matrix with High Vacuum Stability for Extended MALDI-MS Imaging. *Angew. Chem. Int. Ed. Engl.* **2023,** *62* (22), e202217047.

(281) Chen, Y.; Che, J.; Wang, J.; Tuo, Y.; Zhao, H.; Chen, Y.; Sai, L.; Zhao, H.; Zhang, R. Functional Melanin Nanoparticles-Assisted Laser Desorption Ionization Mass Spectrometry for High-Sensitivity Detection of TBBPA and TBBPS Contaminations in Animal-Derived Foodstuffs. *J. Agric. Food Chem.* **2024,** *72* (12), 6744-6753.

(282) Zabet-Moghaddam, M.; Heinzle, E.; Tholey, A. Qualitative and quantitative analysis of low molecular weight compounds by ultraviolet matrix-assisted laser desorption/ionization mass spectrometry using ionic liquid matrices. *Rapid Commun. Mass Spectrom.* **2004,** *18* (2), 141-148.

(283) Meriaux, C.; Franck, J.; Wisztorski, M.; Salzet, M.; Fournier, I. Liquid ionic matrixes for MALDI mass spectrometry imaging of lipids. *J. Proteomics* **2010,** *73* (6), 1204-1218.

(284) Abdelhamid, H. N.; Gopal, J.; Wu, H. F. Synthesis and application of ionic liquid matrices (ILMs) for effective pathogenic bacteria analysis in matrix assisted laser desorption/ionization (MALDI-MS). *Anal. Chim. Acta* **2013,** *767*, 104-111.

(285) Schnoll-Bitai, I.; Ullmer, R.; Hrebicek, T.; Rizzi, A.; Lacik, I. Characterization of the molecular mass distribution of pullulans by matrix-assisted laser desorption/ionization time-of-flight mass spectrometry using 2,5-dihydroxybenzoic acid butylamine (DHBB) as liquid matrix. *Rapid Commun. Mass Spectrom.* **2008,** *22* (19), 2961-2970.

(286) Mank, M.; Stahl, B.; Boehm, G. 2,5-Dihydroxybenzoic acid butylamine and other ionic liquid matrixes for enhanced MALDI-MS analysis of biomolecules. *Anal. Chem.* **2004,** *76* (10), 2938-2950.

(287) Snovida, S. I.; Perreault, H. A 2,5-dihydroxybenzoic acid/N,N-dimethylaniline matrix for the analysis of oligosaccharides by matrix-assisted laser desorption/ionization mass spectrometry. *Rapid Commun. Mass Spectrom.* **2007,** *21* (22), 3711-3715.

(288) Zhao, X.; Shen, S.; Wu, D.; Cai, P.; Pan, Y. Novel ionic liquid matrices for qualitative and quantitative detection of carbohydrates by matrix assisted laser desorption/ionization mass spectrometry. *Anal. Chim. Acta* **2017,** *985*, 114-120.

(289) Barada, E.; Hinou, H. BOA/DHB/Na: An Efficient UV-MALDI Matrix for High-Sensitivity and Auto-Tagging Glycomics. *Int. J. Mol. Sci.* **2022,** *23* (20), 12510.

(290) Jones, J. J.; Batoy, S. M.; Wilkins, C. L.; Liyanage, R.; Lay, J. O., Jr. Ionic liquid matrix-induced metastable decay of peptides and oligonucleotides and stabilization of phospholipids in MALDI FTMS analyses. *J. Am. Soc. Mass Spectrom.* **2005,** *16* (12), 2000-2008.

(291) Tholey, A.; Zabet-Moghaddam, M.; Heinzle, E. Quantification of peptides for the monitoring of protease-catalyzed reactions by matrix-assisted laser desorption/ionization mass spectrometry using ionic liquid matrixes. *Anal. Chem.* **2006,** *78* (1), 291-297.

(292) Lemaire, R.; Tabet, J. C.; Ducoroy, P.; Hendra, J. B.; Salzet, M.; Fournier, I. Solid ionic matrixes for direct tissue analysis and MALDI imaging. *Anal. Chem.* **2006,** *78* (3), 809-819.

(293) Liu, Q.; He, L. Ionic matrix for matrix-enhanced surface-assisted laser desorption ionization mass spectrometry imaging (ME-SALDI-MSI). *J. Am. Soc. Mass Spectrom.* **2009,** *20* (12), 2229-2237.

(294) Calvano, C. D.; Carulli, S.; Palmisano, F. Aniline/alpha-cyano-4-hydroxycinnamic acid is a highly versatile ionic liquid for matrix-assisted laser desorption/ionization mass spectrometry. *Rapid Commun. Mass Spectrom.* **2009,** *23* (11), 1659-1668.

(295) Bonnel, D.; Franck, J.; Meriaux, C.; Salzet, M.; Fournier, I. Ionic matrices pre-spotted matrix-assisted laser desorption/ionization plates for patient maker following in course of treatment, drug titration, and MALDI mass spectrometry imaging. *Anal. Biochem.* **2013,** *434* (1), 187-198.

(296) Li, Y. L.; Gross, M. L.; Hsu, F. F. Ionic-liquid matrices for improved analysis of phospholipids by MALDI-TOF mass spectrometry. *J. Am. Soc. Mass Spectrom.* **2005,** *16* (5), 679-682.

(297) Chan, K.; Lanthier, P.; Liu, X.; Sandhu, J. K.; Stanimirovic, D.; Li, J. MALDI mass spectrometry imaging of gangliosides in mouse brain using ionic liquid matrix. *Anal. Chim. Acta* **2009,** *639* (1-2), 57-61.

(298) Yoon, D.; Lee, D.; Lee, J. H.; Cha, S.; Oh, H. B. Quantitative analysis of polyhexamethylene guanidine (PHMG) oligomers via matrix-assisted laser desorption/ionization time-of-flight mass spectrometry with an ionic-liquid matrix. *Rapid Commun. Mass Spectrom.* **2015,** *29* (2), 213-219.

(299) Laremore, T. N.; Murugesan, S.; Park, T. J.; Avci, F. Y.; Zagorevski, D. V.; Linhardt, R. J. Matrix-assisted laser desorption/ionization mass spectrometric analysis of uncomplexed highly sulfated oligosaccharides using ionic liquid matrices. *Anal. Chem.* **2006,** *78* (6), 1774-1779.

(300) Fitzgerald, J. J.; Kunnath, P.; Walker, A. V. Matrix-enhanced secondary ion mass spectrometry (ME SIMS) using room temperature ionic liquid matrices. *Anal. Chem.* **2010,** *82* (11), 4413-4419.

(301) Crank, J. A.; Armstrong, D. W. Towards a second generation of ionic liquid matrices (ILMs) for MALDI-MS of peptides, proteins, and carbohydrates. *J. Am. Soc. Mass Spectrom.* **2009,** *20* (10), 1790-1800.

(302) Berthod, A.; Crank, J. A.; Rundlett, K. L.; Armstrong, D. W. A second-generation ionic liquid matrix-assisted laser desorption/ionization matrix for effective mass spectrometric analysis of biodegradable polymers. *Rapid Commun. Mass Spectrom.* **2009,** *23* (21), 3409-3422.

(303) Calvano, C. D.; Ceglie, C. D.; D'Accolti, L.; Zambonin, C. G. MALDI-TOF mass spectrometry detection of extra-virgin olive oil adulteration with hazelnut oil by analysis of phospholipids using an ionic liquid as matrix and extraction solvent. *Food Chem.* **2012,** *134* (2), 1192-1198.

(304) Shrivas, K.; Hayasaka, T.; Goto-Inoue, N.; Sugiura, Y.; Zaima, N.; Setou, M. Ionic matrix for enhanced MALDI imaging mass spectrometry for identification of phospholipids in mouse liver and cerebellum tissue sections. *Anal. Chem.* **2010,** *82* (21), 8800-8806.

(305) Naumann, I.; Darsow, K. H.; Walter, C.; Lange, H. A.; Buchholz, R. Identification of sulfoglycolipids from the alga Porphyridium purpureum by matrix-assisted laser desorption/ionisation quadrupole ion trap time-of-flight mass spectrometry. *Rapid Commun. Mass Spectrom.* **2007,** *21* (19), 3185-3192.

(306) Palmblad, M.; Cramer, R. Liquid matrix deposition on conductive hydrophobic surfaces for tuning and quantitation in UV-MALDI mass spectrometry. *J. Am. Soc. Mass Spectrom.* **2007,** *18* (4), 693-697.

(307) Yamazaki, Y.; Nakaya, S.; Ito, K.; Kato, K. Analysis of High-Molecular-Weight Polyrotaxanes by MALDI-TOF-MS Using 3-Aminoquinoline-Based Ionic Liquid Matrix. *J. Am. Soc. Mass Spectrom.* **2020,** *31* (6), 1180-1188.

(308) Towers, M. W.; McKendrick, J. E.; Cramer, R. Introduction of 4-chloro-alpha-cyanocinnamic acid liquid matrices for high sensitivity UV-MALDI MS. *J. Proteome Res.* **2010,** *9* (4), 1931-1940.

(309) Mukherjee, G.; Claudia Rower, C.; Koy, C.; Protzel, C.; Lorenz, P.; Thiesen, H. J.; Hakenberg, O. W.; Glocker, M. O. Ultraviolet matrix-assisted laser desorption/ionization time-of-flight mass spectrometry for phosphopeptide analysis with a solidified ionic liquid matrix. *Eur. J. Mass Spectrom. (Chichester)* **2015,** *21* (2), 65-77.

(310) Laremore, T. N.; Zhang, F.; Linhardt, R. J. Ionic liquid matrix for direct UV-MALDI-TOF-MS analysis of dermatan sulfate and chondroitin sulfate oligosaccharides. *Anal. Chem.* **2007,** *79* (4), 1604-1610.

(311) Fukuyama, Y.; Nakaya, S.; Yamazaki, Y.; Tanaka, K. Ionic liquid matrixes optimized for MALDI-MS of sulfated/sialylated/neutral oligosaccharides and glycopeptides. *Anal. Chem.* **2008,** *80* (6), 2171-2179.

(312) Schmidt De Leon, T.; Salum, M. L.; Erra-Balsells, R. norHarmane containing ionic liquid matrices for low molecular weight MALDI-MS carbohydrate analysis: The perfect couple with alpha-cyano-4-hydroxycinnamic acid. *J. Mass Spectrom.* **2019,** *54* (7), 643-654.

(313) Ramos Catharino, R.; de Azevedo Marques, L.; Silva Santos, L.; Baptista, A. S.; Gloria, E. M.; Calori-Domingues, M. A.; Facco, E. M.; Eberlin, M. N. Aflatoxin screening by MALDI-TOF mass spectrometry. *Anal. Chem.* **2005,** *77* (24), 8155-8157.

(314) Przybylski, C.; Gonnet, F.; Bonnaffe, D.; Hersant, Y.; Lortat-Jacob, H.; Daniel, R. HABA-based ionic liquid matrices for UV-MALDI-MS analysis of heparin and heparan sulfate oligosaccharides. *Glycobiology* **2010,** *20* (2), 224-234.

(315) Ullmer, R.; Rizzi, A. M. Use of a novel ionic liquid matrix for MALDI-MS analysis of glycopeptides and glycans out of total tryptic digests. *J. Mass Spectrom.* **2009,** *44* (11), 1596-1603.

(316) Serrano, C. A.; Zhang, Y.; Yang, J.; Schug, K. A. Matrix-assisted laser desorption/ionization mass spectrometric analysis of aliphatic biodegradable photoluminescent polymers using new ionic liquid matrices. *Rapid Commun. Mass Spectrom.* **2011,** *25* (9), 1152-1158.

(317) Ham, B. M.; Jacob, J. T.; Cole, R. B. MALDI-TOF MS of phosphorylated lipids in biological fluids using immobilized metal affinity chromatography and a solid ionic crystal matrix. *Anal. Chem.* **2005,** *77* (14), 4439-4447.

(318) Wang, H.; Wu, Y.; Guo, B.; Sun, W.; Ding, L.; Chen, B. Quantification of low-polar small molecules using room temperature ionic liquids matrix-assisted desorption corona beam ionization. *Analyst* **2012,** *137* (17), 3982-3988.

(319) Gabriel, S. J.; Pfeifer, D.; Schwarzinger, C.; Panne, U.; Weidner, S. M. Matrix-assisted laser desorption/ionization time-of-flight mass spectrometric imaging of synthetic polymer sample spots prepared using ionic liquid matrices. *Rapid Commun. Mass Spectrom.* **2014,** *28* (5), 489-498.

(320) Abdelhamid, H. N.; Khan, M. S.; Wu, H. F. Design, characterization and applications of new ionic liquid matrices for multifunctional analysis of biomolecules: a novel strategy for pathogenic bacteria biosensing. *Anal. Chim. Acta* **2014,** *823*, 51-60.

(321) Carda-Broch, S.; Berthod, A.; Armstrong, D. W. Ionic matrices for matrix-assisted laser desorption/ionization time-of-flight detection of DNA oligomers. *Rapid Commun. Mass Spectrom.* **2003,** *17* (6), 553-560.

(322) Kosyakov, D. S.; Anikeenko, E. A.; Ul'yanovskii, N. V.; Khoroshev, O. Y.; Shavrina, I. S.; Gorbova, N. S. Ionic liquid matrices for MALDI mass spectrometry of lignin. *Anal. Bioanal. Chem.* **2018,** *410* (28), 7429-7439.

(323) Mechref, Y.; Novotny, M. V. Matrix-assisted laser desorption/ionization mass spectrometry of acidic glycoconjugates facilitated by the use of spermine as a co-matrix. *J. Am. Soc. Mass Spectrom.* **1998,** *9* (12), 1293-1302.

(324) Soltwisch, J.; Berkenkamp, S.; Dreisewerd, K. A binary matrix of 2,5-dihydroxybenzoic acid and glycerol produces homogenous sample preparations for matrix-assisted laser desorption/ionization mass spectrometry. *Rapid Commun. Mass Spectrom.* **2008,** *22* (1), 59-66.

(325) Kim, Y.; Kim, T.; Lee, J.; Im, H.; Kim, J. Enhanced Detection of Glycans by MALDI-TOF Mass Spectrometry Using a Binary Matrix of 2,5-Dihydroxybenzoic Acid and 2,6-Dihydroxybenzoic Acid. *Mass Spectrom. Lett.* **2013,** *4* (2), 38-40.

(326) Zhao, X.; Guo, C.; Huang, Y.; Huang, L.; Ma, G.; Liu, Y.; He, Q.; Wang, H.; Chen, K.; Pan, Y. Combination Strategy of Reactive and Catalytic Matrices for Qualitative and Quantitative Profiling of N-Glycans in MALDI-MS. *Anal. Chem.* **2019,** *91* (14), 9251-9258.

(327) Lavanant, H.; Loutelier-Bourhis, C. Use of procaine and procainamide as derivatizing co-matrices for the analysis of oligosaccharides by matrix-assisted laser desorption/ionization time-of-flight mass spectrometry. *Rapid Commun. Mass Spectrom.* **2012,** *26* (11), 1311-1319.

(328) Urakami, S.; Hinou, H. MALDI O-antigen glycotyping of Y. pseudotuberculosis using DAN/DHB/K matrix. *BBA Adv.* **2025,** *7*, 100131.
(329) Yamaguchi, M.; Fukuyama, Y.; Izumi, S. Alkylated Hydroxychalcone: A Novel Matrix for Peptide Analysis by Matrix-Assisted Laser Desorption Ionization Mass Spectrometry. *Mass Spectrom. (Tokyo)* **2025,** *14* (1), A0170.
(330) Hsieh, C. H.; Tam, M. F. Detection of dimethylarginines in protein hydrolysates by matrix-assisted laser desorption/ionization mass spectrometry. *Anal. Biochem.* **2006,** *350* (1), 151-155.
(331) Guo, Z.; He, L. A binary matrix for background suppression in MALDI-MS of small molecules. *Anal. Bioanal. Chem.* **2007,** *387* (5), 1939-1944.
(332) Zhou, L. H.; Kang, G. Y.; Kim, K. P. A binary matrix for improved detection of phosphopeptides in matrix-assisted laser desorption/ionization mass spectrometry. *Rapid Commun. Mass Spectrom.* **2009,** *23* (15), 2264-2272.
(333) Qiu, X.; Chen, F.; Liu, T.; Feng, F.; Zhang, Y.; Feng, X.; Zhang, F. Developing CHCA/PPD as a novel matrix for enhanced matrix-assisted laser desorption/ionization-mass spectrometry imaging for analysis of antibiotics in grass carp tissues. *Rapid Commun. Mass Spectrom.* **2023,** *37* (5), e9428.
(334) Laugesen, S.; Roepstorff, P. Combination of two matrices results in improved performance of MALDI MS for peptide mass mapping and protein analysis. *J. Am. Soc. Mass Spectrom.* **2003,** *14* (9), 992-1002.
(335) Shanta, S. R.; Zhou, L. H.; Park, Y. S.; Kim, Y. H.; Kim, Y.; Kim, K. P. Binary matrix for MALDI imaging mass spectrometry of phospholipids in both ion modes. *Anal. Chem.* **2011,** *83* (4), 1252-1259.
(336) Liepold, T.; Klafki, H. W.; Kumar, S.; Walter, J.; Wirths, O.; Wiltfang, J.; Jahn, O. Matrix Development for the Detection of Phosphorylated Amyloid-beta Peptides by MALDI-TOF-MS. *J. Am. Soc. Mass Spectrom.* **2023,** *34* (3), 505-512.
(337) Zhu, Y. F.; Chung, C. N.; Taranenko, N. I.; Allman, S. L.; Martin, S. A.; Haff, L.; Chen, C. H. The study of 2,3,4-trihydroxyacetophenone and 2,4,6-trihydroxyacetophenone as matrices for DNA detection in matrix-assisted laser desorption/ionization time-of-flight mass spectrometry. *Rapid Commun. Mass Spectrom.* **1996,** *10* (3), 383-388.
(338) Zhou, L.; Deng, H.; Deng, Q.; Zhao, S. A mixed matrix of 3-hydroxypicolinic acid and pyrazinecarboxylic acid for matrix-assisted laser desorption/ionization time-of-flight mass spectrometry of oligodeoxynucleotides. *Rapid Commun. Mass Spectrom.* **2004,** *18* (7), 787-794.
(339) Shanta, S. R.; Kim, T. Y.; Hong, J. H.; Lee, J. H.; Shin, C. Y.; Kim, K. H.; Kim, Y. H.; Kim, S. K.; Kim, K. P. A new combination MALDI matrix for small molecule analysis: application to imaging mass spectrometry for drugs and metabolites. *Analyst* **2012,** *137* (24), 5757-5762.
(340) Calvano, C. D.; Monopoli, A.; Ditaranto, N.; Palmisano, F. 1,8-bis(dimethylamino)naphthalene/9-aminoacridine: a new binary matrix for lipid fingerprinting of intact bacteria by matrix assisted laser desorption ionization mass spectrometry. *Anal. Chim. Acta* **2013,** *798*, 56-63.
(341) Wang, J.; Wang, C.; Han, X. Enhanced coverage of lipid analysis and imaging by matrix-assisted laser desorption/ionization mass spectrometry via a strategy with an optimized mixture of matrices. *Anal. Chim. Acta* **2018,** *1000*, 155-162.
(342) Yang, C.; Hu, X.; Loboda, A. V.; Lipson, R. H. A useful binary matrix for visible-MALDI of low molecular weight analytes. *J. Am. Soc. Mass Spectrom.* **2010,** *21* (2), 294-299.
(343) Holbrook, J. H.; Sekera, E. R.; Lopez, A.; Fries, B. D.; Tobias, F.; Akkaya, K.; Mihaylova, M. M.; Hummon, A. B. Enhancement of Lipid Signals in Matrix-Assisted Laser Desorption/Ionization Mass Spectrometry with Ammonium Fluoride as a Matrix Additive. *Anal. Chem.* **2023,** *95* (28), 10603-10609.
(344) Yamagaki, T.; Suzuki, H.; Tachibana, K. Solid-phase fluorescence and ionization efficiency in negative-

ion matrix-assisted laser desorption/ionization of neutral oligosaccharides: interaction between beta-carboline matrix and ammonium salt. *J. Am. Soc. Mass Spectrom.* **2007,** *18* (4), 714-723.

(345) Kang, J. H.; Toita, R.; Oishi, J.; Niidome, T.; Katayama, Y. Effect of the addition of diammonium citrate to alpha-cyano-4-hydroxycinnamic acid (CHCA) matrix for the detection of phosphorylated peptide in phosphorylation reactions using cell and tissue lysates. *J. Am. Soc. Mass Spectrom.* **2007,** *18* (11), 1925-1931.

(346) Vandell, V. E.; Limbach, P. A. Polyamine co-matrices for matrix-assisted laser desorption/ionization mass spectrometry of oligonucleotides. *Rapid Commun. Mass Spectrom.* **1999,** *13* (20), 2014-2021.

(347) Asara, J. M.; Allison, J. Enhanced detection of oligonucleotides in UV MALDI MS using the tetraamine spermine as a matrix additive. *Anal. Chem.* **1999,** *71* (14), 2866-2870.

(348) Bashir, S.; Derrick, P. J.; Mutter, R. Parameterising matrix-assisted laser desorption/ionization (MALDI): effect of solvents and co-additives on analyte peak intensities. *Eur. J. Mass Spectrom. (Chichester)* **2004,** *10* (4), 487-493.

(349) Distler, A. M.; Allison, J. Improved MALDI-MS analysis of oligonucleotides through the use of fucose as a matrix additive. *Anal. Chem.* **2001,** *73* (20), 5000-5003.

(350) Billeci, T. M.; Stults, J. T. Tryptic mapping of recombinant proteins by matrix-assisted laser desorption/ionization mass spectrometry. *Anal. Chem.* **1993,** *65* (13), 1709-1716.

(351) Loo, R. R.; Loo, J. A. Matrix-assisted laser desorption/ionization-mass spectrometry of hydrophobic proteins in mixtures using formic acid, perfluorooctanoic acid, and sorbitol. *Anal. Chem.* **2007,** *79* (3), 1115-1125.

(352) Yamaguchi, S.; Fujita, T.; Fujino, T.; Korenaga, T. Suppression of matrix-related ions using cyclodextrin in MALDI mass spectrometry. *Anal. Sci.* **2008,** *24* (11), 1497-1500.

(353) Zhou, P.; Altman, E.; Perry, M. B.; Li, J. Study of matrix additives for sensitive analysis of lipid A by matrix-assisted laser desorption ionization mass spectrometry. *Appl. Environ. Microbiol.* **2010,** *76* (11), 3437-3443.

(354) Jabbar Siddiqui, A.; Le Senechal, C.; Vilain, S.; Bure, C. Effect of matrices and additives on phosphorylated and ketodeoxyoctonic acid lipids A analysis by matrix-assisted laser desorption ionization-mass spectrometry. *J. Mass Spectrom.* **2020,** *55* (10), e4600.

(355) Andrade, L. M.; Mendes, M. A.; Kowalski, P.; Nascimento, C. A. Comparative study of different matrix/solvent systems for the analysis of crude lyophilized microalgal preparations using matrix-assisted laser desorption/ionization time-of-flight mass spectrometry. *Rapid Commun. Mass Spectrom.* **2015,** *29* (3), 295-303.

(356) Kjellstrom, S.; Jensen, O. N. Phosphoric acid as a matrix additive for MALDI MS analysis of phosphopeptides and phosphoproteins. *Anal. Chem.* **2004,** *76* (17), 5109-5117.

(357) Park, S.; Kim, T.; Lee, J.; Seo, M.; Kim, J. Effect of phosphoric acid as a matrix additive in matrix-assisted laser desorption/ionization analysis. *Rapid Commun. Mass Spectrom.* **2013,** *27* (7), 842-846.

(358) Kuyama, H.; Sonomura, K.; Nishimura, O. Sensitive detection of phosphopeptides by matrix-assisted laser desorption/ionization mass spectrometry: use of alkylphosphonic acids as matrix additives. *Rapid Commun. Mass Spectrom.* **2008,** *22* (8), 1109-1116.

(359) Kim, J. S.; Kim, J. Y.; Kim, H. J. Suppression of matrix clusters and enhancement of peptide signals in MALDI-TOF mass spectrometry using nitrilotriacetic acid. *Anal. Chem.* **2005,** *77* (22), 7483-7488.

(360) Fukuyama, Y.; Tanimura, R.; Maeda, K.; Watanabe, M.; Kawabata, S.; Iwamoto, S.; Izumi, S.; Tanaka, K. Alkylated dihydroxybenzoic acid as a MALDI matrix additive for hydrophobic peptide analysis. *Anal. Chem.* **2012,** *84* (9), 4237-4243.

(361) Apicella, B.; Ciajolo, A.; Millan, M.; Galmes, C.; Herod, A. A.; Kandiyoti, R. Oligomeric carbon and siloxane series observed by matrix-assisted laser desorption/ionisation and laser desorption/ionisation mass spectrometry during the analysis of soot formed in fuel-rich flames. *Rapid Commun. Mass Spectrom.* **2004,** *18* (3), 331-338.

(362) Choi, S.-S.; Ha, S.-H. Influence of sample preparation method and silver salt types on MALDI-TOFMS analysis of polybutadiene. *Macromol. Res.* **2008,** *16* (2), 108-112.

(363) Deery, M. J.; Jennings, K. R.; Jasieczek, C. B.; Haddleton, D. M.; Jackson, A. T.; Yates, H. T.; Scrivens, J. H. A Study of Cation Attachment to Polystyrene by Means of Matrix-assisted Laser Desorption/Ionization and Electrospray Ionization-Mass Spectrometry. *Rapid Commun. Mass Spectrom.* **1997,** *11* (1), 57-62.

(364) Meier, F.; Garrard, K. P.; Muddiman, D. C. Silver dopants for targeted and untargeted direct analysis of unsaturated lipids via infrared matrix-assisted laser desorption electrospray ionization (IR-MALDESI). *Rapid Commun. Mass Spectrom.* **2014,** *28* (22), 2461-2470.

(365) Patil, A. A.; Lai, T. K. L.; Lee, C. C.; Chiu, Y. P.; Liu, Z. X.; Lin, C. J.; Peng, W. P. Enhancing mass analysis of ultra-high molecular weight polystyrene: a comparative study of copper and silver salts with MALDI mass spectrometry. *Anal. Methods* **2025,** *17* (8), 1754-1764.

(366) Muyizere, T.; Mukiza, J. Progress on the development of a metal salt-assisted ionization source for the mass spectrometric analysis of polymers. *Anal. Methods* **2022,** *14* (29), 2803-2819.

(367) Keki, S.; Deak, G.; Zsuga, M. Copper(I) chloride: a simple salt for enhancement of polystyrene cationization in matrix-assisted laser desorption/ionization mass spectrometry. *Rapid Commun. Mass Spectrom.* **2001,** *15* (9), 675-678.

(368) Schiller, J.; Suss, R.; Petkovic, M.; Hilbert, N.; Muller, M.; Zschornig, O.; Arnhold, J.; Arnold, K. CsCl as an auxiliary reagent for the analysis of phosphatidylcholine mixtures by matrix-assisted laser desorption and ionization time-of-flight mass spectrometry (MALDI-TOF MS). *Chem. Phys. Lipids* **2001,** *113* (1-2), 123-131.

(369) Cerruti, C. D.; Touboul, D.; Guerineau, V.; Petit, V. W.; Laprevote, O.; Brunelle, A. MALDI imaging mass spectrometry of lipids by adding lithium salts to the matrix solution. *Anal. Bioanal. Chem.* **2011,** *401* (1), 75-87.

(370) Domann, P.; Spencer, D. I.; Harvey, D. J. Production and fragmentation of negative ions from neutral N-linked carbohydrates ionized by matrix-assisted laser desorption/ionization. *Rapid Commun. Mass Spectrom.* **2012,** *26* (4), 469-479.

(371) Griffiths, R. L.; Bunch, J. A survey of useful salt additives in matrix-assisted laser desorption/ionization mass spectrometry and tandem mass spectrometry of lipids: introducing nitrates for improved analysis. *Rapid Commun. Mass Spectrom.* **2012,** *26* (13), 1557-1566.

(372) Wong, A. W.; Wang, H.; Lebrilla, C. B. Selection of anionic dopant for quantifying desialylation reactions with MALDI-FTMS. *Anal. Chem.* **2000,** *72* (7), 1419-1425.

(373) Kobayashi, T.; Kawai, H.; Suzuki, T.; Kawanishi, T.; Hayakawa, T. Improved sensitivity for insulin in matrix-assisted laser desorption/ionization time-of-flight mass spectrometry by premixing alpha-cyano-4-hydroxycinnamic acid matrix with transferrin. *Rapid Commun. Mass Spectrom.* **2004,** *18* (10), 1156-1160.

(374) Ding, J.; Liu, S.; Xiao, H. M.; Ye, T. T.; Zhou, P.; Feng, Y. Q. Matrix-assisted laser desorption/ionization mass spectrometry for the analysis of polyamines in plant micro-tissues using cucurbituril as a host molecule. *Anal. Chim. Acta* **2017,** *987*, 56-63.

(375) Mandal, G.; Moran, L.; Pecinka, L.; Vanhara, P.; Havel, J. Matrix enrichment by black phosphorus

improves ionization and reproducibility of mass spectrometry of intact cells, peptides, and amino acids. *Sci. Rep.* **2022,** *12* (1), 1175.

(376) Wu, H. P.; Yu, C. J.; Lin, C. Y.; Lin, Y. H.; Tseng, W. L. Gold nanoparticles as assisted matrices for the detection of biomolecules in a high-salt solution through laser desorption/ionization mass spectrometry. *J. Am. Soc. Mass Spectrom.* **2009,** *20* (5), 875-882.

(377) Son, J.; Lee, G.; Cha, S. Direct analysis of triacylglycerols from crude lipid mixtures by gold nanoparticle-assisted laser desorption/ionization mass spectrometry. *J. Am. Soc. Mass Spectrom.* **2014,** *25* (5), 891-894.

(378) McLaughlin, N.; Bielinski, T. M.; Tressler, C. M.; Barton, E.; Glunde, K.; Stumpo, K. A. Pneumatically Sprayed Gold Nanoparticles for Mass Spectrometry Imaging of Neurotransmitters. *J. Am. Soc. Mass Spectrom.* **2020,** *31* (12), 2452-2461.

(379) Hsieh, Y. T.; Chen, W. T.; Chang, H. T. Detection of Nucleoside Monophosphates through Surface-Assisted Laser, Desorption/Ionization Mass Spectrometry Using CTAB-Adsorbed Gold, Nanoparticles. *J. Chin. Chem. Soc.* **2011,** *58* (6), 761-768.

(380) Huang, Y. F.; Chang, H. T. Nile Red-adsorbed gold nanoparticle matrixes for determining aminothiols through surface-assisted laser desorption/ionization mass spectrometry. *Anal. Chem.* **2006,** *78* (5), 1485-1493.

(381) Huang, Y. F.; Chang, H. T. Analysis of adenosine triphosphate and glutathione through gold nanoparticles assisted laser desorption/ionization mass spectrometry. *Anal. Chem.* **2007,** *79* (13), 4852-4859.

(382) Kailasa, S. K.; Wu, H. F. One-pot synthesis of dopamine dithiocarbamate functionalized gold nanoparticles for quantitative analysis of small molecules and phosphopeptides in SALDI- and MALDI-MS. *Analyst* **2012,** *137* (7), 1629-1638.

(383) Goto-Inoue, N.; Hayasaka, T.; Zaima, N.; Kashiwagi, Y.; Yamamoto, M.; Nakamoto, M.; Setou, M. The detection of glycosphingolipids in brain tissue sections by imaging mass spectrometry using gold nanoparticles. *J. Am. Soc. Mass Spectrom.* **2010,** *21* (11), 1940-1943.

(384) Liu, R.; Liu, J. F.; Zhou, X. X.; Jiang, G. B. Cysteine modified small ligament Au nanoporous film: an easy fabricating and highly efficient surface-assisted laser desorption/ionization substrate. *Anal. Chem.* **2011,** *83* (10), 3668-3674.

(385) Zhao, Y.; Boukherroub, R.; Xu, G.; Li, H.; Zhao, R. S.; Wei, Q.; Yu, X.; Chen, X. Au@BN-enhanced laser desorption/ionization mass spectrometry and imaging for determination of fipronil and its metabolites in food and biological samples. *Food Chem.* **2023,** *418*, 135935.

(386) Shan, L.; Qiao, Y.; Ma, L.; Zhang, X.; Chen, C.; Xu, X.; Li, D.; Qiu, S.; Xue, X.; Yu, Y.et al. AuNPs/CNC Nanocomposite with A "Dual Dispersion" Effect for LDI-TOF MS Analysis of Intact Proteins in NSCLC Serum Exosomes. *Adv. Sci. (Weinh.)* **2024,** *11* (12), e2307360.

(387) Sekula, J.; Niziol, J.; Rode, W.; Ruman, T. Gold nanoparticle-enhanced target (AuNPET) as universal solution for laser desorption/ionization mass spectrometry analysis and imaging of low molecular weight compounds. *Anal. Chim. Acta* **2015,** *875*, 61-72.

(388) Niziol, J.; Ossolinski, K.; Ossolinski, T.; Ossolinska, A.; Bonifay, V.; Sekula, J.; Dobrowolski, Z.; Sunner, J.; Beech, I.; Ruman, T. Surface-Transfer Mass Spectrometry Imaging of Renal Tissue on Gold Nanoparticle Enhanced Target. *Anal. Chem.* **2016,** *88* (14), 7365-7371.

(389) Krupa, S.; Niziol, J. Fiber Laser-Generated Silver-109 Nanoparticles for Laser Desorption/Ionization Mass Spectrometry of Illicit Drugs. *J. Am. Soc. Mass Spectrom.* **2024,** *35* (6), 1156-1167.

(390) Hua, L.; Chen, J.; Ge, L.; Tan, S. N. Silver nanoparticles as matrix for laser desorption/ionization mass spectrometry of peptides. *J. Nanopart. Res.* **2007,** *9*, 1133-1138.

(391) Kailasa, S. K.; Wu, H. F. Surface modified silver selinide nanoparticles as extracting probes to improve peptide/protein detection via nanoparticles-based liquid phase microextraction coupled with MALDI mass spectrometry. *Talanta* **2010,** *83* (2), 527-534.

(392) Guan, M.; Zhang, Z.; Li, S.; Liu, J.; Liu, L.; Yang, H.; Zhang, Y.; Wang, T.; Zhao, Z. Silver nanoparticles as matrix for MALDI FTICR MS profiling and imaging of diverse lipids in brain. *Talanta* **2018,** *179*, 624-631.

(393) Gamez, R. C.; Castellana, E. T.; Russell, D. H. Sol-gel-derived silver-nanoparticle-embedded thin film for mass spectrometry-based biosensing. *Langmuir* **2013,** *29* (21), 6502-6507.

(394) Schnapp, A.; Niehoff, A. C.; Koch, A.; Dreisewerd, K. Laser desorption/ionization mass spectrometry of lipids using etched silver substrates. *Methods* **2016,** *104*, 194-203.

(395) Niziol, J.; Ruman, T. Surface-transfer mass spectrometry imaging on a monoisotopic silver nanoparticle enhanced target. *Anal. Chem.* **2013,** *85* (24), 12070-12076.

(396) Arendowski, A.; Niziol, J.; Ruman, T. Silver-109-based laser desorption/ionization mass spectrometry method for detection and quantification of amino acids. *J. Mass Spectrom.* **2018,** *53* (4), 369-378.

(397) Niziol, J.; Sunner, J.; Beech, I.; Ossolinski, K.; Ossolinska, A.; Ossolinski, T.; Plaza, A.; Ruman, T. Localization of Metabolites of Human Kidney Tissue with Infrared Laser-Based Selected Reaction Monitoring Mass Spectrometry Imaging and Silver-109 Nanoparticle-Based Surface Assisted Laser Desorption/Ionization Mass Spectrometry Imaging. *Anal. Chem.* **2020,** *92* (6), 4251-4258.

(398) Maslak, E.; Arendowski, A.; Zloch, M.; Walczak-Skierska, J.; Radtke, A.; Piszczek, P.; Pomastowski, P. Silver Nanoparticle Targets Fabricated Using Chemical Vapor Deposition Method for Differentiation of Bacteria Based on Lipidomic Profiles in Laser Desorption/Ionization Mass Spectrometry. *Antibiotics (Basel)* **2023,** *12* (5), 874.

(399) Sibinska, E.; Walczak-Skierska, J.; Arendowski, A.; Ludwiczak, A.; Radtke, A.; Piszczek, P.; Gabrys, D.; Robotnik, K.; Pomastowski, P. Advances in LDI-MS Analysis: The Role of Chemical Vapor Deposition-Synthesized Silver Nanoparticles in Enhancing Detection of Low-Molecular-Weight Biomolecules. *J. Am. Soc. Mass Spectrom.* **2024,** *35* (9), 2041-2055.

(400) Shrivas, K.; Agrawal, K.; Wu, H. F. Application of platinum nanoparticles as affinity probe and matrix for direct analysis of small biomolecules and microwave digested proteins using matrix-assisted laser desorption/ionization mass spectrometry. *Analyst* **2011,** *136* (13), 2852-2857.

(401) Nitta, S.; Kawasaki, H.; Suganuma, T.; Shigeri, Y.; Arakawa, R. Desorption/ionization efficiency of common amino acids in surface-assisted laser desorption/ionization mass spectrometry (SALDI-MS) with nanostructured platinum. *J. Phys. Chem. C* **2013,** *117* (1), 238-245.

(402) Shen, Y. L.; Zhuang, S. J.; Yang, F.; Gong, C.; Xu, X. Prefabricated platinum nanomaterial matrix for MALDI-MS imaging of oligosaccharides and lipids in plant tissues. *Front. Plant Sci.* **2023,** *14*, 1105374.

(403) Silina, Y. E.; Meier, F.; Nebolsin, V. A.; Koch, M.; Volmer, D. A. Novel galvanic nanostructures of Ag and Pd for efficient laser desorption/ionization of low molecular weight compounds. *J. Am. Soc. Mass Spectrom.* **2014,** *25* (5), 841-851.

(404) Liu, Y.; Wang, Y.; Wan, X.; Huang, H.; Shen, J.; Wu, B.; Zhu, L.; Wu, B.; Liu, W.; Huang, L.et al. Ferric particle-assisted LDI-MS platform for metabolic fingerprinting of diabetic retinopathy. *Clin. Chem. Lab. Med.* **2024,** *62* (5), 988-998.

(405) Wan, Q.; Zhang, Z.; Zhao, M.; Ruan, X.; Hao, Y.; Deng, J.; She, Y.; Yang, M.; Song, Y.; Jin, F.et al. Efficient diagnosis of benign and malignant pulmonary nodules based on Nano-zero-valent iron enhanced serum metabolic fingerprinting. *Chin. Chem. Lett.* **2025,** *36* (10), 110794.

(406) Watanabe, T.; Okumura, K.; Kawasaki, H.; Arakawa, R. Effect of urea surface modification and

photocatalytic cleaning on surface-assisted laser desorption ionization mass spectrometry with amorphous TiO2 nanoparticles. *J. Mass Spectrom.* **2009,** *44* (10), 1443-1451.

(407) Yang, J.; Deng, Q.; Luo, C.; Yang, A.; Luo, X.; Guo, M.; Fu, L.; Zhen, D.; Zhou, H. Holmium modified TiO2 nanocomposites as a matrix-assisted laser desorption/ionization time-of-flight mass spectrometry matrix for the detection of bisphenol S and indigo. *Mater. Express* **2023,** *13* (11), 1922-1928.

(408) Wu, Q.; Chu, J. L.; Rubakhin, S. S.; Gillette, M. U.; Sweedler, J. V. Dopamine-modified TiO(2) monolith-assisted LDI MS imaging for simultaneous localization of small metabolites and lipids in mouse brain tissue with enhanced detection selectivity and sensitivity. *Chem. Sci.* **2017,** *8* (5), 3926-3938.

(409) Han, X.; Yang, Y.; Lu, J.; Lin, Y.; Zhang, D.; Lin, L.; Qiao, L. Efficient serum lipids profiling by TiO2-dopamin-assisted MALDI-TOF MS for breast cancer detection. *Chin. Chem. Lett.* **2025,** *36* (5), 110183.

(410) Chen, C. T.; Chen, Y. C. Molecularly imprinted TiO2-matrix-assisted laser desorption/ionization mass spectrometry for selectively detecting alpha-cyclodextrin. *Anal. Chem.* **2004,** *76* (5), 1453-1457.

(411) Wang, H.; Duan, J.; Cheng, Q. Photocatalytically patterned TiO2 arrays for on-plate selective enrichment of phosphopeptides and direct MALDI MS analysis. *Anal. Chem.* **2011,** *83* (5), 1624-1631.

(412) Torta, F.; Fusi, M.; Casari, C. S.; Bottani, C. E.; Bachi, A. Titanium dioxide coated MALDI plate for on target analysis of phosphopeptides. *J. Proteome Res.* **2009,** *8* (4), 1932-1942.

(413) Niklew, M. L.; Hochkirch, U.; Melikyan, A.; Moritz, T.; Kurzawski, S.; Schluter, H.; Ebner, I.; Linscheid, M. W. Phosphopeptide screening using nanocrystalline titanium dioxide films as affinity matrix-assisted laser desorption ionization targets in mass spectrometry. *Anal. Chem.* **2010,** *82* (3), 1047-1053.

(414) Torta, F.; Fusi, M.; Casari, C. S.; Bassi, A. L.; Bachi, A. Nanostructured TiO(2) thin films for phosphoproteomics studies with MALDI mass spectrometry. *Methods Mol. Biol.* **2011,** *790*, 173-181.

(415) Piret, G.; Kim, D.; Drobecq, H.; Coffinier, Y.; Melnyk, O.; Schmuki, P.; Boukherroub, R. Surface-assisted laser desorption-ionization mass spectrometry on titanium dioxide (TiO2) nanotube layers. *Analyst* **2012,** *137* (13), 3058-3063.

(416) Dutkiewicz, E. P.; Su, C. H.; Lee, H. J.; Hsu, C. C.; Yang, Y. L. Visualizing vinca alkaloids in the petal of Catharanthus roseus using functionalized titanium oxide nanowire substrate for surface-assisted laser desorption/ionization imaging mass spectrometry. *Plant J.* **2021,** *105* (4), 1123-1133.

(417) Watanabe, T.; Kawasaki, H.; Yonezawa, T.; Arakawa, R. Surface-assisted laser desorption/ionization mass spectrometry (SALDI-MS) of low molecular weight organic compounds and synthetic polymers using zinc oxide (ZnO) nanoparticles. *J. Mass Spectrom.* **2008,** *43* (8), 1063-1071.

(418) Gedda, G.; Wu, H.-F. Fabrication of surface modified ZnO nanorod array for MALDI-MS analysis of bacteria in a nanoliter droplet: a multiple function biochip. *Sensors Actuators B: Chem.* **2019,** *288*, 667-677.

(419) Chen, W. Y.; Chen, Y. C. Affinity-based mass spectrometry using magnetic iron oxide particles as the matrix and concentrating probes for SALDI MS analysis of peptides and proteins. *Anal. Bioanal. Chem.* **2006,** *386* (3), 699-704.

(420) Kuwata, K.; Itou, K.; Kotani, M.; Ohmura, T.; Naito, Y. DIUTHAME enables matrix-free mass spectrometry imaging of frozen tissue sections. *Rapid Commun. Mass Spectrom.* **2020,** *34* (9), e8729.

(421) Qiu, Z.; Zheng, Z.; Song, Z.; Sun, Y.; Shan, Q.; Lin, Z.; Xie, Z. Co(3)O(4) nanocrystals as matrices for the detection of amino acids, harmful additives and pesticide residues by MALDI-TOF MS. *Talanta* **2022,** *242*, 123299.

(422) Qiu, Z.; Yang, K.; Huang, Z.; Zhao, H.; Lin, Z.; Kuang, Q.; Xie, Z. Application and mechanism of Co(3)O(4)/Co(OH)(2) heterojunctions as matrices for small molecules detection by MALDI-TOF MS. *J. Hazard. Mater.* **2025,** *492*, 138119.

(423) Sun, S.; Ma, H.; Han, G.; Wu, R.; Zou, H.; Liu, Y. Efficient enrichment and identification of phosphopeptides by cerium oxide using on-plate matrix-assisted laser desorption/ionization time-of-flight mass spectrometric analysis. *Rapid Commun. Mass Spectrom.* **2011,** *25* (13), 1862-1868.

(424) Yang, X.; Hu, X. K.; Loboda, A. V.; Lipson, R. H. Microstructured tungsten oxide: a generic desorption/ionization substrate for mass spectrometry. *Adv. Mater.* **2010,** *22* (40), 4520-4523.

(425) Shan, Z.; Han, L.; Yuan, M.; Deng, C.; Zhao, D.; Tu, B.; Yang, P. Mesoporous tungsten titanate as matrix for matrix-assisted laser desorption/ionization time-of-flight mass spectrometry analysis of biomolecules. *Anal. Chim. Acta* **2007,** *593* (1), 13-19.

(426) Kailasa, S. K.; Wu, H. F. Surface modified BaTiO3 nanoparticles as the matrix for phospholipids and as extracting probes for LLME of hydrophobic proteins in Escherichia coli by MALDI-MS. *Talanta* **2013,** *114*, 283-290.

(427) Ocsoy, I.; Gulbakan, B.; Shukoor, M. I.; Xiong, X.; Chen, T.; Powell, D. H.; Tan, W. Aptamer-conjugated multifunctional nanoflowers as a platform for targeting, capture, and detection in laser desorption ionization mass spectrometry. *ACS Nano* **2013,** *7* (1), 417-427.

(428) Lin, H.; Yang, C.; Yin, M.; Qiu, J.; Wang, W.; Lu, J.; Xu, D.; Li, G.; Deng, C. Mesoporous NiO@ZnO nanofiber membranes via single-nozzle electrospinning for urine metabolism analysis of smokers. *Analyst* **2022,** *147* (8), 1688-1694.

(429) Li, W.; He, Q.; Li, J.; Zhou, X.; Hu, Q.; Ma, C.; Wang, X. In Situ Self-Assembled Formation of Nitrogen-Rich Ag@Ti(3)C(2) Film for Sensitive Detection and Spatial Imaging of Pesticides with Laser Desorption/Ionization Mass Spectrometry (LDI-MS). *ACS Appl. Mater. Interfaces* **2023,** *15* (14), 18402-18413.

(430) Zhou, Y.; Li, X.; Zhao, Y.; Yang, S.; Huang, L. Plasmonic alloys for quantitative determination and reaction monitoring of biothiols. *J. Mater. Chem. B* **2023,** *11* (36), 8639-8648.

(431) Su, H.; Song, Y.; Yang, S.; Zhang, Z.; Shen, Y.; Yu, L.; Chen, S.; Gao, L.; Chen, C.; Hou, D.et al. Plasmonic Alloys Enhanced Metabolic Fingerprints for the Diagnosis of COPD and Exacerbations. *ACS Cent. Sci.* **2024,** *10* (2), 331-343.

(432) Meng, K.; Shen, Y.; Hou, D.; Hu, H.; Yang, S.; Zhang, Z.; Yiming, A.; Liang, D.; Tian, W.; He, L.et al. Porous PtCu Alloys Decode Plasma Metabolic Fingerprints for the Recognition of Severe Community-Acquired Pneumonia. *Adv Healthc Mater* **2025**, e2403733.

(433) Mandal, G.; Umar, M.; Lv, R.; Guo, R.; Ge, T.; Awais, M.; Yang, S.; Hasan, M. S. U.; Liu, J. Facile synthesis of plasmonic BP@Au nanomatrix for sensitive detection of irinotecan and its active SN-38 metabolite via laser desorption/ionization mass spectrometry. *Mikrochim. Acta* **2025,** *192* (2), 98.

(434) Sang, Q.; Li, Y.; Liu, W.; Wang, Y.; Qian, K. 2024 IEEE International Conference on Manipulation, Manufacturing and Measurement on the Nanoscale (3M-NANO), 2024; p 614-617.

(435) Hu, T.; Sang, Q.; Liang, D.; Zhang, W.; Wang, Y.; Qian, K. A tunable LDI-MS platform assisted by metal-phenolic network-coated AuNPs for sensitive and customized detection of amino acids. *Talanta* **2025,** *281*, 126928.

(436) Hopwood, F. G.; Michalak, L.; Alderdice, D. S.; Fisher, K. J.; Willett, G. D. C60-assisted laser desorption/ionization mass spectrometry in the analysis of phosphotungstic acid. *Rapid Commun. Mass Spectrom.* **1994,** *8* (11), 881-885.

(437) Michalak, L.; Fisher, K. J.; Alderdice, D. S.; Jardine, D. R.; Willett, G. D. C60-assisted laser desorption-ionization mass spectrometry. *Org. Mass Spectrom.* **1994,** *29* (9), 512-515.

(438) Montsko, G.; Vaczy, A.; Maasz, G.; Mernyak, E.; Frank, E.; Bay, C.; Kadar, Z.; Ohmacht, R.; Wolfling, J.; Mark, L. Analysis of nonderivatized steroids by matrix-assisted laser desorption/ionization time-of-

flight mass spectrometry using C70 fullerene as matrix. *Anal. Bioanal. Chem.* **2009,** *395* (3), 869-874.

(439) Shiea, J.; Huang, J. P.; Teng, C. F.; Jeng, J.; Wang, L. Y.; Chiang, L. Y. Use of a water-soluble fullerene derivative as precipitating reagent and matrix-assisted laser desorption/ionization matrix to selectively detect charged species in aqueous solutions. *Anal. Chem.* **2003,** *75* (14), 3587-3595.

(440) Qin, Z. N.; Ding, J.; Yu, Q. W.; Qi, C. B.; Wu, D. M.; Zhou, P.; Feng, Y. Q. Development of C60-based labeling reagents for the determination of low-molecular-weight compounds by matrix assisted laser desorption ionization mass spectrometry (II): Determination of thiols in human serum. *Anal. Chim. Acta* **2020,** *1105*, 112-119.

(441) Liu, X. P.; Sun, W. Q.; Liu, T. X.; Liu, B. B.; Chen, C. P. Fullerenol as a water-soluble MALDI-MS matrix for rapid analysis of small molecules and efficient quantification of saccharin sodium in foods. *J. Chromatogr. B Analyt. Technol. Biomed. Life Sci.* **2021,** *1178*, 122819.

(442) Ugarov, M. V.; Egan, T.; Khabashesku, D. V.; Schultz, J. A.; Peng, H.; Khabashesku, V. N.; Furutani, H.; Prather, K. S.; Wang, H. W.; Jackson, S. N.et al. MALDI matrices for biomolecular analysis based on functionalized carbon nanomaterials. *Anal. Chem.* **2004,** *76* (22), 6734-6742.

(443) Wang, C. H.; Li, J.; Yao, S. J.; Guo, Y. L.; Xia, X. H. High-sensitivity matrix-assisted laser desorption/ionization Fourier transform mass spectrometry analyses of small carbohydrates and amino acids using oxidized carbon nanotubes prepared by chemical vapor deposition as matrix. *Anal. Chim. Acta* **2007,** *604* (2), 158-164.

(444) Meng, J.; Shi, C.; Deng, C. Facile synthesis of water-soluble multi-wall carbon nanotubes and polyaniline composites and their application in detection of small metabolites by matrix assisted laser desorption/ionization mass spectrometry. *Chem. Commun. (Camb.)* **2011,** *47* (39), 11017-11019.

(445) Shi, C.; Deng, C.; Zhang, X.; Yang, P. Synthesis of highly water-dispersible polydopamine-modified multiwalled carbon nanotubes for matrix-assisted laser desorption/ionization mass spectrometry analysis. *ACS Appl. Mater. Interfaces* **2013,** *5* (16), 7770-7776.

(446) Chen, J.; Liu, F.; Liu, H.; Wang, T.; Hui, Y.; Chen, H.; Kong, Q. Doped-MXene assists in deciphering metabolic signature of psoriasis and unraveling dysregulated leukotriene metabolism. *Arab. J. Chem.* **2024,** *17* (6), 105774.

(447) Zhang, J.; Dong, X.; Cheng, J.; Li, J.; Wang, Y. Efficient analysis of non-polar environmental contaminants by MALDI-TOF MS with graphene as matrix. *J. Am. Soc. Mass Spectrom.* **2011,** *22* (7), 1294-1298.

(448) Abdelhamid, H. N.; Wu, H. F. A method to detect metal-drug complexes and their interactions with pathogenic bacteria via graphene nanosheet assist laser desorption/ionization mass spectrometry and biosensors. *Anal. Chim. Acta* **2012,** *751*, 94-104.

(449) Huang, X.; Liu, Q.; Huang, X.; Nie, Z.; Ruan, T.; Du, Y.; Jiang, G. Fluorographene as a Mass Spectrometry Probe for High-Throughput Identification and Screening of Emerging Chemical Contaminants in Complex Samples. *Anal. Chem.* **2017,** *89* (2), 1307-1314.

(450) Min, Q.; Zhang, X.; Chen, X.; Li, S.; Zhu, J. J. N-doped graphene: an alternative carbon-based matrix for highly efficient detection of small molecules by negative ion MALDI-TOF MS. *Anal. Chem.* **2014,** *86* (18), 9122-9130.

(451) Zhao, H.; Li, Y.; Wang, J.; Cheng, M.; Zhao, Z.; Zhang, H.; Wang, C.; Wang, J.; Qiao, Y.; Wang, J. Dual-Ion-Mode MALDI MS Detection of Small Molecules with the O-P,N-Doped Carbon/Graphene Matrix. *ACS Appl. Mater. Interfaces* **2018,** *10* (43), 37732-37742.

(452) Lu, L.; Zheng, G.; Wang, M.; Wang, D.; Xia, Z. Microwave-prepared mesoporous graphene as adsorbent and matrix of surface-assisted laser desorption/ionization mass spectrometry for the enrichment and rapid

detection of polyphenols in biological samples. *Talanta* **2021,** *222*, 121365.
(453) Liu, Q.; Cheng, M.; Jiang, G. Mildly oxidized graphene: facile synthesis, characterization, and application as a matrix in MALDI mass spectrometry. *Chemistry* **2013,** *19* (18), 5561-5565.
(454) Zhang, J.; Zheng, X.; Ni, Y. Selective Enrichment and MALDI-TOF MS Analysis of Small Molecule Compounds with Vicinal Diols by Boric Acid-Functionalized Graphene Oxide. *J. Am. Soc. Mass Spectrom.* **2015,** *26* (8), 1291-1298.
(455) Liang, K.; Gao, H.; Gu, Y.; Yang, S.; Zhang, J.; Li, J.; Wang, Y.; Wang, Y.; Li, Y. Graphene oxide aggregate-assisted LDI-MS for the direct analysis of triacylglycerol in complex biological samples. *Anal. Chim. Acta* **2018,** *1035*, 108-118.
(456) Shi, C.; Meng, J.; Deng, C. Enrichment and detection of small molecules using magnetic graphene as an adsorbent and a novel matrix of MALDI-TOF-MS. *Chem. Commun. (Camb.)* **2012,** *48* (18), 2418-2420.
(457) Li, J. Y.; Long, X. Y.; Sheng, D.; Lian, H. Z. Organic molecule-assisted synthesis of Fe(3)O(4)/graphene oxide nanocomposites for selective capture of low-abundance peptides and phosphopeptides. *Talanta* **2020,** *208*, 120437.
(458) Yukird, J.; Insin, N.; Chanajaree, R.; Rodthongkum, N. Fe3O4 Nanoparticle/Graphene Oxide Composites as Selective Probes and Self-Matrixes for Pesticide Detection by Electrochemistry and Laser Desorption/Ionization Mass Spectrometry. *ACS Appl. Nano Mater.* **2023,** *6* (13), 11912-11924.
(459) Tang, H. Z.; Wang, Y. H.; Li, S.; Wu, J.; Li, J. W.; Zhou, H. Y.; Gao, Z. X. Graphene oxide composites for magnetic solid-phase extraction of twelve quinolones in water samples followed by MALDI-TOF MS. *Anal. Bioanal. Chem.* **2019,** *411* (26), 7039-7049.
(460) Tang, X.; Chen, Z.; Chen, Y.; Jiang, X.; Zhu, F.; Liu, S.; Wan, K. Hybrid bismuth oxide-graphine oxide nanomaterials improve the signal-to-noise response of small molecules analyzed by matrix assisted laser desorption ionization-time-of-flight mass spectrometry. *Talanta* **2023,** *252*, 123768.
(461) Sunner, J.; Dratz, E.; Chen, Y. C. Graphite surface-assisted laser desorption/ionization time-of-flight mass spectrometry of peptides and proteins from liquid solutions. *Anal. Chem.* **1995,** *67* (23), 4335-4342.
(462) Dale, M. J.; Knochenmuss, R.; Zenobi, R. Graphite/Liquid mixed matrices for laser desorption/ionization mass spectrometry. *Anal. Chem.* **1996,** *68* (19), 3321-3329.
(463) Cha, S.; Yeung, E. S. Colloidal graphite-assisted laser desorption/ionization mass spectrometry and MSn of small molecules. 1. Imaging of cerebrosides directly from rat brain tissue. *Anal. Chem.* **2007,** *79* (6), 2373-2385.
(464) Walton, S. L.; Mitchell, D. J. A novel rapid detection approach for the analysis of radionuclides in environmental samples using graphite MALDI mass spectrometry. *J. Radioanal. Nucl. Chem.* **2012,** *296* (2), 1113-1118.
(465) Kim, J.; Paek, K.; Kang, W. Visible surface-assisted laser desorption/ionization mass spectrometry of small macromolecules deposited on the graphite plate. *Bull. Korean Chem. Soc.* **2002,** *23* (2), 315-319.
(466) Park, K. H.; Kim, H. J. Analysis of fatty acids by graphite plate laser desorption/ionization time-of-flight mass spectrometry. *Rapid Commun. Mass Spectrom.* **2001,** *15* (16), 1494-1499.
(467) Kim, H. J.; Lee, J. K.; Park, S. J.; Ro, H. W.; Yoo, D. Y.; Yoon, D. Y. Observation of low molecular weight poly(methylsilsesquioxane)s by graphite plate laser desorption/ionization time-of-flight mass spectrometry. *Anal. Chem.* **2000,** *72* (22), 5673-5678.
(468) Black, C.; Poile, C.; Langley, J.; Herniman, J. The use of pencil lead as a matrix and calibrant for matrix-assisted laser desorption/ionisation. *Rapid Commun. Mass Spectrom.* **2006,** *20* (7), 1053-1060.
(469) Liu, Z.; Arima, K.; Nishiki, N.; Kuwabara, R.; Ishitani, S.; Matsui, T.; Tanaka, M. Graphite Sheet-Assisted Laser Desorption Ionization-Mass Spectrometry for Small Organic Compound Analysis. *ACS*

Omega **2024,** *9* (25), 27739-27747.

(470) Tanaka, M.; Arima, K.; Takeshita, T.; Kunitake, Y.; Ohno, N.; Imamura, M.; Matsui, T. Laser Desorption Ionization–Mass Spectrometry with Graphite Carbon Black Nanoparticles for Simultaneous Detection of Taste- and Odor-Active Compounds. *ACS Appl. Nano Mater.* **2022,** *5* (2), 2187-2194.

(471) Tanaka, M.; Arima, K.; Ide, H.; Koshi, M.; Ohno, N.; Imamura, M.; Matsui, T. Application of graphite carbon black assisted-laser desorption ionization-mass spectrometry for soy sauce product discrimination. *Biosci. Biotechnol. Biochem.* **2024,** *88* (6), 656-664.

(472) Greiderer, A.; Rainer, M.; Najam-ul-Haq, M.; Vallant, R. M.; Huck, C. W.; Bonn, G. K. Derivatized graphitic nanofibres (GNF) as a new support material for mass spectrometric analysis of peptides and proteins. *Amino Acids* **2009,** *37* (2), 341-348.

(473) Wei, L. M.; Xue, Y.; Zhou, X. W.; Jin, H.; Shi, Q.; Lu, H. J.; Yang, P. Y. Nanodiamond MALDI support for enhancing the credibility of identifying proteins. *Talanta* **2008,** *74* (5), 1363-1370.

(474) Hussain, D.; Najam-ul-Haq, M.; Jabeen, F.; Ashiq, M. N.; Athar, M.; Rainer, M.; Huck, C. W.; Bonn, G. K. Functionalized diamond nanopowder for phosphopeptides enrichment from complex biological fluids. *Anal. Chim. Acta* **2013,** *775*, 75-84.

(475) Ma, R.; Lu, M.; Ding, L.; Ju, H.; Cai, Z. Surface-assisted laser desorption/ionization mass spectrometric detection of biomolecules by using functional single-walled carbon nanohorns as the matrix. *Chemistry* **2013,** *19* (1), 102-108.

(476) Chen, S.; Zheng, H.; Wang, J.; Hou, J.; He, Q.; Liu, H.; Xiong, C.; Kong, X.; Nie, Z. Carbon nanodots as a matrix for the analysis of low-molecular-weight molecules in both positive- and negative-ion matrix-assisted laser desorption/ionization time-of-flight mass spectrometry and quantification of glucose and uric acid in real samples. *Anal. Chem.* **2013,** *85* (14), 6646-6652.

(477) Gedda, G.; Pandey, S.; Bhaisare, M. L.; Wu, H.-F. Carbon dots as nanoantennas for anti-inflammatory drug analysis using surface-assisted laser desorption/ionization time-of-flight mass spectrometry in serum. *RSC Adv.* **2014,** *4* (72), 38027-38033.

(478) Luo, P.; Wang, L.; Jiang, L.; Sun, J.; Li, Y.; Liu, H.; Xiong, C.; Nie, Z. Application of Graphdiyne in Surface-Assisted Laser Desorption Ionization Mass Spectrometry. *ACS Appl. Mater. Interfaces* **2021,** *13* (1), 1914-1920.

(479) Pei, J.; Zhao, Y.; Zhang, S.; Yu, X.; Tian, Z.; Sun, Y.; Ma, S.; Zhao, R. S.; Meng, J.; Chen, X.et al. A Surface Matrix of Au NPs Decorated Graphdiyne for Multifunctional Laser Desorption/Ionization Mass Spectrometry. *ACS Appl. Mater. Interfaces* **2023,** *15* (45), 52814-52826.

(480) Wang, X.; Dou, S.; Wang, Z.; Du, J.; Lu, N. Carbon nanoparticles derived from carbon soot as a matrix for SALDI-MS analysis. *Mikrochim. Acta* **2020,** *187* (3), 161.

(481) Banazadeh, A.; Peng, W.; Veillon, L.; Mechref, Y. Carbon Nanoparticles and Graphene Nanosheets as MALDI Matrices in Glycomics: a New Approach to Improve Glycan Profiling in Biological Samples. *J. Am. Soc. Mass Spectrom.* **2018,** *29* (9), 1892-1900.

(482) Luo, S.; Zhao, Z.; Wu, Q.; Wang, Y.; Lu, H. Porous Graphitic Carbon-Based Imprint Mass Spectrometry Imaging with an Ambient Liquid Extraction Technique for Enhancing Coverage of Glycerolipids and Sphingolipids in Brain Tissue. *Anal. Chem.* **2022,** *94* (40), 13753-13761.

(483) Lin, Z.; Zheng, J.; Lin, G.; Tang, Z.; Yang, X.; Cai, Z. Negative Ion Laser Desorption/Ionization Time-of-Flight Mass Spectrometric Analysis of Small Molecules Using Graphitic Carbon Nitride Nanosheet Matrix. *Anal. Chem.* **2015,** *87* (15), 8005-8012.

(484) Hosu, I. S.; Sobaszek, M.; Ficek, M.; Bogdanowicz, R.; Drobecq, H.; Boussekey, L.; Barras, A.; Melnyk, O.; Boukherroub, R.; Coffinier, Y. Carbon nanowalls: a new versatile graphene based interface for the

laser desorption/ionization-mass spectrometry detection of small compounds in real samples. *Nanoscale* **2017,** *9* (27), 9701-9715.
(485) Hosu, I. S.; Sobaszek, M.; Ficek, M.; Bogdanowicz, R.; Coffinier, Y. Boron-doped carbon nanowalls for fast and direct detection of cytochrome C and ricin by matrix-free laser desorption/ionization mass spectrometry. *Talanta* **2023,** *252*, 123778.
(486) Kraj, A.; Jarzebinska, J.; Gorecka-Drzazga, A.; Dziuban, J.; Silberring, J. Identification of catecholamines in the immune system by desorption/ionization on silicon. *Rapid Commun. Mass Spectrom.* **2006,** *20* (13), 1969-1972.
(487) Patti, G. J.; Woo, H. K.; Yanes, O.; Shriver, L.; Thomas, D.; Uritboonthai, W.; Apon, J. V.; Steenwyk, R.; Manchester, M.; Siuzdak, G. Detection of carbohydrates and steroids by cation-enhanced nanostructure-initiator mass spectrometry (NIMS) for biofluid analysis and tissue imaging. *Anal. Chem.* **2010,** *82* (1), 121-128.
(488) Gao, J.; de Raad, M.; Bowen, B. P.; Zuckermann, R. N.; Northen, T. R. Application of Black Silicon for Nanostructure-Initiator Mass Spectrometry. *Anal. Chem.* **2016,** *88* (3), 1625-1630.
(489) Northen, T. R.; Lee, J. C.; Hoang, L.; Raymond, J.; Hwang, D. R.; Yannone, S. M.; Wong, C. H.; Siuzdak, G. A nanostructure-initiator mass spectrometry-based enzyme activity assay. *Proc. Natl. Acad. Sci. U. S. A.* **2008,** *105* (10), 3678-3683.
(490) Deng, K.; George, K. W.; Reindl, W.; Keasling, J. D.; Adams, P. D.; Lee, T. S.; Singh, A. K.; Northen, T. R. Encoding substrates with mass tags to resolve stereospecific reactions using Nimzyme. *Rapid Commun. Mass Spectrom.* **2012,** *26* (6), 611-615.
(491) Wen, X.; Dagan, S.; Wysocki, V. H. Small-molecule analysis with silicon-nanoparticle-assisted laser desorption/ionization mass spectrometry. *Anal. Chem.* **2007,** *79* (2), 434-444.
(492) Araujo, P.; Ferreira, M. S.; de Oliveira, D. N.; Pereira, L.; Sawaya, A. C.; Catharino, R. R.; Mazzafera, P. Mass spectrometry imaging: an expeditious and powerful technique for fast in situ lignin assessment in Eucalyptus. *Anal. Chem.* **2014,** *86* (7), 3415-3419.
(493) Zhu, X.; Wu, L.; Mungra, D. C.; Xia, S.; Zhu, J. Au@SiO2 core-shell nanoparticles for laser desorption/ionization time of flight mass spectrometry. *Analyst* **2012,** *137* (10), 2454-2458.
(494) Li, Y.; Zhang, H.; Jiang, J.; Zhao, L.; Wang, Y. SiO(2)@Au nanoshell-assisted laser desorption/ionization mass spectrometry for coronary heart disease diagnosis. *J. Mater. Chem. B* **2023,** *11* (13), 2862-2871.
(495) Choi, Y. K.; Cheon, D. H.; Yang, W. S.; Baek, J. H. A Graphene-Coated Silicon Wafer Plate Improves the Sensitivity and Reproducibility of MALDI-TOF MS Analysis of Proteins. *J. Am. Soc. Mass Spectrom.* **2023,** *34* (9), 2034-2042.
(496) Go, E. P.; Apon, J. V.; Luo, G.; Saghatelian, A.; Daniels, R. H.; Sahi, V.; Dubrow, R.; Cravatt, B. F.; Vertes, A.; Siuzdak, G. Desorption/ionization on silicon nanowires. *Anal. Chem.* **2005,** *77* (6), 1641-1646.
(497) Muck, A.; Stelzner, T.; Hubner, U.; Christiansen, S.; Svatos, A. Lithographically patterned silicon nanowire arrays for matrix free LDI-TOF/MS analysis of lipids. *Lab Chip* **2010,** *10* (3), 320-325.
(498) Piret, G.; Drobecq, H.; Coffinier, Y.; Melnyk, O.; Boukherroub, R. Matrix-free laser desorption/ionization mass spectrometry on silicon nanowire arrays prepared by chemical etching of crystalline silicon. *Langmuir* **2010,** *26* (2), 1354-1361.
(499) Liu, X.; Tao, L.; Jiang, X.; Qu, X.; Duan, W.; Yu, J.; Liang, X.; Wu, J. Isoporous Membrane Mediated Imprinting Mass Spectrometry Imaging for Spatially-Resolved Metabolomics and Rapid Histopathological Diagnosis. *Small Methods* **2024,** *8* (10), e2301644.
(500) Jiang, X.; Liu, X.; Qu, X.; Zhu, P.; Wo, F.; Xu, X.; Jin, J.; He, Q.; Wu, J. Integration of metabolomics and peptidomics reveals distinct molecular landscape of human diabetic kidney disease. *Theranostics*

2023, *13* (10), 3188-3203.
(501) Walker, B. N.; Antonakos, C.; Retterer, S. T.; Vertes, A. Metabolic differences in microbial cell populations revealed by nanophotonic ionization. *Angew. Chem. Int. Ed. Engl.* **2013,** *52* (13), 3650-3653.
(502) Fincher, J. A.; Korte, A. R.; Dyer, J. E.; Yadavilli, S.; Morris, N. J.; Jones, D. R.; Shanmugam, V. K.; Pirlo, R. K.; Vertes, A. Mass spectrometry imaging of triglycerides in biological tissues by laser desorption ionization from silicon nanopost arrays. *J. Mass Spectrom.* **2020,** *55* (4), e4443.
(503) Fincher, J. A.; Dyer, J. E.; Korte, A. R.; Yadavilli, S.; Morris, N. J.; Vertes, A. Matrix-free mass spectrometry imaging of mouse brain tissue sections on silicon nanopost arrays. *J. Comp. Neurol.* **2019,** *527* (13), 2101-2121.
(504) Samarah, L. Z.; Vertes, A. In *Mass Spectrometry Imaging of Small Molecules: Methods and Protocols*; Springer, 2021; p 89-98.
(505) Duan, J.; Linman, M. J.; Cheng, Q. Ultrathin calcinated films on a gold surface for highly effective laser desorption/ionization of biomolecules. *Anal. Chem.* **2010,** *82* (12), 5088-5094.
(506) Duan, J.; Wang, H.; Cheng, Q. On-plate desalting and SALDI-MS analysis of peptides with hydrophobic silicate nanofilms on a gold substrate. *Anal. Chem.* **2010,** *82* (22), 9211-9220.
(507) Barros, R. M.; Clemente, M. C. H.; Martins, G. A. V.; Silva, L. P. Application of mesocellular siliceous foams (MCF) for surface-assisted laser desorption ionization mass spectrometry (SALDI-MS) Analysis of fingermarks. *Sci. Justice* **2018,** *58* (4), 264-270.
(508) Tsao, C. W.; Kumar, P.; Liu, J.; DeVoe, D. L. Dynamic electrowetting on nanofilament silicon for matrix-free laser desorption/ionization mass spectrometry. *Anal. Chem.* **2008,** *80* (8), 2973-2981.
(509) Jaschinski, T.; Svatos, A.; Pohnert, G. Laser desorption/ionization mediated by bionanostructures from microalgae. *Rapid Commun. Mass Spectrom.* **2013,** *27* (1), 109-116.
(510) Jaschinski, T.; Thume, K.; Klein, M.; Richter, P.; Popp, J.; Svatos, A.; Pohnert, G. Enhanced signal intensity in matrix-free laser desorption ionization mass spectrometry by chemical modification of bionanostructures from diatom cell walls. *Rapid Commun. Mass Spectrom.* **2014,** *28* (13), 1521-1529.
(511) Shih, Y. H.; Chien, C. H.; Singco, B.; Hsu, C. L.; Lin, C. H.; Huang, H. Y. Metal-organic frameworks: new matrices for surface-assisted laser desorption-ionization mass spectrometry. *Chem. Commun. (Camb.)* **2013,** *49* (43), 4929-4931.
(512) Fu, C. P.; Lirio, S.; Liu, W. L.; Lin, C. H.; Huang, H. Y. A novel type of matrix for surface-assisted laser desorption-ionization mass spectrometric detection of biomolecules using metal-organic frameworks. *Anal. Chim. Acta* **2015,** *888*, 103-109.
(513) Han, G.; Zeng, Q.; Jiang, Z.; Xing, T.; Huang, C.; Li, Y. MIL-101(Cr) as matrix for sensitive detection of quercetin by matrix-assisted laser desorption/ionization mass spectrometry. *Talanta* **2017,** *164*, 355-361.
(514) Chang, Y. J.; Yang, S. S.; Yu, X.; Zhang, H.; Shang, W.; Gu, Z. Y. Ultrahigh efficient laser desorption ionization of saccharides by Ti-based metal-organic frameworks nanosheets. *Anal. Chim. Acta* **2018,** *1032*, 91-98.
(515) Yang, X.; Lin, Z.; Yan, X.; Cai, Z. Zeolitic imidazolate framework nanocrystals for enrichment and direct detection of environmental pollutants by negative ion surface-assisted laser desorption/ionization time-of-flight mass spectrometry. *RSC Adv.* **2016,** *6* (28), 23790-23793.
(516) Lu, L.; Qian, X.; Li, F.; Qin, S.; Luo, Y.; Tang, J.; Zhou, K.; Zheng, G. A mesoporous graphene @ zirconium-based metal-organic frameworks as a matrix and an adsorbent for steroid detection using surface-assisted laser desorption/ionization time-of-flight mass spectrometry. *J. Chromatogr. A* **2023,** *1696*, 463963.

(517) Shih, Y. H.; Fu, C. P.; Liu, W. L.; Lin, C. H.; Huang, H. Y.; Ma, S. Nanoporous Carbons Derived from Metal-Organic Frameworks as Novel Matrices for Surface-Assisted Laser Desorption/Ionization Mass Spectrometry. *Small* **2016,** *12* (15), 2057-2066.

(518) Luo, Y.; Zhao, X.; Gao, Z.; Wang, H.; Liu, Y.; Guo, C.; Pan, Y. Pd nanoparticles decorated thiol-functionalized MOF as an efficient matrix for differentiation and quantitation of oligosaccharide isomers by laser desorption/ionization mass spectrometry. *Anal. Chim. Acta* **2022,** *1202*, 339665.

(519) Li, Z.; Huo, P.; Gong, C.; Deng, C.; Pu, S. Boric-acid-modified Fe(3)O(4)@PDA@UiO-66 for enrichment and detection of glucose by matrix-assisted laser desorption/ionization time-of-flight mass spectrometry. *Anal. Bioanal. Chem.* **2020,** *412* (29), 8083-8092.

(520) Li, Z.; Liu, Q.; Lu, X.; Deng, C.; Sun, N.; Yang, X. Magnetic metal-organic framework nanocomposites for enrichment and direct detection of environmental pollutants by negative-ion matrix-assisted laser desorption/ionization time-of-flight mass spectrometry. *Talanta* **2019,** *194*, 329-335.

(521) Lin, Z.; Bian, W.; Zheng, J.; Cai, Z. Magnetic metal-organic framework nanocomposites for enrichment and direct detection of small molecules by negative-ion matrix-assisted laser desorption/ionization time-of-flight mass spectrometry. *Chem. Commun. (Camb.)* **2015,** *51* (42), 8785-8788.

(522) Chen, Y.; Zhang, M.; Qi, Y.; Lin, Y.; Liu, S.; Deng, C.; Jiang, S.; Sun, N. Efficient extraction via titanium organic frameworks facilitates in-depth profiling of urinary exosome metabolite fingerprints. *Anal. Bioanal. Chem.* **2025,** *417* (8), 1543-1555.

(523) Lin, H.; Yan, Y.; Deng, C.; Sun, N. Engineered Bimetallic MOF-Crafted Bullet Aids in Penetrating Serum Metabolic Traits of Chronic Obstructive Pulmonary Disease. *Anal. Chem.* **2024,** *96* (36), 14688-14696.

(524) Yin, S.-J.; Chen, H.; Wang, S.; Wang, Y.; Yang, F.-Q. Preparation of core-shell MOF@ MOF nanoparticle as matrix for the analysis of rhubarb anthraquinones in plasma by matrix-assisted laser desorption/ionization time-of-flight mass spectrometry. *Heliyon* **2023,** *9* (5), e16245.

(525) Ma, W.; Xu, S.; Ai, W.; Lin, C.; Bai, Y.; Liu, H. A flexible and multifunctional metal-organic framework as a matrix for analysis of small molecules using laser desorption/ionization mass spectrometry. *Chem. Commun. (Camb.)* **2019,** *55* (48), 6898-6901.

(526) Ma, W.; Yang, B.; Li, J.; Liu, M.; Li, X.; Liu, H. Maltose-functional metal-organic framework assisted laser desorption/ionization mass spectrometry for small biomolecule determination. *Mikrochim. Acta* **2022,** *189* (7), 253.

(527) Zheng, R.; Yang, Y.; Xia, Y. NH(2)NH-MOF: a reaction matrix for the specific determination of small aldehydes by MALDI-MS. *Mikrochim. Acta* **2022,** *189* (2), 51.

(528) Feng, D.; Xia, Y. Covalent organic framework as efficient desorption/ionization matrix for direct detection of small molecules by laser desorption/ionization mass spectrometry. *Anal. Chim. Acta* **2018,** *1014*, 58-63.

(529) Wang, S.; Niu, H.; Cao, D.; Cai, Y. Covalent-organic frameworks as adsorbent and matrix of SALDI-TOF MS for the enrichment and rapid determination of fluorochemicals. *Talanta* **2019,** *194*, 522-527.

(530) Ouyang, D.; Luo, K.; Ma, W.; Wu, J.; Li, J.; He, Y.; Cai, Z.; Lin, Z. A spherical covalent-organic framework for enhancing laser desorption/ionization mass spectrometry for small molecule detection. *Analyst* **2020,** *145* (8), 3125-3130.

(531) Ouyang, D.; Zheng, Q.; Huang, H.; Cai, Z.; Lin, Z. Covalent Organic Framework Nanofilm-Based Laser Desorption/Ionization Mass Spectrometry for 5-Fluorouracil Analysis and Tissue Imaging. *Anal. Chem.* **2021,** *93* (47), 15573-15578.

(532) Chen, Z.; Wu, Y.; Ouyang, D.; Lin, Z. Covalent organic framework film-based laser desorption/ionization mass spectrometry for rapid and sensitive quantification of homocysteine in human serum. *Rapid*

Commun. Mass Spectrom. **2023,** *37* (5), e9463.
(533) Hu, K.; Lv, Y.; Ye, F.; Chen, T.; Zhao, S. Boric-Acid-Functionalized Covalent Organic Framework for Specific Enrichment and Direct Detection of cis-Diol-Containing Compounds by Matrix-Assisted Laser Desorption/Ionization Time-of-Flight Mass Spectrometry. *Anal. Chem.* **2019,** *91* (9), 6353-6362.
(534) Tan, W.; Xu, X.; Lv, Y.; Lei, W.; Hu, K.; Ye, F.; Zhao, S. Sulfonic acid functionalized hierarchical porous covalent organic frameworks as a SALDI-TOF MS matrix for effective extraction and detection of paraquat and diquat. *J. Colloid Interface Sci.* **2021,** *603*, 172-181.
(535) Zhang, Y.; Song, Y.; Wu, J.; Li, R.; Hu, D.; Lin, Z.; Cai, Z. A magnetic covalent organic framework as an adsorbent and a new matrix for enrichment and rapid determination of PAHs and their derivatives in PM(2.5) by surface-assisted laser desorption/ionization-time of flight-mass spectrometry. *Chem. Commun. (Camb.)* **2019,** *55* (26), 3745-3748.
(536) Ma, Y. F.; Wang, L. J.; Zhou, Y. L.; Zhang, X. X. A facilely synthesized glutathione-functionalized silver nanoparticle-grafted covalent organic framework for rapid and highly efficient enrichment of N-linked glycopeptides. *Nanoscale* **2019,** *11* (12), 5526-5534.
(537) Ding, F.; Chu, Z.; Zhang, Q.; Liu, H.; Zhang, W. Facile synthesis of layered mesoporous covalent organic polymers for highly selective enrichment of N-glycopeptides. *Anal. Chim. Acta* **2019,** *1057*, 145-151.
(538) Wang, B.; Liu, J.; Yan, Y.; Ding, C. F.; Tang, K. Post-synthesis of boric acid-functionalized magnetic covalent organic framework as an affinity probe for the enrichment of N-glycopeptides. *Mikrochim. Acta* **2021,** *188* (10), 336.
(539) Ouyang, D.; Dan, A.; Lin, Z.; Cai, Z. Spherical covalent-organic framework-assisted laser desorption ionization mass spectrometry reveals the promotional effect of triphenyl phosphate on breast cancer in mice. *Sci. Total Environ.* **2024,** *955*, 177155.
(540) Jin, Y.; Chen, J.; Xie, W.; Zhang, J.; Yan, J.; Chen, C.; Lin, J.; Cai, Z.; Lin, Z. Gold-Modified Covalent Organic Frameworks-Assisted Laser Desorption/Ionization Mass Spectrometry for Analysis of Metabolites Induced by Triclosan Exposure. *ACS Appl. Mater. Interfaces* **2025,** *17* (4), 7056-7065.
(541) Su, H.; Chen, Z.; Lin, J.; Zhong, Y.; Ouyang, D.; Lin, Z. Donor-acceptor covalent organic framework nanofilm-based laser desorption/ionization mass spectrometry for rapid and sensitive determination of creatinine in human serum. *Analyst* **2025,** *150* (11), 2288-2294.
(542) Luo, Y.; Ma, S.; Zhang, J.; Zhang, Q.; Zhang, Y.; Mao, J.; Yuan, H.; Ouyang, G.; Zhang, S.; Zhao, W. Developing a novel strategy for fabricating matrix film to assess the distribution of potassium perfluorooctanic sulfonate by matrix-assisted laser desorption/ionization mass spectrometry imaging. *Anal. Chim. Acta* **2024,** *1303*, 342528.
(543) Yin, S. J.; Zheng, G. C.; Yi, X.; Lv, G. P.; Yang, F. Q. A metal-organic framework@hydrogen-bond framework as a matrix for MALDI-TOF-MS analysis of small molecules. *Chem. Commun. (Camb.)* **2022,** *58* (47), 6701-6704.
(544) Shrivas, K.; Kailasa, S. K.; Wu, H. F. Quantum dots laser desorption/ionization MS: multifunctional CdSe quantum dots as the matrix, concentrating probes and acceleration for microwave enzymatic digestion for peptide analysis and high resolution detection of proteins in a linear MALDI-TOF MS. *Proteomics* **2009,** *9* (10), 2656-2667.
(545) Shastri, L. A.; Kailasa, S. K.; Wu, H. F. Cysteine-capped ZnSe quantum dots as affinity and accelerating probes for microwave enzymatic digestion of proteins via direct matrix-assisted laser desorption/ionization time-of-flight mass spectrometric analysis. *Rapid Commun. Mass Spectrom.* **2009,** *23* (15), 2247-2252.
(546) Wu, H. F.; Chung, F. T. 3-Mercaptopropionic acid modified ZnSe quantum dots as the matrix for direct

surface-assisted laser desorption/ionization mass spectrometric analysis of peptides/proteins from sodium salt solution. *Rapid Commun. Mass Spectrom.* **2011,** *25* (12), 1779-1786.

(547) Bibi, A.; Ju, H. Quantum dots assisted laser desorption/ionization mass spectrometric detection of carbohydrates: qualitative and quantitative analysis. *J. Mass Spectrom.* **2016,** *51* (4), 291-297.

(548) Seino, T.; Sato, H.; Yamamoto, A.; Nemoto, A.; Torimura, M.; Tao, H. Matrix-free laser desorption/ionization-mass spectrometry using self-assembled germanium nanodots. *Anal. Chem.* **2007,** *79* (13), 4827-4832.

(549) Jin, Z.; Liu, M.; Huang, X.; Zhang, X.; Qu, Z.; Zhu, J. J.; Min, Q. Top-Down Rational Engineering of Heteroatom-Doped Graphene Quantum Dots for Laser Desorption/Ionization Mass Spectrometry Detection and Imaging of Small Biomolecules. *Anal. Chem.* **2022,** *94* (21), 7609-7618.

(550) Zhao, Y.; Boukherroub, R.; Liu, L.; Li, H.; Zhao, R. S.; Wei, Q.; Yu, X.; Chen, X. Boron nitride quantum dots-enhanced laser desorption/ionization mass spectrometry analysis and imaging of bisphenol A. *J. Hazard. Mater.* **2023,** *459*, 132336.

(551) Peng, Z.; Zhang, Y.; Dong, Y.; Zhao, W.; Mao, J.; Zhang, Q.; Ouyang, G.; Zhang, S.; Xie, J. A cheap 3D-printed boron, nitrogen, sulfur-MXene quantum dots-based target plate for multi-dimensional MALDI MS imaging of small-molecule environmental pollutants. *Chem. Eng. J.* **2025,** *507*, 160596.

(552) Feenstra, A. D.; O'Neill, K. C.; Yagnik, G. B.; Lee, Y. J. Organic–inorganic binary mixture matrix for comprehensive laser-desorption ionization mass spectrometric analysis and imaging of medium-size molecules including phospholipids, glycerolipids, and oligosaccharides. *RSC Adv.* **2016,** *6* (101), 99260-99268.

(553) Chen, Y.; Gao, D.; Bai, H.; Liu, H.; Lin, S.; Jiang, Y. Carbon Dots and 9AA as a Binary Matrix for the Detection of Small Molecules by Matrix-Assisted Laser Desorption/Ionization Mass Spectrometry. *J. Am. Soc. Mass Spectrom.* **2016,** *27* (7), 1227-1235.

(554) Hou, J.; Chen, S.; Cao, C.; Liu, H.; Xiong, C.; Zhang, N.; He, Q.; Song, W.; Nie, Z. Application of flowerlike MgO for highly sensitive determination of lead via matrix-assisted laser desorption/ionization mass spectrometry. *Rapid Commun. Mass Spectrom.* **2016,** *30* (S1), 208-216.

(555) Wang, M.; Ling, L.; Wang, S.; Ding, C. F. A homogeneous binary matrix assisted laser desorption/ionization time-of-flight mass spectrometry assay for determination of artificial sweeteners in beverages. *Food Chem.* **2024,** *460* (Pt 2), 140597.

(556) Dufresne, M.; Fincher, J. A.; Patterson, N. H.; Schey, K. L.; Norris, J. L.; Caprioli, R. M.; Spraggins, J. M. alpha-Cyano-4-hydroxycinnamic Acid and Tri-Potassium Citrate Salt Pre-Coated Silicon Nanopost Array Provides Enhanced Lipid Detection for High Spatial Resolution MALDI Imaging Mass Spectrometry. *Anal. Chem.* **2021,** *93* (36), 12243-12249.

(557) Zhao, Q.; Xu, J.; Yin, J.; Feng, Y. Q. Humic acids as both matrix for matrix-assisted laser desorption/ionization time-of-flight mass spectrometry and adsorbent for magnetic solid phase extraction. *Anal. Chim. Acta* **2015,** *889*, 138-146.

(558) Tang, H.-z.; Ma, Y.-l.; Liu, F.; Liu, F.; Liu, Z.-w.; Li, J.-w.; Zhou, H.-y.; Gao, Z.-x. Detection of small molecules using SBA-15 modified CHCA as a novel matrix of MALDI-TOF MS. *Int. J. Mass spectrom.* **2017,** *417*, 34-39.

(559) Sun, R.; Zhang, Y.; Tang, W.; Li, B. Submicron 3,4-dihydroxybenzoic acid-TiO(2) composite particles for enhanced MALDI MS imaging of secondary metabolites in the root of differently aged baical skullcap. *Analyst* **2022,** *147* (13), 3017-3024.

(560) Zhang, X.; Ji, X.; Peng, Z.; Zhang, Y.; Cai, Z.; Zhang, S. p-AAB/MXene as a novel adsorbent and SALDI matrix for highly efficient enrichment and rapid MS detection of emerging environmental organic

pollutants in beverages and PM(2.5). *J. Chromatogr. A* **2025,** *1745*, 465759.
(561) Chitanda, J. M.; Zhang, H.; Pahl, E.; Purves, R. W.; El-Aneed, A. The Development of Novel Nanodiamond Based MALDI Matrices for the Analysis of Small Organic Pharmaceuticals. *J. Am. Soc. Mass Spectrom.* **2016,** *27* (10), 1686-1693.
(562) Fleith, C.; Cantel, S.; Subra, G.; Mehdi, A.; Ciccione, J.; Martinez, J.; Enjalbal, C. Laser desorption ionization mass spectrometry of peptides on a hybrid CHCA organic-inorganic matrix. *Analyst* **2014,** *139* (15), 3748-3754.
(563) Duan, J.; Linman, M. J.; Chen, C. Y.; Cheng, Q. J. CHCA-modified Au nanoparticles for laser desorption ionization mass spectrometric analysis of peptides. *J. Am. Soc. Mass Spectrom.* **2009,** *20* (8), 1530-1539.
(564) Asano, T.; Suzuki, J.; Hashimoto, K.; Fujino, T. Laser desorption ionization mass spectrometry of bioactive substances by using 2,4,6-trihydroxyacetophenone on cation-substituted zeolite. *Anal. Sci.* **2013,** *29* (11), 1035-1039.
(565) Springer, V.; Zhou, Y.; Aguilera, A. Y.; Emmer, A. User-friendly platform for analysis of high mass intact proteins and glycopeptides by laser desorption/ionization-mass spectrometry based on copper oxide particles. *Anal. Bioanal. Chem.* **2024,** *416* (4), 861-872.
(566) Liu, H.; Dai, J.; Zhou, J.; Huang, H.; Chen, F.; Liu, Z. A hybrid ionic liquid–matrix material, [TiO 2 – Si–NH 3 +][CHC −], as a novel matrix for the analysis of small molecules by MALDI-TOF MS. *Int. J. Mass spectrom.* **2015,** *376*, 85-89.
(567) Su, X.; Zhou, H.-Y.; Chen, F.-C.; Gao, B.-X.; Liu, Z.-W.; Zhang, Y.-H.; Liu, F.; Liu, F.; Li, Z.-R.; Gao, Z.-X. Modified SBA-15 matrices for high-throughput screening of melamine in milk samples by MALDI-TOF MS. *Int. J. Mass spectrom.* **2013,** *338*, 39-44.
(568) Mullens, C. P.; Anugu, S. R.; Gorski, W.; Bach, S. B. H. Modified silica-containing matrices towards the MALDI-TOF-MS detection of small molecules. *Int. J. Mass spectrom.* **2011,** *308* (2-3), 311-315.
(569) Lin, P. C.; Tseng, M. C.; Su, A. K.; Chen, Y. J.; Lin, C. C. Functionalized magnetic nanoparticles for small-molecule isolation, identification, and quantification. *Anal. Chem.* **2007,** *79* (9), 3401-3408.
(570) Obena, R. P.; Lin, P. C.; Lu, Y. W.; Li, I. C.; del Mundo, F.; Arco, S.; Nuesca, G. M.; Lin, C. C.; Chen, Y. J. Iron oxide nanomatrix facilitating metal ionization in matrix-assisted laser desorption/ionization mass spectrometry. *Anal. Chem.* **2011,** *83* (24), 9337-9343.
(571) Tseng, M. C.; Obena, R.; Lu, Y. W.; Lin, P. C.; Lin, P. Y.; Yen, Y. S.; Lin, J. T.; Huang, L. D.; Lu, K. L.; Lai, L. L.et al. Dihydrobenzoic acid modified nanoparticle as a MALDI-TOF MS matrix for soft ionization and structure determination of small molecules with diverse structures. *J. Am. Soc. Mass Spectrom.* **2010,** *21* (11), 1930-1939.
(572) Ouyang, D.; Wang, C.; Zhong, C.; Lin, J.; Xu, G.; Wang, G.; Lin, Z. Organic metal chalcogenide-assisted metabolic molecular diagnosis of central precocious puberty. *Chem. Sci.* **2023,** *15* (1), 278-284.
(573) Shroff, R.; Svatos, A. Proton sponge: a novel and versatile MALDI matrix for the analysis of metabolites using mass spectrometry. *Anal. Chem.* **2009,** *81* (19), 7954-7959.
(574) Szabo, Z.; Vallant, R. M.; Takatsy, A.; Bakry, R.; Najam-ul-Haq, M.; Rainer, M.; Huck, C. W.; Bonn, G. K. Laser desorption/ionization mass spectrometric analysis of small molecules using fullerene-derivatized silica as energy-absorbing material. *J. Mass Spectrom.* **2010,** *45* (5), 545-552.
(575) Kuo, T. R.; Wang, D. Y.; Chiu, Y. C.; Yeh, Y. C.; Chen, W. T.; Chen, C. H.; Chen, C. W.; Chang, H. C.; Hu, C. C.; Chen, C. C. Layer-by-layer thin film of reduced graphene oxide and gold nanoparticles as an effective sample plate in laser-induced desorption/ionization mass spectrometry. *Anal. Chim. Acta* **2014,** *809*, 97-103.
(576) Wu, H. F.; Gopal, J.; Abdelhamid, H. N.; Hasan, N. Quantum dot applications endowing novelty to

analytical proteomics. *Proteomics* **2012,** *12* (19-20), 2949-2961.
(577) Yang, S. S.; Shi, M. Y.; Tao, Z. R.; Wang, C.; Gu, Z. Y. Recent applications of metal-organic frameworks in matrix-assisted laser desorption/ionization mass spectrometry. *Anal. Bioanal. Chem.* **2019,** *411* (19), 4509-4522.
(578) Pirkl, A.; Soltwisch, J.; Draude, F.; Dreisewerd, K. Infrared matrix-assisted laser desorption/ionization orthogonal-time-of-flight mass spectrometry employing a cooling stage and water ice as a matrix. *Anal. Chem.* **2012,** *84* (13), 5669-5676.
(579) Yazdabadi, S. H.; Farrokhpour, H.; Tabrizchi, M. Using surfactants as matrix for the matrix-assisted laser desorption/ionization time of flight mass spectrometry (MALDI-TOF-MS) of amino acids: Sodium dodecyl sulfate (SDS) and sodium octyl sulfate (SOS). *Biophys. Chem.* **2021,** *278*, 106667.
(580) Zhang, J.; Wang, H.-Y.; Guo, Y.-L. Amino Acids Analysis by MALDI Mass Spectrometry Using Carbon Nanotube as Matrix. *Chin. J. Chem* . **2005,** *23* (2), 185-189.
(581) Marsico, A. L.; Creran, B.; Duncan, B.; Elci, S. G.; Jiang, Y.; Onasch, T. B.; Wormhoudt, J.; Rotello, V. M.; Vachet, R. W. Inkjet-printed gold nanoparticle surfaces for the detection of low molecular weight biomolecules by laser desorption/ionization mass spectrometry. *J. Am. Soc. Mass Spectrom.* **2015,** *26* (11), 1931-1937.
(582) Wu, Z.; Fernandez-Lima, F. A.; Perez, L. M.; Russell, D. H. A new copper containing MALDI matrix that yields high abundances of [peptide + Cu]+ ions. *J. Am. Soc. Mass Spectrom.* **2009,** *20* (7), 1263-1271.
(583) Choi, H.; Lee, D.; Kim, Y.; Nguyen, H. Q.; Han, S.; Kim, J. Effects of Matrices and Additives on Multiple Charge Formation of Proteins in MALDI-MS Analysis. *J. Am. Soc. Mass Spectrom.* **2019,** *30* (7), 1174-1178.
(584) Fang, X.; Zhang, K.; Yang, P.; Qiao, L.; Liu, B. Sensitive and fast beverage/fruit antioxidant evaluation by TiO2 -Au/graphene nanocomposites coupled with MALDI-MS. *Rapid Commun. Mass Spectrom.* **2016,** *30 Suppl 1*, 128-132.
(585) Bailes, J.; Vidal, L.; Ivanov, D. A.; Soloviev, M. Quantum dots improve peptide detection in MALDI MS in a size dependent manner. *J. Nanobiotechnol.* **2009,** *7*, 10.
(586) Nabetani, T.; Miyazaki, K.; Tabuse, Y.; Tsugita, A. Analysis of acidic peptides with a matrix-assisted laser desorption/ionization mass spectrometry using positive and negative ion modes with additive monoammonium phosphate. *Proteomics* **2006,** *6* (16), 4456-4465.
(587) Chen, C. T.; Chen, Y. C. A two-matrix system for MALDI MS analysis of serine phosphorylated peptides concentrated by Fe3O4/Al2O3 magnetic nanoparticles. *J. Mass Spectrom.* **2008,** *43* (4), 538-541.
(588) Hou, J.; Xie, Z.; Xue, P.; Cui, Z.; Chen, X.; Li, J.; Cai, T.; Wu, P.; Yang, F. Enhanced MALDI-TOF MS analysis of phosphopeptides using an optimized DHAP/DAHC matrix. *J. Biomed. Biotechnol.* **2010,** *2010*, 759690.
(589) Fukuyama, Y.; Takeyama, K.; Kawabata, S.; Iwamoto, S.; Tanaka, K. An optimized matrix-assisted laser desorption/ionization sample preparation using a liquid matrix, 3-aminoquinoline/alpha-cyano-4-hydroxycinnamic acid, for phosphopeptides. *Rapid Commun. Mass Spectrom.* **2012,** *26* (20), 2454-2460.
(590) Fukuyama, Y.; Nakajima, C.; Izumi, S.; Tanaka, K. Membrane Protein Analyses Using Alkylated Trihydroxyacetophenone (ATHAP) as a MALDI Matrix. *Anal. Chem.* **2016,** *88* (3), 1688-1695.
(591) Lidgard, R.; Duncan, M. W. Utility of matrix-assisted laser desorption/ionization time-of-flight mass spectrometry for the analysis of low molecular weight compounds. *Rapid Commun. Mass Spectrom.* **1995,** *9* (2), 128-132.
(592) Fagerer, S. R.; Nielsen, S.; Ibanez, A.; Zenobi, R. Matrix-assisted laser desorption/ionization matrices

for negative mode metabolomics. *Eur. J. Mass Spectrom. (Chichester)* **2013,** *19* (1), 39-47.

(593) Krivosheina, M. S.; Borisov, R. S.; Zhilyaev, D. I.; Matveeva, M. D.; Zaikin, V. G. New suitable deprotonating matrices for the analysis of carboxylic acids and some acidic compounds by matrix-assisted laser desorption/ionization mass spectrometry in negative ion mode. *Rapid Commun. Mass Spectrom.* **2021,** *35* (1), e8954.

(594) Zhu, Z.; Shen, J.; Wang, D.; Chen, C.; Xu, Y.; Guo, H.; Kang, D.; Hamada, N.; Dong, J.; Wang, G.et al. An auxiliary matrix for routine analysis of small molecules and biological macromolecules using matrix-assisted laser desorption ionization mass spectrometry. *Anal. Bioanal. Chem.* **2019,** *411* (5), 1041-1052.

(595) Shariatgorji, M.; Nilsson, A.; Goodwin, R. J.; Kallback, P.; Schintu, N.; Zhang, X.; Crossman, A. R.; Bezard, E.; Svenningsson, P.; Andren, P. E. Direct targeted quantitative molecular imaging of neurotransmitters in brain tissue sections. *Neuron* **2014,** *84* (4), 697-707.

(596) Cao, Q.; Wang, Y.; Chen, B.; Ma, F.; Hao, L.; Li, G.; Ouyang, C.; Li, L. Visualization and Identification of Neurotransmitters in Crustacean Brain via Multifaceted Mass Spectrometric Approaches. *ACS Chem. Neurosci.* **2019,** *10* (3), 1222-1229.

(597) Tang, H. W.; Wong, M. Y.; Lam, W.; Cheng, Y. C.; Che, C. M.; Ng, K. M. Molecular histology analysis by matrix-assisted laser desorption/ionization imaging mass spectrometry using gold nanoparticles as matrix. *Rapid Commun. Mass Spectrom.* **2011,** *25* (24), 3690-3696.

(598) Chen, C.; Laviolette, S. R.; Whitehead, S. N.; Renaud, J. B.; Yeung, K. K. Imaging of Neurotransmitters and Small Molecules in Brain Tissues Using Laser Desorption/Ionization Mass Spectrometry Assisted with Zinc Oxide Nanoparticles. *J. Am. Soc. Mass Spectrom.* **2021,** *32* (4), 1065-1079.

(599) Qi, Y.; Muller, M.; Stokes, C. S.; Volmer, D. A. Rapid Quantification of 25-Hydroxyvitamin D(3) in Human Serum by Matrix-Assisted Laser Desorption/Ionization Mass Spectrometry. *J. Am. Soc. Mass Spectrom.* **2018,** *29* (7), 1456-1462.

(600) Chen, Y. T.; Ling, Y. C. Detection of water-soluble vitamins by matrix-assisted laser desorption/ionization time-of-flight mass spectrometry using porphyrin matrices. *J. Mass Spectrom.* **2002,** *37* (7), 716-730.

(601) Noh, J.-Y.; Kim, M.-J.; Park, J.-M.; Yun, T. G.; Kang, M.-J.; Pyun, J.-C. Quantitative analysis of vitamin D using m/MALDI-TOF mass spectrometry based on a parylene matrix chip. *J. Anal. Sci. Technol.* **2022,** *13* (1), 3.

(602) Wu, W.; Liang, Z.; Zhao, Z.; Cai, Z. Direct analysis of alkaloid profiling in plant tissue by using matrix-assisted laser desorption/ionization mass spectrometry. *J. Mass Spectrom.* **2007,** *42* (1), 58-69.

(603) Ha, M.; Kwak, J. H.; Kim, Y.; Zee, O. P. Direct analysis for the distribution of toxic glycoalkaloids in potato tuber tissue using matrix-assisted laser desorption/ionization mass spectrometric imaging. *Food Chem.* **2012,** *133* (4), 1155-1162.

(604) Jaber, A.; Seraphin, D.; Guilet, D.; Osuga, J.; Cheble, E.; Ibrahim, G.; Richomme, P.; Schinkovitz, A. Bithiophenic MALDI matrices as valuable leads for the selective detection of alkaloids. *Anal. Bioanal. Chem.* **2017,** *409* (29), 6791-6801.

(605) Wu, J.; Cui, C.; Zhao, H.; Zhou, G.; Qin, L.; Li, X.; Chen, L.; Wang, X.; Wan, Y. In-situ detection and imaging of Areca catechu fruit alkaloids by MALDI-MSI. *Ind. Crop. Prod.* **2022,** *188*, 115533.

(606) Yang, S.; Zhan, L.; Liu, C.; Fu, L.; Chen, R.; Nie, Z. Mass spectrometry imaging of small molecule in situ in Lepidium meyenii (Maca) using gold nanoparticles matrix. *Microchem. J.* **2019,** *150*, 104190.

(607) Shiono, K.; Hashizaki, R.; Nakanishi, T.; Sakai, T.; Yamamoto, T.; Ogata, K.; Harada, K. I.; Ohtani, H.; Katano, H.; Taira, S. Multi-imaging of Cytokinin and Abscisic Acid on the Roots of Rice (Oryza sativa) Using Matrix-Assisted Laser Desorption/Ionization Mass Spectrometry. *J. Agric. Food Chem.* **2017,** *65*

(35), 7624-7628.
(608) Shiono, K.; Taira, S. Imaging of Multiple Plant Hormones in Roots of Rice (Oryza sativa) Using Nanoparticle-Assisted Laser Desorption/Ionization Mass Spectrometry. *J. Agric. Food Chem.* **2020,** *68* (24), 6770-6775.
(609) Wang, J.; Sporns, P. MALDI-TOF MS analysis of isoflavones in soy products. *J. Agric. Food Chem.* **2000,** *48* (12), 5887-5892.
(610) Li, L.; Qiu, Z.; Jiang, M.; Zhang, B.; Chen, Q.; Zhang, C.; Zheng, Z.; Qiao, X. Visualizing the Spatial Distribution of Arctium lappa L. Root Components by MALDI-TOF Mass Spectrometry Imaging. *Foods* **2022,** *11* (24), 3957.
(611) Li, F.; Wang, M.; Zhou, J.; Yang, M.; Wang, T. Nanocomposites of boronic acid-functionalized magnetic multi-walled carbon nanotubes with flexible branched polymers as a novel desorption/ionization matrix for the capture and direct detection of cis-diol-flavonoid compounds coupled with MALDI-TOF-MS. *J. Hazard. Mater.* **2022,** *429*, 128055.
(612) Cha, S.; Zhang, H.; Ilarslan, H. I.; Wurtele, E. S.; Brachova, L.; Nikolau, B. J.; Yeung, E. S. Direct profiling and imaging of plant metabolites in intact tissues by using colloidal graphite-assisted laser desorption ionization mass spectrometry. *Plant J.* **2008,** *55* (2), 348-360.
(613) Schiller, J.; Arnhold, J.; Benard, S.; Muller, M.; Reichl, S.; Arnold, K. Lipid analysis by matrix-assisted laser desorption and ionization mass spectrometry: A methodological approach. *Anal. Biochem.* **1999,** *267* (1), 46-56.
(614) Niehaus, M.; Soltwisch, J.; Belov, M. E.; Dreisewerd, K. Transmission-mode MALDI-2 mass spectrometry imaging of cells and tissues at subcellular resolution. *Nat. Methods* **2019,** *16* (9), 925-931.
(615) Stoyanovsky, D. A.; Sparvero, L. J.; Amoscato, A. A.; He, R. R.; Watkins, S.; Pitt, B. R.; Bayir, H.; Kagan, V. E. Improved spatial resolution of matrix-assisted laser desorption/ionization imaging of lipids in the brain by alkylated derivatives of 2,5-dihydroxybenzoic acid. *Rapid Commun. Mass Spectrom.* **2014,** *28* (5), 403-412.
(616) Thomas, A.; Deglon, J.; Lenglet, S.; Mach, F.; Mangin, P.; Wolfender, J. L.; Steffens, S.; Staub, C. High-Throughput Phospholipidic Fingerprinting by Online Desorption of Dried Spots and Quadrupole-Linear Ion Trap Mass Spectrometry: Evaluation of Atherosclerosis Biomarkers in Mouse Plasma. *Anal. Chem.* **2010,** *82* (15), 6687-6694.
(617) Estrada, R.; Yappert, M. C. Alternative approaches for the detection of various phospholipid classes by matrix-assisted laser desorption/ionization time-of-flight mass spectrometry. *J. Mass Spectrom.* **2004,** *39* (4), 412-422.
(618) Steven, R. T.; Race, A. M.; Bunch, J. para-Nitroaniline is a promising matrix for MALDI-MS imaging on intermediate pressure MS systems. *J. Am. Soc. Mass Spectrom.* **2013,** *24* (5), 801-804.
(619) Haiqiang, Y.; Lopez, E.; Young, S. W. Quantitative analysis of free fatty acids in rat plasma using matrix-assisted laser desorption/ionization time-of-Xight mass spectrometry with meso-tetrakis porphyrin as matrix. *Anal. Biochem.* **2006,** *354*, 182-191.
(620) Zhang, Y.; Wang, Y.; Guo, S.; Guo, Y.; Liu, H.; Li, Z. Ammonia-treated N-(1-naphthyl) ethylenediamine dihydrochloride as a novel matrix for rapid quantitative and qualitative determination of serum free fatty acids by matrix-assisted laser desorption/ionization-Fourier transform ion cyclotron resonance mass spectrometry. *Anal. Chim. Acta* **2013,** *794*, 82-89.
(621) Khamidova, N.; Pergande, M. R.; Pathmasiri, K. C.; Khan, R.; Mohr, J. T.; Cologna, S. M. DBDA Matrix Increases Ion Abundance of Fatty Acids and Sulfatides in MALDI-TOF and Mass Spectrometry Imaging Studies. *J. Am. Soc. Mass Spectrom.* **2023,** *34* (8), 1593-1597.

(622) Jun, J. H.; Song, Z.; Liu, Z.; Nikolau, B. J.; Yeung, E. S.; Lee, Y. J. High-spatial and high-mass resolution imaging of surface metabolites of Arabidopsis thaliana by laser desorption-ionization mass spectrometry using colloidal silver. *Anal. Chem.* **2010,** *82* (8), 3255-3265.

(623) Dutta, T.; Stekly, T.; Kucera, L.; Lemr, K. Dual-polarity MALDI mass spectrometry and imaging of oil binders and fatty acids in artworks using cyanographene as a single matrix. *Talanta* **2022,** *242*, 123291.

(624) Liu, Y.; Liu, J.; Deng, C.; Zhang, X. Graphene and graphene oxide: two ideal choices for the enrichment and ionization of long-chain fatty acids free from matrix-assisted laser desorption/ionization matrix interference. *Rapid Commun. Mass Spectrom.* **2011,** *25* (21), 3223-3234.

(625) Hua, Y.; Dagan, S.; Wickramasekara, S.; Boday, D. J.; Wysocki, V. H. Analysis of deprotonated acids with silicon nanoparticle-assisted laser desorption/ ionization mass spectrometry. *J. Mass Spectrom.* **2010,** *45* (12), 1394-1401.

(626) Dufresne, M.; Masson, J. F.; Chaurand, P. Sodium-Doped Gold-Assisted Laser Desorption Ionization for Enhanced Imaging Mass Spectrometry of Triacylglycerols from Thin Tissue Sections. *Anal. Chem.* **2016,** *88* (11), 6018-6025.

(627) Shrivas, K.; Tapadia, K. Ionic liquid matrix-based dispersive liquid-liquid microextraction for enhanced MALDI-MS analysis of phospholipids in soybean. *J. Chromatogr. B Analyt. Technol. Biomed. Life Sci.* **2015,** *1001*, 124-130.

(628) Liu, X.; Chen, Z.; Wang, T.; Jiang, X.; Qu, X.; Duan, W.; Xi, F.; He, Z.; Wu, J. Tissue Imprinting on 2D Nanoflakes-Capped Silicon Nanowires for Lipidomic Mass Spectrometry Imaging and Cancer Diagnosis. *ACS Nano* **2022,** *16* (4), 6916-6928.

(629) Jackson, S. N.; Wang, H. Y.; Woods, A. S. In situ structural characterization of glycerophospholipids and sulfatides in brain tissue using MALDI-MS/MS. *J. Am. Soc. Mass Spectrom.* **2007,** *18* (1), 17-26.

(630) Wang, H. Y.; Jackson, S. N.; Woods, A. S. Direct MALDI-MS analysis of cardiolipin from rat organs sections. *J. Am. Soc. Mass Spectrom.* **2007,** *18* (3), 567-577.

(631) Yang, H.; Jackson, S. N.; Woods, A. S.; Goodlett, D. R.; Ernst, R. K.; Scott, A. J. Streamlined Analysis of Cardiolipins in Prokaryotic and Eukaryotic Samples Using a Norharmane Matrix by MALDI-MSI. *J. Am. Soc. Mass Spectrom.* **2020,** *31* (12), 2495-2502.

(632) Ma, G.; Zhao, X.; Guo, M.; Liu, Y.; Shi, K.; Guo, C.; Pan, Y. 6-Glycosylaminoquinoline-assisted LDI MS for detection and imaging of small molecules with enhanced detection selectivity and sensitivity. *Anal. Chim. Acta* **2022,** *1201*, 339620.

(633) Han, C.; Li, S.; Yue, Q.; Li, N.; Yang, H.; Zhao, Z. Polydopamine-capped AgNPs as a novel matrix overcoming the ion suppression of phosphatidylcholine for MALDI MS comprehensive imaging of glycerophospholipids and sphingolipids in impact-induced injured brain. *Analyst* **2019,** *144* (21), 6304-6312.

(634) Yang, Z.; Chang, Z.; Deng, K.; Gu, J.; Wu, Y.; Sun, Q.; Luo, Q. Reactive Matrices for MALDI-MS of Cholesterol. *Anal. Chem.* **2023,** *95* (46), 16786-16790.

(635) Yu, J.; Kang, Y.; Zhang, H.; Yang, F.; Zhen, H.; Zhu, X.; Wu, T.; Du, Y. A Polymer-Based Matrix for Effective SALDI Analysis of Lipids. *J. Am. Soc. Mass Spectrom.* **2021,** *32* (5), 1189-1195.

(636) Koktava, M.; Valasek, J.; Bezdekova, D.; Prysiazhnyi, V.; Adamova, B.; Benes, P.; Navratilova, J.; Hendrych, M.; Vlcek, P.; Preisler, J.et al. Metal Oxide Laser Ionization Mass Spectrometry Imaging of Fatty Acids and Their Double Bond Positional Isomers. *Anal. Chem.* **2022,** *94* (25), 8928-8936.

(637) Wang, J.; Sporns, P.; Low, N. H. Analysis of food oligosaccharides using MALDI-MS: quantification of fructooligosaccharides. *J. Agric. Food Chem.* **1999,** *47* (4), 1549-1557.

(638) Calvano, C. D.; Cataldi, T. R. I.; Kogel, J. F.; Monopoli, A.; Palmisano, F.; Sundermeyer, J. Structural

Characterization of Neutral Saccharides by Negative Ion MALDI Mass Spectrometry Using a Superbasic Proton Sponge as Deprotonating Matrix. *J. Am. Soc. Mass Spectrom.* **2017,** *28* (8), 1666-1675.

(639) Mernie, E. G.; Tolesa, L. D.; Lee, M. J.; Tseng, M. C.; Chen, Y. J. Direct Oligosaccharide Profiling Using Thin-Layer Chromatography Coupled with Ionic Liquid-Stabilized Nanomatrix-Assisted Laser Desorption-Ionization Mass Spectrometry. *Anal. Chem.* **2019,** *91* (18), 11544-11552.

(640) Paek, J.; Kim, Y.; Lee, D.; Kim, J. MALDI-MS Analysis of Sucrose Using a Charcoal Matrix with Different Cationization Agents. *Bull. Korean Chem. Soc.* **2018,** *39* (6), 750-756.

(641) Lopez-Garcia, M.; Garcia, M. S.; Vilarino, J. M.; Rodriguez, M. V. MALDI-TOF to compare polysaccharide profiles from commercial health supplements of different mushroom species. *Food Chem.* **2016,** *199*, 597-604.

(642) Lin, X.; Xiao, C.; Ling, L.; Guo, L.; Guo, X. A dual-mode reactive matrix for sensitive and quantitative analysis of carbohydrates by MALDI-TOF MS. *Talanta* **2021,** *235*, 122792.

(643) Wang, J.; Zhao, J.; Nie, S.; Xie, M.; Li, S. Rapid profiling strategy for oligosaccharides and polysaccharides by MALDI TOF mass spectrometry. *Food Hydrocoll.* **2022,** *124*, 107237.

(644) Urakami, S.; Hinou, H. Direct MALDI Glycotyping of Glycoproteins toward Practical Subtyping of Biological Samples. *ACS Omega* **2022,** *7* (43), 39280-39286.

(645) Zhao, X.; Huang, Y.; Ma, G.; Liu, Y.; Guo, C.; He, Q.; Wang, H.; Liao, J.; Pan, Y. Parallel On-Target Derivatization for Mass Calibration and Rapid Profiling of N-Glycans by MALDI-TOF MS. *Anal. Chem.* **2020,** *92* (1), 991-998.

(646) Pouria, S.; Corran, P. H.; Smith, A. C.; Smith, H. W.; Hendry, B. M.; Challacombe, S. J.; Tarelli, E. Glycoform composition profiling of O-glycopeptides derived from human serum IgA1 by matrix-assisted laser desorption ionization-time of flight-mass spectrometry. *Anal. Biochem.* **2004,** *330* (2), 257-263.

(647) Nimptsch, K.; Süß, R.; Schnabelrauch, M.; Nimptsch, A.; Schiller, J. Positive ion MALDI-TOF mass spectra are more suitable than negative ion spectra to characterize sulphated glycosaminoglycan oligosaccharides. *Int. J. Mass spectrom.* **2012,** *310*, 72-76.

(648) Dai, Y.; Whittal, R. M.; Bridges, C. A.; Isogai, Y.; Hindsgaul, O.; Li, L. Matrix-assisted laser desorption ionization mass spectrometry for the analysis of monosulfated oligosaccharides. *Carbohydr. Res.* **1997,** *304* (1), 1-9.

(649) Witt, L.; Pirkl, A.; Draude, F.; Peter-Katalinic, J.; Dreisewerd, K.; Mormann, M. Water ice is a soft matrix for the structural characterization of glycosaminoglycans by infrared matrix-assisted laser desorption/ionization. *Anal. Chem.* **2014,** *86* (13), 6439-6446.

(650) Fuchs, B. Analysis of phospolipids and glycolipids by thin-layer chromatography-matrix-assisted laser desorption and ionization mass spectrometry. *J. Chromatogr. A* **2012,** *1259*, 62-73.

(651) Suzuki, Y.; Kabayama, K. Convenient and rapid removal of detergent from glycolipids in detergent-resistant membrane microdomains. *J. Lipid Res.* **2012,** *53* (3), 599-608.

(652) Liu, Y.; Larrouy-Maumus, G. In *Biology of Mycobacterial Lipids*; Elsevier, 2022; p 83-104.

(653) Itonori, S.; Hashimoto, K.; Nakagawa, M.; Harada, M.; Suzuki, T.; Kojima, H.; Ito, M.; Sugita, M. Structural analysis of neutral glycosphingolipids from the silkworm Bombyx mori and the difference in ceramide composition between larvae and pupae. *J. Biochem.* **2018,** *163* (3), 201-214.

(654) Frison-Norrie, S.; Sporns, P. Identification and quantification of flavonol glycosides in almond seedcoats using MALDI-TOF MS. *J. Agric. Food Chem.* **2002,** *50* (10), 2782-2787.

(655) Beck, S.; Stengel, J. Mass spectrometric imaging of flavonoid glycosides and biflavonoids in Ginkgo biloba L. *Phytochemistry* **2016,** *130*, 201-206.

(656) Dreisbach, D.; Petschenka, G.; Spengler, B.; Bhandari, D. R. 3D-surface MALDI mass spectrometry imaging for visualising plant defensive cardiac glycosides in Asclepias curassavica. *Anal. Bioanal. Chem.* **2021,** *413* (8), 2125-2134.
(657) Yoshimura, Y.; Enomoto, H.; Moriyama, T.; Kawamura, Y.; Setou, M.; Zaima, N. Visualization of anthocyanin species in rabbiteye blueberry Vaccinium ashei by matrix-assisted laser desorption/ionization imaging mass spectrometry. *Anal. Bioanal. Chem.* **2012,** *403* (7), 1885-1895.
(658) Petroselli, G.; Parapugna, T. L.; Lagorio, M. G.; Erra-Balsells, R. MALDI- and LDI-MS saponin fingerprint of leaves and stick components of commercial yerba mate (Ilex paraguariensis). *J. Mass Spectrom.* **2019,** *54* (2), 195-203.
(659) Pislyagin, E. A.; Dmitrenok, P. S.; Gorpenchenko, T. Y.; Avilov, S. A.; Silchenko, A. S.; Aminin, D. L. Determination of cucumarioside A(2)-2 in mouse spleen by radiospectroscopy, MALDI-MS and MALDI-IMS. *Eur. J. Pharm. Sci.* **2013,** *49* (4), 461-467.
(660) Tang, K.; Taranenko, N. I.; Allman, S. L.; Chang, L. Y.; Chen, C. H. Detection of 500-nucleotide DNA by laser desorption mass spectrometry. *Rapid Commun. Mass Spectrom.* **1994,** *8* (9), 727-730.
(661) Fu, Y.; Xu, S.; Pan, C.; Ye, M.; Zou, H. 3,4-diaminobenzophenone matrix for analysis of oligonucleotides by MALDI-TOF mass spectrometry. *Curr. Protoc. Nucleic Acid Chem.* **2007,** *Chapter 10*, Unit 10 12.
(662) Hagan, N. A.; Smith, C. A.; Antoine, M. D.; Lin, J. S.; Feldman, A. B.; Demirev, P. A. Enhanced in-source fragmentation in MALDI-TOF-MS of oligonucleotides using 1,5-diaminonapthalene. *J. Am. Soc. Mass Spectrom.* **2012,** *23* (4), 773-777.
(663) Li, Y. C. L.; Cheng, S.-w.; Chan, T. W. D. Evaluation of ammonium salts as co-matrices for matrix-assisted laser desorption/ionization mass spectrometry of oligonucleotides. *Rapid Commun. Mass Spectrom.* **1998,** *12* (15), 993-998.
(664) Currie, G. J.; Yates, J. R. Analysis of oligodeoxynucleotides by negative-ion matrix-assisted laser desorption mass spectrometry. *J. Am. Soc. Mass Spectrom.* **1993,** *4* (12), 955-963.
(665) Spengler, B.; Pan, Y.; Cotter, R. J.; Kan, L. S. Molecular weight determination of underivatized oligodeoxyribonucleotides by positive-ion matrix-assisted ultraviolet laser-desorption mass spectrometry. *Rapid Commun. Mass Spectrom.* **1990,** *4* (4), 99-102.
(666) Nordhoff, E.; Ingendoh, A.; Cramer, R.; Overberg, A.; Stahl, B.; Karas, M.; Hillenkamp, F.; Crain, P. F. Matrix-assisted laser desorption/ionization mass spectrometry of nucleic acids with wavelengths in the ultraviolet and infrared. *Rapid Commun. Mass Spectrom.* **1992,** *6* (12), 771-776.
(667) Berkenkamp, S.; Kirpekar, F.; Hillenkamp, F. Infrared MALDI mass spectrometry of large nucleic acids. *Science* **1998,** *281* (5374), 260-262.
(668) Taranenko, N. I.; Tang, K.; Allman, S. L.; Ch'ang, L. Y.; Chen, C. H. 3-Aminopicolinic acid as a matrix for laser desorption mass spectrometry of biopolymers. *Rapid Commun. Mass Spectrom.* **1994,** *8* (12), 1001-1006.
(669) Kong, X.; Huang, L. C.; Liau, S. C.; Han, C. C.; Chang, H. C. Polylysine-coated diamond nanocrystals for MALDI-TOF mass analysis of DNA oligonucleotides. *Anal. Chem.* **2005,** *77* (13), 4273-4277.
(670) Hong, M.; Zhou, X.; Li, J.; Tian, Y.; Zhu, J. Nanoscale architecture dictates detection profile of surface-confined DNA by MALDI-TOF MS. *Anal. Chem.* **2009,** *81* (21), 8839-8845.
(671) Shahgholi, M.; Garcia, B. A.; Chiu, N. H.; Heaney, P. J.; Tang, K. Sugar additives for MALDI matrices improve signal allowing the smallest nucleotide change (A:T) in a DNA sequence to be resolved. *Nucleic Acids Res.* **2001,** *29* (19), E91.
(672) Kuang, F. Y.; Hu, D. J.; Wang, L.; Chen, F.; Lv, G. P. Ti-based MOF nanosheets as a mass spectrometry imaging matrix for low molecular weight compounds to reveal the spatiotemporal content changes of

hepatotoxic components during the processing of Polygonum multiflorum. *Analyst* **2024,** *150* (1), 120-130.
(673) Li, Y.; Wang, Y.; Ma, D.; Ding, C. F.; Yan, Y. MOF-Derived Erythrocyte-like CuO/Cu(2)O@ZnO-CN Nanomaterials for Enhanced Laser Desorption/Ionization Mass Spectrometric Detection of Saccharides and Glucose Quantification in Diabetic Patient Serum. *Anal. Chem.* **2025,** *97* (46), 25635-25642.
(674) Liu, X. P.; Sun, W. Q.; Zhao, M. G.; Zhang, X. J.; Liu, L. H.; Chen, C. P. Fluoro-functionalized ionic covalent organic frameworks (F-iCOFs) for highly selective enrichment and sensitive determination of perfluorinated sulfonates by MALDI-MS. *Mikrochim. Acta* **2022,** *189* (12), 442.
(675) Yan, L.; Zheng, W.; Lin, Z. Covalent Organic Framework Nanofilm-Assisted Laser Desorption Ionization Mass Spectrometry for the Determination of Benzophenone Derivatives in Personal Care Products. *Rapid Commun. Mass Spectrom.* **2025,** *39* (10), e10009.
(676) Liu, X.; Zhao, M.; Wang, L.; Chen, Y.; Niu, G.; Zhang, X.; Liu, L.; Chen, C. Ultrasensitive quantification of illegal cationic dyes in foods with low matrix effects: Carboxyl-functionalized COFs as novel adsorbents and MALDI-TOF MS matrices. *Microchem. J.* **2025,** *219*, 116207.
(677) Wang, H.; Zhao, X.; Huang, Y.; Liao, J.; Liu, Y.; Pan, Y. Rapid quality control of medicine and food dual purpose plant polysaccharides by matrix assisted laser desorption/ionization mass spectrometry. *Analyst* **2020,** *145* (6), 2168-2175.
(678) Zhao, Y.-Z.; Xu, Y.; Gong, C.; Ju, Y.-R.; Liu, Z.-X.; Xu, X. Analysis of Small Molecule Compounds by Matrix-assisted Laser Desorption Ionization Mass Spectrometry with Fe3O4 Nanoparticles as Matrix. *Chinese J. Anal. Chem.* **2021,** *49* (1), 103-112.
(679) Nezhad, Z. S.; Salazar, J. P.; Pryce, R. S.; Munter, L. M.; Chaurand, P. Absolute quantification of cholesterol from thin tissue sections by silver-assisted laser desorption ionization mass spectrometry imaging. *Anal. Bioanal. Chem.* **2022,** *414* (23), 6947-6954.
(680) Chiang, C. K.; Lin, Y. W.; Chen, W. T.; Chang, H. T. Accurate quantitation of glutathione in cell lysates through surface-assisted laser desorption/ionization mass spectrometry using gold nanoparticles. *Nanomedicine* **2010,** *6* (4), 530-537.
(681) Hu, C. C.; Huang, M. F.; Chang, H. T. Quantitative surface-assisted laser desorption/ionization-MS approaches for bioanalysis. *Bioanalysis* **2013,** *5* (6), 633-635.
(682) Cho, H. S.; Koh, J.; Yim, G.; Jang, H.; Kim, Y. K. Laser Desorption/Ionization on Au@TiO(2) Core@Shell Nanostars for Mass Spectrometric Analysis of Small Molecules. *Nanomaterials (Basel)* **2024,** *14* (23), 1946.
(683) Wang, X. N.; Tang, W.; Gordon, A.; Wang, H. Y.; Xu, L.; Li, P.; Li, B. Porous TiO(2) Film Immobilized with Gold Nanoparticles for Dual-Polarity SALDI MS Detection and Imaging. *ACS Appl. Mater. Interfaces* **2020,** *12* (38), 42567-42575.
(684) Kailasa, S. K.; Wu, H. F. Functionalized quantum dots with dopamine dithiocarbamate as the matrix for the quantification of efavirenz in human plasma and as affinity probes for rapid identification of microwave tryptic digested proteins in MALDI-TOF-MS. *J. Proteomics* **2012,** *75* (10), 2924-2933.
(685) Ho, Y. C.; Tseng, M. C.; Lu, Y. W.; Lin, C. C.; Chen, Y. J.; Fuh, M. R. Nanoparticle-assisted MALDI-TOF MS combined with seed-layer surface preparation for quantification of small molecules. *Anal. Chim. Acta* **2011,** *697* (1-2), 1-7.
(686) Li, B.; Gao, W.; Ling, L.; Yu, S. Enzyme-assisted ReMALDI-MS assay for quantification of cholesterol in food. *Food Chem.* **2022,** *383*, 132444.
(687) Han, Y.; Qu, X.; Geng, H.; Wang, L.; Zhu, Z.; Zhang, Y.; Cui, X.; Lu, H.; Wang, X.; Chen, P.et al. Isotope-Coded On-Tissue Derivatization for Quantitative Mass Spectrometry Imaging of Short-Chain Fatty

Acids in Biological Tissues. *Anal. Chem.* **2023,** *95* (48), 17622-17628.

(688) Jadoul, L.; Smargiasso, N.; Pamelard, F.; Alberts, D.; Noel, A.; De Pauw, E.; Longuespee, R. An Improved Molecular Histology Method for Ion Suppression Monitoring and Quantification of Phosphatidyl Cholines During MALDI MSI Lipidomics Analyses. *OMICS* **2016,** *20* (2), 110-121.

(689) Jadoul, L.; Longuespee, R.; Noel, A.; De Pauw, E. A spiked tissue-based approach for quantification of phosphatidylcholines in brain section by MALDI mass spectrometry imaging. *Anal. Bioanal. Chem.* **2015,** *407* (8), 2095-2106.

(690) Kaneshiro, K.; Watanabe, M.; Terasawa, K.; Uchimura, H.; Fukuyama, Y.; Iwamoto, S.; Sato, T. A.; Shimizu, K.; Tsujimoto, G.; Tanaka, K. Rapid quantitative profiling of N-glycan by the glycan-labeling method using 3-aminoquinoline/alpha-cyano-4-hydroxycinnamic acid. *Anal. Chem.* **2012,** *84* (16), 7146-7151.

(691) Zhou, S.; Liao, J.; Jiang, K.; Wang, H.; Liu, Y.; Xiong, H.; Wang, P.; Pan, Y.; Feng, H. Rapid and Non-Targeted Qualitative and Quantitative Detection of miRNA in Complex Biological Samples Using Matrix-Assisted Laser Desorption/Ionization Mass Spectrometry with a 3-Aminoquinoline and 2',4',6'-Trihydroxyacetophenone Ionic Liquid Matrix. *J. Am. Soc. Mass Spectrom.* **2025,** *36* (3), 495-503.

(692) Zhang, Y.; Wang, J.; Liu, J.; Han, J.; Xiong, S.; Yong, W.; Zhao, Z. Combination of ESI and MALDI mass spectrometry for qualitative, semi-quantitative and in situ analysis of gangliosides in brain. *Sci. Rep.* **2016,** *6*, 25289.

(693) Snovida, S. I.; Rak-Banville, J. M.; Perreault, H. On the use of DHB/aniline and DHB/N,N-dimethylaniline matrices for improved detection of carbohydrates: automated identification of oligosaccharides and quantitative analysis of sialylated glycans by MALDI-TOF mass spectrometry. *J. Am. Soc. Mass Spectrom.* **2008,** *19* (8), 1138-1146.

(694) Leipert, J.; Treitz, C.; Leippe, M.; Tholey, A. Identification and Quantification of N-Acyl Homoserine Lactones Involved in Bacterial Communication by Small-Scale Synthesis of Internal Standards and Matrix-Assisted Laser Desorption/Ionization Mass Spectrometry. *J. Am. Soc. Mass Spectrom.* **2017,** *28* (12), 2538-2547.

(695) Stauber, J.; El Ayed, M.; Wisztorski, M.; Day, R.; Fournier, I.; Salzet, M. Polymerase chain reaction and immunoassay--matrix assisted laser desorption mass spectrometry using tag-mass technology: new tools to break down quantification limits and multiplexes. *Anal. Chem.* **2009,** *81* (22), 9512-9521.

(696) Nikolopoulou, V.; Stem, A.; Vasiliou, V.; Aalizadeh, R. Investigating the Quantitative Structure-Ionization Efficiency Relationship of Small Molecules and Lipids in the Presence of Ammonium Fluoride in MALDI-TIMS-QTOF Mass Spectrometry Imaging. *J. Am. Soc. Mass Spectrom.* **2025,** *36* (11), 2460-2469.

(697) Ding, Y.; Kawakita, K.; Xu, J.; Akiyama, K.; Fujino, T. Analyte-Size-Dependent Ionization and Quantification of Monosaccharides in Human Plasma Using Cation-Exchanged Smectite Layers. *Anal. Chem.* **2015,** *87* (15), 7944-7950.

(698) Jaskolla, T. W.; Onischke, K.; Schiller, J. 2,5-dihydroxybenzoic acid salts for matrix-assisted laser desorption/ionization time-of-flight mass spectrometric lipid analysis: simplified spectra interpretation and insights into gas-phase fragmentation. *Rapid Commun. Mass Spectrom.* **2014,** *28* (12), 1353-1363.

(699) Gusev, A. I.; Wilkinson, W. R.; Proctor, A.; Hercules, D. M. Direct quantitative analysis of peptides using matrix assisted laser desorption ionization. *Anal. Bioanal. Chem.* **1996,** *354* (4), 455-463.

(700) Sugiyama, E.; Masaki, N.; Matsushita, S.; Setou, M. Ammonium Sulfate Improves Detection of Hydrophilic Quaternary Ammonium Compounds through Decreased Ion Suppression in Matrix-Assisted Laser Desorption/Ionization Imaging Mass Spectrometry. *Anal. Chem.* **2015,** *87* (22), 11176-11181.

(701) Harvey, D. J. Quantitative aspects of the matrix-assisted laser desorption mass spectrometry of complex oligosaccharides. *Rapid Commun. Mass Spectrom.* **1993,** *7* (7), 614-619.

(702) Wang, C.; Wu, Y.; Zhang, L.; Liu, B. F.; Lin, Y.; Liu, X. Relative quantitation of neutral and sialylated N-glycans using stable isotopic labeled d0/d5-benzoyl chloride by MALDI-MS. *Anal. Chim. Acta* **2018,** *1002*, 50-61.

(703) Jeong, H. J.; Kim, Y. G.; Yang, Y. H.; Kim, B. G. High-throughput quantitative analysis of total N-glycans by matrix-assisted laser desorption/ionization time-of-flight mass spectrometry. *Anal. Chem.* **2012,** *84* (7), 3453-3460.

(704) Zhou, H.; Warren, P. G.; Froehlich, J. W.; Lee, R. S. Dual modifications strategy to quantify neutral and sialylated N-glycans simultaneously by MALDI-MS. *Anal. Chem.* **2014,** *86* (13), 6277-6284.

(705) Fujiwaki, T.; Tasaka, M.; Yamaguchi, S. Quantitative evaluation of sphingomyelin and glucosylceramide using matrix-assisted laser desorption ionization time-of-flight mass spectrometry with sphingosylphosphorylcholine as an internal standard. Practical application to tissues from patients with Niemann-Pick disease types A and C, and Gaucher disease. *J. Chromatogr. B Analyt. Technol. Biomed. Life Sci.* **2008,** *870* (2), 170-176.

(706) Landgraf, R. R.; Garrett, T. J.; Conaway, M. C.; Calcutt, N. A.; Stacpoole, P. W.; Yost, R. A. Considerations for quantification of lipids in nerve tissue using matrix-assisted laser desorption/ionization mass spectrometric imaging. *Rapid Commun. Mass Spectrom.* **2011,** *25* (20), 3178-3184.

(707) Wei, Y.; Li, S.; Wang, J.; Shu, C.; Liu, J.; Xiong, S.; Song, J.; Zhang, J.; Zhao, Z. Polystyrene spheres-assisted matrix-assisted laser desorption ionization mass spectrometry for quantitative analysis of plasma lysophosphatidylcholines. *Anal. Chem.* **2013,** *85* (9), 4729-4734.

(708) Vandenbosch, M.; Mutuku, S. M.; Mantas, M. J. Q.; Patterson, N. H.; Hallmark, T.; Claesen, M.; Heeren, R. M. A.; Hatcher, N. G.; Verbeeck, N.; Ekroos, K.et al. Toward Omics-Scale Quantitative Mass Spectrometry Imaging of Lipids in Brain Tissue Using a Multiclass Internal Standard Mixture. *Anal. Chem.* **2023,** *95* (51), 18719-18730.

(709) Fresnais, M.; Yildirim, E.; Karabulut, S.; Jager, D.; Zornig, I.; Benzel, J.; Pajtler, K. W.; Pfister, S. M.; Burhenne, J.; Haefeli, W. E.et al. Rapid MALDI-MS Assays for Drug Quantification in Biological Matrices: Lessons Learned, New Developments, and Future Perspectives. *Molecules* **2021,** *26* (5), 1281.

(710) Fresnais, M.; Muck, A.; Majewsky, M.; Statz, B.; Krausert, S.; Benzel, J.; Castel, D.; Le Dret, L.; Pfister, S.; Haefeli, W. E.et al. Rapid and Sensitive Drug Quantification in Tissue Sections Using Matrix Assisted Laser Desorption Ionization-Ion Mobility-Mass Spectrometry Profiling. *J. Am. Soc. Mass Spectrom.* **2020,** *31* (3), 742-751.

(711) Ling, Y. C.; Lin, L.; Chen, Y. T. Quantitative analysis of antibiotics by matrix-assisted laser desorption/ionization time-of-flight mass spectrometry. *Rapid Commun. Mass Spectrom.* **1998,** *12* (6), 317-327.

(712) Wang, J.; Sporns, P. MALDI-TOF MS quantification of coccidiostats in poultry feeds. *J. Agric. Food Chem.* **2000,** *48* (7), 2807-2811.

(713) Rao, T.; Shen, B.; Zhu, Z.; Shao, Y.; Kang, D.; Li, X.; Yin, X.; Li, H.; Xie, L.; Wang, G.et al. Optimization and evaluation of MALDI TOF mass spectrometric imaging for quantification of orally dosed octreotide in mouse tissues. *Talanta* **2017,** *165*, 128-135.

(714) Takai, N.; Tanaka, Y.; Watanabe, A.; Saji, H. Quantitative imaging of a therapeutic peptide in biological tissue sections by MALDI MS. *Bioanalysis* **2013,** *5* (5), 603-612.

(715) Bae, Y. J.; Park, K. M.; Ahn, S. H.; Moon, J. H.; Kim, M. S. Spectral reproducibility and quantification

of peptides in MALDI of samples prepared by micro-spotting. *J. Am. Soc. Mass Spectrom.* **2014,** *25* (8), 1502-1505.

(716) Griffin, T. J.; Gygi, S. P.; Rist, B.; Aebersold, R.; Loboda, A.; Jilkine, A.; Ens, W.; Standing, K. G. Quantitative proteomic analysis using a MALDI quadrupole time-of-flight mass spectrometer. *Anal. Chem.* **2001,** *73* (5), 978-986.

(717) Kurogochi, M.; Amano, J. Relative quantitation of glycopeptides based on stable isotope labeling using MALDI-TOF MS. *Molecules* **2014,** *19* (7), 9944-9961.

(718) Xu, H.; Liu, M.; Huang, X.; Min, Q.; Zhu, J. J. Multiplexed Quantitative MALDI MS Approach for Assessing Activity and Inhibition of Protein Kinases Based on Postenrichment Dephosphorylation of Phosphopeptides by Metal-Organic Framework-Templated Porous CeO(2). *Anal. Chem.* **2018,** *90* (16), 9859-9867.

(719) Chen, J.; Li, H. F.; Zhao, G.; Lin, J. M.; He, X. Matrix-assisted laser desorption ionization mass spectrometry based quantitative analysis of cordycepin from Cordyceps militaris. *J. Pharm. Anal.* **2021,** *11* (4), 499-504.

(720) Li, S.; Ng, T. T.; Yao, Z. P. Quantitative analysis of blended oils by matrix-assisted laser desorption/ionization mass spectrometry and partial least squares regression. *Food Chem.* **2021,** *334*, 127601.

(721) Li, S.; Lin, X.; Ng, T. T.; Yao, Z. P. Quantitative Analysis of Blended Oils Based on Intensity Ratios of Marker Ions in MALDI-MS Spectra. *J. Agric. Food Chem.* **2024,** *72* (27), 15376-15386.

(722) Pirman, D. A.; Reich, R. F.; Kiss, A.; Heeren, R. M.; Yost, R. A. Quantitative MALDI tandem mass spectrometric imaging of cocaine from brain tissue with a deuterated internal standard. *Anal. Chem.* **2013,** *85* (2), 1081-1089.

(723) Wang, L.; Han, Y.; Zhang, Y.; Geng, H.; Zhu, Z.; Chen, P.; Cui, X.; Wang, X.; Sun, C. In-depth profiling of carbohydrate isomers in biological tissues by chemical derivatization-assisted mass spectrometry imaging. *Anal. Chim. Acta* **2023,** *1278*, 341741.

(724) Ostermann, K. M.; Dieplinger, R.; Lutsch, N. M.; Strupat, K.; Metz, T. F.; Mechtler, T. P.; Kasper, D. C. Matrix-assisted laser desorption/ionization for simultaneous quantitation of (acyl-)carnitines and organic acids in dried blood spots. *Rapid Commun. Mass Spectrom.* **2013,** *27* (13), 1497-1504.

(725) Niklas, J.; Hollemeyer, K.; Heinzle, E. High-throughput phospholipid quantitation in mammalian cells using matrix-assisted laser desorption ionization-time of flight mass spectrometry with N-trifluoroacetyl-phosphatidylethanolamine as internal standard. *Anal. Biochem.* **2011,** *419* (2), 351-353.

(726) Sugiyama, E.; Hara, A.; Uemura, K. A quantitative analysis of serum sulfatide by matrix-assisted laser desorption ionization time-of-flight mass spectrometry with delayed ion extraction. *Anal. Biochem.* **1999,** *274* (1), 90-97.

(727) Tuerk, R. D.; Auchli, Y.; Thali, R. F.; Scholz, R.; Wallimann, T.; Brunisholz, R. A.; Neumann, D. Tracking and quantification of 32P-labeled phosphopeptides in liquid chromatography matrix-assisted laser desorption/ionization mass spectrometry. *Anal. Biochem.* **2009,** *390* (2), 141-148.

(728) Pabst, M.; Fagerer, S. R.; Kohling, R.; Kuster, S. K.; Steinhoff, R.; Badertscher, M.; Wahl, F.; Dittrich, P. S.; Jefimovs, K.; Zenobi, R. Self-aliquoting microarray plates for accurate quantitative matrix-assisted laser desorption/ionization mass spectrometry. *Anal. Chem.* **2013,** *85* (20), 9771-9776.

(729) Sobsey, C. A.; Froehlich, B.; Batist, G.; Borchers, C. H. Immuno-MALDI-MS for Accurate Quantitation of Targeted Peptides from Volume-Restricted Samples. *Methods Mol. Biol.* **2022,** *2515*, 203-225.

(730) Rubakhin, S. S.; Sweedler, J. V. Quantitative measurements of cell-cell signaling peptides with single-cell MALDI MS. *Anal. Chem.* **2008,** *80* (18), 7128-7136.

(731) Gobom, J.; Kraeuter, K. O.; Persson, R.; Steen, H.; Roepstorff, P.; Ekman, R. Detection and quantification of neurotensin in human brain tissue by matrix-assisted laser desorption/ionization time-of-flight mass spectrometry. *Anal. Chem.* **2000,** *72* (14), 3320-3326.
(732) Yoneyama, T.; Ohtsuki, S.; Tachikawa, M.; Uchida, Y.; Terasaki, T. Scrambled Internal Standard Method for High-Throughput Protein Quantification by Matrix-Assisted Laser Desorption Ionization Tandem Mass Spectrometry. *J. Proteome Res.* **2017,** *16* (4), 1556-1565.
(733) Froehlich, B. C.; Popp, R.; Sobsey, C. A.; Ibrahim, S.; LeBlanc, A.; Mohammed, Y.; Buchanan, M.; Aguilar-Mahecha, A.; Potz, O.; Chen, M. X.et al. A multiplexed, automated immuno-matrix assisted laser desorption/ionization mass spectrometry assay for simultaneous and precise quantitation of PTEN and p110alpha in cell lines and tumor tissues. *Analyst* **2021,** *146* (21), 6566-6575.
(734) Yang, Q.; Liu, C.; Qi, K.; Xiong, Y.; Pan, Y.; Tian, C. Imaging and quantification of neuropeptides in mouse pituitary tissue by atmospheric pressure matrix-assisted laser desorption/ionization mass spectrometry. *Rapid Commun. Mass Spectrom.* **2024,** *38* (12), e9755.
(735) Ferranti, P.; Nasi, A.; Bruno, M.; Basile, A.; Serpe, L.; Gallo, P. A peptidomic approach for monitoring and characterising peptide cyanotoxins produced in Italian lakes by matrix-assisted laser desorption/ionisation and quadrupole time-of-flight mass spectrometry. *Rapid Commun. Mass Spectrom.* **2011,** *25* (9), 1173-1183.
(736) Li, H.; Popp, R.; Frohlich, B.; Chen, M. X.; Borchers, C. H. Peptide and Protein Quantification Using Automated Immuno-MALDI (iMALDI). *J. Vis. Exp.* **2017,** (126), 55933.
(737) Bansal, S. S.; Halket, J. M.; Fusova, J.; Bomford, A.; Simpson, R. J.; Vasavda, N.; Thein, S. L.; Hider, R. C. Quantification of hepcidin using matrix-assisted laser desorption/ionization time-of-flight mass spectrometry. *Rapid Commun. Mass Spectrom.* **2009,** *23* (11), 1531-1542.
(738) Tang, X.; Sadeghi, M.; Olumee, Z.; Vertes, A.; Braatz, J. A.; McIlwain, L. K.; Dreifuss, P. A. Detection and quantitation of beta-2-microglobulin glycosylated end products in human serum by matrix-assisted laser desorption/ionization mass spectrometry. *Anal. Chem.* **1996,** *68* (21), 3740-3745.
(739) Mirgorodskaya, O. A.; Korner, R.; Novikov, A.; Roepstorff, P. Absolute quantitation of proteins by a combination of acid hydrolysis and matrix-assisted laser desorption/ionization mass spectrometry. *Anal. Chem.* **2004,** *76* (13), 3569-3575.
(740) Cruz Villarreal, J.; Kruithoff, R.; Egatz-Gomez, A.; Coleman, P. D.; Ros, R.; Sandrin, T. R.; Ros, A. MIMAS: microfluidic platform in tandem with MALDI mass spectrometry for protein quantification from small cell ensembles. *Anal. Bioanal. Chem.* **2022,** *414* (13), 3945-3958.
(741) Yang, M.; Cruz Villarreal, J.; Ariyasinghe, N.; Kruithoff, R.; Ros, R.; Ros, A. Quantitative Approach for Protein Analysis in Small Cell Ensembles by an Integrated Microfluidic Chip with MALDI Mass Spectrometry. *Anal. Chem.* **2021,** *93* (15), 6053-6061.
(742) Jakoby, T.; Tholey, A.; van den Berg, B. H. Improved reporter ion assignment of raw isobaric stable isotope labeled liquid chromatography/matrix-assisted laser desorption/ionization tandem time-of-flight mass spectral data for quantitative proteomics. *Rapid Commun. Mass Spectrom.* **2012,** *26* (23), 2777-2785.
(743) Zhang, J.; Zhang, L.; Zhou, Y.; Guo, Y. L. A novel pyrimidine-based stable-isotope labeling reagent and its application to quantitative analysis using matrix-assisted laser desorption/ionization mass spectrometry. *J. Mass Spectrom.* **2007,** *42* (11), 1514-1521.
(744) Yang, C.; Lee, H. K.; Zhang, Y.; Jiang, L. L.; Chen, Z. F.; Chung, A. C. K.; Cai, Z. In Situ Detection and Imaging of PFOS in Mouse Kidney by Matrix-Assisted Laser Desorption/Ionization Imaging Mass Spectrometry. *Anal. Chem.* **2019,** *91* (14), 8783-8788.

(745) Wu, N.; Jiao, L.; Butikofer, M.; Zeng, Z.; Zenobi, R. High-Mass Matrix-Assisted Laser Desorption/Ionization Mass Spectrometry for Absolute Quantitation of Noncovalent Protein-Protein Binding Interactions. *Anal. Chem.* **2021,** *93* (31), 10982-10989.

(746) Chen, S. H.; Liao, H. K.; Chang, C. Y.; Juo, C. G.; Chen, J. H.; Chan, S. I.; Chen, Y. J. Targeted protein quantitation and profiling using PVDF affinity probe and MALDI-TOF MS. *Proteomics* **2007,** *7* (17), 3038-3050.

(747) Hattan, S. J.; Parker, K. C.; Vestal, M. L.; Yang, J. Y.; Herold, D. A.; Duncan, M. W. Analysis and Quantitation of Glycated Hemoglobin by Matrix Assisted Laser Desorption/Ionization Time of Flight Mass Spectrometry. *J. Am. Soc. Mass Spectrom.* **2016,** *27* (3), 532-541.

(748) Shroff, R.; Schramm, K.; Jeschke, V.; Nemes, P.; Vertes, A.; Gershenzon, J.; Svatos, A. Quantification of plant surface metabolites by matrix-assisted laser desorption-ionization mass spectrometry imaging: glucosinolates on Arabidopsis thaliana leaves. *Plant J.* **2015,** *81* (6), 961-972.

(749) Nakayama, K.; Li, X.; Shimizu, K.; Akamatsu, S.; Inoue, T.; Kobayashi, T.; Ogawa, O.; Goto, T. qShot MALDI analysis: A rapid, simple, convenient, and reliable quantitative phospholipidomics approach using MALDI-TOF/MS. *Talanta* **2023,** *254*, 124099.

(750) Serna, J.; Garcia-Seisdedos, D.; Alcazar, A.; Lasuncion, M. A.; Busto, R.; Pastor, O. Quantitative lipidomic analysis of plasma and plasma lipoproteins using MALDI-TOF mass spectrometry. *Chem. Phys. Lipids* **2015,** *189*, 7-18.

(751) Ren, H.; Chen, W.; Wang, H.; Kang, Y.; Zhu, X.; Li, J.; Wu, T.; Du, Y. Quantitative analysis of free fatty acids in gout by disposable paper-array plate based MALDI MS. *Anal. Biochem.* **2019,** *579*, 38-43.

(752) van Kampen, J. J.; Burgers, P. C.; Gruters, R. A.; Osterhaus, A. D.; de Groot, R.; Luider, T. M.; Volmer, D. A. Quantitative analysis of antiretroviral drugs in lysates of peripheral blood mononuclear cells using MALDI-triple quadrupole mass spectrometry. *Anal. Chem.* **2008,** *80* (13), 4969-4975.

(753) Zhan, L.; Xie, X.; Li, Y.; Liu, H.; Xiong, C.; Nie, Z. Differentiation and Relative Quantitation of Disaccharide Isomers by MALDI-TOF/TOF Mass Spectrometry. *Anal. Chem.* **2018,** *90* (3), 1525-1530.

(754) Schwaiger-Haber, M.; Stancliffe, E.; Anbukumar, D. S.; Sells, B.; Yi, J.; Cho, K.; Adkins-Travis, K.; Chheda, M. G.; Shriver, L. P.; Patti, G. J. Using mass spectrometry imaging to map fluxes quantitatively in the tumor ecosystem. *Nat. Commun.* **2023,** *14* (1), 2876.

(755) Bruenner, B. A.; Yip, T. T.; Hutchens, T. W. Quantitative analysis of oligonucleotides by matrix-assisted laser desorption/ionization mass spectrometry. *Rapid Commun. Mass Spectrom.* **1996,** *10* (14), 1797-1801.
